# Divergent Catalysis: Catalytic Asymmetric [4+2] Cycloaddition of Palladium Enolates

**DOI:** 10.1021/jacs.3c02104

**Published:** 2023-05-15

**Authors:** Kaylin N. Flesch, Alexander Q. Cusumano, Peng-Jui Chen, Christian Santiago Strong, Stephen R. Sardini, Yun E. Du, Michael D. Bartberger, William A. Goddard, Brian M. Stoltz

**Affiliations:** † The Warren and Katharine Schlinger Laboratory for Chemistry and Chemical Engineering, Division of Chemistry and Chemical Engineering, California Institute of Technology, Pasadena, California 91125, United States; ‡ Alterome Therapeutics, San Diego, California 92127, United States; § Materials and Process Simulation Center, Beckman Institute, California Institute of Technology, Pasadena, California 91125, United States

## Abstract

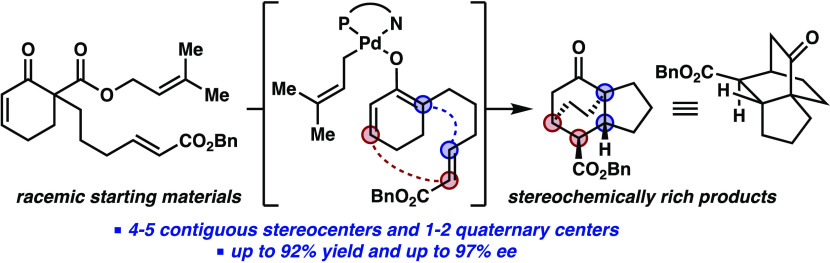

An asymmetric decarboxylative [4+2] cycloaddition from a catalytically generated chiral Pd enolate was developed, forging four contiguous stereocenters in a single transformation. This was achieved through a strategy termed divergent catalysis, wherein departure from a known catalytic cycle enables novel reactivity of a targeted intermediate prior to re-entry into the original cycle. Mechanistic studies including quantum mechanics calculations, Eyring analysis, and KIE studies offer insight into the reaction mechanism.

## 1 Introduction

Enantioselective construction of all-carbon quaternary stereogenic centers represents a central and ongoing challenge in synthetic organic chemistry.^1^ The asymmetric allylic alkylation of enolate nucleophiles serves as a powerful strategy for accessing such motifs.^2^

**Figure 1:**
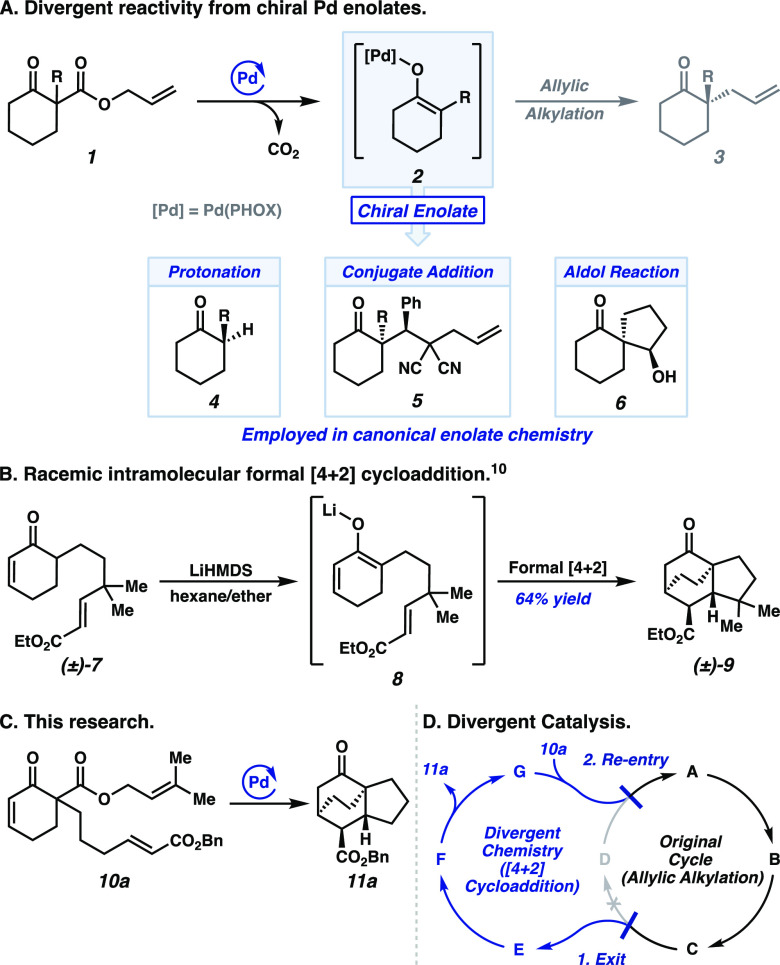
(A) Examples of chiral Pd enolate reactivity. (B) Lithium base-promoted intramolecular formal [4+2] cycloaddition. (C) Proposed asymmetric intramolecular [4+2] reaction. (D) Divergent catalytic cycle.

A unique aspect of the Pd-catalyzed allylic alkylation methods developed by our group is the inner-sphere reductive elimination from a chiral O-bound Pd enolate intermediate (**2**), yielding enantioenriched ketones (**3**) (Figure 1A).^3,4^ This intermediate is generated catalytically from achiral or racemic enolate precursors, such as allyl enol carbonates^5^ and β-ketoesters^6^ (**1**). The Pd enolate is accessed in the absence of a base, under neutral conditions, and in a regiospecific fashion. Conversely, canonical conditions for enolate formation are plagued by regioselectivity challenges and typically require the use of a strong base or Lewis acid. Given the inherent advantages of Pd enolates, we sought to exploit their reactivity beyond simple allylic alkylations in more general asymmetric transformations.

Highlighting the utility of this concept, our lab has demonstrated the enantioselective protonation of Pd enolates as a valuable strategy to access ketones with tertiary stereocenters (**4**).^7^ Building upon this success, we subsequently developed methods to construct quaternary centers via enantioselective conjugate additions^8^ (**5**) and intramolecular aldol reactions (**6**).^9^ Taken together, these advances underscore the feasibility of employing Pd enolates as stereogenic nucleophiles.

In a unique example of enolate reactivity, Fukumoto and co-workers reported a formal [4+2] reaction from in situ-generated conjugated lithium enolate **8**, forging tricyclic adduct **9** in a racemic fashion (Figure 1B).^10^ We envisioned that an analogous asymmetric transformation would be tractable from a chiral, conjugated Pd enolate—derived from the decarboxylation of unsaturated β-ketoester **10a** using an asymmetric ligand on Pd (Figure 1C).

**Scheme 1:**
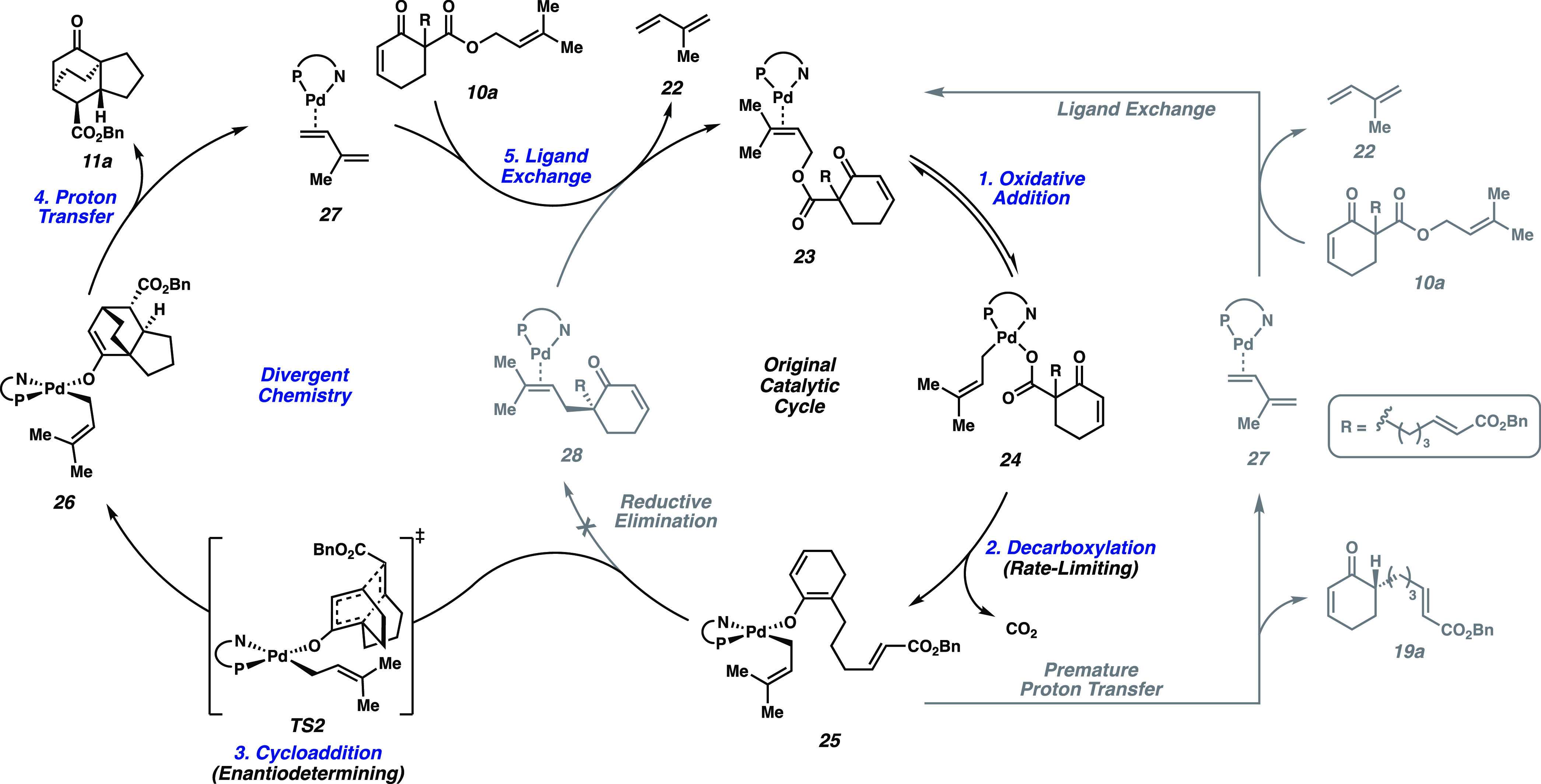
Proposed Divergent Catalytic Cycle Undesired reaction pathways in gray.

To realize this transformation, we sought to develop a conceptual framework based on our mechanistic understanding to expand the general utility of the Pd enolate. As such, we employed a strategy of divergent catalysis (Figure 1D), where deviation occurs at the common Pd enolate (i.e., **C**, Figure 1D, cf. Scheme 1, vide infra), allowing for desired alternative reactivity in the diverged cycle. Subsequent re-entry into the original catalytic cycle turns over the catalyst, allowing regeneration of the Pd enolate.

Applying this strategy of divergent catalysis, we developed a catalytic decarboxylative asymmetric intramolecular [4+2] cycloaddition from conjugated Pd enolates. Mechanistic studies including quantum mechanics calculations, Eyring analysis, and KIE studies offer insights into the reaction mechanism. This transformation enables access to tricyclic scaffolds bearing at least four contiguous stereocenters, at least one of which is quaternary.

## 2 Results and Discussion

### 2.1 Reaction Design and Optimization

**Figure 2:**
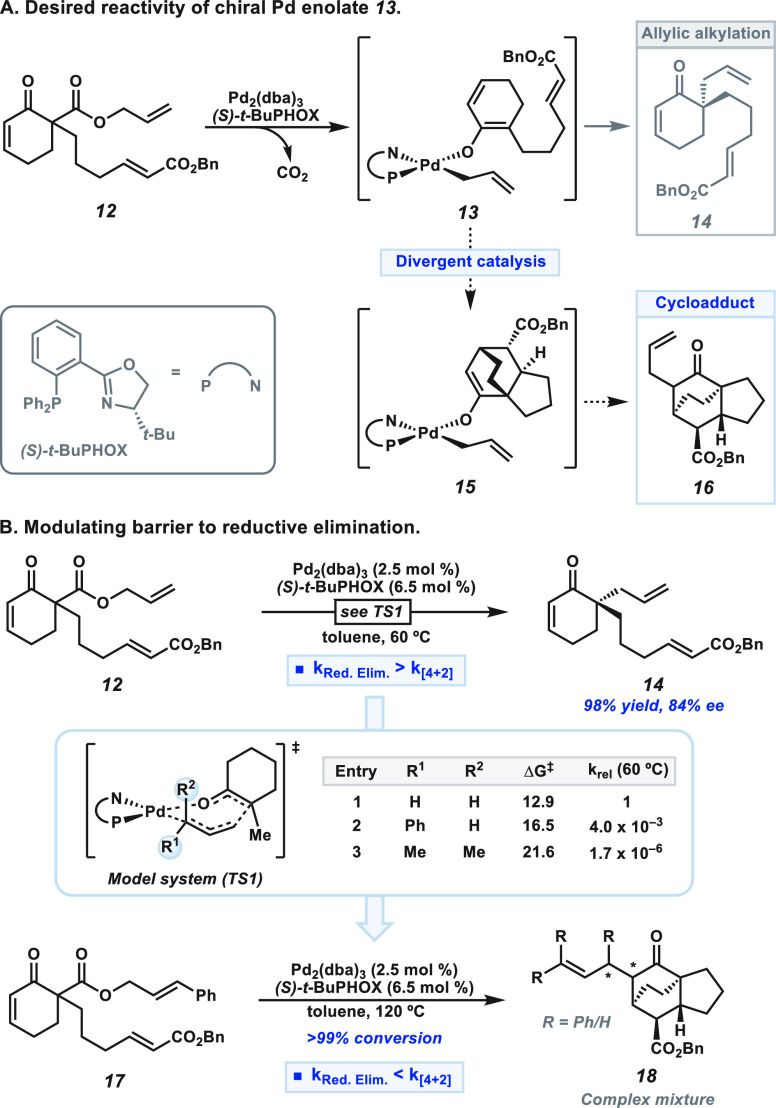
(A) General reactivity paradigm from Pd enolate **13**. (B) Computed substituent effects on the rate of C–C bond formation and successful application. See SI for computational details and discussion of other isomeric transition states. Yield determined by ^1^H NMR with respect to 1,3,5-trimethoxybenzene as internal standard. Enantiomeric excess determined by chiral SFC.

Employing unsaturated β-ketoester **12** as a precursor for conjugated Pd enolate **13**, we hypothesized that the precedented allylic alkylation forming **14** could be interrupted by a [4+2] cycloaddition to generate **15** (Figure 2A). Alkylation of the transposed enolate (**15**) would then turn over the catalyst and forge tricyclic product **16**.

Unfortunately, direct allylic alkylation of enolate **13** kinetically outcompetes the desired divergent reactivity. Treatment of β-ketoester **12** under our standard conditions produces ketone **14** in 98% yield and 84% ee (Figure 2B). This prompted us to redesign our exit strategy (Figure 1D). Increasing the rate of the cycloaddition through modification of the diene or dienophile could circumvent the formation of premature allylic alkylation product **14** but would limit the generality of this transformation. Therefore, we sought to impede alkylation through modification of the allyl moiety.

Computational investigation of a model system (**TS1**) suggested that introducing terminal substitution on the allyl group raises the barrier to reductive elimination, decreasing the rate of allylic alkylation (Figure 2B, see SI for computational details).^11^ For example, phenyl substitution (entry 2, Figure 2B) slows the rate of inner-sphere reductive elimination by roughly three orders of magnitude. Inspired by these computational results, we explored the efficacy of the cinnamyl ester substrate **17** in the transformation. In line with our hypothesis, the desired tricyclic core was observed (**18**), albeit as a complex mixture of isomers—hampering the synthetic utility. To this end, we sought to develop an alternative strategy for catalyst turnover that could potentially simplify the product outcomes.

**Figure 3:**
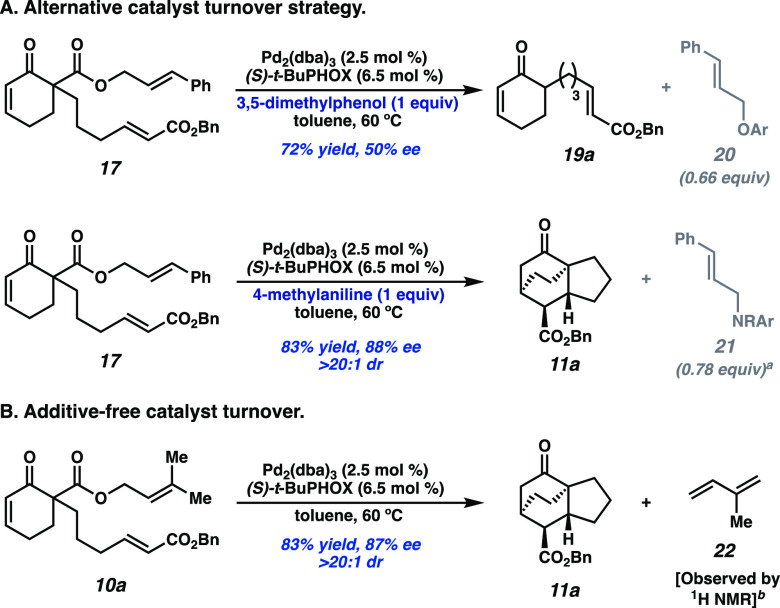
(A) Sacrificial additives to enable catalyst turnover. ^a^Equivalents include a mixture of branched and linear constitutional isomers, as well as double alkylation of aniline. (B) Additive-free reaction with prenyl ester **10a**. ^b^Isoprene observed in 0.94:1 ratio with **11a** by ^1^H NMR (J Young tube, toluene-*d*_8_).

Building upon previous findings from our group, we sought to employ stoichiometric acidic additives for catalyst turnover. The exogenous acid serves the dual purpose of protonating the final enolate (analogous to **15**) and turning over the catalyst by trapping the cinnamyl group. Addition of 3,5-dimethylphenol^9^ exclusively yielded undesired protonation product **19a** along with aryl ether **20** (Figure 3A). To our delight, replacing the phenol additive with 4-methylaniline afforded the desired *endo* [4+2] cycloadduct (**11a**) as a single diastereomer in 83% yield and 88% ee.

Seeking to improve the reaction yield, the competency of β-ketoester **10a**, derived from the commodity chemical prenyl alcohol, was explored. According to our computations, a substrate containing a di-substituted allyl fragment would be similarly effective in hindering premature allylic alkylation by increasing the barrier to reductive elimination (Figure 2B, entry 3). Perplexingly, while the desired tricyclic product was generated in 73% isolated yield and 88% ee, no alkylated 4-methylaniline (analogous to **21**) was observed as a byproduct (entry 8, Table 1).

A control reaction excluding 4-methylaniline was carried out, and surprisingly, desired product **11a** was formed in 83% yield and 87% ee (Figure 3B). This suggests that an alternative catalyst turnover mechanism is operative. Further NMR experiments revealed the stoichiometric evolution of isoprene (**22**) accompanying the formation of product **11a**. Intrigued by this unexpected finding and clean reaction profile, we pursued the optimization of additive-free reaction conditions.

The reaction proceeds in THF and benzene albeit in slightly diminished yield and enantioselectivity (entries 2–3, Table 1). Employing 1,4-dioxane as the solvent, protonation product **19a** was obtained as the major product in 63% yield and 49% ee, while cycloadduct **11a** was observed in only 12% yield (entry 4, Table 1). Lowering the temperature to 40 °C slightly improved the ee to 89% at the cost of decreased conversion (entry 5, Table 1). Modification of the electronic properties of the PHOX ligand deleteriously impacted the product distribution (entries 6–7, Table 1). Phenol and aniline additives do not improve the reaction (entries 8–10, Table 1). Ultimately, optimized reaction conditions were determined to be additive-free with *(S)*-*t*-BuPHOX in toluene at 60 °C. The reaction affords **11a**, a bridged bicycle with a pendant fused ring, in 83% isolated yield and 87% ee. The transformation allows for the simultaneous construction of four contiguous stereocenters, including one all-carbon quaternary center. Gratifyingly, the reaction can be performed with reduced catalyst loading (0.625 mol %) on a 1.0 mmol scale to afford **11a** in 59% yield and 89% ee. The ability to efficiently construct these complex building blocks on scale highlights the synthetic utility of this transformation.

**Table 1:**
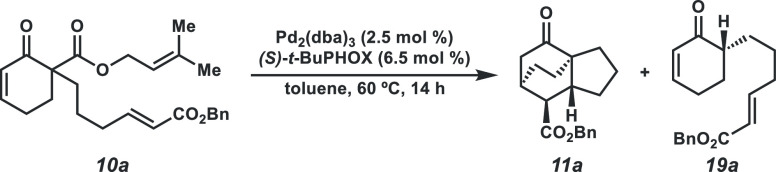

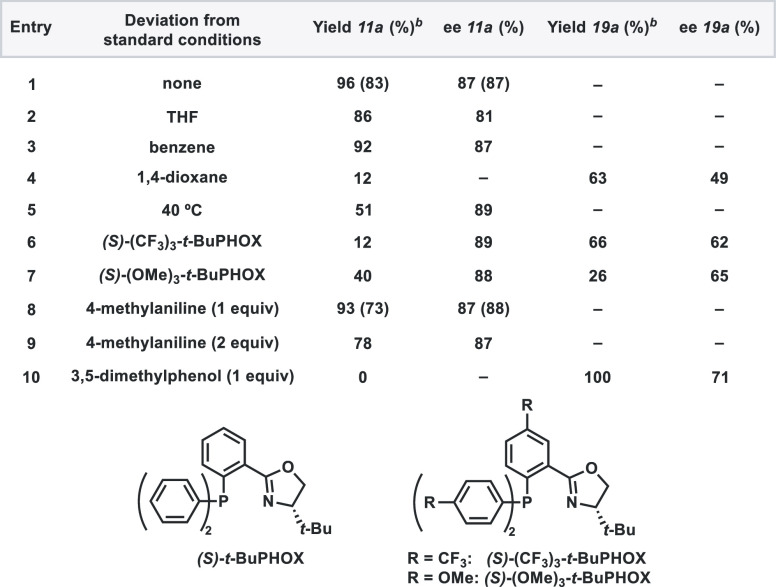
Optimization of [4+2] Reaction Conditions^a^,^b^

a Conditions: 0.02 mmol **10a**, 2.5 mol % Pd_2_(dba)_3_, 6.5 mol % ligand, in 1.0 mL of solvent (0.02 M).

b Yields determined by ^1^H NMR with respect to 1,3,5-trimethoxybenzene as internal standard. Isolated yields on a 0.2 mmol scale in parentheses.

### 2.2 Proposed Mechanism

We sought to capitalize on these initial exciting results by constructing a mechanistic framework to inform rational design. Based on our lab’s prior investigations of Pd-catalyzed decarboxylative asymmetric allylic alkylation reactions, we propose that oxidative addition of Pd^0^ to β-ketoester **10a** proceeds through complex **23** to afford the η^1^-allyl carboxylate resting state **24** (Scheme 1).^12^ Rate-limiting decarboxylation ensues, affording O-bound Pd enolate **25**.^3,12^ This chiral conjugated enolate then serves as the diene in a [4+2] cycloaddition with the pendant dienophile to form tricyclic enolate **26**. Subsequent proton transfer would generate product **11a**. Concomitant isoprene generation, followed by ligand exchange, allows for re-entry into the original catalytic cycle at **23**. We posit that the formation of undesired ketone **19a** arises from an off-cycle pathway, where catalyst turnover occurs prior to cycloaddition via premature proton transfer to **25**.

### 2.3 Substrate Scope

With a working mechanistic hypothesis in hand, we sought to draw further mechanistic insights from substrate design while simultaneously exploring the limits of the reaction.

Considering the inverse relationship between diene ring size and Diels–Alder reaction rate,^13^ we explored whether this trend impacts the generality of our transformation. However, with cyclopentyl diene derived from enone **10c**, a decrease in yield and ee, relative to a six-membered parent substrate **10a**, was noted (Table 2). In comparison to smaller ring sizes, seven-membered cyclic dienes require increased distortion energy to reach the desired transition state.^13^ Despite this, a seven-membered ring substrate **10d** leads to a high yield and improved ee. Thus, this transformation represents a powerful method to synthesize various challenging bicyclic cores.

**Table 2:**
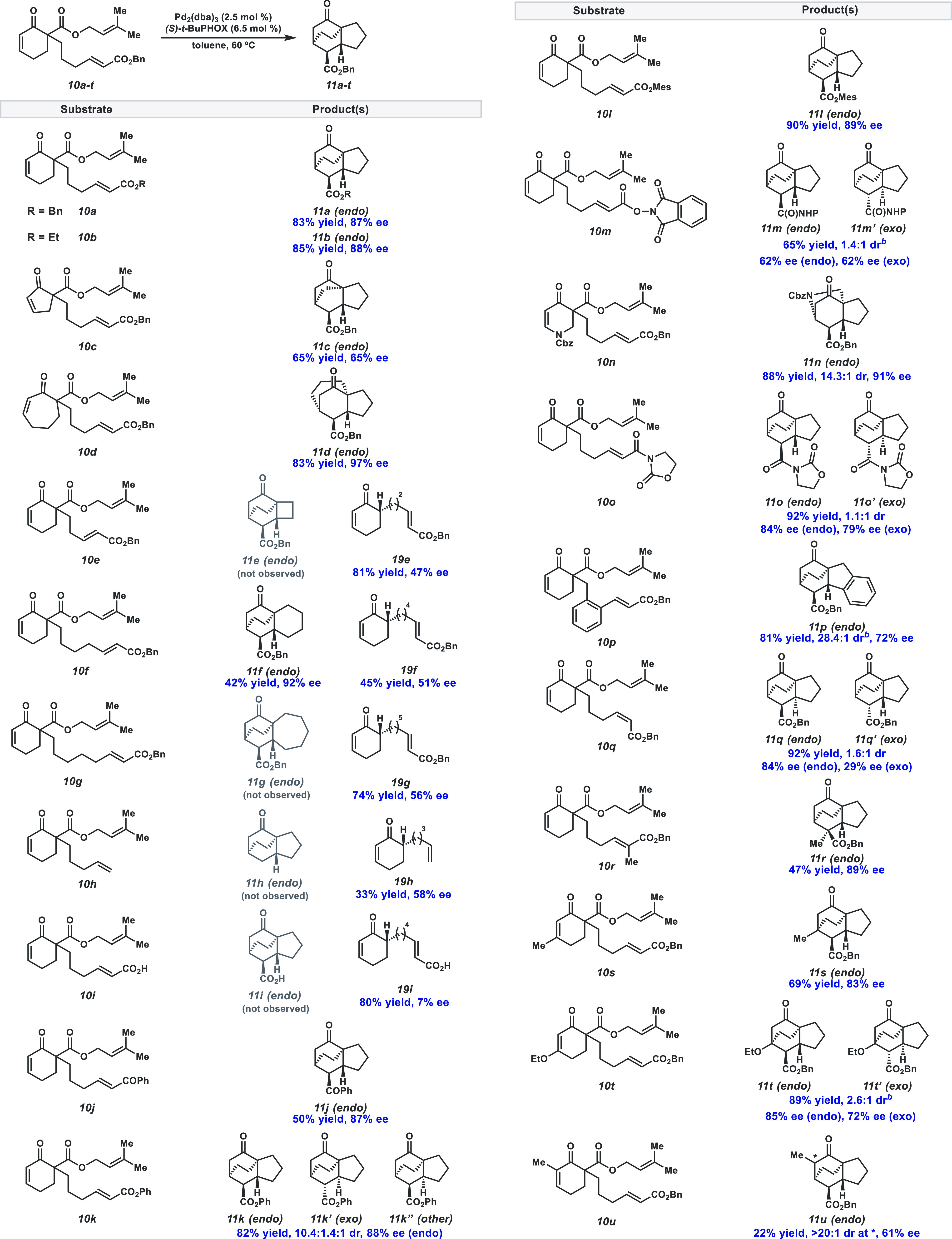
Substrate Scope of the [4+2] Reaction^a^,^b^

a Conditions: 0.2 mmol **10a**, 2.5 mol % Pd_2_(dba)_3_, 6.5 mol % ligand, in toluene (10 mL, 0.02 M), isolated yields, dr determined by ^1^H NMR analysis of reaction crude.

b dr determined by isolated yields of *endo*/*exo* products.

The dienophile tether length was subsequently modulated to test its influence on product distribution. The ethylene tethered substrate **10e** yields solely the premature protonation product **19e**, likely due to insurmountable developing ring strain in the cycloaddition transition state. In contrast to the propylene tethered substrate **10a**, the butylene-tethered substrate **10f** leads to a near equal distribution of cycloadduct **11f** (42% yield) and prematurely protonation product **19f** (45% yield). Following this trend, the pentylene-tethered substrate **10g** leads only to protonation product **19g**. Rationalizing this phenomenon, we propose that lengthening the tether increases conformational flexibility and imposes a greater entropic penalty to the highly organized [4+2] transition state. In contrast, increased tether length is inconsequential to the protonation process, which does not involve the dienophile.

**Figure 4:**
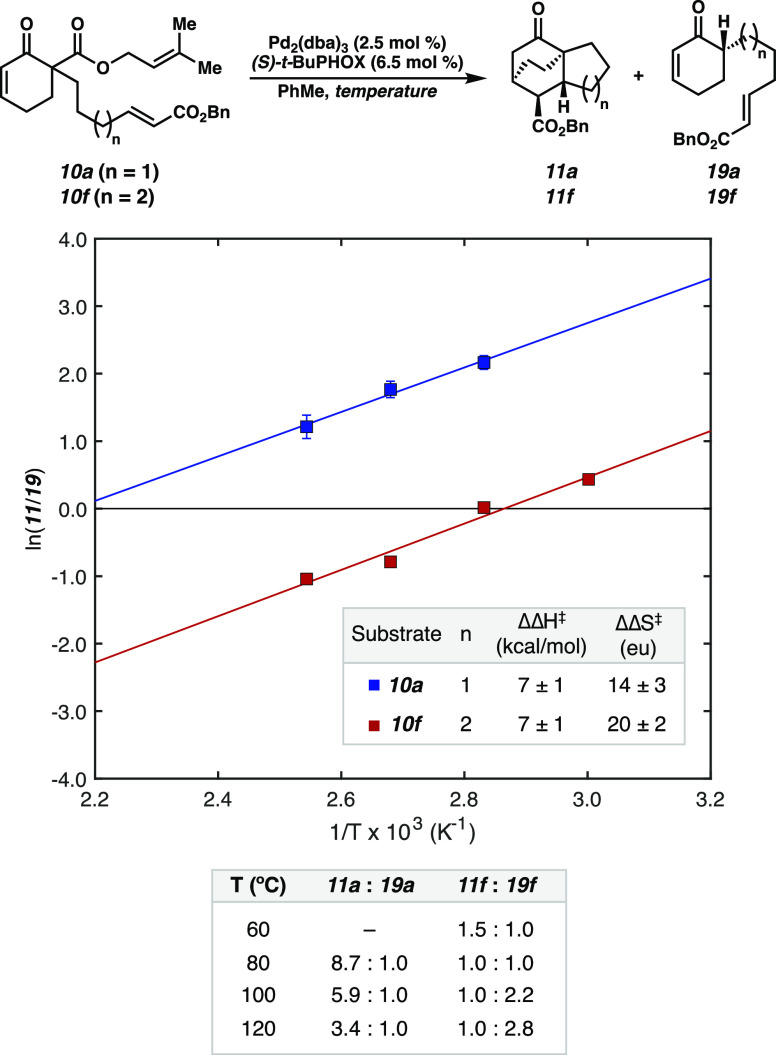
Eyring analysis of **11**/**19** product ratio for propylene and butylene-tethered substrates **10a** and **10f**. All data points collected in triplicate, and error bars and ranges reflect a 95% confidence interval.^14^ Reactions carried out on 0.02 mmol scale with product ratios determined by crude ^1^H NMR analysis.

Eyring analysis of product distributions from reactions of **10a** and **10f** further supports the hypothesis of an entropic preference for the formation of **19a/f** over **11a/f** (Figure 4). With **10a**, cycloaddition (**11a**) is enthalpically favored (ΔΔ*H*^‡^ = 7 kcal/mol) but entropically disfavored (ΔΔ*S*^‡^ = 14 eu) over protonation (**19a**). As anticipated, increasing the tether length to four methylene units (**10f**) further increases the relative entropic penalty for cycloaddition (ΔΔ*S*^‡^ = 20 eu), while the differential enthalpy of activation remains similar (ΔΔ*H*^‡^ = 7 kcal/mol). Hence, entropy differences associated with tether length lead to the formation of differential amounts of undesired ketones **19a** and **19f**.

We then surveyed the scope of functional groups that are tolerated in this reaction (Table 2). The cycloaddition does not proceed in the absence of a π-acceptor (**10h**), and carboxylic acid **10i** exclusively affords undesired ketone **19i**. To our delight, a variety of functional groups are compatible, including ethyl ester **10b**, phenyl ketone **10j**, phenyl ester **10k**, mesityl ester **10l**, N-hydroxyphthalimido (NHP) ester **10m**, enecarbamate **10n**, and *N*-acyl oxazolidinone **10o**. Additionally, further conjugated cinnamic ester dienophile **10p** affords tetracycle **11p**. These results demonstrate the ability to tolerate varying dienophile electronics, incorporate additional functional handles, and access alternate ring systems.

The majority of the substrate scope is reflective of a stereospecific process, yielding only *endo* and *exo* diastereomers. We sought to exploit this property of the reaction to access other diastereomers of **11a** by employing *(Z)*-olefin dienophile **10q**. Gratifyingly, desired cycloadducts **11q** (*endo*) and **11q′** (*exo*) are furnished in a 1.6:1 ratio with a 92% combined yield, in 84 and 29% ee, respectively.

Further substitution patterns on the substrate were explored with the aim of increasing the stereochemical complexity of the products. Trisubstituted benzyl ester dienophile **10r** furnished cycloadduct **11r**, featuring two all-carbon quaternary centers, in 47% yield and 90% ee. β-Methyl (**10s**) and β-ethoxy (**10t**) α,β-unsaturated enones are also competent substrates, forging additional tetrasubstituted bridgehead stereocenters. Finally, we explored α-methyl substituted enone **10u**. The corresponding product **11u** was produced, bearing five contiguous stereocenters in >20:1 dr.

In summary, the transformation described herein represents a versatile method for the preparation of a variety of enantioenriched polycyclic scaffolds. Inspired by these results, we sought to explore the origins of enantioinduction and the mechanism by which catalyst turnover is achieved.

### 2.4 C–C Bond Formation

**Figure 5:**
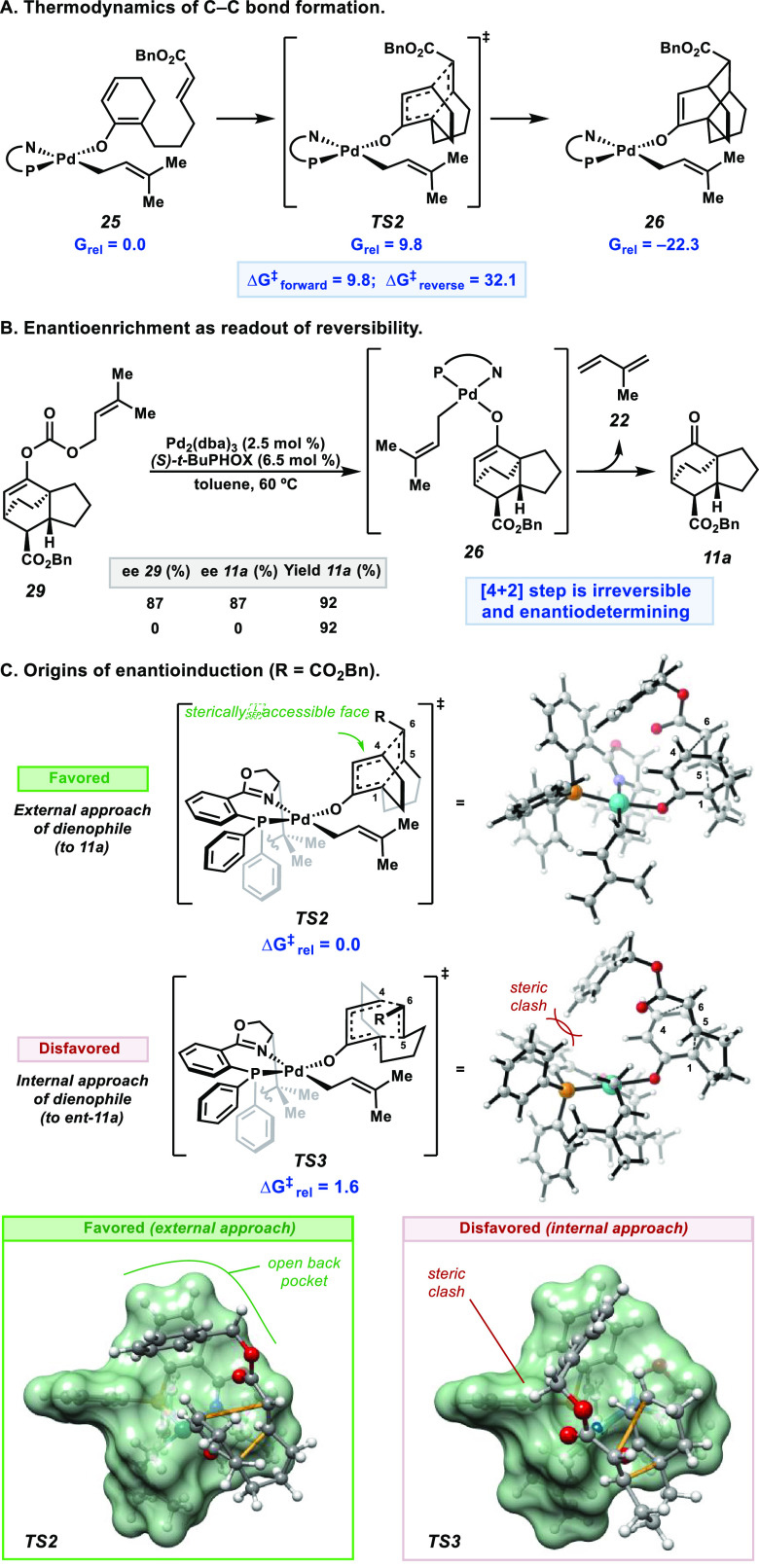
(A) Computed barriers to irreversible C–C bond formation. (B) Experimentally verifying irreversibility of the C–C bond formation. (C) Origins of enantioinduction in the [4+2] cycloaddition step.

In order to probe the origins of enantioinduction in the transformation, we first aimed to elucidate the enantiodetermining step in the catalytic cycle. We hypothesized that either the cycloaddition is irreversible and dictates the stereochemical outcome, or a reversible [4+2] is coupled to a subsequent enantiodetermining step. First, we computationally evaluated the energetics of the [4+2] process. Cycloaddition directly from conjugated enolate **25** to transposed enolate **26** via **TS2** is achieved with a Δ*G*^‡^ of 9.8 and Δ*G* of −22.3 kcal/mol (Figure 5A). The 32.1 kcal/mol barrier to the reverse process renders the cycloaddition step irreversible under the reaction conditions. Hence, our computations suggest that the cycloaddition step is enantiodetermining.

To assess this hypothesis experimentally, reaction product **11a** was converted to its corresponding prenyl enol carbonate **29**. Under the standard reaction conditions, Pd^0^ undergoes oxidative addition to **29**, and decarboxylation affords target common intermediate **26** (Figure 5B).^5^ When enantioenriched or racemic **29** is subjected to the reaction conditions, cycloadduct **11a** is obtained in high yield and identical enantiopurity to that of the respective enol carbonate precursor (**29**) (Figure 5B). No stereochemical resolution in product **11a** is observed from racemic enol carbonate **29**, indicating that a post-cycloaddition process is not responsible for enantioinduction. In addition to verifying the irreversibility of the cycloaddition step, these experiments also support the viability of enolate **26** as an intermediate in the catalytic cycle (Scheme 1).

Considering the [4+2] cycloaddition as the enantiodetermining process, the origin of enantioinduction in this step was investigated. As such, the lowest-energy *endo* transition states giving rise to each enantiomer of **11a** were evaluated (Figure 5C). The minimum energy pathway to each enantiomer of the product features a transition state in which the dienophile tether is *syn* to the *t*-Bu group of the PHOX ligand—in accord with prior observations in inner-sphere allylic alkylation transition states.^3,15^ From this orientation, the dienophile preferentially approaches the externally exposed enantiotopic face of the diene to avoid a steric clash between the benzyl ester and the phenyl groups of the PHOX ligand scaffold (Figure 5C). A 1.6 kcal/mol preference for external (**TS2**) over internal (**TS3**) approach is calculated, in accord with the experimentally observed 87% ee.^16^ The major enantiomer of the product (**11a**) predicted by computations matches that of the major enantiomer obtained experimentally, as confirmed by vibrational circular dichroism (VCD) spectroscopy (see SI for details).

In summary, our investigations reveal C–C bond formation to be the enantiodetermining step, with enantioselectivity achieved by biasing *external* over *internal* dienophile approach (Figure 5).

### 2.5 Catalyst Turnover

Our [4+2] transformation is rendered catalytic by a unique reduction of Pd^II^ to Pd^0^ that occurs concomitantly with the formation of isoprene (**22**) and ketone **11a**. This observation motivated computational investigations to elucidate the catalyst turnover mechanism.

**Figure 6:**
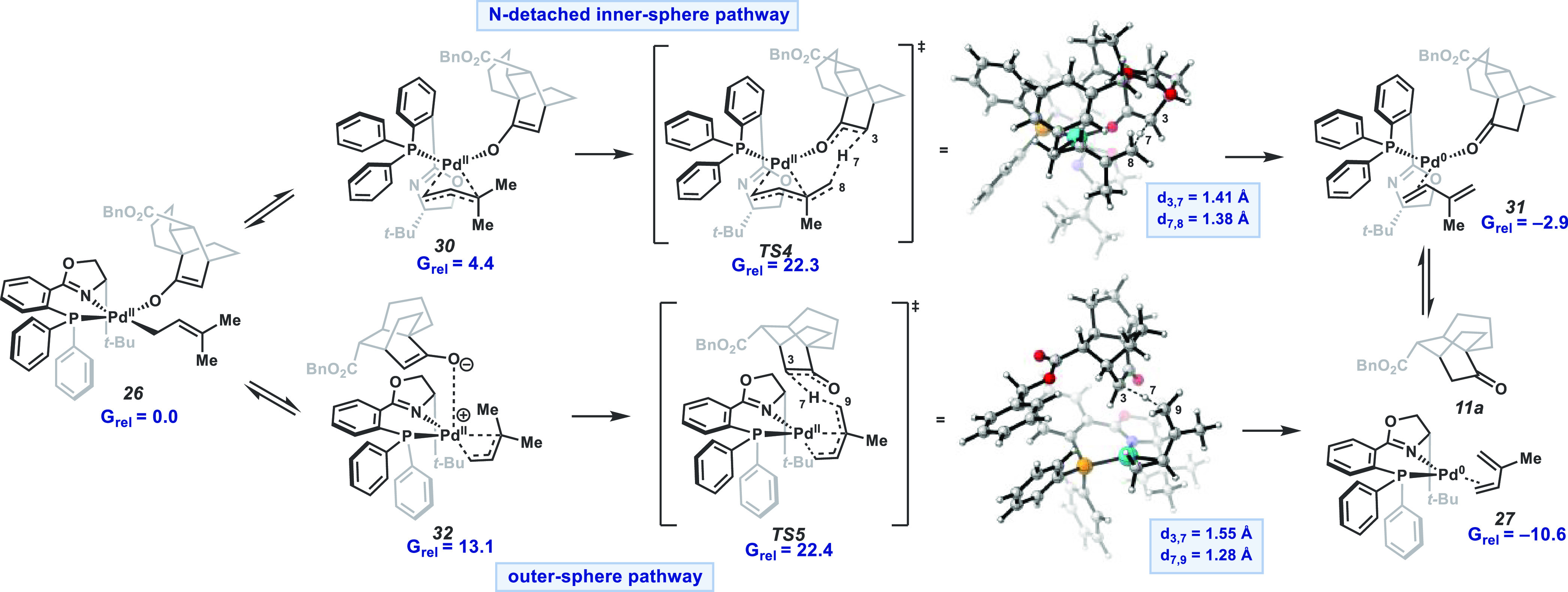
Relative free energies (in kcal/mol) of the two lowest-energy pathways for catalyst turnover.

Of the numerous mechanisms explored, the minimum energy pathway involves the isomerization of **26** to an N-detached π-allyl Pd species (**30**) and subsequent inner-sphere proton transfer (**TS4**) (Figure 6). Additionally, a pathway featuring outer-sphere proton transfer (**TS5**) was found to be highly competitive for catalyst turnover. These two processes present very similar free energy barriers of 22.3 and 22.4 kcal/mol, respectively, which are readily surmountable at 60 °C. A single favored pathway is not identified as the energy difference between the two mechanisms is within error of computations. In both pathways, subsequent ligand exchange of isoprene (**22**) for starting material **10a** completes the catalytic cycle. Analysis of Intrinsic Bonding Orbitals (IBOs)^17^ along the reaction coordinate suggests these processes are best conceptualized as the transfer of a proton, rather than a hydride, to the Pd enolate (see SI for details).^18^ Analogous mechanisms were found to be operative from pre-cycloaddition enolate **25**, giving rise to premature protonation product **19a**.

### 2.6 Further Mechanism-Based Developments

While this method allows access to a variety of complex scaffolds, premature protonation remains an outstanding challenge we sought to address. As such, we aimed to leverage our mechanistic insights surrounding this process to inhibit byproduct formation.

**Figure 7:**
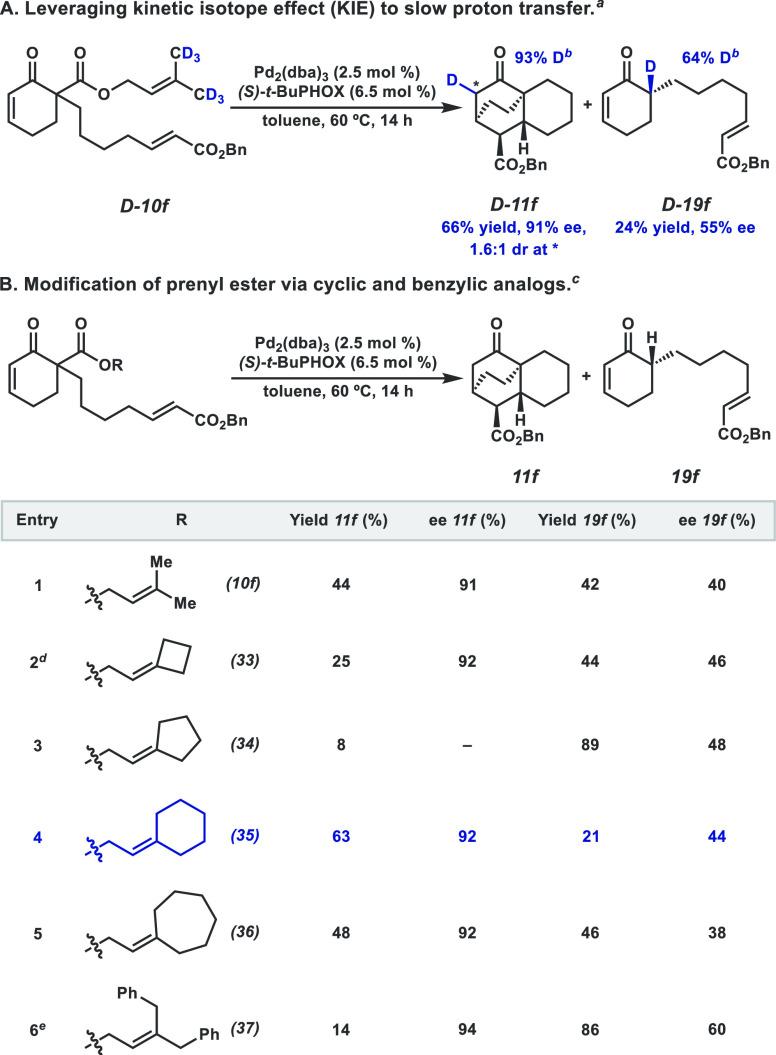
(A) KIE study. (B) Prenyl ester modification. ^a^Conditions: 0.20 mmol **D-10f**, 2.5 mol % Pd_2_(dba)_3_, 6.5 mol % ligand, in 10 mL of solvent (0.02 M). ^b^Deuterium incorporation determined by HRMS. ^c^Conditions: 0.02 mmol substrate, 2.5 mol % Pd_2_(dba)_3_, 6.5 mol % ligand, in 1.0 mL of solvent (0.02 M). Yield determined by ^1^H NMR with respect to 1,3,5-trimethoxybenzene as internal standard. ^d^21% of allylic alkylation product was also observed (see SI for details). ^e^The corresponding benzylic diene was also observed (see SI for details).

To that end, we turned our attention to the butylene-tethered substrate **10f** given its similar yield of desired **11f** (44%) and byproduct **19f** (42%). We envisioned favoring the formation of **11f** by modification of the ancillary prenyl moiety. By introducing a kinetic isotope effect, we aimed to slow down the protonation processes. To our delight, employing hexa-deutero prenyl ester **D-10f** (Figure 7A) increases the yield of desired cycloadduct **D-11f** to 66%, with 91% ee.^19^ Next, cyclic analogs of the prenyl ester **10f** were prepared (Figure 7B). At one extreme, seven-membered exocycle **36** affords a product distribution which closely mirrors that of the parent substrate **10f** (entry 5). Excitingly, contracting the ring by one methylene (**35**) shifts the distribution favorably toward **11f** (entry 4, 3:1 ratio of **11f**:**19f**). However, five- and four-membered exocycles (**34** and **33**), as well as acyclic bis-benzylic allylic ester **37**, afford unfavorable product distributions.

In summary, we find appropriate modification of the prenyl moiety to be effective in suppressing deleterious side reactions. This is particularly important as the ring system generated in this reaction is a scaffold relevant to natural product synthesis.

### 2.7 Product Derivatization

**Figure 8:**
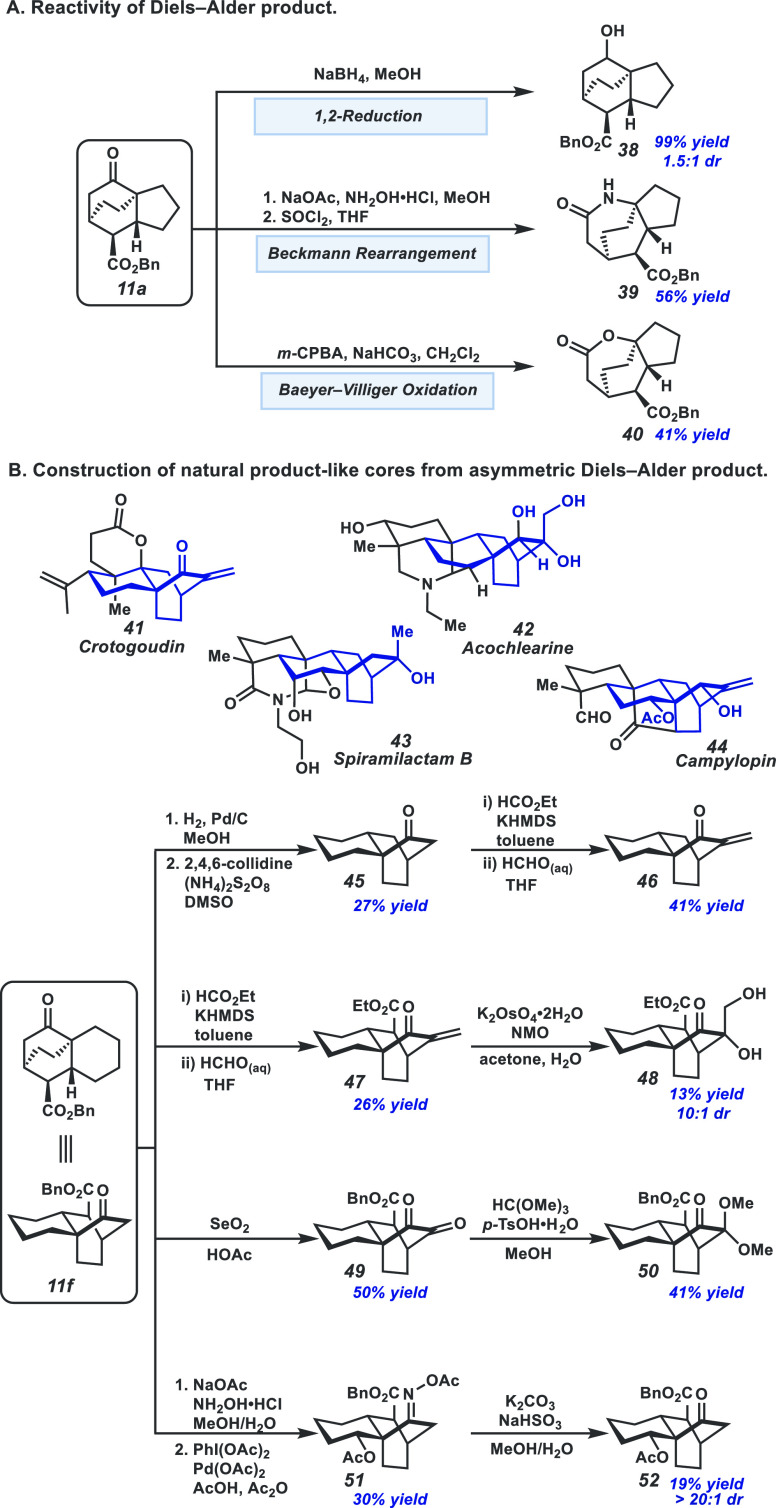
(A) Oxidation state alterations, ring system adjustments, and heteroatom incorporation on **11a**. (B) Reaction sequences to construct natural product-like cores.

To assess the utility of the asymmetric intramolecular [4+2] products, we started by altering the oxidation state of ketone **11a** (Figure 8A) through a 1,2-reduction, which provided alcohol **38** in quantitative yield and in 1.5:1 dr. Subsequently, we explored ring expansion strategies to incorporate heteroatoms and to furnish different ring systems (Figure 8A). From ketone **11a**, oxime condensation and subsequent Beckmann rearrangement afforded lactam **39** as a single isomer in 56% yield over two steps. Analogously, Baeyer–Villiger oxidation furnished lactone **40** in 41% yield as a single isomer.

Furthermore, the tricyclic cycloaddition products closely resemble many members of the atisane family of diterpenoids (Figure 8B, **41**–**44**). Therefore, reactions to further functionalize these scaffolds were explored. First, hydrogenolysis followed by persulfate-mediated radical decarboxylation of **11f** afforded ketone **45** in 27% yield over two steps.^20^ We were delighted to find that the exocyclic methylene motif presented in both crotogoudin (**41**) and campylopin (**44**) could be achieved through aldol condensations from both **45** and **11f** to yield crotogoudin-like enone **46** in 41% yield and analogous enone **47** in 26% yield.^21^ Enone **47** can be further functionalized through dihydroxylation to furnish the primary and tertiary alcohols of the acochlearine (**42**) core in 13% yield and 10:1 dr (**48**).^22^ A wider spectrum of natural product cores could also be accessed through oxidation at different sites of the tricyclic hydrocarbon backbone. For example, Riley oxidation of **11f** provided diketone **49** in 50% yield,^23^ which can then be selectively monoprotected as acetal **50** in 41% yield.^24^ Further manipulations to the exposed ketone of **50** could yield spiramilactam B (**43**)-like oxidation patterns. To that end, directed C–H oxidation following an oxime condensation of **11f** yielded oxime **51** in 30% yield. Deprotection of the oxime afforded the desired acetate on campylopin (**44**)-like tricycle **52** in 19% yield as a single diastereomer.^25^

Overall, derivatization of the Diels–Alder product **11f** allowed access to four natural product-like motifs, demonstrating the potential of applying this transformation to asymmetric natural product syntheses.

## 3 Conclusions

We developed an asymmetric decarboxylative [4+2] cycloaddition employing a key catalytically generated chiral Pd enolate intermediate—analogous to those implicated in inner-sphere allylic alkylation reactions. To enable this transformation, we first systematically modified the allyl moiety to disfavor undesired allylic alkylation. This allows the conjugated Pd enolate to engage in a [4+2] cycloaddition with a pendant dienophile. Computational and experimental analyses support the role of C–C bond formation as the enantiodetermining step. Further computational investigation reveals that the catalyst turnover occurs through a proton transfer from the prenyl group directly to the transposed enolate, forming the desired product and releasing isoprene. Building upon these mechanistic insights, we were able to further favor the desired [4+2] cycloaddition over premature protonation for challenging substrates relevant to complex natural product synthesis.

In summary, our approach to divergent catalysis serves as a powerful framework for rational design in asymmetric catalytic reactions. Studies applying this strategy more broadly in other synthetically relevant transformations are currently underway.

## Supporting Information Available

Complete experimental and computational details (PDF)
Cartesian coordinates of the calculated structures (ZIP)
QM energies and analysis (XLSX)
