# Supplementary material for: Divergent Catalysis: Catalytic Asymmetric [4+2] Cycloaddition of Palladium Enolates
Source: J Am Chem Soc. 2023 May 15;145(20):11301–10. doi: 10.1021/jacs.3c02104 (PMC10388310; doi:10.1021/jacs.3c02104)
Supplement: Supplementary file 1 — ja3c02104_si_001.pdf [file ja3c02104_si_001.pdf]

Supporting Information for

Divergent Catalysis: Catalytic Asymmetric [4+2] Cycloaddition of Palladium Enolates

Kaylin N. Flesch[‡], Alexander Q. Cusumano[‡], Peng-Jui Chen, Christian Santiago Strong, Stephen R. Sardini, Yun E. Du, Michael D. Bartberger, William A. Goddard III*, Brian M. Stoltz*

Warren and Katharine Schlinger Laboratory of Chemistry and Chemical Engineering, Division of Chemistry and Chemical Engineering, California Institute of Technology, MC 101-20, Pasadena, California 91125, United States

stoltz@caltech.edu

wag@caltech.edu

Table of Contents:

Materials and Methods 2

List of Abbreviations: 3

Pd-Catalyzed Decarboxylative Cycloadditions 3

Preparation of Unsaturated β-Ketoester Starting Materials 42

Preparation of Aldehyde Precursors 68

β-Ketoesters Synthesis 81

Protonated Enone Byproducts 92

Product Derivatizations 108

Preparation of Additional Compounds 120

Determination of Absolute and Relative Stereochemistry by VCD 121

2D NMR Analysis of Select Compounds 146

Computational details 152

References 159

NMR and IR Spectra of New Compounds 163

Materials and Methods

Unless otherwise stated, reactions were performed in flame-dried glassware under an argon or nitrogen atmosphere using dry, deoxygenated solvents. Solvents were dried by passage through an activated alumina column under argon.[1] Reaction progress was monitored by thin-layer chromatography (TLC) or Agilent 1290 UHPLC-MS. TLC was performed using E. Merck silica gel 60 F254 precoated glass plates (0.25 mm) and visualized by UV fluorescence quenching or $KMnO_4$ staining. Silicycle Silia*Flash*® P60 Academic Silica gel (particle size 40–63 nm) was used for flash chromatography. ^{1}H NMR spectra were recorded on a Bruker 400 MHz spectrometer and are reported relative to residual $CHCl_3$ (δ 7.26 ppm). ^{13}C NMR spectra were recorded on a Bruker 400 MHz spectrometer (100 MHz) and are reported relative to $CHCl_3$ (δ 77.16 ppm). ^{2}H NMR spectra were recorded on a Bruker 400 MHz (61 MHz) spectrometer and are reported relative to residual $CDCl_3$ (δ 7.26 ppm). Data for ^{1}H NMR are reported as follows: chemical shift (δ ppm) (multiplicity, coupling constant (Hz), integration). Multiplicities are reported as the peaks appear as follows: s = singlet, d = doublet, t = triplet, q = quartet, p = pentet, sept = septuplet, m = multiplet, br s = broad singlet, br d = broad doublet. Data for ^{13}C NMR are reported in terms of chemical shifts (δ ppm). Some reported spectra include minor solvent impurities of water (δ 1.56ppm), ethyl acetate (δ 4.12, 2.05, 1.26 ppm), methylene chloride (δ 5.30 ppm), acetone (δ 2.17 ppm), grease (δ 1.26, 0.86 ppm), and/or silicon grease (δ 0.07 ppm), which do not impact product assignments. ^{13}C NMR spectra of deuterated compounds are complicated by the low intensity of peaks of deuterium-substituted carbon atoms. IR spectra were obtained by use of a Perkin Elmer Spectrum BXII spectrometer or Nicolet 6700 FTIR spectrometer using thin films deposited on NaCl plates and reported in frequency of absorption (cm^{-1}). Optical rotations were measured with a Jasco P-2000 polarimeter operating on the sodium D-line (589 nm), using a 100 mm path-length cell. Analytical SFC was performed with a Mettler SFC supercritical CO_2 analytical chromatography system utilizing Chiralpak (AD-H or IC) or Chiralcel (OD-H, OJ-H, or OB-H) columns (4.6 mm x 25 cm) obtained from Daicel Chemical Industries, Ltd. High resolution mass spectra (HRMS) were obtained from the Caltech Mass Spectral Facility using a JEOL JMS-600H High Resolution Mass Spectrometer in Field Desorption (FD+) mode. Absolute stereochemical assignments were made by vibrational circular dichroism analysis for select compounds with related compounds assigned by analogy.

Reagents were purchased from commercial sources and used as received unless otherwise stated. Ligands were prepared according to literature procedures.[2]

List of Abbreviations:

ee – enantiomeric excess, SFC – supercritical fluid chromatography, TLC – thin-layer chromatography, IPA – isopropanol, VCD – vibrational circular dichroism.

Pd-Catalyzed Decarboxylative Cycloadditions

General Procedure A: Asymmetric Pd-Catalyzed Decarboxylative Cycloadditions.

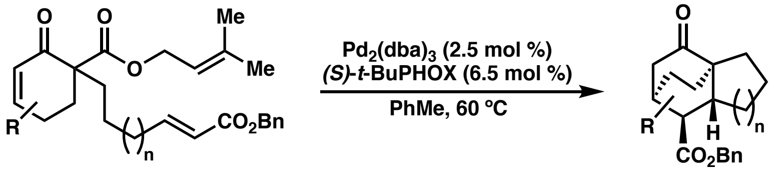

10 *11*

In a nitrogen filled glovebox, an oven-dried 20 mL vial was charged with a stir bar, $Pd_2(dba)_3$ (4.6 mg, 0.005 mmol, 2.5 mol %), *(S)*-*t*-BuPHOX (5.0 mg, 0.013 mmol, 6.5 mol %), and toluene (5 mL). The catalyst solution was stirred at 23 ºC for 20 min. A solution of substrate **10** (0.2 mmol, 1 equiv) in toluene (5 mL) was added to the vial. The resultant solution was then heated to 60 ºC for 14 h. The solution was then cooled to 23 ºC and concentrated under reduced pressure. The crude reaction mixture was loaded directly onto a flash column and the product (**11**) was isolated by silica gel flash column chromatography.

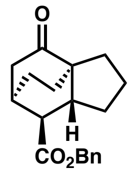

11a

benzyl (3a*R*,6*R*,7*S*,7a*R*)-4-oxooctahydro-3a,6-ethanoindene-7-carboxylate (11a)

Prepared from **10a** following General Procedure A. Purification by flash column chromatography (0–35% EtOAc/hexanes) afforded the title compound as a colorless oil (49.7 mg, 0.167 mmol, 83% yield, 87% ee). Absolute and relative stereochemistry were assigned by VCD (vida infra). 2D NMR studies independently confirm the relative stereochemistry (vida infra).

^{1}H NMR (400 MHz, CDCl$_3$): δ 7.40 – 7.31 (m, 5H), 5.14 (d, *J* = 1.6 Hz, 2H), 2.54 (dt, *J* = 18.8, 2.3 Hz, 1H), 2.51 – 2.47 (m, 2H), 2.21 (dddd, *J* = 10.6, 8.7, 7.2, 1.7 Hz, 1H), 2.14 – 2.04 (m, 3H), 1.86 (ddd, *J* = 13.0, 11.1, 6.8 Hz, 1H), 1.81 – 1.72 (m, 2H), 1.70 – 1.52 (m, 3H), 1.44 (ddt, *J* = 12.9, 10.9, 1.8 Hz, 1H), 1.22 (ddd, *J* = 13.9, 9.2, 4.9 Hz, 1H).

^{13}C NMR (100 MHz, CDCl$_3$): δ 215.1, 174.7, 136.0, 128.8, 128.5, 128.2, 66.7, 54.2, 47.5, 43.3, 41.3, 32.9, 29.1, 27.4, 26.5, 25.1, 22.6.

IR (Neat Film, NaCl): 2947, 2873, 1726, 1455, 1267, 1160 cm^{-1}.

HRMS (MM: FD+): *m/z* calc'd for $C_{19}H_{22}O_3$ $[M]^+$: 298.1564, found 298.1576.

Optical Rotation: $[\alpha]_D^{21}$ –20.3 (c 1.00, $CHCl_3$).

SFC conditions: 15% IPA, 2.5 mL/min, Chiralpak AD-H column, λ = 210 nm, t_R (min): minor = 4.21, major = 5.30

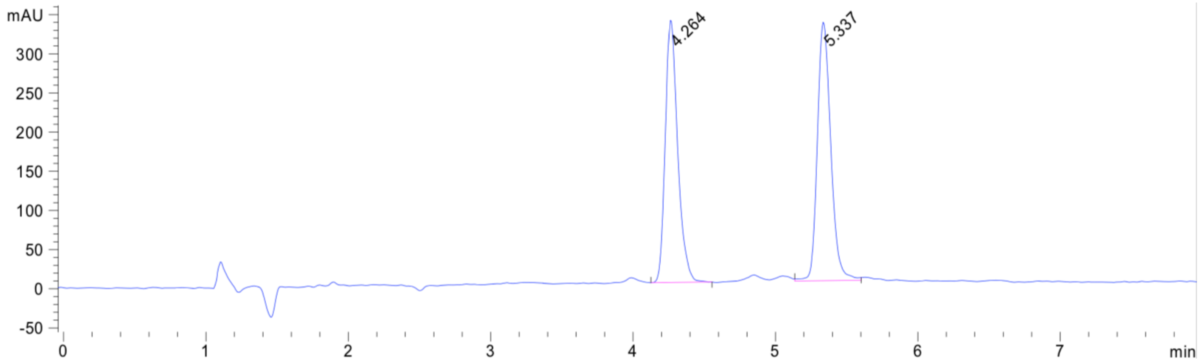

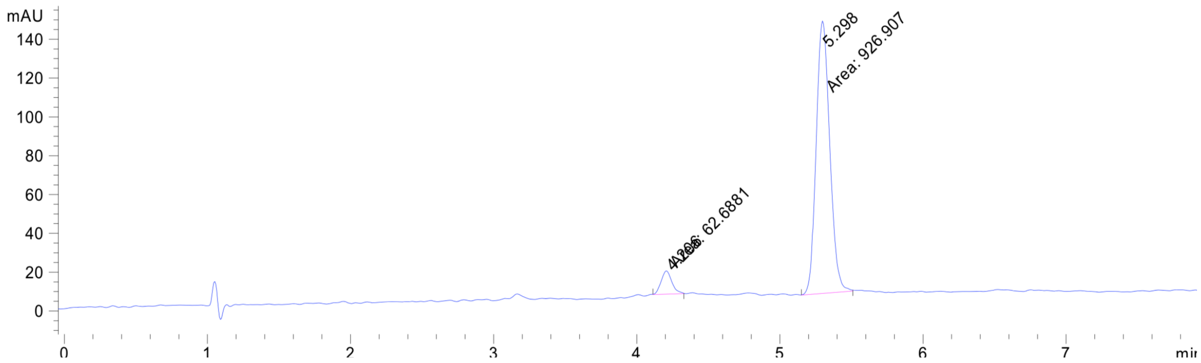

Peak #	RetTime [min]	Type	Width [min]	Area [mAU*s]	Height [mAU]	Area %
1	4.206	MM	0.0866	62.68811	12.05797	6.3347
2	5.298	MM	0.1099	926.90741	140.55699	93.6653
Totals :				989.59552	152.61496	

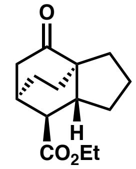

11b

ethyl (3a*R*,6*R*,7*S*,7a*R*)-4-oxooctahydro-3a,6-ethanoindene-7-carboxylate (11b)

Prepared from **10b** following General Procedure A. Purification by flash column chromatography (5–30% EtOAc/hexanes) afforded the title compound as a colorless oil (39.9 mg, 0.169 mmol, 84% yield, 88% ee).

^{1}H NMR (400 MHz, $CDCl_3$): δ 4.15 (q, *J* = 7.1 Hz, 2H), 2.55 (dt, *J* = 18.8, 2.6 Hz, 1H), 2.49 – 2.44 (m, 1H), 2.42 (d, *J* = 8.6 Hz, 1H), 2.23 – 2.14 (m, 1H), 2.15 – 2.04 (m, 3H), 1.91 – 1.71 (m, 3H), 1.71 – 1.52 (m, 3H), 1.50 – 1.40 (m, 1H), 1.26 (t, *J* = 7.1 Hz, 3H), 1.21 (dt, *J* = 9.2, 4.9 Hz, 1H).

^{13}C NMR (100 MHz, $CDCl_3$): δ 215.2, 174.9, 60.8, 54.2, 47.5, 43.3, 41.3, 32.9, 29.1, 27.4, 26.5, 25.1, 22.6, 14.4.

IR (Neat Film, NaCl): 2947, 2873, 1725, 1270, 1170 cm^{-1}.

HRMS (MM: FD+): *m/z* calc'd for $C_{14}H_{20}O_3$ $[M]^+$: 236.1412, found 236.1415.

Optical Rotation: $[\alpha]_D^{21}$ –34.2 (c 1.00, $CHCl_3$).

Enantiomeric excess determined by converting ethyl ester to benzyl ester through saponification and Steglish esterification.

SFC conditions: 15% IPA, 2.5 mL/min, Chiralpak AD-H column, λ = 210 nm, t_R (min): minor = 4.21, major = 5.30

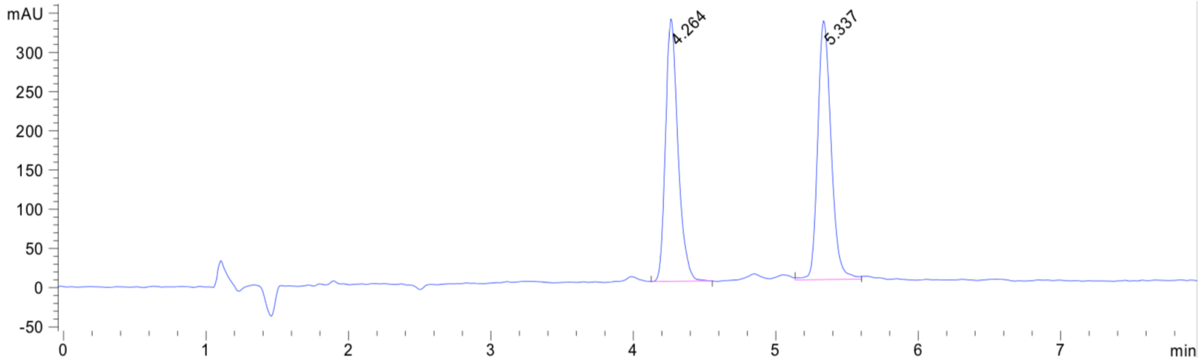

DAD1 A, Sig=210,16 Ref=370,60 (C:\CHEM32\1\DATA\AQC\AQC5-287_6.D)

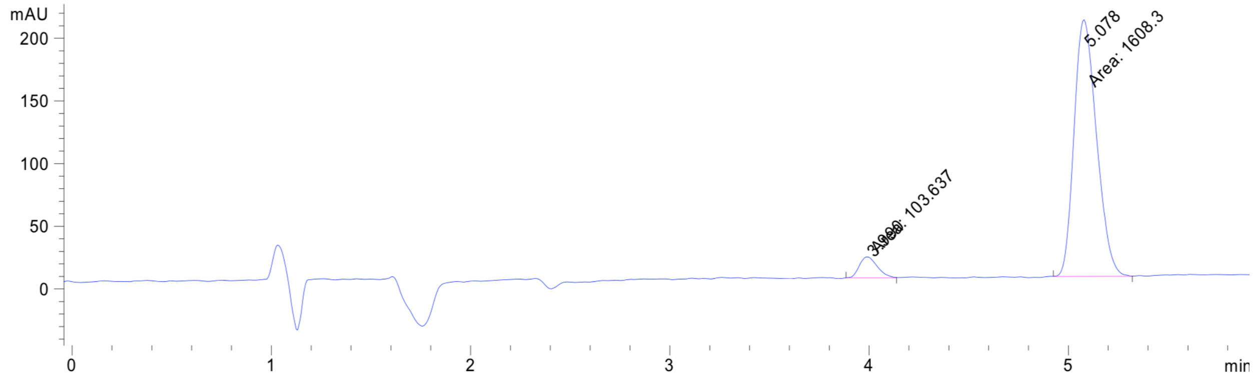

Peak #	RetTime [min]	Type	Width [min]	Area [mAU*s]	Height [mAU]	Area %
1	3.990	MM	0.1027	103.63741	16.82343	6.0538
2	5.078	MM	0.1305	1608.29517	205.39177	93.9462

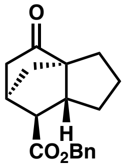

11c

benzyl (3a*R*,6*R*,7*S*,7a*R*)-4-oxooctahydro-3a,6-methanoindene-7-carboxylate (11c)

Prepared from **10c** following General Procedure A. Purification by flash column chromatography (0–30% EtOAc/hexanes) afforded the title compound as a colorless oil (37.0 mg, 0.130 mmol, 65% yield, 65% ee).

^{1}H NMR (400 MHz, CDCl$_3$): δ 7.42 – 7.29 (m, 5H), 5.13 (dd, *J* = 12.3, 7.9 Hz, 2H), 2.96 (t, *J* = 4.2, 1H), 2.92 (ddd, *J* = 5.2, 3.7, 1.4, 1H), 2.42 – 2.35 (m, 1H), 2.22 – 2.17 (m, 1H), 2.16 – 2.05

(m, 3H), 1.95 (ddtd, J = 12.9, 8.4, 5.1, 2.2 Hz, 1H), 1.90 – 1.80 (m, 2H), 1.66 (dt, J = 10.6, 1.6 Hz, 1H), 1.52 – 1.37 (m, 2H).

^{13}C NMR (100 MHz, CDCl$_3$): δ 213.9, 173.3, 136.0, 128.8, 128.5, 128.3, 67.8, 66.6, 52.4, 48.5, 41.8, 40.6, 40.5, 32.2, 27.5, 22.1.

IR (Neat Film, NaCl): 2960, 2358, 1739, 1164, 730, 668 cm^{-1}.

HRMS (MM: FD+): *m/z* calc'd for $C_{18}H_{20}O_3$ [M]$^+$: 284.1414, found 284.1407.

Optical Rotation: $[\alpha]_D^{21}$ +20.0 (c 1.00, CHCl$_3$).

SFC conditions: 15% IPA, 2.5 mL/min, Chiralpak AD-H column, λ = 210 nm, t_R (min): minor = 3.97, major = 4.33.

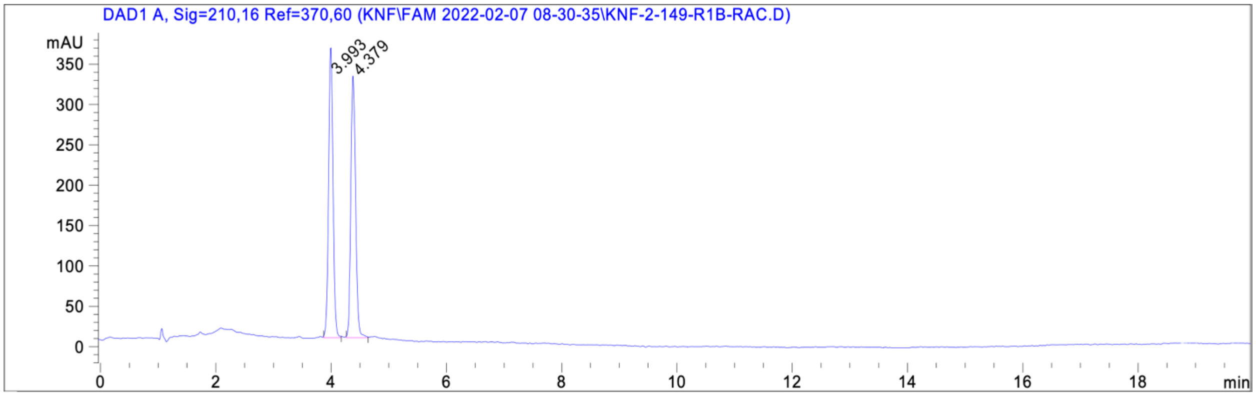

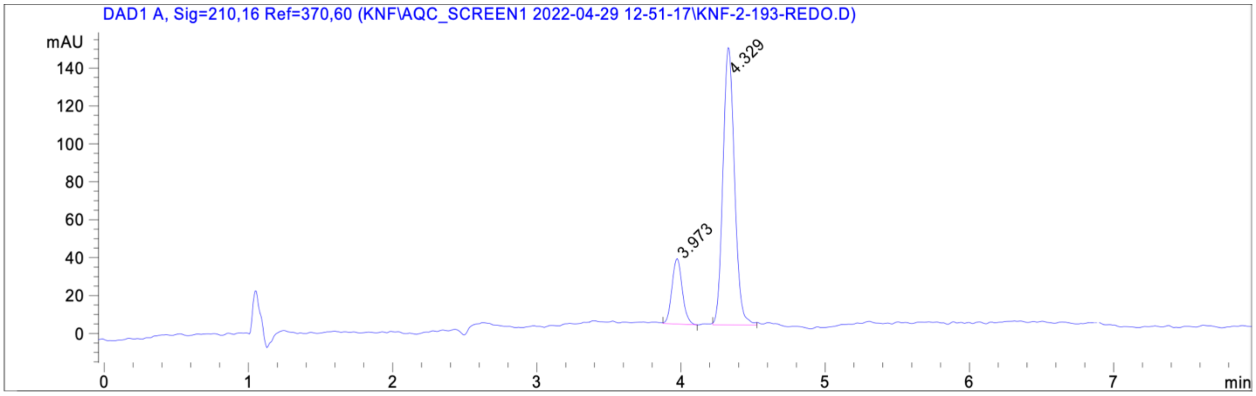

Peak #	RetTime [min]	Type	Width [min]	Area [mAU*s]	Height [mAU]	Area %
1	3.973	BB	0.0781	171.79271	34.50575	17.3986
2	4.329	BB	0.0868	815.60052	146.78046	82.6014

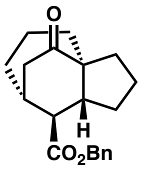

11d

benzyl (3a*R*,7*R*,8*S*,8a*R*)-10-oxooctahydro-1*H*-3a,7-ethanoazulene-8-carboxylate (11d)

Prepared from **10d** following General Procedure A. Purification by flash column chromatography (0–35% EtOAc/hexanes) afforded the title compound as a colorless oil (51.8 mg, 0.166 mmol, 83% yield, 97% ee).

^{1}H NMR (400 MHz, $CDCl_3$): δ 7.40 – 7.31 (m, 5H), 5.16 (d, *J* = 12.4 Hz, 1H), 5.12 (d, *J* = 12.3 Hz, 1H), 2.70 – 2.63 (m, 2H), 2.64 – 2.56 (m, 1H), 2.25 (ddd, *J* = 18.5, 2.0, 1.0 Hz, 1H), 2.08 (td, *J* = 10.5, 7.8 Hz, 1H), 2.03 – 1.94 (m, 1H), 1.93 – 1.79 (m, 3H), 1.78 – 1.60 (m, 4H), 1.59 – 1.43 (m, 4H).

^{13}C NMR (100 MHz, $CDCl_3$): δ 215.7, 175.8, 136.0, 128.8, 128.5, 128.3, 66.7, 58.0, 50.2, 45.4, 41.0, 35.7, 33.6, 33.3, 32.0, 28.0, 21.8, 21.2.

IR (Neat Film, NaCl): 2934, 2873, 1727, 1713, 1455, 1161 cm^{-1}.

HRMS (MM: FD+): *m/z* calc'd for $C_{20}H_{24}O_3$ $[M+H]^+$: 312.1720, found 312.1734.

Optical Rotation: $[\alpha]_D^{21}$ +21.4 (c 1.00, $CHCl_3$).

SFC conditions: 15% IPA, 2.5 mL/min, Chiralpak AD-H column, λ = 210 nm, t_R (min): minor = 4.70, major = 6.23.

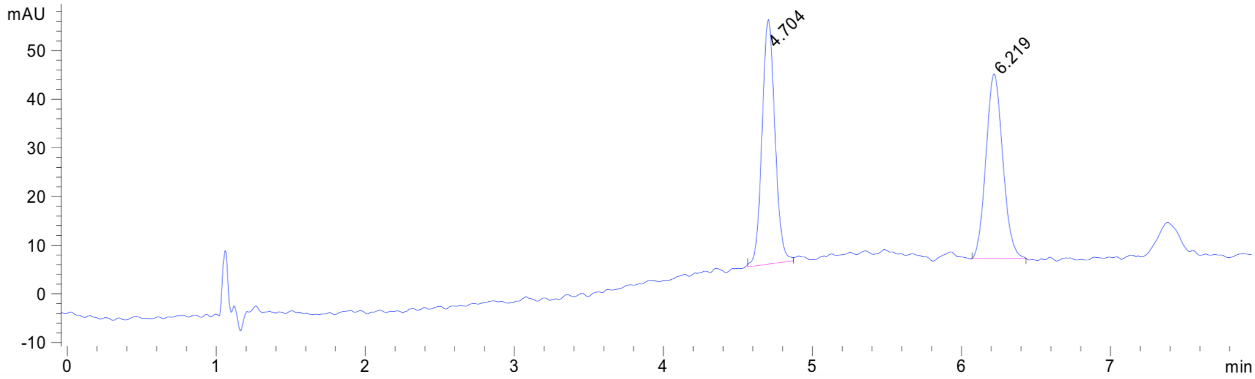

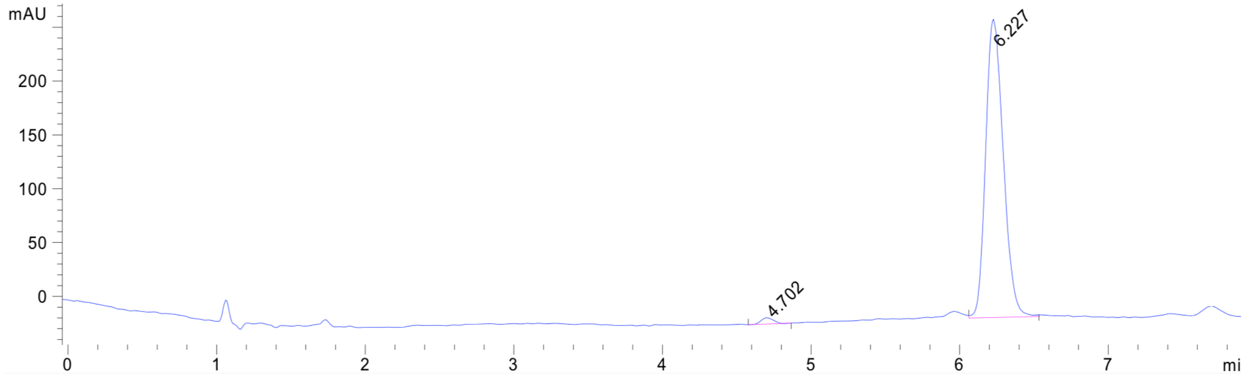

Peak #	RetTime [min]	Type	Width [min]	Area [mAU*s]	Height [mAU]	Area %
1	4.702	BB	0.0874	33.07693	5.90439	1.4241
2	6.227	VB	0.1284	2289.63403	277.00049	98.5759
Totals :				2322.71096	282.90488	

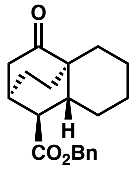

11f

benzyl (1*S*,2*R*,4a*R*,8a*R*)-4-oxooctahydro-2*H*-2,4a-ethanonaphthalene-1-carboxylate (11f)

Prepared from **10f** following General Procedure A. Purification by flash column chromatography (0–35% EtOAc/hexanes) afforded the title compound as a colorless oil (26.3 mg, 0.084 mmol, 42% yield, 92% ee).

^{1}H NMR (400 MHz, $CDCl_3$): δ 7.39 – 7.29 (m, 5H), 5.16 (d, *J* = 12.4 Hz, 1H), 5.10 (d, *J* = 12.2 Hz, 1H), 2.47 (dt, *J* = 17.0, 2.8 Hz, 2H), 2.28 – 2.19 (m, 2H), 2.14 (ddd, *J* = 19.7, 3.8, 1.8 Hz, 1H), 2.01 (dddd, *J* = 11.8, 6.8, 4.5, 1.7 Hz, 1H), 1.87 (ddtd, *J* = 12.8, 4.5, 3.4, 1.6 Hz, 1H), 1.83 – 1.71 (m, 1H), 1.71 – 1.56 (m, 4H), 1.51 – 1.11 (m, 5H).

^{13}C NMR (100 MHz, $CDCl_3$): δ 216.3, 174.5, 136.0, 128.7, 128.4, 128.2, 66.7, 49.8, 45.1, 40.5, 37.1, 30.9, 30.0, 28.9, 26.2, 25.6, 21.7, 21.1.

IR (Neat Film, NaCl): 2928, 2856, 1721, 1170 cm^{-1}.

HRMS (MM: FD+): *m/z* calc'd for $C_{20}H_{24}O_3$ $[M]^+$: 312.1720, found 312.1732.

Optical Rotation: $[\alpha]_D^{21}$ –15.5 (c 1.00, $CHCl_3$).

SFC conditions: 15% IPA, 2.5 mL/min, Chiralpak AD-H column, λ = 210 nm, t_R (min): minor = 4.70, major = 6.23.

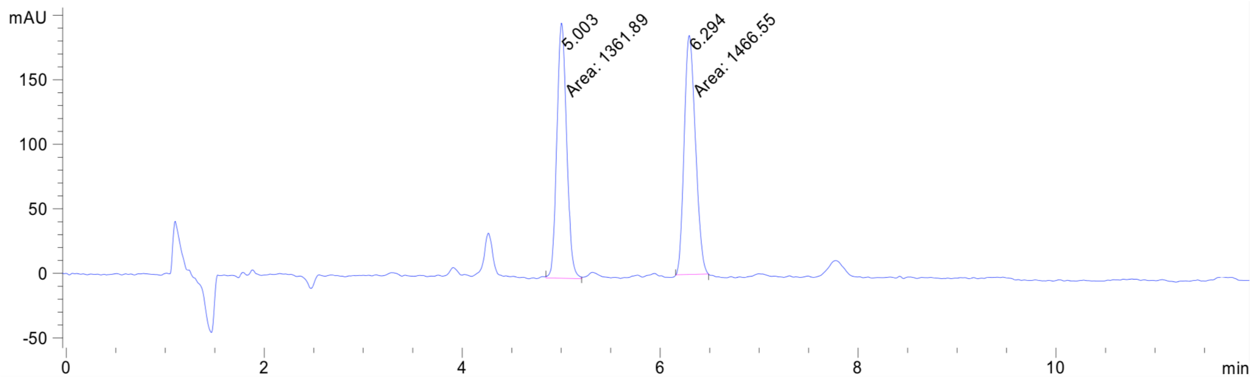

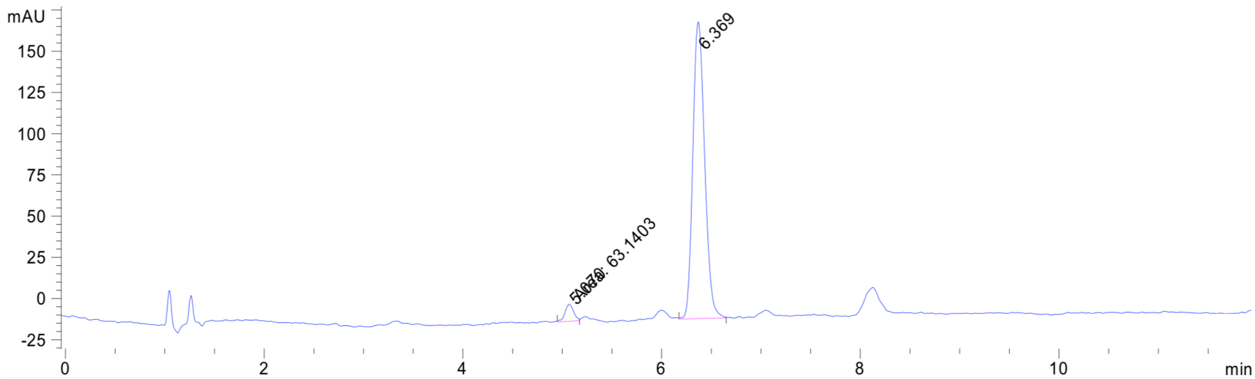


```
Peak RetTime Type  Width      Area       Height      Area
  #   [min]        [min]    [mAU*s]      [mAU]        %
----|-------|----|-------|----------|----------|--------|
   1   5.070 MM    0.1028   63.14032   10.23250   4.0756
   2   6.369 VB    0.1303 1486.08533  179.99663  95.9244

Totals :                  1549.22564  190.22913
```

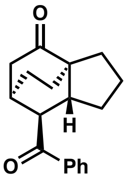

11j

(3a*R*,6*R*,7*S*,7a*R*)-7-benzoylhexahydro-3a,6-ethanoinden-4(1*H*)-one (11j)

Prepared from **10j** following General Procedure A. Purification by flash column chromatography (0–30% EtOAc/hexanes) afforded the title compound as a colorless oil (27.1 mg, 0.101 mmol, 50% yield, 87% ee).

^{1}H NMR (400 MHz, $CDCl_3$): δ 7.97 – 7.94 (m, 2H), 7.61 – 7.56 (m, 1H), 7.51 – 7.46 (m, 2H), 3.42 (d, *J* = 8.5 Hz, 1H), 2.58 – 2.50 (m, 2H), 2.41 – 2.38 (m, 1H), 2.16 (ddd, *J* = 13.7, 11.1, 6.2 Hz, 1H), 2.08 – 1.87 (m, 4H), 1.82 – 1.74 (m, 1H), 1.70 – 1.50 (m, 4H), 1.25 (ddd, *J* = 14.0, 9.2, 5.1 Hz, 1H).

^{13}C NMR (100 MHz, $CDCl_3$): δ 214.9, 201.0, 136.4, 133.4, 128.9, 128.5, 54.2, 49.4, 41.4, 40.8, 34.1, 28.9, 27.8, 26.5, 25.6, 22.7.

IR (Neat Film, NaCl): 2945, 2871, 1720, 1677, 1447, 1217 cm^{-1}.

HRMS (MM: FD+): *m/z* calc'd for $C_{18}H_{20}O_2$ $[M]^+$: 268.1463, found 268.1463.

Optical Rotation: $[\alpha]_D^{21}$ –32.7 (c 1.00, $CHCl_3$).

SFC conditions: 30% IPA, 2.5 mL/min, Chiralpak AD-H column, λ = 210 nm, t_R (min): minor = 2.55, major = 3.40.

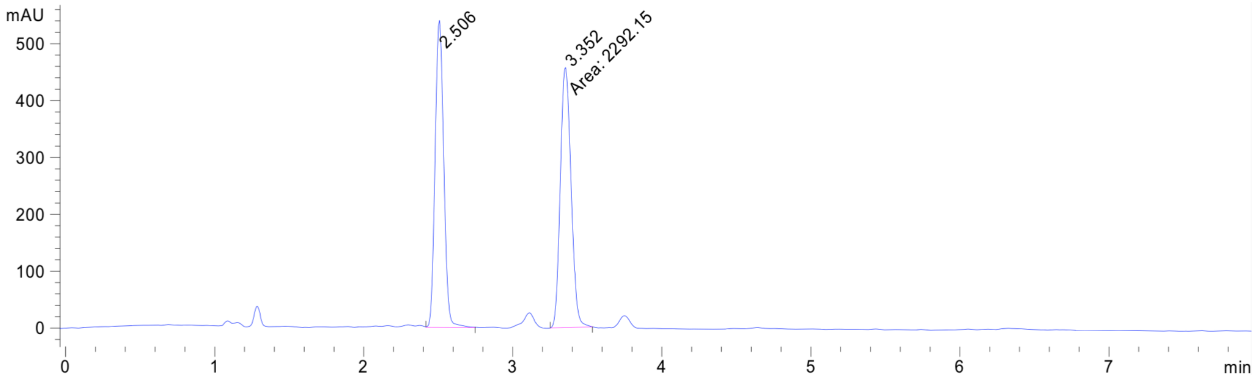

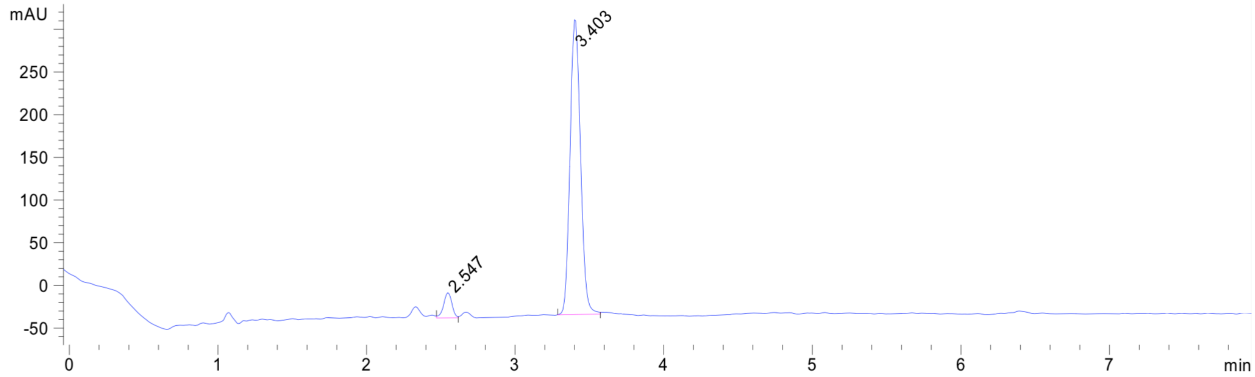

Peak #	RetTime [min]	Type	Width [min]	Area [mAU*s]	Height [mAU]	Area %
1	2.547	VV	0.0595	115.24327	29.56192	6.3730
2	3.403	BB	0.0770	1693.07263	346.39813	93.6270

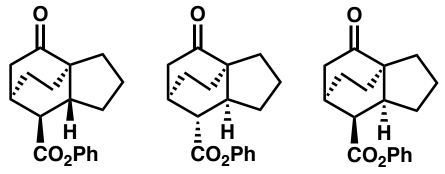

phenyl (3a*R*,6*R*,7*S*,7a*R*)-4-oxooctahydro-3a,6-ethanoindene-7-carboxylate (11k, 11k' and 11k'')

Prepared from **10k** following General Procedure A. Purification by flash column chromatography (0–35% EtOAc/hexanes) afforded the title compound as a colorless oil (47.2 mg, 0.166 mmol, 83% yield, 14.8:1.6:1.0 *endo-trans*/*endo-cis*/*exo-trans*, 88% ee (*endo-trans*)). Crude analysis by ^{1}H NMR affords a 10.4:1.4:1.0 ratio of *endo-trans*/*exo-trans*/*endo-cis*. The diastereomers were subsequently separated by preparative HPLC (15% IPA/hexanes, 25 mL/min, Chiralpak AD-H column) for independent characterization. Absolute and relative stereochemistry were assigned/confirmed by VCD where applicable (vida infra) in addition to 2D NMR.

11k (endo-trans):

^{1}H NMR (400 MHz, CDCl$_3$): δ 7.41 – 7.34 (m, 2H), 7.25 – 7.20 (m, 1H), 7.09 – 7.04 (m, 2H), 2.70 (d, *J* = 8.7 Hz, 1H), 2.67 – 2.60 (m, 2H), 2.31 (dddd, *J* = 10.6, 8.8, 7.3, 1.7 Hz, 1H), 2.23 – 2.09 (m, 3H), 1.97 – 1.76 (m, 3H), 1.67 (ddddd, *J* = 13.5, 11.1, 9.0, 6.2, 4.5 Hz, 3H), 1.50 (ddt, *J* = 12.5, 10.6, 1.7 Hz, 1H), 1.31 – 1.17 (m, 1H).

^{13}C NMR (100 MHz, CDCl$_3$): δ 214.8, 173.4, 150.7, 129.6, 126.0, 121.5, 54.2, 47.5, 43.4, 41.2, 32.9, 29.1, 27.3, 26.5, 25.0, 22.5.

IR (Neat Film, NaCl): 2948, 2872, 1750, 1721, 1592, 1492, 1192, 1144 cm^{-1}.

HRMS (MM: FD+): *m/z* calc'd for $C_{18}H_{20}O_3$ [M]$^+$: 284.1412, found 284.1411.

Optical Rotation: $[\alpha]_D^{21}$ –16.7 (c 0.20, CHCl$_3$). *(single major enantiomer of 11j)*

SFC conditions: 15% IPA, 2.5 mL/min, Chiralpak AD-H column, λ = 210 nm, t_R (min): minor = 4.09, major = 6.08.

Isolated sample of ***11j''*** (both enantiomers)*:*

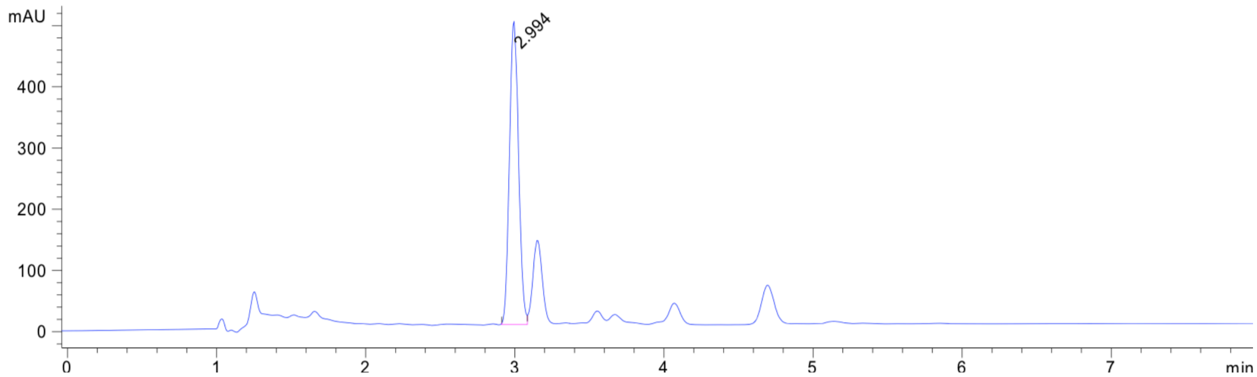

Isolated sample minor enantiomer of ***11k****:*

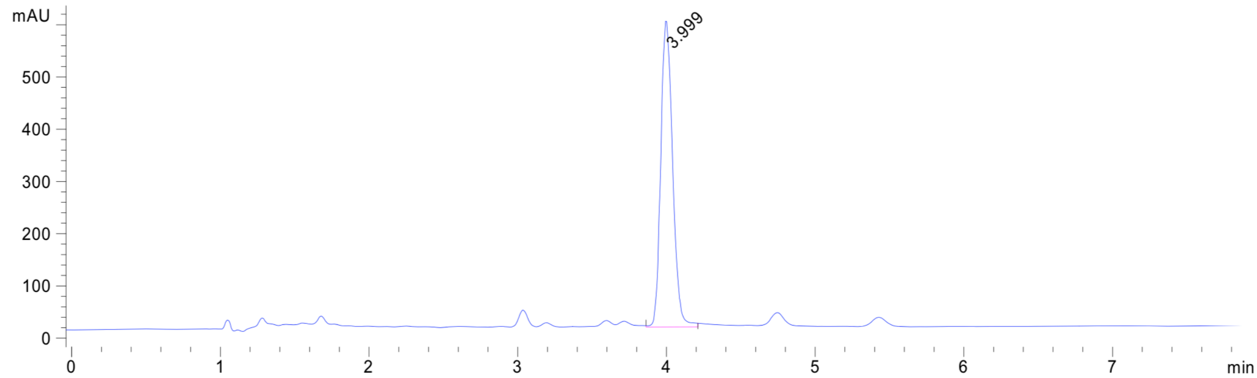

Isolated sample of ***11k''*** (both enantiomers)*:*

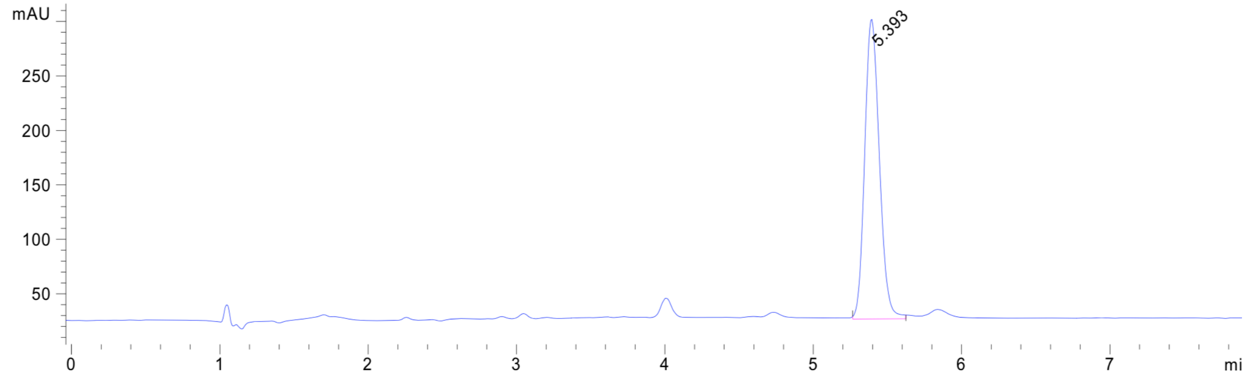

Isolated sample minor enantiomer of ***11k****:*

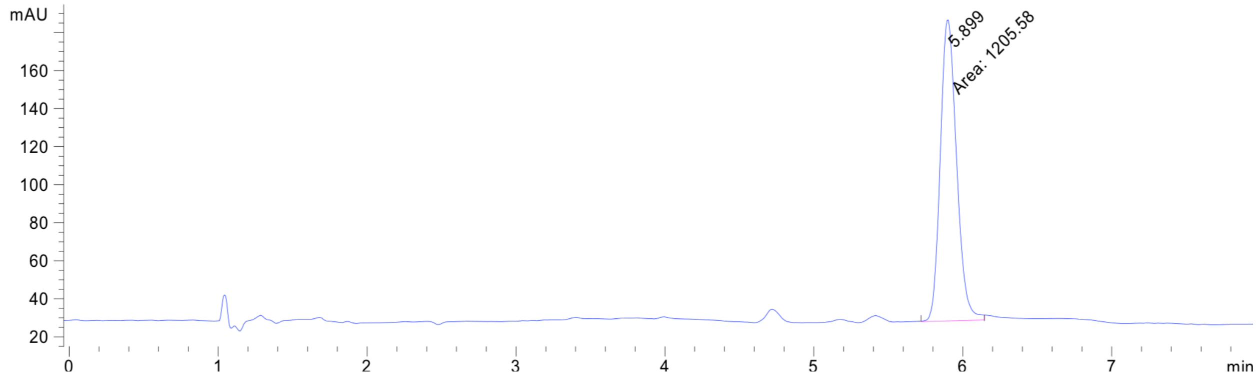

SFC trace from reaction (purified but mixture of diastereomers above):

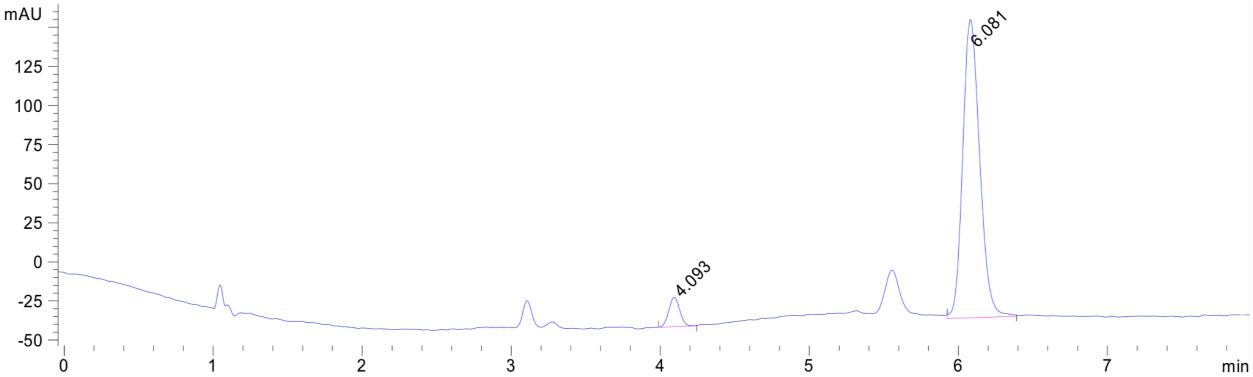

Peak #	RetTime [min]	Type	Width [min]	Area [mAU*s]	Height [mAU]	Area %
1	4.093	BB	0.0818	96.53783	18.83285	5.9918
2	6.081	BB	0.1246	1514.61462	190.92795	94.0082
Totals :				1611.15245	209.76080	

11k' (exo-trans):

^{1}H NMR (400 MHz, $CDCl_3$): δ 7.39 – 7.34 (m, 2H), 7.25 – 7.19 (m, 1H), 7.06 – 7.01 (m, 2H), 3.30 (dt, *J* = 12.0, 2.3 Hz, 1H), 3.08 (dt, *J* = 19.5, 2.8 Hz, 1H), 2.58 (h, *J* = 2.8 Hz, 1H), 2.47 – 2.33 (m, 2H), 2.21 (dt, *J* = 19.6, 2.4 Hz, 1H), 2.08 – 2.01 (m, 2H), 1.93 – 1.71 (m, 5H), 1.70 – 1.59 (m, 1H), 1.04 (ddd, *J* = 12.8, 11.4, 6.7 Hz, 1H).

^{13}C NMR (100 MHz, $CDCl_3$): δ 215.2, 171.8, 150.6, 129.6, 126.0, 121.7, 54.6, 45.8, 44.0, 40.1, 31.4, 28.4, 28.2, 27.0, 26.3, 21.8.

IR ($CDCl_3$ solution): 2951, 2870, 1751, 1717, 1194, 1163, 1146 cm^{-1}.

HRMS (MM: FD+): *m/z* calc'd for $C_{18}H_{20}O_3$ $[M]^+$: 284.1412, found 284.1417.

11k'' (endo-cis):

^{1}H NMR (400 MHz, $CDCl_3$): δ 7.43 – 7.36 (m, 2H), 7.27 – 7.22 (m, 1H), 7.12 – 7.07 (m, 2H), 2.76 – 2.67 (m, 1H), 2.58 (dt, *J* = 8.3, 1.7 Hz, 1H), 2.48 – 2.33 (m, 4H), 2.12 – 2.01 (m, 2H), 1.91 – 1.63 (m, 5H), 1.10 (ddd, *J* = 13.0, 11.2, 6.5 Hz, 1H), 1.01 (ddd, *J* = 12.4, 9.5, 2.8 Hz, 1H).

^{13}C NMR (100 MHz, CDCl$_3$): δ 215.1, 172.9, 150.9, 129.6, 126.1, 121.6, 53.8, 49.4, 46.0, 44.2, 32.7, 32.0, 28.6, 27.4, 22.6, 21.5.

IR (CDCl$_3$ solution): 2945, 2872, 1751, 1717, 1194, 1163, 1130 cm^{-1}.

HRMS (MM: FD+): *m/z* calc'd for $C_{18}H_{20}O_3$ [M]$^+$: 284.1412, found 284.1407.

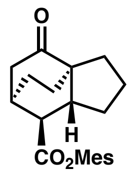

11l

mesityl (3a*R*,6*R*,7*S*,7a*R*)-4-oxooctahydro-3a,6-ethanoindene-7-carboxylate (11l)

Prepared from **10l** following General Procedure A. Purification by flash column chromatography (0–35% EtOAc/hexanes) afforded the title compound as a colorless oil (58.6 mg, 0.180 mmol, 90% yield, 89% ee).

^{1}H NMR (400 MHz, CDCl$_3$):) δ 6.87 (s, 2H), 2.75 (d, *J* = 8.8 Hz, 1H), 2.72 – 2.65 (m, 2H), 2.36 (dddd, *J* = 10.6, 8.9, 7.3, 1.6 Hz, 1H), 2.26 (s, 3H), 2.24 – 2.09 (m, 3H), 2.08 (s, 6H), 1.98 – 1.78 (m, 3H), 1.75 – 1.61 (m, 3H), 1.55 – 1.48 (m, 1H), 1.31 – 1.24 (m, 1H).

^{13}C NMR (100 MHz, CDCl$_3$): δ 214.8, 172.8, 145.9, 135.6, 129.5, 129.5, 54.2, 47.4, 43.5, 41.3, 33.2, 29.2, 27.5, 26.5, 25.1, 22.6, 20.9, 16.4.

IR (Neat Film, NaCl): 2946, 2873, 1747, 1723, 1485, 1458, 1189, 1137 cm^{-1}.

HRMS (MM: FD+): *m/z* calc'd for $C_{21}H_{26}O_3$ [M]$^+$: 326.1877, found 326.1886.

Optical Rotation: $[\alpha]_D^{21}$ –25.8 (c 1.00, CHCl$_3$).

SFC conditions: 15% IPA, 2.5 mL/min, Chiralpak AD-H column, λ = 210 nm, t_R (min): minor = 4.06, major = 4.33.

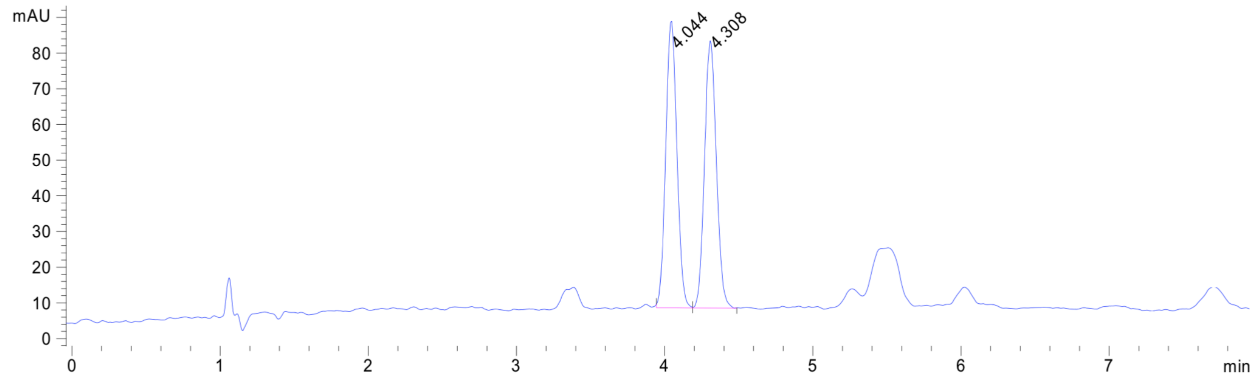

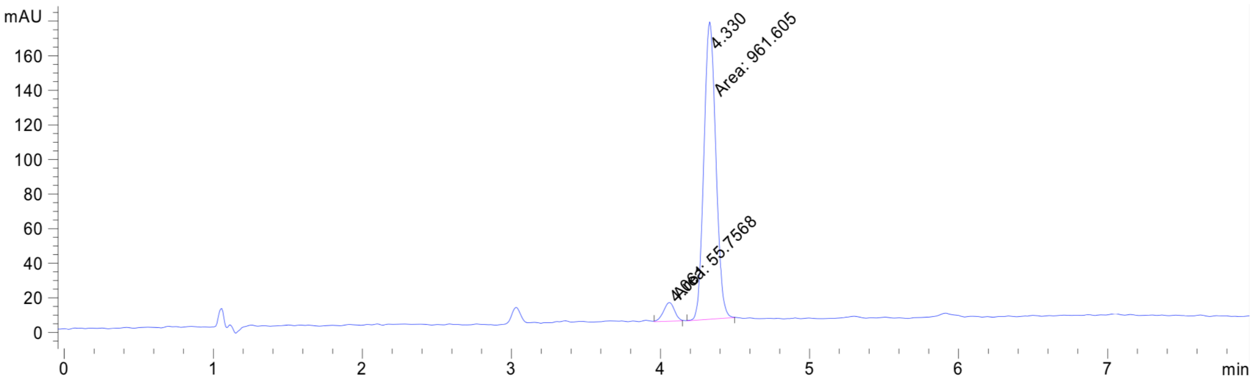

Peak #	RetTime [min]	Type	Width [min]	Area [mAU*s]	Height [mAU]	Area %
1	4.061	MM	0.0854	55.75682	10.87858	5.4805
2	4.330	MM	0.0931	961.60522	172.17397	94.5195
Totals :				1017.36205	183.05255	

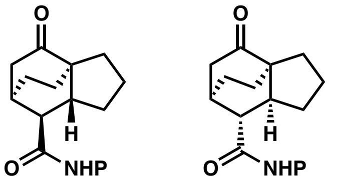

11m **(endo)** **11m'** **(exo)**

1,3-dioxoisoindolin-2-yl (3a*R*,6*R*,7*S*,7a*R*)-4-oxooctahydro-3a,6-ethanoindene-7-carboxylate (11m and 11m')

Prepared from **10m** following General Procedure A. Purification by flash column chromatography (0–35% EtOAc/hexanes) afforded the title compounds as colorless oils (**Endo**: 37.7 mg, 0.106 mmol, 53% yield, 62% ee; **Exo**: 8.6 mg, 0.024 mmol, 12% yield, 62% ee).

11m (endo):

^{1}H NMR (400 MHz, CDCl$_3$): δ 7.92 – 7.85 (m, 2H), 7.83 – 7.75 (m, 2H), 2.84 (dd, *J* = 8.7, 1.3 Hz, 1H), 2.71 (m, 1H), 2.64 (dt, *J* = 18.9, 2.5 Hz, 1H), 2.46 – 2.28 (m, 1H), 2.28 – 2.18 (m, 2H), 2.13 (m, 1H), 1.98 – 1.76 (m, 3H), 1.76 – 1.60 (m, 3H), 1.56 – 1.48 (m, 1H), 1.34 – 1.19 (m, 1H).

^{13}C NMR (100 MHz, CDCl$_3$): δ 214.0, 171.4, 162.1, 135.0, 129.0, 124.2, 54.1, 44.8, 43.3, 41.0, 33.2, 28.9, 27.2, 26.4, 24.9, 22.4.

IR (Neat Film, NaCl): 2948, 2873, 1782, 1742, 1718, 1466, 1362, 1185 cm^{-1}.

HRMS (MM: FD+): *m/z* calc'd for $C_{20}H_{19}NO_5$ [M]$^+$: 353.1263, found 353.1251.

Optical Rotation: $[\alpha]_D^{21}$ –0.2 (c 1.00, CHCl$_3$).

SFC conditions: 30% IPA, 2.5 mL/min, Chiralpak IC column, l = 210 nm, t_R (min): minor = 4.03, major = 3.00

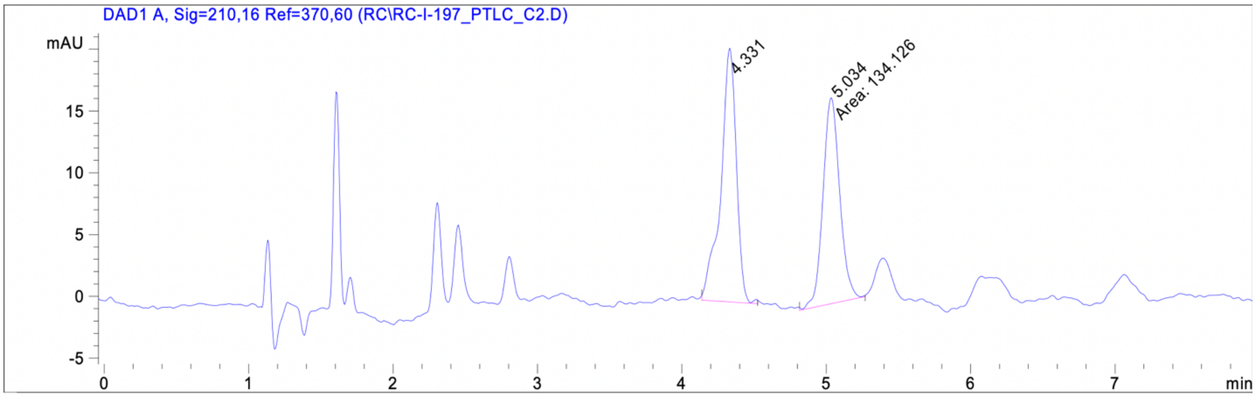

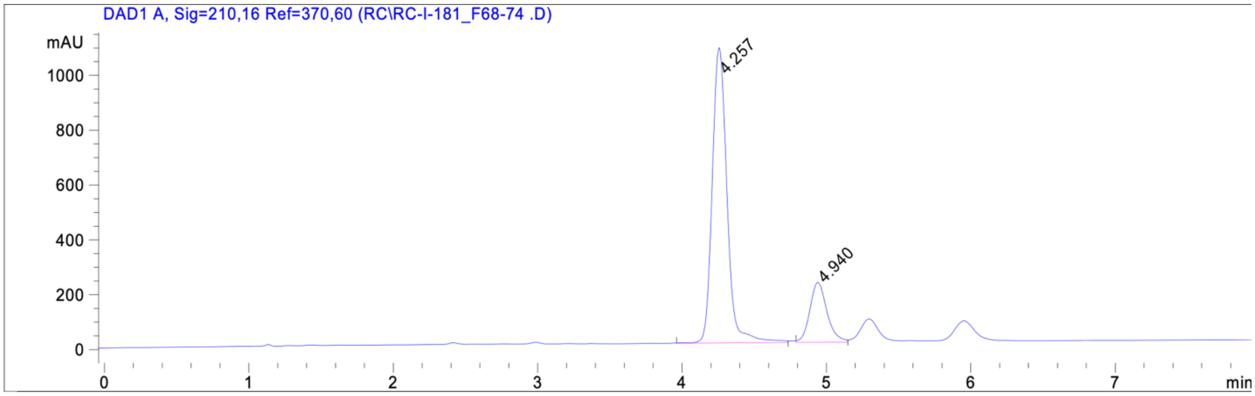

Peak #	RetTime [min]	Type	Width [min]	Area [mAU*s]	Height [mAU]	Area %
1	4.257	BB	0.1113	7675.99756	1075.37146	80.9443
2	4.940	BV	0.1286	1807.06042	218.16873	19.0557

11m' (exo):

^{1}H NMR (400 MHz, CDCl$_3$): δ 7.90 (dd, *J* = 5.5, 3.1 Hz, 2H), 7.81 (dd, *J* = 5.5, 3.1 Hz, 2H), 2.77 (tt, *J* = 3.9, 2.2 Hz, 1H), 2.73 (d, *J* = 8.4 Hz, 1H), 2.47 – 2.28 (m, 4H), 2.19 – 2.02 (m, 2H), 1.92 – 1.71 (m, 4H), 1.71 – 1.61 (m, 1H), 1.11 (ddd, *J* = 13.1, 11.2, 6.6 Hz, 1H), 1.08 – 0.95 (m, 1H).

^{13}C NMR (100 MHz, CDCl$_3$): δ 214.2, 170.8, 162.1, 135.0, 129.1, 124.2, 53.7, 46.7, 46.0, 44.1, 32.9, 31.8, 28.4, 27.2, 22.5, 21.2.

IR (Neat Film, NaCl): 2947, 2868, 1809, 1784, 1743, 1717, 1466, 1362, 1185 cm^{-1}.

HRMS (MM: FD+): *m/z* calc'd for $C_{20}H_{19}NO_5$ [M]$^+$: 353.1263, found 353.1261.

Optical Rotation: $[\alpha]_D^{21}$ –13.6 (c 0.81, CHCl$_3$).

SFC conditions: 30% IPA, 2.5 mL/min, Chiralpak IC column, l = 210 nm, t_R (min): minor = 4.98, major = 4.29.

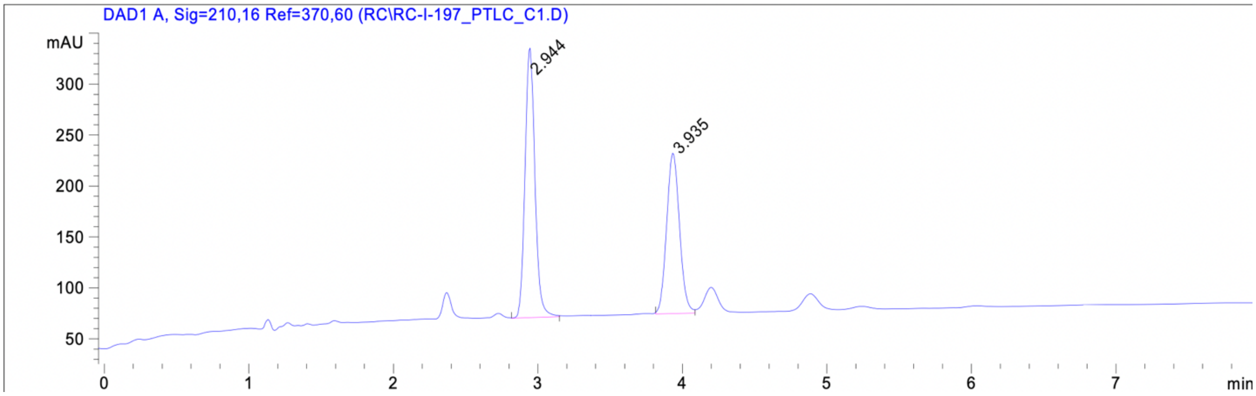

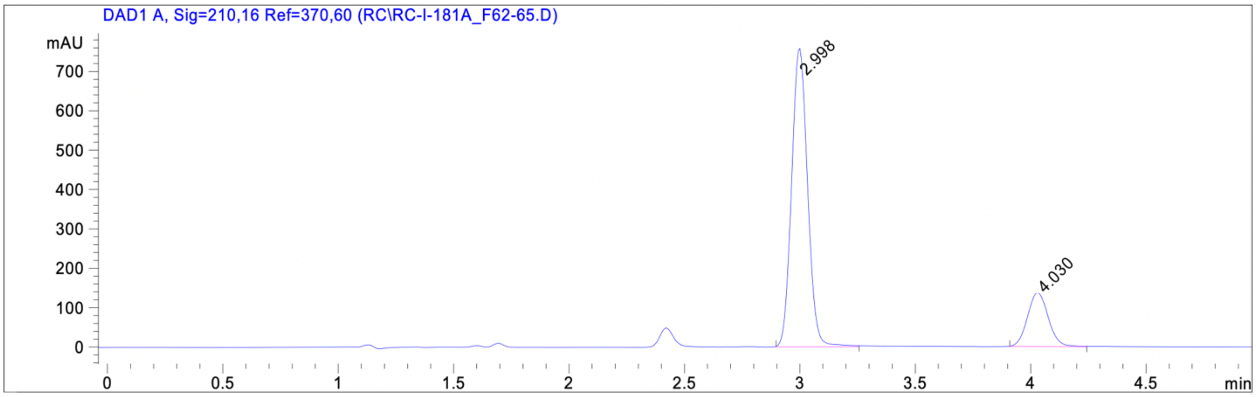

Peak #	RetTime [min]	Type	Width [min]	Area [mAU*s]	Height [mAU]	Area %
1	2.998	BB	0.0766	3651.06763	751.95715	81.0529
2	4.030	BB	0.1011	853.48383	136.20329	18.9471

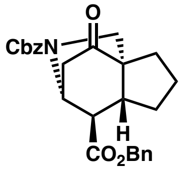

11n

dibenzyl (3aR,4R,5R,7aS)-7-oxooctahydro-5,7a-(epiminomethano)indene-4,9-dicarboxylate (11n)

Prepared from **10n** following General Procedure A. Purification by flash column chromatography (0–40 % EtOAc/hexanes) afforded the title compound as a colorless oil (76.1 mg, 0.176 mmol, 88% yield, 14.3:1 endo/exo, 91% ee (endo)).

^{1}H NMR (400 MHz, $CDCl_3$): δ 7.35 (dd, J = 7.1, 4.9 Hz, 10H), 5.21 – 5.07 (m, 4H), 4.90 – 4.74 (m, 1H), 3.49 (dd, J = 12.1, 5.8 Hz, 1H), 3.40 – 3.27 (m, 1H), 2.88 (t, J = 8.9 Hz, 1H), 2.66 – 2.42 (m, 2H), 2.30 (p, J = 9.3 Hz, 1H), 2.23 – 2.10 (m, 2H), 1.91 – 1.56 (m, 3H), 1.33 – 1.14 (m, 1H).

^{13}C NMR (100 MHz, $CDCl_3$): δ 210.1, 209.7, 172.2, 154.4, 136.3, 135.5, 128.9, 128.7, 128.7, 128.4, 128.4, 128.4, 128.3, 128.2, 128.1, 67.6, 67.2, 55.9, 55.8, 50.4, 50.0, 48.8, 48.6, 45.7, 45.6, 42.6, 42.6, 41.7, 41.6, 29.0, 28.9, 24.1, 22.9.

IR (Neat Film, NaCl): 3399, 2963, 2874, 2357, 1729, 1700, 1652, 1414, 1288, 1156, 1115, 748, 681 cm^{-1}.

HRMS (MM: FD+): *m/z* calc'd for $C_{26}H_{27}NO_5$ $[M]^+$: 433.1889, found 433.1874.

Optical Rotation: $[\alpha]_D^{21}$ –39.8 (c 0.75, $CHCl_3$).

SFC conditions: 15% IPA, 2.5 mL/min, Chiralpak AD-H column, l = 210 nm, t_R (min): minor = 9.19, major = 11.59.

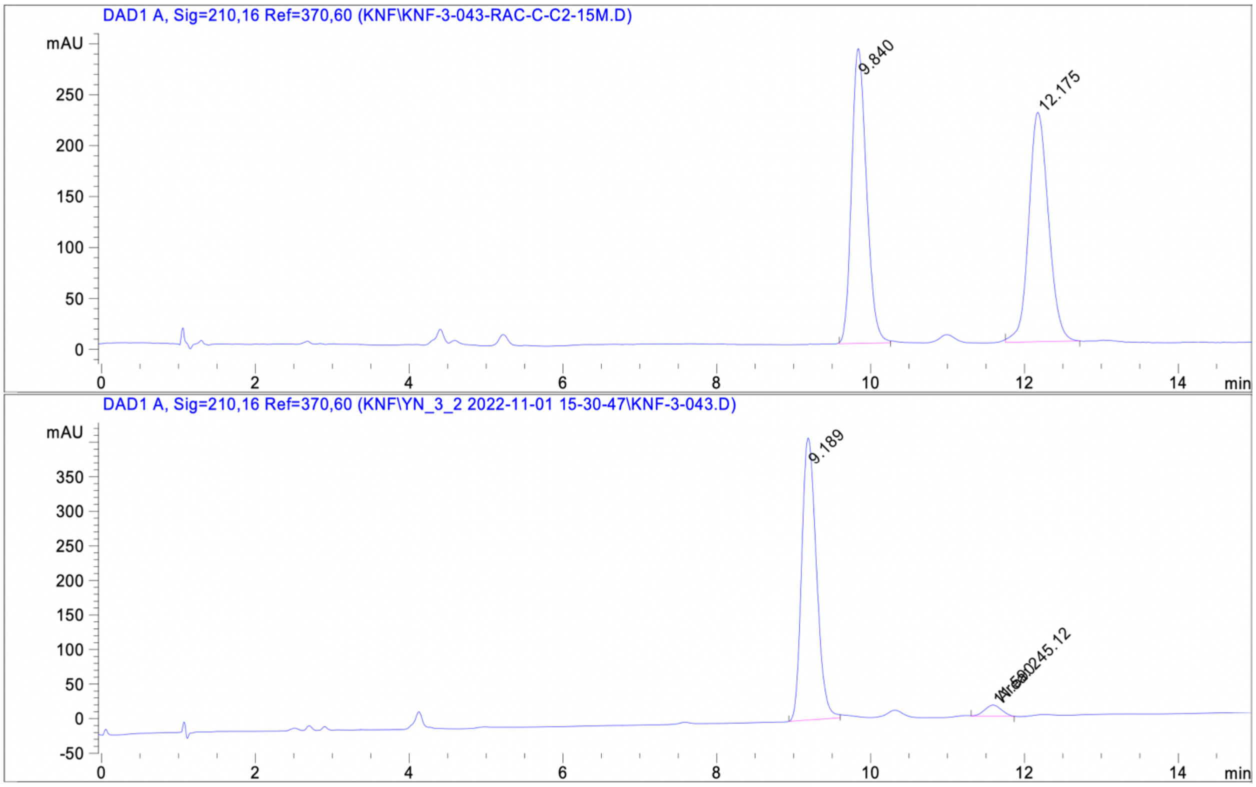

Peak #	RetTime [min]	Type	Width [min]	Area [mAU*s]	Height [mAU]	Area %
1	9.189	BB	0.2090	5429.62988	408.62552	95.6805
2	11.590	MF	0.2557	245.11951	15.97795	4.3195

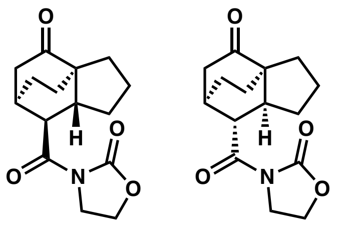

11o (endo) ***11o' (exo)***

3-((3a*R*,6*R*,7*S*,7a*R*)-4-oxooctahydro-3a,6-ethanoindene-7-carbonyl)oxazolidin-2-one (11o and 11o')

Prepared from **10o** following General Procedure A. Purification by flash column chromatography (0–90 % EtOAc/hexanes) afforded the title compound as a colorless oil (51.2 mg, 0.185 mmol, 92% yield, 1.1:1 endo/exo (ratio from crude ^{1}H NMR analysis), 84% ee (endo), 79% ee (exo)).

11o (endo):

^{1}H NMR (400 MHz, CDCl$_3$): δ 4.43 (t, *J* = 8.1 Hz, 2H), 4.15 – 4.00 (m, 2H), 3.54 – 3.47 (m, 1H), 2.66 (ddd, *J* = 12.4, 8.3, 6.9 Hz, 1H), 2.52 – 2.25 (m, 4H), 1.92 – 1.68 (m, 5H), 1.67 – 1.53 (m, 2H), 1.08 (ddd, *J* = 12.9, 11.2, 6.4 Hz, 1H), 0.89 (tt, *J* = 12.3, 9.5 Hz, 1H).

^{13}C NMR (100 MHz, CDCl$_3$): δ 215.5, 173.8, 153.4, 62.1, 53.7, 48.3, 44.1, 44.0, 43.1, 33.8, 31.3, 28.7, 27.3, 22.8, 20.8.

IR (Neat Film, NaCl): 2942, 2867, 1775, 1714, 1693, 1387, 1267, 1222, 1040 cm^{-1}.

HRMS (MM: FD+): *m/z* calc'd for $C_{15}H_{19}NO_4$ [M]$^+$: 277.1314, found 277.1321.

Optical Rotation: $[\alpha]_D^{21}$ –39.5 (c 1.00, CHCl$_3$).

SFC conditions: 20% IPA, 2.5 mL/min, Chiralpak AD-H column, λ = 210 nm, t_R (min): minor = 4.15, major = 5.17.

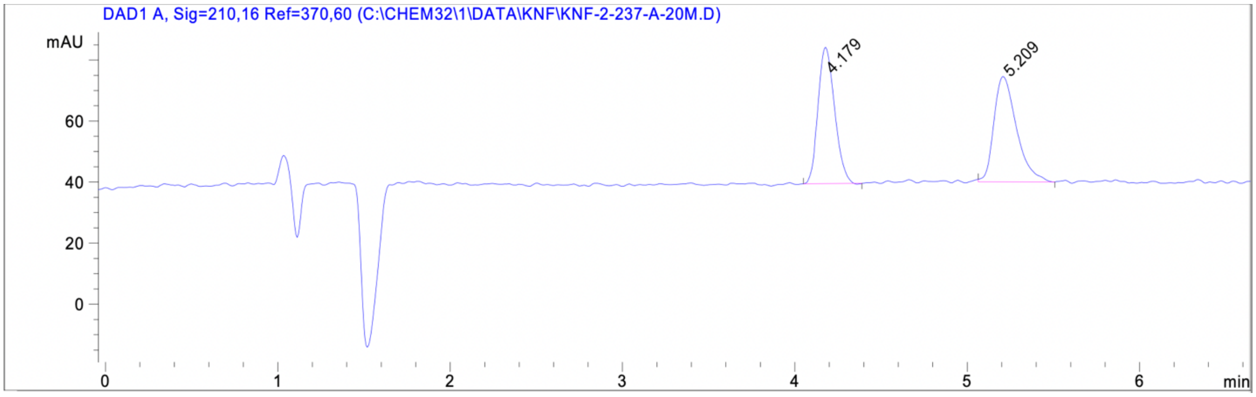

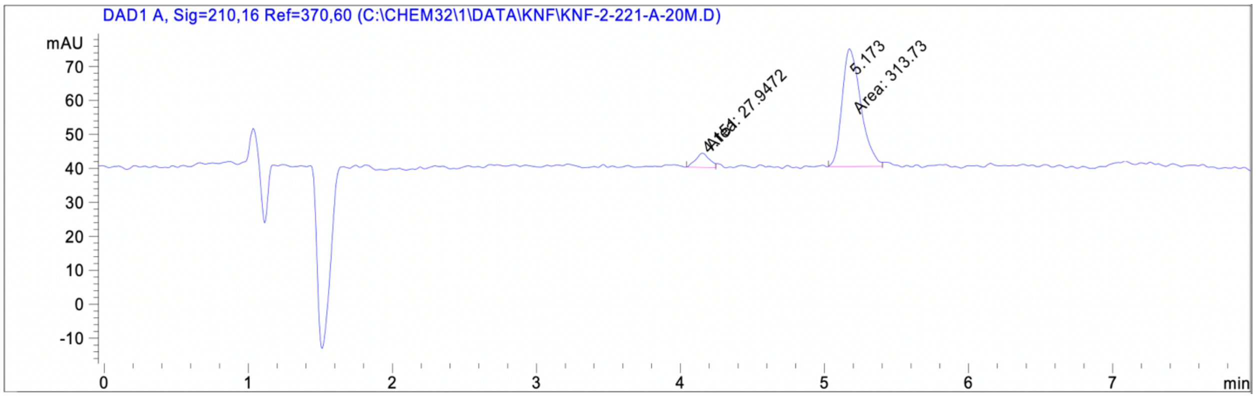

Peak #	RetTime [min]	Type	Width [min]	Area [mAU*s]	Height [mAU]	Area %
1	4.151	MF	0.1118	27.94722	4.16664	8.1794
2	5.173	MF	0.1506	313.73016	34.72126	91.8206

11o' (exo):

^{1}H NMR (400 MHz, CDCl$_3$): δ 4.43 (t, *J* = 8.1 Hz, 2H), 4.13 – 3.97 (m, 2H), 3.71 (d, *J* = 8.8 Hz, 1H), 2.59 – 2.41 (m, 2H), 2.35 (dd, *J* = 3.5, 2.3 Hz, 1H), 2.19 – 1.84 (m, 5H), 1.81 – 1.41 (m, 6H), 1.27 – 1.18 (m, 1H).

^{13}C NMR (100 MHz, CDCl$_3$): δ 214.9, 174.6, 153.5, 62.13, 54.1, 45.6, 43.1, 41.2, 40.6, 34.5, 28.4, 27.3, 26.5, 25.3, 22.7.

IR (Neat Film, NaCl): 2949, 2872, 1775, 1718, 1692, 1387, 1221,1040, 759 cm^{-1}.

HRMS (MM: FD+): *m/z* calc'd for $C_{15}H_{19}NO_4$ [M]$^+$: 277.1314, found 277.1317.

Optical Rotation: $[\alpha]_D^{21}$ –5.8 (c 1.00, CHCl$_3$).

SFC conditions: 15% IPA, 2.5 mL/min, Chiralpak AD-H column, λ = 210 nm, t_R (min): minor = 6.80, major = 6.38.

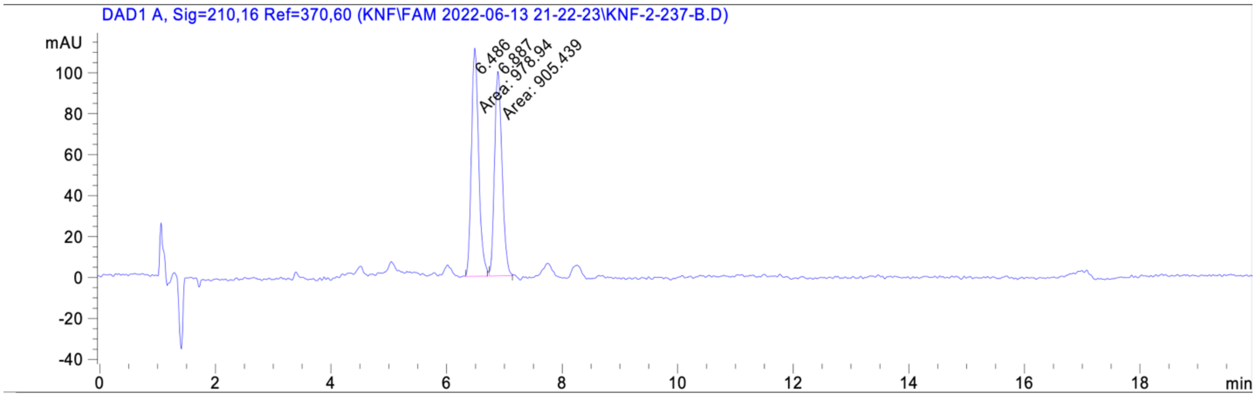

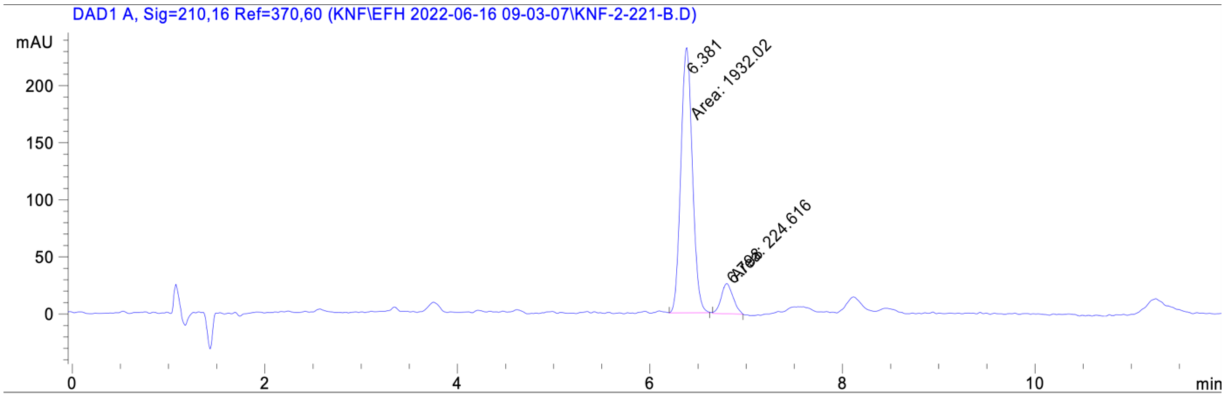

Peak #	RetTime [min]	Type	Width [min]	Area [mAU*s]	Height [mAU]	Area %
1	6.381	MM	0.1384	1932.02209	232.63237	89.5849
2	6.798	MM	0.1418	224.61641	26.40123	10.4151

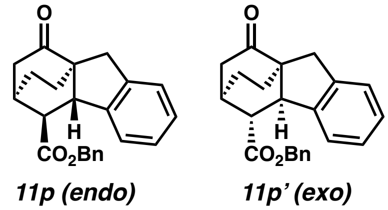

benzyl (3*R*,4*S*,4a*S*,9a*S*)-1-oxo-1,2,3,4,4a,9-hexahydro-3,9a-ethanofluorene-4-carboxylate (11p and 11p')

Prepared from **10p** following General Procedure A. Purification by flash column chromatography (0–35% EtOAc/hexanes) afforded the title compounds as colorless oils (**Endo**: 56.8 mg, 0.156 mmol, 78% yield, 72% ee; **Exo**: 2.0 mg, 5.48 µmol, 3% yield).

11p (endo):

^{1}H NMR (400 MHz, CDCl$_3$): δ 7.28 – 7.20 (m, 5H), 7.14 – 7.05 (m, 4H), 5.13 (d, *J* = 2.4 Hz, 2H), 3.53 (dd, *J* = 9.4, 1.0 Hz, 1H), 3.16 (d, *J* = 15.7 Hz, 1H), 2.74 (dt, *J* = 9.3, 1.1 Hz, 1H), 2.61 (dt, *J* = 18.8, 2.4 Hz, 1H), 2.51 (ttd, *J* = 3.5, 2.2, 1.1 Hz, 1H), 2.31 (d, *J* = 15.8 Hz, 1H), 2.11 (ddd, *J* = 18.8, 3.5, 1.2 Hz, 1H), 1.70 – 1.56 (m, 2H), 1.54 – 1.45 (m, 1H), 1.42 – 1.34 (m, 1H).

^{13}C NMR (100 MHz, CDCl$_3$): δ 214.0, 174.6, 142.3, 140.6, 135.8, 128.8, 128.6, 128.4, 127.3, 127.0, 125.5, 124.3, 67.1, 56.6, 47.8, 45.6, 42.1, 35.0, 33.2, 27.0, 24.8.

IR (Neat Film, NaCl): 2942, 2869, 1726, 1457, 1164 cm^{-1}.

HRMS (MM: FD+): *m/z* calc'd for $C_{23}H_{22}O_3$ $[M+H]^+$: 346.1564, found 346.1571.

Optical Rotation: $[\alpha]_D^{21}$ +1.4 (c 1.00, $CHCl_3$).

SFC conditions: 15% IPA, 2.5 mL/min, Chiralpak AD-H column, λ = 210 nm, t_R (min): minor = 6.74, major = 6.28.

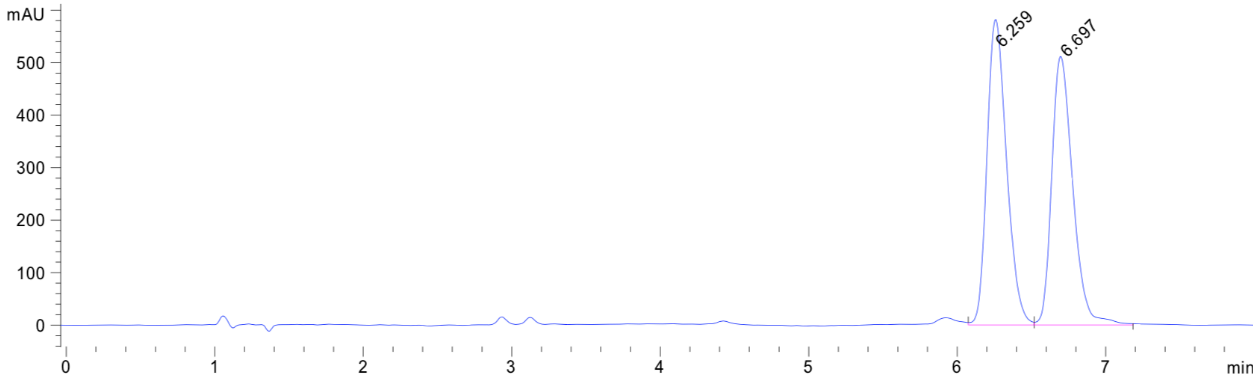

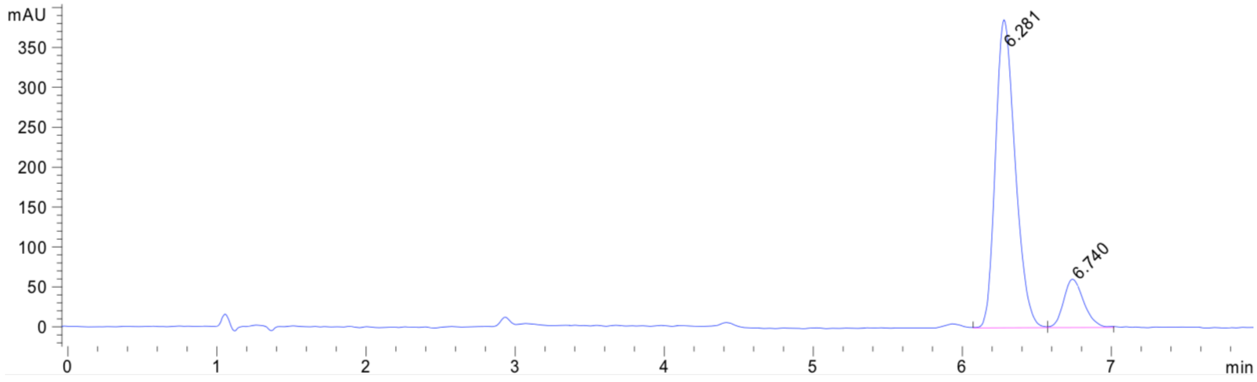

Peak #	RetTime [min]	Type	Width [min]	Area [mAU*s]	Height [mAU]	Area %
1	6.281	VV	0.1439	3561.85718	384.97012	86.1407
2	6.740	VB	0.1505	573.07172	60.50558	13.8593

11p' (exo):

^{1}H NMR (400 MHz, CDCl$_3$): δ 7.41 – 7.34 (m, 5H), 7.24 (d, *J* = 7.1 Hz, 1H), 7.14 – 7.07 (m, 3H), 5.30 (d, *J* = 12.3 Hz, 1H), 5.25 (d, *J* = 12.3 Hz, 1H), 3.85 (d, *J* = 9.4 Hz, 1H), 3.46 (d, *J* = 14.7 Hz, 1H), 2.75 (d, *J* = 9.3 Hz, 1H), 2.69 – 2.65 (m, 1H), 2.44 (d, *J* = 14.9 Hz, 1H), 2.37 (dd, *J* = 18.7, 2.1 Hz, 1H), 2.19 (ddd, *J* = 18.6, 3.4, 2.1 Hz, 1H), 2.15 – 2.07 (m, 2H), 1.90 – 1.84 (m, 1H), 1.81 – 1.76 (m, 1H).

^{13}C NMR (100 MHz, CDCl$_3$): δ 213.5, 174.0, 143.8, 142.7, 135.9, 128.8, 128.6, 128.4, 127.2, 126.6, 124.8, 122.5, 67.1, 56.4, 49.9, 48.3, 44.1, 35.3, 31.9, 27.5, 21.8.

IR (Neat Film, NaCl): 2918, 1727, 1161 cm^{-1}.

HRMS (MM: FD+): *m/z* calc'd for $C_{23}H_{22}O_3$ [M+H]$^+$: 346.1569, found 346.1568.

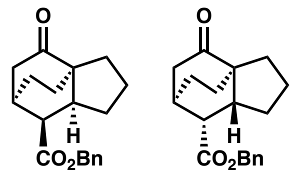

11q (endo) *11q' (exo)*

benzyl (3a*R*,6*R*,7*S*,7a*S*)-4-oxooctahydro-3a,6-ethanoindene-7-carboxylate (11q and 11q')

Prepared from **10q** following General Procedure A. Purification by flash column chromatography (0–35% EtOAc/hexanes) afforded the title compound as a colorless oil (54.9 mg, 0.183 mmol, 92% yield, 1.6:1 endo/exo, 84% ee (endo), 29% ee (exo)). The *endo* (**11q**) and *exo* (**11q'**) diastereomers were subsequently separated by preparative TLC (25% EtOAc/hexanes) for independent characterization. Absolute and relative stereochemistry were assigned/confirmed by VCD (see below).

11q (endo):

^{1}H NMR (400 MHz, $CDCl_3$): δ 7.39 – 7.31 (m, 5H), 5.13 – 5.02 (m, 2H), 3.10 – 2.99 (m, 2H), 2.47 (h, *J* = 2.9 Hz, 1H), 2.36 – 2.21 (m, 2H), 2.15 (dt, *J* = 19.3, 2.3 Hz, 1H), 1.86 – 1.63 (m, 6H), 1.56 – 1.48 (m, 1H), 1.06 – 0.92 (m, 2H).

^{13}C NMR (100 MHz, $CDCl_3$): δ 215.5, 172.9, 135.9, 128.7, 128.7, 128.5, 66.3, 54.4, 45.9, 44.0, 40.2, 31.4, 28.3, 28.1, 27.0, 26.4, 21.8.

IR (Neat Film, NaCl): 2940, 2868, 1728, 1456, 1174, 1166, 1146 cm^{-1}.

HRMS (MM: FD+): *m/z* calc'd for $C_{19}H_{22}O_3$ $[M]^+$: 298.1564, found 298.1578.

Optical Rotation: $[\alpha]_D^{21}$ –32.4 (c 1.00, $CHCl_3$).

SFC conditions: 15% IPA, 2.5 mL/min, Chiralpak AD-H column, λ = 210 nm, t_R (min): minor = 5.44, major = 6.53.

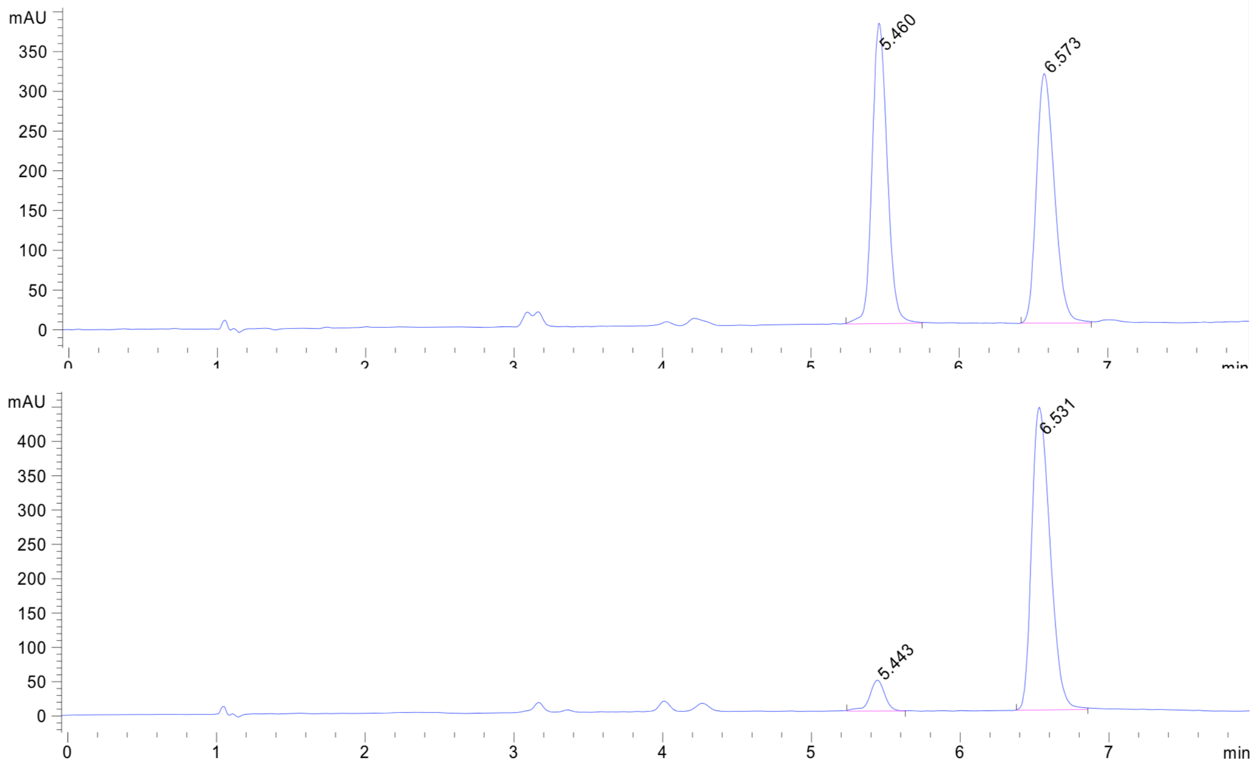

Peak #	RetTime [min]	Type	Width [min]	Area [mAU*s]	Height [mAU]	Area %
1	5.443	BB	0.1088	319.38480	45.02604	7.7563
2	6.531	BB	0.1345	3798.38599	440.86404	92.2437
Totals :				4117.77078	485.89008	

11q' (exo):

^{1}H NMR (400 MHz, CDCl$_3$): δ 7.40 – 7.31 (m, 5H), 5.14 (d, *J* = 12.3 Hz, 1H), 5.09 (d, *J* = 12.2 Hz, 1H), 3.01 (ddd, *J* = 11.7, 3.3, 1.4 Hz, 1H), 2.46 (h, *J* = 3.3 Hz, 1H), 2.35 – 2.25 (m, 3H), 2.14 (tdd, *J* = 11.6, 7.9, 1.6 Hz, 1H), 2.07 – 1.94 (m, 2H), 1.88 – 1.69 (m, 3H), 1.63 – 1.57 (m, 1H), 1.47 – 1.34 (m, 2H), 1.16 (ddd, *J* = 13.9, 9.1, 5.0 Hz, 1H).

^{13}C NMR (100 MHz, CDCl$_3$): δ 215.5, 173.1, 136.0, 128.8, 128.5, 128.5, 66.3, 53.6, 45.4, 42.7, 41.2, 32.0, 26.4, 25.3, 24.4, 22.3, 21.8.

IR (Neat Film, NaCl): 2946, 2847, 1720, 1457, 1154 cm^{-1}.

HRMS (MM: FD+): *m/z* calc'd for $C_{19}H_{22}O_3$ [M]$^+$: 298.1564, found 298.1578.

Optical Rotation: $[\alpha]_D^{21}$ –5.1 (c 1.00, CHCl$_3$).

SFC conditions: 15% IPA, 2.5 mL/min, Chiralpak IC column, λ = 210 nm, t_R (min): minor = 7.01, major = 7.42.

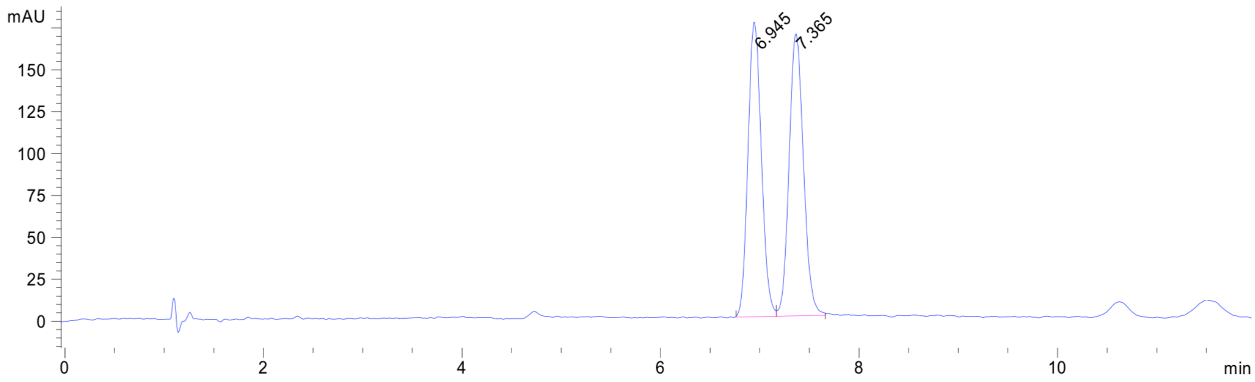

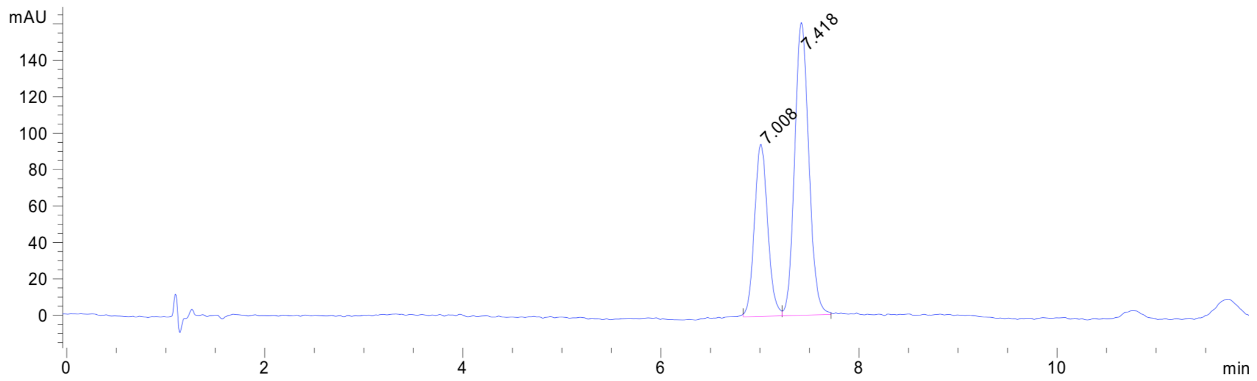

Peak #	RetTime [min]	Type	Width [min]	Area [mAU*s]	Height [mAU]	Area %
1	7.008	BV	0.1445	879.16510	94.55819	35.4023
2	7.418	VB	0.1544	1604.19067	160.74629	64.5977
Totals :				2483.35577	255.30448	

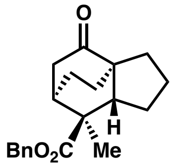

11r

benzyl (3a*R*,6*R*,7*S*,7a*S*)-7-methyl-4-oxooctahydro-3a,6-ethanoindene-7-carboxylate (11r)
Prepared from **10r** following General Procedure A. Purification by flash column chromatography (0–30% EtOAc/hexanes) afforded the title compound as a colorless oil (29.4 mg, 0.094 mmol, 47% yield, 89% ee).

^{1}H NMR (400 MHz, $CDCl_3$): δ 7.39 – 7.29 (m, 5H), 5.16 (d, *J* = 12.4 Hz, 1H), 5.10 (d, *J* = 12.4 Hz, 1H), 2.49 – 2.38 (m, 2H), 2.32 (dt, *J* = 18.9, 2.8 Hz, 1H), 2.19 – 2.10 (m, 2H), 2.01 – 1.92 (m, 1H), 1.85 – 1.66 (m, 4H), 1.65 – 1.55 (m, 1H), 1.49 – 1.31 (m, 5H), 1.13 (ddd, *J* = 14.5, 9.1, 6.0 Hz, 1H).

^{13}C NMR (100 MHz, $CDCl_3$): δ 215.2, 177.8, 136.1, 128.7, 128.4, 128.0, 66.8, 54.37, 45.8, 44.7, 43.4, 37.3, 26.5, 24.4, 24.1, 22.7, 22.4, 20.8.

IR (Neat Film, NaCl): 2951, 2875, 1723, 1454, 1239, 1212, 1106 cm^{-1}.

HRMS (MM: FD+): *m/z* calc'd for $C_{20}H_{24}O_3$ $[M]^+$: 312.1725, found 312.1732.

Optical Rotation: $[\alpha]_D^{21}$ –15.3 (c 1.00, $CHCl_3$).

SFC conditions: 40% IPA, 2.5 mL/min, Chiralpak IC column, λ = 210 nm, t_R (min): minor = 2.68, major = 3.51.

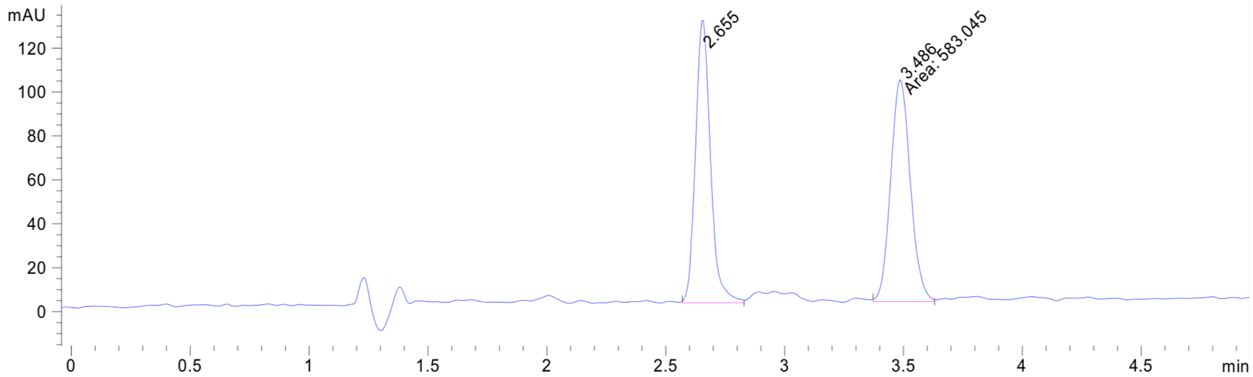

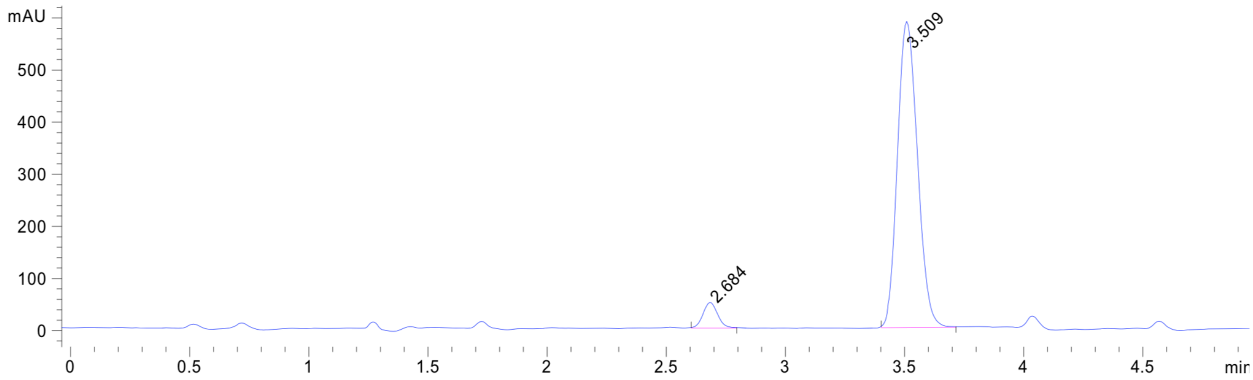

Peak #	RetTime [min]	Type	Width [min]	Area [mAU*s]	Height [mAU]	Area %
1	2.684	BB	0.0658	209.61984	49.13795	5.7437
2	3.509	BB	0.0923	3439.92700	587.48627	94.2563
Totals :				3649.54684	636.62422	

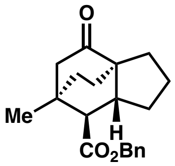

11s

benzyl (3aR,6R,7R,7aR)-6-methyl-4-oxooctahydro-3a,6-ethanoindene-7-carboxylate (11s)

Prepared from **10s** following General Procedure A. Purification by flash column chromatography (0–25% EtOAc/hexanes) afforded the title compound as a colorless oil (43.6 mg, 0.140 mmol, 69% yield, 83% ee).

^{1}H NMR (400 MHz, CDCl$_3$): δ 7.42 – 7.29 (m, 5H), 5.13 (d, *J* = 1.1 Hz, 2H), 2.86 (dd, *J* = 18.5, 3.5 Hz, 1H), 2.37 (dd, *J* = 8.8, 1.4 Hz, 1H), 2.29 – 2.15 (m, 1H), 2.15 – 2.04 (m, 1H), 2.01 – 1.70 (m, 3H), 1.84 (dd, *J* = 18.7, 1.4 Hz, 1H), 1.68 – 1.36 (m, 4H), 1.30 – 1.15 (m, 2H), 0.94 (s, 3H).

^{13}C NMR (100 MHz, CDCl$_3$): δ 214.5, 174.9, 135.9, 128.8, 128.5, 128.5, 66.7, 54.2, 52.6, 47.0, 44.9, 38.0, 36.0, 28.7, 26.3, 26.0, 23.8, 22.8.

IR (Neat Film, NaCl): 2949, 2873, 1750, 1498, 1454, 1384, 1324, 1155, 1114, 977, 754, 698, 678, 556 cm^{-1}.

HRMS (MM: FD+): *m/z* calc'd for $C_{20}H_{24}O_3$ $[M]^+$: 312.1703, found 312.1720.

Optical Rotation: $[\alpha]_D^{21}$ –68.2 (c 0.75, CHCl$_3$).

SFC conditions: 15% IPA, 2.5 mL/min, Chiralpak IC column, λ = 210 nm, t_R (min): minor = 10.23, major = 12.31.

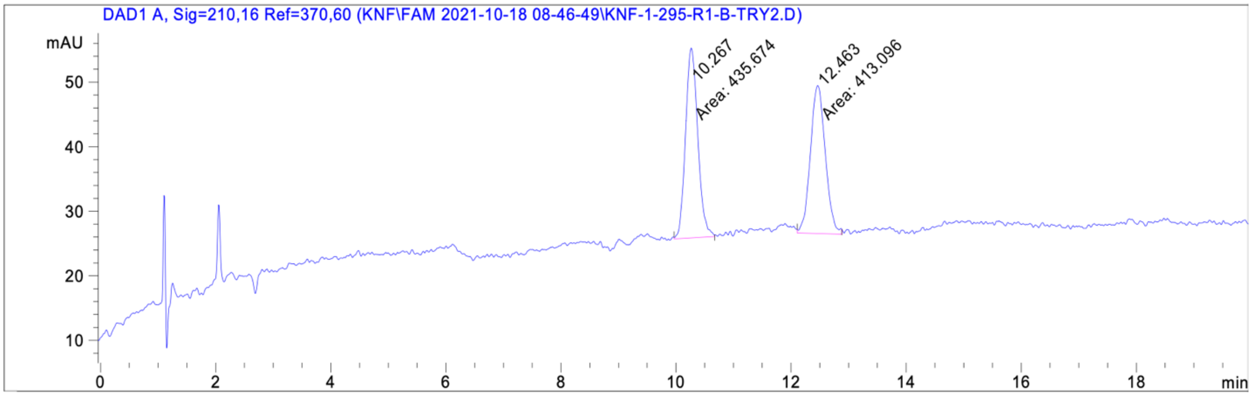

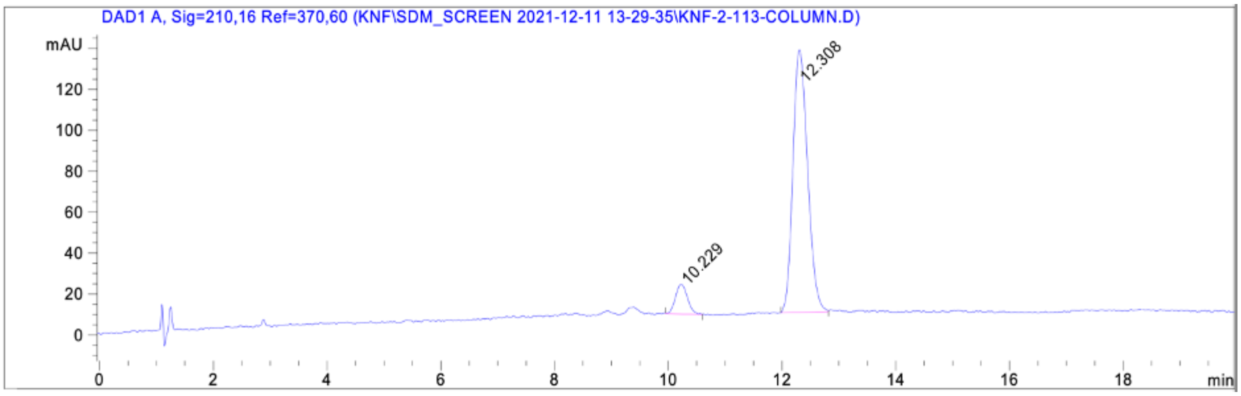

Peak #	RetTime [min]	Type	Width [min]	Area [mAU*s]	Height [mAU]	Area %
1	10.229	BB	0.2208	212.41829	14.50660	8.4608
2	12.308	BB	0.2769	2298.20020	128.32610	91.5392

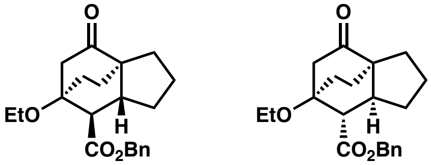

11t (endo) ***11t' (exo)***

benzyl (3a*R*,6*S*,7*R*,7a*R*)-6-ethoxy-4-oxooctahydro-3a,6-ethanoindene-7-carboxylate (11t and 11t')

Prepared from **10t** following General Procedure A. Purification by flash column chromatography (0–50% EtOAc/hexanes) afforded the title compounds as colorless oils (**Endo**: 44.0 mg, 0.128 mmol, 64% yield, 85% ee; **Exo**: 17.0 mg, 0.050 mmol, 25% yield, 72% ee). Absolute and relative stereochemistry were assigned/confirmed by VCD (see below).

11t (endo):

^{1}H NMR (400 MHz, CDCl$_3$): δ 7.38 – 7.28 (m, 5H), 5.16 (d, *J* = 1.5 Hz, 2H), 3.55 – 3.32 (m, 2H), 3.17 (dd, *J* = 18.5, 3.1 Hz, 1H), 2.86 (dd, *J* = 8.5, 1.5 Hz, 1H), 2.38 – 2.25 (m, 2H), 2.16 – 2.02 (m, 1H), 2.02 – 1.86 (m, 3H), 1.87 – 1.39 (m, 5H), 1.20 (ddd, *J* = 14.1, 9.1, 5.1 Hz, 1H), 1.03 (t, *J* = 7.0 Hz, 3H).

^{13}C NMR (100 MHz, CDCl$_3$): δ 210.8, 173.7, 136.0, 128.7, 128.4, 128.3, 78.2, 66.8, 58.0, 54.2, 51.9, 45.1, 45.1, 30.7, 28.8, 25.8, 24.8, 22.9, 15.8.

IR (Neat Film, NaCl): 2944, 2875, 1726, 1458, 1390, 1320, 1282, 1153, 1110, 1039, 746, 700 cm^{-1}.

HRMS (MM: FD+): *m/z* calc'd for $C_{21}H_{26}O_4$ $[M]^+$: 342.1832, found 342.1826.

Optical Rotation: $[\alpha]_D^{21}$ –47.4 (c 0.75, $CHCl_3$).

SFC conditions: 15% IPA, 2.5 mL/min, Chiralpak AD-H column, λ = 210 nm, t_R (min): minor = 4.73, major = 5.13.

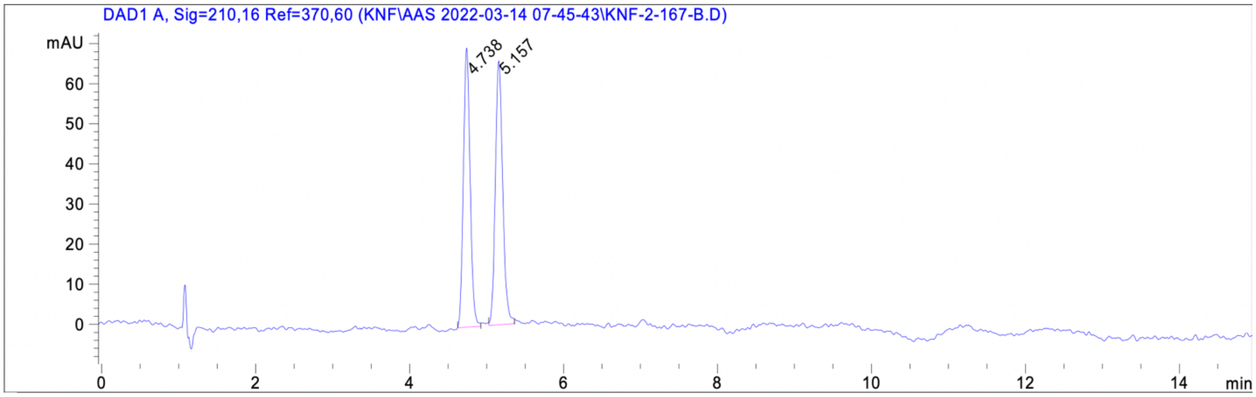

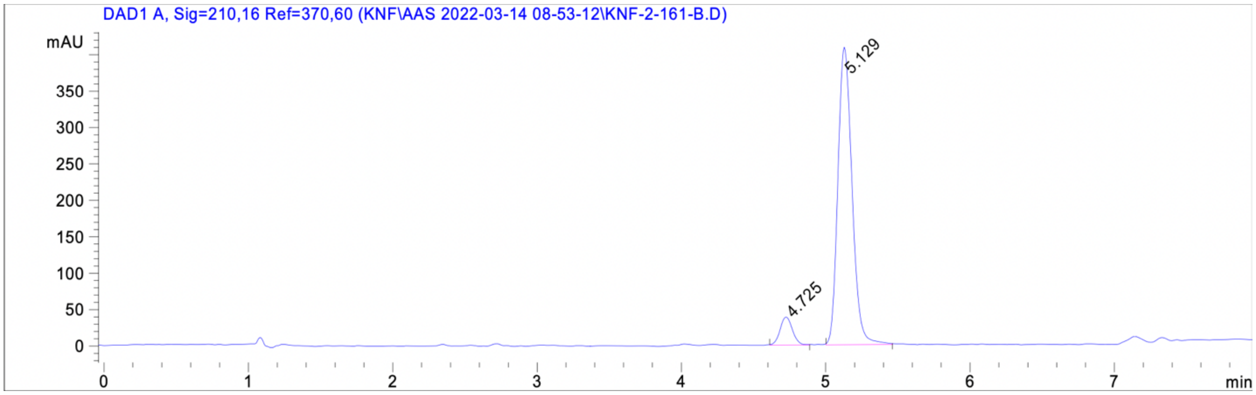

Peak #	RetTime [min]	Type	Width [min]	Area [mAU*s]	Height [mAU]	Area %
1	4.725	BB	0.0913	227.48351	38.31538	7.5556
2	5.129	BB	0.1075	2783.31470	408.39993	92.4444

11t' (exo):

^{1}H NMR (400 MHz, CDCl$_3$): δ 7.42 – 7.28 (m, 5H), 5.19 (d, *J* = 1.1 Hz, 2H), 3.57 – 3.35 (m, 2H), 2.69 (dd, *J* = 7.8, 1.7 Hz, 1H), 2.60 – 2.47 (m, 1H), 2.47 – 2.44 (m, 2H), 2.39 (ddd, *J* = 12.5, 7.9, 6.9 Hz, 1H), 2.28 (ddd, *J* = 13.5, 9.4, 4.6 Hz, 1H), 1.92 – 1.78 (m, 3H), 1.77 – 1.66 (m, 1H), 1.67 – 1.58 (m, 1H), 1.11 (ddd, *J* = 11.3, 6.6, 3.7 Hz, 1H), 1.03 (t, *J* = 6.9 Hz, 3H), 0.89 (tt, *J* = 12.4, 9.6 Hz, 1H).

^{13}C NMR (100 MHz, CDCl$_3$): δ 211.5, 173.4, 136.0, 128.7, 128.4, 128.3, 66.9, 58.1, 54.1, 52.8, 47.5, 46.8, 31,0, 27.7, 26.5, 25.9, 23.1, 15.7.

IR (Neat Film, NaCl): 2946, 1721, 1451, 1390, 1328, 1154, 1117, 767, 698 cm^{-1}.

HRMS (MM: FD+): *m/z* calc'd for $C_{21}H_{26}O_4$ [M]$^+$: 342.1833, found 342.1826.

Optical Rotation: $[\alpha]_D^{21}$ +1.8 (c 0.75, CHCl$_3$).

SFC conditions: 15% IPA, 2.5 mL/min, Chiralpak IC column, λ = 210 nm, t_R (min): minor = 3.29, major = 4.04.

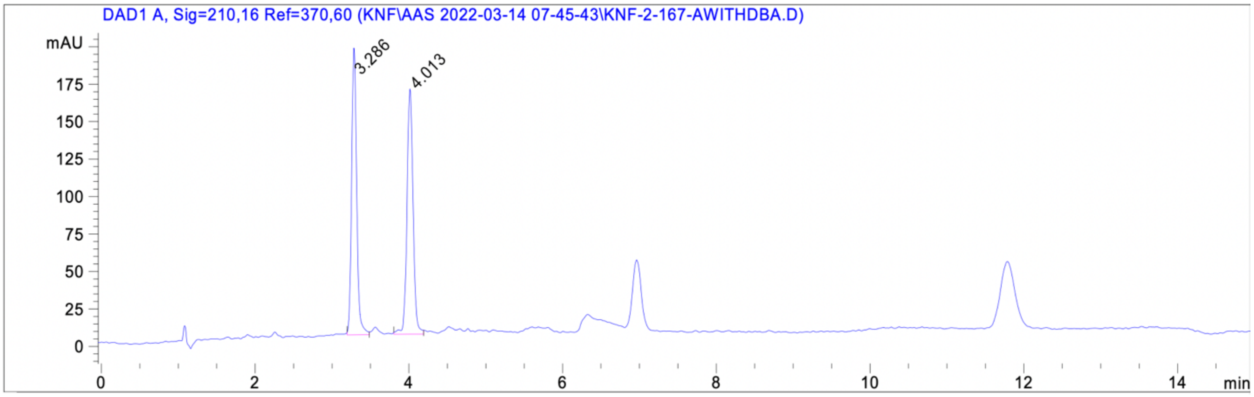

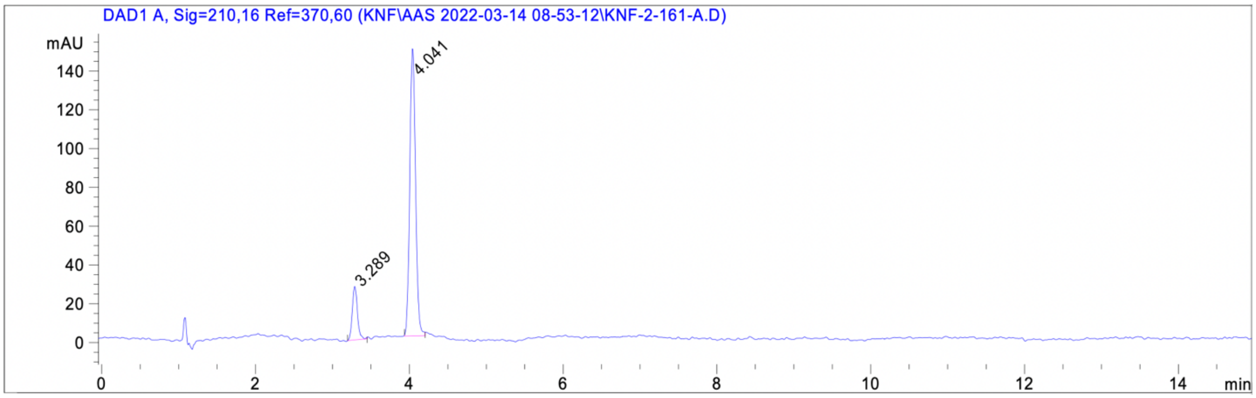

Peak #	RetTime [min]	Type	Width [min]	Area [mAU*s]	Height [mAU]	Area %
1	3.289	BB	0.0724	129.22192	27.70107	14.1656
2	4.041	BB	0.0815	783.00317	148.62152	85.8344

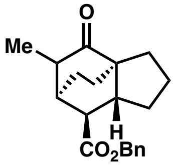

11u

benzyl (3a*R*,6*R*,7*S*,7a*R*)-5-methyl-4-oxooctahydro-3a,6-ethanoindene-7-carboxylate (11u)

Prepared from **10u** following General Procedure A. Purification by flash column chromatography (0–25% EtOAc/hexanes) afforded the title compound as a colorless oil (13.6 mg, 0.044 mmol, 22% yield, 61% ee).

^{1}H NMR (400 MHz, $CDCl_3$): δ 7.39 – 7.32 (m, 5H), 5.14 (dd, *J* = 17.8, 12.2 Hz, 2H), 2.61 (dd, *J* = 3.7, 2.1 Hz, 1H), 2.45 – 2.30 (m, 3H), 2.21 – 2.06 (m, 4H), 1.90 – 1.63 (m, 3H), 1.61 – 1.35 (m, 2H), 1.32 – 1.16 (m, 1H), 0.97 (d, *J* = 7.7 Hz, 3H).

^{13}C NMR (100 MHz, $CDCl_3$): δ 217.7, 174.8, 135.9, 128.7, 128.6, 128.5, 66.8, 54.2, 48.3, 46.9, 42.2, 38.1, 29.7, 28.9, 26.6, 24.1, 22.5, 15.8.

IR (Neat Film, NaCl): 2943, 2873, 1718, 1455, 1197, 1171 cm^{-1}.

HRMS (MM: FD+): *m/z* calc'd for $C_{20}H_{24}O_3$ $[M]^+$: 312.1725, found 312.1730.

Optical Rotation: $[\alpha]_D^{21}$ +10.9 (c 0.75, $CHCl_3$).

SFC conditions: 15% IPA, 2.5 mL/min, Chiralpak AD-H column, λ = 210 nm, t_R (min): minor = 5.84, major = 5.43.

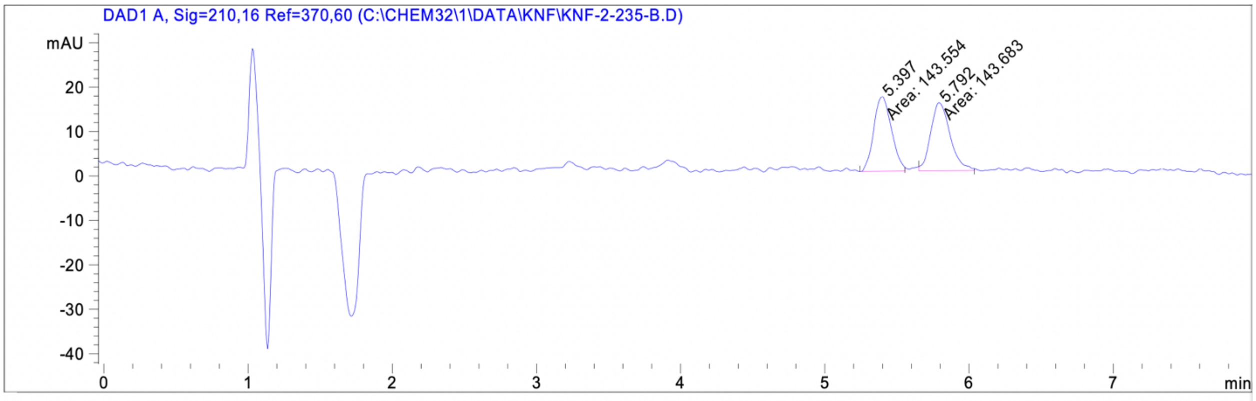

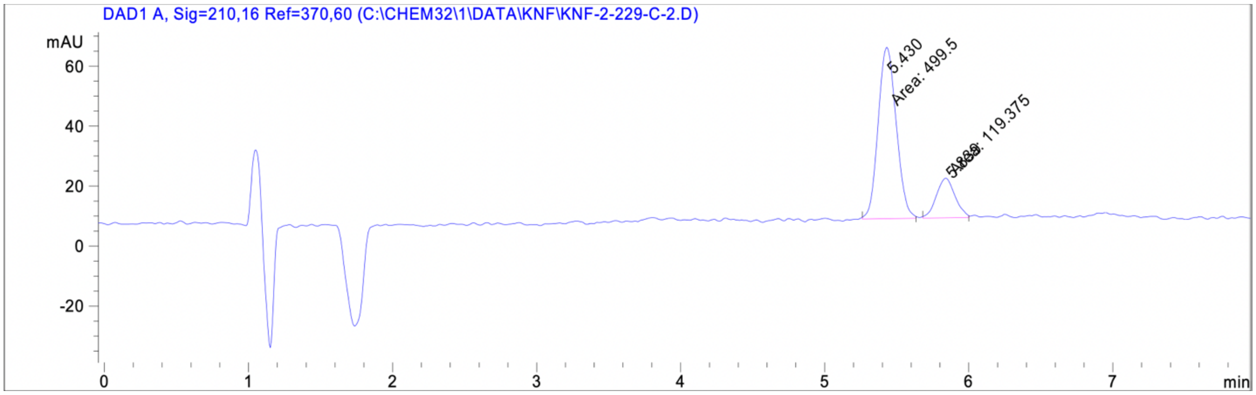

Peak #	RetTime [min]	Type	Width [min]	Area [mAU*s]	Height [mAU]	Area %
1	5.430	MF	0.1453	499.49976	57.29463	80.7110
2	5.839	MF	0.1507	119.37487	13.20177	19.2890

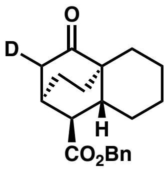

***D*-11f**

benzyl (1*S*,2*R*,4a*R*,8a*R*)-4-oxooctahydro-2*H*-2,4a-ethanonaphthalene-1-carboxylate-3-*d* (*D*-11f)

Prepared from ***D*-10f** following General Procedure A. Purification by flash column chromatography (0–30% EtOAc/hexanes) afforded the title compound as a colorless oil (41.3 mg, 0.132 mmol, 66% yield, 91% ee).

^{1}H NMR (400 MHz, $CDCl_3$): δ 7.39 – 7.30 (m, 5H), 5.16 (d, *J* = 12.4 Hz, 1H), 5.11 (d, *J* = 12.2 Hz, 1H), 2.51 – 2.41 (m, 1.6H), 2.36 – 2.10 (m, 2.7H), 2.02 (dddd, *J* = 11.8, 9.1, 5.3, 2.9 Hz, 1H), 1.91 – 1.83 (m, 1H), 1.82 – 1.71 (m, 1H), 1.70 – 1.55 (m, 4H), 1.52 – 1.11 (m, 5H).

^{13}C NMR (100 MHz, $CDCl_3$): δ 216.4, 174.5, 136.0, 128.8, 128.4, 128.2, 77.5, 77.2, 76.8, 66.7, 49.9, 45.1, 40.5, 37.1, 31.0, 30.9, 30.8, 30.0, 28.9, 26.2, 25.6, 21.8, 21.2.
**Partial deuteration complicates ^{13}C NMR spectrum. Peaks are listed as they appear.*

^{2}H NMR (61 MHz, $CHCl_3$): δ 2.46, 2.14.
**Trace D-exchanged water observed in spectrum.*

IR (Neat Film, NaCl): 2928, 2858, 1723, 1169 cm^{-1}.

HRMS (MM: FD+): *m/z* calc'd for $C_{20}H_{23}DO_3$ $[M+H]^+$: 313.1783, found 313.1795.

Optical Rotation: $[\alpha]_D^{21}$ –21.3 (c 1.00, $CHCl_3$).

SFC conditions: 15% IPA, 2.5 mL/min, Chiralpak AD-H column, λ = 210 nm, t_R (min): minor = 5.07, major = 6.38.

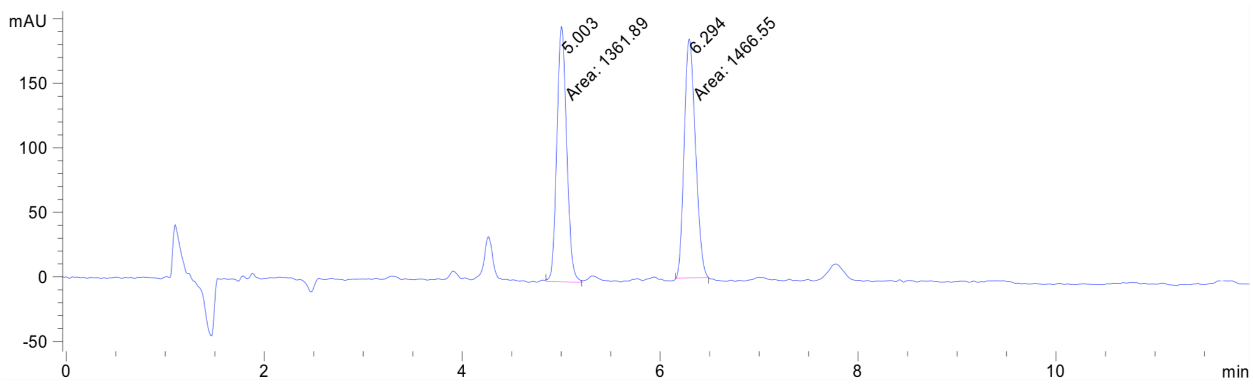

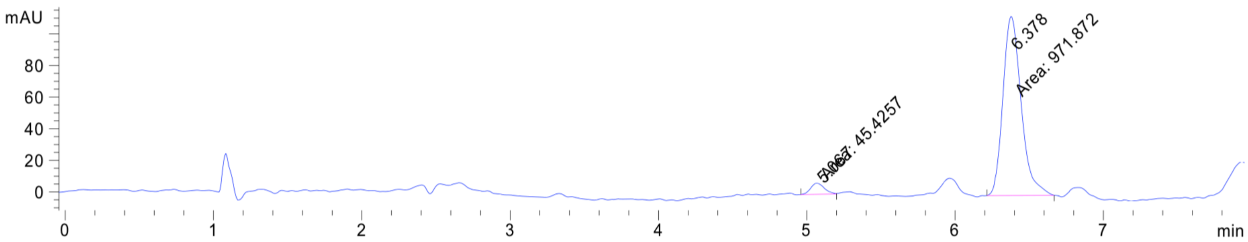

Peak #	RetTime [min]	Type	Width [min]	Area [mAU*s]	Height [mAU]	Area %
1	5.067	MM	0.1068	45.42569	7.09195	4.4653
2	6.378	MM	0.1429	971.87183	113.34013	95.5347

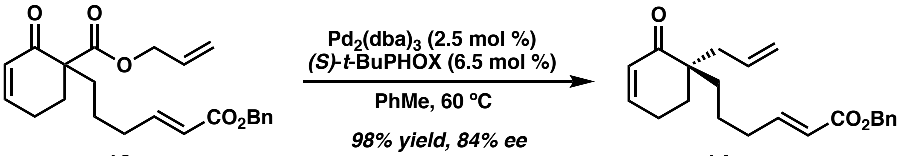

12 **14**

benzyl (*S*,*E*)-6-(1-allyl-2-oxocyclohex-3-en-1-yl)hex-2-enoate (14)

Prepared from **12** (0.02 mmol) following General Procedure A. Purification by preparatory thin layer chromatography (25% EtOAc/hexanes) afforded the title compound as a colorless oil (10.1 mg, 0.0196 mmol, 98% yield, 84 % ee).

^{1}H NMR (400 MHz, CDCl$_3$): δ 7.40 – 7.29 (m, 5H), 6.97 (dt, *J* = 15.6, 6.9 Hz, 1H), 6.85 (dt, *J* = 10.1, 3.9 Hz, 1H), 5.91 (dt, *J* = 10.0, 2.0 Hz, 1H), 5.85 (dt, *J* = 15.7, 1.6 Hz, 1H), 5.69 (ddt, *J* = 16.6, 10.5, 7.3 Hz, 1H), 5.17 (s, 2H), 5.09 – 5.00 (m, 2H), 2.41 – 2.29 (m, 3H), 2.26 – 2.13 (m, 3H), 1.87 (t, *J* = 6.1 Hz, 2H), 1.63 – 1.23 (m, 4H).

^{13}C NMR (100 MHz, CDCl$_3$): δ 202.9, 166.6, 149.6, 148.7, 136.3, 134.0, 129.0, 128.7, 128.3, 128.3, 121.4, 118.3, 66.2, 47.6, 39.1, 33.9, 32.9, 30.8, 23.1, 22.4.

IR (Neat Film, NaCl): 2936, 2358, 1718, 1669, 1262, 992 cm^{-1}.

HRMS (MM: FD+): *m/z* calc'd for $C_{22}H_{26}O_3$ $[M]^+$: 338.1881, found 338.1877.

Optical Rotation: $[\alpha]_D^{21}$ –0.69 (c 0.62, CHCl$_3$).

SFC conditions: 15% IPA, 2.5 mL/min, Chiralpak IC column, λ = 210 nm, t_R (min): minor = 14.49, major = 11.94.

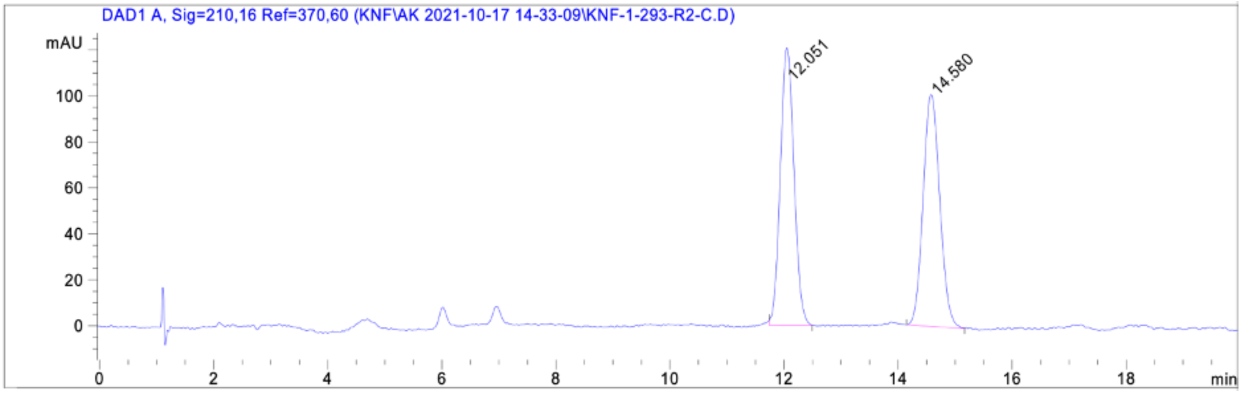

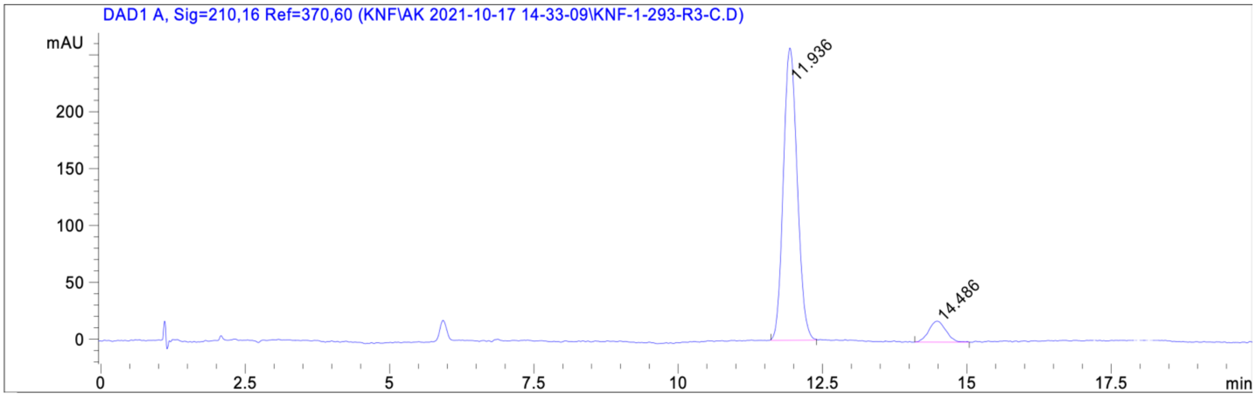

Peak #	RetTime [min]	Type	Width [min]	Area [mAU*s]	Height [mAU]	Area %
1	11.936	BB	0.2606	4289.03320	257.13303	91.9192
2	14.486	BB	0.3166	377.05917	18.28423	8.0808

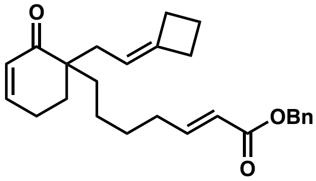

S5

benzyl (*E*)-7-(1-(2-cyclobutylideneethyl)-2-oxocyclohex-3-en-1-yl)hept-2-enoateenoate (S5)

Prepared from **33** following General Procedure A, with the modification of being on 0.1 mmol scale. Purification by preparatory thin layer chromatography (20% EtOAc/hexanes) afforded the title compound as a clear oil (2.4 mg, 0.006 mmol, 6% yield).

^{1}H NMR (400 MHz, $CDCl_3$): δ 7.41 – 7.29 (m, 5H), 6.99 (dt, *J* = 15.6, 6.9 Hz, 1H), 6.83 (dt, *J* = 10.0, 3.9 Hz, 1H), 5.92 – 5.81 (m, 2H), 5.17 (s, 2H), 5.01 – 4.92 (m, 1H), 2.67 – 2.54 (m, 3H), 2.40 – 2.30 (m, 2H), 2.27 – 1.99 (m, 4H), 1.96 – 1.82 (m, 3H), 1.63 – 1.49 (m, 3H), 1.49 – 1.36 (m, 3H), 1.32 – 1.13 (m, 2H).

^{13}C NMR (100 MHz, $CDCl_3$): δ 203.6, 166.6, 150.1, 148.5, 143.1, 136.3, 129.1, 128.7, 128.3, 128.3, 121.1, 115.4, 66.2, 48.3, 34.1, 33.1, 32.3, 31.2, 30.8, 29.6, 28.8, 23.6, 23.2, 17.1.

IR (Neat Film, NaCl): 2929, 1720, 1670, 1185 cm^{-1}.

HRMS (MM: FD+): *m/z* calc'd for $C_{26}H_{32}O_3$ $[M]^+$: 392.2351, found 392.2341.

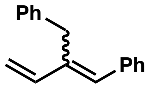

S6

(2-vinylprop-1-ene-1,3-diyl)dibenzene (S6)

Prepared from **37** following General Procedure A, with the modification of being on 0.1 mmol scale. Purification by flash column chromatography (0–35% EtOAc/hexanes) afforded the title compound as a colorless oil (6.5 mg, 0.03 mmol, 29% yield).

^{1}H NMR (400 MHz, $CDCl_3$): δ 7.39 – 7.18 (m, 11.5H), 6.91 – 6.80 (m, 1.15H), 6.56 (ddd, *J* = 17.4, 10.8, 0.9 Hz, 0.15H), 6.45 (s, 1H), 5.42 – 5.33 (m, 1H), 5.19 – 5.13 (m, 1.15H), 5.11 – 5.06 (m, 0.15H), 3.90 (s, 0.3H), 3.73 (s, 2H).

**Isolated as an apparent 1:0.15 mixture of alkene isomers.*

^{13}C NMR (100 MHz, $CDCl_3$): δ 140.5, 140.2, 139.9, 137.7, 137.5, 137.3, 134.2, 133.8, 131.8, 129.6, 128.9, 128.8, 128.7, 128.5, 128.2, 128.2, 127.3, 127.0, 126.2, 126.1, 116.4, 114.8, 40.3, 33.2.

**Isolated as an apparent 1:0.15 mixture of alkene isomers.*

IR (Neat Film, NaCl): 3060, 3023, 2919, 1601, 1493, 1455, 1165, 1074 cm^{-1}.

HRMS (MM: FD+): *m/z* calc'd for $C_{17}H_{16}$ $[M]^+$: 220.1252, found 220.1257.

Preparation of Unsaturated β-Ketoester Starting Materials

General Procedure B: Horner–Wadsworth–Emmons Olefination

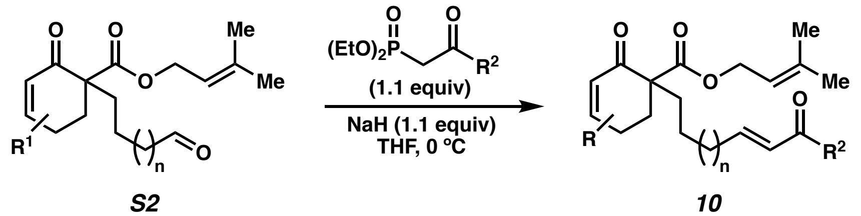

To a suspension of NaH (60% by weight in mineral oil, 1.1 equiv) in THF (0.5 M) at 0 °C was dropwise added a solution of the appropriate phosphonate ester (1.1 equiv) in THF (1.0 M). Stirred at 0 °C was continued for 30 minutes. To the reaction was then dropwise added a solution of aldehyde **S2** (1.0 equiv) in THF (0.5 M). Upon complete consumption of starting material (as determined by TLC), the reaction mixture was diluted with a saturated solution of $NaHCO_3$ and extracted with EtOAc (3x). The combined organic layers were dried over Na_2SO_4, filtered, and concentrated under reduced pressure. The product (**10**) was purified by silica gel flash column chromatography.

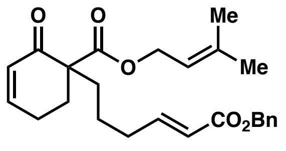

10a

3-methylbut-2-en-1-yl (*E*)-1-(6-(benzyloxy)-6-oxohex-4-en-1-yl)-2-oxocyclohex-3-ene-1-carboxylate (10a)

Prepared from **S2a** and benzyl 2-(diethoxyphosphoryl)acetate[3] following General Procedure B. Purification by flash column chromatography (20% EtOAc/hexanes) afforded the title compound as a colorless oil (0.410 g, 1.00 mmol, 67 % yield).

^{1}H NMR (400 MHz, CDCl$_3$): δ 7.38 – 7.29 (m, 5H), 6.98 (dt, *J* = 15.7, 6.9 Hz, 1H), 6.90 – 6.84 (m, 1H), 6.02 (ddd, *J* = 10.1, 2.5, 1.5 Hz, 1H), 5.87 (dt, *J* = 15.6, 1.6 Hz, 1H), 5.31 – 5.24 (m, 1H), 5.17 (s, 2H), 4.58 (dt, *J* = 7.2, 1.0 Hz, 2H), 2.55 – 2.41 (m, 2H), 2.37 – 2.27 (m, 1H), 2.22 (qd, *J* = 7.3, 1.6 Hz, 2H), 1.97 – 1.86 (m, 2H), 1.77 – 1.69 (m, 4H), 1.67 (d, *J* = 1.4 Hz, 3H), 1.55 – 1.38 (m, 2H).

^{13}C NMR (100 MHz, $CDCl_3$): δ 196.3, 171.6, 166.5, 149.3, 149.3, 139.7, 136.3, 129.4, 128.7, 128.3, 128.3, 121.5, 118.3, 66.2, 62.3, 57.0, 33.5, 32.7, 30.5, 25.8, 23.9, 23.2, 18.2.

IR (Neat Film, NaCl): 3034, 2938, 1723, 1684, 1653, 1455, 1384, 1246, 1174, 1166 cm^{-1}.

HRMS (MM: FD+): *m/z* calc'd for $C_{25}H_{30}O_5$ $[M]^+$: 410.2088, found 410.2097.

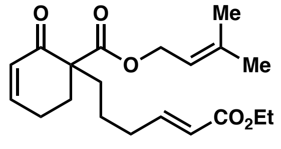

10b

3-methylbut-2-en-1-yl (*E*)-1-(6-(benzyloxy)-6-oxohex-4-en-1-yl)-2-oxocyclohex-3-ene-1-carboxylate (10b)

Prepared from **S2a** and ethyl 2-(diethoxyphosphoryl)acetate following General Procedure B. Purification by flash column chromatography (20% EtOAc/hexanes) afforded the title compound as a colorless oil (0.291 g, 0.835 mmol, 47 % yield).

^{1}H NMR (400 MHz, $CDCl_3$): δ 6.97 – 6.85 (m, 2H), 6.02 (ddd, *J* = 10.0, 2.6, 1.5 Hz, 1H), 5.81 (dt, *J* = 15.6, 1.6 Hz, 1H), 5.31 – 5.24 (m, 1H), 4.64 – 4.54 (m, 2H), 4.18 (q, *J* = 7.1 Hz, 2H), 2.55 – 2.41 (m, 2H), 2.37 – 2.27 (m, 1H), 2.21 (qd, *J* = 7.3, 1.6 Hz, 2H), 1.98 – 1.87 (m, 2H), 1.78 – 1.69 (m, 4H), 1.68 (s, 3H), 1.55 – 1.37 (m, 2H), 1.28 (t, *J* = 7.1 Hz, 3H).

^{13}C NMR (100 MHz, $CDCl_3$): δ 196.3, 171.6, 166.8, 149.3, 148.5, 139.7, 129.4, 121.8, 118.3, 62.3, 60.3, 57.0, 33.5, 32.6, 30.5, 25.8, 23.9, 23.3, 18.2, 14.4.

IR (Neat Film, NaCl): 2934, 1714, 1682, 1168 cm^{-1}.

HRMS (MM: FD+): *m/z* calc'd for $C_{20}H_{28}O_5$ $[M]^+$: 348.1937, found 348.1943.

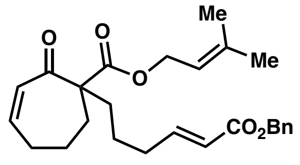

10d

3-methylbut-2-en-1-yl (*E*)-1-(6-(benzyloxy)-6-oxohex-4-en-1-yl)-2-oxocyclohept-3-ene-1-carboxylate (10d)

Prepared from cyclohept-2-en-1-one following General Procedures B–D. Note that inseparable impurities plagued the β-ketoester and aldehyde intermediates. Fortunately, these intermediates could be brought through the sequence in sub-optimal purity to still afford the title compound **10d** as a colorless oil (265 mg, 0.62 mmol, 3.2% yield from cyclohept-2-en-1-one) after a final purification by flash column chromatography (20% EtOAc/hexanes).

^{1}H NMR (400 MHz, CDCl$_3$): δ 7.39 – 7.30 (m, 5H), 6.97 (dt, *J* = 15.6, 6.9 Hz, 1H), 6.36 (ddd, *J* = 12.3, 5.5, 3.9 Hz, 1H), 5.98 (ddd, *J* = 12.3, 2.4, 1.4 Hz, 1H), 5.86 (dt, *J* = 15.6, 1.6 Hz, 1H), 5.28 (ddp, *J* = 8.6, 5.7, 1.4 Hz, 1H), 5.17 (s, 2H), 4.57 (d, *J* = 7.2 Hz, 2H), 2.47 – 2.27 (m, 3H), 2.19 (qd, *J* = 7.2, 1.6 Hz, 2H), 1.99 – 1.79 (m, 3H), 1.77 – 1.69 (m, 4H), 1.69 – 1.61 (m, 4H), 1.43 – 1.32 (m, 2H).

^{13}C NMR (100 MHz, CDCl$_3$): δ 201.1, 172.9, 166.5, 149.3, 143.2, 139.7, 136.3, 131.6, 128.7, 128.3, 128.3, 121.5, 118.2, 66.2, 63.9, 62.2, 36.5, 32.6, 32.3, 31.2, 25.8, 24.3, 23.0, 18.2.

IR (Neat Film, NaCl): 2927, 1720, 1686, 1453, 1162 cm^{-1}.

HRMS (MM: FD+): *m/z* calc'd for $C_{26}H_{32}O_5$ $[M]^+$: 424.2244, found 424.2241.

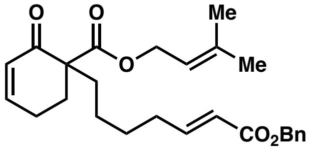

10f

3-methylbut-2-en-1-yl (*E*)-1-(7-(benzyloxy)-7-oxohept-5-en-1-yl)-2-oxocyclohex-3-ene-1-carboxylate (10f)

Prepared from **S2b** and benzyl 2-(diethoxyphosphoryl)acetate[3] following General Procedure B. Purification by flash column chromatography (15–20% EtOAc/hexanes) afforded the title compound as a colorless oil (652 mg, 1.54 mmol, 69% yield).

^{1}H NMR (400 MHz, CDCl$_3$): δ 7.39 – 7.30 (m, 5H), 6.99 (ddd, *J* = 15.5, 7.3, 6.3 Hz, 1H), 6.90 – 6.84 (m, 1H), 6.01 (d, *J* = 10.1 Hz, 1H), 5.85 (d, *J* = 15.6 Hz, 1H), 5.30 – 5.25 (m, 1H), 5.17 (s, 2H), 4.58 (d, *J* = 6.6 Hz, 2H), 2.55 – 2.40 (m, 2H), 2.36 – 2.26 (m, 1H), 2.24 – 2.16 (m, 2H), 1.96 – 1.86 (m, 2H), 1.77 – 1.65 (m, 7H), 1.47 (p, *J* = 7.4 Hz, 2H), 1.38 – 1.24 (m, 2H).

^{13}C NMR (100 MHz, CDCl$_3$): δ 196.2, 171.6, 166.5, 149.7, 149.2, 139.4, 136.2, 129.2, 128.6, 128.2, 128.2, 121.1, 118.2, 66.0, 62.1, 56.9, 33.5, 32.0, 30.2, 28.4, 25.7, 24.2, 23.7, 18.1.

IR (Neat Film, NaCl): 2932, 2861, 1722, 1684, 1653, 1456, 1263, 1181 cm^{-1}.

HRMS (MM: FD+): *m/z* calc'd for $C_{26}H_{32}O_5$ $[M]^+$: 424.2244, found 424.2247.

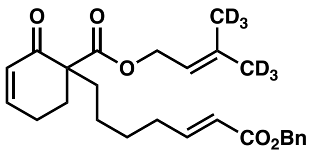

***D*-10f**

3-(methyl-*d*$_3$)but-2-en-1-yl-4,4,4-*d*$_3$ (*E*)-1-(7-(benzyloxy)-7-oxohept-5-en-1-yl)-2-oxocyclohex-3-ene-1-carboxylate (*D*-10f)

Prepared from ***D*-S2b** and benzyl 2-(diethoxyphosphoryl)acetate[3] following General Procedure B. Purification by flash column chromatography (15–20% EtOAc/hexanes) afforded the title compound as a colorless oil (201 mg, 0.467 mmol, 48 % yield).

^{1}H NMR (400 MHz, CDCl$_3$): δ 7.39 – 7.30 (m, 5H), 6.99 (dt, *J* = 15.6, 6.9 Hz, 1H), 6.90 – 6.85 (m, 1H), 6.01 (ddd, *J* = 10.1, 2.6, 1.5 Hz, 1H), 5.85 (dt, *J* = 15.6, 1.5 Hz, 1H), 5.27 (t, *J* = 7.1 Hz, 1H), 5.17 (s, 2H), 4.58 (dd, *J* = 7.2, 1.7 Hz, 2H), 2.55 – 2.41 (m, 2H), 2.36 – 2.27 (m, 1H), 2.25 – 2.15 (m, 2H), 1.98 – 1.85 (m, 2H), 1.73 (ddd, *J* = 13.6, 11.2, 5.3 Hz, 1H), 1.51 – 1.42 (m, 2H), 1.37 – 1.25 (m, 2H).

^{13}C NMR (100 MHz, CDCl$_3$): δ 196.4, 171.7, 166.6, 149.8, 149.3, 139.4, 136.3, 129.4, 128.7, 128.3, 128.3, 121.2, 118.4, 66.2, 62.3, 57.0, 33.6, 32.2, 30.3, 28.5, 24.3, 23.9.

^{2}H NMR (61 MHz, CHCl$_3$): δ 1.69, 1.65.

IR (Neat Film, NaCl): 2930, 1720, 1683, 1264, 1167 cm^{-1}.

HRMS (MM: FD+): *m/z* calc'd for $C_{26}H_{26}D_6O_5$ $[M]^+$: 430.2621, found 430.2622.

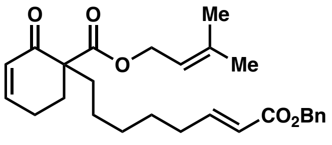

10g

3-methylbut-2-en-1-yl (*E*)-1-(8-(benzyloxy)-8-oxooct-6-en-1-yl)-2-oxocyclohex-3-ene-1-carboxylate (10g)

Prepared from **S2c** and benzyl 2-(diethoxyphosphoryl)acetate[3] following General Procedure B. Purification by flash column chromatography (10–25% EtOAc/hexanes) afforded the title compound as a colorless oil (773 mg, 1.76 mmol, 65 % yield).

^{1}H NMR (400 MHz, CDCl$_3$): δ 7.40 – 7.29 (m, 5H), 6.99 (dt, J = 15.6, 6.9 Hz, 1H), 6.89 – 6.84 (m, 1H), 6.01 (ddd, J = 10.0, 2.5, 1.5 Hz, 1H), 5.85 (dt, J = 15.6, 1.6 Hz, 1H), 5.28 (ddp, J = 8.6, 5.7, 1.4 Hz, 1H), 5.17 (s, 2H), 4.63 – 4.53 (m, 2H), 2.55 – 2.40 (m, 2H), 2.36 – 2.26 (m, 1H), 2.19 (qd, J = 7.1, 1.6 Hz, 2H), 1.98 – 1.84 (m, 2H), 1.77 – 1.69 (m, 4H), 1.67 (s, 3H), 1.49 – 1.41 (m, 2H), 1.36 – 1.23 (m, 4H).

^{13}C NMR (100 MHz, CDCl$_3$): δ 196.5, 171.7, 166.6, 150.1, 149.3, 139.5, 136.3, 129.4, 128.7, 128.3, 128.3, 121.2, 118.4, 66.2, 62.2, 57.1, 33.7, 32.3, 30.3, 29.6, 27.8, 25.8, 24.4, 23.9, 18.2.

IR (Neat Film, NaCl): 2929, 2858, 1721, 1684, 1654, 1456, 1264, 1168 cm^{-1}.

HRMS (MM: FD+): *m/z* calc'd for $C_{27}H_{34}O_5$ $[M]^+$: 438.2401, found 438.2396.

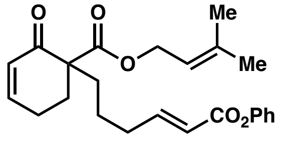

10k

3-methylbut-2-en-1-yl (*E*)-2-oxo-1-(6-oxo-6-phenoxyhex-4-en-1-yl)cyclohex-3-ene-1-carboxylate (10k)

Prepared from **S2a** and benzyl 2-(diethoxyphosphoryl)acetate[4] following General Procedure B. Purification by flash column chromatography (20% EtOAc/hexanes) afforded the title compound as a colorless oil (409 mg, 1.03 mmol, 57 % yield).

^{1}H NMR (400 MHz, $CDCl_3$): δ 7.38 (t, *J* = 8.0 Hz, 2H), 7.22 (t, *J* = 7.4 Hz, 1H), 7.18 – 7.10 (m, 3H), 6.91 – 6.87 (m, 1H), 6.05 – 6.00 (m, 2H), 5.29 (tt, *J* = 7.1, 1.3 Hz, 1H), 4.61 (d, *J* = 7.1 Hz, 2H), 2.56 – 2.44 (m, 2H), 2.37 – 2.27 (m, 3H), 2.00 – 1.92 (m, 2H), 1.82 – 1.73 (m, 4H), 1.69 (s, 3H), 1.63 – 1.46 (m, 2H).

^{13}C NMR (100 MHz, $CDCl_3$): δ 196.3, 171.6, 165.1, 150.9, 150.9, 149.3, 139.7, 129.5, 129.4, 125.8, 121.8, 121.1, 118.3, 62.4, 57.0, 33.6, 32.8, 30.5, 25.8, 23.9, 23.2, 18.2.

IR (Neat Film, NaCl): 2930, 1732, 1684, 1652, 1458, 1245, 1195 cm^{-1}.

HRMS (MM: FD+): *m/z* calc'd for $C_{24}H_{28}O_5$ $[M]^+$: 396.1931, found 396.1945.

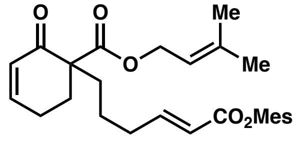

10l

3-methylbut-2-en-1-yl (*E*)-1-(6-(mesityloxy)-6-oxohex-4-en-1-yl)-2-oxocyclohex-3-ene-1-carboxylate (10l)

Prepared from **S2a** and mesityl 2-(diethoxyphosphoryl)acetate[5] following General Procedure B. Purification by flash column chromatography (20% EtOAc/hexanes) afforded the title compound as a colorless oil (597 mg, 1.36 mmol, 76% yield).

^{1}H NMR (400 MHz, CDCl$_3$): δ 7.17 (dt, *J* = 15.7, 6.8 Hz, 1H), 6.92 – 6.84 (m, 3H), 6.09 – 6.01 (m, 2H), 5.32 – 5.26 (m, 1H), 4.61 (d, *J* = 5.6 Hz, 2H), 2.57 – 2.43 (m, 2H), 2.39 – 2.27 (m, 3H), 2.26 (s, 3H), 2.09 (s, 6H), 2.01 – 1.92 (m, 2H), 1.79 (ddd, *J* = 13.6, 11.9, 4.8 Hz, 1H), 1.73 (s, 3H), 1.69 (s, 3H), 1.64 – 1.46 (m, 2H).

^{13}C NMR (100 MHz, CDCl$_3$): δ 196.3, 171.6, 164.6, 150.7, 149.3, 146.0, 139.7, 135.3, 130.0, 129.4, 129.3, 120.7, 118.3, 62.4, 57.0, 33.6, 32.8, 30.5, 25.8, 23.9, 23.2, 20.9, 18.2, 16.4.

IR (Neat Film, NaCl: 2920, 1733, 1684, 1458, 1248, 1192, 1140 cm^{-1}.

HRMS (MM: FD+): *m/z* calc'd for $C_{27}H_{34}O_5$ [M]$^+$: 438.2401, found 438.2401.

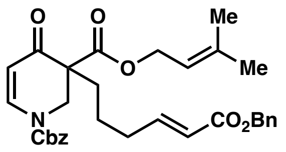

10n

1-benzyl 3-(3-methylbut-2-en-1-yl) (*E*)-3-(6-(benzyloxy)-6-oxohex-4-en-1-yl)-4-oxo-3,4-dihydropyridine-1,3(2*H*)-dicarboxylate (10n)

Prepared from **S2e** and benzyl 2-(diethoxyphosphoryl)acetate[3] following General Procedure B. Purification by flash column chromatography (20-30% EtOAc/hexanes) afforded the title compound as a colorless oil (181.3 mg, 0.332 mmol, 55% yield).

^{1}H NMR (400 MHz, CDCl$_3$): δ 7.79 (s, 1H), 7.44 – 7.28 (m, 10H), 6.94 (dt, *J* = 15.5, 6.8 Hz, 1H), 5.85 (dt, *J* = 15.7, 1.6 Hz, 1H), 5.42 – 5.21 (m, 4H), 5.17 (s, 2H), 4.67 – 4.51 (m, 3H), 3.71 (d, *J* = 13.6 Hz, 1H), 2.20 (q, *J* = 7.3 Hz, 2H), 2.07 – 1.92 (m, 1H), 1.69 – 1.58 (m, 1H), 1.71 (s, 3H), 1.66 (s, 3H), 1.49 – 1.40 (m, 2H).

^{13}C NMR (100 MHz, CDCl$_3$): δ 190.5, 169.5, 166.4, 148.7, 142.7, 140.1, 136.2, 135.0, 129.0, 128.9, 128.7, 128.6, 128.4, 128.3, 121.7, 118.0, 106.5, 69.4, 66.2, 62.8, 55.3, 48.3, 32.4, 31.3, 25.8, 23.0, 18.2.

IR (Neat Film, NaCl): 2938, 2338, 1726, 1676, 1604, 1456, 1388, 1303, 1201, 975 cm^{-1}.

HRMS (MM: FD+): *m/z* calc'd for $C_{32}H_{35}NO_7$ $[M]^+$: 545.2414, found 545.2408.

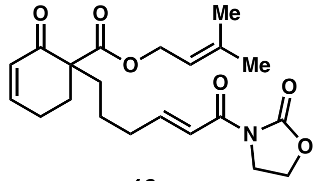

10o

3-methylbut-2-en-1-yl (*E*)-2-oxo-1-(6-oxo-6-(2-oxooxazolidin-3-yl)hex-4-en-1-yl)cyclohex-3-ene-1-carboxylate (10o)

Prepared from **S2a** and diethyl (2-oxo-2-(2-oxooxazolidin-3-yl)ethyl)phosphonate[6] following General Procedure B. Purification by flash column chromatography (0-80% EtOAc/hexanes) afforded the title compound as a colorless oil (275.1 mg, 0.706 mmol, 71% yield).

^{1}H NMR (400 MHz, CDCl$_3$): δ 7.23 (d, *J* = 15.6 Hz, 1H), 7.12 (dt, *J* = 15.4, 6.7 Hz, 1H), 6.87 (ddd, *J* = 7.8, 6.0, 3.9 Hz, 1H), 6.01 (dt, *J* = 10.3, 2.0 Hz, 1H), 5.28 (tt, *J* = 7.2, 1.3 Hz, 1H), 4.62 – 4.54 (m, 2H), 4.41 (dd, *J* = 8.5, 7.6 Hz, 2H), 4.11 – 4.02 (m, 2H), 2.55 – 2.40 (m, 2H), 2.38 – 2.25 (m, 3H), 2.00 – 1.86 (m, 2H), 1.82 – 1.69 (m, 2H), 1.73 (s, 3H), 1.68 (s, 3H), 1.50 (dtd, *J* = 17.3, 12.4, 7.6 Hz, 2H).

^{13}C NMR (100 MHz, CDCl$_3$): δ 196.3, 171.6, 165.3, 153.6, 150.9, 149.4, 139.6, 129.3, 120.5, 118.3, 62.3, 62.2, 57.0, 42.8, 33.4, 33.1, 30.3, 25.8, 23.9, 23.3, 18.2.

IR (Neat Film, NaCl): 2927, 1774, 1724, 1684, 1636, 1385, 1359, 1222, 1042 cm^{-1}.

HRMS (MM: FD+): *m/z* calc'd for $C_{21}H_{27}NO_6$ $[M]^+$: 389.1838, found 389.1827.

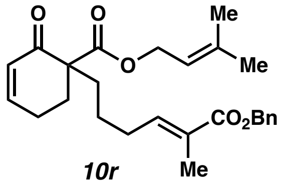

10r

3-methylbut-2-en-1-yl (*E*)-1-(6-(benzyloxy)-5-methyl-6-oxohex-4-en-1-yl)-2-oxocyclohex-3-ene-1-carboxylate (10r)

Prepared from **S2a** and benzyl 2-(diethoxyphosphoryl)propanoate[7] following General Procedure B. Purification by flash column chromatography (0–30% EtOAc/hexanes) afforded the title compound as a colorless oil (220 mg, 0.518 mmol, 29% yield).

^{1}H NMR (400 MHz, $CDCl_3$): δ 7.39 – 7.29 (m, 5H), 6.87 (dddd, *J* = 10.1, 4.9, 3.0, 1.1 Hz, 1H), 6.78 (tq, *J* = 7.5, 1.5 Hz, 1H), 6.01 (ddd, *J* = 10.1, 2.5, 1.5 Hz, 1H), 5.31 – 5.24 (m, 1H), 5.18 (s, 2H), 4.58 (d, *J* = 6.9 Hz, 2H), 2.55 – 2.41 (m, 2H), 2.36 – 2.26 (m, 1H), 2.19 (qd, *J* = 7.5, 1.1 Hz, 2H), 1.98 – 1.87 (m, 2H), 1.85 (s, 3H), 1.79 – 1.69 (m, 4H), 1.67 (s, 3H), 1.53 – 1.37 (m, 2H).

^{13}C NMR (100 MHz, $CDCl_3$): δ 196.3, 171.6, 168.0, 149.3, 142.3, 139.6, 136.5, 129.3, 128.6, 128.2, 128.1, 128.1, 118.3, 66.3, 62.3, 57.0, 33.7, 30.4, 29.2, 25.8, 23.9, 23.9, 18.2, 12.6.

IR (Neat Film, NaCl): 2930, 1711, 1686, 1452, 1265, 1180 cm^{-1}.

HRMS (MM: FD+): *m/z* calc'd for $C_{26}H_{32}O_5$ $[M]^+$: 424.2250, found 424.2250.

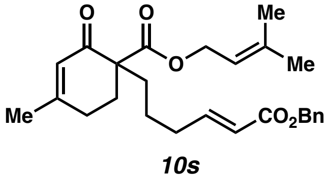

10s

3-methylbut-2-en-1-yl (*E*)-1-(6-(benzyloxy)-6-oxohex-4-en-1-yl)-4-methyl-2-oxocyclohex-3-ene-1-carboxylate (10s)

Prepared from **S2f** and benzyl 2-(diethoxyphosphoryl)acetate[3] following General Procedure B. Purification by flash column chromatography (15–20–25% EtOAc/hexanes) afforded the title compound as a colorless oil (827.2 mg, 2.16 mmol, 64% yield).

^{1}H NMR (400 MHz, CDCl$_3$): δ 7.39 – 7.28 (m, 5H), 6.98 (dt, *J* = 15.6, 6.8 Hz, 1H), 5.87 (dq, *J* = 2.7, 1.3 Hz, 1H), 5.86 (dt, *J* = 15.6, 1.6 Hz, 1H), 5.27 (tp, *J* = 7.1, 1.4 Hz, 1H), 5.16 (s, 2H), 4.64 – 4.51 (m, 2H), 2.52 – 2.36 (m, 2H), 2.28 – 2.14 (m, 3H), 1.98 – 1.83 (m, 5H), 1.72 (s, 4H), 1.67 (s, 3H), 1.54 – 1.34 (m, 2H).

^{13}C NMR (100 MHz, CDCl$_3$): δ 195.9, 171.7, 166.5, 161.4, 149.3, 139.6, 136.3, 128.7, 128.3, 128.3, 126.1, 121.5, 118.4, 66.2, 62.3, 55.9, 33.5, 32.7, 30.2, 28.8, 25.8, 24.2, 23.3, 18.2.

IR (Neat Film, NaCl): 3032, 2938, 1723, 1674, 1438, 1379, 1264, 1212, 1168, 1013, 741, 698 cm^{-1}.

HRMS (MM: FD+): *m/z* calc'd for $C_{26}H_{32}O_5$ [M]$^+$: 424.2235, found 424.2244.

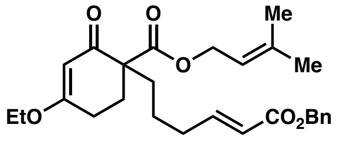

10t

3-methylbut-2-en-1-yl (*E*)-1-(6-(benzyloxy)-6-oxohex-4-en-1-yl)-4-ethoxy-2-oxocyclohex-3-ene-1-carboxylate (10t)

Prepared from **S2g** and benzyl 2-(diethoxyphosphoryl)acetate[3] following General Procedure B. Purification by flash column chromatography (30% EtOAc/hexanes) afforded the title compound as a colorless oil (814.7 mg, 1.79 mmol, 88% yield).

^{1}H NMR (400 MHz, CDCl$_3$): δ 7.40 – 7.28 (m, 5H), 6.98 (dt, *J* = 15.6, 6.8 Hz, 1H), 5.86 (dt, *J* = 15.7, 1.6 Hz, 1H), 5.34 (d, *J* = 1.2 Hz, 1H), 5.28 (dddt, *J* = 7.0, 5.6, 2.8, 1.4 Hz, 1H), 5.16 (s, 2H), 4.66 – 4.52 (m, 2H), 3.89 (qd, *J* = 7.1, 1.6 Hz, 2H), 2.61 (dddd, *J* = 17.9, 10.1, 4.9, 1.2 Hz, 1H), 2.46 – 2.27 (m, 2H), 2.21 (qd, *J* = 7.3, 1.6 Hz, 2H), 2.02 – 1.83 (m, 2H), 1.82 – 1.72 (m, 1H), 1.71 (s, 3H), 1.67 (s, 3H), 1.45 (dddd, *J* = 13.3, 11.2, 6.4, 2.7 Hz, 2H), 1.35 (t, *J* = 7.0 Hz, 3H).

^{13}C NMR (100 MHz, CDCl$_3$): δ 195.8, 176.6, 171.8, 166.5, 149.4, 139.5, 136.3, 128.7, 128.3, 128.3, 121.4, 118.5, 102.3, 66.2, 64.5, 62.3, 56.0, 33.8, 32.7, 28.7, 26.6, 25.8, 23.3, 18.2, 14.3.

IR (Neat Film, NaCl): 2939, 1721, 1655, 1608, 1446, 1380, 1314, 1242, 1190, 1026, 736 cm^{-1}.

HRMS (MM: FD+): *m/z* calc'd for $C_{27}H_{34}O_6$ $[M]^+$: 454.2349, found 454.2350.

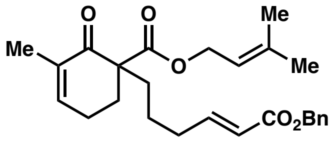

10u

3-methylbut-2-en-1-yl (*E*)-1-(6-(benzyloxy)-6-oxohex-4-en-1-yl)-3-methyl-2-oxocyclohex-3-ene-1-carboxylate (10u)

Prepared from **S2h** and benzyl 2-(diethoxyphosphoryl)acetate[3] following General Procedure B. Purification by flash column chromatography (10–25% EtOAc/hexanes) afforded the title compound as a colorless oil (1176.8 mg, 2.77 mmol, 71% yield).

^{1}H NMR (400 MHz, $CDCl_3$): δ 7.41 – 7.26 (m, 5H), 6.98 (dt, *J* = 15.6, 6.8 Hz, 1H), 6.59 (ddt, *J* = 4.7, 3.1, 1.3 Hz, 1H), 5.87 (dt, *J* = 15.6, 1.6 Hz, 1H), 5.27 (tdq, *J* = 7.1, 2.8, 1.5 Hz, 1H), 5.17 (s, 2H), 4.57 (d, *J* = 6.7 Hz, 2H), 2.50 – 2.36 (m, 2H), 2.33 – 2.16 (m, 3H), 1.98 – 1.83 (m, 2H), 1.78 (q, *J* = 1.7 Hz, 3H), 1.77 – 1.64 (m, 1H), 1.72 (s, 3H), 1.67 (s, 3H) 1.61 – 1.34 (m, 3H).

^{13}C NMR (100 MHz, $CDCl_3$): δ 197.0, 171.9, 166.5, 149.4, 143.8, 139.6, 136.3, 135.4, 128.7, 128.3, 128.3, 121.5, 118.3, 66.2, 62.2, 56.9, 33.6, 32.7, 30.9, 25.8, 23.6, 23.4, 18.2, 16.6.

IR (Neat Film, NaCl): 2921, 1721, 1677, 1450, 1377, 1248, 1168, 728 cm^{-1}.

HRMS (MM: FD+): *m/z* calc'd for $C_{26}H_{32}O_5$ $[M]^+$: 424.2244, found 424.2244.

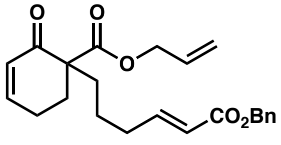

12

allyl (*E*)-1-(6-(benzyloxy)-6-oxohex-4-en-1-yl)-2-oxocyclohex-3-ene-1-carboxylate (12)

Prepared from **S2i** and benzyl 2-(diethoxyphosphoryl)acetate[3] following General Procedure B. Purification by flash column chromatography (20–25% EtOAc/hexanes) afforded the title compound as a colorless oil (599.1 mg, 1.41 mmol, 46% yield).

^{1}H NMR (400 MHz, $CDCl_3$): δ 7.41 – 7.26 (m, 5H), 6.98 (dt, *J* = 15.6, 6.9 Hz, 1H), 6.89 (dddd, *J* = 10.1, 4.8, 3.1, 1.0 Hz, 1H), 6.03 (ddd, *J* = 10.1, 2.5, 1.6 Hz, 1H), 5.93 – 5.77 (m, 2H), 5.27 (dq, *J* = 17.2, 1.5 Hz, 1H), 5.21 (dq, *J* = 10.4, 1.3 Hz, 1H), 5.17 (s, 2H), 4.60 (dq, *J* = 5.6, 1.6 Hz, 2H), 2.57 – 2.42 (m, 2H), 2.39 – 2.27 (m, 1H), 2.22 (qd, *J* = 7.3, 1.6 Hz, 2H), 2.02 – 1.87 (m, 2H), 1.77 (ddd, *J* = 13.7, 11.7, 5.2 Hz, 1H), 1.57 – 1.40 (m, 2H).

^{13}C NMR (100 MHz, $CDCl_3$): δ 196.0, 171.2, 166.5, 149.5, 149.1, 136.2, 131.7, 129.3, 128.7, 128.3, 128.3, 127.8, 127.1, 121.6, 118.7, 66.2, 65.9, 57.0, 33.4, 32.6, 30.3, 23.8, 23.2.

IR (Neat Film, NaCl): 2937, 2357, 1723, 1684, 1456, 1262, 1165, 992 cm^{-1}.

HRMS (MM: FD+): *m/z* calc'd for $C_{23}H_{27}O_5$ $[M]^+$: 383.1871, found 383.1853.

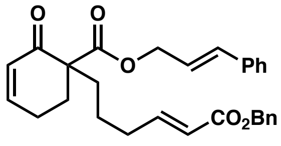

17

cinnamyl 1-((*E*)-6-(benzyloxy)-6-oxohex-4-en-1-yl)-2-oxocyclohex-3-ene-1-carboxylate (17)

Prepared from **S2d** and benzyl 2-(diethoxyphosphoryl)acetate[3] following General Procedure B. Purification by flash column chromatography (20% EtOAc/hexanes) afforded the title compound as a colorless oil (569 mg, 1.24 mmol, 67% yield).

^{1}H NMR (400 MHz, $CDCl_3$): δ 7.37 – 7.34 (m, 6H), 7.34 – 7.29 (m, 3H), 7.27 – 7.24 (m, 1H), 6.97 (dt, *J* = 15.6, 6.9 Hz, 1H), 6.89 (dddd, *J* = 10.1, 4.8, 3.1, 1.0 Hz, 1H), 6.62 (d, *J* = 15.9 Hz, 1H), 6.22 (dt, *J* = 15.9, 6.4 Hz, 1H), 6.04 (ddd, *J* = 10.1, 2.5, 1.6 Hz, 1H), 5.86 (dt, *J* = 15.7, 1.6 Hz, 1H), 5.16 (s, 2H), 4.76 (dt, *J* = 6.4, 1.4 Hz, 2H), 2.56 – 2.45 (m, 2H), 2.37 – 2.28 (m, 1H), 2.22

(ddd, *J* = 7.4, 7.4, 1.6 Hz, 2H), 1.95 (ddt, *J* = 16.8, 7.9, 5.7 Hz, 2H), 1.78 (ddd, *J* = 13.7, 11.7, 5.0 Hz, 1H), 1.56 – 1.42 (m, 2H).

^{13}C NMR (100 MHz, $CDCl_3$): δ 196.0, 171.4, 166.5, 149.5, 149.1, 136.2, 136.2, 134.7, 129.3, 128.7, 128.7, 128.3, 128.3, 126.8, 122.6, 121.6, 66.2, 65.9, 57.0, 33.4, 32.6, 30.3, 23.8, 23.2.

IR (Neat Film, NaCl): 3034, 2942, 1718, 1700, 1684, 1247, 1166 cm^{-1}

HRMS (MM: FD+): *m/z* calc'd for $C_{29}H_{30}O_5$ [M]$^+$: 458.2088, found 458.2082.

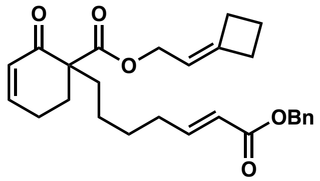

33

22-cyclobutylideneethyl (*E*)-1-(7-(benzyloxy)-7-oxohept-5-en-1-yl)-2-oxocyclohex-3-ene-1-carboxylatecarboxylate (33)

Prepared from **S2l** and benzyl 2-(diethoxyphosphoryl)acetate[3] following General Procedure B. Purification by flash column chromatography (5–60% EtOAc/hexanes) afforded the title compound as a colorless oil (354 mg, 0.81 mmol, 39.8% yield).

^{1}H NMR (400 MHz, $CDCl_3$): δ 7.40 – 7.29 (m, 5H), 6.99 (dt, *J* = 14.9, 1.0 Hz, 1H), 6.91 – 6.84 (m, 1H), 6.02 (d, *J* = 1.2 Hz, 1H), 5.86 (d, *J* = 15.7 Hz, 1H), 5.24 – 5.13 (m, 3H), 4.51 – 4.41 (m, 2H), 2.75 – 2.62 (m, 4H), 2.56 – 2.39 (m, 2H), 2.36 – 2.27 (m, 1H), 2.25 – 2.16 (m, 2H), 2.02 – 1.84 (m, 4H), 1.73 (ddd, *J* = 13.6, 11.2, 5.3 Hz, 1H), 1.47 (p, *J* = 7.5 Hz, 2H), 1.40 – 1.23 (m, 2H).

^{13}C NMR (100 MHz, $CDCl_3$): δ 196.4, 171.6, 166.6, 149.8, 149.3, 148.8, 136.3, 129.4, 128.7, 128.3, 128.3, 121.2, 114.0, 66.2, 62.3, 57.1, 33.6, 32.2, 31.2, 30.3, 29.6, 28.5, 24.3, 23.9, 17.1.

IR (Neat Film, NaCl): 2945, 1722, 1687, 1446, 1169 cm^{-1}.

HRMS (MM: FD+): *m/z* calc'd for $C_{27}H_{32}O_5$ [M]$^+$: 436.2250, found 436.2222.

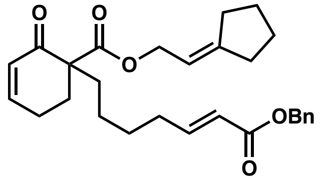

34

2-cyclopentylideneethyl (*E*)-1-(7-(benzyloxy)-7-oxohept-5-en-1-yl)-2-oxocyclohex-3-ene-1-carboxylate (34)

Prepared from **S2k** and benzyl 2-(diethoxyphosphoryl)acetate[3] following General Procedure B. Purification by flash column chromatography (10–60% EtOAc/hexanes) afforded the title compound as a colorless oil (1.40 g, 4.41 mmol, 38.9% yield).

^{1}H NMR (400 MHz, CDCl$_3$): δ 7.40 – 7.28 (m, 5H), 6.99 (dt, *J* = 15.6, 6.9 Hz, 1H), 6.91 – 6.83 (m, 1H), 6.01 (ddd, *J* = 10.1, 2.6, 1.5 Hz, 1H), 5.85 (dt, *J* = 15.6, 1.6 Hz, 1H), 5.37 (tp, *J* = 7.0, 2.2 Hz, 1H), 5.17 (s, 2H), 4.60 – 4.53 (m, 2H), 2.56 – 2.39 (m, 2H), 2.37 – 2.15 (m, 7H), 1.98 – 1.85 (m, 2H), 1.78 – 1.54 (m, 5H), 1.53 – 1.41 (m, 2H), 1.40 – 1.22 (m, 2H).

^{13}C NMR (100 MHz, CDCl$_3$): δ 196.4, 171.7, 166.6, 151.1, 149.8, 149.3, 136.3, 129.4, 128.7, 128.3, 128.3, 121.2, 113.8, 66.2, 63.7, 57.1, 33.9, 33.6, 32.2, 30.3, 29.0, 28.5, 26.4, 26.2, 24.3, 23.9.

IR (Neat Film, NaCl): 2946, 1719, 1686, 1457, 1165 cm^{-1}.

HRMS (MM: FD+): *m/z* calc'd for $C_{28}H_{34}O_5$ $[M]^+$: 450.2406, found 450.2394.

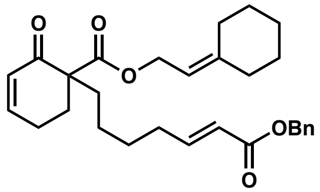

35

2-cyclohexylideneethyl (*E*)-1-(7-(benzyloxy)-7-oxohept-5-en-1-yl)-2-oxocyclohex-3-ene-1-carboxylate (35)

Prepared from **S2j** and benzyl 2-(diethoxyphosphoryl)acetate[3] following General Procedure B. Purification by flash column chromatography (10–60% EtOAc/hexanes) afforded the title compound as a colorless oil (81 mg, 0.17 mmol, 32.9% yield).

^{1}H NMR (400 MHz, CDCl$_3$): δ 7.41 – 7.27 (m, 5H), 6.99 (dt, *J* = 15.6, 6.9 Hz, 1H), 6.87 (dddd, *J* = 10.1, 5.2, 2.5, 1.1 Hz, 1H), 6.01 (ddd, *J* = 10.1, 2.6, 1.5 Hz, 1H), 5.85 (dt, *J* = 15.6, 1.6 Hz, 1H), 5.23 (tp, *J* = 7.3, 1.2 Hz, 1H), 5.17 (s, 2H), 4.59 (d, *J* = 7.2 Hz, 2H), 2.56 – 2.39 (m, 2H), 2.36 – 2.26 (m, 1H), 2.25 – 2.13 (m, 4H), 2.12 – 2.03 (m, 2H), 1.98 – 1.84 (m, 2H), 1.78 – 1.66 (m, 1H), 1.63 – 1.42 (m, 8H), 1.41 – 1.21 (m, 2H).

^{13}C NMR (100 MHz, CDCl$_3$): δ 196.4, 171.6, 166.6, 149.8, 149.3, 147.7, 136.3, 129.4, 128.7, 128.3, 128.3, 121.2, 114.9, 66.2, 61.5, 57.0, 37.1, 33.6, 32.2, 30.4, 29.2, 28.5, 28.5, 27.9, 26.7, 24.3, 23.9.

IR (Neat Film, NaCl): 2929, 2853, 1723, 1681, 1456, 1385, 1266, 1184 cm^{-1}.

HRMS (MM: FD+): *m/z* calc'd for $C_{29}H_{36}O_5$ [M]$^+$: 464.2563, found 464.2543.

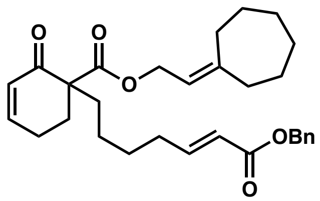

36

2-cycloheptylideneethyl (*E*)-1-(7-(benzyloxy)-7-oxohept-5-en-1-yl)-2-oxocyclohex-3-ene-1-carboxylate (36)

Prepared from **S2n** and benzyl 2-(diethoxyphosphoryl)acetate[3] following General Procedure B. Purification by flash column chromatography (5–70% EtOAc/hexanes) afforded the title compound as a colorless oil (135 mg, 0.28 mmol, 30% yield).

^{1}H NMR (400 MHz, CDCl$_3$): δ 7.43 – 7.29 (m, 5H), 6.99 (dt, *J* = 15.7, 6.9 Hz, 1H), 6.91 – 6.82 (m, 1H), 6.01 (ddd, *J* = 10.2, 2.6, 1.5 Hz, 1H), 5.85 (dt, *J* = 15.6, 1.6 Hz, 1H), 5.27 (tt, *J* = 7.1, 1.3 Hz, 1H), 5.17 (s, 2H), 4.59 (d, *J* = 7.1 Hz, 2H), 2.57 – 2.39 (m, 2H), 2.36 – 2.16 (m, 7H), 1.98 –

1.84 (m, 2H), 1.73 (ddd, *J* = 13.6, 11.3, 5.2 Hz, 1H), 1.61 – 1.43 (m, 10H), 1.31 (dddd, *J* = 13.2, 11.8, 8.6, 6.2 Hz, 2H).

^{13}C NMR (100 MHz, $CDCl_3$): δ 196.4, 171.7, 166.6, 149.8, 149.3, 149.0, 136.3, 129.4, 128.7, 128.3, 128.3, 121.2, 118.4, 66.2, 62.0, 57.0, 37.7, 33.6, 32.2, 30.4, 30.2, 29.8, 29.1, 28.9, 28.5, 27.3, 24.3, 23.9.

IR (Neat Film, NaCl): 2919, 2361, 1722, 1682, 1651, 1443, 1234, 1187 cm^{-1}.

HRMS (MM: FD+): *m/z* calc'd for $C_{30}H_{38}O_5$ $[M]^+$: 478.2719, found 478.2716.

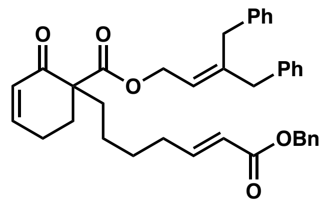

37

3-benzyl-4-phenylbut-2-en-1-yl (*E*)-1-(7-(benzyloxy)-7-oxohept-5-en-1-yl)-2-oxocyclohex-3-ene-1-carboxylate (37)

Prepared from **S2m** and benzyl 2-(diethoxyphosphoryl)acetate[3] following General Procedure B. Purification by flash column chromatography (5–70% EtOAc/hexanes) afforded the title compound as a colorless oil (205 mg, 0.36 mmol, 39% yield).

^{1}H NMR (400 MHz, $CDCl_3$): δ 7.39 – 7.24 (m, 9H), 7.23 – 7.17 (m, 2H), 7.09 (dd, *J* = 11.6, 7.3 Hz, 4H), 6.98 (dt, *J* = 15.6, 6.9 Hz, 1H), 6.89 – 6.80 (m, 1H), 6.02 (d, *J* = 10.1 Hz, 1H), 5.85 (d, *J* = 16.1 Hz, 1H), 5.51 (t, *J* = 7.2 Hz, 1H), 5.17 (s, 2H), 4.83 – 4.69 (m, 2H), 3.36 (s, 2H), 3.23 (s, 2H), 2.55 – 2.40 (m, 2H), 2.37 – 2.24 (m, 1H), 2.18 (q, *J* = 7.2 Hz, 2H), 2.00 – 1.85 (m, 2H), 1.75 (ddd, *J* = 13.5, 11.1, 5.2 Hz, 1H), 1.46 (p, *J* = 7.4 Hz, 1H), 1.38 – 1.22 (m, 2H).

^{13}C NMR (100 MHz, $CDCl_3$): δ 196.2, 171.6, 166.6, 149.8, 149.3, 144.8, 139.0, 138.8, 136.3, 129.4, 129.3, 128.8, 128.7, 128.7, 128.5, 128.3, 128.3, 126.5, 126.4, 121.7, 121.3, 66.2, 61.9, 57.1, 42.9, 35.8, 33.6, 32.1, 30.3, 28.5, 24.3, 23.9.

IR (Neat Film, NaCl): 3027, 2931, 1722, 1682, 1493, 1387, 1264, 1165 cm^{-1}.

HRMS (MM: FD+): *m/z* calc'd for $C_{38}H_{40}O_5$ $[M]^+$: 576.2876, found 576.2857.

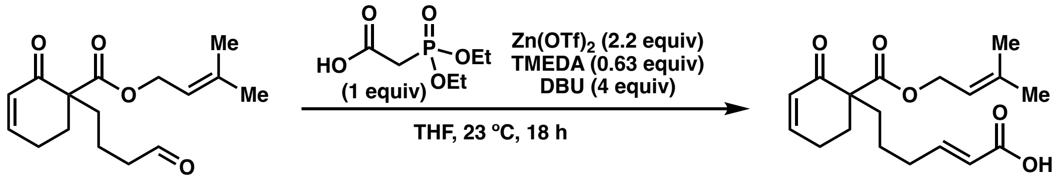

S2a **10i**

(*E*)-6-(1-(((3-methylbut-2-en-1-yl)oxy)carbonyl)-2-oxocyclohex-3-en-1-yl)hex-2-enoic acid (10i)[8]

To a suspension of $Zn(OTf)_2$ (6.6 mmol, 2.2 equiv) in THF (15 mL) was added (diethoxyphosphinyl)acetic acid (3 mmol, 1 equiv), followed by the addition of TMEDA (1.89 mmol, 0.63 equiv), DBU (12 mmol, 4 equiv), and then a solution of aldehyde **S2a** (3 mmol, 1 equiv) in THF (2 mL). The solution was stirred at 23 ºC for 18 h, and the reaction was diluted with 1 M HCl and extracted with dichloromethane (4x). The combined organic layers were dried over Na_2SO_4, filtered, and concentrated under reduced pressure. Purification by silica gel flash column chromatography (35% EtOAc/hexanes with 3% AcOH) afforded the title compound as a white solid (137.4 mg, 0.43 mmol, 43% yield).

^{1}H NMR (400 MHz, $CDCl_3$): δ 7.04 (dt, *J* = 15.7, 6.8 Hz, 1H), 6.93 – 6.84 (m, 1H), 6.03 (ddd, *J* = 10.2, 2.6, 1.5 Hz, 1H), 5.83 (dt, *J* = 15.6, 1.6 Hz, 1H), 5.28 (tp, *J* = 7.2, 1.4 Hz, 1H), 4.66 – 4.53 (m, 2H), 2.58 – 2.40 (m, 2H), 2.37 – 2.28 (m, 1H), 2.25 (qd, *J* = 7.3, 1.6 Hz, 2H), 1.98 – 1.87 (m, 2H), 1.80 – 1.68 (m, 1H), 1.73 (s, 3H), 1.68 (s, 3H), 1.61 – 1.37 (m, 3H).

^{13}C NMR (100 MHz, $CDCl_3$): δ 196.3, 171.6, 170.2, 151.4, 149.3, 139.7, 129.4, 120.8, 118.3, 62.4, 57.0, 33.5, 32.7, 30.5, 25.8, 23.9, 23.1, 18.2.

IR (Neat Film, NaCl): 2929, 1725, 1694, 1424, 1384, 1236, 1171 cm^{-1}.

HRMS (MM: FD+): *m/z* calc'd for $C_{18}H_{24}O_5$ $[M]^+$: 320.1624, found 320.1636.

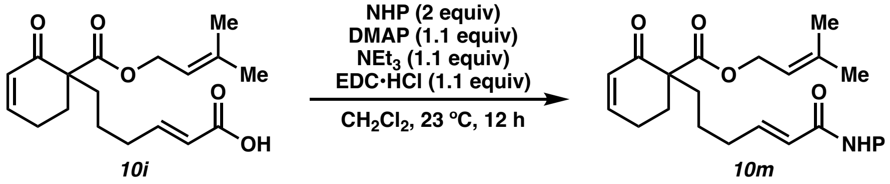

3-methylbut-2-en-1-yl (*E*)-1-(6-((1,3-dioxoisoindolin-2-yl)oxy)-6-oxohex-4-en-1-yl)-2-oxocyclohex-3-ene-1-carboxylate (10m)[9]

To a round bottom flask was added crude acid **10i** (assumed quantitative yield from previous reaction, 1 mmol, 1 equiv), DMAP (1.1 mmol, 1.1 equiv), NHP (2 mmol, 2 equiv), dichloromethane (9.5 mL), and triethylamine (1.1 mmol, 1.1 equiv). EDC•HCl (1.1 mmol, 1.1 equiv) was then added under N_2 atmosphere in a single portion, and the reaction was stirred vigorously at 23 ºC for 12 h. The reaction mixture was diluted with dichloromethane and washed with 0.5 N HCl, saturated aqueous $NaHCO_3$, and brine. The combined organic layers were dried over Na_2SO_4 and concentrated under reduced pressure. Purification by flash column chromatography (30–35% EtOAc/hexanes) afforded the title compound as a colorless oil (109.6 mg, 0.48 mmol, 24% yield over two steps).

^{1}H NMR (400 MHz, CDCl$_3$): δ 7.96 – 7.84 (m, 2H), 7.79 (dd, *J* = 5.5, 3.1 Hz, 2H), 7.30 (dd, *J* = 15.8, 6.7 Hz, 1H), 6.89 (dddd, *J* = 10.1, 4.9, 3.0, 1.1 Hz, 1H), 6.10 (dt, *J* = 15.8, 1.6 Hz, 1H), 6.04 (ddd, *J* = 10.1, 2.5, 1.5 Hz, 1H), 5.29 (tdt, *J* = 5.7, 2.8, 1.4 Hz, 1H), 4.67 – 4.55 (m, 2H), 2.58 – 2.40 (m, 2H), 2.35 (m, 3H), 2.03 – 1.89 (m, 2H), 1.87 – 1.74 (m, 1H), 1.74 (s, 3H), 1.69 (d, *J* = 1.3 Hz, 3H), 1.66 – 1.45 (m, 3H).

^{13}C NMR (100 MHz, CDCl$_3$): δ 196.2, 162.4, 162.2, 155.2, 149.3, 139.8, 134.8, 129.4, 129.1, 124.1, 118.3, 116.0, 62.4, 57.0, 33.5, 33.3, 30.6, 25.9, 23.9, 23.0, 18.3.

IR (Neat Film, NaCl): 1771, 1744, 1682, 1185 cm^{-1}.

HRMS (MM: FD+): *m/z* calc'd for $C_{26}H_{27}NO_7$ [M]$^+$: 465.1788, found 465.1779.

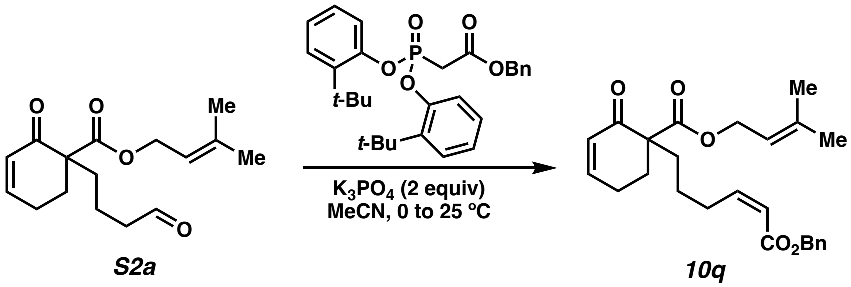

3-methylbut-2-en-1-yl (*Z*)-1-(6-(benzyloxy)-6-oxohex-4-en-1-yl)-2-oxocyclohex-3-ene-1-carboxylate (10q)

To a solution of benzyl 2-(bis(2-(*tert*-butyl)phenoxy)phosphoryl)acetate[10] (890 mg, 1.80 mmol, 1.00 equiv) in MeCN (18 mL, 0.1 M) was added K_2CO_3 (783 mg, 3.69 mmol, 2.05 equiv). The reaction was cooled to 0 ºC and a solution of **S2a** (500 mg, 1.80 mmol, 1.00 equiv) in MeCN (18 mL, 0.1 M) was dropwise added. The reaction was gradually warmed to 25 ºC and stirring was continued until consumption of **S2a** as determined by TLC (around 16 h). The reaction mixture was filtered through a plug of Celite® to remove solids and volatiles were removed in vacuo. Purification by flash column chromatography (0–40% EtOAc/hexanes) afforded the title compound as a colorless oil (482 mg, 1.17 mmol, 65 % yield).

^{1}H NMR (400 MHz, $CDCl_3$): δ 7.39 – 7.29 (m, 5H), 6.90 – 6.84 (m, 1H), 6.24 (dt, *J* = 11.5, 7.4 Hz, 1H), 6.01 (ddd, *J* = 10.1, 2.5, 1.6 Hz, 1H), 5.83 (dt, *J* = 11.5, 1.7 Hz, 1H), 5.28 (ddp, *J* = 8.6, 5.7, 1.4 Hz, 1H), 5.15 (s, 2H), 4.59 (d, *J* = 7.2 Hz, 2H), 2.68 (qd, *J* = 7.4, 1.8 Hz, 2H), 2.53 – 2.41 (m, 2H), 2.35 – 2.26 (m, 1H), 1.98 – 1.87 (m, 2H), 1.79 – 1.71 (m, 4H), 1.67 (s, 3H), 1.52 – 1.37 (m, 2H).

^{13}C NMR (100 MHz, $CDCl_3$): δ 196.4, 171.7, 166.2, 150.5, 149.3, 139.5, 136.3, 129.3, 128.7, 128.3, 128.3, 119.9, 118.4, 65.9, 62.3, 57.1, 33.4, 30.3, 29.4, 25.8, 24.2, 23.8, 18.2.

IR (Neat Film, NaCl): 2918, 1714, 1447, 1178, 1161 cm^{-1}.

HRMS (MM: FD+): *m/z* calc'd for $C_{25}H_{30}O_5$ $[M]^+$: 410.2093, found 410.2108.

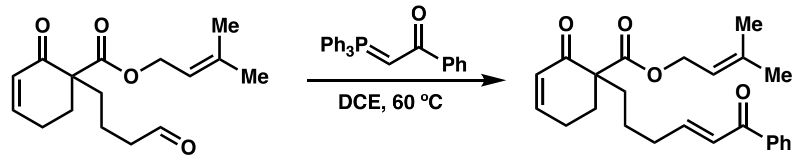

3-methylbut-2-en-1-yl (*E*)-2-oxo-1-(6-oxo-6-phenylhex-4-en-1-yl)cyclohex-3-ene-1-carboxylate (10j)

(2-oxo-2-phenylethyl)triphenylphosphonium bromide[11] (996 mg, 2.16 mmol, 1.2 equiv) was stirred in 26 mL of a 3:2 CH_2Cl_2/2 M aq. NaOH mixture for 30 minutes at 23 ºC. The layers were separated, and the aqueous layer was extracted twice with CH_2Cl_2. The combined organic layers were washed with brine, dried over Na_2SO_4, filtered, and solvent was removed in vacuo. To a solution of this crude ylide in DCE (22 mL, 0.1 M) was added aldehyde **S2a** (500 mg, 1.80 mmol, 1 equiv). The reaction was stirred at 65 ºC for 36 hours. Upon complete consumption of **S2a**, as determined by TLC, volatiles were removed in vacuo. Purification by flash column chromatography (30% EtOAc/hexanes) afforded the title compound as a colorless oil (330 mg, 0.867 mmol, 48% yield).

^{1}H NMR (400 MHz, $CDCl_3$): δ 7.95 – 7.90 (m, 2H), 7.60 – 7.51 (m, 1H), 7.46 (tt, *J* = 6.8, 1.5 Hz, 2H), 7.02 (dt, *J* = 15.4, 6.7 Hz, 1H), 6.93 – 6.85 (m, 2H), 6.03 (ddd, *J* = 10.2, 2.6, 1.5 Hz, 1H), 5.30 – 5.25 (m, 1H), 4.59 (d, *J* = 7.1 Hz, 2H), 2.55 – 2.43 (m, 2H), 2.38 – 2.27 (m, 3H), 2.01 – 1.91 (m, 2H), 1.80 (ddd, *J* = 13.6, 12.0, 4.7 Hz, 1H), 1.72 (s, 3H), 1.67 (s, 3H), 1.63 – 1.47 (m, 2H).

^{13}C NMR (100 MHz, $CDCl_3$): δ 196.3, 191.0, 171.6, 149.3, 149.1, 139.7, 138.1, 132.8, 129.4, 128.7, 128.7, 126.4, 118.3, 62.4, 57.0, 33.6, 33.2, 30.5, 25.8, 23.9, 23.4, 18.2.

IR (Neat Film, NaCl): 2931, 1724, 1671, 1619, 1447, 1229, 1177 cm^{-1}.

HRMS (MM: FD+): *m/z* calc'd for $C_{24}H_{28}O_4$ $[M]^+$: 380.1988, found 380.1982.

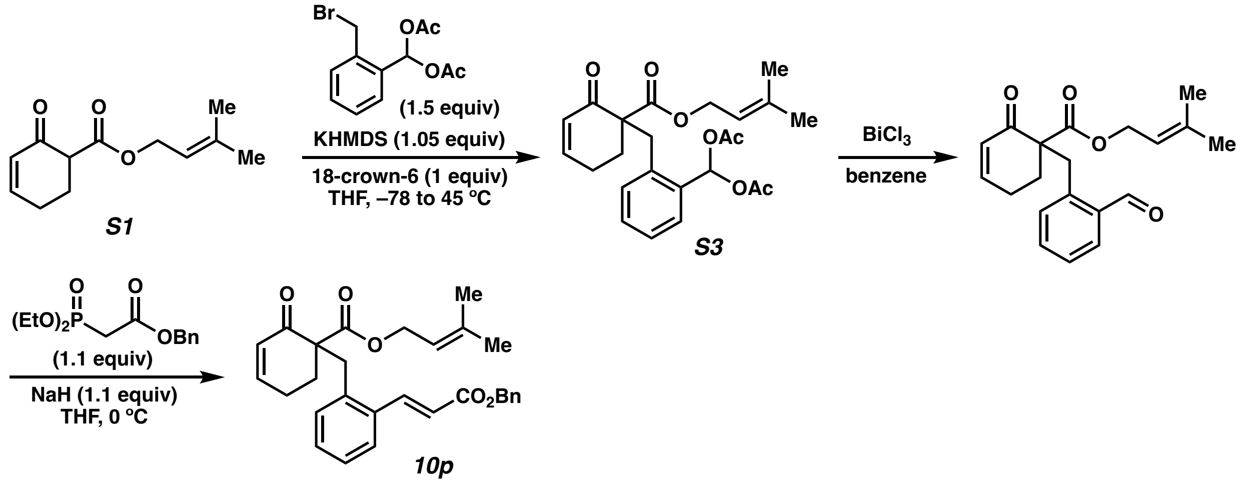

(2-((1-(((3-methylbut-2-en-1-yl)oxy)carbonyl)-2-oxocyclohex-3-en-1-yl)methyl)phenyl)methylene diacetate (S3)

An oven dried round bottom flask was charged with KHMDS (837 mg, 4.20 mmol, 1.05 equiv), 18-crown-6 (1.06 g, 4.00 mmol, 1.0 equiv), and THF (21 mL). The mixture was cooled to –78 ºC and a solution of enone **S1** (830 mg, 4.00 mmol, 1.0 equiv) in THF (10 mL) was added. The reaction mixture was stirred for 15 minutes then (2-(bromomethyl)phenyl)methylene diacetate[12] (1.86 g, 6.00 mmol, 1.5 equiv) was added in a minimal amount of THF (*ca* 5 mL). The solution was slowly warmed to 45 ºC and stirred for 14 h. Upon complete consumption of starting material (as determined by TLC), the solution was cooled to 23 ºC, diluted with a saturated aqueous solution of NH_4Cl, and the reaction mixture was extracted thrice with EtOAc. The combined organic layers were dried over Na_2SO_4, filtered, and concentrated under reduced pressure. Purification by flash column chromatography (20–60% EtOAc/hexanes) afforded the title compound as a colorless oil (1.00 g, 2.33 mmol, 58% yield).

^{1}H NMR (400 MHz, $CDCl_3$): δ 7.87 (s, 1H), 7.58 – 7.52 (m, 1H), 7.30 – 7.24 (m, 2H), 7.21 – 7.16 (m, 1H), 6.88 – 6.82 (m, 1H), 6.07 (ddd, J = 10.1, 2.8, 1.3 Hz, 1H), 5.24 (ddq, J = 8.6, 5.7, 1.4 Hz, 1H), 4.56 (d, J = 7.2 Hz, 2H), 3.52 (d, J = 14.8 Hz, 1H), 3.45 (d, J = 14.8 Hz, 1H), 2.53 – 2.41 (m, 1H), 2.36 (dddd, J = 13.6, 4.9, 2.6, 1.3 Hz, 1H), 2.29 – 2.19 (m, 1H), 2.12 (s, 3H), 2.10 (s, 3H), 1.86 (ddd, J = 13.6, 10.4, 5.3 Hz, 1H), 1.73 (s, 3H), 1.67 (s, 3H).

^{13}C NMR (100 MHz, CDCl$_3$): δ 195.4, 171.1, 168.8, 168.8, 149.6, 139.7, 135.5, 134.9, 131.5, 129.6, 129.5, 127.6, 127.2, 118.2, 88.4, 62.5, 58.2, 34.2, 30.2, 25.9, 24.1, 21.0, 21.0, 18.2.

IR (Neat Film, NaCl): 2935, 1759, 1731, 1682, 1447, 1371, 1236, 1206 cm^{-1}.

HRMS (MM: FD+): *m/z* calc'd for $C_{24}H_{28}O_7$ $[M]^+$: 428.1835, found 428.1833.

3-methylbut-2-en-1-yl (*E*)-1-(6-(benzyloxy)-6-oxohex-4-en-1-yl)-2-oxocyclohept-3-ene-1-carboxylate (10p)

To a solution of diacetate **S3** (500 mg, 1.17 mmol, 1 equiv) in benzene (11.7 mL, 0.1 M) was added bismuth chloride (38 mg, 0.12 mmol, 0.1 equiv). The reaction mixture was heated to 35 ºC for 3 hours. Upon cooling to 25 ºC, the reaction mixture was diluted with water and the layers were separated. The aqueous layer was extracted twice with chloroform. The combined organic layers were washed with brine, dried over Na_2SO_4, and volatiles were removed in vacuo. The crude aldehyde was used directly in the subsequent Horner–Wadsworth–Emmons olefination.

To a suspension of NaH (52 mg, 1.29 mmol, 60% by weight in mineral oil, 1.1 equiv) in THF (2.6 mL, 0.5 M) at 0 ºC was dropwise added a solution of benzyl 2-(diethoxyphosphoryl)acetate[3] (369 mg, 1.29 mmol, 1.1 equiv) in THF (1.3 mL, 1.0 M). Stirring at 0 ºC was continued for 30 minutes. To the reaction was then dropwise added a solution of the crude aldehyde in THF (2.4 mL, 0.5 M). Upon complete consumption of starting material (as determined by TLC), the reaction mixture was diluted with a saturated solution of $NaHCO_3$ and extracted with EtOAc (3x). The combined organic layers were dried over Na_2SO_4, filtered, and concentrated under reduced pressure. Purification by flash column chromatography (5–40% EtOAc/hexanes) afforded the title compound as a colorless oil (230 mg, 0.502 mmol, 43% yield).

^{1}H NMR (400 MHz, CDCl$_3$): δ 8.08 (d, *J* = 15.7 Hz, 1H), 7.57 (dd, *J* = 7.5, 1.9 Hz, 1H), 7.44 – 7.31 (m, 5H), 7.27 – 7.17 (m, 3H), 6.85 – 6.80 (m, 1H), 6.40 (d, *J* = 15.7 Hz, 1H), 6.06 (ddd, *J* = 10.1, 2.9, 1.2 Hz, 1H), 5.28 – 5.21 (m, 3H), 4.56 – 4.49 (m, 2H), 3.57 (d, *J* = 14.3 Hz, 1H), 3.31 (d, *J* = 14.4 Hz, 1H), 2.51 – 2.39 (m, 1H), 2.30 – 2.16 (m, 2H), 1.82 – 1.70 (m, 4H), 1.65 (s, 3H).

^{13}C NMR (100 MHz, CDCl$_3$): δ 195.1, 170.4, 166.7, 149.7, 143.2, 139.7, 136.7, 136.2, 134.7, 132.1, 130.0, 129.3, 128.7, 128.4, 128.3, 127.5, 126.8, 119.4, 118.2, 66.5, 62.5, 58.4, 35.4, 30.2, 25.9, 24.1, 18.2.

IR (Neat Film, NaCl): 3028, 2927, 1720, 1686, 1629, 1168 cm^{-1}.

HRMS (MM: FD+): *m/z* calc'd for $C_{29}H_{30}O_5$ [M]$^+$: 458.2088, found 458.2086.

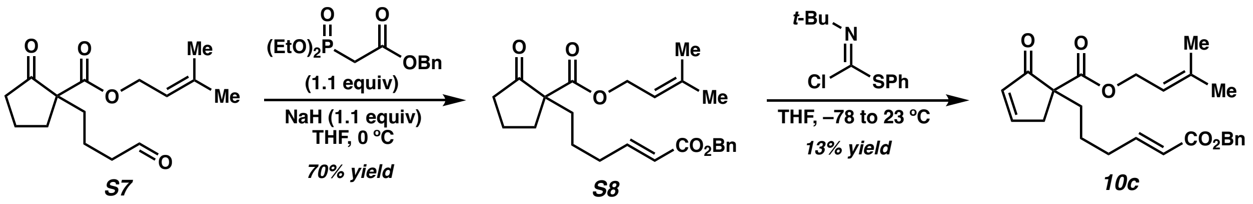

3-methylbut-2-en-1-yl (*E*)-1-(6-(benzyloxy)-6-oxohex-4-en-1-yl)-2-oxocyclopentane-1-carboxylate (S8)

Prepared from **S7** and benzyl 2-(diethoxyphosphoryl)acetate[3] following General Procedure B. Purification by flash column chromatography (15% EtOAc/hexanes) afforded the title compound as a colorless oil (1.40 g, 3.51 mmol, 70% yield).

^{1}H NMR (400 MHz, CDCl$_3$): δ 7.76 (dt, *J* = 5.6, 2.7 Hz, 1H), 7.42 – 7.26 (m, 5H), 6.95 (dt, *J* = 15.6, 6.9 Hz, 1H), 6.16 (dt, *J* = 5.8, 2.2 Hz, 1H), 5.85 (dt, *J* = 15.6, 1.6 Hz, 1H), 5.27 (tdq, *J* = 7.2, 2.9, 1.5 Hz, 1H), 5.16 (s, 2H), 4.59 (d, *J* = 7.1 Hz, 2H), 3.33 – 3.19 (m, 1H), 2.68 – 2.51 (m, 1H), 2.21 (qd, *J* = 7.3, 1.6 Hz, 2H), 1.99 (ddd, *J* = 13.7, 12.3, 4.5 Hz, 1H), 1.82 – 1.70 (m, 1H), 1.73 (s, 3H), 1.67 (s, 3H), 1.53 – 1.23 (m, 3H).

^{13}C NMR (100 MHz, CDCl$_3$): δ 205.7, 170.6, 166.4, 163.9, 148.9, 139.6, 136.2, 132.4, 128.7, 128.4, 128.3, 121.7, 118.3, 66.2, 62.7, 58.0, 39.5, 34.0, 32.4, 25.9, 23.2, 18.2.

IR (Neat Film, NaCl): 2932, 2356, 1715, 1263, 1164, 976, 754 cm^{-1}.

HRMS (MM: FD+): *m/z* calc'd for $C_{24}H_{28}O_5$ [M]$^+$: 396.1920, found 396.1931.

3-methylbut-2-en-1-yl (*E*)-1-(6-(benzyloxy)-6-oxohex-4-en-1-yl)-2-oxocyclopent-3-ene-1-carboxylate (10c)

A flame dried round bottom flask was charged with *i*-Pr_2NH (0.35 mL, 2.5 mmol, 1.25 equiv) and THF (8.0 mL, 0.25 M). The solution was cooled to –78 ºC and *n*-BuLi (0.96 mL, 2.4 mmol, 1.2 equiv) was added dropwise and the resultant solution was stirred for 30 min. **S8** (797 mg, 2.00 mmol, 1.0 equiv) in THF (8.0 mL, 0.25 M) was added dropwise and the mixture was stirred for 1 h. *N*-*tert*-Butylbenzenesulfinimidoyl chloride[13] (560.9 mg, 2.6 mmol, 1.3 equiv) in THF (4.0 mL, 0.5 M) was added dropwise and the solution was slowly warmed to 23 ºC. Upon complete consumption of starting material (as determined by TLC), the reaction mixture was diluted with saturated aqueous $NaHCO_3$ solution and extracted with Et_2O (25 mL x 3). The combined organic layers were dried with Na_2SO_4, filtered, and concentrated under reduced pressure. The crude product was purified by column chromatography (SiO_2, 0–40% EtOAc/Hexanes) to afford enone **10b** as a colorless oil (100 mg, 0.25 mmol, 13% yield).

^{1}H NMR (400 MHz, $CDCl_3$): δ 7.76 (dt, *J* = 5.6, 2.7 Hz, 1H), 7.42 – 7.26 (m, 5H), 6.95 (dt, *J* = 15.6, 6.9 Hz, 1H), 6.16 (dt, *J* = 5.8, 2.2 Hz, 1H), 5.85 (dt, *J* = 15.6, 1.6 Hz, 1H), 5.27 (tdq, *J* = 7.2, 2.9, 1.5 Hz, 1H), 5.16 (s, 2H), 4.59 (d, *J* = 7.1 Hz, 2H), 3.33 – 3.19 (m, 1H), 2.68 – 2.51 (m, 1H), 2.21 (qd, *J* = 7.3, 1.6 Hz, 2H), 1.99 (ddd, *J* = 13.7, 12.3, 4.5 Hz, 1H), 1.82 – 1.70 (m, 1H), 1.73 (s, 3H), 1.67 (s, 3H), 1.53 – 1.23 (m, 3H).

^{13}C NMR (100 MHz, $CDCl_3$): δ 205.7, 170.6, 166.4, 163.9, 148.9, 139.6, 136.2, 132.4, 128.7, 128.4, 128.3, 121.7, 118.3, 66.2, 62.7, 58.0, 39.5, 34.0, 32.4, 25.9, 23.2, 18.2.

IR (Neat Film, NaCl): 2932, 2356, 1715, 1263, 1164, 976, 754 cm^{-1}.

HRMS (MM: FD+): *m/z* calc'd for $C_{24}H_{28}O_5$ $[M]^+$: 396.1920, found 396.1931.

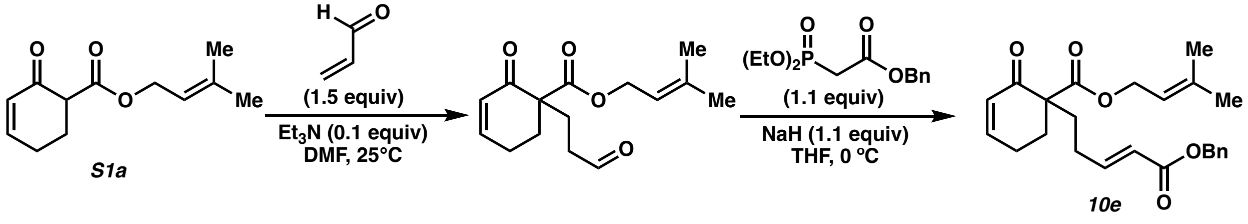

3-methylbut-2-en-1-yl (*E*)-1-(5-(benzyloxy)-5-oxopent-3-en-1-yl)-2-oxocyclohex-3-ene-1-carboxylate (10e)

To a solution of enone **S1a** (1.04 g, 5.00 mmol, 1.0 equiv) in DMF (10 mL, 0.5 M) at 25°C was added dropwise triethylamine (0.07 mL, 0.50 mmol, 0.1 equiv) followed by acrolein (0.50 mL, 7.50 mmol, 1.5 equiv). Upon consumption of starting material (as determined by TLC), the reaction mixture was diluted with water and extracted thrice with diethyl ether. The combined organic layers were washed with water followed by brine, dried over Na_2SO_4, and volatiles were removed in vacuo. The crude aldehyde was used directly in the subsequent Horner–Wadsworth–Emmons olefination.

To a suspension of NaH (132 mg, 3.30 mmol, 60% by weight in mineral oil, 1.1 equiv) in THF (6 mL, 0.5 M) at 0 ºC was dropwise added a solution of benzyl 2-(diethoxyphosphoryl)acetate (945 mg, 3.30 mmol, 1.1 equiv) in THF (3.0 mL, 1.0 M). Stirred at 0 ºC was continued for 30 minutes. To the reaction was then dropwise added a solution of the crude aldehyde in THF (6.0 mL, 0.5 M). Upon complete consumption of starting material (as determined by TLC), the reaction mixture was diluted with a saturated solution of $NaHCO_3$ and extracted with EtOAc (3x). The combined organic layers were dried over Na_2SO_4, filtered, and concentrated under reduced pressure. Purification by flash column chromatography (10–20% EtOAc/hexanes) afforded the title compound as a colorless oil (490 mg, 1.24 mmol, 41% yield).

^{1}H NMR (400 MHz, CDCl$_3$): δ 7.41 – 7.29 (m, 5H), 6.99 (dt, *J* = 15.7, 6.8 Hz, 1H), 6.93 – 6.84 (m, 1H), 6.03 (ddd, *J* = 10.1, 2.5, 1.5 Hz, 1H), 5.88 (dt, *J* = 15.6, 1.6 Hz, 1H), 5.27 (ddt, *J* = 7.2, 5.8, 1.4 Hz, 1H), 5.17 (s, 2H), 4.59 (d, *J* = 7.1 Hz, 2H), 2.56 – 2.41 (m, 2H), 2.38 – 2.13 (m, 3H), 2.08 – 1.81 (m, 3H), 1.73 (s, 3H), 1.67 (s, 3H).

^{13}C NMR (100 MHz, CDCl$_3$): δ 196.0, 171.4, 166.4, 149.3, 148.9, 139.9, 136.2, 129.4, 128.7, 128.3, 121.5, 118.2, 66.2, 62.4, 56.6, 32.2, 30.7, 27.6, 25.8, 23.8, 18.2.

IR (Neat Film, NaCl): 3032, 2934, 1737, 1681, 1445, 1384, 1265, 1175, 1137 cm^{-1}.

HRMS (MM: FD+): *m/z* calc'd for $C_{24}H_{28}O_5$ $[M]^+$: 396.1937, found 396.1926.

Preparation of Aldehyde Precursors

General Procedure C: Alkylation of β-Ketoesters

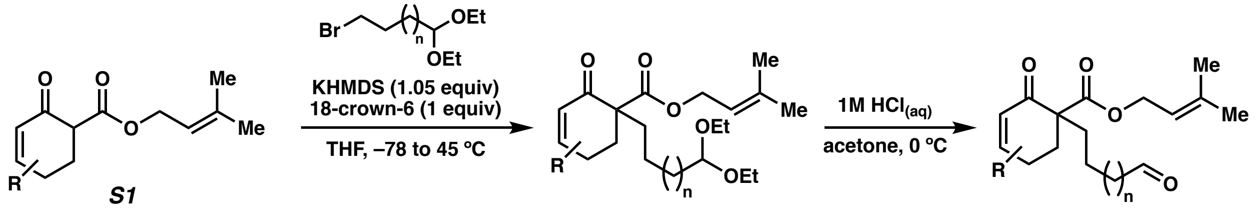

An oven dried round bottom flask was charged with KHMDS (1.05 equiv), 18-crown-6 (1.0 equiv), and THF (0.2 M with respect to KHMDS). The mixture was cooled to –78 ºC and a solution of acyclated enone **S1** (1.0 equiv) in THF (0.4 M) was added. The reaction mixture was stirred for 15 minutes and then the appropriate alkyl bromide (1.5 equiv) was added neat dropwise. The solution was slowly warmed to 45 ºC and stirred for 14 h. Upon complete consumption of starting material (as determined by TLC), the solution was cooled to 23 ºC, diluted with a saturated aqueous solution of NH_4Cl and the reaction mixture was extracted thrice with EtOAc. The combined organic layers were dried over Na_2SO_4, filtered, and concentrated under reduced pressure to afford the crude diethyl acetal which was used directly in the next step. A round bottom flask was charged with the crude acetal and acetone (0.5 M), then cooled to 0 ºC. Aqueous 1 M HCl (1:1 volume with respect to acetone) was added and stirring was continued for 1 h. Upon complete consumption of starting material (as determined by TLC), the reaction mixture was extracted with EtOAc (3x). The combined organic layers were washed with brine, dried over Na_2SO_4, filtered, and concentrated under reduced pressure. The crude product was purified by silica gel flash column chromatography to afford the respective aldehyde product (**S2**).

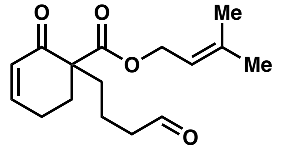

S2a

3-methylbut-2-en-1-yl 2-oxo-1-(4-oxobutyl)cyclohex-3-ene-1-carboxylate (S2a)

Prepared from **S1a** and 4-bromo-1,1-diethoxybutane [14] following General Procedure C. Purification by flash column chromatography (25% EtOAc/hexanes) afforded the title compound as a colorless oil (1.81 g, 6.50 mmol, 49% yield).

^{1}H NMR (400 MHz, CDCl$_3$): δ 9.75 (t, *J* = 1.5 Hz, 1H), 6.89 (dddd, *J* = 10.1, 4.4, 3.1, 1.1 Hz, 1H), 6.02 (ddd, *J* = 10.1, 2.5, 1.6 Hz, 1H), 5.31 – 5.25 (m, 1H), 4.59 (d, *J* = 6.8 Hz, 2H), 2.55 – 2.43 (m, 4H), 2.39 – 2.30 (m, 1H), 2.02 – 1.94 (m, 1H), 1.89 (ddd, *J* = 12.5, 11.7, 4.5 Hz, 1H), 1.78 (dd, *J* = 11.6, 4.9 Hz, 1H), 1.73 (s, 3H), 1.71 – 1.59 (m, 5H)

^{13}C NMR (100 MHz, CDCl$_3$): δ 202.2, 196.2, 171.5, 149.5, 139.7, 129.3, 118.3, 62.4, 57.0, 44.2, 33.2, 30.3, 25.8, 23.8, 18.2, 17.5.

IR (Neat Film, NaCl): 2942, 1732, 1716, 1456, 1180 cm^{-1}

HRMS (MM: FD+): *m/z* calc'd for $C_{16}H_{22}O_4$ [M]$^+$: 278.1518, found 278.1509.

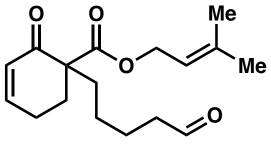

S2b

3-methylbut-2-en-1-yl 2-oxo-1-(5-oxopentyl)cyclohex-3-ene-1-carboxylate (S2b)

Prepared from **S1a** and 5-bromo-1,1-diethoxypentane[14] following General Procedure C. Purification by flash column chromatography (10–25% EtOAc/hexanes) afforded the title compound as a colorless oil (648 mg, 2.22 mmol, 38% yield).

^{1}H NMR (400 MHz, CDCl$_3$): δ 9.75 (s, 1H), 6.91 – 6.84 (m, 1H), 6.01 (ddd, *J* = 10.1, 2.6, 1.6 Hz, 1H), 5.27 (tdq, *J* = 7.1, 2.8, 1.4 Hz, 1H), 4.58 (d, *J* = 7.1 Hz, 2H), 2.54 – 2.40 (m, 4H), 2.36 – 2.27 (m, 1H), 1.98 – 1.86 (m, 2H), 1.78 – 1.60 (m, 10H), 1.40 – 1.26 (m, 2H).

^{13}C NMR (100 MHz, CDCl$_3$): δ 202.6, 196.4, 171.7, 149.4, 139.6, 129.3, 118.3, 62.3, 57.0, 43.7, 33.6, 30.4, 25.8, 24.3, 23.8, 22.5, 18.2.

IR (Neat Film, NaCl): 2941, 1733, 1717, 1456, 1219 cm^{-1}

HRMS (MM: FD+): *m/z* calc'd for $C_{16}H_{22}O_4$ $[M]^+$: 293.1747, found 293.1768.

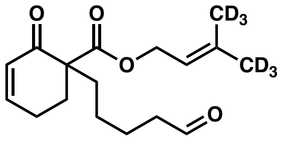

D-S2b

3-(methyl-*d*3)but-2-en-1-yl-4,4,4-*d*3 2-oxo-1-(5-oxopentyl)cyclohex-3-ene-1-carboxylate (*D*-S2b)

Prepared from ***D*-S1a** and 5-bromo-1,1-diethoxypentane[14] following General Procedure C. Purification by flash column chromatography (10–25% EtOAc/hexanes) afforded the title compound as a colorless oil (290 mg, 0.971 mmol, 42% yield).

^{1}H NMR (400 MHz, CDCl$_3$): δ 9.75 (t, *J* = 1.7 Hz, 1H), 6.90 – 6.85 (m, 1H), 6.01 (ddd, *J* = 10.1, 2.6, 1.6 Hz, 1H), 5.27 (t, *J* = 7.2 Hz, 1H), 4.58 (dd, *J* = 7.2, 1.8 Hz, 2H), 2.55 – 2.41 (m, 4H), 2.36 – 2.26 (m, 1H), 1.97 – 1.86 (m, 2H), 1.74 (ddd, *J* = 13.6, 11.6, 5.1 Hz, 1H), 1.68 – 1.60 (m, 2H), 1.40 – 1.24 (m, 2H).

^{13}C NMR (100 MHz, CDCl$_3$): δ 202.6, 196.4, 171.7, 149.4, 139.4, 129.3, 118.4, 62.3, 57.0, 43.7, 33.6, 30.4, 24.3, 23.9, 22.5.

^{2}H NMR (61 MHz, CHCl$_3$): δ 1.69, 1.65.

IR (Neat Film, NaCl): 2941, 1726, 1682, 1238, 1186 cm^{-1}

HRMS (MM: FD+): *m/z* calc'd for $C_{17}H_{18}D_6O_4$ $[M]^+$: 298.2051, found 298.2052.

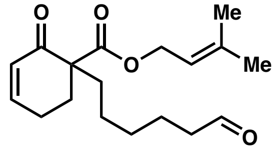

S2c

3-methylbut-2-en-1-yl 2-oxo-1-(6-oxohexyl)cyclohex-3-ene-1-carboxylate (S2c)

Prepared from **S1a** and 6-bromo-1,1-diethoxyhexane[14] following General Procedure C. Purification by flash column chromatography (10–25% EtOAc/hexanes) afforded the title compound as a colorless oil (838 mg, 2.73 mmol, 32% yield).

^{1}H NMR (400 MHz, CDCl$_3$): δ 9.75 (t, *J* = 1.8 Hz, 1H), 6.90 – 6.85 (m, 1H), 6.01 (ddd, *J* = 10.0, 2.5, 1.5 Hz, 1H), 5.30 – 5.25 (m, 1H), 4.62 – 4.55 (m, 2H), 2.54 – 2.39 (m, 4H), 2.36 – 2.27 (m, 1H), 1.98 – 1.84 (m, 2H), 1.73 (t, *J* = 1.2 Hz, 4H), 1.69 – 1.59 (m, 5H), 1.39 – 1.24 (m, 4H).

^{13}C NMR (100 MHz, CDCl$_3$): δ 202.8, 196.5, 171.7, 149.3, 139.5, 129.4, 118.4, 62.3, 57.1, 43.9, 33.6, 30.3, 29.6, 25.8, 24.4, 23.9, 21.9, 18.2.

IR (Neat Film, NaCl): 2934, 2864, 1733, 1717, 1684, 1456, 1220 cm^{-1}

HRMS (MM: FD+): *m/z* calc'd for $C_{18}H_{26}O_4$ [M]$^+$: 306.1831, found 306.1854.

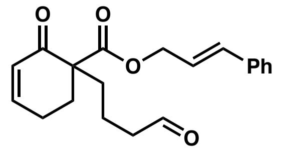

S2d

cinnamyl 2-oxo-1-(4-oxobutyl)cyclohex-3-ene-1-carboxylate (S2d)

Prepared from **S1b** and 4-bromo-1,1-diethoxybutane[14] following General Procedure C. Purification by flash column chromatography (10–25% EtOAc/hexanes) afforded the title compound as a colorless oil (0.607 g, 1.86 mmol, 49% yield).

^{1}H NMR (400 MHz, CDCl$_3$): δ 9.74 (t, *J* = 1.5 Hz, 1H), 7.38 – 7.35 (m, 2H), 7.35 – 7.30 (m, 2H), 7.28 – 7.24 (m, 1H), 6.91 (dddd, *J* = 10.1, 4.3, 3.1, 1.1 Hz, 1H), 6.63 (dt, *J* = 16.0, 1.4 Hz, 1H), 6.23 (dt, *J* = 15.8, 6.4 Hz, 1H), 6.05 (ddd, *J* = 10.1, 2.4, 1.7 Hz, 1H), 4.77 (dt, *J* = 6.5, 1.1 Hz, 2H), 2.57 – 2.45 (m, 4H), 2.42 – 2.32 (m, 1H), 2.05 – 1.98 (m, 1H), 1.93 (ddd, *J* = 13.2, 11.7, 5.1 Hz, 1H), 1.80 (ddd, *J* = 13.2, 11.1, 5.5 Hz, 1H), 1.73 – 1.63 (m, 2H).

^{13}C NMR (100 MHz, CDCl$_3$): δ 202.1, 196.0, 171.3, 149.7, 136.2, 134.7, 129.2, 128.8, 128.3, 126.8, 122.7, 66.0, 57.1, 44.1, 33.3, 30.1, 23.8, 17.5.

IR (Neat Film, NaCl): 2941, 1732, 1717, 1700, 1181, 734, 701 cm^{-1}

HRMS (MM: FD+): *m/z* calc'd for $C_{20}H_{22}O_4$ [M]$^+$: 326.1513, found 326.1510.

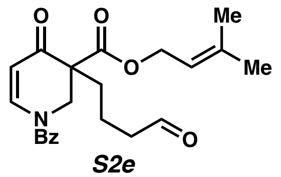

S2e

1-benzyl 3-(3-methylbut-2-en-1-yl) 4-oxo-3-(4-oxobutyl)-3,4-dihydropyridine-1,3(2*H*)-dicarboxylate (S2e)

Prepared from **S1g** and 4-bromo-1,1-diethoxybutane[14] following General Procedure C. Purification by flash column chromatography (15–30% EtOAc/hexanes) afforded the title compound as a colorless oil (603.9 mg, 1.46 mmol, 60% yield).

^{1}H NMR (400 MHz, CDCl$_3$): δ 9.73 (t, *J* = 1.4 Hz, 1H), 7.80 (s, 1H), 7.40 (d, *J* = 3.5 Hz, 5H), 5.27 (m, 4H), 4.61 (m, 3H), 3.78 (d, *J* = 13.6 Hz, 1H), 2.45 (tt, *J* = 6.8, 1.7 Hz, 2H), 2.03 – 1.90 (m, 1H), 1.72 (s, 3H), 1.67 (s, 3H), 1.70 – 1.60 (m, 1H).

^{13}C NMR (100 MHz, CDCl$_3$): δ 201.6, 190.5, 169.4, 142.7, 140.1, 135.0, 129.0, 128.9, 128.6, 118.0, 106.5, 69.4, 62.8, 55.4, 48.2, 43.9, 31.1, 25.8, 18.2, 17.2.

IR (Neat Film, NaCl): 2945, 2338, 1727, 1670, 1604, 1389, 1302, 1201, 932 cm^{-1}.

HRMS (MM: FD+): *m/z* calc'd for $C_{23}H_{27}NO_6$ [M]$^+$: 413.1838, found 413.1852.

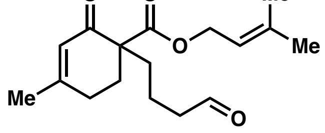

S2f

3-methylbut-2-en-1-yl 4-methyl-2-oxo-1-(4-oxobutyl)cyclohex-3-ene-1-carboxylate (S2f)

Prepared from **S1e** and 4-bromo-1,1-diethoxybutane[14] following General Procedure C. Purification by flash column chromatography (25–50% EtOAc/hexanes) afforded the title compound as a colorless oil (1141.4 mg, 3.90 mmol, 78% yield).

^{1}H NMR (400 MHz, CDCl$_3$): δ 9.74 (s, 1H), 5.87 (dt, *J* = 2.6, 1.2 Hz, 1H), 5.27 (tp, *J* = 7.1, 1.4 Hz, 1H), 4.58 (d, *J* = 7.2 Hz, 2H), 2.45 (ddd, *J* = 8.0, 4.8, 1.7 Hz, 4H), 2.29 – 2.18 (m, 1H), 1.92 (s, 3H), 2.00 – 1.84 (m, 2H), 1.72 (s, 3H), 1.67 (s, 3H), 1.79 – 1.54 (m, 3H).

^{13}C NMR (100 MHz, CDCl$_3$): δ 202.2, 195.9, 171.7, 161.6, 139.6, 126.0, 118.3, 62.3, 56.0, 44.2, 33.2, 30.0, 28.7, 25.8, 24.2, 18.2, 17.5.

IR (Neat Film, NaCl): 3426, 2936, 2730, 1725, 1672, 1637, 1440, 1380, 1348, 1311, 1272, 1233, 1214, 1177, 1104, 1050, 1016, 986, 939, 870, 842, 820, 776 cm^{-1}.

HRMS (MM: FD+): *m/z* calc'd for $C_{17}H_{24}O_4$ $[M]^+$: 292.1682, found 292.1669.

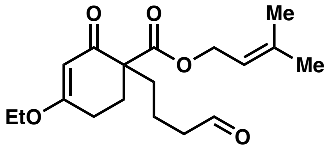

S2g

3-methylbut-2-en-1-yl 4-ethoxy-2-oxo-1-(4-oxobutyl)cyclohex-3-ene-1-carboxylate (S2g)

Prepared from **S1f** and 4-bromo-1,1-diethoxybutane[14] following General Procedure C. Purification by flash column chromatography (30–40% EtOAc/hexanes) afforded the title compound as a colorless oil (653.2 mg, 2.03 mmol, 21% yield).

^{1}H NMR (400 MHz, CDCl$_3$): δ 9.75 (t, *J* = 1.5 Hz, 1H), 5.34 (d, *J* = 1.1 Hz, 1H), 5.29 (tp, *J* = 7.2, 1.4 Hz, 1H), 4.66 – 4.53 (m, 2H), 3.86 (q, *J* = 7.18, 2H), 2.61 (dddd, *J* = 17.8, 10.3, 4.7, 1.3 Hz, 1H), 2.50 – 2.30 (m, 4H), 2.02 – 1.87 (m, 2H), 1.82 – 1.73 (m, 1H), 1.72 (s, 3H), 1.68 (s, 3H), 1.67 – 1.58 (m, 2H), 1.35 (t, *J* = 7.0 Hz, 3H).

^{13}C NMR (100 MHz, CDCl$_3$): δ 201.9, 195.5, 176.5, 171.5, 139.1, 118.1, 101.9, 64.3, 62.0, 55.7, 43.9, 33.1, 28.2, 26.3, 25.5, 17.9, 17.2, 13.9.

IR (Neat Film, NaCl): 2939, 2728, 1723, 1659, 1608, 1447, 1380, 1315, 1242, 1179, 1108, 1027, 942, 816, 769 cm^{-1}.

HRMS (MM: FD+): *m/z* calc'd for $C_{18}H_{26}O_5$ [M]$^+$: 322.1790, found 322.1775.

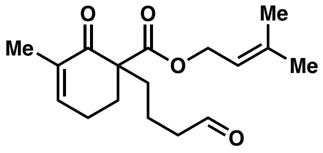

S2h

3-methylbut-2-en-1-yl 3-methyl-2-oxo-1-(4-oxobutyl)cyclohex-3-ene-1-carboxylate (S2h)
Prepared from **S1d** and 4-bromo-1,1-diethoxybutane[14] following General Procedure C. Purification by flash column chromatography (15–20% EtOAc/hexanes) afforded the title compound as a colorless oil (1.14 g, 3.90 mmol, 70% yield).

^{1}H NMR (400 MHz, CDCl$_3$): δ 9.75 (s, 1H), 6.64 – 6.57 (m, 1H), 5.27 (tt, *J* = 7.1, 1.4 Hz, 1H), 4.64 – 4.51 (m, 2H), 2.51 – 2.36 (m, 4H), 2.36 – 2.22 (m, 1H), 2.01 – 1.92 (m, 1H), 1.87 (m, 1H), 1.83-1.53 (m, 3H) 1.78 (s, 3H), 1.73 (s, 3H), 1.67 (d, *J* = 1.3 Hz, 3H).

^{13}C NMR (100 MHz, CDCl$_3$): δ 202.2, 196.9, 171.9, 143.9, 139.6, 135.3, 118.3, 62.2, 57.0, 44.2, 33.3, 30.7, 25.8, 23.5, 18.2, 17.7, 16.6.

IR (Neat Film, NaCl): 3500, 2925, 2333, 1725, 1681, 1449, 1361, 1182 cm^{-1}.

HRMS (MM: FD+): *m/z* calc'd for $C_{17}H_{24}O_4$ [M]$^+$: 292.1680, found 292.1669.

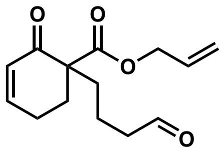

S2i

allyl 2-oxo-1-(4-oxobutyl)cyclohex-3-ene-1-carboxylate (S2i)

Prepared from allyl 2-oxocyclohex-3-ene-1-carboxylate [15] and 4-bromo-1,1-diethoxybutane[14] following General Procedure C. Purification by flash column chromatography (15–30% EtOAc/hexanes) afforded the title compound as a colorless oil (773.2 mg, 3.09 mmol, 21% yield).

^{1}H NMR (400 MHz, $CDCl_3$): δ 9.75 (t, *J* = 1.4 Hz, 1H), 6.91 (dddd, *J* = 10.1, 4.4, 3.1, 1.1 Hz, 1H), 6.03 (dt, *J* = 10.1, 2.0 Hz, 1H), 5.86 (ddt, *J* = 17.1, 10.2, 5.6 Hz, 1H), 5.28 (dq, *J* = 17.2, 1.6 Hz, 1H), 5.22 (dq, *J* = 10.4, 1.3 Hz, 1H), 4.60 (dq, *J* = 5.4, 1.6 Hz, 2H), 2.58 – 2.43 (m, 4H), 2.43 – 2.30 (m, 1H), 2.07 – 1.95 (m, 1H), 1.91 (ddd, *J* = 13.2, 11.6, 5.2 Hz, 1H), 1.79 (ddd, *J* = 13.2, 10.9, 5.6 Hz, 1H), 1.75 – 1.56 (m, 2H).

^{13}C NMR (100 MHz, $CDCl_3$): δ 202.1, 196.0, 171.2, 149.7, 131.7, 129.2, 118.7, 65.9, 57.1, 44.1, 33.1, 30.1, 23.7, 17.4.

IR (Neat Film, NaCl): 2947, 2732, 1726, 1680, 1238, 1184 cm^{-1}.

HRMS (MM: FD+): *m/z* calc'd for $C_{14}H_{19}O_4$ $[M]^+$: 251.1281, found 251.1278.

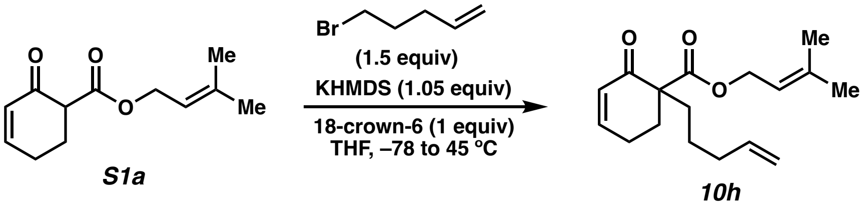

3-methylbut-2-en-1-yl 2-oxo-1-(pent-4-en-1-yl)cyclohex-3-ene-1-carboxylate (10h)

Prepared from **S1a** and 5-bromopent-1-ene following General Procedure C (without hydrolysis step). Purification by flash column chromatography (0-20% EtOAc/hexanes) afforded the title compound as a colorless oil (93.8 mg, 0.34 mmol, 23% yield).

^{1}H NMR (400 MHz, CDCl$_3$): δ 6.87 (dddd, *J* = 10.1, 4.8, 3.1, 1.1 Hz, 1H), 6.01 (ddd, *J* = 10.1, 2.5, 1.6 Hz, 1H), 5.78 (ddt, *J* = 16.9, 10.2, 6.6 Hz, 1H), 5.28 (tdq, *J* = 7.1, 2.9, 1.4 Hz, 1H), 5.00 (dq, *J* = 17.1, 1.6 Hz, 1H), 4.94 (ddt, *J* = 10.2, 2.2, 1.2 Hz, 1H), 4.59 (dd, *J* = 7.2, 3.7 Hz, 1H), 4.64 – 4.53 (m, 2H), 2.57 – 2.41 (m, 2H), 2.38 – 2.24 (m, 2H), 2.12 – 2.01 (m, 2H), 2.00 – 1.86 (m, 2H), 1.77 – 1.69 (m, 1H), 1.73 (s, 3H), 1.68 (s, 3H), 1.51 – 1.28 (m, 2H).

^{13}C NMR (100 MHz, CDCl$_3$): δ 196.5, 171.7, 149.3, 139.5, 138.4, 129.4, 118.4, 114.9, 62.2, 57.1, 34.2, 33.4, 30.3, 25.8, 24.0, 23.9, 18.2.

IR (Neat Film, NaCl): 2928, 1726, 1683, 1440, 1383, 1186, 912 cm^{-1}.

HRMS (MM: FD+): *m/z* calc'd for $C_{17}H_{24}O_3$ $[M]^+$: 276.1725, found 276.1718.

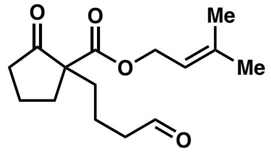

S7

3-methylbut-2-en-1-yl 2-oxo-1-(4-oxobutyl)cyclopentane-1-carboxylate (S7)

Prepared from 3-methylbut-2-en-1-yl 2-oxocyclopentane-1-carboxylate [16] and 4-bromo-1,1-diethoxybutane[14] following General Procedure C. Purification by flash column chromatography (20–25% EtOAc/hexanes) afforded the title compound as a colorless oil (1.86 g, 6.97 mmol, 87% yield).

^{1}H NMR (400 MHz, CDCl$_3$): δ 9.74 (t, *J* = 1.4 Hz, 1H), 5.29 (tp, *J* = 7.3, 1.4 Hz, 1H), 4.59 (d, *J* = 7.2 Hz, 2H), 2.58 – 2.47 (m, 1H), 2.44 (tt, *J* = 7.0, 1.5 Hz, 2H), 2.44 – 2.36 (m, 1H), 2.31 – 2.18 (m, 1H), 2.11 – 1.85 (m, 4H), 1.74 (s, 3H), 1.68 (s, 3H), 1.67 – 1.48 (m, 3H).

^{13}C NMR (100 MHz, CDCl$_3$): δ 214.8, 201.9, 171.0, 139.7, 118.2, 62.5, 60.4, 44.0, 38.0, 33.2, 33.0, 25.9, 19.8, 18.2, 17.6.

IR (Neat Film, NaCl): 3456, 2954, 2724, 1745, 1721, 1446, 1406, 1384, 1154, 953 cm^{-1}.

HRMS (MM: FD+): *m/z* calc'd for $C_{15}H_{22}O_4$ $[M]^+$: 266.1525, found 266.1513.

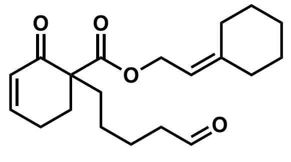

S2j

2-cyclohexylideneethyl 2-oxo-1-(5-oxopentyl)cyclohex-3-ene-1-carboxylate (S2j)

Prepared from **S1h** and 5-bromo-1,1-diethoxypentane[14] following General Procedure C. Purification by flash column chromatography (10–50% EtOAc/hexanes) afforded the title compound as a colorless oil (177 mg, 0.53 mmol, 25% yield).

^{1}H NMR (400 MHz, CDCl$_3$): δ 9.75 (t, *J* = 1.7 Hz, 1H), 6.93 – 6.83 (m, 1H), 6.02 (ddd, *J* = 10.1, 2.6, 1.6 Hz, 1H), 5.23 (tt, *J* = 7.2, 1.2 Hz, 1H), 4.60 (d, *J* = 7.2 Hz, 2H), 2.56 – 2.39 (m, 4H), 2.37 – 2.26 (m, 1H), 2.20 – 2.13 (m, 2H), 2.12 – 2.05 (m, 2H), 1.99 – 1.85 (m, 2H), 1.74 (ddd, *J* = 13.6, 11.7, 5.0 Hz, 1H), 1.64 (p, *J* = 7.5 Hz, 2H), 1.58 – 1.46 (m, 6H), 1.43 – 1.20 (m, 2H).

^{13}C NMR (100 MHz, CDCl$_3$): δ 202.6, 196.4, 171.6, 149.3, 147.7, 129.4, 114.8, 61.5, 57.0, 43.7, 37.1, 33.6, 30.4, 29.2, 28.5, 27.9, 26.7, 24.3, 23.9, 22.5.

IR (Neat Film, NaCl): 2929, 2858, 1731, 1446, 1170, 938 cm^{-1}.

HRMS (MM: FD+): *m/z* calc'd for $C_{20}H_{28}O_4$ $[M]^+$: 332.1988, found 332.1991.

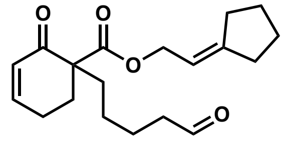

S2k

2-cyclopentylideneethyl 2-oxo-1-(5-oxopentyl)cyclohex-3-ene-1-carboxylate (S2k)

Prepared from **S1i** and 5-bromo-1,1-diethoxypentane[14] following General Procedure C. Purification by flash column chromatography (10–60% EtOAc/hexanes) afforded the title compound as a colorless oil (1.40 g, 4.40 mmol, 39% yield).

^{1}H NMR (400 MHz, CDCl$_3$): δ 9.75 (t, *J* = 1.7 Hz, 3H), 6.92 – 6.83 (m, 1H), 6.02 (ddd, *J* = 10.1, 2.6, 1.6 Hz, 1H), 5.41 – 5.32 (m, 1H), 4.64 – 4.50 (m, 2H), 2.56 – 2.39 (m, 4H), 2.37 – 2.20 (m, 5H), 1.99 – 1.83 (m, 2H), 1.80 – 1.57 (m, 7H), 1.45 – 1.22 (m, 2H).

^{13}C NMR (100 MHz, CDCl$_3$): δ 202.6, 196.4, 171.7, 151.1, 149.4, 129.4, 113.8, 63.8, 57.0, 43.7, 33.9, 33.6, 30.4, 29.0, 26.4, 26.2, 24.3, 23.9, 22.5.

IR (Neat Film, NaCl): 2947, 2725, 1729, 1697, 1456, 1356, 1215 cm^{-1}.

HRMS (MM: FD+): *m/z* calc'd for $C_{19}H_{26}O_4$ $[M]^+$: 318.1831, found 318.1809.

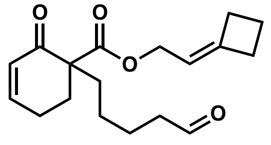

S2l

2-cyclobutylideneethyl 2-oxo-1-(5-oxopentyl)cyclohex-3-ene-1-carboxylate (S2l)

Prepared from **S1j** and 5-bromo-1,1-diethoxypentane[14] following General Procedure C. Purification by flash column chromatography (10–60% EtOAc/hexanes) afforded the title compound as a colorless oil (649 mg, 2.13 mmol, 29% yield).

^{1}H NMR (400 MHz, CDCl$_3$): δ 9.75 (t, *J* = 1.7 Hz, 1H), 6.94 – 6.83 (m, 1H), 6.02 (ddd, *J* = 10.1, 2.6, 1.5 Hz, 1H), 5.20 (tp, *J* = 7.1, 2.3 Hz, 1H), 4.46 (ddt, *J* = 7.4, 2.3, 1.1 Hz, 2H), 2.69 (dt, *J* = 16.0, 8.4 Hz, 4H), 2.56 – 2.40 (m, 4H), 2.37 – 2.26 (m, 1H), 2.03 – 1.84 (m, 4H), 1.75 (ddd, *J* = 13.6, 11.6, 5.1 Hz, 1H), 1.64 (p, *J* = 7.5 Hz, 2H), 1.42 – 1.26 (m, 2H).

^{13}C NMR (100 MHz, CDCl$_3$): δ 202.6, 196.4, 171.6, 149.4, 148.9, 129.4, 114.0, 62.3, 57.0, 43.7, 33.6, 31.2, 30.4, 29.6, 24.3, 23.9, 22.5, 17.1.

IR (Neat Film, NaCl): 2941, 1726, 1681, 1446, 1387, 1240, 1171, 1103 cm^{-1}.

HRMS (MM: FD+): *m/z* calc'd for $C_{18}H_{24}O_4$ $[M]^+$: 304.1675, found 304.1677.

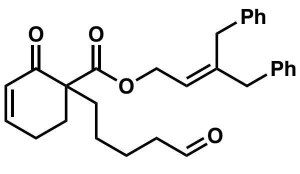

S2m

3-benzyl-4-phenylbut-2-en-1-yl 2-oxo-1-(5-oxopentyl)cyclohex-3-ene-1-carboxylate (S2m)

Prepared from **S1k** and 5-bromo-1,1-diethoxypentane[14] following General Procedure C. Purification by flash column chromatography (10–60% Et_2O/hexanes) afforded the title compound as a colorless oil (425 mg, 0.955 mmol, 18% yield).

^{1}H NMR (400 MHz, $CDCl_3$): δ 9.73 (t, *J* = 1.7 Hz, 1H), 7.32 – 7.27 (m, 4H), 7.21 (tt, *J* = 7.5, 2.3 Hz, 2H), 7.15 – 7.03 (m, 4H), 6.90 – 6.82 (m, 1H), 6.02 (ddd, *J* = 10.2, 2.5, 1.6 Hz, 1H), 5.51 (d, *J* = 7.4 Hz, 1H), 4.83 – 4.70 (m, 2H), 3.36 (s, 2H), 3.24 (s, 2H), 2.54 – 2.24 (m, 5H), 2.00 – 1.86 (m, 2H), 1.77 (ddd, *J* = 13.6, 11.6, 5.1 Hz, 1H), 1.63 (p, *J* = 7.5 Hz, 2H), 1.44 – 1.22 (m, 2H).

^{13}C NMR (100 MHz, $CDCl_3$): δ 202.5, 196.2, 171.6, 149.4, 144.8, 139.0, 138.8, 129.4, 129.3, 128.8, 128.7, 128.5, 126.5, 126.4, 121.7, 62.0, 57.0, 43.7, 42.9, 35.8, 33.6, 30.4, 24.3, 23.9, 22.5.

IR (Neat Film, NaCl): 2923, 1723, 1684, 1493, 1451, 1386, 1231 cm^{-1}.

HRMS (MM: FD+): *m/z* calc'd for $C_{29}H_{32}O_4$ $[M]^+$: 444.2301, found 444.2300.

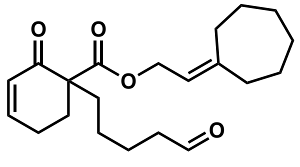

S2n

2-cycloheptylideneethyl 2-oxo-1-(5-oxopentyl)cyclohex-3-ene-1-carboxylate (S2n)

Prepared from **S1l** and 5-bromo-1,1-diethoxypentane[14] following General Procedure C. Purification by flash column chromatography (10–70% Et_2O/hexanes) afforded the title compound as a colorless oil (349 mg, 1.01 mmol, 15% yield).

^{1}H NMR (400 MHz, $CDCl_3$): δ 9.75 (t, *J* = 1.7 Hz, 1H), 6.91 – 6.84 (m, 1H), 6.02 (ddd, *J* = 10.1, 2.6, 1.6 Hz, 1H), 5.28 (tt, *J* = 7.1, 1.3 Hz, 1H), 4.60 (d, *J* = 7.1 Hz, 2H), 2.56 – 2.40 (m, 4H), 2.38 – 2.20 (m, 5H), 1.98 – 1.87 (m, 2H), 1.76 (ddd, *J* = 13.7, 12.0, 4.8 Hz, 1H), 1.65 (p, *J* = 7.5 Hz, 2H), 1.57 (q, *J* = 5.4 Hz, 4H), 1.50 (dt, *J* = 5.2, 2.4 Hz, 4H), 1.42 – 1.27 (m, 2H).

^{13}C NMR (100 MHz, $CDCl_3$): δ 202.6, 196.4, 171.7, 149.4, 149.0, 129.4, 118.4, 62.1, 57.0, 43.7, 37.7, 33.6, 30.4, 30.2, 29.8, 29.1, 28.9, 27.3, 24.3, 23.9, 22.5.

IR (Neat Film, NaCl): 2923, 2854, 1737, 1681, 1443, 1385, 1235, 1172 cm^{-1}.

HRMS (MM: FD+): *m/z* calc'd for $C_{21}H_{30}O_4$ $[M]^+$: 346.2144, found 346.2139.

β-Ketoesters Synthesis

General Procedure D: Prenyl β-ketoesters Synthesis through Acylation

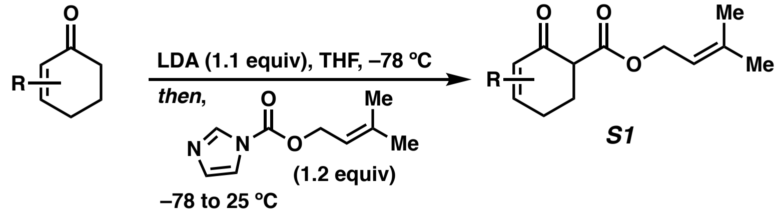

A flame dried round bottom flask was charged with iPr_2NH (1.1 equiv) and THF (1.75 M). The solution was cooled to 0 ºC and *n*-BuLi (2.5 M in hexanes, 1.05 equiv) was added dropwise. The resultant solution was stirred for 30 min at 0 ºC. The corresponding cyclohexenone (1.0 equiv) in THF (1.25 M) was added dropwise and stirring was continued at 0 ºC for 30 minutes. The solution was cooled to –78 ºC, and the appropriate N-acyl imidazole (1.2 equiv) in THF (3.25 M) was added dropwise. After 2 h, the reaction was gradually warmed to 23 ºC and diluted with 2 M aqueous HCl until reaching a pH < 7. The reaction mixture was extracted three times with EtOAc. The combined organic layers were washed with brine, dried over Na_2SO_4, filtered, and concentrated under reduced pressure. The crude product was purified by flash silica gel column chromatography to afford the corresponding acylated enone.

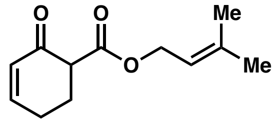

S1a

3-methylbut-2-en-1-yl 2-oxocyclohex-3-ene-1-carboxylate (S1a)

Prepared from 2-cyclohexen-1-one and 3-methylbut-2-en-1-yl 1H-imidazole-1-carboxylate [17] following General Procedure D. Purification by flash column chromatography (25% EtOAc/hexanes) afforded the title compound as a colorless oil (6.65 g, 31.9 mmol, 41% yield).

^{1}H NMR (400 MHz, $CDCl_3$): δ 7.02 – 6.97 (m, 1H), 6.07 (dt, *J* = 10.2, 2.0 Hz, 1H), 5.37 – 5.32 (m, 1H), 4.65 (d, *J* = 7.1 Hz, 2H), 3.42 – 3.39 (m, 1H), 2.55 – 2.45 (m, 1H), 2.44 – 2.34 (m, 2H), 2.26 – 2.18 (m, 1H), 1.75 (s, 3H), 1.71 (s, 3H).

^{13}C NMR (100 MHz, $CDCl_3$): δ 194.1, 170.2, 150.7, 139.6, 129.3, 118.4, 62.3, 53.6, 25.9, 25.8, 24.5, 18.2.

IR (Neat Film, NaCl): 3033, 2934, 1736, 1682, 1447, 1387, 1302, 1233, 1159, 1123 cm^{-1}

HRMS (MM: FD+): *m/z* calc'd for $C_{12}H_{16}O_3$ $[M]^+$: 208.1099, found 208.1090.

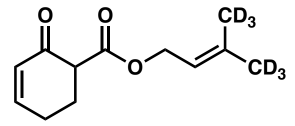

D-S1a

3-(methyl-*d*$_3$)but-2-en-1-yl-4,4,4-*d*$_3$ 2-oxocyclohex-3-ene-1-carboxylate (S1a-*D*)

Prepared from 2-cyclohexen-1-one and 3-(methyl-*d*$_3$)but-2-en-1-yl-4,4,4-*d*$_3$ 1*H*-imidazole-1-carboxylate[18] following General Procedure D. Purification by flash column chromatography (25% EtOAc/hexanes) afforded the title compound as a colorless oil (1.00 g, 4.67 mmol, 37% yield). *Note that 1.0 equiv of the N-acyl imidazole can be employed.*

^{1}H NMR (400 MHz, $CDCl_3$): δ 6.99 (dt, *J* = 10.0, 3.7 Hz, 1H), 6.06 (dt, *J* = 10.2, 2.1 Hz, 1H), 5.34 (t, *J* = 7.2 Hz, 1H), 4.65 (d, *J* = 7.2 Hz, 2H), 3.40 (dd, *J* = 9.7, 5.0 Hz, 1H), 2.54 – 2.44 (m, 1H), 2.44 – 2.32 (m, 2H), 2.22 (ddt, *J* = 13.7, 8.8, 3.0 Hz, 1H).

^{13}C NMR (100 MHz, $CDCl_3$): δ 194.1, 170.2, 150.7, 139.4, 129.3, 118.4, 62.3, 53.6, 25.8, 24.5.

^{2}H NMR (61 MHz, $CHCl_3$): δ 1.72, 1.67.

IR (Neat Film, NaCl): 2942, 1736, 1681, 1388, 1164 cm^{-1}

HRMS (MM: FD+): *m/z* calc'd for $C_{12}H_{10}D_6O_3$ $[M]^+$: 214.1476, found 214.1476.

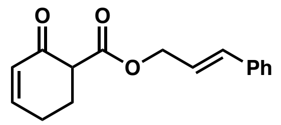

S1b

cinnamyl 2-oxocyclohex-3-ene-1-carboxylate (S1b)

Prepared from 2-cyclohexen-1-one and cinnamyl 1*H*-imidazole-1-carboxylate[17] following General Procedure D. Purification by flash column chromatography (15–25% EtOAc/hexanes) afforded the title compound as a colorless oil (0.98 g, 3.82 mmol, 39% yield).

^{1}H NMR (400 MHz, CDCl$_3$): δ 7.40 – 7.37 (m, 2H), 7.34 – 7.30 (m, 2H), 7.28 – 7.24 (m, 1H), 7.01 (dt, *J* = 10.3, 3.8 Hz, 1H), 6.67 (dt, *J* = 15.9, 1.3 Hz, 1H), 6.29 (dt, *J* = 15.9, 6.4 Hz, 1H), 6.09 (dt, *J* = 10.2, 2.0 Hz, 1H), 4.83 (d, *J* = 6.5 Hz, 2H), 3.47 (dd, *J* = 10.2, 4.9 Hz, 1H), 2.57 – 2.47 (m, 1H), 2.47 – 2.34 (m, 2H), 2.28 – 2.21 (m, 1H).

^{13}C NMR (100 MHz, CDCl$_3$): δ 193.9, 169.9, 150.8, 136.3, 134.6, 129.3, 128.7, 128.2, 126.8, 122.9, 65.9, 53.6, 25.8, 24.5.

IR (Neat Film, NaCl): 3024, 2940, 1734, 1676, 1304, 1223, 1157, 1123, 969 cm^{-1}

HRMS (MM: FD+): *m/z* calc'd for $C_{16}H_{16}NaO_3$ $[M+Na]^+$: 279.0997, found 279.0983.

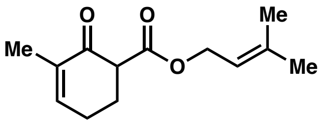

S1d

3-methylbut-2-en-1-yl 3-methyl-2-oxocyclohex-3-ene-1-carboxylate (S1d)

Prepared from 2-methylcyclohex-2-en-1-one [19] and 3-methylbut-2-en-1-yl 1H-imidazole-1-carboxylate[17] following General Procedure D. Purification by flash column chromatography (10–20% EtOAc/hexanes) afforded the title compound as a colorless oil (1.09 g, 4.90 mmol, 23% yield).

^{1}H NMR (400 MHz, CDCl$_3$): δ 6.78 – 6.69 (m, 1H), 5.35 (tdq, *J* = 7.2, 2.9, 1.5 Hz, 1H), 4.65 (d, *J* = 7.2 Hz, 2H), 3.42 – 3.36 (m, 1H), 2.48 – 2.11 (m, 5H), 1.79 (d, *J* = 1.6 Hz, 3H), 1.75 (s, 3H), 1.70 (s, 3H).

^{13}C NMR (100 MHz, CDCl$_3$): δ 194.7, 170.6, 145.5, 139.5, 135.4, 118.5, 62.2, 53.8, 26.3, 25.9, 24.6, 18.2, 16.2.

IR (Neat Film, NaCl): 2925, 1736, 1676, 1449, 1381, 1249, 1151 cm^{-1}.

HRMS (MM: FD+): *m/z* calc'd for $C_{13}H_{18}O_3$ $[M]^+$: 222.1255, found 222.1251.

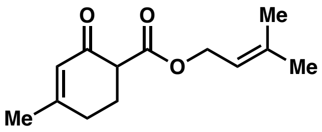

S1e

3-methylbut-2-en-1-yl 4-methyl-2-oxocyclohex-3-ene-1-carboxylate (S1e)

Prepared from 3-methylcyclohex-2-en-1-one and 3-methylbut-2-en-1-yl 1H-imidazole-1-carboxylate[17] following General Procedure D. Purification by flash column chromatography (10–20% EtOAc/hexanes) afforded the title compound as a colorless oil (2.95 g, 13.3 mmol, 17% yield).

^{1}H NMR (400 MHz, CDCl$_3$): δ 5.91 (h, *J* = 1.4 Hz, 1H), 5.35 (tp, *J* = 7.1, 1.6 Hz, 1H), 4.65 (d, *J* = 7.2 Hz, 2H), 3.37 – 3.27 (m, 1H), 2.48 – 2.24 (m, 3H), 2.21 – 2.15 (m, 1H), 1.97 (s, 3H), 1.75 (d, *J* = 1.3 Hz, 3H), 1.70 (s, 3H).

^{13}C NMR (100 MHz, CDCl$_3$): δ 193.9, 170.5, 163.0, 139.5, 126.0, 118.5, 62.3, 52.6, 29.5, 25.9, 25.7, 24.5, 18.2.

IR (Neat Film, NaCl): 2938, 1732, 1668, 1632, 1434, 1378, 1357, 1302, 1246, 1216, 1170, 1152, 1018 cm^{-1}.

HRMS (MM: FD+): *m/z* calc'd for $C_{13}H_{18}O_3$ $[M]^+$: 222.1257, found 222.1251.

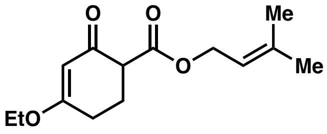

S1f

3-methylbut-2-en-1-yl 4-ethoxy-2-oxocyclohex-3-ene-1-carboxylate (S1f)

Prepared from 3-ethoxycyclohex-2-en-1-one and 3-methylbut-2-en-1-yl 1H-imidazole-1-carboxylate[17] following General Procedure D. Purification by flash column chromatography (25–30% EtOAc/hexanes) afforded the title compound as a colorless oil (2.84 g, 9.51 mmol, 19% yield).

^{1}H NMR (400 MHz, $CDCl_3$): δ 5.38 (s, 1H), 5.37 – 5.32 (m, 1H), 4.72 – 4.59 (m, 2H), 3.91 (qd, *J* = 7.0, 2.3 Hz, 2H), 3.36 – 3.27 (m, 1H), 2.56 (ddd, *J* = 16.6, 6.2, 4.5 Hz, 1H), 2.47 – 2.27 (m, 2H), 2.24 – 2.08 (m, 1H), 1.75 (s, 3H), 1.70 (s, 3H), 1.36 (t, *J* = 7.0 Hz, 3H).

^{13}C NMR (100 MHz, $CDCl_3$): δ 194.0, 177.7, 170.6, 139.4, 118.5, 102.3, 64.6, 62.3, 52.5, 27.5, 25.9, 24.3, 18.2, 14.2.

IR (Neat Film, NaCl): 2980, 2357, 1730, 1648, 1605, 1380, 1192, 1026, 668 cm^{-1}.

HRMS (MM: FD+): *m/z* calc'd for $C_{14}H_{20}O_4$ $[M]^+$: 252.1363, found 252.1356.

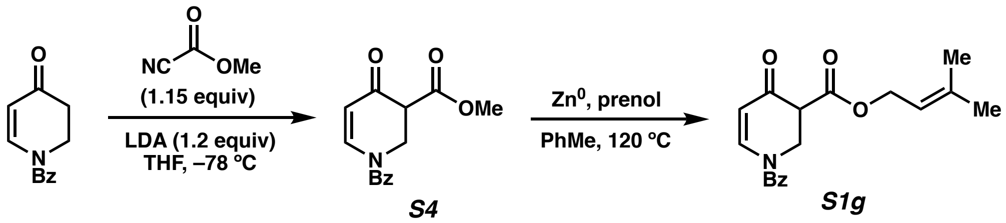

1-benzyl 3-methyl 4-oxo-3,4-dihydropyridine-1,3(2*H*)-dicarboxylate (S4)

A flame dried round bottom flask was charged with *i*-Pr_2NH (2.52 mL, 18.0 mmol, 1.2 equiv) and THF (167 mL, 0.1 M). The solution was cooled to –78 ºC and *n*-BuLi (7.20 mL, 18.0 mmol, 1.2 equiv) was added dropwise. The resultant solution was slowly warmed to 0 ºC over 1 h and then cooled to – 78 ºC. The LDA solution was added dropwise to a solution of 1-benzoyl-2,3-dihydropyridin-4(1*H*)-one[20] (3.47 g, 15.0 mmol, 1.0 equiv) in THF (239 mL, 0.06 M) at –78 ºC. The resultant solution was stirred for 1 h. Then methyl cyanoformate (1.37 mL, 17.25 mmol, 1.15 equiv) was added dropwise. Upon complete consumption of starting material (as determined by TLC), the reaction was diluted with a saturated solution of NH_4Cl and the product was extracted with EtOAc (3 x 200 mL). The combined organic layers were dried over Na_2SO_4, filtered, and

concentrated under reduced pressure. The crude product was purified by column chromatography (SiO_2, 20–30% EtOAc/Hexanes) to afford acylated enone **S4** (1.17 g, 4.05 mmol, 27% yield).

^{1}H NMR (400 MHz, $CDCl_3$): δ 7.88 (s, 1H), 7.39 (d, *J* = 2.3 Hz, 5H), 5.40 (s, 1H), 5.28 (s, 1H), 4.39 (dd, *J* = 13.6, 8.9 Hz, 1H), 4.18 (dd, *J* = 13.6, 5.4 Hz, 1H), 3.76 (s, 3H), 3.51 (dd, *J* = 8.9, 5.4 Hz, 1H).

^{13}C NMR (100 MHz, $CDCl_3$): δ 187.8, 168.2, 143.6, 134.8, 129.1, 128.9, 128.8, 128.7, 106.8, 69.6, 52.9, 50.6, 44.4.

IR (Neat Film, NaCl): 2952, 2332, 1734, 1670, 1601, 1388, 1293, 1213 cm^{-1}.

HRMS (MM: FD+): *m/z* calc'd for $C_{15}H_{15}NO_5$ $[M]^+$: 289.0950, found 289.0948.

1-benzyl 3-(3-methylbut-2-en-1-yl) 4-oxo-3,4-dihydropyridine-1,3(2*H*)-dicarboxylate (S1g)

A flame dried round bottom flask equipped with a reflux condenser was charged with Zn^0 dust (51.5 mg, 0.787 mmol, 0.2 equiv), acylated enone **S4** (1.14 g, 3.40 mmol, 1.0 equiv), and toluene (19.7 mL, 0.2 M). To the stirred solution, prenyl alcohol was added neat (2.00 mL, 19.68 mmol, 5.0 equiv). The resultant solution was heated to reflux for 3 days. The solution was cooled to 23 ºC, filtered through a celite plug and eluted with CH_2Cl_2, and concentrated under reduced pressure. The crude product was purified by column chromatography (SiO_2, 15–25% EtOAc/Hexanes) to afford acylated enone **S1g** (883 mg, 2.57 mmol, 65% yield).

^{1}H NMR (400 MHz, $CDCl_3$): δ 7.86 (s, 1H), 7.39 (m, 5H), 5.39 (s, 1H), 5.32 (tp, *J* = 7.3, 1.4 Hz, 1H), 5.27 (s, 2H), 4.65 (dd, *J* = 7.3, 2.9 Hz, 2H), 4.38 (dd, *J* = 13.6, 8.9 Hz, 1H), 4.17 (dd, *J* = 13.5, 5.4 Hz, 1H), 3.48 (dd, *J* = 9.1, 5.3 Hz, 1H), 1.74 (s, 3H), 1.69 (s, 3H).

^{13}C NMR (100 MHz, $CDCl_3$): δ 187.9, 167.8, 143.5, 140.1, 134.9, 129.0, 128.9, 128.8, 128.7, 118.0, 106.9, 69.5, 62.8, 50.8, 44.5, 25.9, 18.2.

IR (Neat Film, NaCl): 2965, 1727, 1676, 1599, 1388, 1293, 1209, 940 cm^{-1}.

HRMS (MM: FD+): *m/z* calc'd for $C_{19}H_{21}HO_5$ $[M]^+$: 343.1420, found 343.1420.

General Procedure E: Substituted β-Ketoesters Synthesis through Acylation[17]

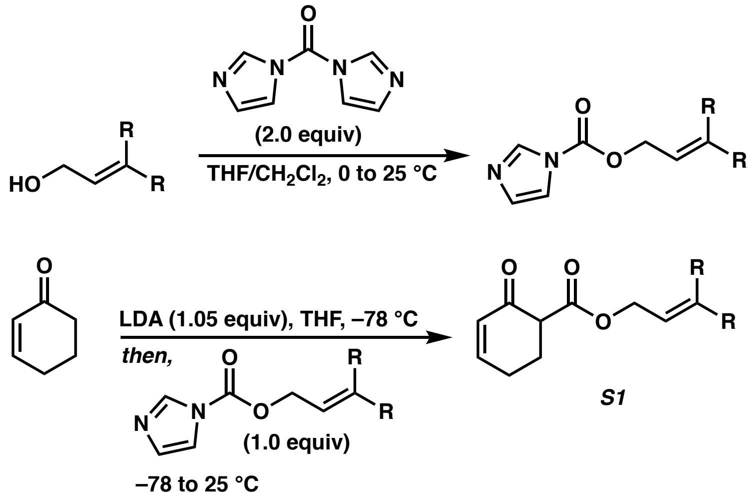

To a solution of di(1*H*-imidazol-1-yl)methanone (2.0 equiv) in THF (2.0 M) at 0 °C was added dropwise a solution of the corresponding alcohol (1.0 equiv) in CH_2Cl_2 (1.0 M). After 3 h, the reaction mixture was gradually warmed to 25 °C. Upon consumption of starting material (as determined by TLC), the reaction mixture was concentrated under reduced pressure then filtered through a silica plug and eluted with 50% EtOAc/Hexanes. The resulting solution was concentrated under reduced pressure.

A flame dried round bottom flask was charged with iPr_2NH (1.1 equiv) and THF (1.75 M). The solution was cooled to 0 ºC and *n*-BuLi (2.5 M in hexanes, 1.05 equiv) was added dropwise. The resultant solution was stirred for 30 min at 0 ºC. 2-cyclohexen-1-one (1.0 equiv) in THF (1.25 M) was added dropwise and stirring was continued at 0 ºC for 30 minutes. The solution was cooled to –78 ºC, and the corresponding crude 1*H*-imidazole-1-carboxylate (1.2 equiv) in THF (3.25 M) was added dropwise. After 2 h, the reaction was gradually warmed to 23 ºC and diluted with 2 M aqueous HCl until reaching a pH < 7. The reaction mixture was extracted three times with EtOAc. The combined organic layers were washed with brine, dried over Na_2SO_4, filtered, and concentrated under reduced pressure. The crude product was purified by column chromatography to afford the corresponding acylated enone.

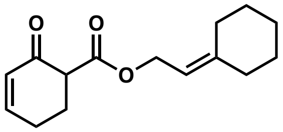

S1h

2-cyclohexylideneethyl 2-oxocyclohex-3-ene-1-carboxylate (S1h)

Prepared from 2-cyclohexen-1-one and 2-cyclohexylideneethan-1-ol [21] following General Procedure E, with the modification of 1.5 equiv of di(1*H*-imidazol-1-yl)methanone and 1.2 equiv of 2-cyclohexylideneethyl 1*H*-imidazole-1-carboxylate being used. Purification by flash column chromatography (5–30% EtOAc/hexanes) afforded the title compound as a colorless oil (535 mg, 2.15 mmol, 28% yield).

^{1}H NMR (400 MHz, $CDCl_3$): δ7.04 – 6.95 (m, 1H), 6.07 (dt, J = 10.1, 2.0 Hz, 1H), 5.29 (tt, J = 7.2, 1.2 Hz, 1H), 4.67 (d, J = 7.2 Hz, 2H), 3.45 – 3.36 (m, 1H), 2.56 – 2.30 (m, 3H), 2.27 – 2.15 (m, 3H), 2.14 – 2.08 (m, 2H), 1.60 – 1.48 (m, 6H).

^{13}C NMR (100 MHz, $CDCl_3$): δ 194.1, 170.2, 150.6, 147.5, 129.3, 115.0, 61.5, 53.6, 37.1, 29.2, 28.5, 27.9, 26.7, 25.8, 24.5.

IR (Neat Film, NaCl): 2930, 2852, 1735, 1683, 1447, 1388, 1298, 1169, 1122 cm^{-1}.

HRMS (MM: FD+): *m/z* calc'd for $C_{15}H_{20}O_3$ $[M]^+$: 248.1412, found 248.1418.

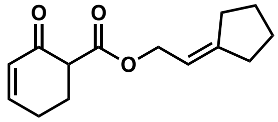

S1i

2-cyclopentylideneethyl 2-oxocyclohex-3-ene-1-carboxylate (S1i)

Prepared from 2-cyclohexen-1-one and 2-cyclopentylideneethan-1-ol[21] following General Procedure E. Purification by flash column chromatography (5–50% EtOAc/hexanes) afforded the title compound as a colorless oil (2.69 g, 11.46 mmol, 31.9% yield).

^{1}H NMR (400 MHz, CDCl$_3$): δ 6.99 (dt, *J* = 10.1, 3.8 Hz, 1H), 6.07 (dt, *J* = 10.2, 2.0 Hz, 1H), 5.50 – 5.40 (m, 1H), 4.64 (dt, *J* = 7.2, 1.1 Hz, 2H), 3.45 – 3.37 (m, 1H), 2.57 – 2.15 (m, 8H), 1.75 – 1.58 (m, 4H).

^{13}C NMR (100 MHz, CDCl$_3$): δ 194.1, 170.2, 151.2, 150.7, 129.3, 113.8, 63.8, 53.6, 34.0, 29.0, 26.4, 26.2, 25.8, 24.5.

IR (Neat Film, NaCl): 2946, 2869, 1782, 1681, 1455, 1387, 1304, 1224, 1156 cm^{-1}.

HRMS (MM: FD+): *m/z* calc'd for $C_{14}H_{18}O_3$ [M]$^+$: 234.1256, found 234.1255.

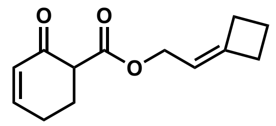

S1j

2-cyclobutylideneethyl 2-oxocyclohex-3-ene-1-carboxylate (S1j)

Prepared from 2-cyclohexen-1-one and 2-cyclobutylideneethan-1-ol [22] following General Procedure E. Purification by flash column chromatography (5–50% EtOAc/hexanes) afforded the title compound as a colorless oil (2.02 g, 9.19 mmol, 31.7% yield).

^{1}H NMR (400 MHz, CDCl$_3$): δ 7.00 (dt, *J* = 10.1, 3.7 Hz, 1H), 6.07 (dt, *J* = 10.2, 2.0 Hz, 1H), 5.27 (tp, *J* = 6.9, 2.2 Hz, 1H), 4.53 (d, *J* = 7.2 Hz, 2H), 3.45 – 3.36 (m, 1H), 2.80 – 2.65 (m, 4H), 2.56 – 2.31 (m, 3H), 2.28 – 2.15 (m, 1H), 1.98 (p, *J* = 8.0 Hz, 2H).

^{13}C NMR (100 MHz, CDCl$_3$): δ 194.1, 170.1, 150.7, 148.9, 129.3, 114.1, 62.3, 53.6, 31.2, 29.6, 25.8, 24.5, 17.1.

IR (Neat Film, NaCl): 2947, 1737, 1681, 1457, 1397, 1301, 1229, 1163, 1123 cm^{-1}.

HRMS (MM: FD+): *m/z* calc'd for $C_{13}H_{16}O_3$ [M]$^+$: 220.1099, found 220.1093.

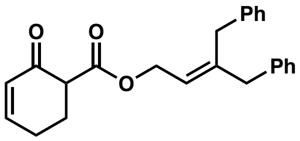

S1k

3-benzyl-4-phenylbut-2-en-1-yl 2-oxocyclohex-3-ene-1-carboxylate (S1k)

Prepared from 2-cyclohexen-1-one and 3-benzyl-4-phenylbut-2-en-1-ol [23] following General Procedure E. Purification by flash column chromatography (5–50% EtOAc/hexanes) afforded the title compound as a colorless oil (1.96 g, 5.43 mmol, 21.3% yield).

^{1}H NMR (400 MHz, CDCl$_3$): δ 7.33 – 7.25 (m, 4H), 7.24 – 7.18 (m, 2H), 7.17 – 7.07 (m, 4H), 7.03 – 6.97 (m, 1H), 6.08 (dt, *J* = 10.1, 2.0 Hz, 1H), 5.59 (tt, *J* = 7.1, 1.1 Hz, 1H), 4.86 – 4.80 (m, 2H), 3.48 – 3.35 (m, 3H), 3.26 (s, 2H), 2.55 – 2.32 (m, 3H), 2.30 – 2.15 (m, 1H).

^{13}C NMR (100 MHz, CDCl$_3$): δ 193.9, 170.1, 150.7, 144.4, 139.0, 138.9, 129.3, 129.3, 128.9, 128.7, 128.5, 126.5, 126.4, 121.9, 62.0, 53.6, 42.9, 35.9, 25.8, 24.5.

IR (Neat Film, NaCl): 3026, 2927, 1738, 1681, 1493, 1388, 1304, 1230, 1157, 1123 cm^{-1}.

HRMS (MM: FD+): *m/z* calc'd for $C_{24}H_{24}O_3$ $[M]^+$: 360.1725, found 360.17302.

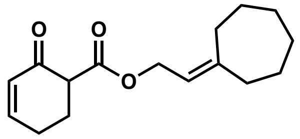

S1l

2-cycloheptylideneethyl 2-oxocyclohex-3-ene-1-carboxylate (S1l)

Prepared from 2-cyclohexen-1-one and 2-cycloheptylideneethan-1-ol [24] following General Procedure E. Purification by flash column chromatography (5–50% EtOAc/hexanes) afforded the title compound as a colorless oil (1.73 g, 5.43 mmol, 28.1% yield).

^{1}H NMR (400 MHz, CDCl$_3$): δ 6.99 (dt, *J* = 10.0, 3.8 Hz, 1H), 6.07 (dt, *J* = 10.1, 2.0 Hz, 1H), 5.34 (tt, *J* = 7.1, 1.3 Hz, 1H), 4.66 (d, *J* = 7.0 Hz, 2H), 3.45 – 3.37 (m, 1H), 2.57 – 2.15 (m, 8H), 1.63 – 1.44 (m, 8H).

^{13}C NMR (100 MHz, CDCl$_3$): δ 194.1, 170.2, 150.6, 148.8, 129.3, 118.5, 62.1, 53.6, 37.8, 30.2, 29.9, 29.1, 28.8, 27.3, 25.8, 24.5.

IR (Neat Film, NaCl): 2923, 2853, 1736, 1681, 1442, 1388, 1300, 1231, 1155, 1122, 1076 cm^{-1}.

HRMS (MM: FD+): *m/z* calc'd for $C_{26}H_{22}O_3$ $[M]^+$: 262.1569, found 262.1577.

Protonated Enone Byproducts

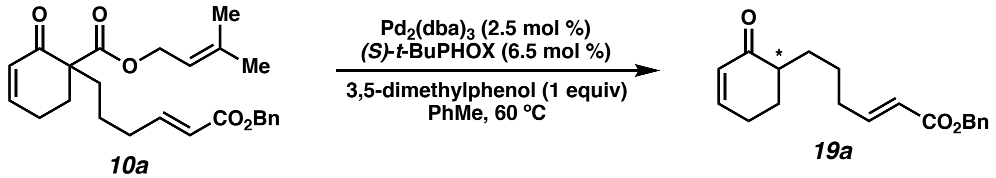

10a **19a**

benzyl (*R*,*E*)-6-(2-oxocyclohex-3-en-1-yl)hex-2-enoate (19a)

In a nitrogen filled glovebox, an oven-dried 20 mL vial was charged with a stir bar, $Pd_2(dba)_3$ (0.46 mg, 0.50 μmol, 2.5 mol %), *(S)-t*-BuPHOX (0.50 mg, 1.3 μmol, 6.5 mol %), and toluene (0.5 mL). The catalyst solution was stirred at 23 ºC for 20 min. A solution of substrate **10a** (8.2 mg, 0.020 mmol, 1 equiv) and 3,5-dimethylphenol (2.4 mg, 0.020 mmol, 1 equiv) in toluene (0.5 mL) was added to the vial. The resultant solution was then heated to 60 ºC for 14 h. The solution was then cooled to 23 ºC and concentrated under reduced pressure. NMR analysis of the crude reaction mixture affords an NMR yield of 100% (with respect to 1,3,5-trimethoxybenzene as an internal standard). The sample was purified by preparatory TLC (25% EtOAc/hexanes) to afford **19a** (71% ee).

^{1}H NMR (400 MHz, $CDCl_3$): δ 7.39 – 7.29 (m, 5H), 7.01 (dt, J = 15.7, 6.9 Hz, 1H), 6.92 (dddd, J = 10.1, 4.5, 3.5, 0.9 Hz, 1H), 5.98 (ddd, J = 10.1, 2.3, 1.7 Hz, 1H), 5.88 (dt, J = 15.6, 1.6 Hz, 1H), 5.17 (s, 2H), 2.46 – 2.34 (m, 2H), 2.32 – 2.20 (m, 3H), 2.09 (dqd, J = 13.3, 4.8, 1.0 Hz, 1H), 1.89 – 1.80 (m, 1H), 1.75 (dddd, J = 13.3, 11.0, 8.4, 5.8 Hz, 1H), 1.55 – 1.35 (m, 3H).

^{13}C NMR (100 MHz, $CDCl_3$): δ 201.6, 166.6, 149.7, 149.6, 136.3, 129.7, 128.7, 128.3, 128.3, 121.4, 66.2, 46.5, 32.5, 29.0, 28.0, 25.6, 25.3.

IR (Neat Film, NaCl): 2921, 1712, 1673, 1257 cm^{-1}.

HRMS (MM: FD+): *m/z* calc'd for $C_{19}H_{22}O_3$ $[M]^+$: 298.1569, found 298.1565.

Optical Rotation: $[\alpha]_D^{21}$ +3.5 (c 0.20, $CHCl_3$).

SFC conditions: 40% IPA, 2.5 mL/min, Chiralpak IC column, λ = 210 nm, t_R (min): minor = 3.81, major = 4.34.

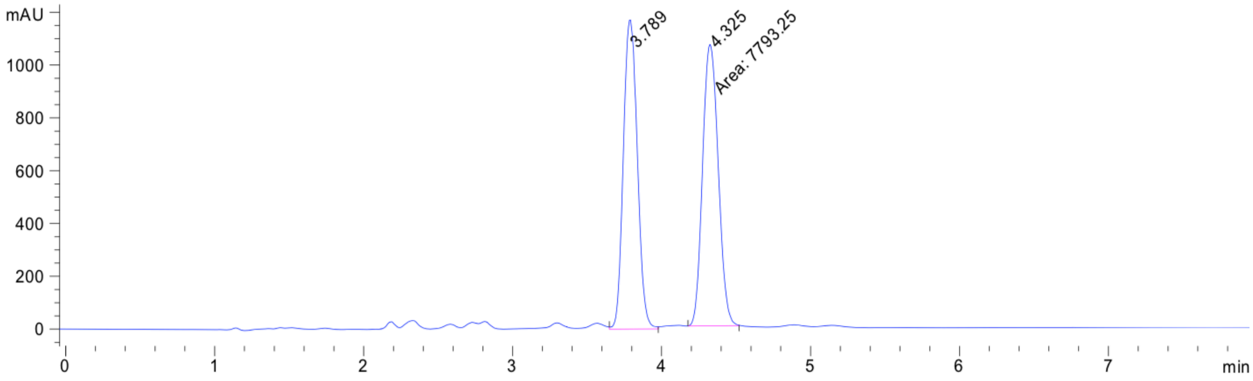

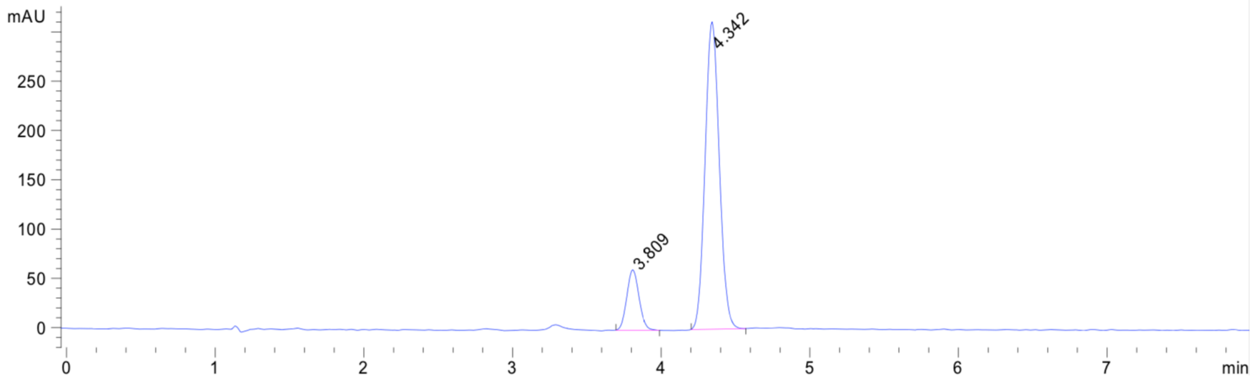

Peak #	RetTime [min]	Type	Width [min]	Area [mAU*s]	Height [mAU]	Area %
1	3.809	BB	0.0888	351.40958	61.33134	14.4774
2	4.342	BB	0.1037	2075.88965	311.79468	85.5226

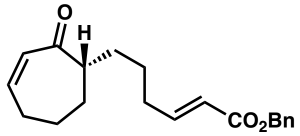

19d

benzyl (*R*,*E*)-7-(2-oxocyclohex-3-en-1-yl)hept-2-enoate (19d)

Isolated as a byproduct from the reaction of **10d** to **11d** as a colorless oil (10.4 mg, 0.0332 mmol, 17% yield, 67% ee).

^{1}H NMR (400 MHz, $CDCl_3$): δ 7.32 – 7.22 (m, 5H), 6.93 (dt, *J* = 15.7, 6.9 Hz, 1H), 6.53 (ddd, *J* = 12.0, 6.7, 4.0 Hz, 1H), 5.94 (ddd, *J* = 11.9, 2.5, 0.9 Hz, 1H), 5.80 (dt, *J* = 15.6, 1.6 Hz, 1H), 5.10

(s, 3H), 2.59 – 2.49 (m, 1H), 2.42 – 2.25 (m, 2H), 2.14 (tdd, J = 7.7, 4.8, 1.4 Hz, 2H), 1.90 – 1.48 (m, 5H), 1.44 – 1.29 (m, 4H).

^{13}C NMR (100 MHz, $CDCl_3$): δ 205.7, 166.5, 149.6, 146.0, 136.2, 132.8, 128.6, 128.2, 128.2, 121.2, 66.0, 51.7, 32.4, 31.1, 29.9, 29.5, 25.8, 25.4.

IR (Neat Film, NaCl): 2917, 1719, 1671, 1266, 1165 cm^{-1}.

HRMS (MM: FD+): *m/z* calc'd for $C_{20}H_{24}O_3$ [M]$^+$: 312.1720, found 312.1734.

Optical Rotation: $[\alpha]_D^{21}$ –0.6 (c 1.00, $CHCl_3$).

SFC conditions: 15% IPA, 2.5 mL/min, Chiralpak AD-H column, λ = 210 nm, t_R (min): minor = 8.23, major = 7.63.

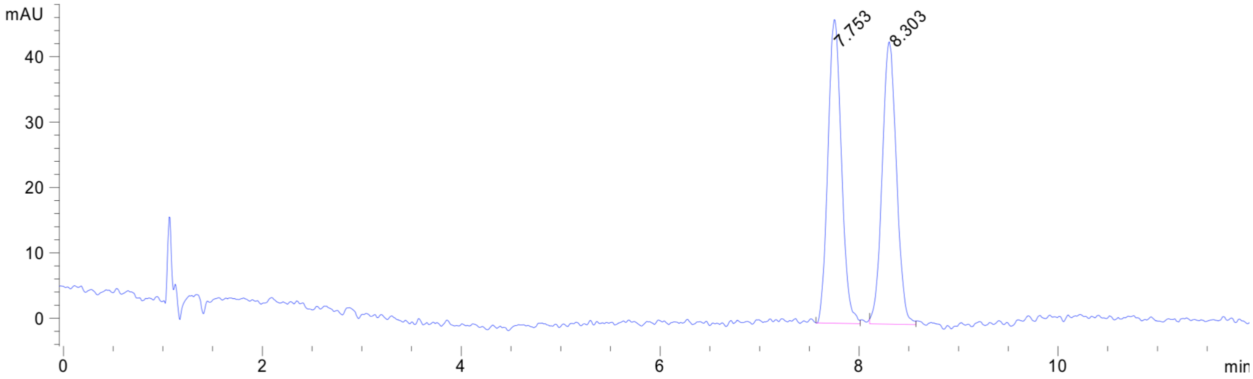

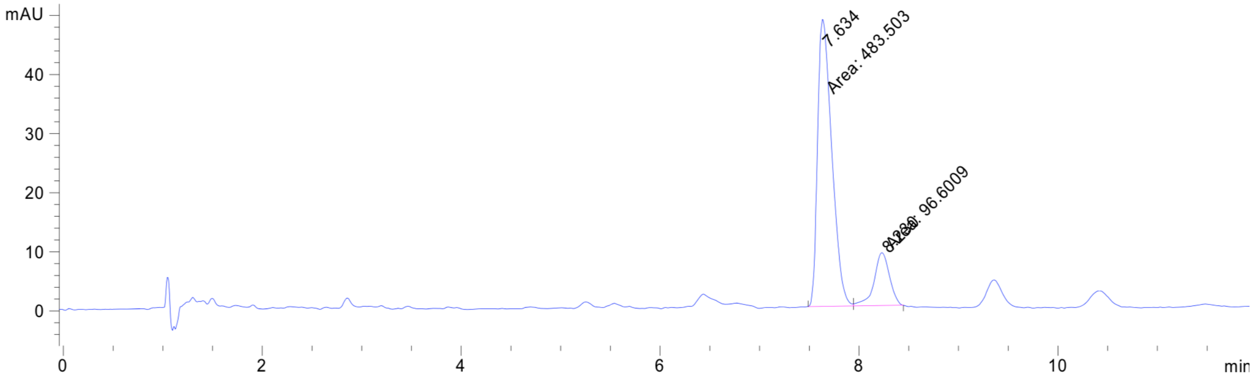

Peak #	RetTime [min]	Type	Width [min]	Area [mAU*s]	Height [mAU]	Area %
1	7.634	MF	0.1658	483.50348	48.61549	83.3477
2	8.230	FM	0.1800	96.60093	8.94598	16.6523

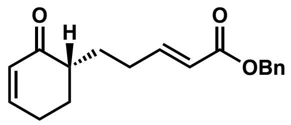

19e

benzyl (*S*,*E*)-5-(2-oxocyclohex-3-en-1-yl)pent-2-enoate (19e)

Isolated as the major byproduct from the reaction of **10e** to **11e**. Purification by flash column chromatography (0–45% EtOAc/hexanes) afforded the title compound as a colorless oil (46.1 mg, 0.16 mmol, 81% yield, 47% ee).

^{1}H NMR (400 MHz, CDCl$_3$): δ 7.42 – 7.28 (m, 5H), 7.01 (dt, *J* = 15.6, 6.9 Hz, 1H), 6.92 (dddd, *J* = 10.0, 4.4, 3.6, 1.0 Hz, 1H), 5.98 (dt, *J* = 10.0, 2.0 Hz, 1H), 5.90 (dt, *J* = 15.7, 1.6 Hz, 1H), 5.17 (s, 2H), 2.44 – 2.24 (m, 5H), 2.15 – 1.96 (m, 2H), 1.82 – 1.69 (m, 1H), 1.62 – 1.44 (m, 1H).

^{13}C NMR (100 MHz, CDCl$_3$): δ 201.3, 166.5, 149.6, 149.4, 136.2, 129.7, 128.7, 128.3, 128.3, 121.6, 66.2, 45.9, 29.7, 28.2, 27.8, 25.4.

IR (Neat Film, NaCl): 3032, 2932, 1719, 1675, 1455, 1386, 1265, 1171 cm^{-1}.

HRMS (MM: FD+): *m/z* calc'd for $C_{18}H_{20}O_3$ $[M]^+$: 284.1412, found 284.1407.

Optical Rotation: $[\alpha]_D^{21}$ +11.8 (c 1.00, $CHCl_3$).

SFC conditions: 10% IPA, 2.5 mL/min, Chiralpak AS-H column, λ = 210 nm, t_R (min): minor = 5.69, major = 6.54.

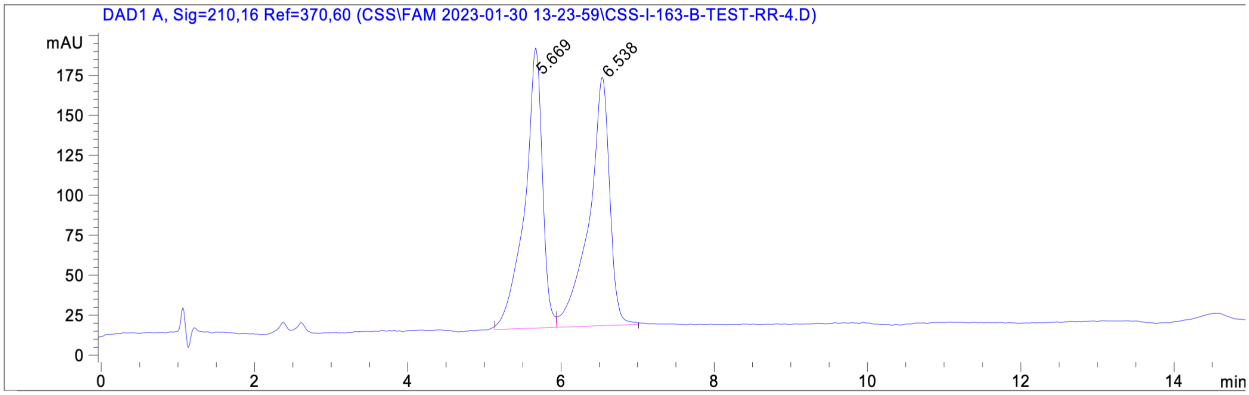

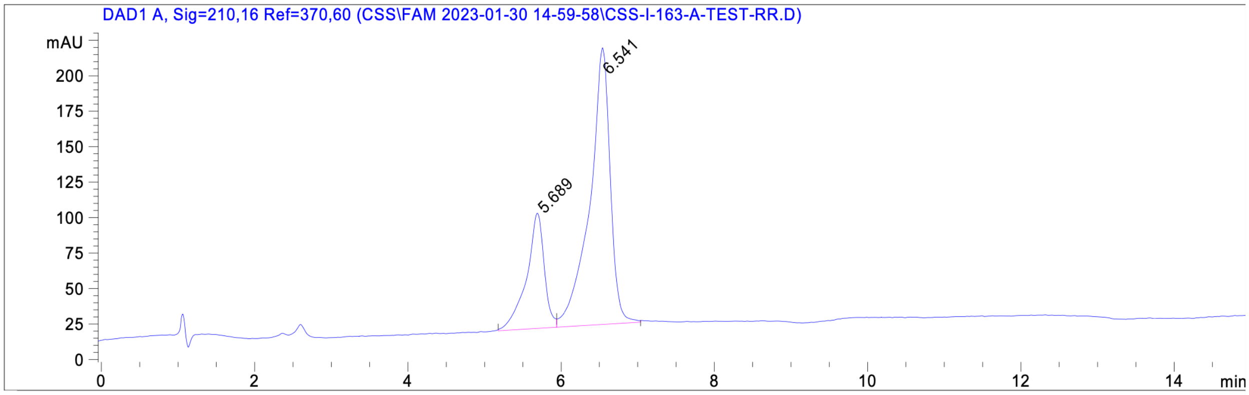

Peak #	RetTime [min]	Type	Width [min]	Area [mAU*s]	Height [mAU]	Area %
1	5.689	BV	0.2296	1305.76550	81.18665	26.5007
2	6.541	VB	0.2626	3621.51782	195.07269	73.4993

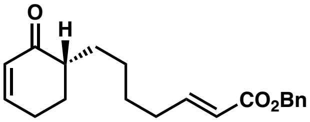

19f

benzyl (*R*,*E*)-7-(2-oxocyclohex-3-en-1-yl)hept-2-enoate (19f)

Isolated as a byproduct from the reaction of **10f** to **11f** as a colorless oil (28.1 mg, 0.0899 mmol, 45% yield, 51% ee). Absolute stereochemistry proposed based on VCD analysis (vide infra).

^{1}H NMR (400 MHz, CDCl$_3$): δ 7.42 – 7.29 (m, 5H), 7.00 (dt, *J* = 15.6, 6.9 Hz, 1H), 6.90 (dddd, *J* = 10.0, 4.5, 3.5, 0.9 Hz, 1H), 5.97 (dt, *J* = 10.1, 2.0 Hz, 1H), 5.86 (dt, *J* = 15.6, 1.6 Hz, 1H), 5.17 (s, 2H), 2.42 – 2.33 (m, 2H), 2.29 – 2.18 (m, 3H), 2.08 (dqd, *J* = 13.3, 4.8, 0.9 Hz, 1H), 1.88 – 1.80 (m, 1H), 1.78 – 1.69 (m, 1H), 1.52 – 1.29 (m, 5H).

^{13}C NMR (100 MHz, CDCl$_3$): δ 201.8, 166.6, 150.0, 149.5, 136.3, 129.6, 128.6, 128.3, 128.3, 121.2, 66.1, 46.6, 32.2, 29.0, 28.2, 27.9, 26.6, 25.2.

IR (Neat Film, NaCl): 2927, 2859, 1716, 1675, 1652, 1262, 1172 cm^{-1}

HRMS (MM: FD+): *m/z* calc'd for $C_{20}H_{24}O_3$ [M]$^+$: 312.1725, found 312.1737.

Optical Rotation: $[\alpha]_D^{21}$ +4.1 (c 1.00, $CHCl_3$).

SFC conditions: 15% IPA, 2.5 mL/min, Chiralpak AD-H column, λ = 210 nm, t_R (min): minor = 7.77, major = 8.44.

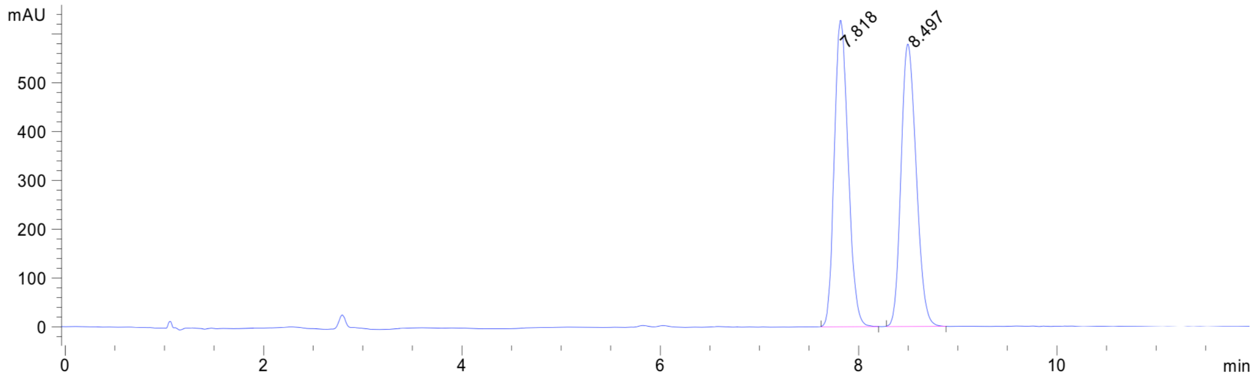

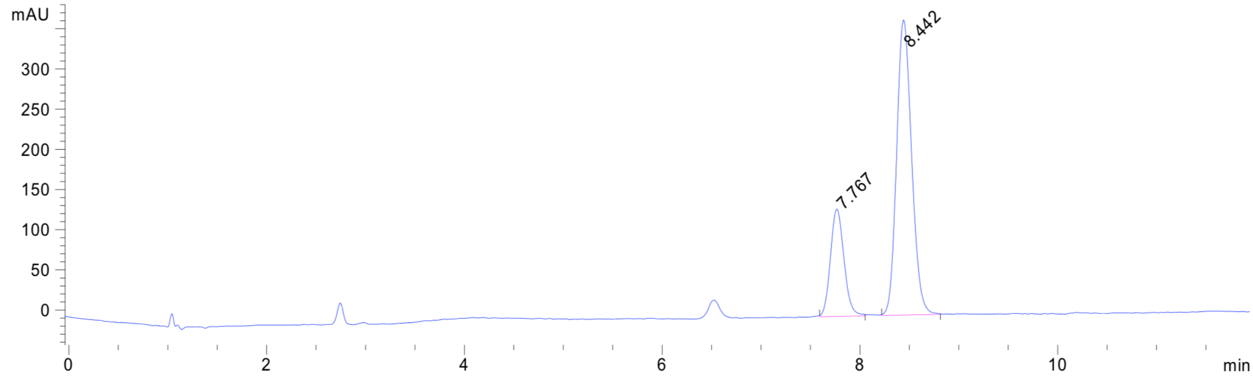

Peak #	RetTime [min]	Type	Width [min]	Area [mAU*s]	Height [mAU]	Area %
1	7.767	BB	0.1469	1268.99805	133.48303	24.6325
2	8.442	BB	0.1634	3882.73218	367.22330	75.3675

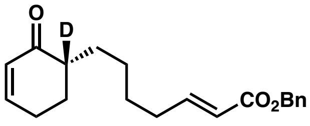

***D*-19f**

benzyl (*R*,*E*)-7-(2-oxocyclohex-3-en-1-yl-1-*d*)hept-2-enoate (*D*-19f)

Isolated as a byproduct from the reaction of ***D*-10f** to ***D*-11f** as a colorless oil (14.9 mg, 0.0475 mmol, 24% yield, 55% ee).

^{1}H NMR (400 MHz, $CDCl_3$): δ 7.39 – 7.29 (m, 5H), 7.00 (dt, *J* = 15.6, 7.0 Hz, 1H), 6.91 (dddd, *J* = 10.1, 4.5, 3.5, 0.9 Hz, 1H), 5.97 (dddd, *J* = 10.0, 2.3, 1.6, 0.6 Hz, 1H), 5.86 (dt, *J* = 15.6, 1.6 Hz, 1H), 5.17 (s, 2H), 2.44 – 2.33 (m, 2H), 2.28 – 2.18 (m, 2.5H), 2.09 (ddt, *J* = 13.3, 5.8, 4.5 Hz, 1H), 1.87 – 1.70 (m, 2H), 1.53 – 1.27 (m, 5H).

^{13}C NMR (100 MHz, $CDCl_3$): δ 201.9, 166.6, 150.0, 149.6, 129.7, 129.7, 128.7, 128.3, 128.3, 121.2, 66.2, 46.6, 32.3, 29.0, 28.9, 28.2, 27.9, 27.8, 26.7, 26.6, 25.3, 25.2.

^{2}H NMR (61 MHz, $CHCl_3$): δ 2.25.

IR (Neat Film, NaCl): 2929, 2857, 1714, 1697, 1267, 1174 cm^{-1}.

HRMS (MM: FD+): *m/z* calc'd for $C_{20}H_{23}DO_3$ $[M+H]^+$: 313.1783, found 313.1788.

Optical Rotation: $[\alpha]_D^{21}$ +5.8 (c 0.50, $CHCl_3$).

SFC conditions: 15% IPA, 2.5 mL/min, Chiralpak AD-H column, λ = 210 nm, t_R (min): minor = 7.96, major = 8.67.

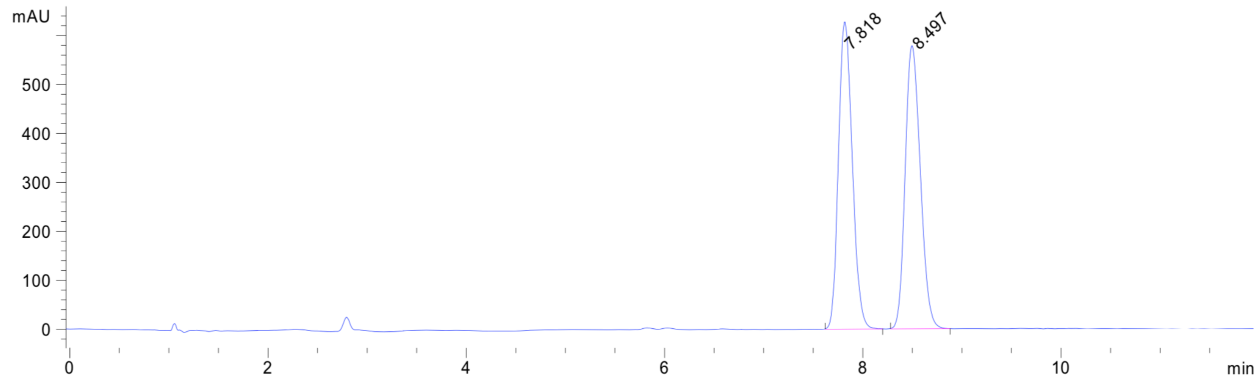

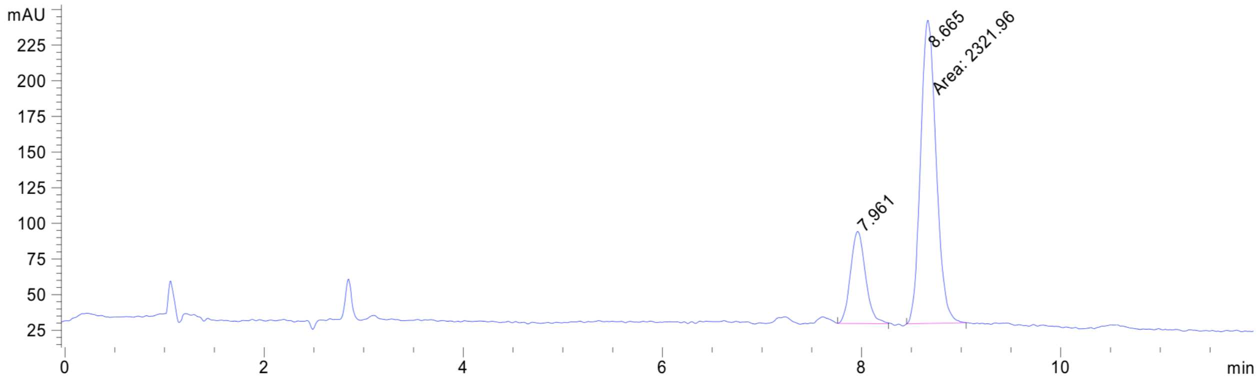

Peak #	RetTime [min]	Type	Width [min]	Area [mAU*s]	Height [mAU]	Area %
1	7.961	VB	0.1623	678.20441	64.68045	22.6055
2	8.665	MM	0.1818	2321.96411	212.87651	77.3945

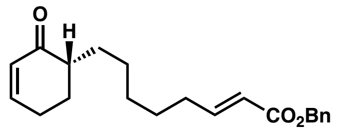

19g

benzyl (*R*,*E*)-8-(2-oxocyclohex-3-en-1-yl)oct-2-enoate (19g)

Isolated as the major product from the reaction of **10g** to **11g** as a colorless oil (50.5 mg, 0.155 mmol, 77% yield, 56% ee).

^{1}H NMR (400 MHz, $CDCl_3$): δ 7.40 – 7.29 (m, 5H), 7.01 (dt, *J* = 15.6, 6.9 Hz, 1H), 6.91 (dddd, *J* = 10.0, 4.5, 3.5, 0.9 Hz, 1H), 5.97 (dt, *J* = 10.0, 2.0 Hz, 1H), 5.86 (dt, *J* = 15.6, 1.6 Hz, 1H), 5.17 (s, 2H), 2.45 – 2.33 (m, 2H), 2.30 – 2.16 (m, 3H), 2.09 (dqd, *J* = 14.5, 5.0, 0.9 Hz, 1H), 1.86 – 1.70 (m, 2H), 1.51 – 1.42 (m, 2H), 1.41 – 1.28 (m, 5H).

^{13}C NMR (100 MHz, $CDCl_3$): δ 202.0, 166.7, 150.2, 149.5, 136.3, 129.7, 128.7, 128.3, 128.3, 121.1, 66.1, 46.6, 32.3, 29.3, 29.1, 28.0, 27.9, 26.8, 25.2.

IR (Neat Film, NaCl): 2927, 2859, 1718, 1677, 1555, 1450, 1257, 1165 cm^{-1}

HRMS (MM: FD+): *m/z* calc'd for $C_{21}H_{26}O_3$ $[M]^+$: 326.1877, found 326.1891.

Optical Rotation: $[\alpha]_D^{21}$ +7.0 (c 1.00, $CHCl_3$).

SFC conditions: 15% IPA, 2.5 mL/min, Chiralpak AD-H column, λ = 210 nm, t_R (min): minor = 9.70, major = 10.46.

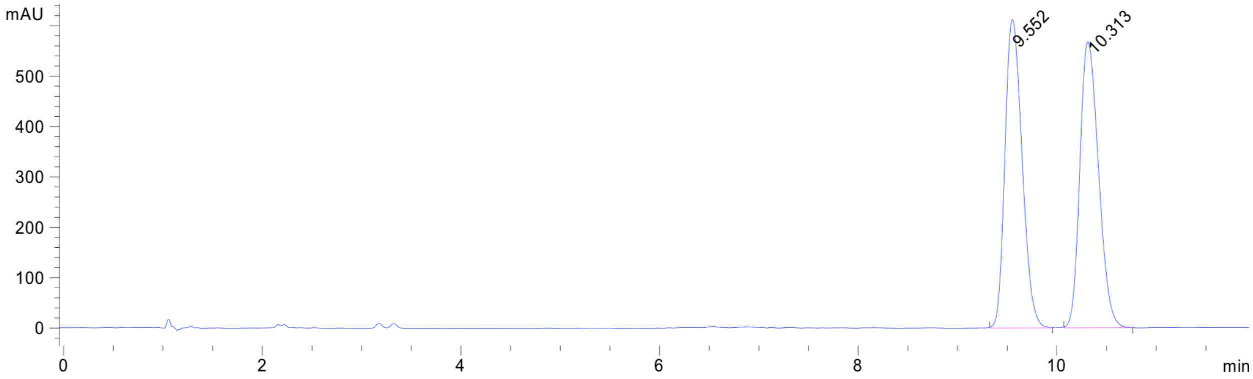

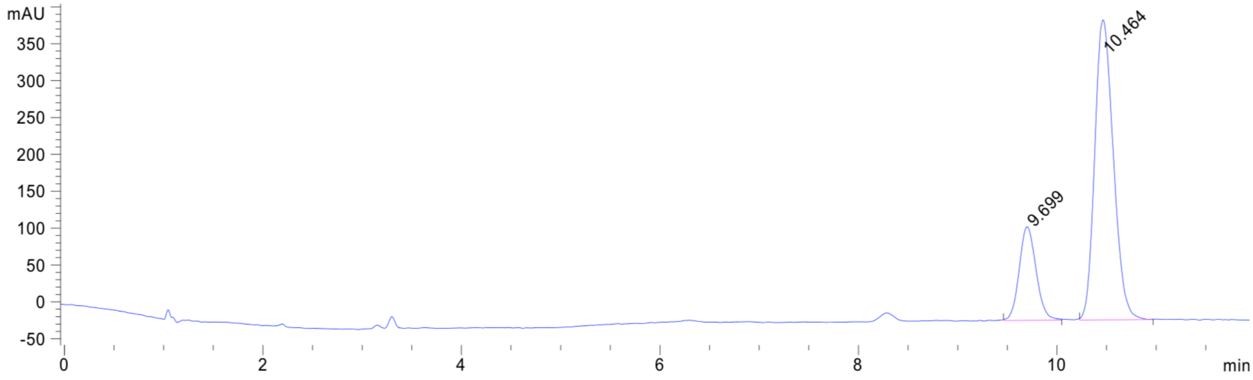

Peak #	RetTime [min]	Type	Width [min]	Area [mAU*s]	Height [mAU]	Area %
1	9.699	BB	0.1842	1504.03284	126.98264	22.0210
2	10.464	BB	0.2024	5325.96289	407.59467	77.9790

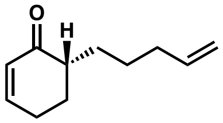

19h

6-(pent-4-en-1-yl)cyclohex-2-en-1-one (19h)

Isolated as the major product from the reaction of **10h** to **11h** as a colorless oil. Purification by flash column chromatography (0–15% EtOAc/hexanes) afforded the title compound as a colorless oil (11.0 mg, 0.067 mmol, 33% yield, 58% ee)

^{1}H NMR (400 MHz, CDCl$_3$): δ 6.91 (dddd, J = 10.1, 4.4, 3.5, 0.9 Hz, 1H), 5.97 (dt, J = 10.0, 2.1 Hz, 1H), 5.81 (ddt, J = 16.9, 10.2, 6.6 Hz, 1H), 5.01 (dq, J = 17.2, 1.8 Hz, 1H), 4.94 (dd, J = 10.1, 1.3 Hz, 1H), 2.45 – 2.24 (m, 3H), 2.16 – 1.98 (m, 2=3H), 1.95 – 1.69 (m, 2H), 1.55 – 1.31 (m, 3H).

^{13}C NMR (100 MHz, CDCl$_3$): δ 202.0, 149.5, 138.8, 129.7, 114.7, 46.6, 34.0, 28.8, 27.9, 26.4, 25.2.

IR (Neat Film, NaCl): 2925, 2859, 1677, 1639, 1456, 1387, 1215, 912 cm^{-1}.

HRMS (MM: FD+): *m/z* calc'd for $C_{11}H_{16}O$ $[M]^+$: 164.1201, found 164.1201.

Optical Rotation: $[\alpha]_D^{21}$ –111.5 (c 1.00, CHCl$_3$).

SFC conditions: 3% IPA, 2.5 mL/min, Chiralpak AD-H column, λ = 210 nm, t_R (min): minor = 4.41, major = 4.11.

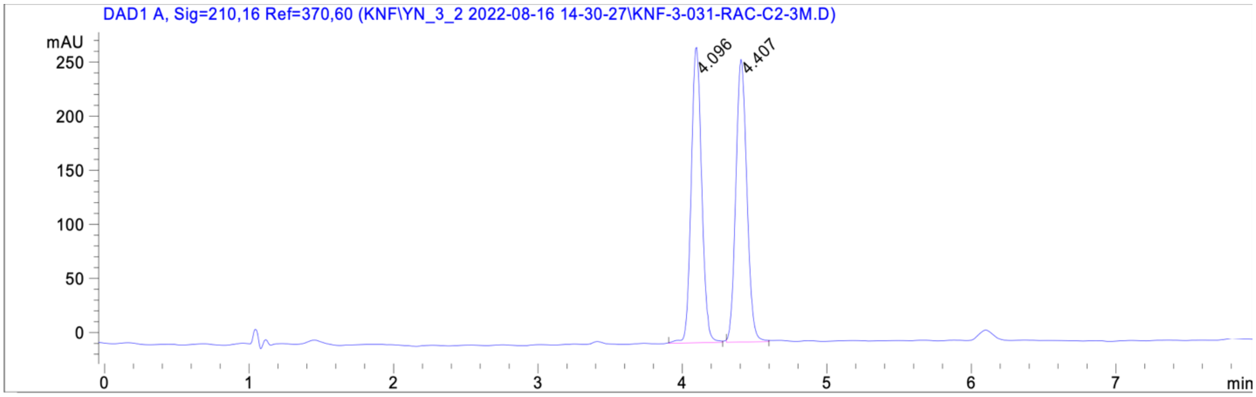

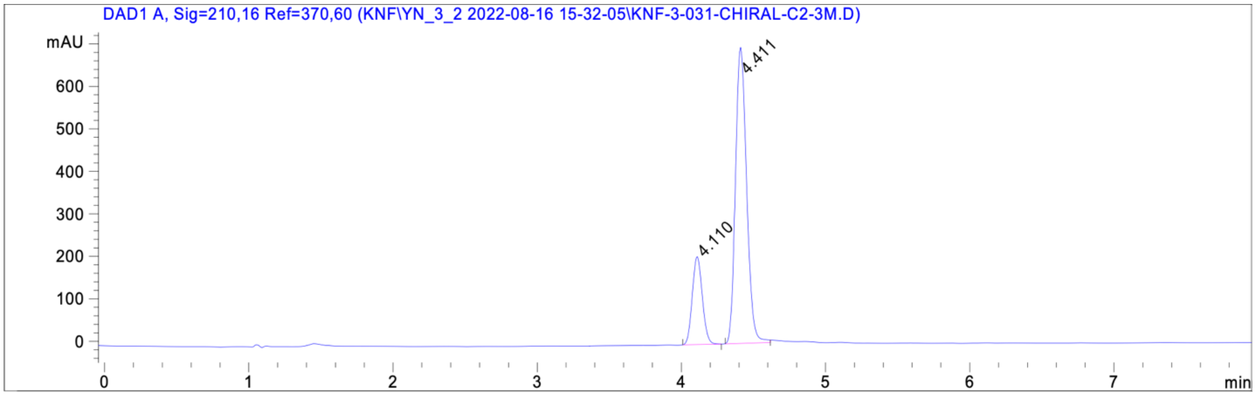

Peak #	RetTime [min]	Type	Width [min]	Area [mAU*s]	Height [mAU]	Area %
1	4.110	BB	0.0764	1001.27991	207.19360	21.1231
2	4.411	BB	0.0846	3738.93604	696.40063	78.8769

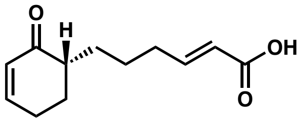

19i

(*E*)-6-(2-oxocyclohex-3-en-1-yl)hex-2-enoic acid (19i)

Isolated as the major product from the reaction of **10i** to **11hi** as a colorless oil. Purification by flash column chromatography (35% EtOAc/hexanes with 3% AcOH) afforded the title compound as a white solid (33.2 mg, 0.16 mmol, 80% yield, 9% ee).

^{1}H NMR (400 MHz, CDCl$_3$): δ 7.07 (dtd, *J* = 15.5, 7.0, 1.5 Hz, 1H), 6.97 – 6.87 (m, 1H), 5.98 (dq, *J* = 10.1, 1.9 Hz, 1H), 5.84 (dt, *J* = 15.6, 1.6 Hz, 1H), 2.49 – 2.35 (m, 2H), 2.34 – 2.19 (m, 3H), 2.16 – 2.04 (m, 1H), 1.92 – 1.81 (m, 1H), 1.81 – 1.70 (m, 1H), 1.63 – 1.48 (m, 2H), 1.48 – 1.35 (m, 1H).

^{13}C NMR (100 MHz, CDCl$_3$): δ 201.7, 171.5, 151.9, 149.7, 129.7, 120.9, 46.5, 32.5, 29.0, 28.0, 25.5.

IR (Neat Film, NaCl): 2928, 2857, 1731, 1454, 1155 cm^{-1}

HRMS (MM: FD+): *m/z* calc'd for $C_{12}H_{17}O_3$ [M]$^+$: 209.1178, found 209.1168.

Optical Rotation: $[\alpha]_D^{21}$ 2.2 (c 1.00, $CHCl_3$).

SFC conditions: 30% IPA, 2.5 mL/min, Chiralpak IC column, l = 210 nm, t_R (min): minor = 3.30, major = 4.02

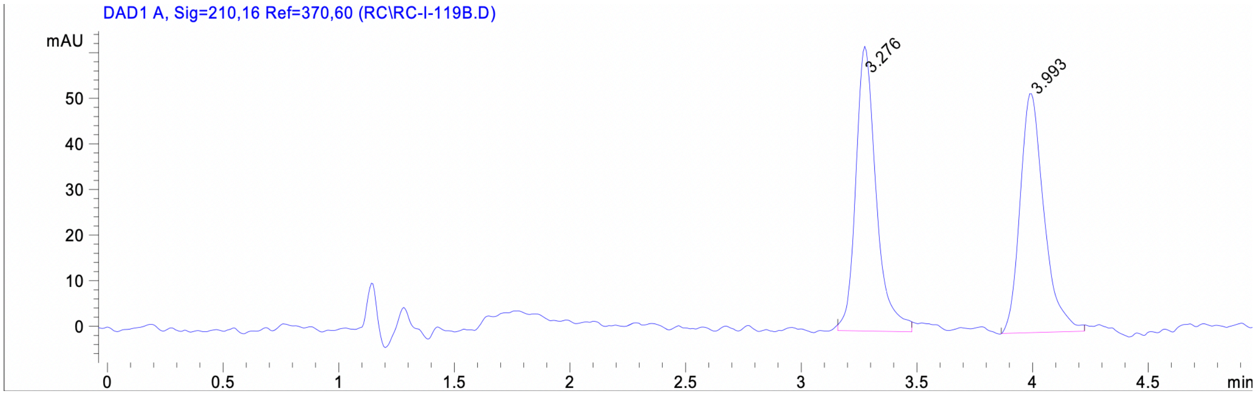

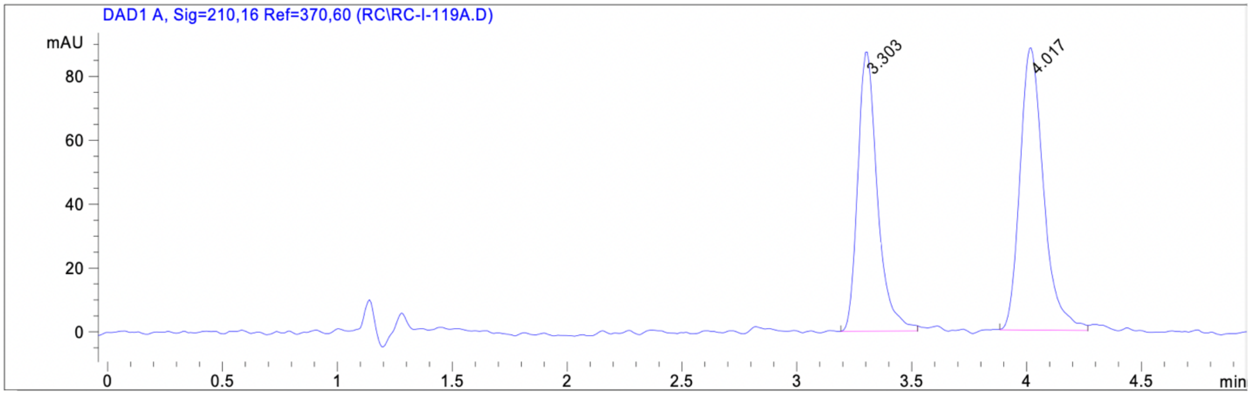

Peak #	RetTime [min]	Type	Width [min]	Area [mAU*s]	Height [mAU]	Area %
1	3.303	BB	0.0936	523.17853	87.69068	45.2708
2	4.017	BB	0.1114	632.48499	88.47423	54.7292

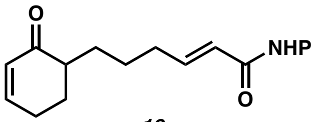

19m

1,3-dioxoisoindolin-2-yl (*E*)-6-(2-oxocyclohex-3-en-1-yl)hex-2-enoate (19m)

Isolated as a byproduct product from the reaction of **10m** to **11m** as a colorless oil. Purification by flash column chromatography (0–35% EtOAc/hexanes) afforded the title compound as a white solid (7.7 mg, 0.022 mmol, 11% yield).

^{1}H NMR (400 MHz, CDCl$_3$): δ 7.89 (dd, *J* = 5.5, 3.1 Hz, 2H), 7.79 (dd, *J* = 5.5, 3.1 Hz, 2H), 7.32 (dt, *J* = 15.8, 6.9 Hz, 1H), 6.93 (dddd, *J* = 10.1, 4.5, 3.5, 1.0 Hz, 1H), 6.10 (dt, *J* = 15.8, 1.6 Hz, 1H), 5.99 (ddd, *J* = 10.0, 2.3, 1.7 Hz, 1H), 2.50 – 2.25 (m, 5H), 2.12 (m, 1H), 1.95 – 1.84 (m, 1H), 1.78 (m, 1H), 1.59 (m, 2H), 1.45 (m, 1H).

^{13}C NMR (100 MHz, CDCl$_3$): δ 201.5, 162.5, 162.2, 155.6, 149.7, 134.9, 129.7, 129.1, 124.1, 115.9, 46.5, 33.2, 29.1, 28.1, 25.4, 25.3.

IR (Neat Film, NaCl): 2931, 1770, 1745, 1673, 1466, 1360, 1186 cm^{-1}.

HRMS (MM: FD+): *m/z* calc'd for $C_{20}H_{19}NO_5$ $[M]^+$: 353.1263, found 353.1242.

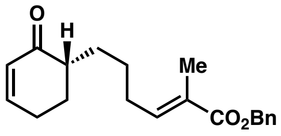

19r

benzyl (*R*,*E*)-2-methyl-6-(2-oxocyclohex-3-en-1-yl)hex-2-enoate (19r)

Isolated as a byproduct from the reaction of **10r** to **11r** as a colorless oil (23.2 mg, 0.0743 mmol, 37% yield, 59% ee).

^{1}H NMR (400 MHz, CDCl$_3$): δ 7.40 – 7.29 (m, 5H), 6.91 (dddd, *J* = 10.1, 4.5, 3.5, 0.9 Hz, 1H), 6.81 (td, *J* = 7.5, 1.5 Hz, 1H), 5.97 (dt, *J* = 10.0, 1.9 Hz, 1H), 5.18 (s, 2H), 2.45 – 2.34 (m, 2H), 2.32 – 2.17 (m, 3H), 2.09 (dqd, *J* = 13.4, 4.9, 1.0 Hz, 1H), 1.90 – 1.81 (m, 4H), 1.80 – 1.71 (m, 1H), 1.55 – 1.36 (m, 3H).

^{13}C NMR (100 MHz, CDCl$_3$): δ 201.7, 168.1, 149.6, 142.7, 136.6, 129.7, 128.6, 128.2, 128.1, 127.9, 66.3, 46.6, 29.2, 29.0, 28.0, 26.2, 25.2, 12.6.

IR (Neat Film, NaCl): 3033, 2932, 2861, 1710, 1677, 1256 cm^{-1}.

HRMS (MM: FD+): *m/z* calc'd for $C_{20}H_{24}O_3$ $[M]^+$: 312.1725, found 312.1729.

Optical Rotation: $[\alpha]_D^{21}$ +8.6 (c 1.00, $CHCl_3$).

SFC conditions: 15% IPA, 2.5 mL/min, Chiralpak AD-H column, λ = 210 nm, t_R (min): minor = 5.89, major = 6.22.

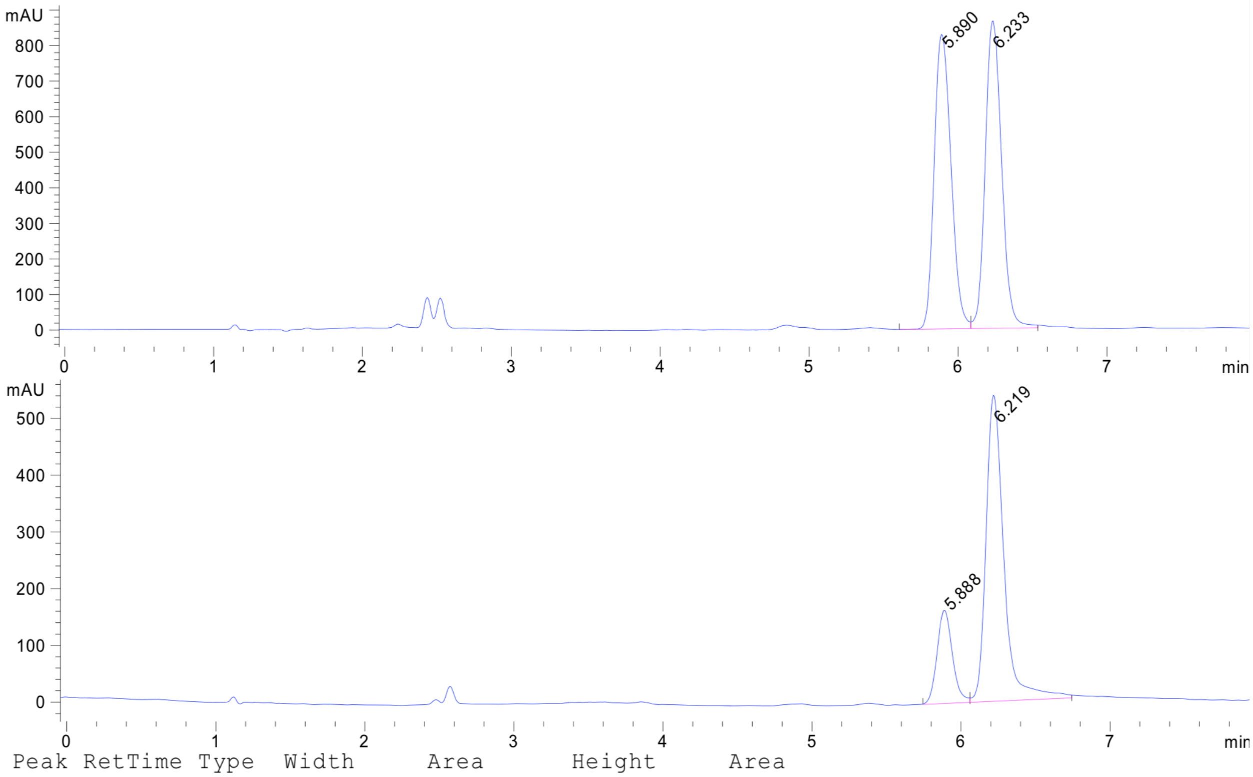

Peak #	RetTime [min]	Type	Width [min]	Area [mAU*s]	Height [mAU]	Area %
1	5.888	BV	0.1116	1179.92444	164.63115	20.7199
2	6.219	VB	0.1256	4514.71436	539.84412	79.2801

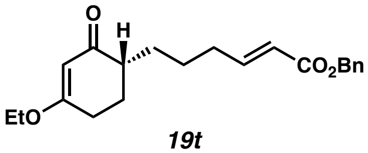

19t

benzyl (*E*)-6-(4-ethoxy-2-oxocyclohex-3-en-1-yl)hex-2-enoate (19t)

Isolated as a byproduct from the reaction of **10t** to **11t** as a colorless oil (5.2 mg, 0.015 mmol, 8% yield, 45% ee)

^{1}H NMR (400 MHz, $CDCl_3$): δ 7.41 – 7.27 (m, 5H), 7.01 (dt, *J* = 15.6, 6.9 Hz, 1H), 5.87 (dt, *J* = 15.6, 1.6 Hz, 1H), 5.31 (s, 1H), 5.17 (s, 2H), 3.88 (qd, *J* = 7.1, 1.4 Hz, 2H), 2.41 (dd, *J* = 7.2, 5.3

Hz, 2H), 2.29 – 2.13 (m, 3H), 2.06 (dq, J = 13.2, 5.2 Hz, 1H), 1.86 (ddt, J = 13.3, 11.1, 5.2 Hz, 1H), 1.78 – 1.64 (m, 1H), 1.59 – 1.37 (m, 3H), 1.35 (t, J = 7.0 Hz, 3H).

^{13}C NMR (100 MHz, $CDCl_3$): δ 201.4, 176.9, 166.6, 149.7, 136.3, 128.7, 128.3, 128.3, 121.3, 102.3, 66.1, 64.4, 45.1, 32.5, 29.3, 28.2, 26.4, 25.7, 14.3.

IR (Neat Film, NaCl): 2919, 1718, 1648, 1605, 1456, 1377, 1260, 1190, 732 cm^{-1}.

HRMS (MM: FD+): *m/z* calc'd for $C_{21}H_{26}O_4$ $[M]^+$: 342.1828, found 342.1826.

Optical Rotation: $[\alpha]_D^{21}$ –5.5 (c 0.34, $CHCl_3$).

SFC conditions: 15% IPA, 2.5 mL/min, Chiralpak AD-H column, λ = 210 nm, t_R (min): minor = 10.17, major = 11.69.

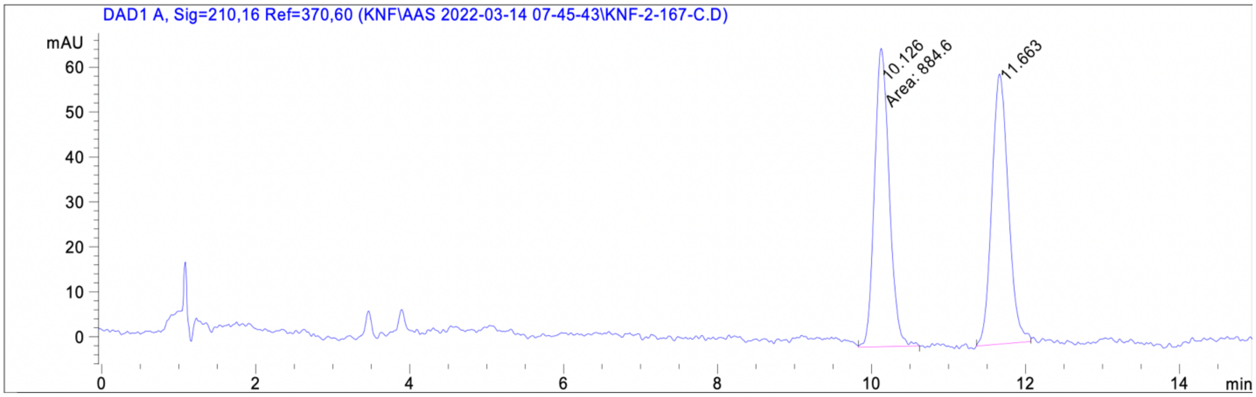

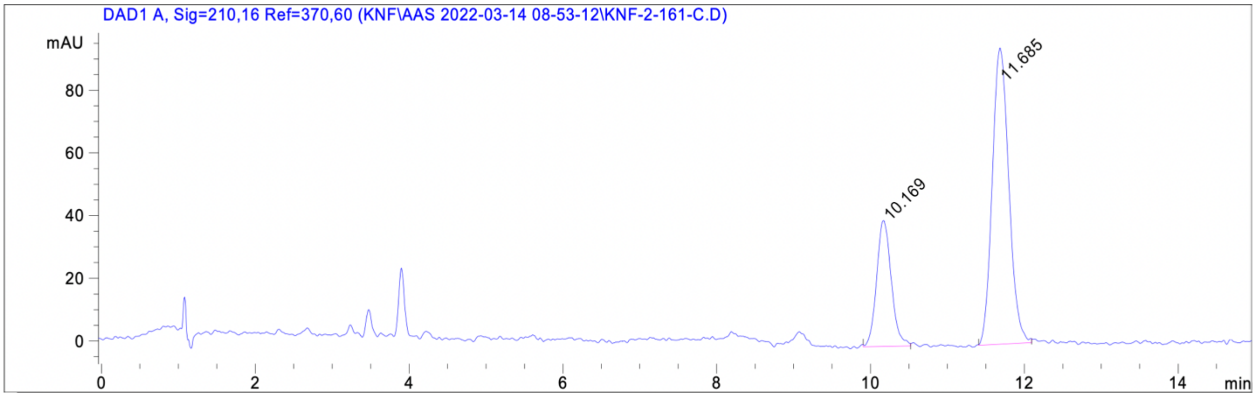

Peak #	RetTime [min]	Type	Width [min]	Area [mAU*s]	Height [mAU]	Area %
1	10.169	BB	0.2026	531.98450	40.15448	27.3169
2	11.685	BB	0.2307	1415.47424	94.55222	72.6831

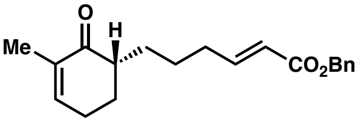

19u

benzyl (*E*)-6-(3-methyl-2-oxocyclohex-3-en-1-yl)hex-2-enoate (19u)

Isolated as a byproduct from the reaction of **10u** to **11u** as a colorless oil (23.6 mg, 0.075 mmol, 37% yield, 46% ee).

^{1}H NMR (400 MHz, CDCl$_3$): δ 7.41 – 7.28 (m, 5H), 7.01 (dt, *J* = 15.5, 6.9 Hz, 1H), 6.71 – 6.63 (m, 1H), 5.88 (dt, *J* = 15.6, 1.6 Hz, 1H), 5.17 (s, 2H), 2.33 (ddq, *J* = 6.3, 4.8, 2.0 Hz, 2H), 2.29 – 2.19 (m, 3H), 2.10 – 2.01 (m, 1H), 1.88 – 1.77 (m, 1H), 1.76 (q, *J* = 1.7 Hz, 3H), 1.74 – 1.69 (m, 1H), 1.56 – 1.34 (m, 3H).

^{13}C NMR (100 MHz, CDCl$_3$): δ 201.9, 166.6, 149.8, 144.5, 136.3, 135.3, 128.68, 128.3, 128.3, 121.3, 66.2, 46.6, 32.5, 29.2, 28.4, 25.7, 25.2, 16.3.

IR (Neat Film, NaCl): 2925, 1718, 1670, 1455, 1262, 1172, 1013 cm^{-1}.

HRMS (MM: FD+): *m/z* calc'd for $C_{20}H_{24}O_3$ [M]$^{+}$: 312.1725, found 312.1721.

Optical Rotation: $[\alpha]_D^{21}$ –14.7 (c 0.35, CHCl$_3$).

SFC conditions: 10% IPA, 2.5 mL/min, Chiralpak OJ-H column, λ = 210 nm, t_R (min): minor = 7.18, major = 7.62.

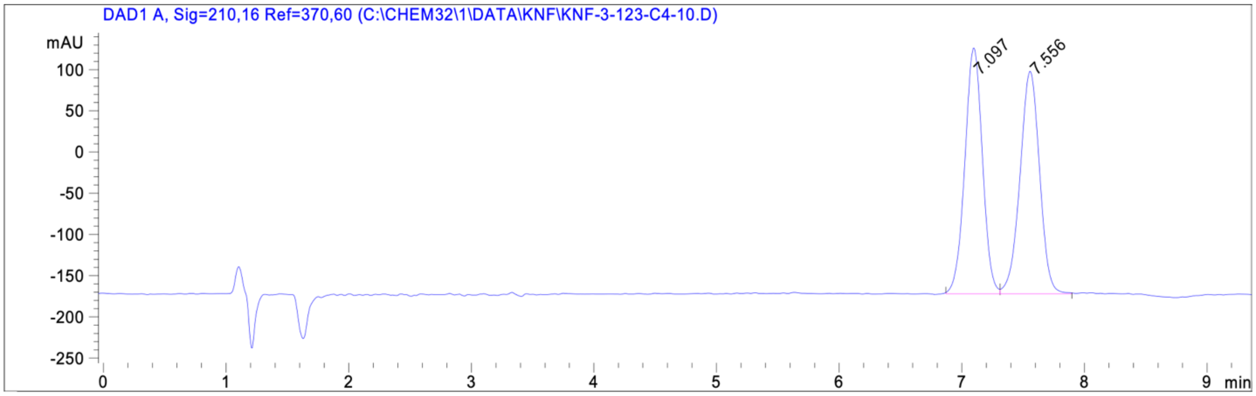

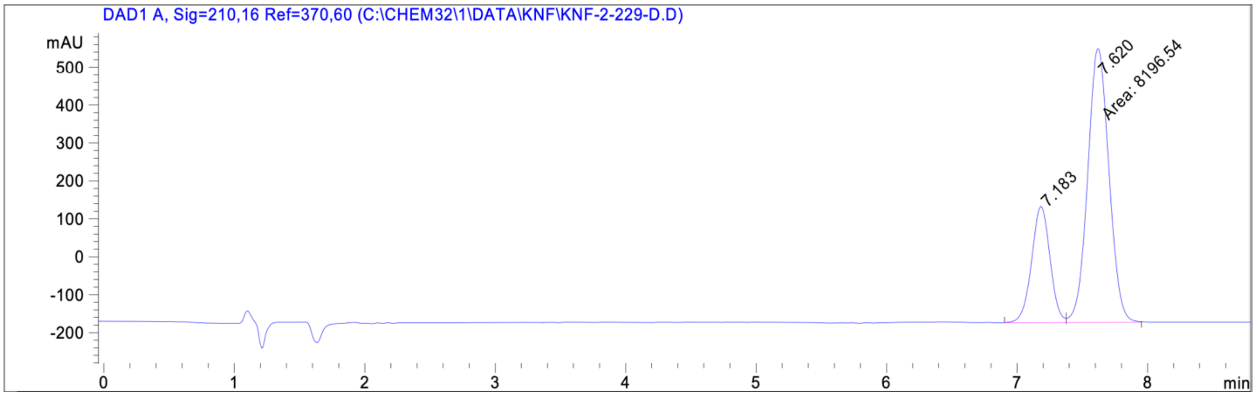

Peak #	RetTime [min]	Type	Width [min]	Area [mAU*s]	Height [mAU]	Area %
1	7.183	BV	0.1527	3013.37598	306.49200	26.8813
2	7.620	MF	0.1887	8196.54199	723.78693	73.1187

Product Derivatizations

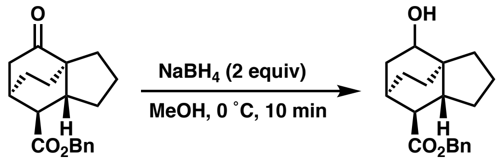

benzyl (3a*R*,6*R*,7*S*,7a*R*)-4-hydroxyoctahydro-3a,6-ethanoindene-7-carboxylate (38)

To a solution of ketone **11a** (0.125 mmol, 1 equiv) in methanol (4.4 mL) was added $NaBH_4$ (0.25 mmol, 2 equiv) at 0 ºC. The reaction was allowed to stir for 10 min at 0 ºC and then was diluted with water. The aqueous layer was extracted with dichloromethane (3x), and the combined organic layers were dried over Na_2SO_4. Concentration under reduced pressure afforded the title compound as a colorless oil (37.6 mg, 0.125 mmol, 99% yield).

^{1}H NMR (400 MHz, CDCl$_3$): δ 7.42 – 7.30 (m, 5H), 5.19 – 5.05 (m, 2H), 3.76 (dd, *J* = 8.9, 5.3 Hz, 0.4H, minor), 3.69 (dt, *J* = 8.9, 1.5 Hz, 0.6H, major), 2.35 – 2.24 (m, 1H), 2.15 – 1.95 (m, 2H), 1.95 – 1.83 (m, br, 1H), 1.83 – 1.56 (m, 6H), 1.53 – 1.23 (m, 5H), 1.15 – 1.01 (m, 1H).

^{13}C NMR (100 MHz, CDCl$_3$): δ 175.8, 175.6, 136.3, 128.6, 128.2, 128.2, 128.0, 128.0, 75.6, 70.3, 66.3, 66.3, 49.21, 48.1, 44.5, 44.2, 43.2, 36.8, 35.0, 34.9, 34.0, 30.5, 30.3, 30.1, 29.6, 29.0, 26.7, 26.3, 22.8, 22.8, 20.3.

IR (Neat Film, NaCl): 3438, 3032, 2942, 2865, 1730, 1455, 1162 cm^{-1}

HRMS (MM: FD+): *m/z* calc'd for $C_{19}H_{24}O_3$ [M]$^+$: 300.1725, found 300.1730.

Optical Rotation: $[\alpha]_D^{21}$ –31.1 (c 1.00, $CHCl_3$).

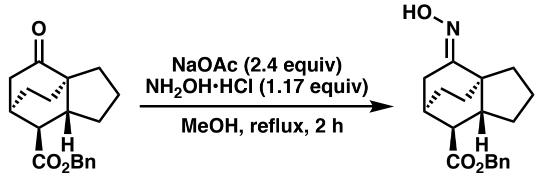

11a ***S10***

benzyl (3a*R*,6*R*,7*S*,7a*R*,*E*)-4-(hydroxyimino)octahydro-3a,6-ethanoindene-7-carboxylate (S10)

To a stirred solution of ketone **11a** (0.125 mmol, 1 equiv) in methanol (1.25 mL) was added NaOAc (0.3 mmol, 2.4 equiv), NH_2OH•HCl (0.15 mmol. 1.17 equiv), and water (0.05 mL). The reaction was brought to reflux for 2 h and was subsequently cooled to 23 ºC and concentrated under reduced pressure. The crude mixture was then diluted with water and extracted with EtOAc (3x), washed with a saturated aqueous solution of $NaHCO_3$ and brine, dried with Na_2SO_4, and concentrated under reduced pressure. The material was used in the next step without further purification assuming quantitative yield.

^{1}H NMR (400 MHz, CDCl$_3$): δ 7.42 – 7.28 (m, 5H), 5.20 – 5.06 (m, 2H), 2.52 – 2.46 (m, 2H), 2.43 – 2.39 (m, 1H), 2.39 – 2.36 (m, 1H), 2.15 (dtd, *J* = 10.2, 8.2, 1.8 Hz, 1H), 2.07 (ddt, *J* = 11.9, 8.3, 4.1 Hz, 1H), 1.98 (ddd, *J* = 13.0, 11.0, 8.0 Hz, 1H), 1.82 – 1.71 (m, 3H), 1.67 (ddd, *J* = 10.9,

3.6, 1.8 Hz, 1H), 1.48 (dddd, J = 12.5, 7.8, 6.5, 3.7 Hz, 2H), 1.36 (dddd, J = 23.6, 11.1, 8.8, 2.6 Hz, 2H).

^{13}C NMR (100 MHz, $CDCl_3$): δ 174.8, 165.4, 136.2, 128.7, 128.4, 128.2, 66.5, 48.2, 45.7, 44.4, 30.6, 29.2, 28.9, 28.1, 27.2, 25.5, 22.6.

IR (Neat Film, NaCl): 2945, 1731, 1161 cm^{-1}.

HRMS (MM: FD+): *m/z* calc'd for $C_{19}H_{23}NO_3$ $[M]^+$: 313.1678, found 313.1676.

Optical Rotation: $[\alpha]_D^{21}$ –22.0 (c 0.37, $CHCl_3$).

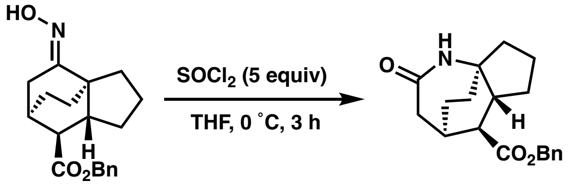

S10 *39*

benzyl (4*R*,5*S*,5a*R*,8a*R*)-2-oxooctahydro-1*H*-4,8a-ethanocyclopenta[*b*]azepine-5-carboxylate (39)

To a solution of **S10** (0.125 mmol, 1 equiv) in THF (1.25 mL) at 0 ºC was added a solution of $SOCl_2$ (0.625 mmol, 5 equiv) in THF (0.23 mL). The reaction was stirred for 3 h at 0 ºC, followed by dilution with water. Aqueous solution NH_4OH was added to the reaction mixture until neutral, and the aqueous layer was extracted with dichloromethane (3x). The combined organic layers were washed with water and brine, dried over Na_2SO_4, and concentrated under reduced pressure. Purification by flash column chromatography (35% EtOAc/hexanes) afforded the title compound as a colorless oil (22 mg, 0.167 mmol, 56% yield over two steps).

^{1}H NMR (400 MHz, $CDCl_3$): δ 7.41 – 7.27 (m, 5H), 6.67 (s, 1H, br), 5.15 (q, J = 12.3 Hz, 2H), 2.73 (dt, J = 18.1, 1.6 Hz, 1H), 2.62 (tdd, J = 9.9, 8.0, 1.4 Hz, 1H), 2.48 (dd, J = 18.4, 6.9 Hz, 1H), 2.40 – 2.32 (m, 2H), 2.29 – 2.16 (m, 1H), 1.95 (dtd, J = 12.7, 8.7, 2.2 Hz, 1H), 1.82 – 1.62 (m, 7H), 1.62 – 1.52 (m, 1H), 1.44 – 1.30 (m, 1H).

^{13}C NMR (100 MHz, CDCl$_3$): δ 174.6, 174.2, 135.9, 128.8, 128.4, 128.3, 66.7, 59.9, 48.8, 48.5, 39.2, 33.3, 31.1, 29.8, 25.1, 23.3.

IR (Neat Film, NaCl): 3182, 3055, 2934, 1727, 1648, 1456, 1398, 1167 cm^{-1}.

HRMS (MM: FD+): *m/z* calc'd for $C_{19}H_{23}NO_3$ [M]$^+$: 313.1678, found 313.1678.

Optical Rotation: $[\alpha]_D^{21}$ –5.5 (c 0.89, $CHCl_3$).

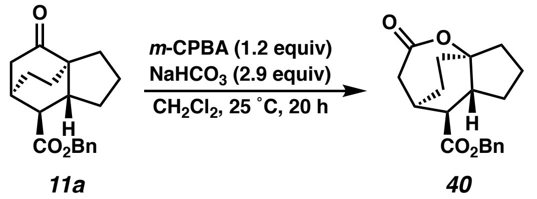

benzyl (4*R*,5*S*,5a*R*,8a*R*)-2-oxooctahydro-4,8a-ethanocyclopenta[*b*]oxepine-5-carboxylate (40)

To a solution ketone **11a** (37 mg, 0.13 mmol, 1 equiv) in CH_2Cl_2 (1.25 mL, 0.1 M) at 0°C was added $NaHCO_3$ (30.8 mg, 0.37 mmol, 2.9 equiv). Subsequently, *m*-CPBA (31 mg, 0.15 mmol, 1.2 equiv) was added and the reaction was allowed to warm to 25 °C. Upon complete consumption of starting material (as determined by TLC), the reaction mixture was diluted with a saturated solution of $Na_2S_2O_3$ and extracted with Et_2O (3x). The combined organic layers were washed with a saturated solution of $NaHCO_3$ followed by brine, dried over Na_2SO_4, and volatiles were removed in vacuo. Purification by preparatory thin layer chromatography (30% EtOAc/hexanes) afforded the title compound as a clear oil (16 mg, 0.05 mmol, 41% yield).

^{1}H NMR (400 MHz, CDCl$_3$): δ 7.43 – 7.29 (m, 5H), 5.22 – 5.10 (m, 2H), 2.97 (ddd, *J* = 19.0, 2.5, 1.4 Hz, 1H), 2.83 – 2.61 (m, 2H), 2.41 – 2.32 (m, 2H), 2.28 – 2.09 (m, 3H), 2.05 – 1.93 (m, 1H), 1.81 – 1.56 (m, 5H), 1.40 – 1.26 (m, 1H).

^{13}C NMR (100 MHz, CDCl$_3$): δ 173.9, 172.9, 135.7, 128.8, 128.6, 128.3, 88.4, 66.9, 47.8, 46.5, 39.6, 39.1, 32.8, 30.4, 29.7, 24.9, 22.7.

IR (Neat Film, NaCl): 2943, 2873, 1722, 1255, 1189, 1167 cm^{-1}.

HRMS (MM: FD+): *m/z* calc'd for $C_{19}H_{22}O_4$ $[M]^+$: 314.1518, found 314.1521.

Optical Rotation: $[\alpha]_D^{21}$ –15.8 (c 1.00, $CHCl_3$).

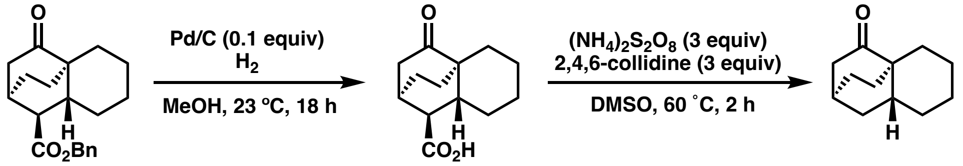

(2*S*,4a*R*,8a*R*)-octahydro-2*H*-2,4a-ethanonaphthalen-9-one (45)

A vial containing ketone **11f** (0.72 mmol, 1 equiv) and Pd/C (10 wt. % with 67% H_2O, 0.072 mmol, 0.1 equiv) was evacuated and backfilled with H_2. Methanol (1.06 mL) was subsequently added, and the reaction was stirred at 23 ºC overnight. The crude reaction mixture was filtered through a silica plug and concentrated under reduced pressure to afford acid **S11** as a white solid, which was used without further purification.

To a solution of acid **S11** (0.62 mmol, 1 equiv) in DMSO (1.24 mL) was added $(NH_4)_2S_2O_8$ (1.86 mmol, 3 equiv) and 2,4,6-collidine (1.86 mmol, 3 equiv). The mixture was purged with N_2 for 5 min and was subsequently sealed and heated to 60 ºC for 2 h with stirring. The reaction mixture was diluted with dichloromethane, washed with brine (1x), and the aqueous layer was extracted with dichloromethane (3x). The combined organic layers were washed with brine (3x), dried over Na_2SO_4, and concentrated under reduced pressure. Purification by flash column chromatography (15% EtOAc/hexanes) afforded the title compound as a white solid (30.2 mg, 0.167 mmol, 27% yield).

^{1}H NMR (400 MHz, $CDCl_3$): δ 2.33 – 2.21 (m, 3H), 2.13 – 2.07 (m, 1H), 1.93 (dddd, *J* = 13.3, 10.6, 3.8, 2.8 Hz, 1H), 1.75 – 1.58 (m, 6H), 1.53 (dd, *J* = 13.6, 4.0 Hz, 1H), 1.47 – 1.38 (m, 2H), 1.38 – 1.28 (m, 1H), 1.27 – 1.17 (m, 1H), 1.17 – 1.05 (m, 2H).

^{13}C NMR (100 MHz, CDCl$_3$): δ 218.6, 44.9, 43.9, 34.5, 34.1, 30.6, 29.0, 27.7, 25.9, 25.9, 21.8, 21.4.

IR (Neat Film, NaCl): 2925, 1716 cm^{-1}

HRMS (MM: FI+): *m/z* calc'd for $C_{12}H_{18}O$ [M]$^+$: 178.1358, found 178.1359.

Optical Rotation: $[\alpha]_D^{21}$ –59.9 (c 0.89, $CHCl_3$).

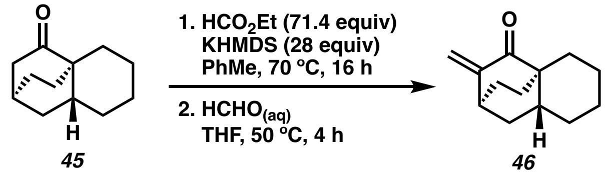

(2*S*,4a*R*,8a*R*)-10-methyleneoctahydro-2*H*-2,4a-ethanonaphthalen-9-one (46)

To a solution of ketone **45** (0.056 mmol, 1 equiv) and ethyl formate (4 mmol, 71.4 equiv) in toluene (3.2 mL) was added a solution of KHMDS (0.5 M in toluene, 1.6 mmol, 28 equiv) at 23 ºC. The reaction mixture was stirred at 70 ºC for 16 h. Upon cooling to 0 ºC, THF (6.4 mL) and formalin (37% in water, 3.2 mL) was added, and then the reaction was heated to 50 ºC for 4 h. The reaction mixture was diluted with a saturated aqueous solution of NH_4Cl, extracted with EtOAc (3x), dried over Na_2SO_4, and concentrated under reduced pressure. Purification by preparatory thin layer chromatography (30% EtOAc/hexanes, 2x) afforded the title compound as a yellow oil (4.4 mg, 0.023 mmol, 41% yield).

^{1}H NMR (400 MHz, CDCl$_3$): δ 5.93 (d, *J* = 1.8 Hz, 1H), 5.15 (d, *J* = 1.8 Hz, 1H), 2.66 (p, *J* = 3.0 Hz, 1H), 2.39 – 2.19 (m, 1H), 1.98 (dddd, *J* = 13.2, 10.6, 3.9, 2.8 Hz, 1H), 1.81 – 1.61 (m, 6H), 1.51 – 1.42 (m, 2H), 1.41 – 1.31 (m, 2H), 1.23 – 1.15 (m, 2H), 1.11 (dt, *J* = 12.9, 3.6 Hz, 1H).

^{13}C NMR (100 MHz, CDCl$_3$): δ 205.3, 147.9, 116.3, 45.0, 36.1, 35.0, 34.7, 30.8, 29.0, 26.5, 26.0, 21.6, 21.3.

IR (Neat Film, NaCl): 2926, 2859, 1708, 1630, 1464, 1449 cm^{-1}

HRMS (MM: FD+): *m/z* calc'd for $C_{13}H_{18}O$ $[M]^+$: 190.1358, found 190.1353.

Optical Rotation: $[\alpha]_D^{21}$ –34.2 (c 0.29, $CHCl_3$).

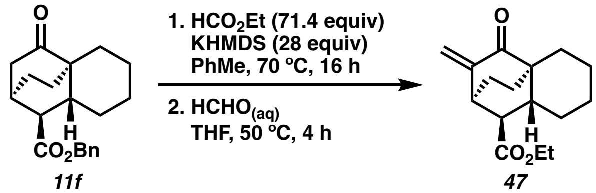

ethyl (1*S*,2*S*,4a*R*,8a*R*)-3-methylene-4-oxooctahydro-2*H*-2,4a-ethanonaphthalene-1-carboxylate (47)

To a solution of **11f** (0.192 mmol, 1 equiv) and ethyl formate (13.71 mmol, 71.4 equiv) in toluene (11.1 mL) was added a solution of KHMDS (0.5 M in toluene, 5.49 mmol, 28 equiv) at 23 ºC. The reaction mixture was stirred at 70 ºC for 16 h. Upon cooling to 0 ºC, THF (22.2 mL) and formalin (37% in water, 11.1 mL) was added, and then the reaction was heated to 50 ºC for 4 h. The reaction mixture was diluted with a saturated aqueous solution of NH_4Cl, extracted with EtOAc (3x), dried over Na_2SO_4, and concentrated under reduced pressure. Purification by preparatory thin layer chromatography (30% EtOAc/hexanes, 2x) afforded the title compound as a yellow oil (13.1 mg, 0.05 mmol, 26% yield).

^{1}H NMR (400 MHz, $CDCl_3$): δ 5.99 (d, *J* = 1.7 Hz, 1H), 5.17 (d, *J* = 1.7 Hz, 1H), 4.21 – 4.01 (m, 2H), 3.04 (td, *J* = 3.1, 2.0 Hz, 1H), 2.27 (ddd, *J* = 14.2, 11.4, 5.4 Hz, 1H), 2.21 (dd, *J* = 6.8, 2.0 Hz, 1H), 2.01 (dddd, *J* = 11.6, 6.5, 4.4, 1.7 Hz, 1H), 1.92 – 1.75 (m, 3H), 1.73 – 1.65 (m, 2H), 1.56 – 1.50 (m, 1H), 1.47 – 1.31 (m, 2H), 1.29 – 1.16 (m, 6H).

^{13}C NMR (100 MHz, $CDCl_3$): δ 203.7, 173.8, 144.3, 119.0, 60.8, 51.1, 45.0, 39.1, 37.2, 30.3, 28.9, 26.2, 25.8, 21.3, 21.1, 14.4.

IR (Neat Film, NaCl): 2927, 2867, 1732, 1708, 1449, 1180 cm^{-1}.

HRMS (MM: FD+): *m/z* calc'd for $C_{16}H_{22}O_3$ $[M]^+$: 262.1569, found 262.1576.

Optical Rotation: $[\alpha]_D^{21}$ –3.9 (c 0.38, $CHCl_3$).

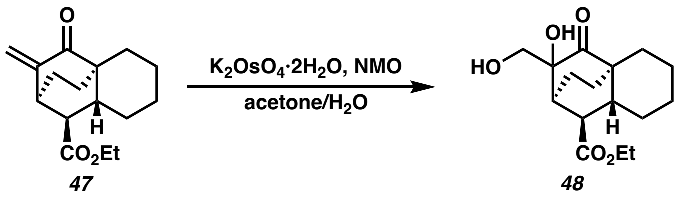

ethyl (1*R*,2*S*,4a*R*,8a*R*)-3-hydroxy-3-(hydroxymethyl)-4-oxooctahydro-2*H*-2,4a-ethanonaphthalene-1-carboxylate (48)

A flame dried vial was charged with enone **47** (14.7 mg, 0.056 mmol, 1 equiv) and acetone (2.3 mL, 0.024 M) and water (0.6 mL, 0.094 M). NMO (50 wt. % in H_2O) (23 µL, 0.112 mmol, 2 equiv) was added and the solution was cooled to 0 ºC. $K_2OsO_4 \cdot 2H_2O$ (2.1 mg, 0.006 mmol, 0.1 equiv) was added to the solution. The resultant solution was slowly warmed to 23 ºC. Upon complete consumption of starting material (as determined by TLC), the reaction was quenched with a saturated solution of $Na_2S_2O_3$ and stirred for 30 min. The mixture was then diluted with CH_2Cl_2 and the product was extracted with CH_2Cl_2 (2 x). The combined organic layers were washed with brine, dried over Na_2SO_4, filtered, and concentrated under reduced pressure. Purification by preparatory thin layer chromatography (50% EtOAc/hexanes) afforded the title compound as a colorless oil (2.1 mg, 0.007 mmol,13% yield, 10:1 dr). In the ^{1}H NMR, peaks that correspond to the minor diastereomer closely resemble the major diastereomer. dr was determine through integration of 1H NMR peaks 2.69 ppm (major) and 2.96 ppm (minor).

^{1}H NMR (400 MHz, $CDCl_3$): δ 4.15 (q, *J* = 7.1 Hz, 2H), 3.81 (dd, *J* = 11.8, 2.6 Hz, 1H), 3.54 (dd, *J* = 11.9, 2.7 Hz, 1H), 2.71 – 2.67 (m, 1H), 2.38 – 2.24 (m, 2H), 2.10 (d, *J* = 7.3 Hz, 1H), 1.94 – 1.64 (m, 7H), 1.47 – 1.18 (m, 9H).

^{13}C NMR (100 MHz, $CDCl_3$): δ 218.4, 174.8, 75.8, 65.8, 61.0, 48.9, 45.4, 37.3, 37.2, 30.3, 28.3, 25.6, 22.5, 21.0, 14.3.

IR (Neat Film, NaCl): 3468, 2930, 2355, 1716, 1197, 1033 cm^{-1}.

HRMS (MM: FD+): *m/z* calc'd for $C_{16}H_{24}O_5$ $[M]^+$: 296.1624, found 296.1619.

Optical Rotation: $[\alpha]_D^{21}$ 2.1 (c 0.24, $CHCl_3$).

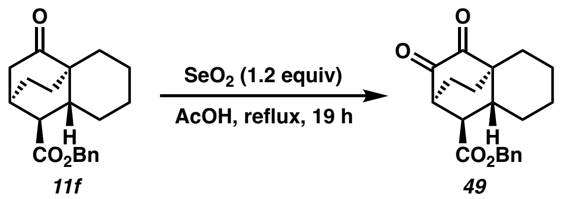

benzyl (1*R*,2*S*,4a*R*,8a*R*)-3,4-dioxooctahydro-2*H*-2,4a-ethanonaphthalene-1-carboxylate (49) To a solution of **11f** (0.064 mmol, 1 equiv) in glacial acetic acid (0.1 mL) was added SeO_2 (0.077 mmol, 1.2 equiv). The reaction was brought to reflux for 19 h. After cooling to 23 ºC, the reaction mixture was filtered and concentrated under reduced pressure. The resulting crude mixture was dissolved in EtOAc and washed with water (5x), dried over Na_2SO_4, and concentrated under reduced pressure. Purification by preparatory thin layer chromatography (30% EtOAc/hexanes) afforded the title compound as a yellow oil (10.4 mg, 0.031 mmol, 48% yield).

^{1}H NMR (400 MHz, CDCl$_3$): δ 7.41 – 7.28 (m, 5H), 5.19 – 5.04 (m, 2H), 3.00 (m, 1H), 2.54 (dd, *J* = 6.4, 2.3 Hz, 1H), 2.40 (ddd, *J* = 14.6, 10.3, 6.9 Hz, 1H), 2.02 (m, 3H), 1.80 – 1.69 (m, 2H), 1.68 – 1.50 (m, 3H), 1.48 – 1.38 (m, 1H), 1.33 (dt, *J* = 13.4, 3.5 Hz, 1H), 1.29 – 1.15 (m, 2H).

^{13}C NMR (100 MHz, CDCl$_3$): δ 198.7, 196.1, 173.2, 135.4, 128.8, 128.6, 128.4, 67.4, 49.9, 48.0, 46.3, 37.9, 30.2, 28.3, 25.5, 22.7, 20.5, 20.2.

IR (Neat Film, NaCl): 2932, 2857, 1731, 1454, 1155 cm^{-1}.

HRMS (MM: FD+): *m/z* calc'd for $C_{20}H_{22}O_4$ $[M]^+$: 326.1518, found 326.1532.

Optical Rotation: $[\alpha]_D^{21}$ –49.2 (c 1.04, $CHCl_3$).

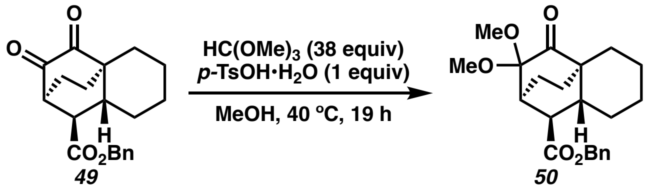

benzyl (1*R*,2*S*,4a*R*,8a*R*)-3,3-dimethoxy-4-oxooctahydro-2*H*-2,4a-ethanonaphthalene-1-carboxylate (50)

To a solution of diketone **49** (0.031 mmol, 1 equiv) in methanol (0.13 mL) was added $HC(OMe)_3$ (1.2 mmol, 38 equiv) and *p*-TsOH•H_2O (0.031 mmol, 1 equiv). The reaction was stirred for 19 h at 40 ºC, followed by dilution with a saturated aqueous solution of $NaHCO_3$. The aqueous layer was extracted with EtOAc (4x), dried over Na_2SO_4, and concentrated under reduced pressure. Purification by preparatory thin layer chromatography (30% EtOAc/hexanes) afforded the title compound as a yellow oil (4.7 mg, 0.013 mmol, 41% yield).

^{1}H NMR (400 MHz, $CDCl_3$): δ 7.42 – 7.29 (m, 5H), 5.26 (d, *J* = 12.3 Hz, 1H), 5.02 (d, *J* = 12.3 Hz, 1H), 3.20 (s, 3H), 3.06 (s, 3H), 2.93 (dt, *J* = 4.2, 2.2 Hz, 1H), 2.29 – 2.11 (m, 3H), 1.98 – 1.89 (m, 1H), 1.83 (dddd, *J* = 13.8, 11.4, 6.7, 2.4 Hz, 1H), 1.70 – 1.58 (m, 4H), 1.54 (d, *J* = 3.9 Hz, 1H), 1.31 (dd, *J* = 10.8, 2.4 Hz, 1H), 1.27 – 1.19 (m, 3H).

^{13}C NMR (100 MHz, $CDCl_3$): δ 209.0, 173.5, 136.5, 128.6, 128.5, 128.2, 97.4, 66.4, 50.4, 48.8, 48.4, 45.5, 36.4, 34.9, 30.0, 29.1, 25.5, 23.1, 21.0, 21.0.

IR (Neat Film, NaCl): 2933, 2855, 1736, 1449, 1172 cm^{-1}

HRMS (MM: FD+): *m/z* calc'd for $C_{22}H_{28}O_5$ [M]$^+$: 372.1937, found 372.1931.

Optical Rotation: $[\alpha]_D^{21}$ –26.2 (c 0.42, $CHCl_3$).

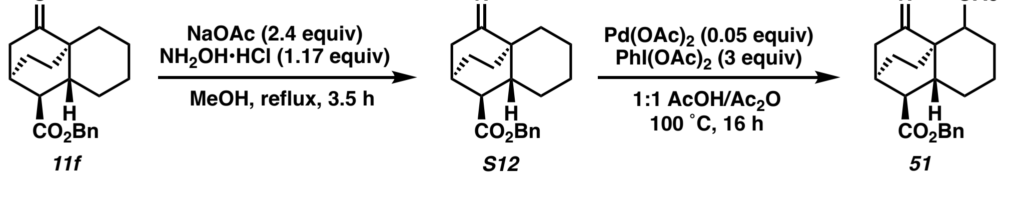

benzyl (1*S*,2*R*,4a*S*,8a*R*,*E*)-5-acetoxy-4-(acetoxyimino)octahydro-2*H*-2,4a-ethanonaphthalene-1-carboxylate (51)

To a stirred solution of ketone **11f** (0.096 mmol, 1 equiv) in methanol (0.93 mL) was added NaOAc (0.23 mmol, 2.4 equiv), NH_2OH•HCl (0.111 mmol, 1.17 equiv), and water (0.033 mL). The reaction was brought to reflux for 3.5 h and was subsequently cooled to 23 ºC and concentrated under reduced pressure. The crude mixture was then diluted with water and extracted with EtOAc (3x), washed with a saturated aqueous solution of $NaHCO_3$ and brine, dried with Na_2SO_4, and concentrated under reduced pressure to afford oxime **S12**.

Oxime **S12** (0.096 mmol, 1 equiv) was dissolved in a 1:1 mixture of AcOH/Ac_2O (0.78 mL). The reaction vessel was sealed and stirred at 23 ºC for 2 h. $Pd(OAc)_2$ (0.0048 mmol, 0.05 equiv) and $PhI(OAc)_2$ (0.288 mmol, 3 equiv) were subsequently added, and the reaction was heated to 100 ºC for 16 h. The reaction mixture was cooled to 23 ºC, filtered through a silica plug, and the filtrate was diluted with EtOAc. The organic layer was washed with a saturated solution of $NaHCO_3$ until not acidic, washed with brine, dried over Na_2SO_4, and concentrated under reduced pressure. Purification by preparatory thin layer chromatography (30% EtOAc/hexanes) afforded the title compound as a yellow oil (12.3 mg, 0.0288 mmol, 30% yield over two steps).

^{1}H NMR (400 MHz, $CDCl_3$): δ 7.43 – 7.27 (m, 5H), 5.31 – 5.20 (m, 1H), 5.20 – 5.02 (m, 2H), 2.62 (dt, *J* = 20.2, 3.5 Hz, 1H), 2.40 – 2.29 (m, 2H), 2.27 – 2.17 (m, 1H), 2.14 (s, 3H), 2.02 (s, 3H), 1.89 (m, 1H), 1.83 – 1.69 (m, 4H), 1.62 (t, *J* = 2.8 Hz, 2H), 1.46 – 1.28 (m, 4H).

^{13}C NMR (100 MHz, $CDCl_3$): δ 173.9, 170.6, 170.4, 168.9, 135.9, 128.8, 128.5, 128.2, 71.7, 66.9, 49.6, 42.2, 39.1, 29.5, 28.9, 28.6, 26.9, 25.5, 23.0, 21.4, 20.2, 16.7.

IR (Neat Film, NaCl): 2930, 1764, 1731, 1456, 1371, 1248, 1210 cm^{-1}.

HRMS (MM: FD+): *m/z* calc'd for $C_{24}H_{29}NO_6$ $[M]^+$: 427.1995, found 427.2017.

Optical Rotation: $[\alpha]_D^{21}$ –27.6 (c 1.00, $CHCl_3$).

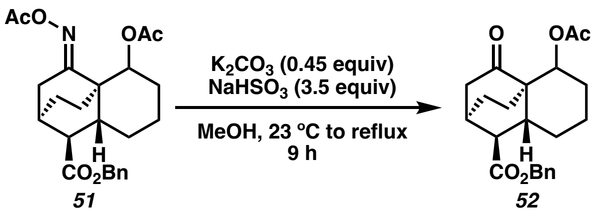

benzyl (1*S*,2*R*,4a*R*,8a*R*)-5-acetoxy-4-oxooctahydro-2*H*-2,4a-ethanonaphthalene-1-carboxylate (52)

To a solution of **51** (0.029 mmol, 1 equiv) in methanol (0.06 mL) in a loosely capped vial was added K_2CO_3 (0.013 mmol, 0.45 equiv) at 23 ºC in three portions over 6 h. $NaHSO_3$ (0.1 mmol, 3.5 equiv) and water (0.06 mL) were subsequently added, and the vial was sealed and heated to 80 ºC for 3 h. The reaction mixture was diluted with $CHCl_3$, rinsed with 1 M HCl, and the aqueous layer was extracted with $CHCl_3$ (3x). The combined organic layers were neutralized with a saturated solution of $NaHCO_3$, washed with brine, dried over Na_2SO_4, and concentrated under reduced pressure. Purification by preparatory thin layer chromatography (35% EtOAc/hexanes) afforded the title compound as a colorless oil (1.8 mg, 0.0056 mmol, 19% yield).

^{1}H NMR (400 MHz, $CDCl_3$): δ 7.41 – 7.30 (m, 5H), 5.19 – 5.06 (m, 3H), 2.48 (m, 1H), 2.41 (dt, *J* = 19.2, 2.8 Hz, 1H), 2.29 (dt, *J* = 6.9, 2.0 Hz, 1H), 2.16 – 2.04 (m, 3H), 1.98 (s, 3H), 1.94 – 1.89 (m, 1H), 1.87 – 1.79 (m, 3H), 1.78 – 1.73 (m, 1H), 1.71 (m, 2H), 1.46 – 1.29 (m, 3H).

^{13}C NMR (100 MHz, $CDCl_3$): δ 212.2, 173.9, 170.2, 135.9, 128.8, 128.5, 128.3, 70.8, 66.9, 49.4, 48.5, 40.5, 37.9, 30.7, 29.0, 26.7, 25.8, 22.9, 21.3, 15.7.

IR (Neat Film, NaCl): 2928, 1781, 1375, 1246, 1173 cm^{-1}.

HRMS (MM: FD+): *m/z* calc'd for $C_{22}H_{26}O_5$ $[M]^+$: 370.1780, found 370.1774.

Optical Rotation: $[\alpha]_D^{21}$ –18.3 (c 0.18, $CHCl_3$).

Preparation of Additional Compounds

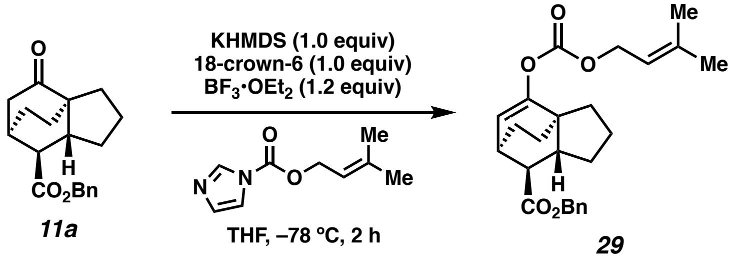

benzyl (3a*R*,6*R*,7*S*,7a*R*)-4-oxooctahydro-3a,6-ethanoindene-7-carboxylate (29)[17]

To a solution of KHMDS (40 mg, 0.20 mmol, 1.0 equiv) and 18-crown-6 (53 mg, 0.20 mmol, 1 equiv) in THF (2.0 mL) at –78 ºC was added a solution of **11a** (60 mg, 0.20 mmol, 1 equiv). Stirring was continued at –78 ºC for 30 minutes, then a pre-mixed solution of 3-methylbut-2-en-1-yl 1*H*-imidazole-1-carboxylate (43 mg, 0.24 mmol, 1.2 equiv) and boron trifluoride diethyl etherate (30 µL, 0.24 mmol, 1.2 equiv) in THF (1.2 mL) was added dropwise. After two additional hours of stirring at –78 ºC, EtOAc and saturated aqueous NH_4Cl were added. The layers were separated, and the aqueous layer was extracted twice with EtOAc. The combined organic layers were washed with brine, dried over Na_2SO_4, filtered, and solvent was removed in vacuo. The crude mixture was purified by silica gel flash column chromatography (0–40% EtOAc/hexanes) to afford enol carbonate **29** as a colorless oil (44 mg, 0.11 mmol, 55% yield).

^{1}H NMR (400 MHz, $CDCl_3$): δ 7.39 – 7.29 (m, 5H), 5.70 (d, *J* = 6.8 Hz, 1H), 5.42 – 5.36 (m, 1H), 5.09 (d, *J* = 3.6 Hz, 2H), 4.65 (d, *J* = 7.3 Hz, 2H), 3.08 – 2.96 (m, 1H), 2.25 (d, *J* = 5.6 Hz, 1H), 2.07 – 1.97 (m, 2H), 1.91 – 1.79 (m, 3H), 1.77 (s, 3H), 1.73 (s, 3H), 1.63 – 1.54 (m, 2H), 1.52 – 1.38 (m, 3H), 1.35 – 1.28 (m, 1H).

^{13}C NMR (100 MHz, $CDCl_3$): δ 174.9, 155.6, 153.5, 140.6, 136.4, 128.7, 128.2, 128.0, 117.9, 112.8, 66.3, 65.3, 51.3, 48.1, 48.1, 35.5, 28.4, 28.4, 27.3, 25.9, 24.6, 22.8, 18.3.

IR (Neat Film, NaCl): 2953, 2870, 1754, 1735, 1241, 1226, 1150 cm^{-1}

HRMS (MM: FD+): *m/z* calc'd for $C_{25}H_{30}O_5$ $[M]^+$: 410.2093, found 410.2094.

SFC conditions: 20% IPA, 2.5 mL/min, Chiralpak IC column, λ = 210 nm, t_R (min): minor = 3.05, major = 3.40.

Prepared from ***(±)-11a***:

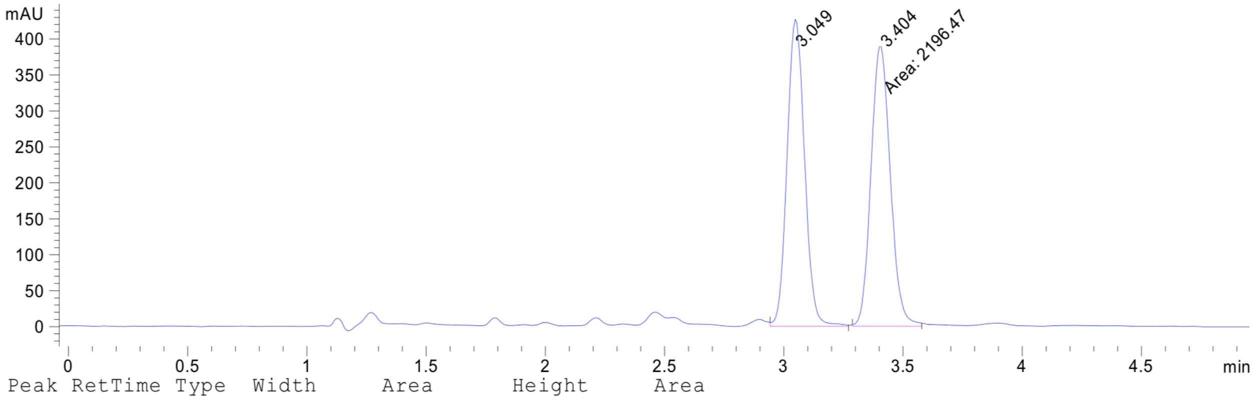

Peak #	RetTime [min]	Type	Width [min]	Area [mAU*s]	Height [mAU]	Area %
1	3.049	VV	0.0796	2180.62427	426.89822	49.8190
2	3.404	FM	0.0935	2196.47119	391.35312	50.1810
Totals :				4377.09546	818.25134	

Prepared from enantioenriched ***11a*** *(87% ee)*:

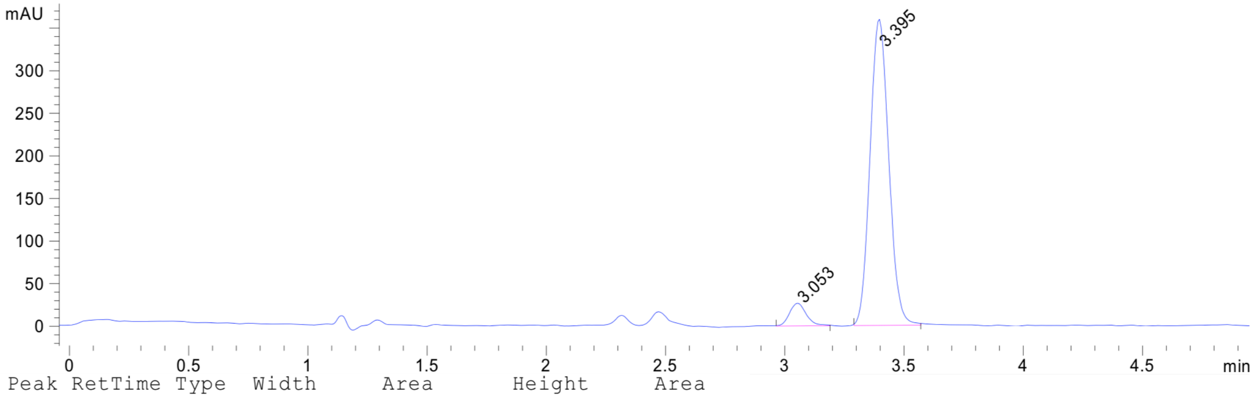

Peak #	RetTime [min]	Type	Width [min]	Area [mAU*s]	Height [mAU]	Area %
1	3.053	BB	0.0775	132.07602	26.78439	6.3039
2	3.395	BB	0.0857	1963.08484	359.75577	93.6961
Totals :				2095.16086	386.54016	

Determination of Absolute and Relative Stereochemistry by VCD

Experimental Protocol: A solution of the compound of interest (50 mg/mL) in $CDCl_3$ was loaded into a front-loading SL-4 cell (International Crystal Laboratories) possessing BaF_2 windows and a

100 mm path length. Infrared (IR) and VCD spectra were acquired on a BioTools ChiralIR-2X VCD spectrometer as a set of 24 one-hour blocks (24 blocks, 3120 scans per block) in dual PEM mode. A 15-minute acquisition of neat (+)-α-pinene control yielded a VCD spectrum in agreement with literature spectra. IR and VCD spectra were background corrected using a 30-minute block IR acquisition of the empty instrument chamber under gentle N_2 purge, and were solvent corrected using a 16-hour (16 blocks, 3120 scans per block) IR/VCD acquisition of $CDCl_3$ in the same 100 µm BaF2 cell. The reported spectra represent the result of block averaging.

Both enantiomers of compounds **11a**, **11p**, and **11p'** were prepared from the *(S)* and *(R)* enantiomers of the *t*-BuPHOX ligand. Data were collected for both enantiomers of compounds at identical concentration and the final reported VCD spectra are the half-difference of the spectra of the compounds derived from the *(S)* minus *(R)* enantiomer of ligand.

Due to limited sample size, spectra of cycloadducts **11j'** and **11j''** were collected at concentrations of 11.7 and 9.3 mg/mL, respectively.

Computational Protocol: An arbitrarily chosen enantiomer of the compound of interest was subjected to an exhaustive initial molecular mechanics-based conformational search (OPLS_2005 force field, $CHCl_3$ solvent, 10.0 kcal/mol cutoff, "Enhanced" torsional sampling) as implemented in MacroModel program.[25] The resulting ensemble of conformers was subsequently optimized using the B3PW91 functional, cc-pVTZ(-f) basis, and implicit PBF solvation model for chloroform using the Jaguar program.[26] Harmonic frequencies computed at the B3PW91/cc-pVTZ(-f)/PBF(chloroform) level were scaled by 0.98. The resultant structurally unique conformers possessing all positive Hessian eigenvalues were Boltzmann weighted by relative free energy at 298.15 K. The predicted IR and VCD frequencies and intensities of the retained conformers were convolved using Lorentzian line shapes (γ = 4 cm^{-1}) and summed using the respective Boltzmann weights to yield the final predicted IR and VCD spectra. The predicted VCD of the opposite enantiomer was generated by inversion of sign.

VCD Analysis for diastereomers 11a, 11p, and 11p'

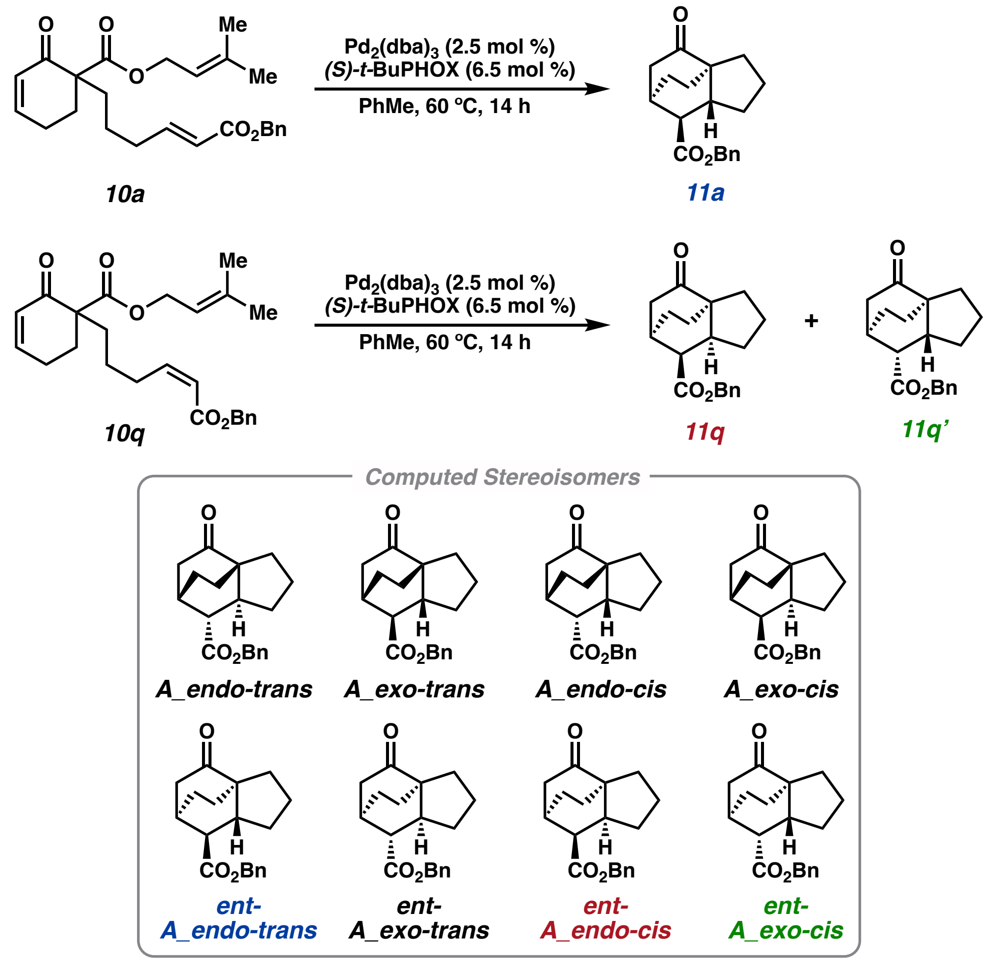

Figure S1. Three diastereomers **11a**, **11q**, and **11q'** to be compared to spectra computed from all eight possible stereoisomers.

*Comparisons between computed and experimental spectra for isomer **11a**:*

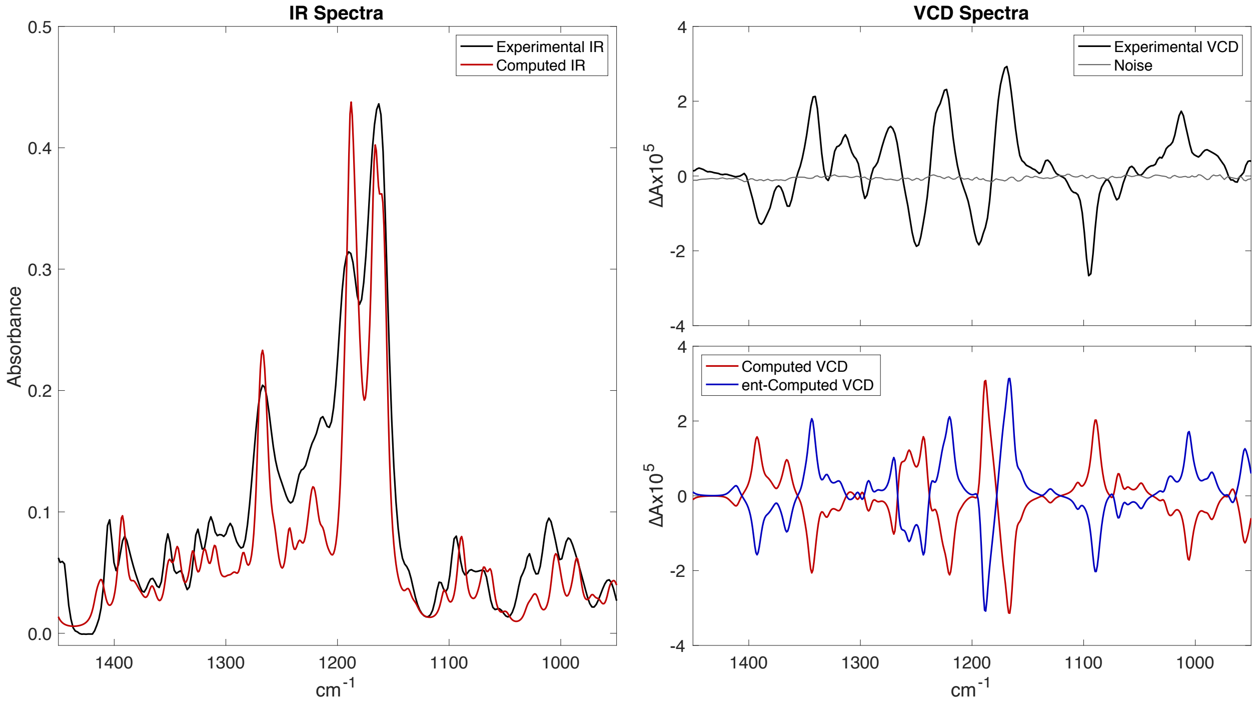

Figure S2. Comparison of experimental VCD and IR spectra for product **11a** to computed spectra for **A_endo-trans**. Experimental IR spectrum in good agreement with computed spectrum. Experimental VCD spectrum for **10a** is in excellent agreement with computed spectrum for ***ent*-A_endo-trans**.

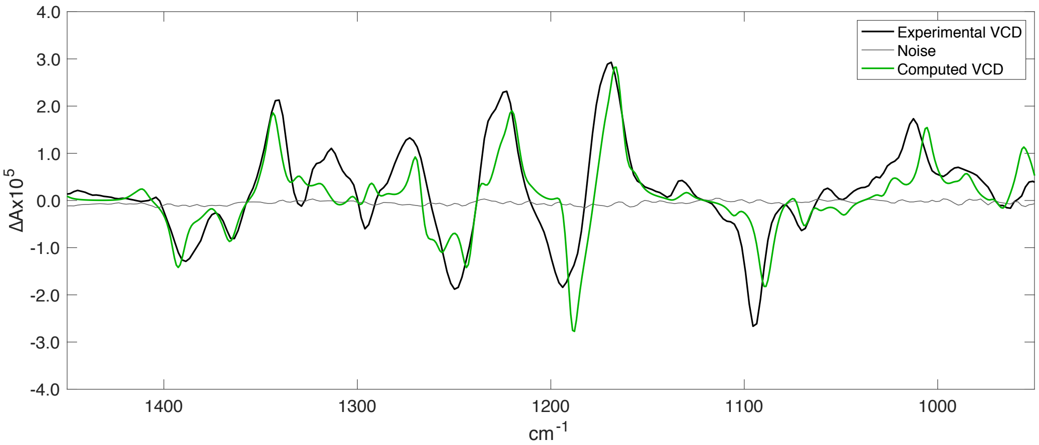

Figure S3. Overlayed experimental and calculated VCD spectra for **11a** – assigned as ***ent*-A_endo-trans**.

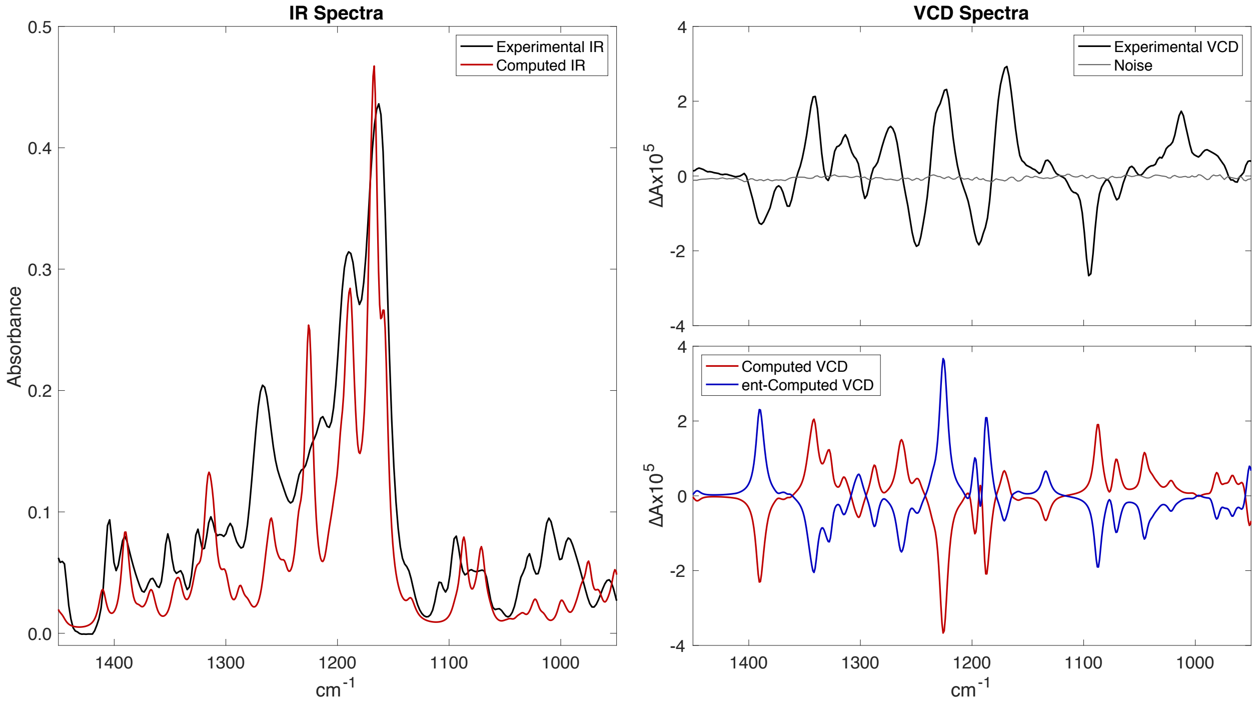

Figure S4. Comparison of experimental VCD and IR spectra for product **11a** to computed spectra for **A_exo-trans**. A shift of -3 cm^{-1} along x-axis applied to computed spectra in fitting. Experimental data from **11a** do not match computed data of **A_exo-trans**.

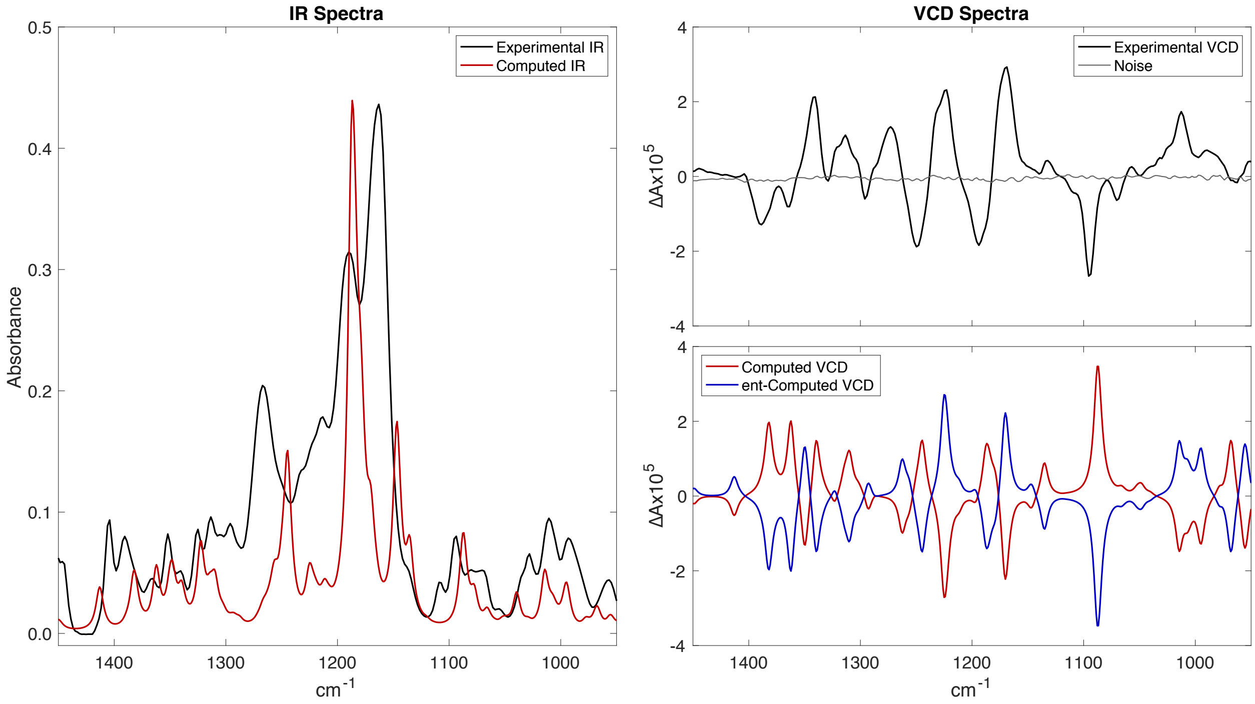

Figure S5. Comparison of experimental VCD and IR spectra for product **11a** to computed spectra for **A_endo-cis**. Experimental data from **11a** do not match computed data of **A_endo-cis**.

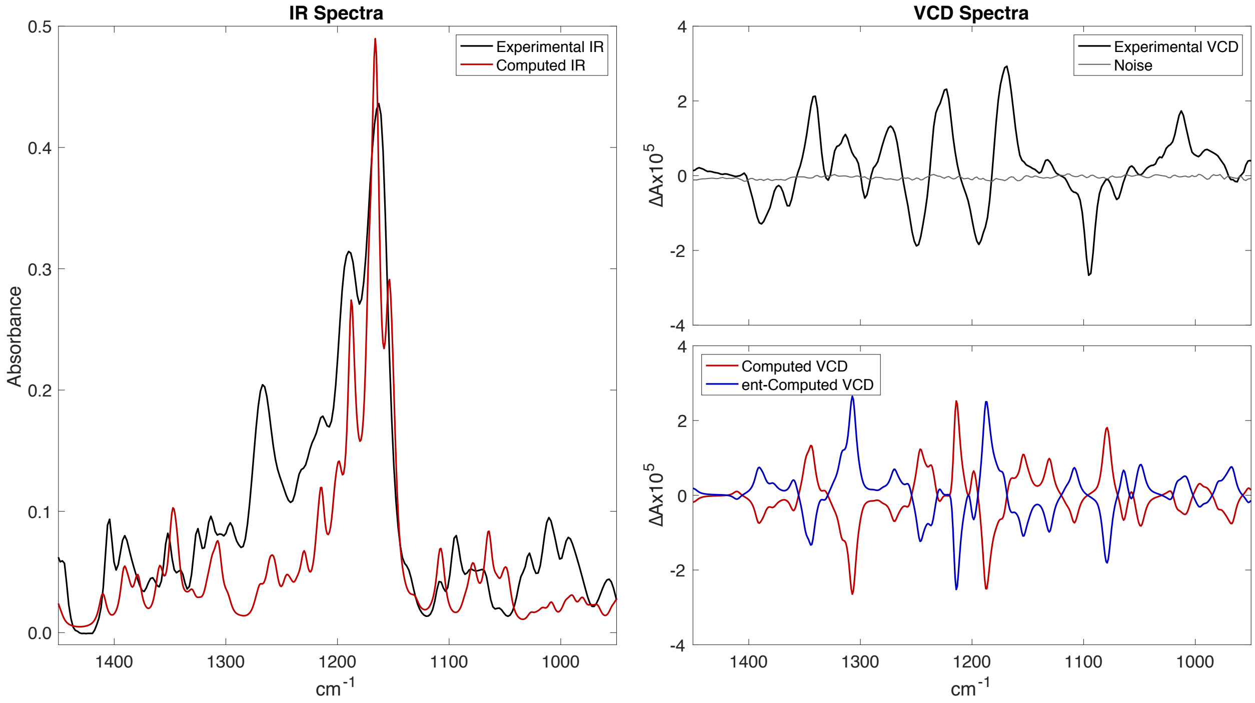

Figure S6. Comparison of experimental VCD and IR spectra for product **11a** to computed spectra for **A_exo-cis**. A shift of -3 cm^{-1} along x-axis applied to computed spectra in fitting. Experimental data from **11a** do not match computed data of **A_exo-cis**.

*Comparisons between computed and experimental spectra for isomer **11q**:*

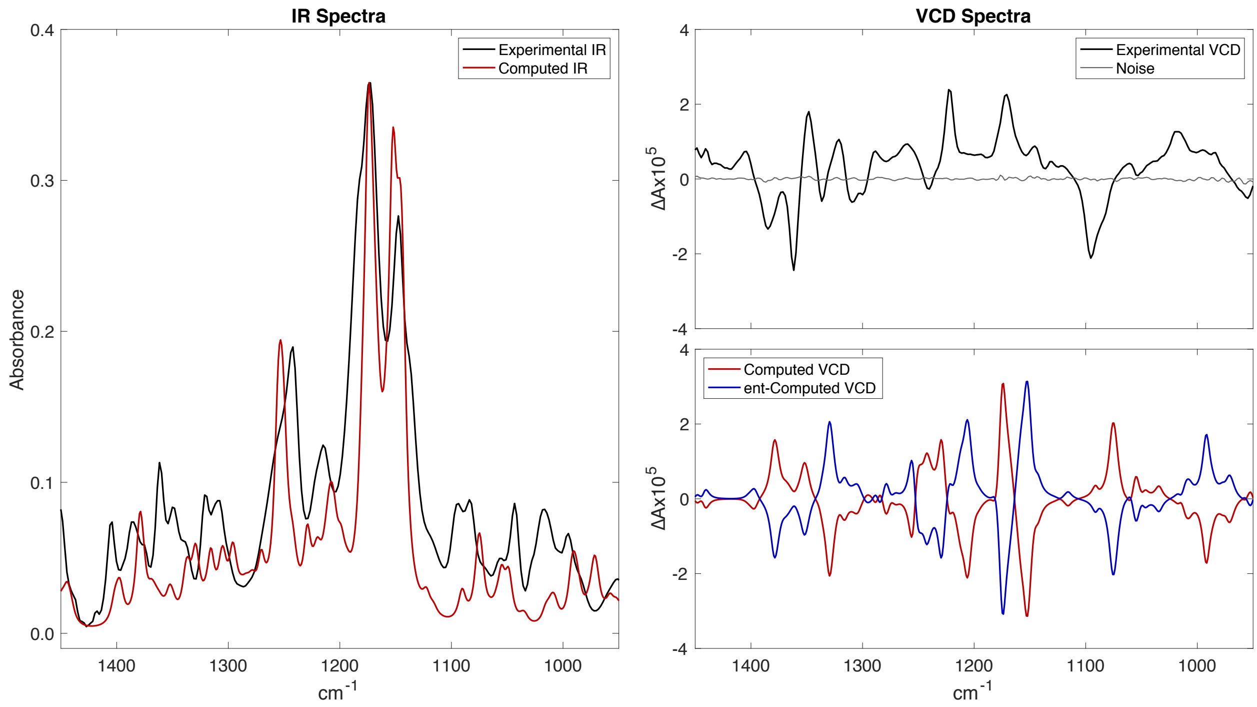

Figure S7. Comparison of experimental VCD and IR spectra for product **11q** to computed spectra for **A_endo-trans**. A shift of +14 cm^{-1} along x-axis applied to computed spectra in fitting. The IR spectrum of **Y** contains similar features to the calculated spectrum for **A_endo-trans**; however, the VCD spectrum displays large discrepancies at 1174, 1152, 1330, 1076, and 992 cm^{-1}. **11q** is <u>not</u> assigned as **A_endo-trans**.

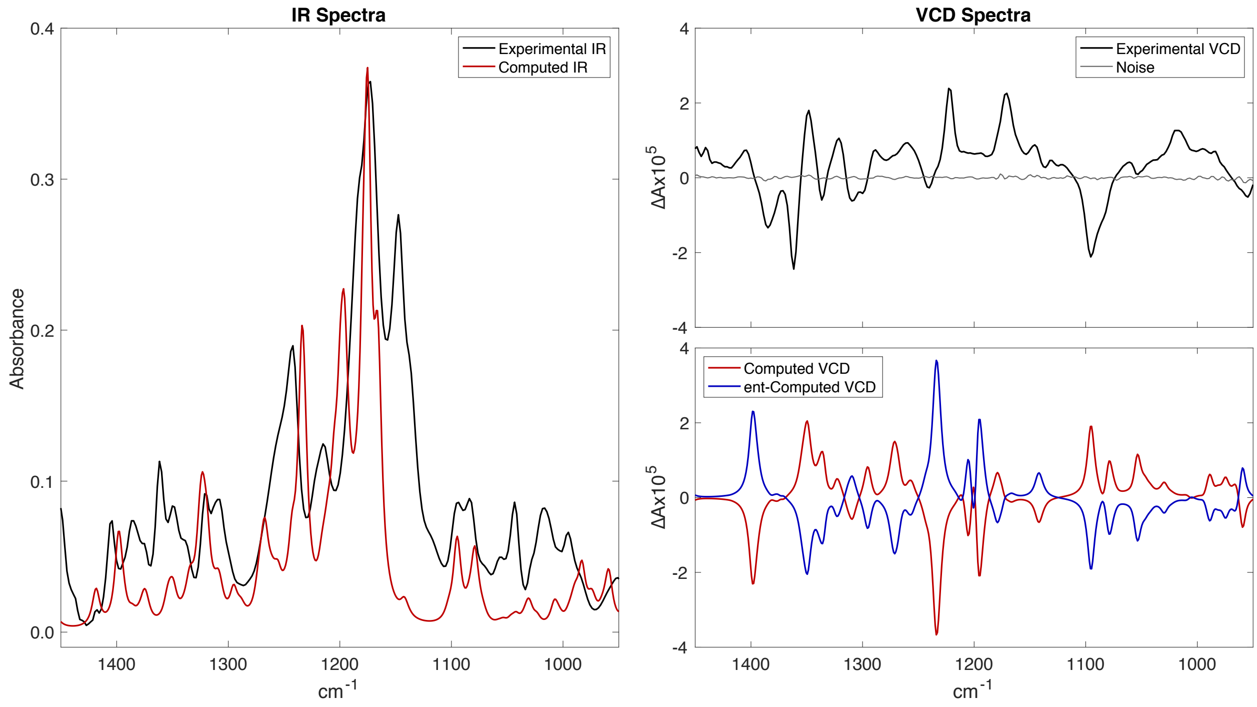

Figure S8. Comparison of experimental VCD and IR spectra for product **11q** to computed spectra for **A_exo-trans**. Experimental data from **11q** do not match computed data of **A_exo-trans**.

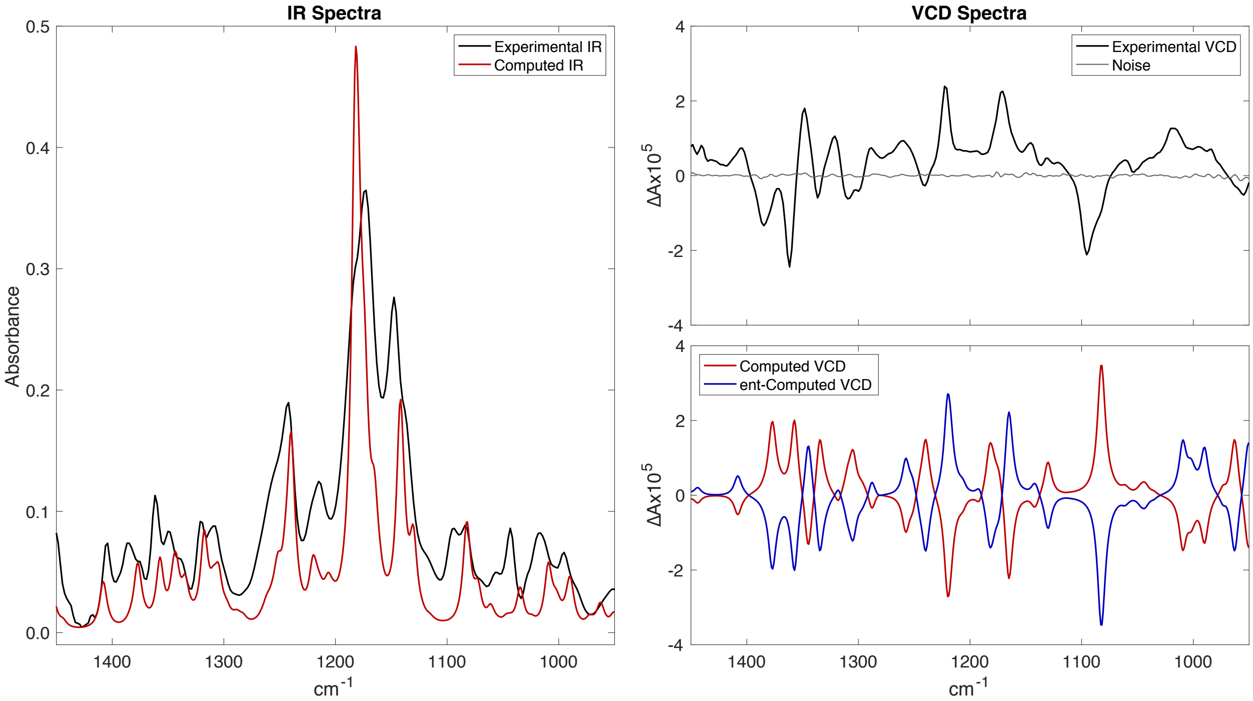

Figure S9. Comparison of experimental VCD and IR spectra for product **11q** to computed spectra for **A_endo-cis**. A shift of +5 cm^{-1} along x-axis applied to computed spectra in fitting. The IR spectrum of **11q** is in good agreement with that of the computed IR spectrum of **A_endo-cis**. Experimental VCD spectrum for **11q** is in excellent agreement with computed spectrum for ***ent*-A_endo-cis**.

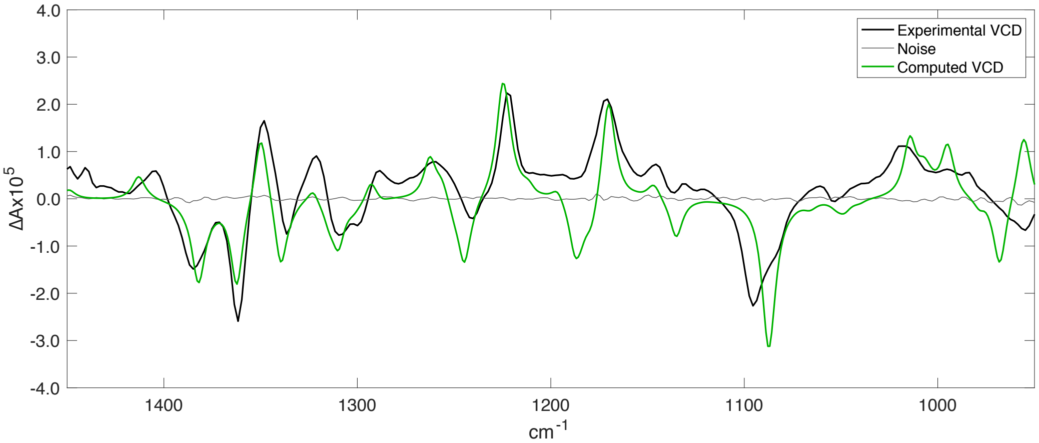

Figure S10. Overlayed experimental and calculated VCD spectra for **11q** – assigned as ***ent*-A_endo-cis**.

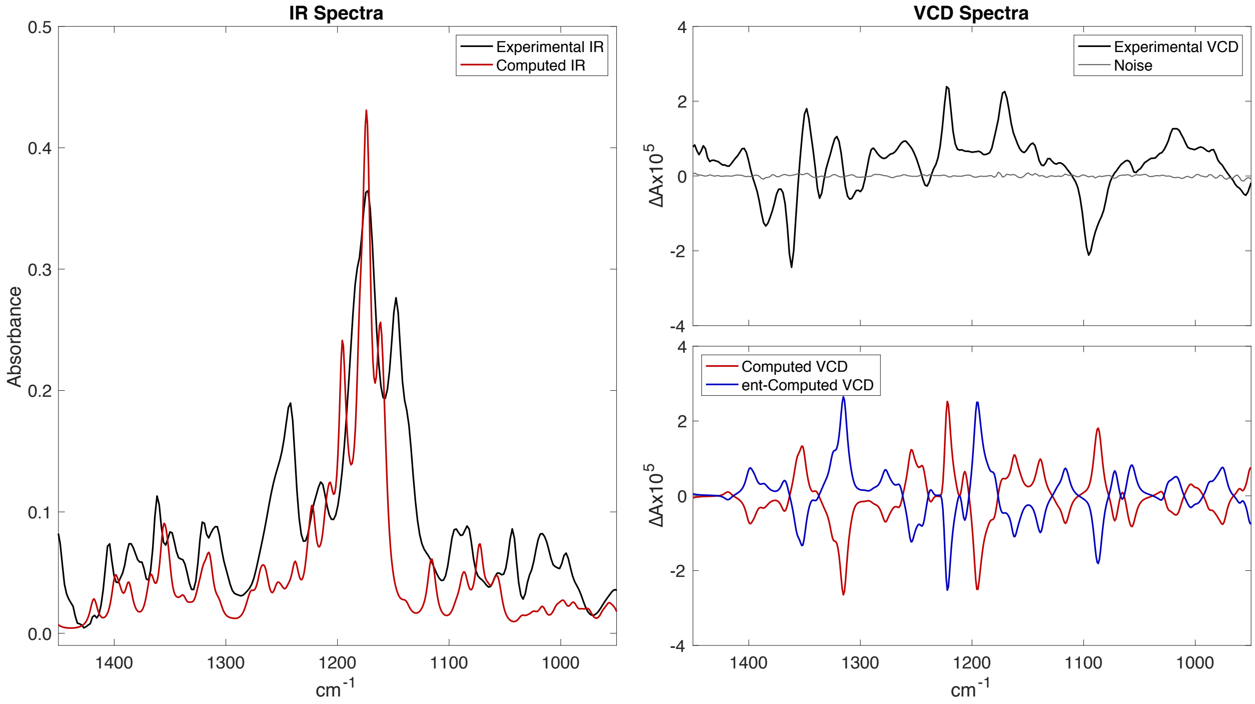

Figure S11. Comparison of experimental VCD and IR spectra for product **11q** to computed spectra for **A_exo-cis**. Experimental data from **11q** do not match computed data of **A_exo-cis**.

*Comparisons between computed and experimental spectra for isomer **11q'**:*

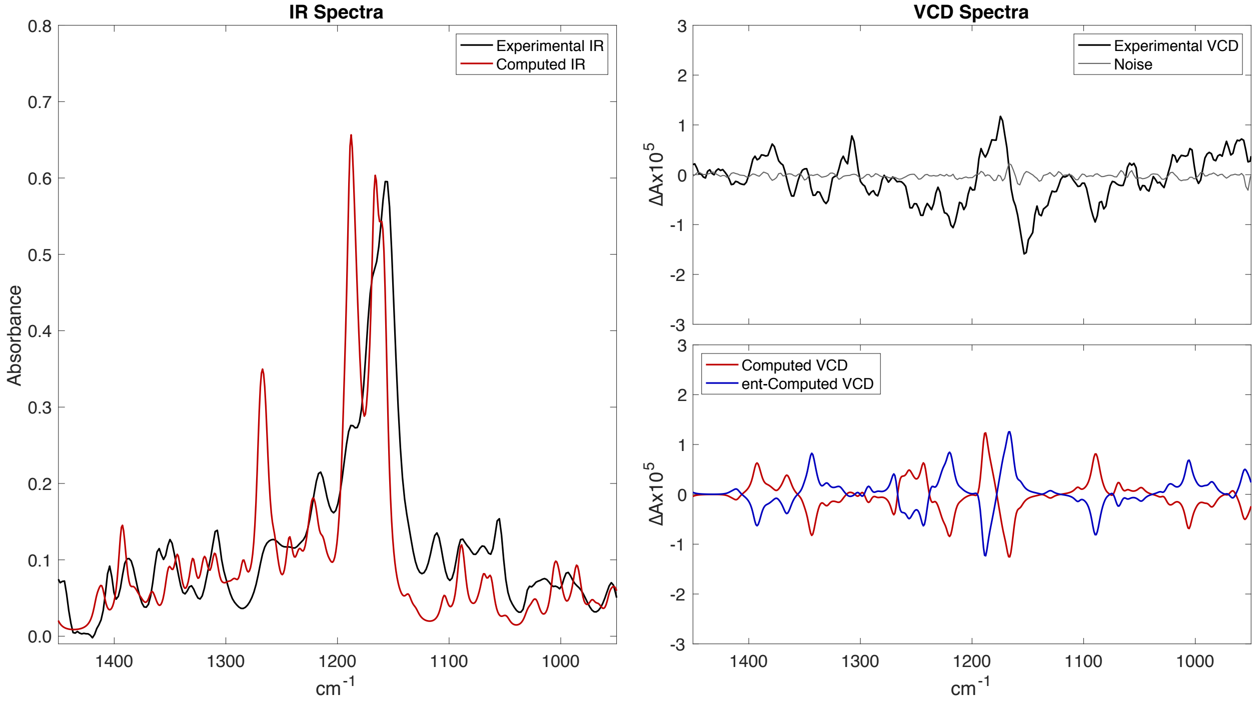

Figure S12. Comparison of experimental VCD and IR spectra for product **11q'** to computed spectra for **A_endo-trans**. The VCD spectrum of **Z** was baseline-corrected with a shift of +7 cm^{-1} along y-axis. Experimental data from **11q'** do not match computed data of **A_endo-trans**.

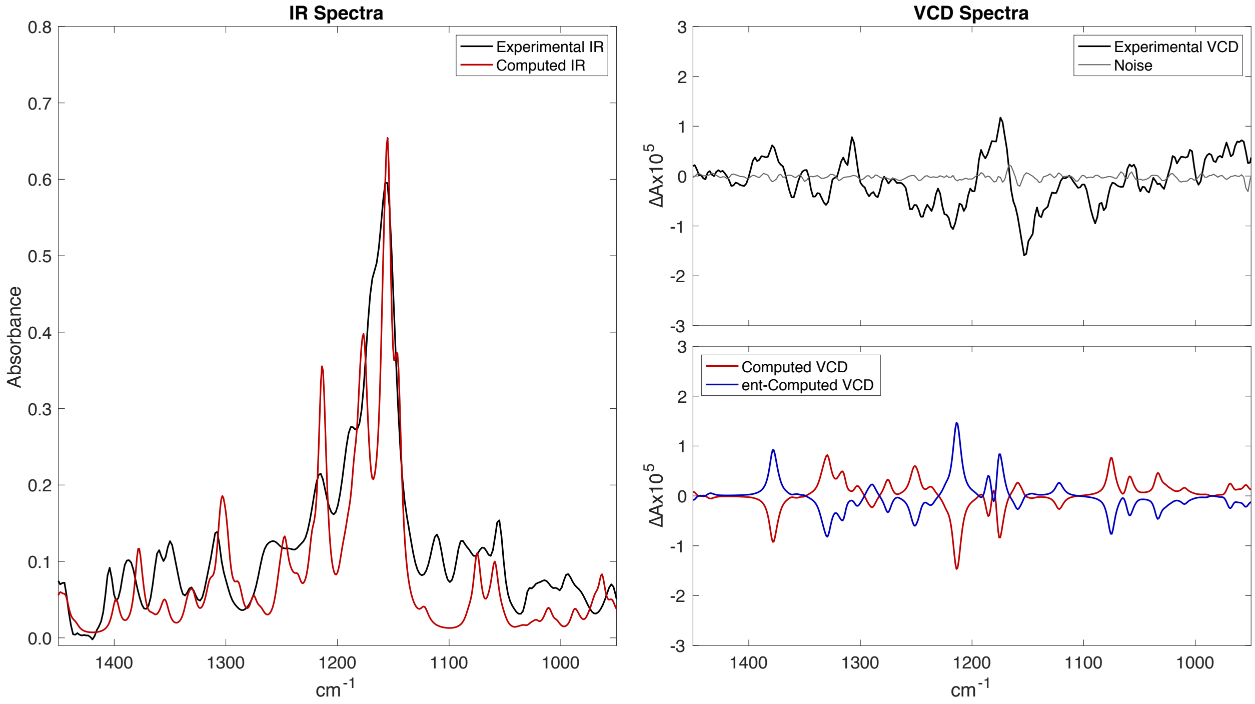

Figure S13. Comparison of experimental VCD and IR spectra for product **11q'** to computed spectra for **A_exo-trans**. The VCD spectrum of **11q'** was baseline-corrected with a shift of +7 cm^{-1} along y-axis. A shift of -15 cm^{-1} along x-axis applied to computed spectra in fitting. Experimental data from **11q'** do not match computed data of **A_exo-trans**.

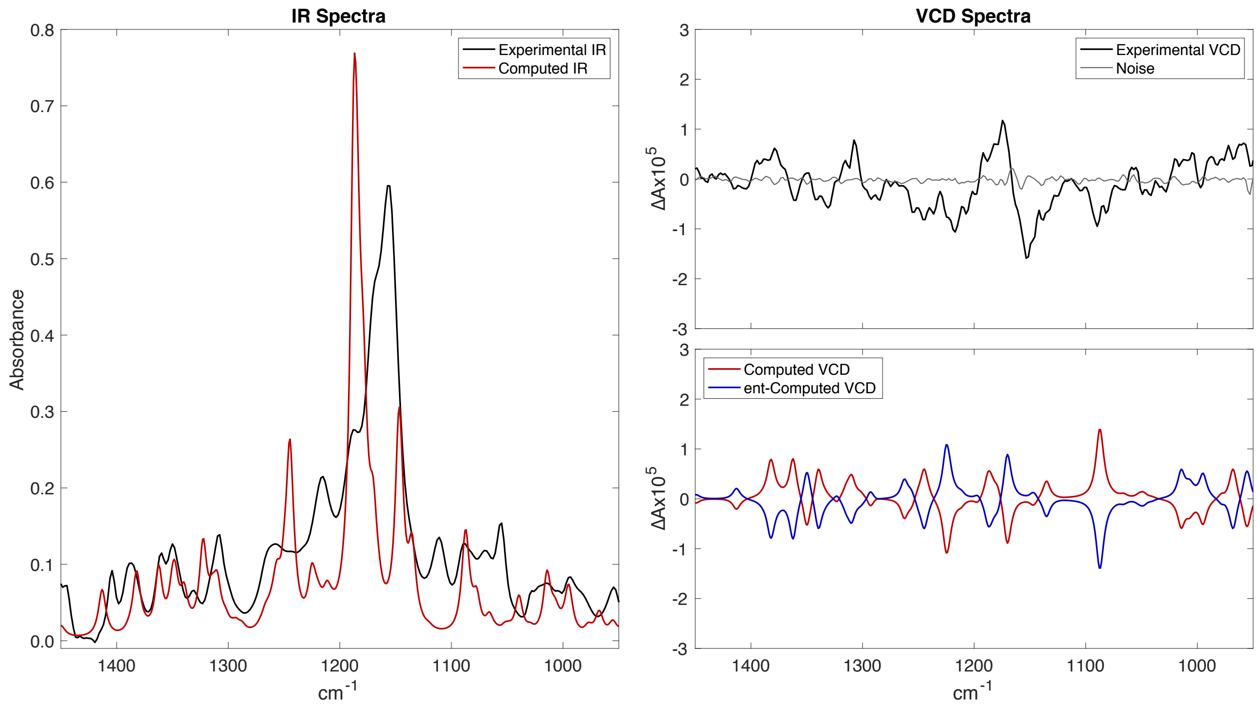

Figure S14. Comparison of experimental VCD and IR spectra for product **11q’** to computed spectra for **A_endo-cis**. The VCD spectrum of **11q’** was baseline-corrected with a shift of +7 cm^{-1} along y-axis. Experimental data from **11q’** do not match computed data of **A_endo-cis**.

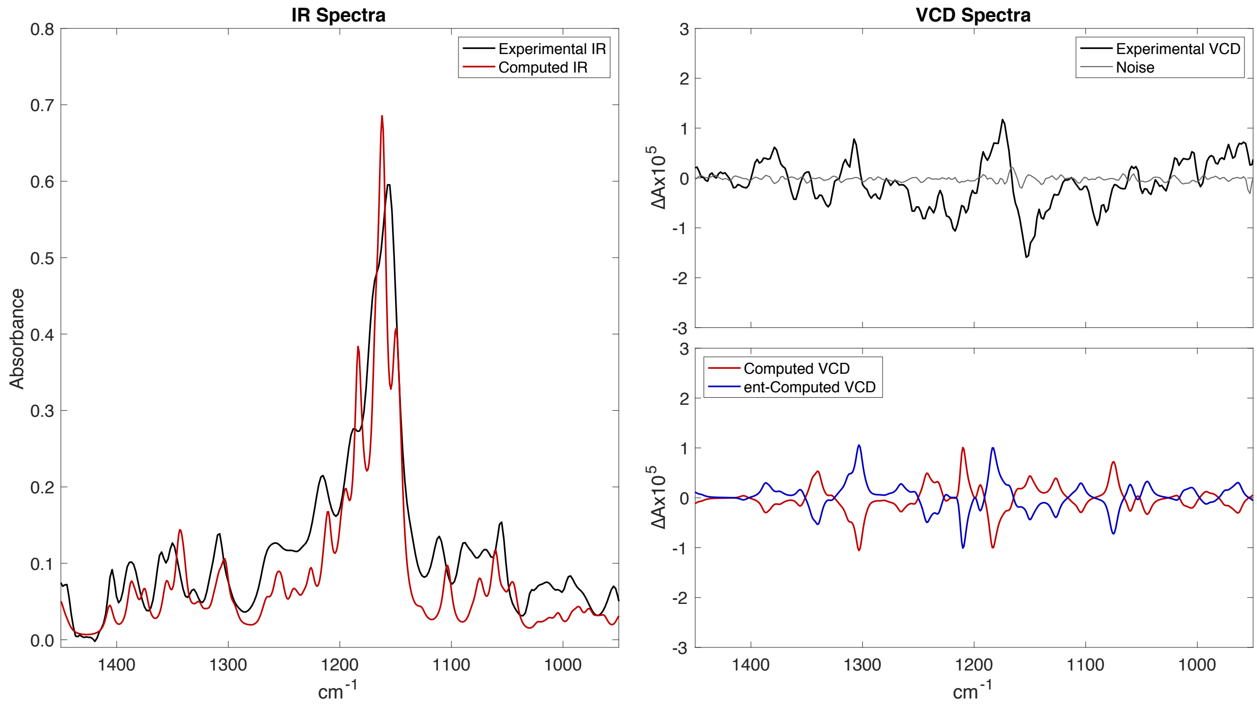

Figure S15. Comparison of experimental VCD and IR spectra for product **11q'** to computed spectra for **A_exo-cis**. The VCD spectrum of **11q'** was baseline-corrected with a shift of +7 cm^{-1} along y-axis. A shift of +7 cm^{-1} along x-axis applied to computed spectra in fitting. Experimental IR spectrum in good agreement with computed spectrum. Experimental VCD spectrum for **11q'** is in good agreement with computed spectrum for ***ent*-A_exo-cis**.

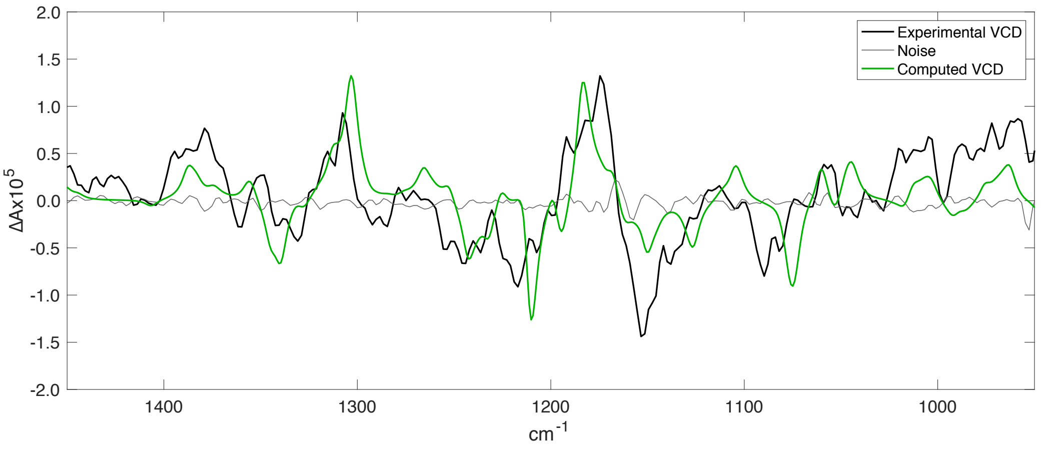

Figure S16. Overlayed experimental and calculated VCD spectra for **11p'** – assigned as ***ent*-A_exo-cis**.

VCD Analysis for diastereomers 11k, 11k', and 11k''.

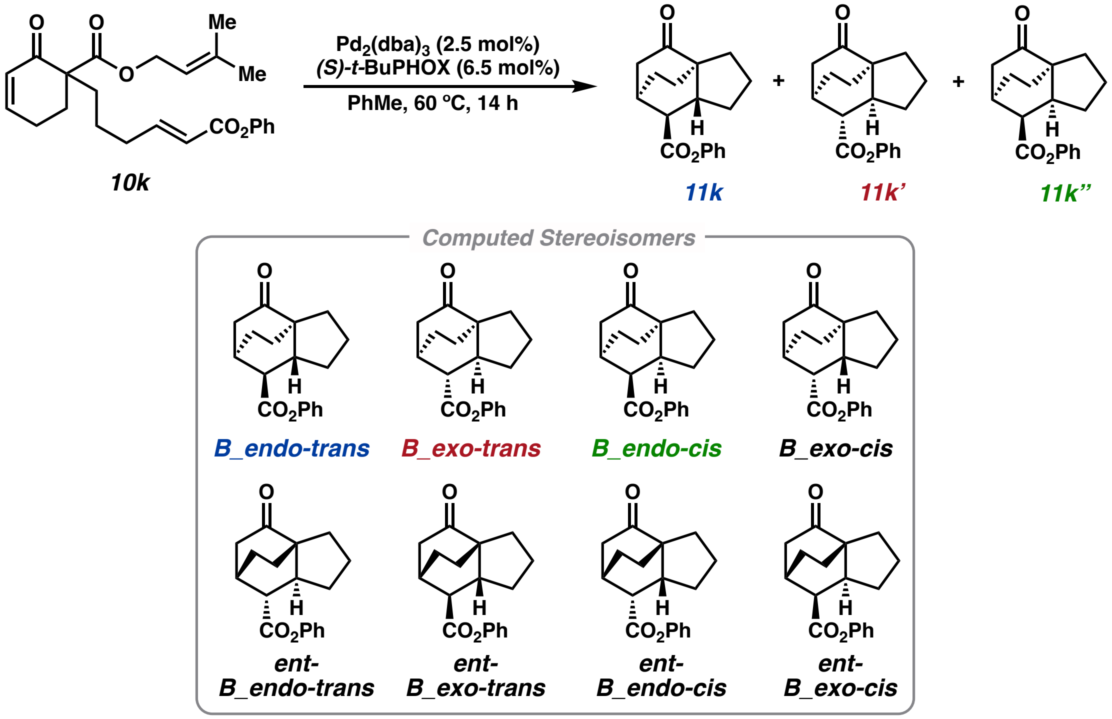

Figure S17. Three diastereomers **11k**, **11k'**, and **11k''** to be compared to spectra computed from all eight possible stereoisomers.

*Comparisons between computed and experimental spectra for isomer **11k**:*

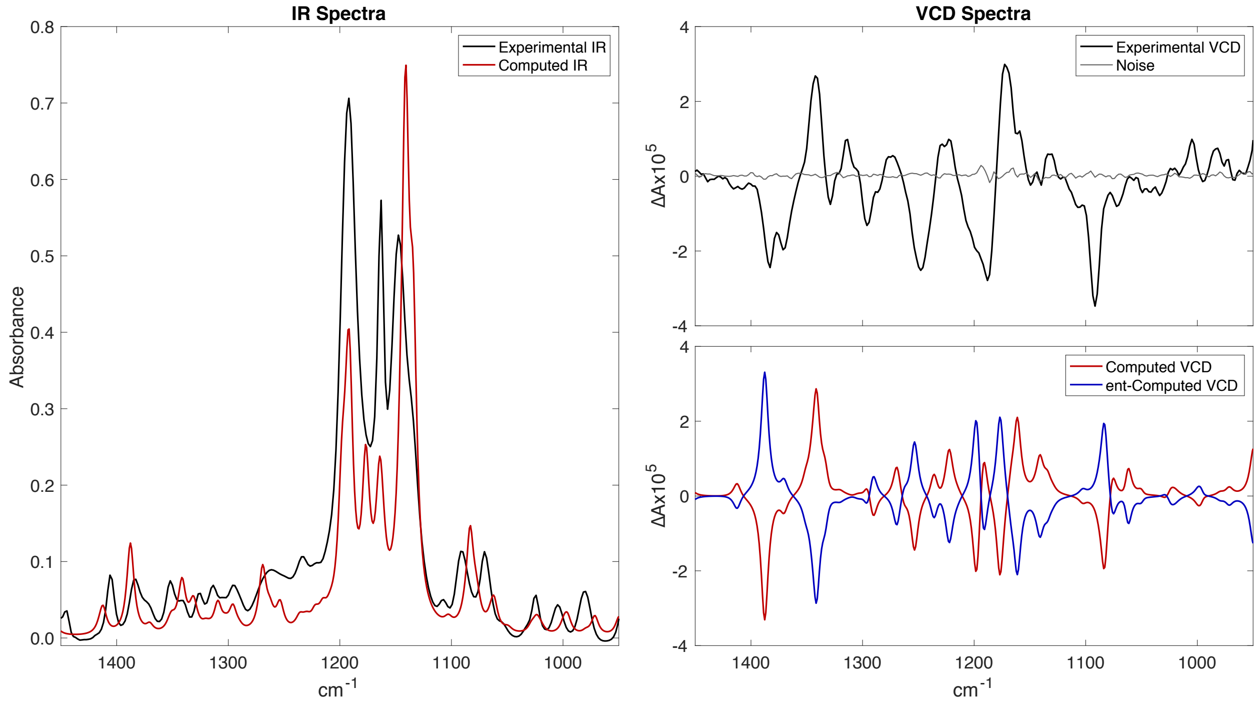

Figure S18. Experimental VCD and IR spectra for product **11k** compared to computed spectra for **B_endo-trans**. Experimental IR spectrum in good agreement with computed spectrum. Experimental VCD spectrum for **11k** is in excellent agreement with computed spectrum for **B_endo-trans**.

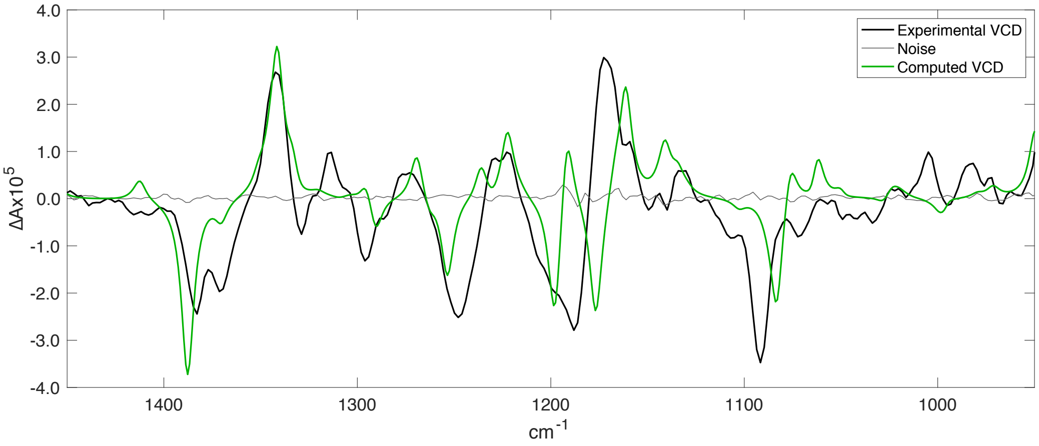

Figure S19. Overlayed experimental and calculated VCD spectra for **11k** – assigned as **B_endo-trans**.

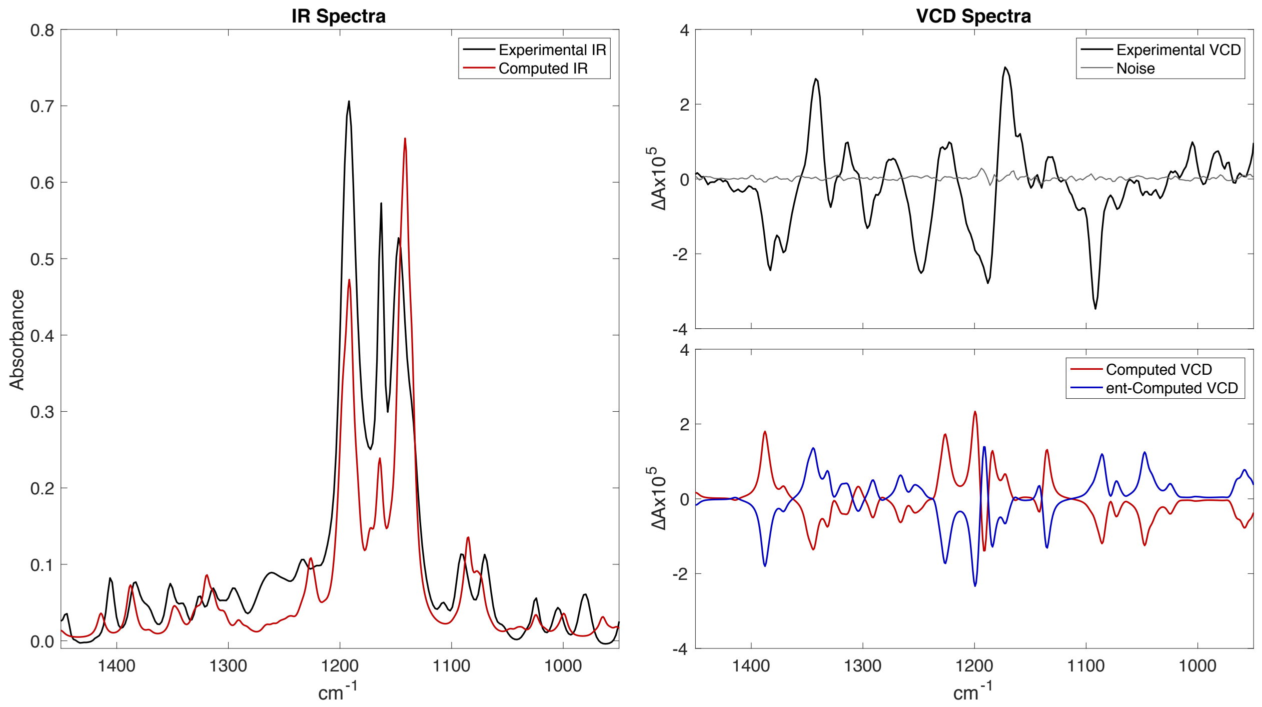

Figure S20. Experimental VCD and IR spectra for product **11k** compared to computed spectra for **B_exo-trans**. Experimental IR spectrum in good agreement with computed spectrum. However, VCD spectrum contain key sign mismatches in regions around 1400 and 1100 cm^{-1}. Hence, **11k** is <u>not</u> assigned as **B_exo-trans**.

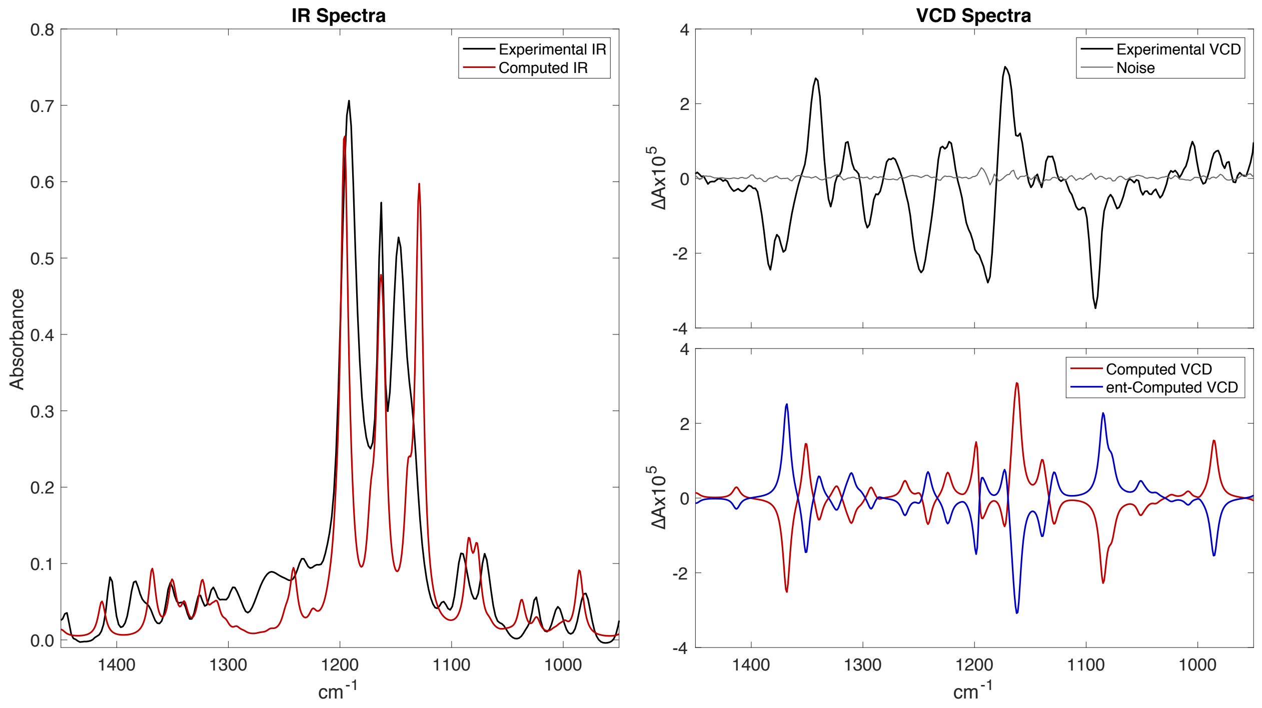

Figure S21. Experimental VCD and IR spectra for product **11k** compared to computed spectra for **B_endo-cis**. Experimental data do not match computed data and **11k** is <u>not</u> assigned as **B_endo-cis**.

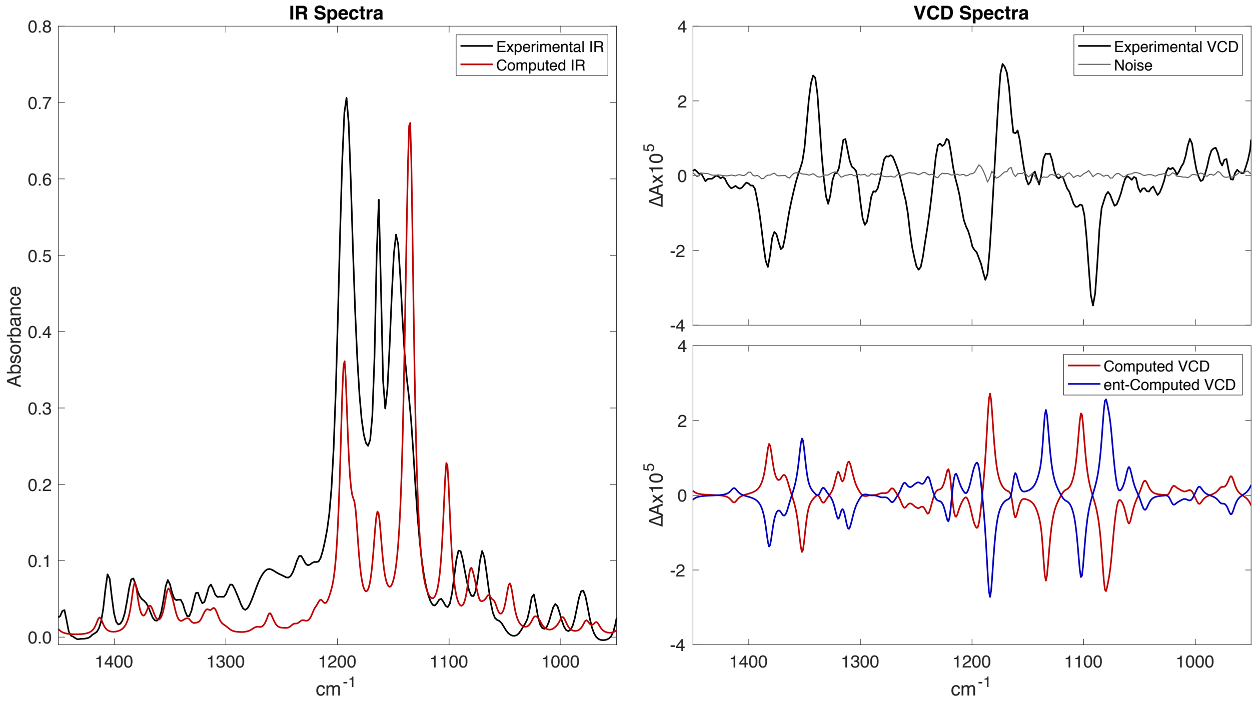

Figure 22. Experimental VCD and IR spectra for product **11k** compared to computed spectra for **B_exo-cis**. Experimental data do not match computed data and **11k** is <u>not</u> assigned as **B_exo-cis**.

*Comparisons between computed and experimental spectra for isomer **11k'**:*

Due to limited sample size (< 3 mg), useful VCD spectra of **11k'** were unable to be obtained. Enantiomeric series was assigned by analogy to the **11a**, **11q** and **11q'** series. The 1000–1500 cm^{-1} region of the IR spectra are still analyzed to support relative stereochemical assignments made by 2D NMR.

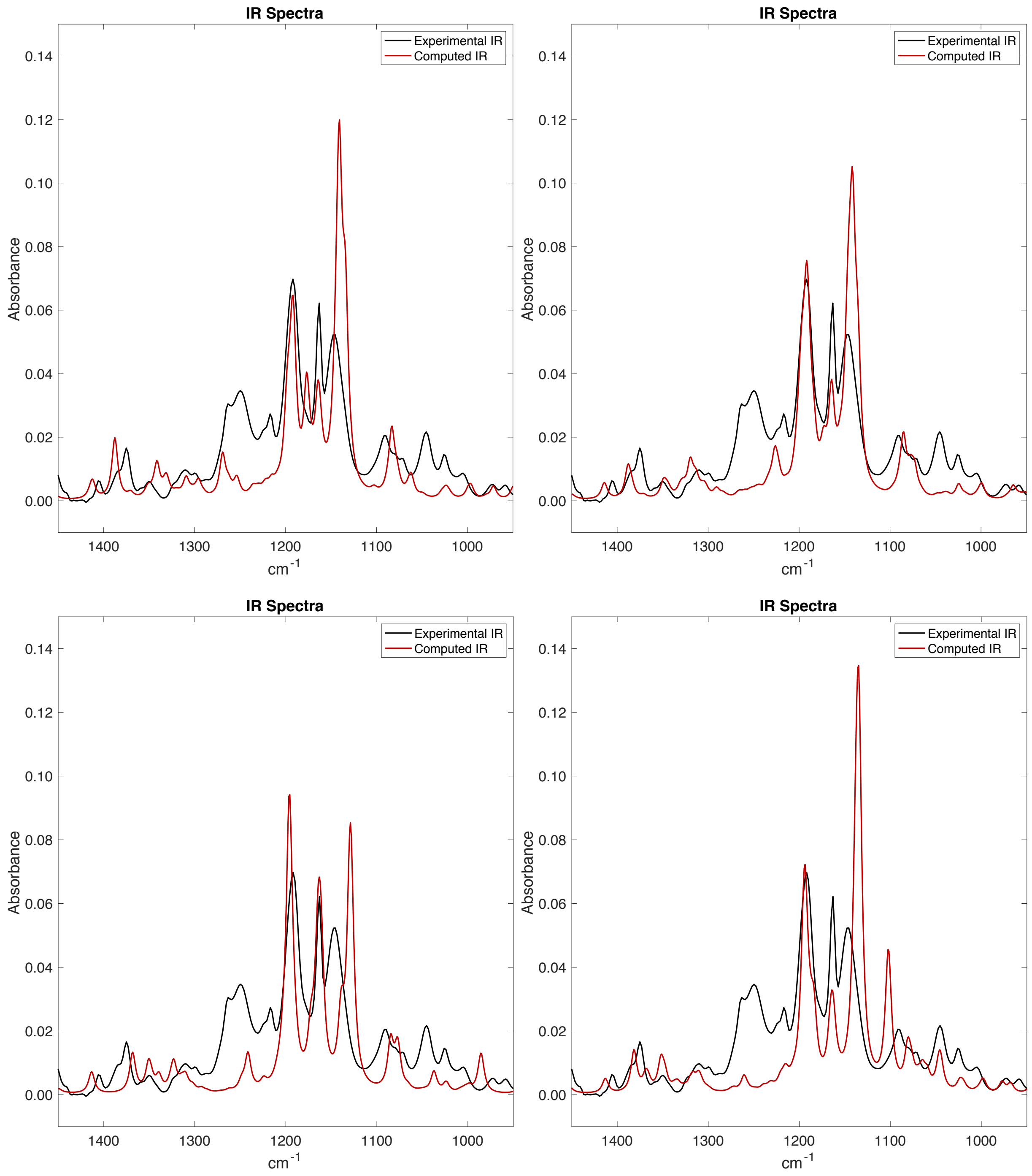

Figure S23. Experimental IR spectrum for product **11k'** compared to computed spectra for **B_endo-trans** (top left), **B_exo-trans** (top right), **B_endo-cis** (bottom left), **B_exo-cis** (bottom right). The *trans* relationship is supported, in accord with 2D NMR data. In contrast to *endo-cis* and *exo-cis*, the computed IR spectra for both *endo-trans* and *exo-trans* are similar and do not offer key features for distinguishing the two. Given the *trans* stereochemistry, with **11k** known as **B_endo-trans**, **11k'** is assigned as **B_exo-trans** with absolute stereochemistry assigned based on analogy to **11q'**.

*Comparisons between computed and experimental spectra for isomer **11k''**:*

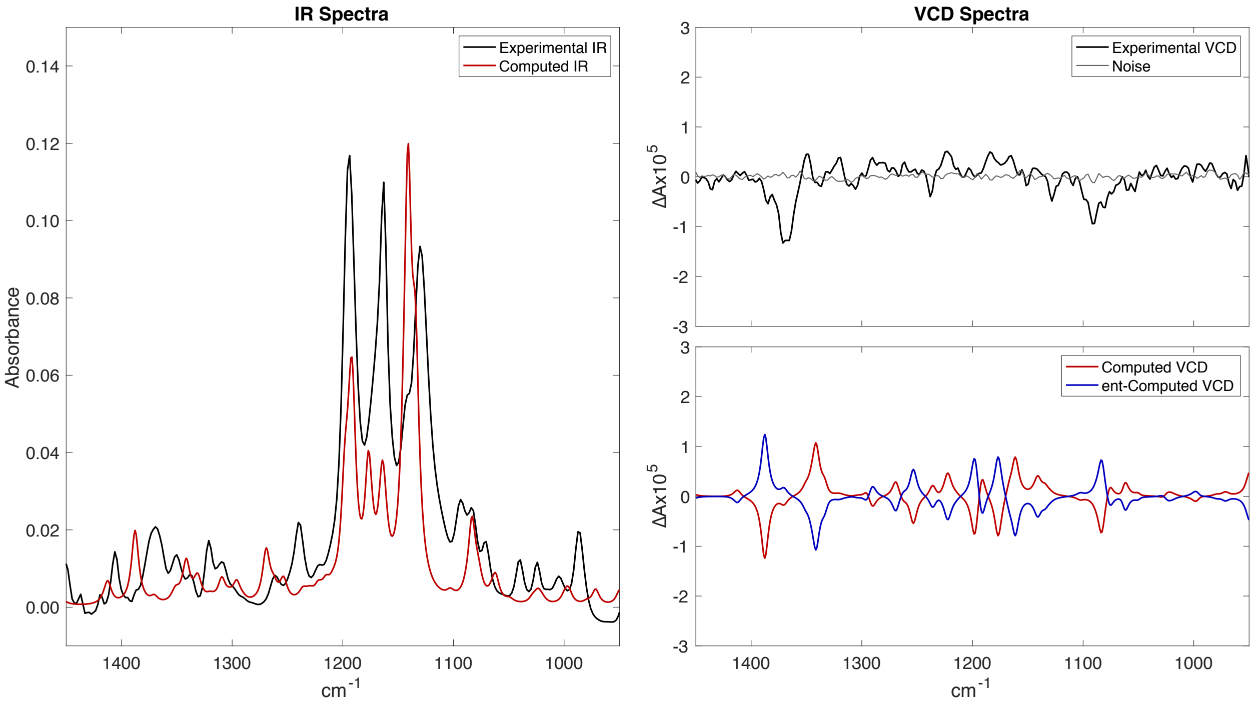

Figure S24. Experimental VCD and IR spectra for product **11k''** compared to computed spectra for **B_endo-trans**. Experimental data do not match computed data and **11k''** is not assigned as **B_endo-trans**.

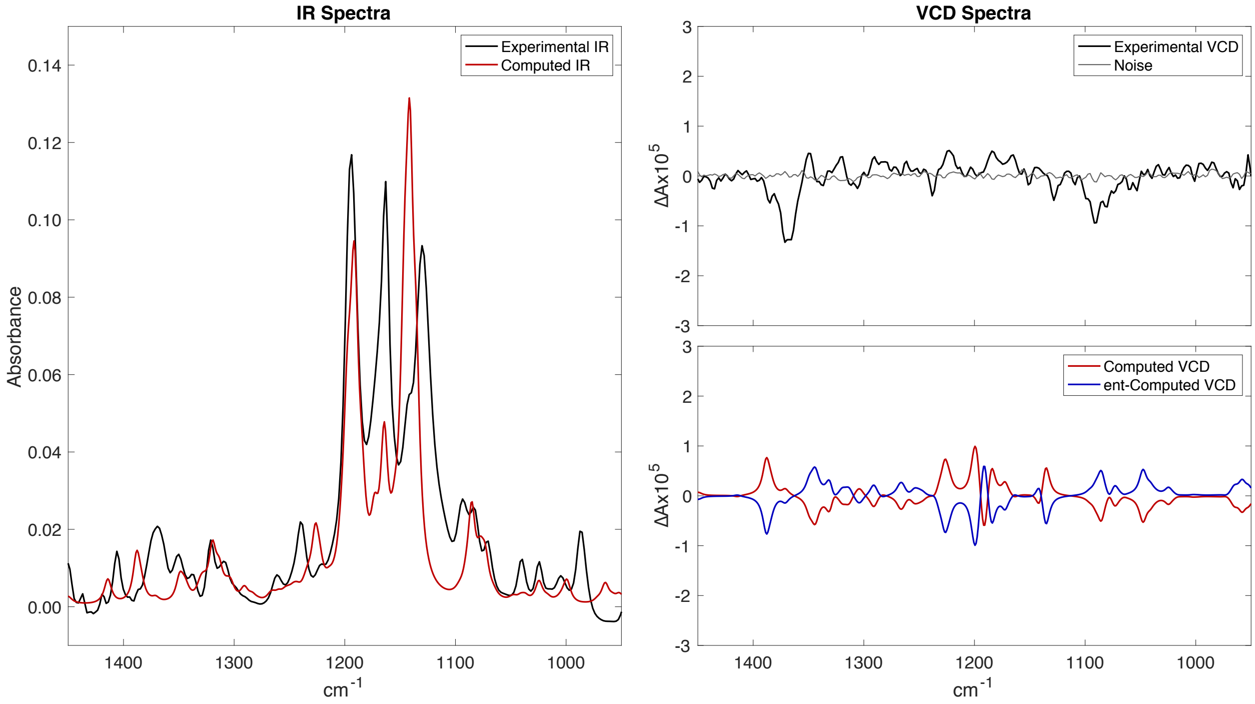

Figure S25. Experimental VCD and IR spectra for product **11k’’** compared to computed spectra for **B_exo-trans**. Experimental data do not match computed data and **11k’’** is <u>not</u> assigned as **B_exo-trans**.

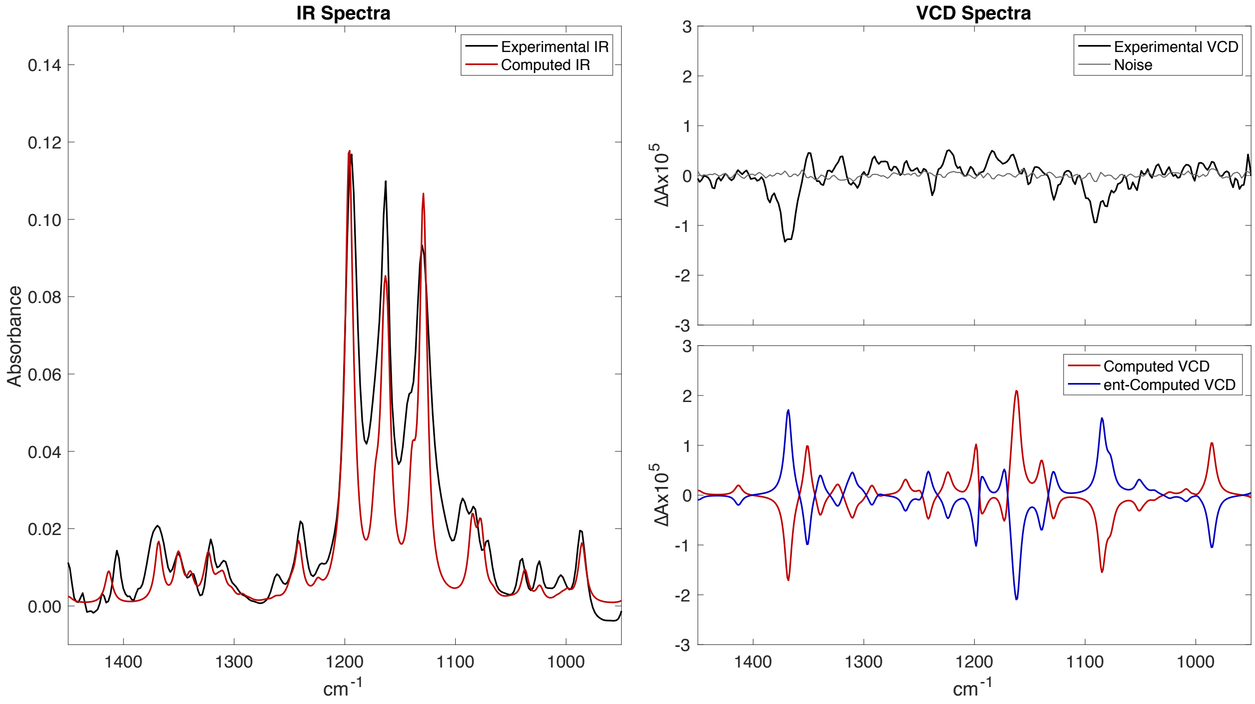

Figure S26. Experimental VCD and IR spectra for product **11k’’** compared to computed spectra for **B_endo-cis**. Experimental IR spectrum is in excellent agreement with the computed IR spectrum of **B_endo-cis**.

Assignment of absolute stereochemistry is based the on the sign of the three most intense peaks in VCD spectrum, 1368, 1350, and 1085 cm^{-1}. These match **B_endo-cis**, the same enantiomeric series as **11k**.

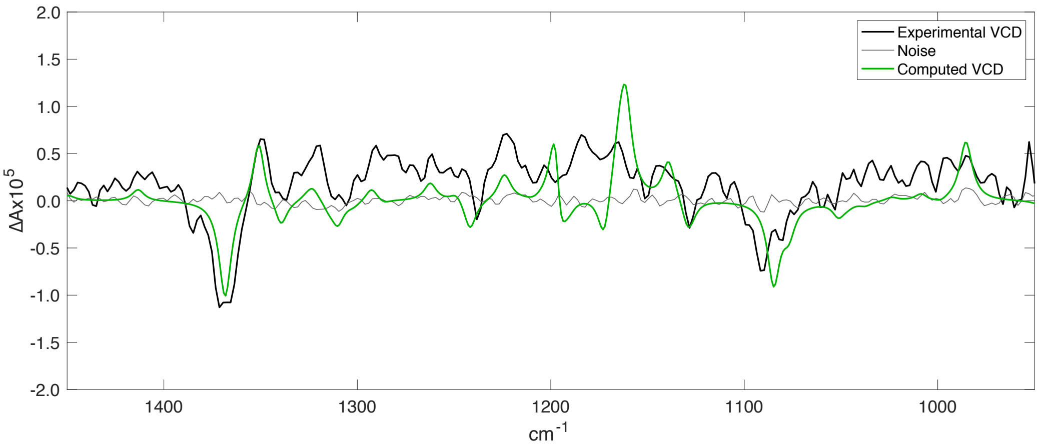

Figure S27. Overlayed experimental and calculated VCD spectra for **11k''** – assigned as **B_endo-cis**.

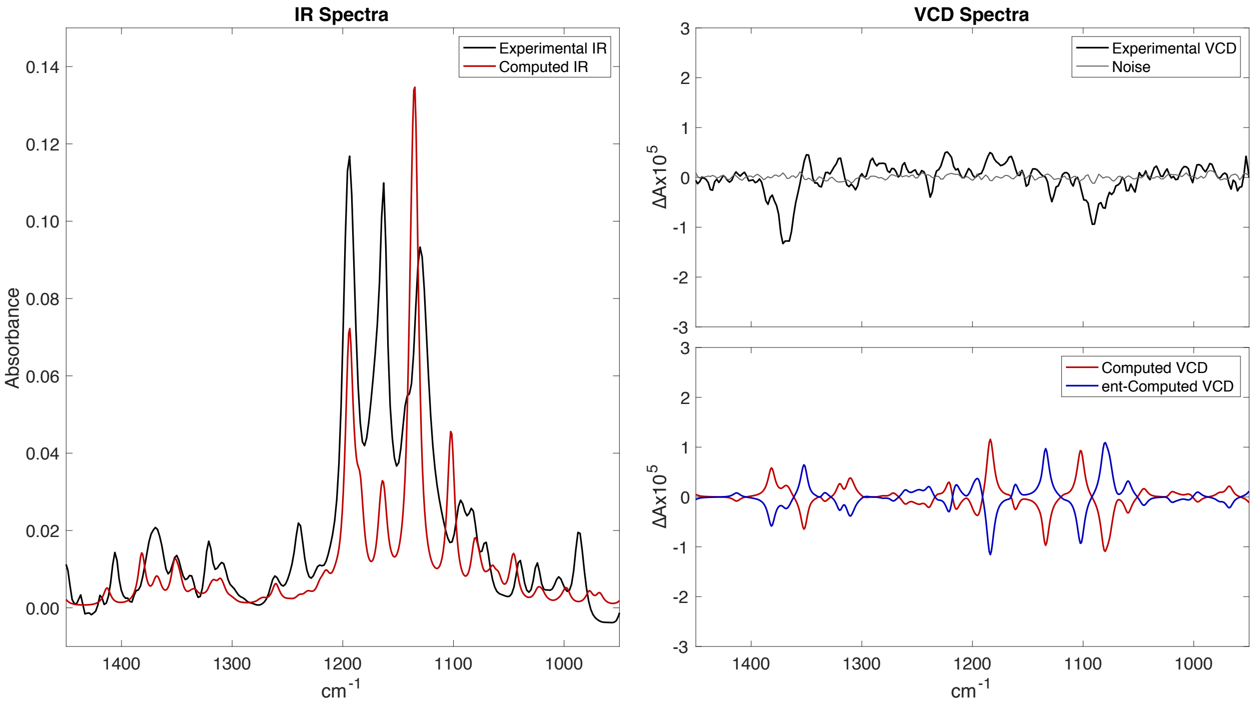

Figure S28. Experimental VCD and IR spectra for product **11j''** compared to computed spectra for **B_exo-cis**. Experimental data do not match computed data and **11j''** is <u>not</u> assigned as **B_exo-cis**.

VCD Analysis for diastereomers 11t and 11t'

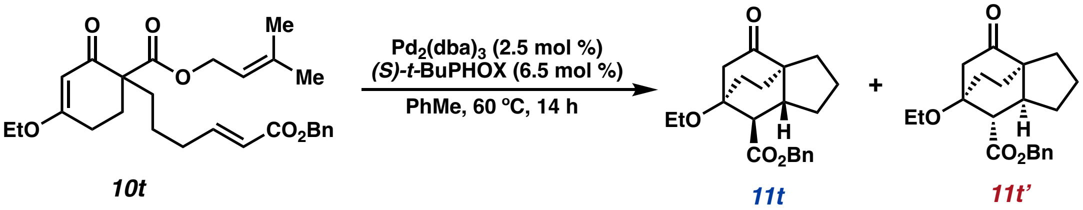

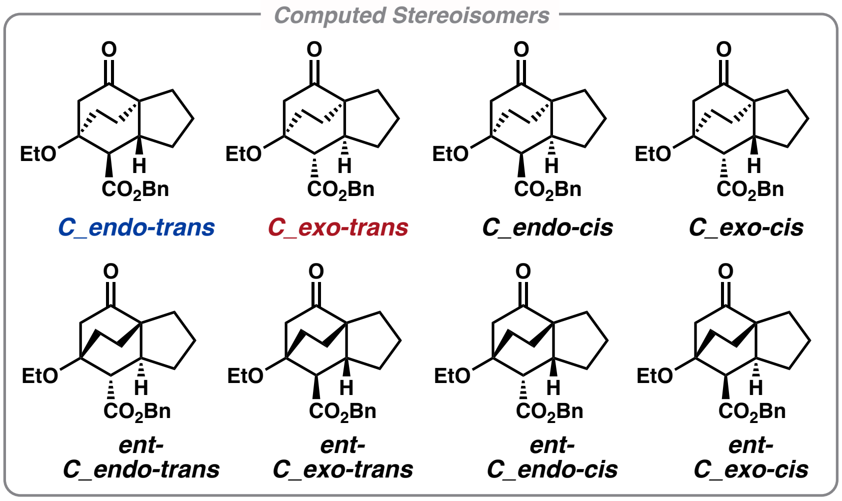

Figure S29. Two diastereomers **11t** and **11t'** to be compared to spectra computed from all eight possible stereoisomers.

Comparisons between computed and experimental spectra for isomer ***11t****:*

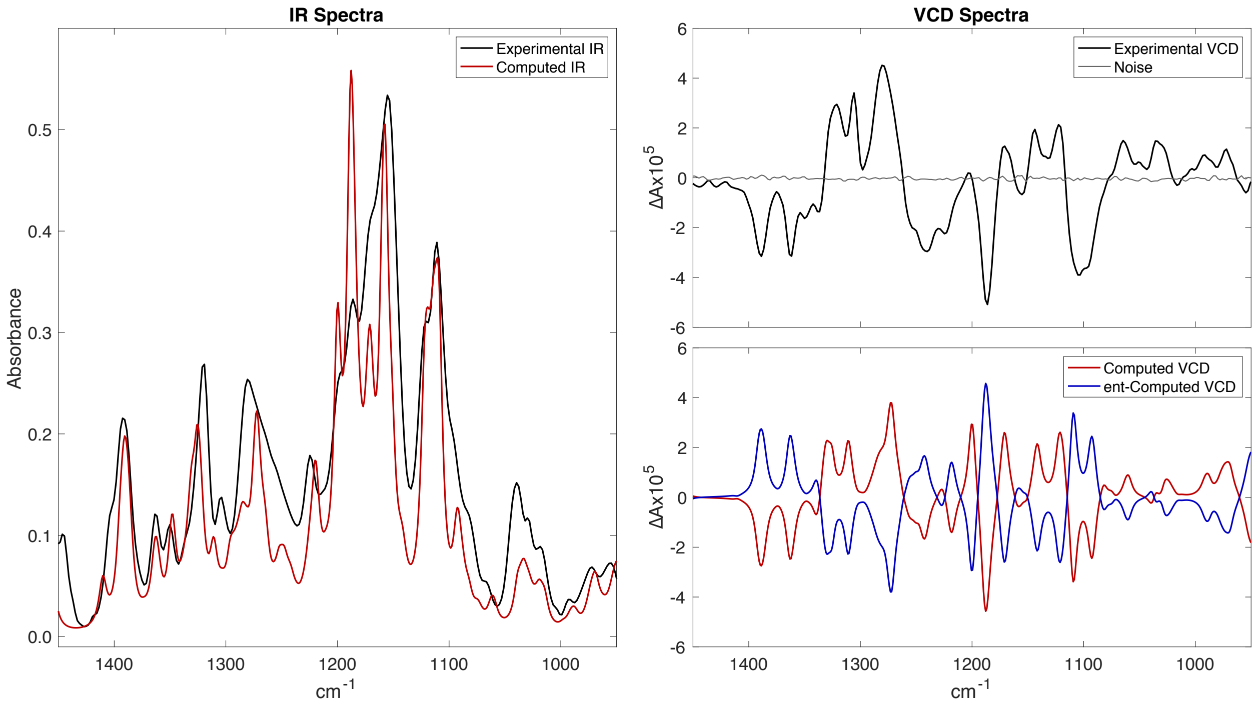

Figure S30. Experimental VCD and IR spectra for product **11t** compared to computed spectra for **C_endo-trans**. Experimental IR spectrum in excellent agreement with computed spectrum. Experimental VCD spectrum for **11t** is in excellent agreement with computed spectrum for **C_endo-trans**.

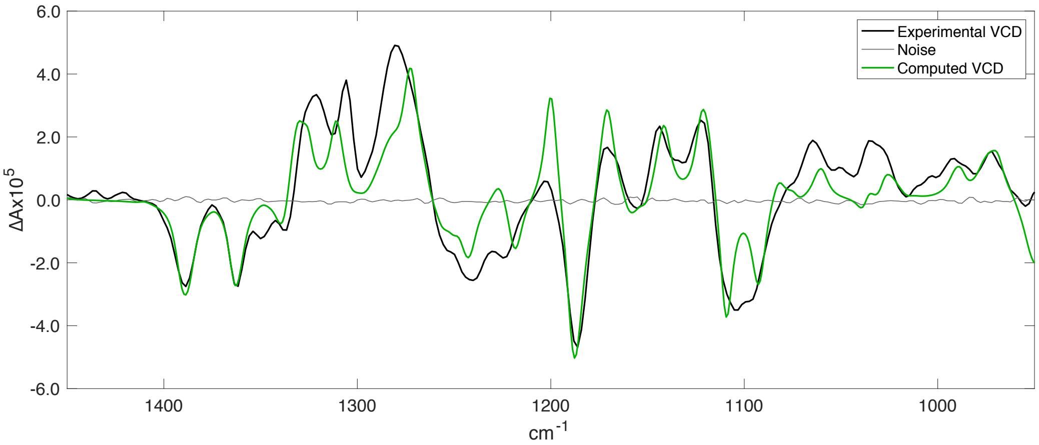

Figure S31. Overlayed experimental and calculated VCD spectra for **11t** – assigned as **C_endo-trans**.

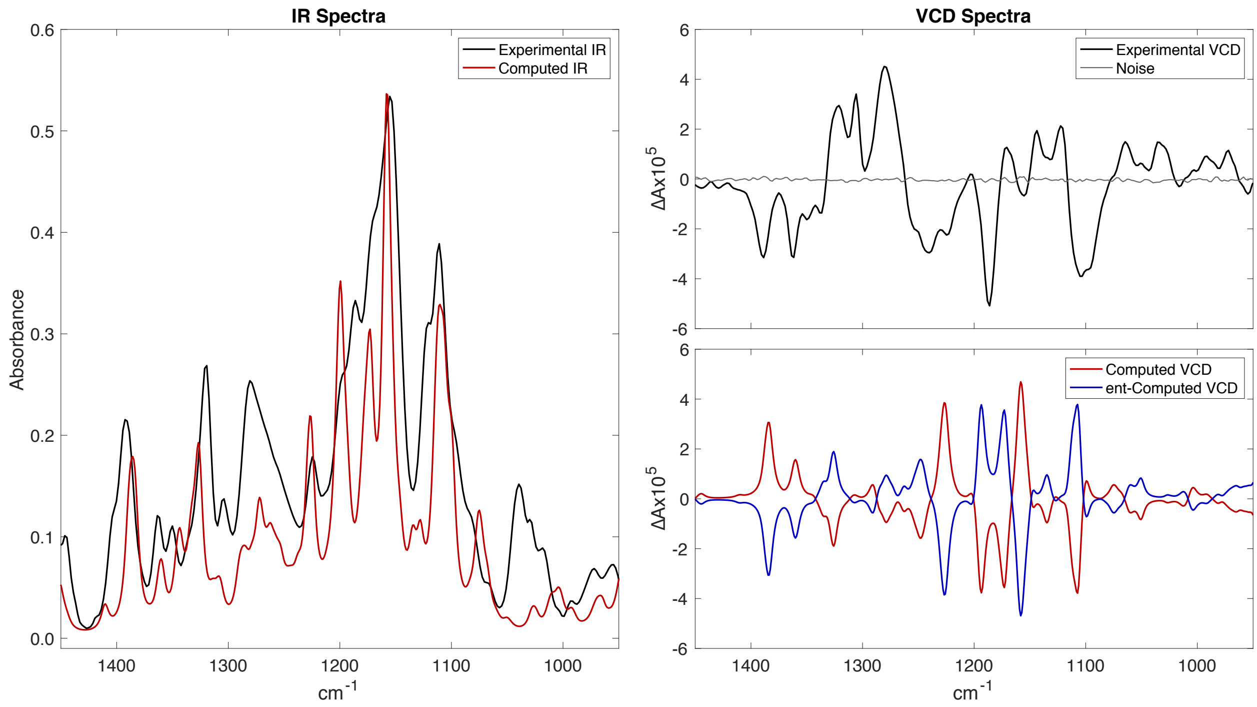

Figure S32. Comparison of experimental VCD and IR spectra for product **11t** to computed spectra for **C_exo-trans**. A shift of -5 cm^{-1} along x-axis applied to computed spectra in fitting. Experimental data from **11t** do <u>not</u> match computed data of **C_exo-trans**.

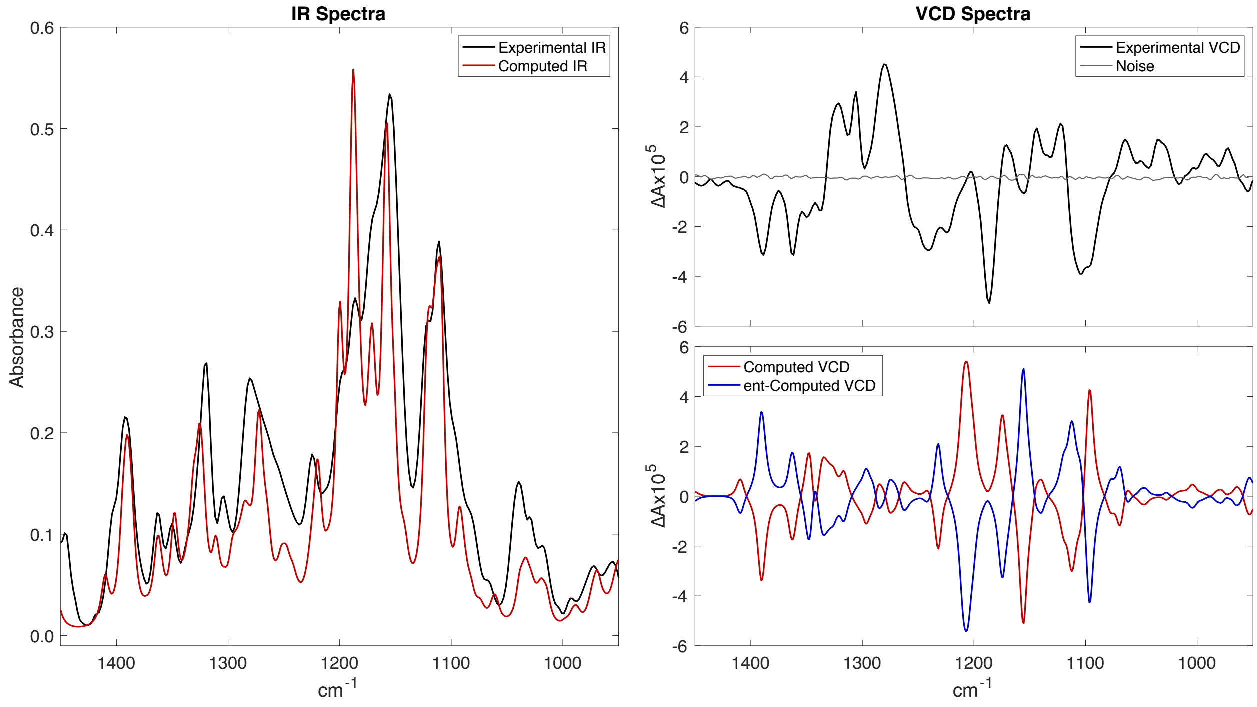

Figure S33. Comparison of experimental VCD and IR spectra for product **11t** to computed spectra for **C_endo-cis**. Experimental data from **11t** do <u>not</u> match computed data of **C_endo-cis**.

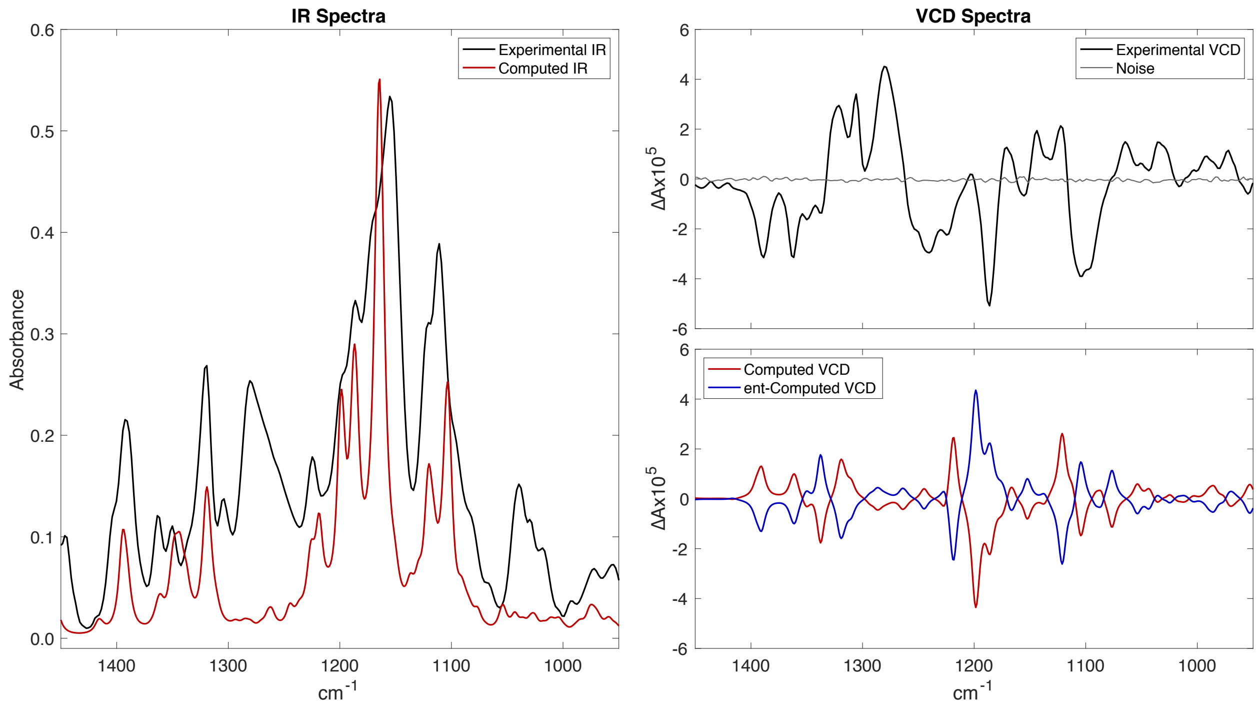

Figure S34. Comparison of experimental VCD and IR spectra for product **11t** to computed spectra for **C_exo-cis**. Experimental data from **11t** do <u>not</u> match computed data of **C_exo-cis**.

*Comparisons between computed and experimental spectra for isomer **11t'**:*

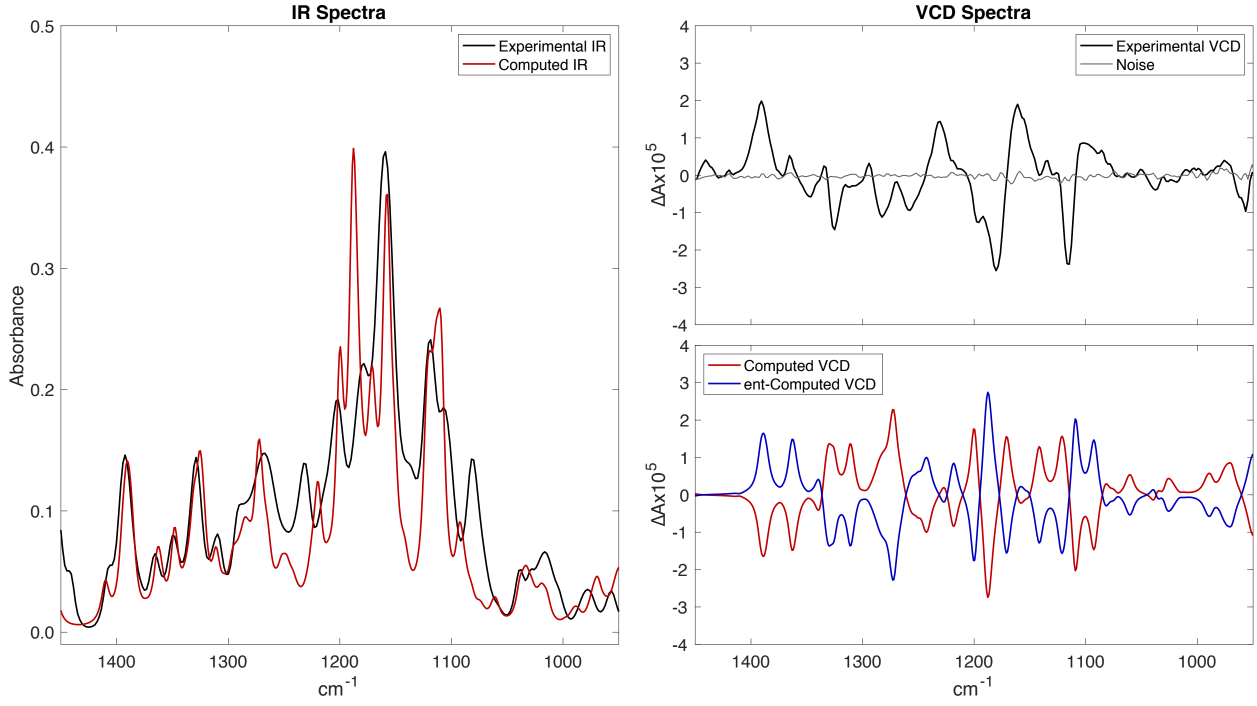

Figure S35. Comparison of experimental VCD and IR spectra for product **11t'** to computed spectra for **C_endo-trans**. Experimental data from **11t'** do <u>not</u> match computed data of **C_endo-trans**.

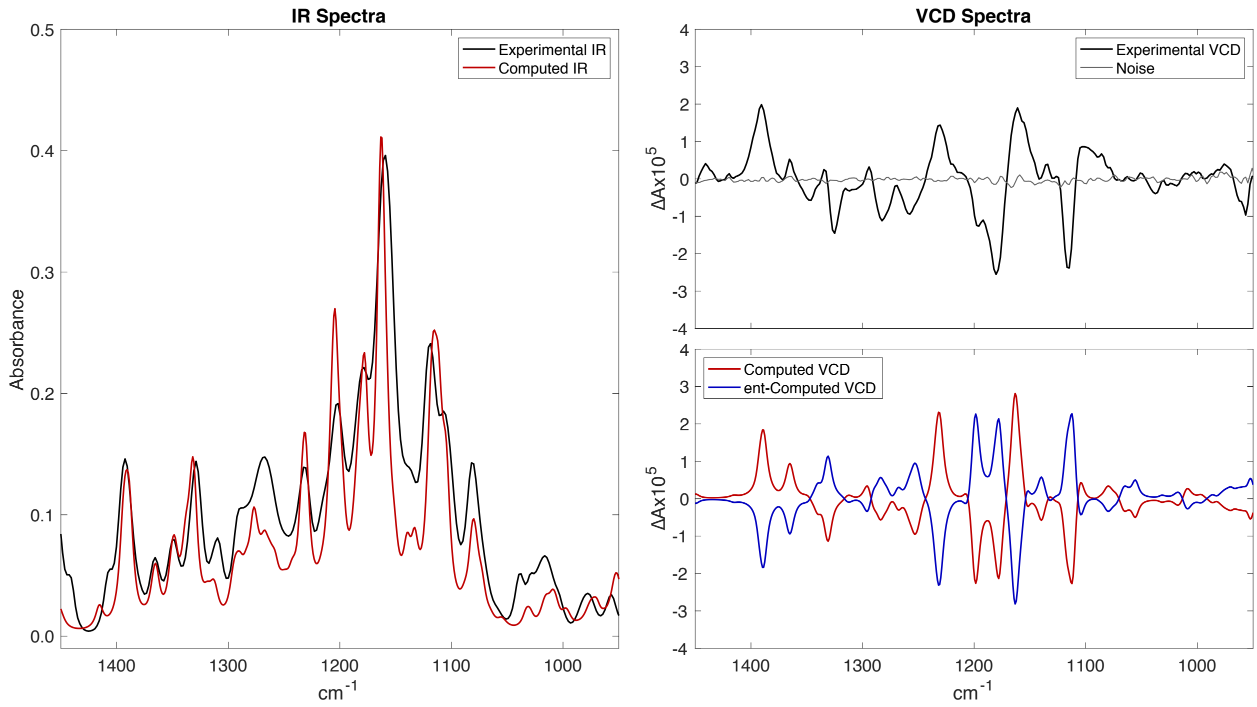

Figure S36. Experimental VCD and IR spectra for product **11t'** compared to computed spectra for **C_exo-trans**. Experimental IR spectrum in excellent agreement with computed spectrum. Experimental VCD spectrum for **11t'** is in excellent agreement with computed spectrum for **C_exo-trans**.

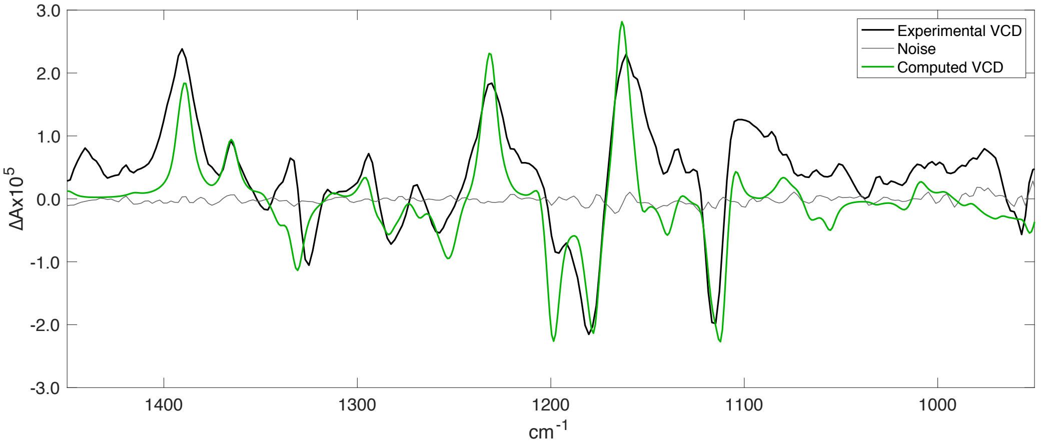

Figure S37. Overlayed experimental and calculated VCD spectra for **11s'** – assigned as **C_exo-trans**.

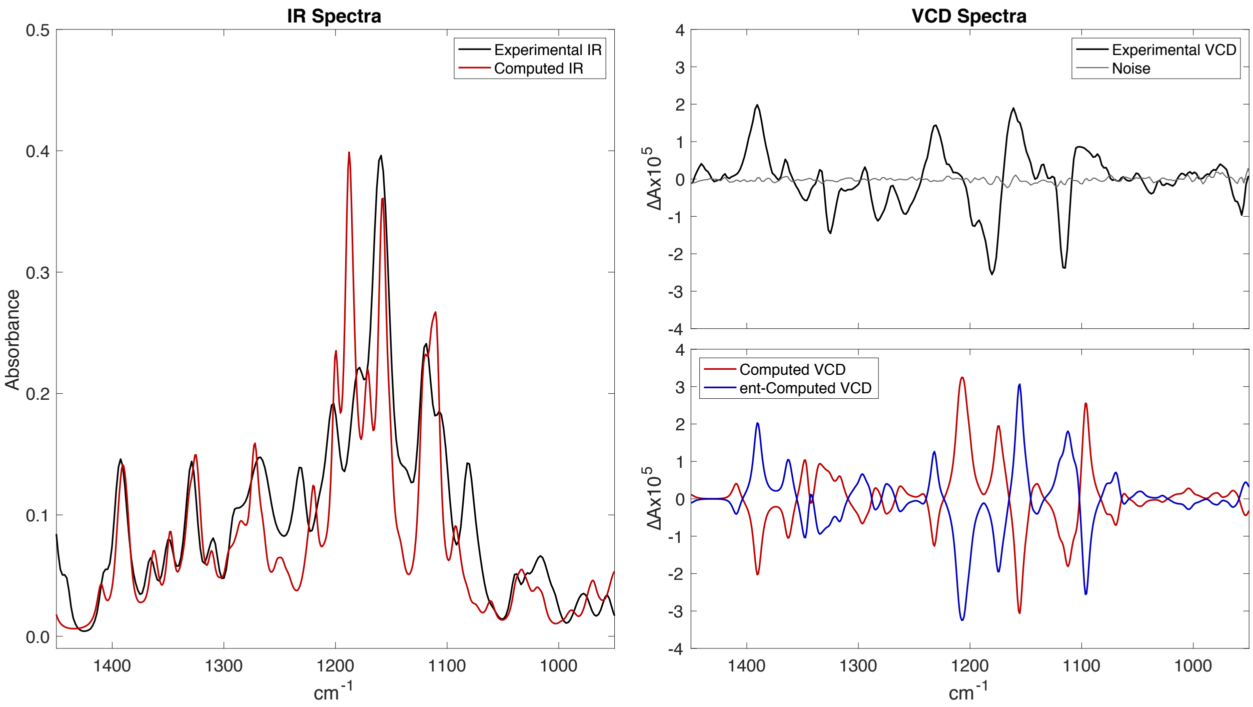

Figure S38. Comparison of experimental VCD and IR spectra for product **11t'** to computed spectra for **C_endo-trans**. Experimental VCD data from **11t'** do <u>not</u> match computed data of **C_endo-trans**.

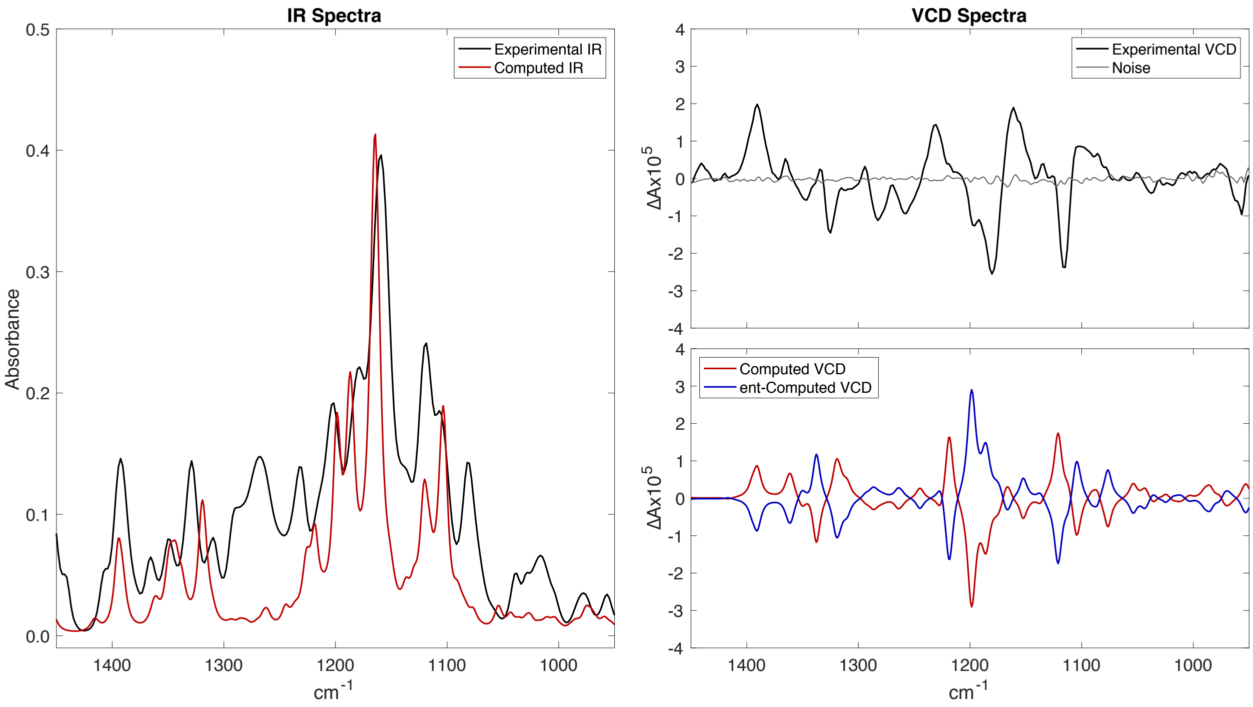

Figure S39. Comparison of experimental VCD and IR spectra for product **11t'** to computed spectra for **C_exo-trans**. Experimental VCD data from **11t'** do <u>not</u> match computed data of **C_exo-trans**.

2D NMR Analysis of Select Compounds

*Compound **11a**:*

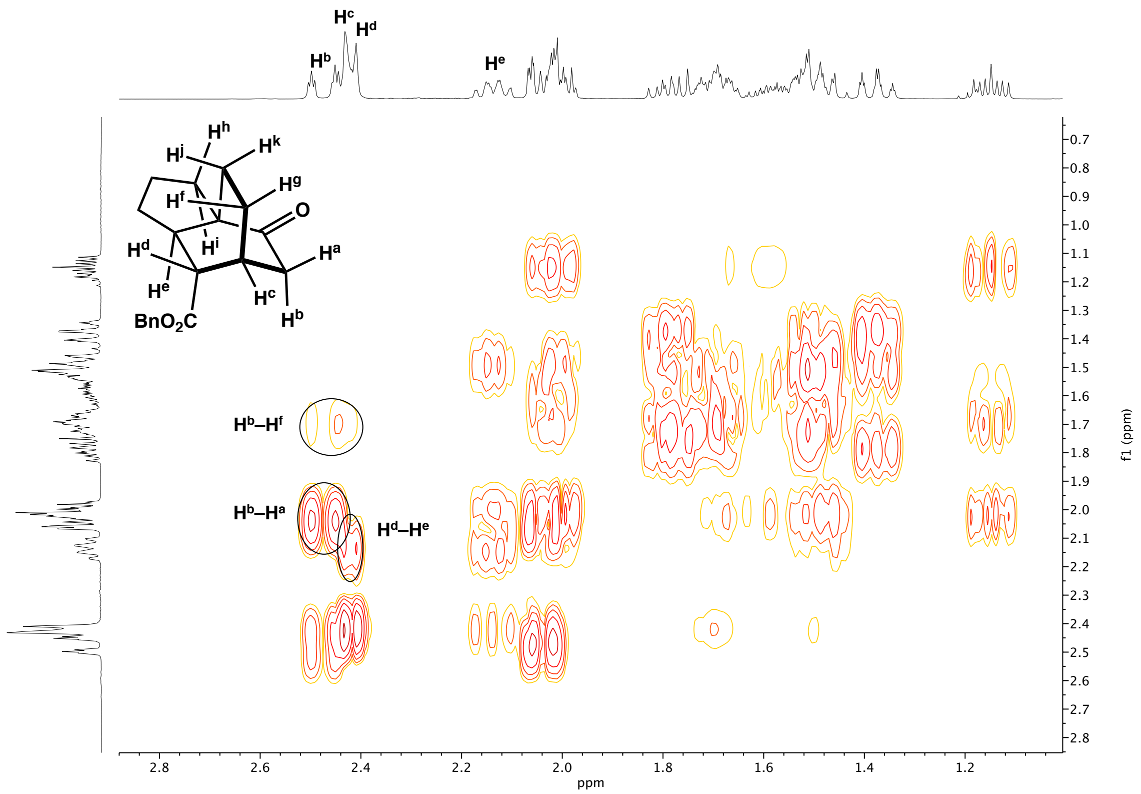

Figure S40. ^{1}H-^{1}H COSY NMR spectrum of **11a** (400 MHz, $CDCl_3$).

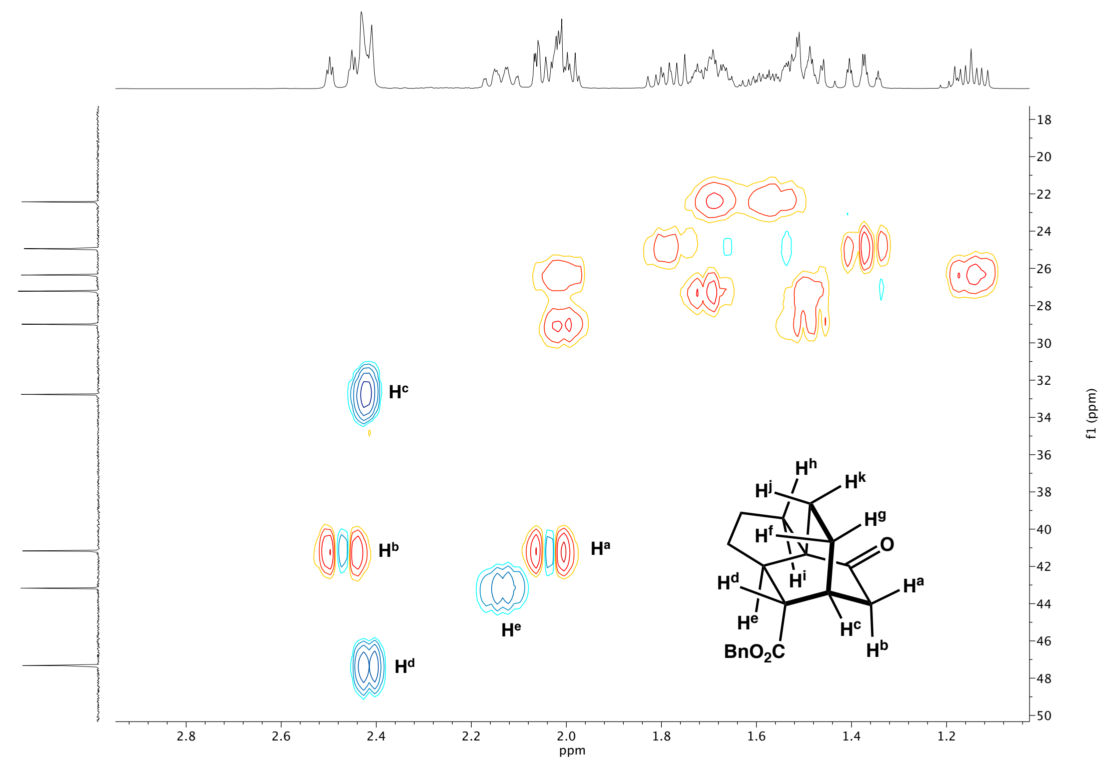

Figure S41. ^{1}H-^{13}C HSQC NMR spectrum of **11a** (400 MHz, $CDCl_3$).

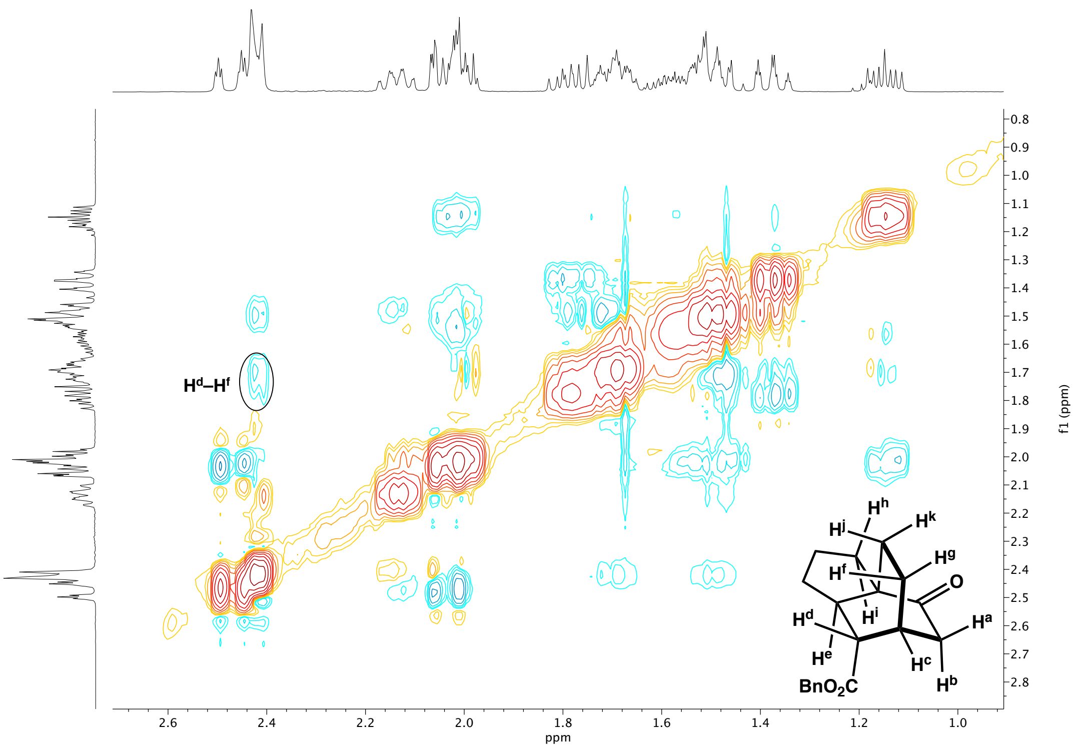

Figure S42. ^{1}H-^{1}H NOESY NMR spectrum of **11a** (400 MHz, $CDCl_3$).

*Compound **11p**:*

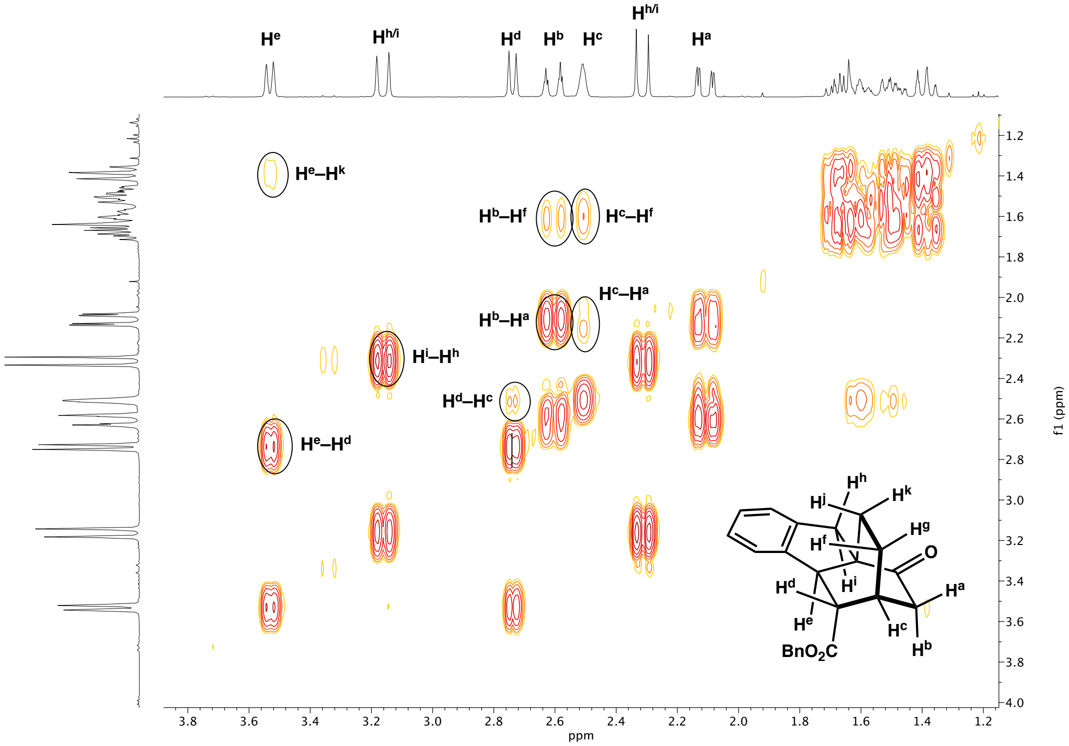

Figure S43. ^{1}H-^{1}H COSY NMR spectrum of **11p** (400 MHz, $CDCl_3$).

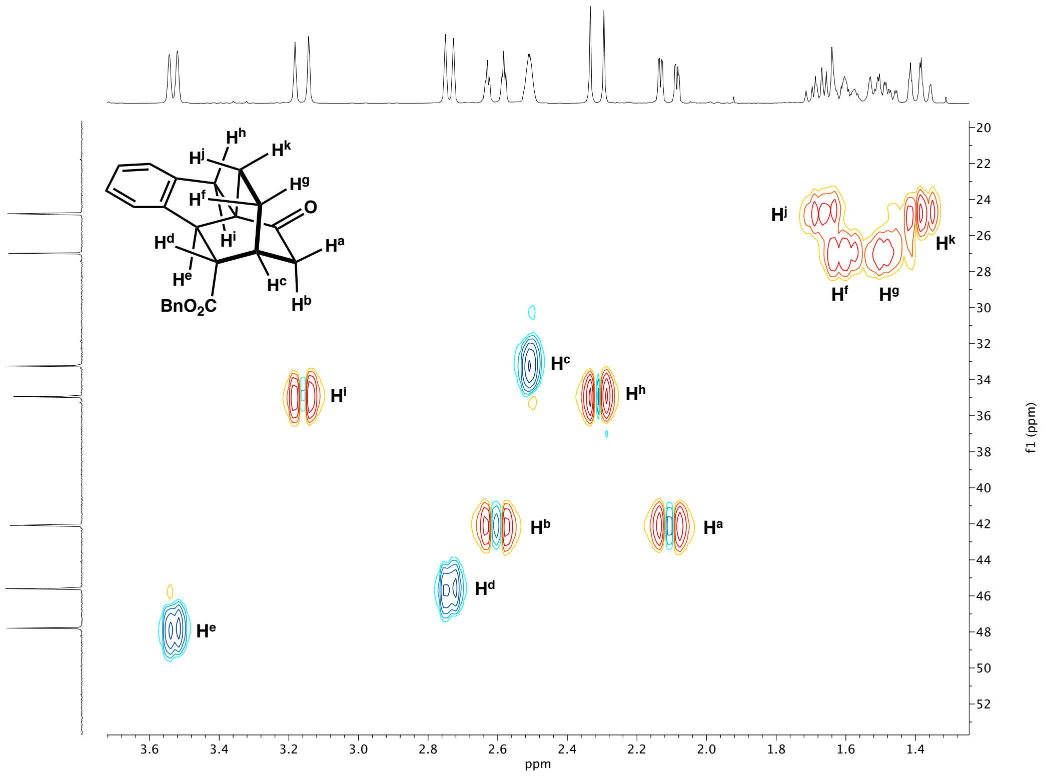

Figure S44. ^{1}H-^{13}C HSQC NMR spectrum of **11p** (400 MHz, $CDCl_3$).

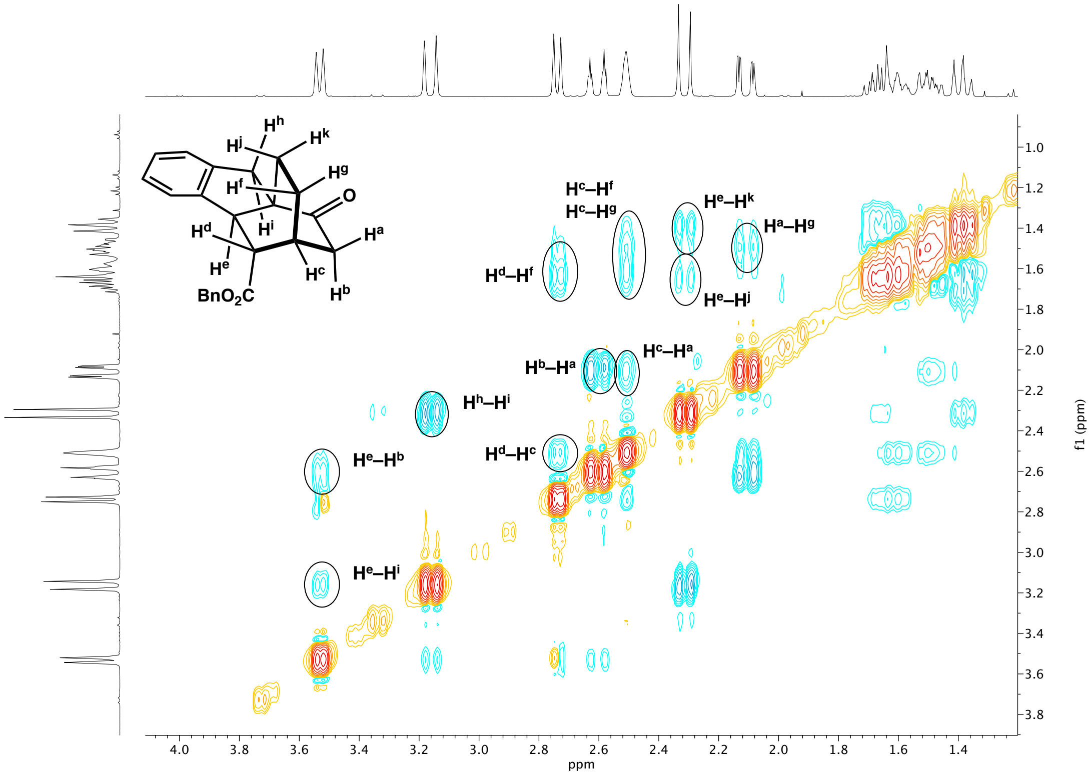

Figure S45. ^{1}H-^{1}H NOESY NMR spectrum of **11p** (400 MHz, $CDCl_3$).

*Compound **11q**:*

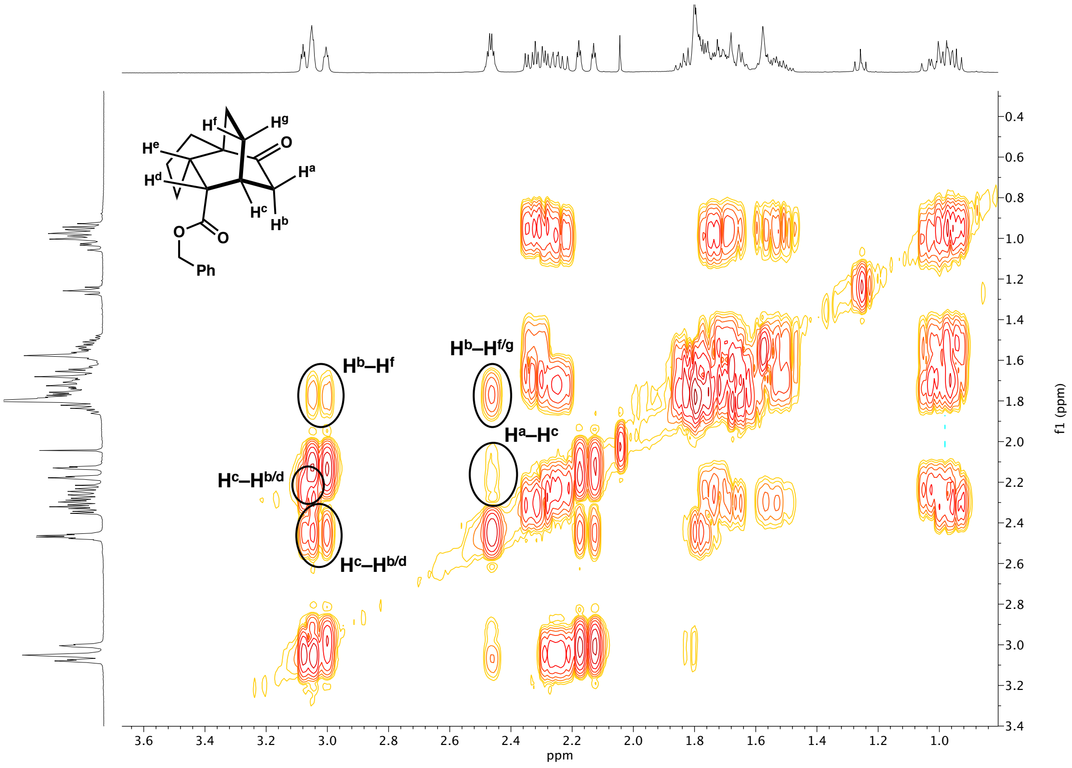

Figure S46. ^{1}H-^{1}H COSY NMR spectrum of **11q** (400 MHz, $CDCl_3$).

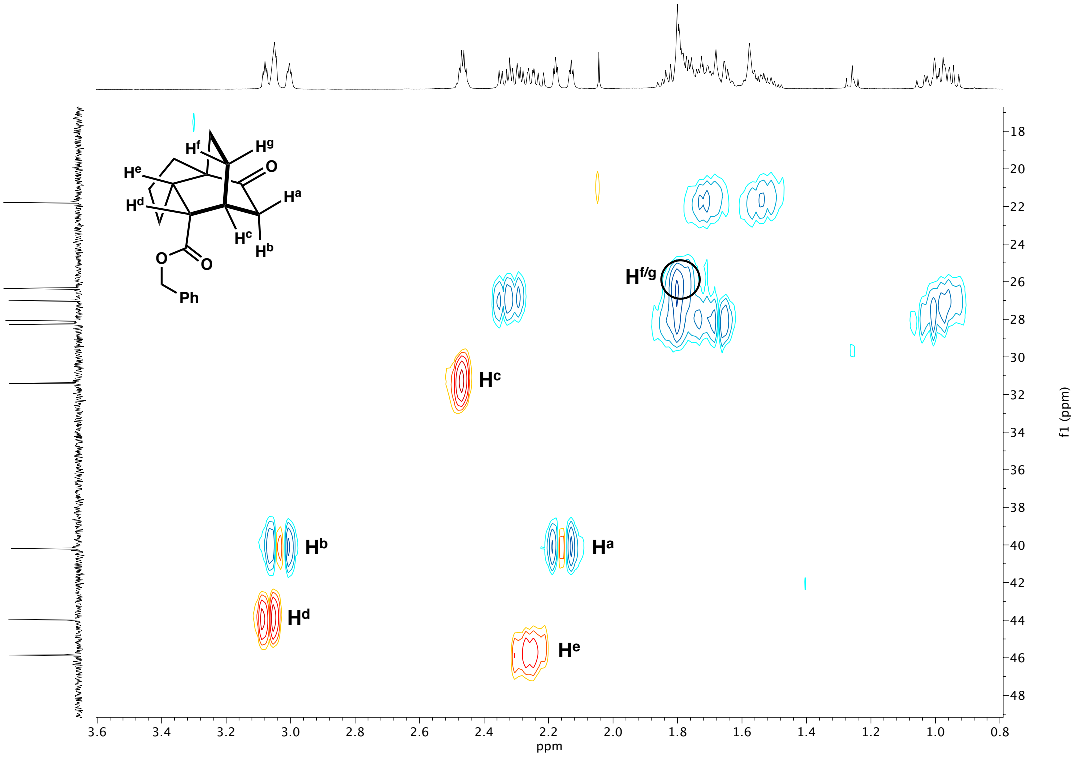

Figure S47. ^{1}H-^{13}C HSQC NMR spectrum of **11q** (400 MHz, $CDCl_3$).

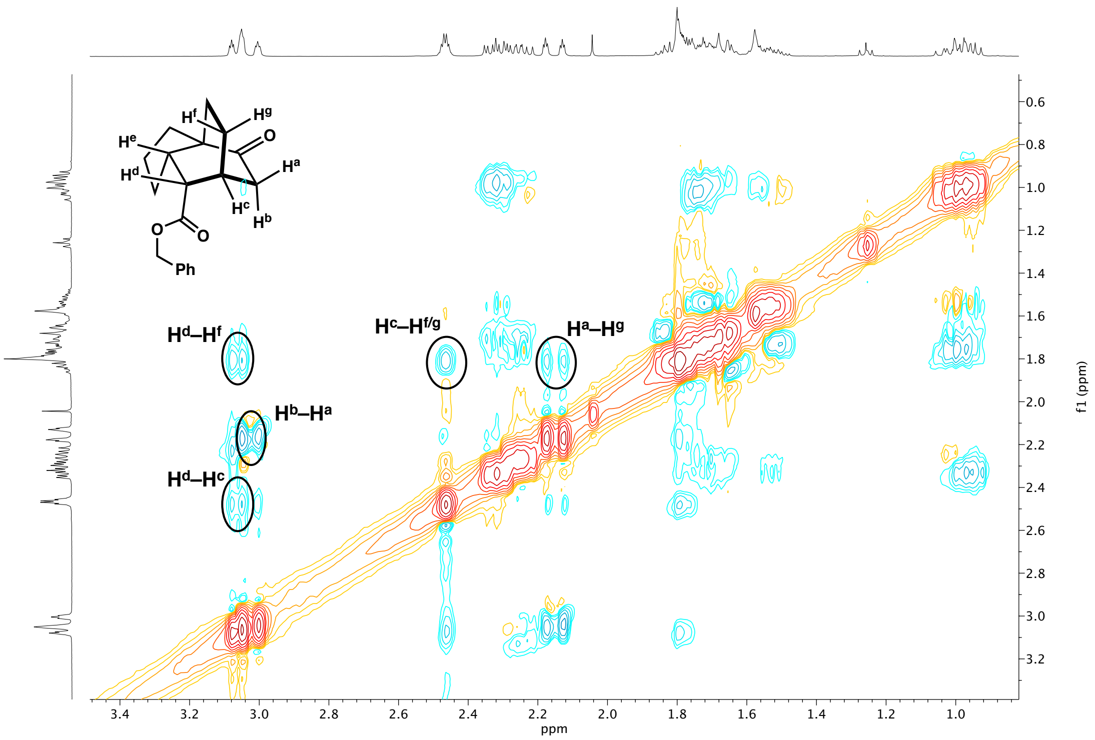

Figure S48. ^{1}H-^{1}H NOESY NMR spectrum of **11q** (400 MHz, $CDCl_3$).

*Compound **11q’**:*

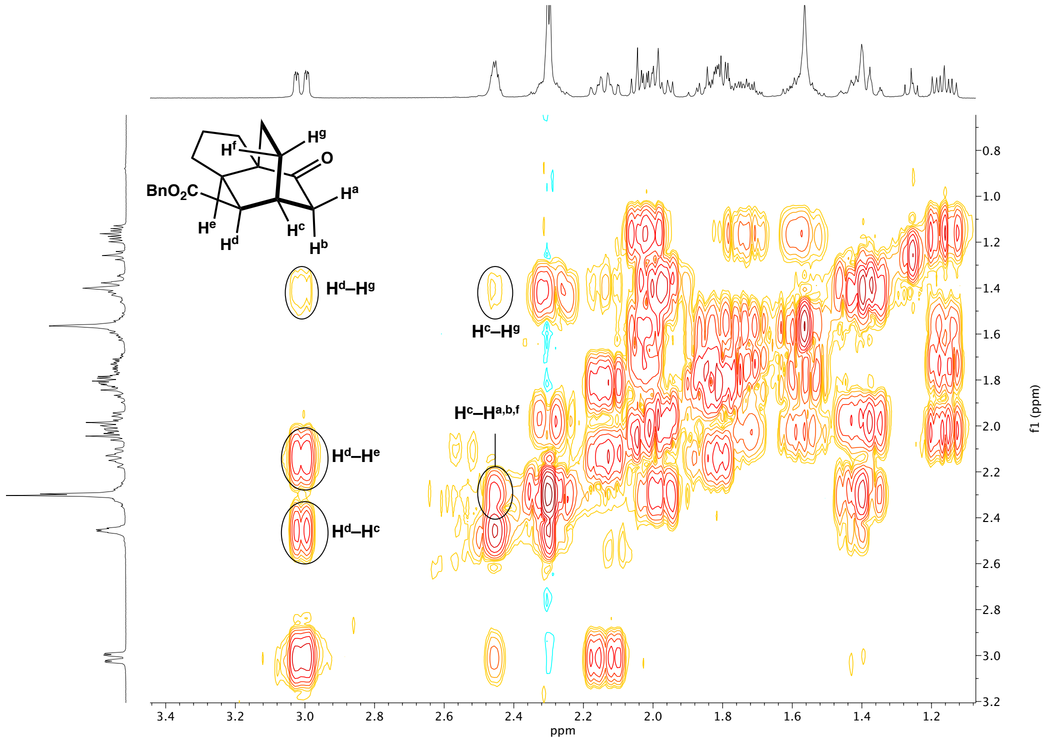

Figure S49. ^{1}H-^{1}H COSY NMR spectrum of **11q’** (400 MHz, $CDCl_3$).

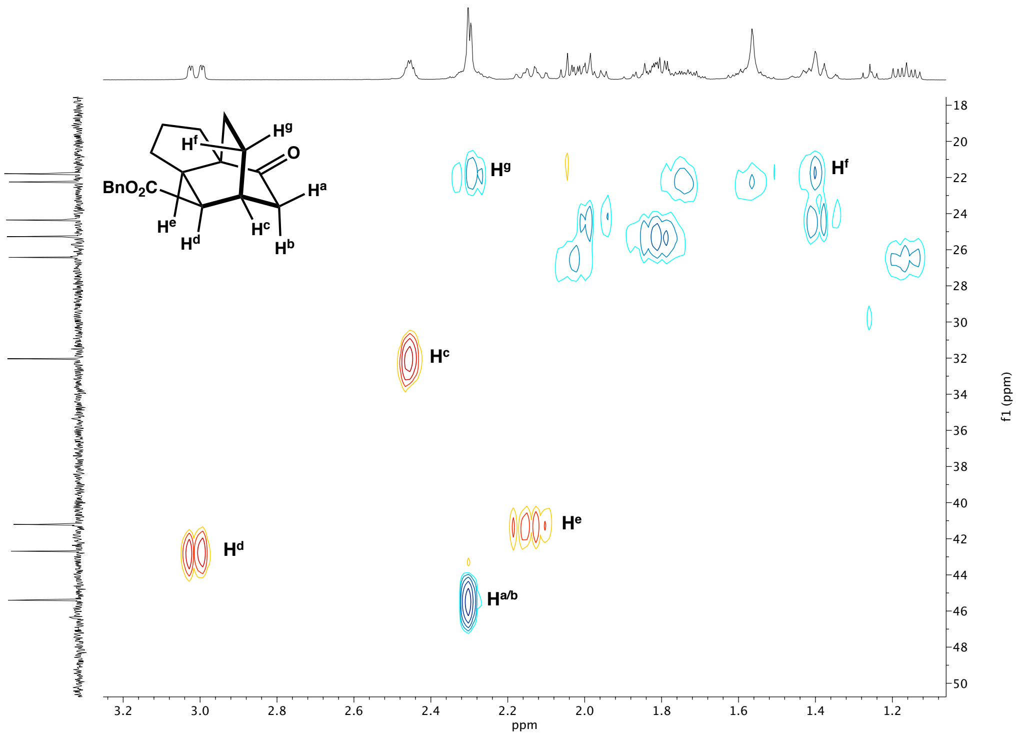

Figure S50. ^{1}H-^{13}C HSQC NMR spectrum of **11q'** (400 MHz, CDCl$_3$).

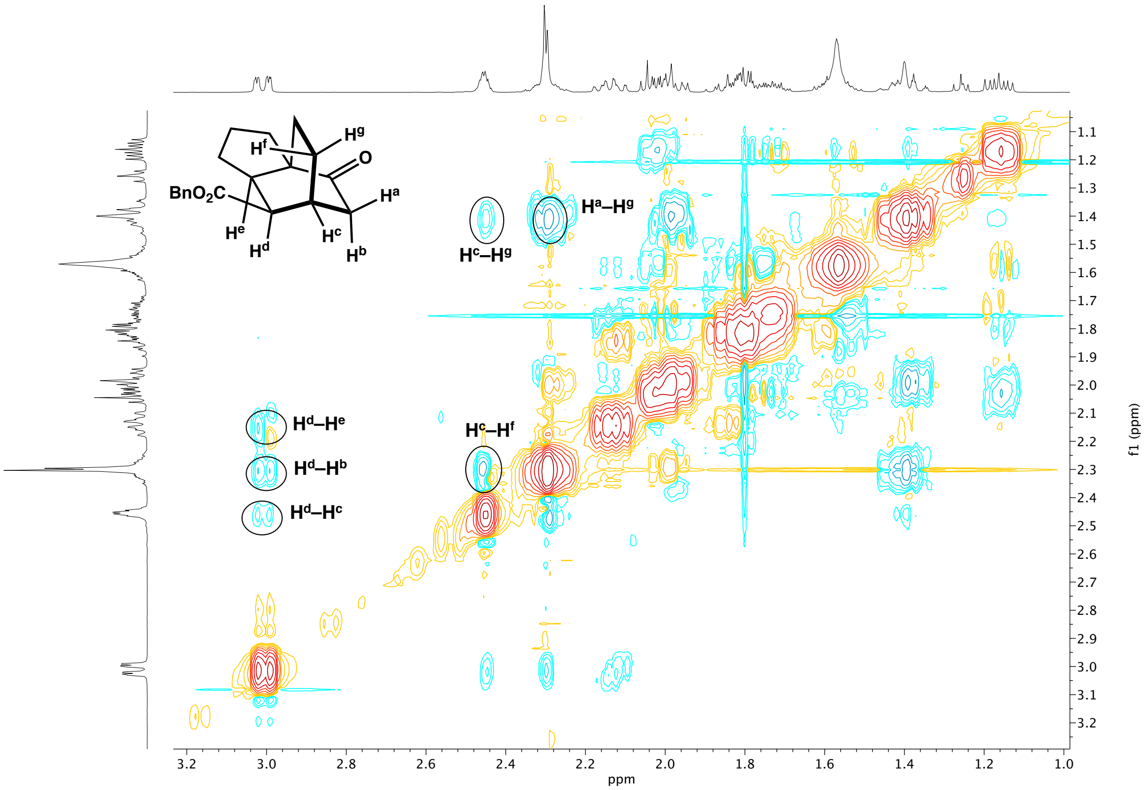

Figure S51. ^{1}H-^{1}H NOESY NMR spectrum of **11q'** (400 MHz, CDCl$_3$).

Computational details

General Notes

All quantum mechanics calculations were carried out with the ORCA program.[27] Geometry optimizations, harmonic frequency calculations, and single-point energy evaluations were carried out with density functional theory (DFT). The PBE0 functional[28] paired with Becke–Johnson damped D4 dispersion corrections[29], henceforth referred to as PBE0-D4, was used as it has proven a robust method for such systems in our prior studies.[30] For geometry optimization and harmonic frequency calculations, Pd is described by the def2-TZVP basis set[31] and the ECP28MWB small-core (18 explicit valence electrons) quasi-relativistic pseudopotential,[32] while C, H, N, and P are assigned the def2-SVP basis. Diffuse functions are added to oxygen (ma-def2-SVP). Herein, we refer to this composite basis set as BS1. Geometry optimization and harmonic frequency calculations were carried out with the CPCM implicit solvation model for toluene (PhMe, ε = 2.4).

For all calculations employing CPCM, surface charges are described by the improved Gaussian charge scheme of Neese and coworkers with a scaled Van der Waals cavity (α = 1.2).[33] All Hessians were computed analytically. Stationary points are characterized by the correct number of imaginary vibrational modes (zero for minima and one for saddle points). Intrinsic reaction coordinate (IRC) analysis confirms the nature of transition states.[34] Cartesian coordinates of all optimized structures are included as ".xyz" files are available online in a compressed in a zip file format.

Electronic energies are further refined with single-point calculations employing the PBE0-D4 functional[35] and the def2-TZVPP basis set on all atoms (with the ECP28MWB pseudopotential for Pd) with additional diffuse functions on O (ma-def2-TZVPP). This mixed basis is henceforth referred to as BS2. Solvation was accounted for with CPCM as mentioned above (PhMe, ε = 2.4). Final Gibbs free energies were obtained by applying thermodynamic corrections obtained at the optimization level of theory to these refined electronic energies. Thermodynamic corrections from harmonic frequency calculations employ the quasi-ridged rotor harmonic oscillator approach to correct for the breakdown of the harmonic oscillator approximation at low vibrational frequencies.[36] Note that free energies are adjusted to a 1 M standard state. The translational (S_{trans}) and rotational entropy (S_{rot}) contributions to the Gibbs free energy calculated for a complex in condensed phase are *ca.* 40–60% of the values obtained assuming an ideal gas.[37] As suggested in the literature, S_{trans} and S_{rot} obtained by ideal gas treatment are scaled by a factor of 0.5 to obtain the final condensed phase values.[38] Hence, the Gibbs free energy at 333.15 K is calculated as:

$$G^*_{solv} = E^{BS2}_{el,solv} + ZPE + E_{trans} + E_{rot} + E_{vib} + k_bT - T\left(S_{el} + S_{vib} + \frac{1}{2}S_{trans} + \frac{1}{2}S_{rot}\right) + \Delta G^{0\rightarrow *}$$

The resolution of identity (RI) and Chain-of-Spheres (COS) approximations are employed for efficient evaluation of Coulomb and exchange integrals, respectively.[39] The def2/J auxiliary basis[40] is employed for all atoms except oxygen, for which a suitable auxiliary was obtained via the automatic generation algorithm in the ORCA program (keyword: *AutoAux*).[41] Very fine grid settings are employed in all calculations (optimization/frequency calculations: DefGrid2, single point calculations: DefGrid3).

Conformer searching was carried out for each stationary point using the meta-dynamics-based CREST program (using GNF-FF) from the Grimme group. Duplicate conformers were removed, and low energy conformers were subsequently optimized and energies evaluated at the

cheaper PBE0-D4/def2-TZVP (Pd), ma-def2-SVP (O), def2-SVP/CPCM(PhMe)//PBE-D4/def2-TZVP (Pd), ma-def2-SV(P) (O), def2-SV(P) level of theory. The final low energy conformers were further optimized at the level of theory mentioned prior. Note that for enantiodetermining transition states (such as **TS2** and **TS3**) conformer searching also explicitly includes rotation about the Pd–O–C–C(enolate) dihedral, consideration of s-cis and s-trans ester conformations, as well as all permutations of the considered stereochemical elements.

Finally, conformational entropy[42] (entropy arising from multiple low energy thermally populated conformers) is accounted for by the *mixture of components* model of DeTar.[43] Conformational entropy (S_{conf}) is defined as:

$$S_{conf} = -R \sum \chi_i \ln(\chi_i)$$

where χ_i is the mole fraction (thermal population) of the ith conformer based on its relative free energy within the conformer ensemble. Given the computational demand for computing free energies for large ensembles of conformers, χ_i was derived from the free energies initially computed during the conformer screening process (PBE0-D4/def2-TZVP (Pd), ma-def2-SVP (O), def2-SVP/CPCM(PhMe)//PBE-D4/def2-TZVP (Pd), ma-def2-SV(P) (O), def2-SV(P))

$$G_{final} = G^{*}_{solv} - TS_{conf}$$

For the systems at hand, values of TS_{conf} (at 333.15 K) can be on the order of magnitude of a few kcal/mol.

Comparison of Barrier Heights to Inner-sphere Reductive Elimination

Employing cyclohexanone-derived Pd enolate as a model system (**S13a**–**S13c**), the barrier to inner-sphere reductive elimination was investigated while varying substitution on the allyl moiety (Figure S52).

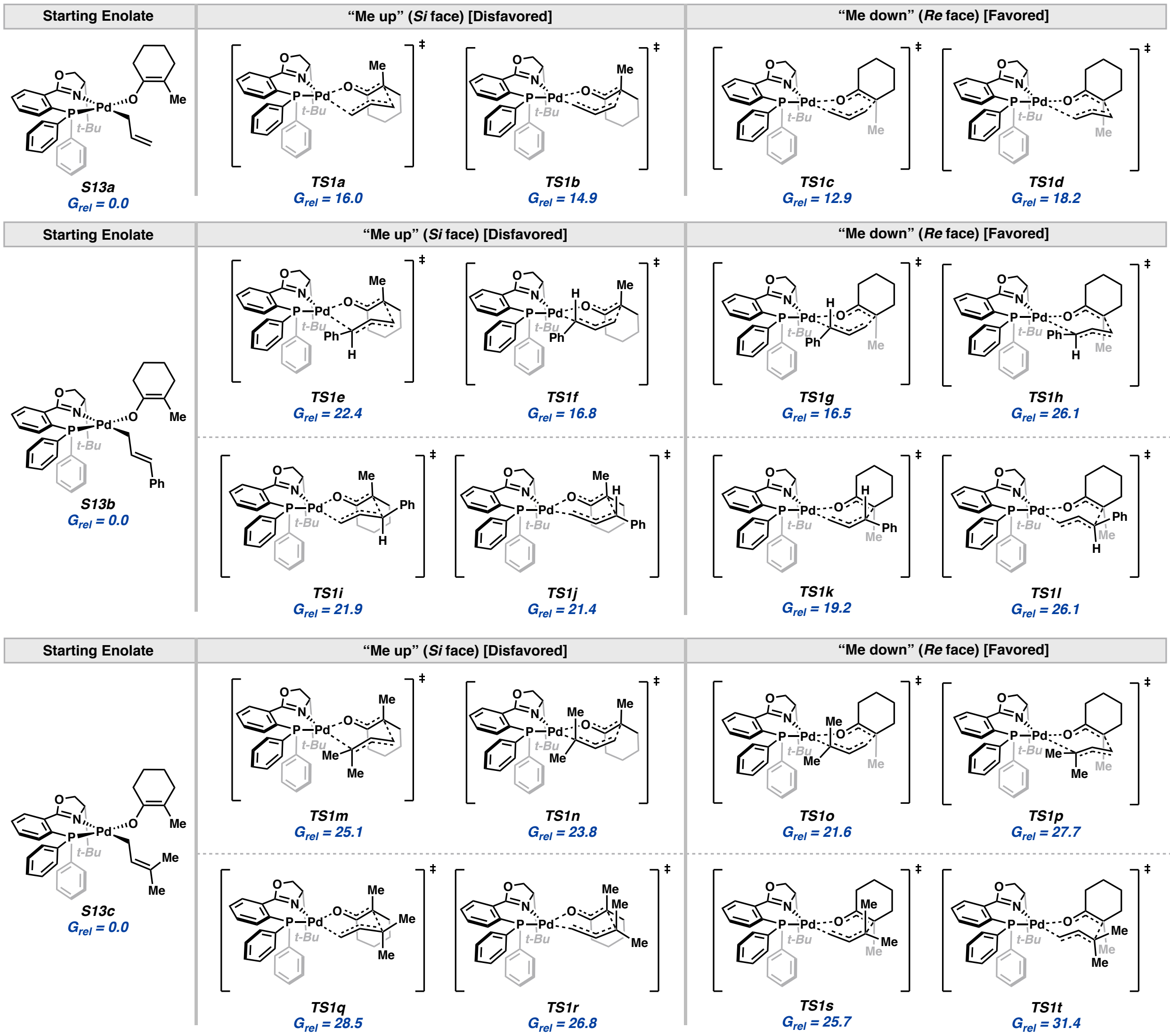

Figure S52. Relative free energies for various inner-sphere reductive elimination transition states from allyl (**S13a**), cinnamyl (**S13b**), and prenyl (**S13c**) complexes. Gibbs free energies in kcal/mol computed at the PBE0-D4/BS2/CPCM(PhMe)//PBE0-D4/BS1/CPCM(PhMe) level of theory at 333.15 K.

Mechanism of Catalyst Turnover

Of all the sampled transition states, we found the outer-sphere and N-detached inner-sphere pathways to be highly competitive and lowest in energy. Additional transition states were also explored, and the lowest energy pathway of each type of mechanism are shown in the following table.

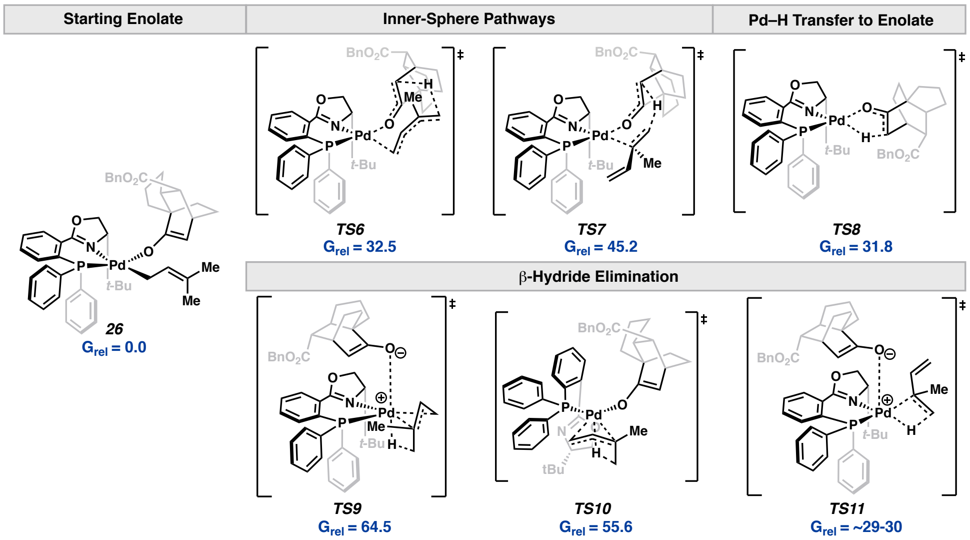

Figure S53. Relative free energies for various proton transfer transition states from post-cycloaddition enolate **26**. Gibbs free energies in kcal/mol computed at the PBE0-D4/def2-TZVP (Pd), ma-def2-SVP, def2-SVP/CPCM(PhMe)//PBE-D4/def2-TZVP (Pd), ma-def2-SV(P), def2-SV(P) level of theory at 333.15 K.

Mechanism of Premature Protonation

Analogous to the catalyst turnover mechanism, N-detached inner-sphere pathways were found to be lowest-energy for premature protonation. Of these transition states (**TS12a** and **TS12b**) that would yield enantiomeric protonation products, the lowest-energy **TS12a** provides the enantiomer consistent with the major reaction product. In addition, an outer-sphere pathway (**TS13**) was also found to be competitive.

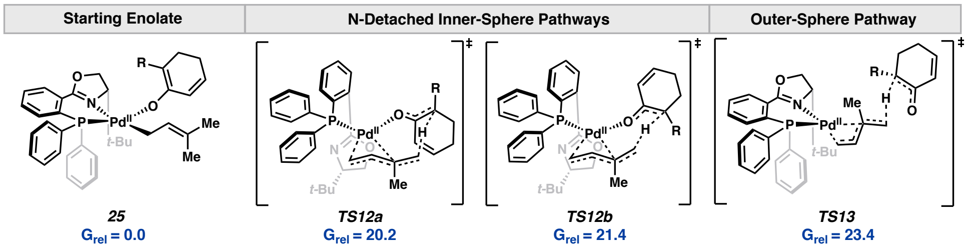

Figure S54. Relative free energies for proton transfer transition states from conjugated enolate **25**. Gibbs free energies in kcal/mol computed at the PBE0-D4/BS2/CPCM(PhMe)//PBE0-D4/BS1/CPCM(PhMe) level of theory at 333.15 K.

Intrinsic Bonding Orbital (IBO) Analysis of Inner-sphere Proton Transfer

IBO analysis along the reaction coordinate of the N-detached inner-sphere proton transfer to post-cycloaddition enolate confirms the role of prenyl as a proton source.

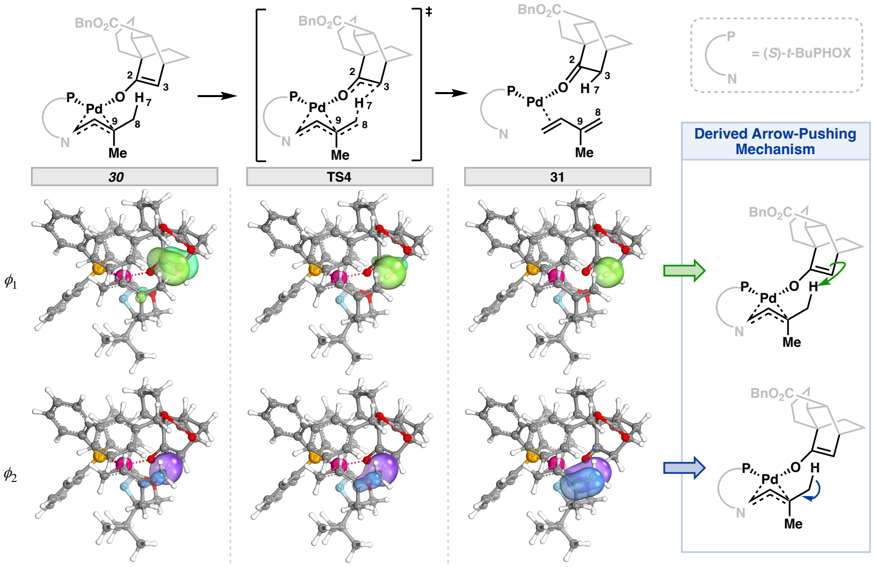

Figure S55. IBO analysis of N-detached inner-sphere mechanism and corresponding derived arrow-pushing mechanism.

pKa Calculations and Thermodynamics of Outer-Sphere Proton Transfer

The pKa values of the π-allyl Pd complex **S14** and ketones were calculated, and the results verify that the proton transfers to both pre- and post-cycloaddition enolates are thermodynamically favorable.

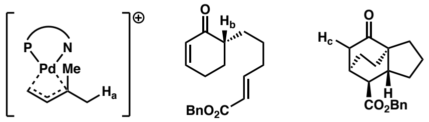

S14 *19a* *11a*

Compound	Proton	pK_a in water	pK_a in DMSO
S18	H_a	20.0	19.5
19a	H_b	30.1	30.3
11a	H_c	31.3	30.8

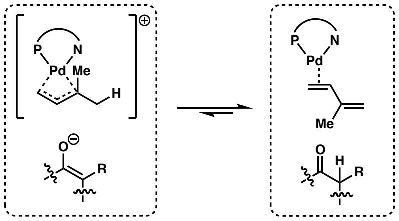

Figure S56. Computed pKa values of cationic π-allyl Pd complex **S14** and ketones **19a** and **11a**.

Enantiodetermining [4+2] cycloaddition

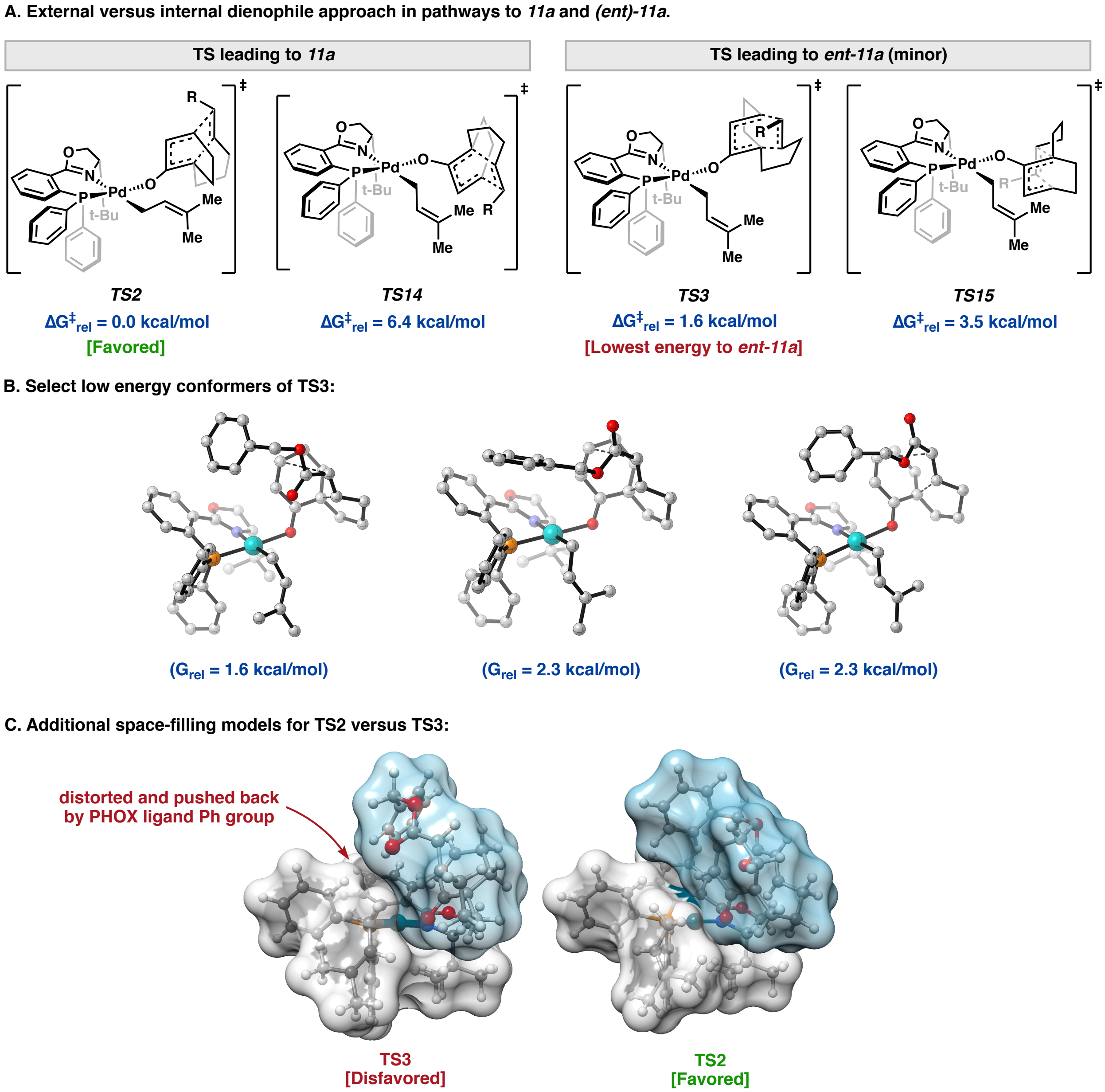

Figure S57. (A) Comparison of internal versus external dienophile approach to both enantiotopic diene faces. (B) Select low energy conformers of **TS3** (allyl isomers not pictured). (C) Additional space-filling models for TS2 and TS3.

References

1) Pangborn, A. M.; Giardello, M. A.; Grubbs, R. H.; Rosen, R. K.; Timmers, F. J. Safe and Convenient Procedure for Solvent Purification. *Organometallics* **1996**, *15*, 1518–1520.
2) McDougal, N. T.; Streuff, J.; Mukherjee, H.; Virgil, S. C.; Stoltz, B. M. Rapid synthesis of an electron-deficient *t*-BuPHOX ligand: cross-coupling of aryl bromides with secondary phosphine oxides. *Tetrahedron Lett*. **2010**, *51*, 5550–5554.
3) Ando, K.; Narumiya, K.; Takada, H.; Teruya, T. Z-Selective Intramolecular Horner−Wadsworth−Emmons Reaction for the Synthesis of Macrocyclic Lactones. *Org. Lett.* **2010**, *12*, 1460–1463.
4) Shelkov, R.; Nahmany, M.; Melman, A. Selective Esterifications of Alcohols and Phenols through Carbodiimide Couplings. *Org. Biomol. Chem.* **2004**, *2*, 397–401.
5) Taguchi, T.; Sasaki, H.; Shibuya, A.; Morikawa, T. Regio- and Stereoselective Synthesis of Gem-Difluorocyclopropanes Using 4-Bromo-4,4-Difluorocrotonate. *Tetrahedron Lett.* **1994**, *35*, 913–916.
6) Fornicola, R. S.; Subburaj, K.; Montgomery, J. A New Entry to the Isogeissoschizoid Skeleton. *Org. Lett.* **2002**, *4*, 615–617.
7) Fillion, E.; Dumas, A. M. Synthesis of Fused 4,5-Disubstituted Indole Ring Systems by Intramolecular Friedel−Crafts Acylation of 4-Substituted Indoles. *J. Org. Chem.* **2008**, *73*, 2920–2923.
8) Brandt, D.; Dittoo, A.; Bellosta, V.; Cossy, J. Synthetic Approach to Wortmannilactone C. *Org. Lett.* **2015**, *17*, 816–819.
9) Fulton, T. J.; Cusumano, A. Q.; Alexy, E. J.; Du, Y. E.; Zhang, H.; Houk, K. N.; Stoltz, B. M. Global Diastereoconvergence in the Ireland–Claisen Rearrangement of Isomeric Enolates: Synthesis of Tetrasubstituted α-Amino Acids. *J. Am. Chem. Soc.* **2020**, *142*, 21938–21947.
10) Touchard, F. P.; Capelle, N.; Mercier, M. Efficient and Scalable Protocol for the Z-Selective Synthesis of Unsaturated Esters by Horner–Wadsworth–Emmons Olefination. *Adv. Synth. Catal.* **2005**, *347*, 707–711.
11) Romano, C.; Fiorito, D.; Mazet, C. Remote Functionalization of α,β-Unsaturated Carbonyls by Multimetallic Sequential Catalysis. *J. Am. Chem. Soc.* **2019**, *141*, 16983–16990.
12) Sureshbabu, R.; Saravanan, V.; Dhayalan, V.; Mohanakrishnan, A. K. Lewis Acid Mediated One-Pot Synthesis of Aryl/Heteroaryl-Fused Carbazoles Involving a Cascade Friedel–Crafts Alkylation/Electrocyclization/Aromatization Reaction Sequence. *Eur. J. Org. Chem.* **2011**, 922–935.

13) Mukaiyama, T.; Matsuo, J.; Yanagisawa, M. A New and Efficient Method for Oxidation of Various Alcohols by Using N-Tert-Butyl Phenylsulfinimidoyl Chloride. *Chem. Lett.* **2000**, *29*, 1072–1073.

14) Inanaga, K.; Wollenburg, M.; Bachman, S.; Hafeman, N. J.; Stoltz, B. M. Catalytic Enantioselective Synthesis of Carbocyclic and Heterocyclic Spiranes via a Decarboxylative Aldol Cyclization. *Chem. Sci.* **2020**, *11*, 7390–7395.

15) Mohr, J. T.; Behenna, D. C.; Harned, A. M.; Stoltz, B. M. Deracemization of Quaternary Stereocenters by Pd-Catalyzed Enantioconvergent Decarboxylative Allylation of Racemic β-Ketoesters. *Angew. Chem. Int. Ed.* **2005**, *44*, 6924–6927.

16) Hierold, J.; Lupton, D. W. Synthesis of Spirocyclic γ-Lactones by Cascade Beckwith–Dowd Ring Expansion/Cyclization. *Org. Lett.* **2012**, *14*, 3412–3415.

17) Trost, B. M.; Xu, J. The O-Acylation of Ketone Enolates by Allyl 1H-Imidazole-1-Carboxylate Mediated with Boron Trifluoride Etherate: A Convenient Procedure for the Synthesis of Substituted Allyl Enol Carbonates. *J. Org. Chem.* **2007**, *72*, 9372–9375.

18) Prepared following ref. 17 with 3-(methyl-d_3)but-2-en-4,4,4-d_3-1-ol (Mosaferi, S.; Jelley, R. E.; Fedrizzi, B.; Barker, D. Scalable Synthesis of the Aroma Compounds D6-β-Ionone and D6-β-Cyclocitral for Use as Internal Standards in Stable Isotope Dilution Assays. *Tetrahedron Lett.* **2020**, *61*, 152642.).

19) Shimizu, I.; Tsuji, J. Palladium-Catalyzed Decarboxylation-Dehydrogenation of Allyl β-Oxo Carboxylates and Allyl Enol Carbonates as a Novel Synthetic Method for α-Substituted α,β-Unsaturated Ketones. *J. Am. Chem. Soc.* **1982**, *104*, 5844–5846.

20) Knapp, S.; Yang, C.; Pabbaraja, S.; Rempel, B.; Reid, S.; Withers, S. G. Synthesis and Kinetic Analysis of the N-Acetylhexosaminidase Inhibitor XylNAc-Isofagomine. *J. Org. Chem.* **2005**, *70*, 7715–7720.

21) Srikrishna, A.; Kumar, P.P. Claisen Rearrangement Based Methodology for the Spiroannulation of a Cyclopentane Ring. Formal Total Synthesis of (±)-Acorone and Isoacorones. *Tetrahedron* **2000**, *56*, 8189–8195.

22) Szczesńiak, P.; Pieczykolan, M.; Steck, S. The Synthesis of α,α-Disubstituted α-Amino Acids via Ichikawa Rearrangement. *J. Org. Chem.* **2016**, *81*, 1057–1074.

23) Hou, H.; Xu, Y.; Yang, H.; Chen, X.; Yan, C.; Shi, Y.; Zhu, S. Visible-Light Mediated Hydrosilylative and Hydrophosphorylative Cyclizations of Enynes and Dienes. *Org. Lett.* **2020**, *22*, 1748-1753.

24) Yang, J.; Lu, K.; Li, C.; Zhao, Z.; Zhang, X.; Zhang, F.; Tu, Y. Chiral 1,2,3-Triazolium Salt Catalyzed Asymmetric Mono- and Dialkylation of 2,5-Diketopiperazines with the Construction of Tetrasubstituted Carbon Centers. *Angew. Chem. Int. Ed.* **2022**, *61*, e202114129.

25) **Schrödinger Release 2021-4:** MacroModel, Schrödinger, LLC, New York, NY, 2021.

26) Bochevarov, A.D.; Harder, E.; Hughes, T.F.; Greenwood, J.R.; Braden, D.A.; Philipp, D.M.; Rinaldo, D.; Halls, M.D.; Zhang, J.; Friesner, R.A., "Jaguar: A high-performance quantum

chemistry software program with strengths in life and materials sciences," *Int. J. Quantum Chem.*, **2013**, *113*, 2110-2142.

27) (a) Neese, F. Software Update: The ORCA Program System, Version 4.0. *Wiley Interdiscip. Rev.: Comput. Mol. Sci.* **2018,** *8*, No. e1327. (b) Neese, F. The ORCA Program System. *Wiley Interdiscip. Rev.: Comput. Mol. Sci.* **2012**, *2*, 73−78.

28) Adamo, C.; Barone, V. Toward Reliable Density Functional Methods without Adjustable Parameters: The PBE0 Model. *J. Chem. Phys.* **1999**, *110*, 6158−6170.

29) (a) Caldeweyher, E.; Ehlert, S.; Hansen, A.; Neugebauer, H.; Spicher, S.; Bannwarth, C.; Grimme, S. A Generally Applicable Atomic-Charge Dependent London Dispersion Correction. *J. Chem. Phys.* **2019**, *150*, 154122. (b) Caldeweyher, E.; Bannwarth, C.; Grimme, S. Extension of the D3 Dispersion Coefficient Model. *J. Chem. Phys.* **2017**, *147*, 034112.

30) Cusumano, A. Q.; Goddard, W. A. I.; Stoltz, B. M. The Transition Metal Catalyzed [σ2s + σ2s + π2s + π2s] Pericyclic Reaction: Woodward–Hoffmann Rules, Aromaticity, and Electron Flow. *J. Am. Chem. Soc.* **2020**, *142*, 19033–19039.

31) Weigend, F.; Ahlrichs, R. Balanced Basis Sets of Split Valence, Triple Zeta Valence and Quadruple Zeta Valence Quality for H to Rn: Design and Assessment of Accuracy. *Phys. Chem. Chem. Phys.* **2005**, *7*, 3297–3305.

32) Peterson, K. A.; Figgen, D.; Goll, E.; Stoll, H.; Dolg, M. Systematically Convergent Basis Sets with Relativistic Pseudopotentials. II. Small-Core Pseudopotentials and Correlation Consistent Basis Sets for the Post- *d* Group 16–18 Elements. *J. Chem. Phys.* **2003**, *119*, 11113–11123.

33) Garcia-Ratés, M.; Neese, F. Effect of the Solute Cavity on the Solvation Energy and Its Derivatives within the Framework of the Gaussian Charge Scheme. *J. Comput. Chem.* **2020**, *41*, 922–939.

34) Ishida, K.; Morokuma, K.; Komornicki, A. The Intrinsic Reaction Coordinate. An Ab Initio Calculation for HNC→HCN and H−+CH4→CH4+H−. *J. Chem. Phys.* **1977**, *66*, 2153−2156.

35) Zhao, Y.; Truhlar, D. G. The M06 Suite of Density Functionals for Main Group Thermochemistry, Thermochemical Kinetics, Noncovalent Interactions, Excited States, and Transition Elements: Two New Functionals and Systematic Testing of Four M06-Class Functionals and 12 Other Functionals. *Theor. Chem. Account.* **2008**, *120*, 215–241.

36) Grimme, S. Supramolecular Binding Thermodynamics by Dispersion-Corrected Density Functional Theory. *Chem. Eur. J.* **2012**, *18*, 9955–9964.

37) Izato, Y.; Matsugi, A.; Koshi, M.; Miyake, A. A Simple Heuristic Approach to Estimate the Thermochemistry of Condensed-Phase Molecules Based on the Polarizable Continuum Model. *Phys. Chem. Chem. Phys.* **2019**, *21*, 18920–18929.

38) Finkelstein, A. V.; Janin, J. The Price of Lost Freedom: Entropy of Bimolecular Complex Formation. *Protein Engineering, Design and Selection* **1989**, *3*, 1–3.

39) Neese, F.; Wennmohs, F.; Hansen, A.; Becker, U. Efficient, Approximate and Parallel Hartree-Fock and Hybrid DFT Calculations. A 'Chain-of-Spheres' Algorithm for the Hartree-Fock Exchange. *Chem. Phys.* **2009**, *356*, 98–109.
40) Weigend, F. Accurate Coulomb-Fitting Basis Sets for H to Rn. *Phys. Chem. Chem. Phys.* **2006**, *8*, 1057–1065.
41) Stoychev, G. L.; Auer, A. A.; Neese, F. Automatic Generation of Auxiliary Basis Sets. *J. Chem. Theory Comput.* **2017**, *13*, 554–562.
42) (a) Pracht, P.; Grimme, S. Calculation of Absolute Molecular Entropies and Heat Capacities Made Simple. *Chem. Sci.* **2021**, *12*, 6551–6568. (b) Gorges, J.; Grimme, S.; Hansen, A.; Pracht, P. Towards Understanding Solvation Effects on the Conformational Entropy of Non-Rigid Molecules. *Phys. Chem. Chem. Phys.* **2022**, *24*, 12249–12259.
43) (a) DeTar, D. F. Theoretical Ab Initio Calculation of Entropy, Heat Capacity, and Heat Content. *J. Phys. Chem. A* **1998**, *102*, 5128–5141. See also: (b) Guthrie, J. P. Use of DFT Methods for the Calculation of the Entropy of Gas Phase Organic Molecules: An Examination of the Quality of Results from a Simple Approach. *J. Phys. Chem. A* **2001**, *105*, 8495–8499.

NMR and IR Spectra for New Compounds

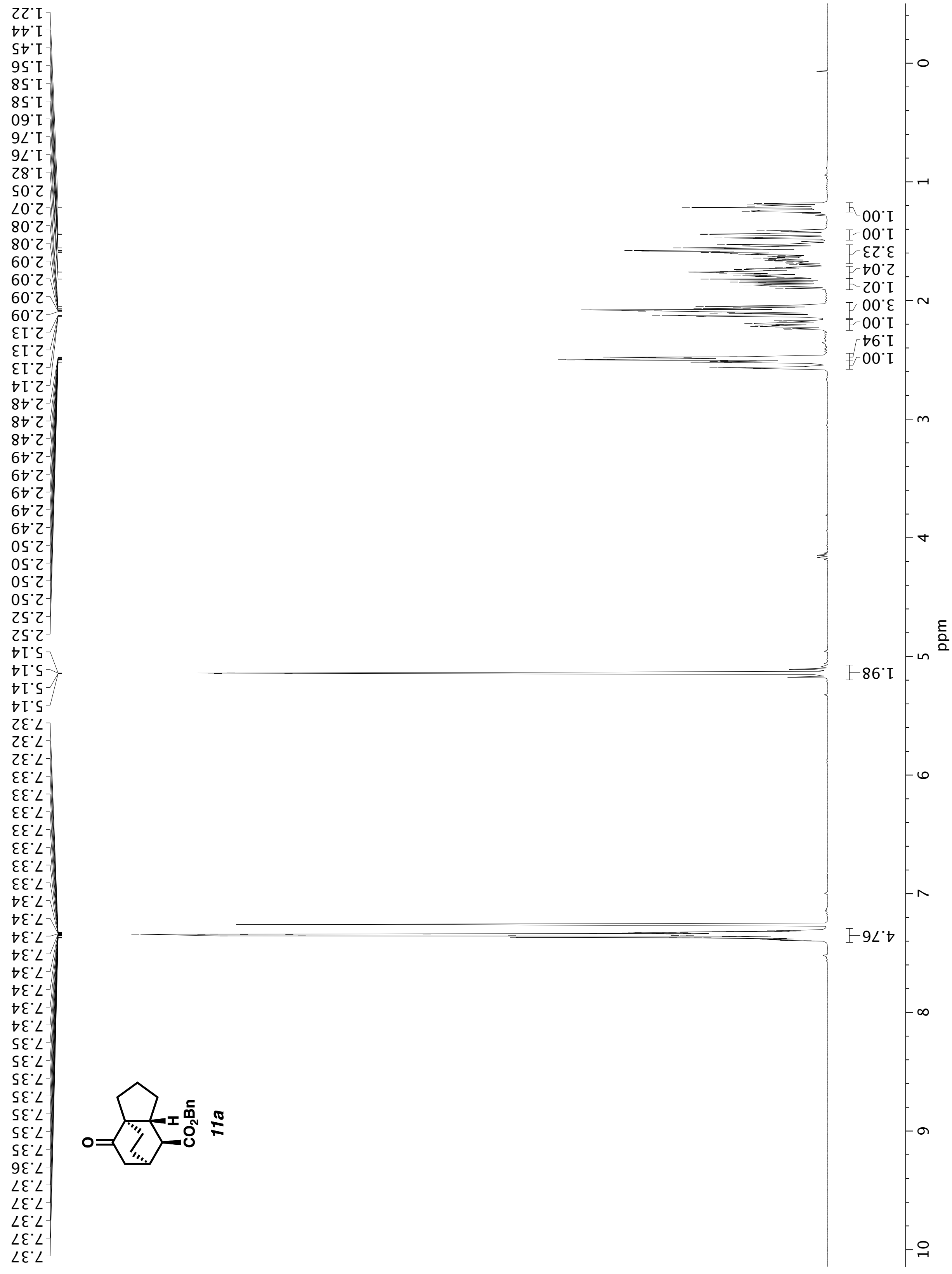

^{1}H NMR (400 MHz, $CDCl_3$) of compound **11a**.

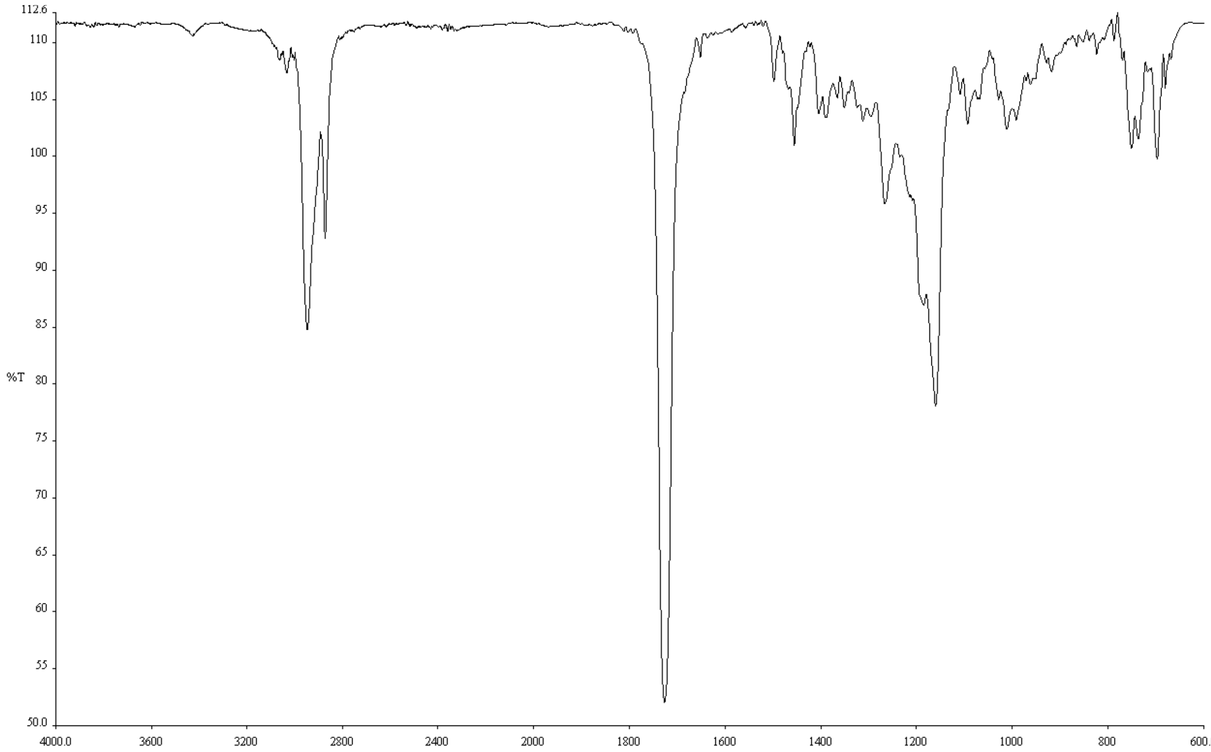

Infrared spectrum (Thin Film, NaCl) of compound **11a**.

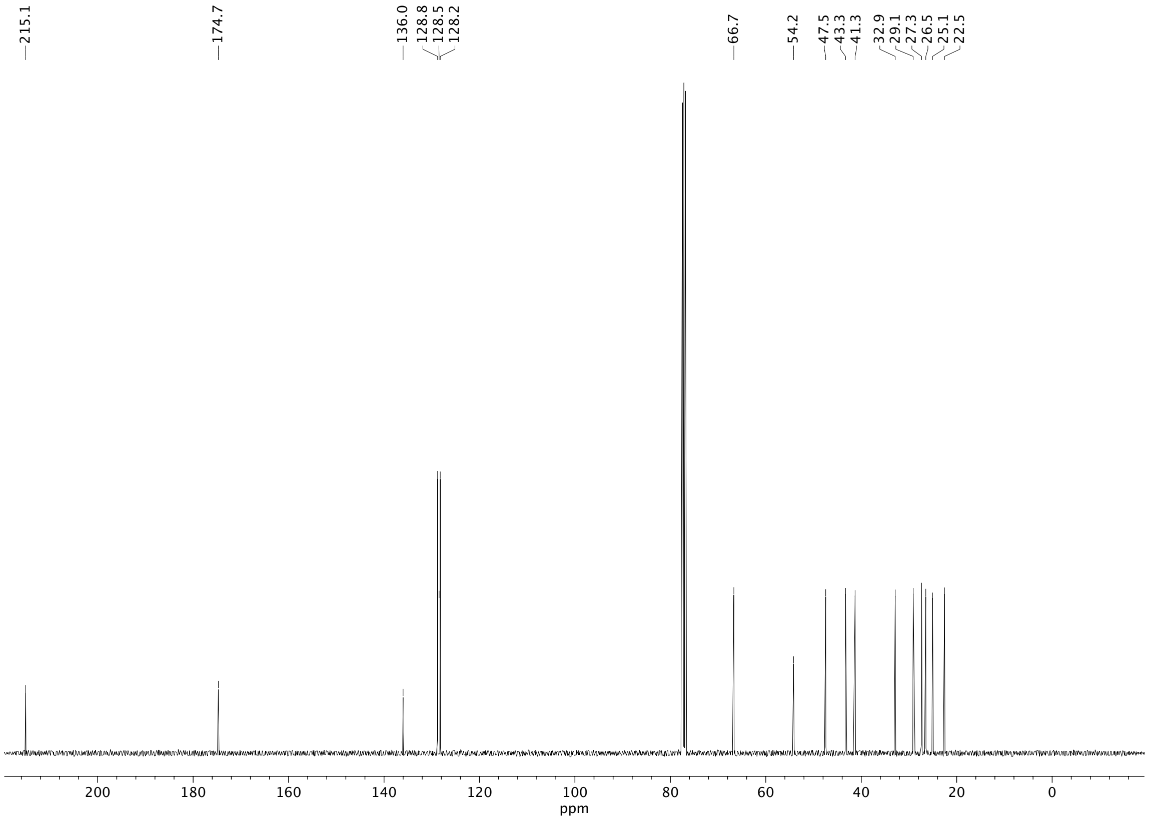

^{13}C NMR (100 MHz, $CDCl_3$) of compound **11a**.

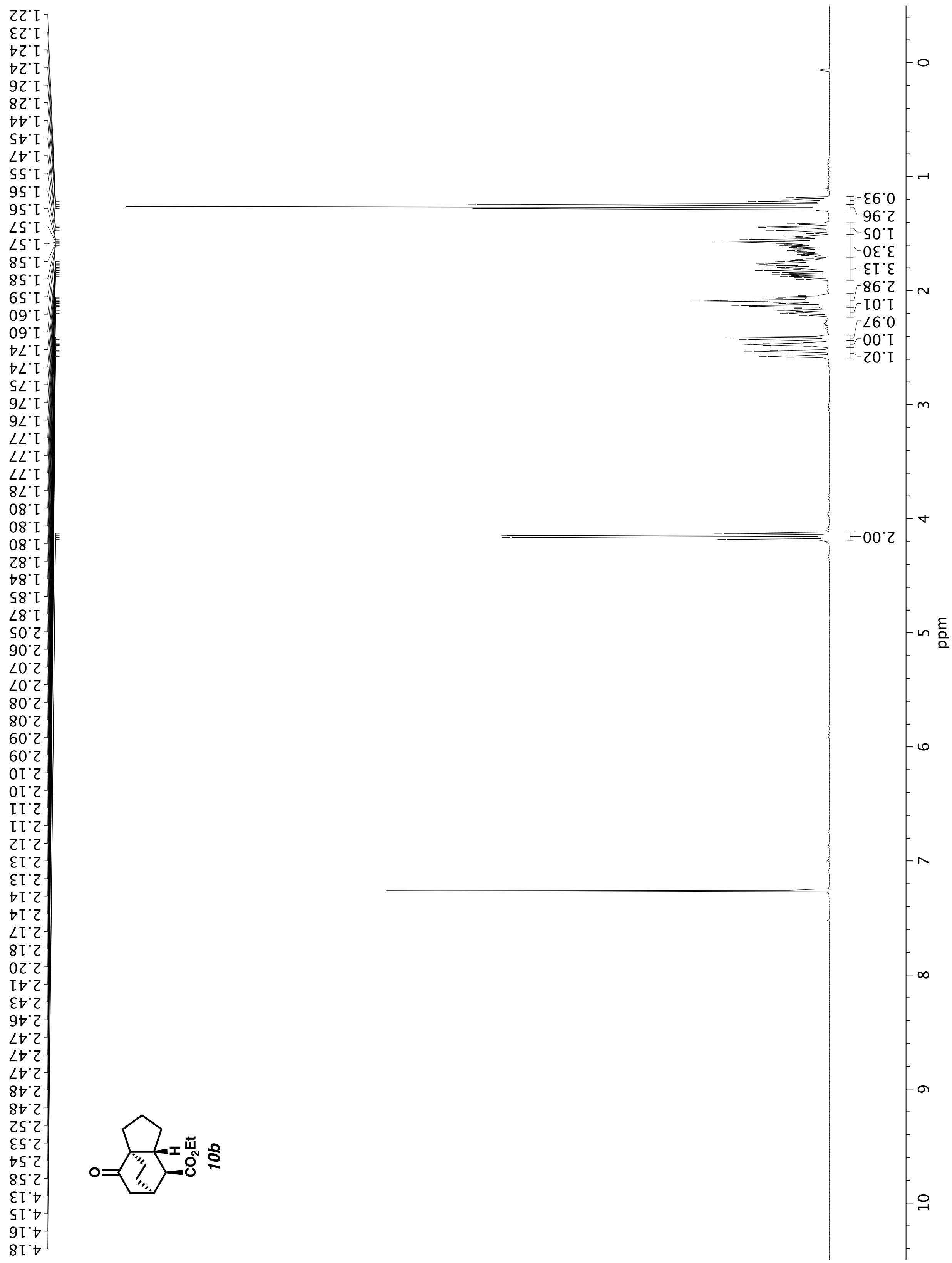

^{1}H NMR (400 MHz, $CDCl_3$) of compound **11b**.

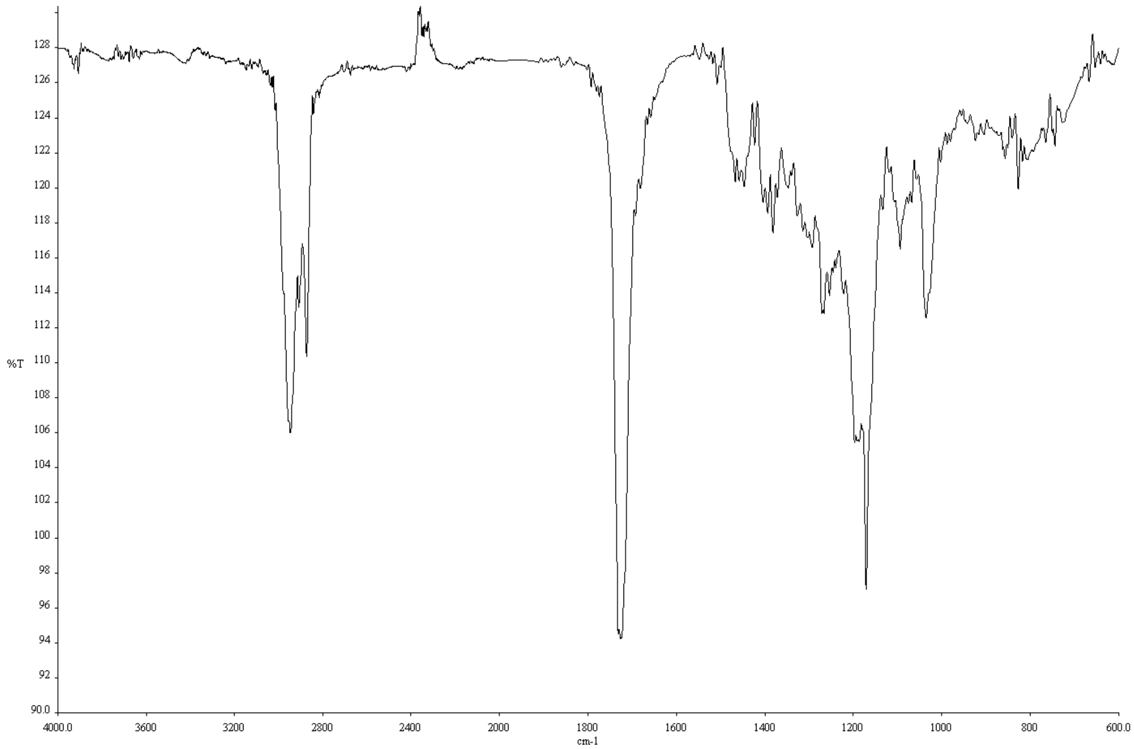

Infrared spectrum (Thin Film, NaCl) of compound **11b**.

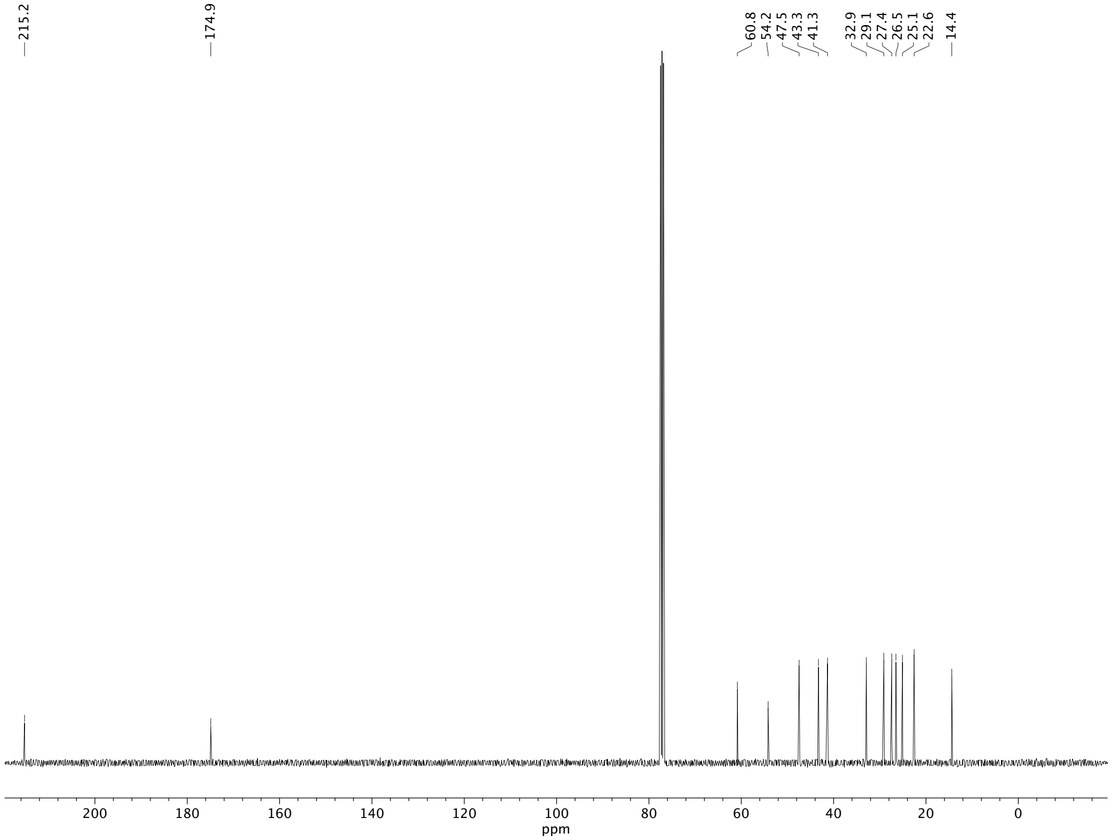

^{13}C NMR (100 MHz, $CDCl_3$) of compound **11b**.

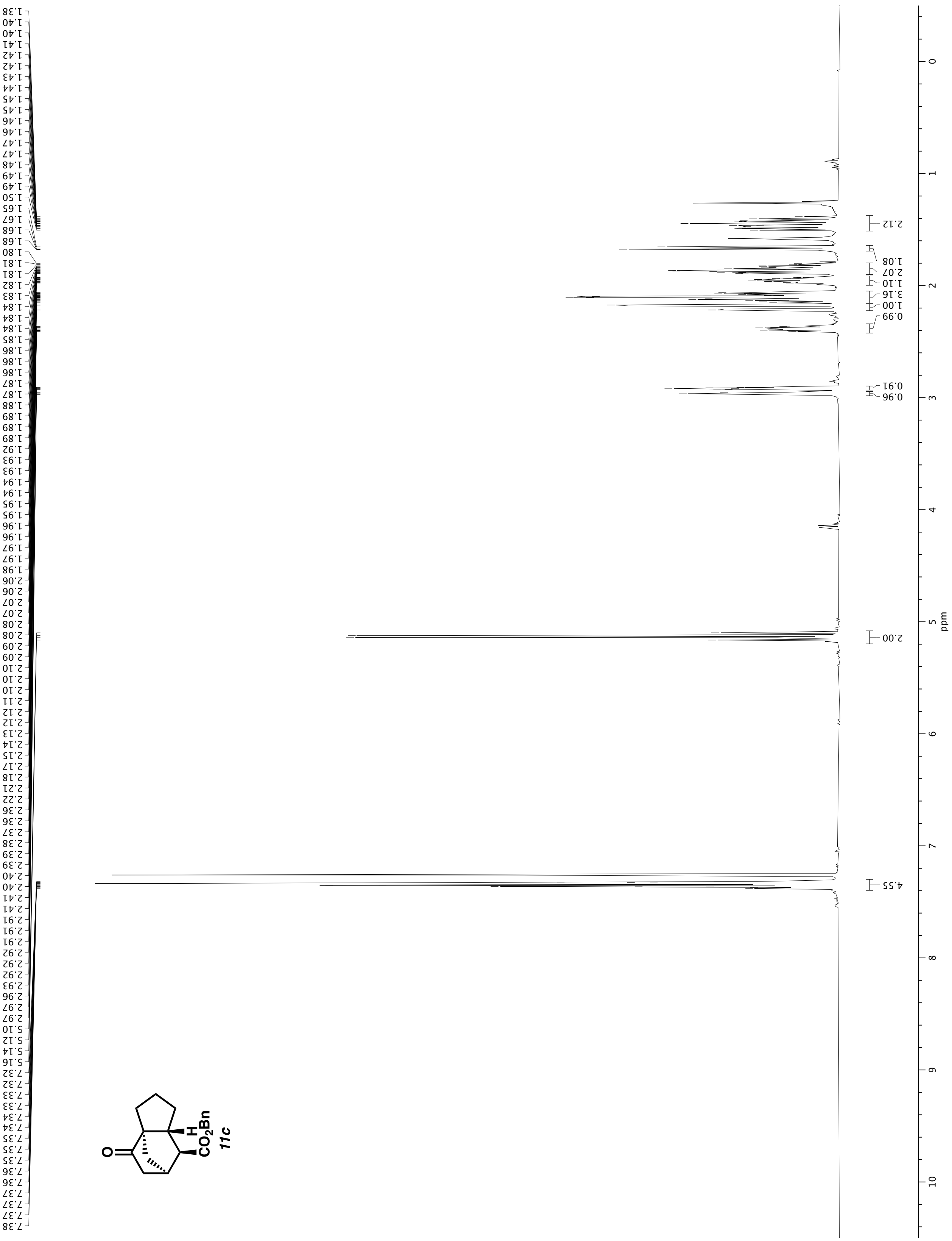

^1H NMR (400 MHz, $CDCl_3$) of compound **11c**.

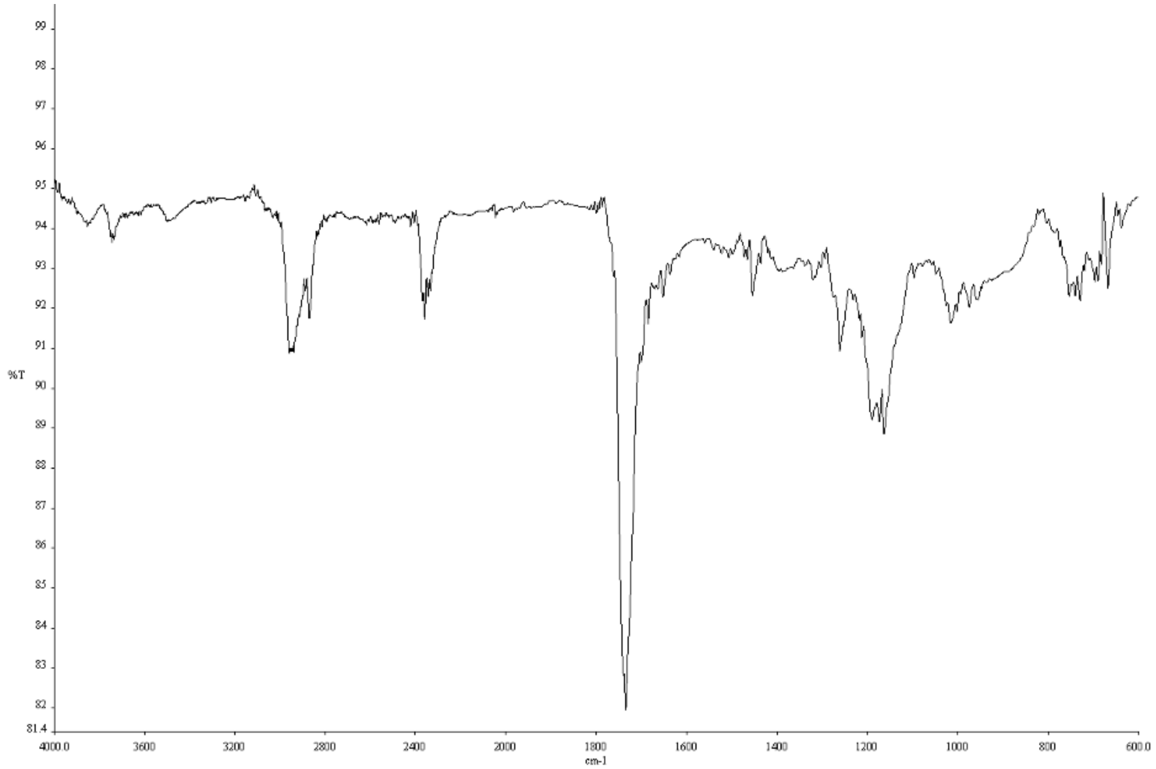

Infrared spectrum (Thin Film, NaCl) of compound **11c**.

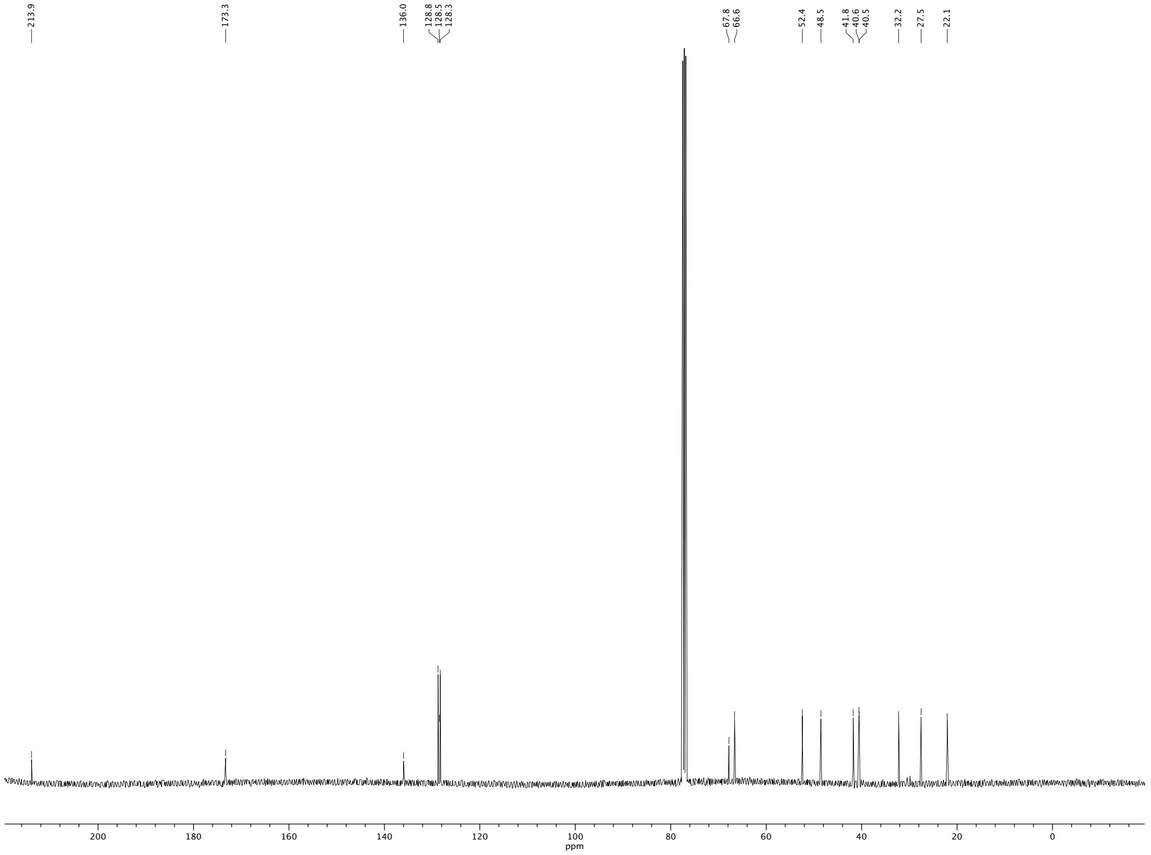

^{13}C NMR (100 MHz, $CDCl_3$) of compound **11c**.

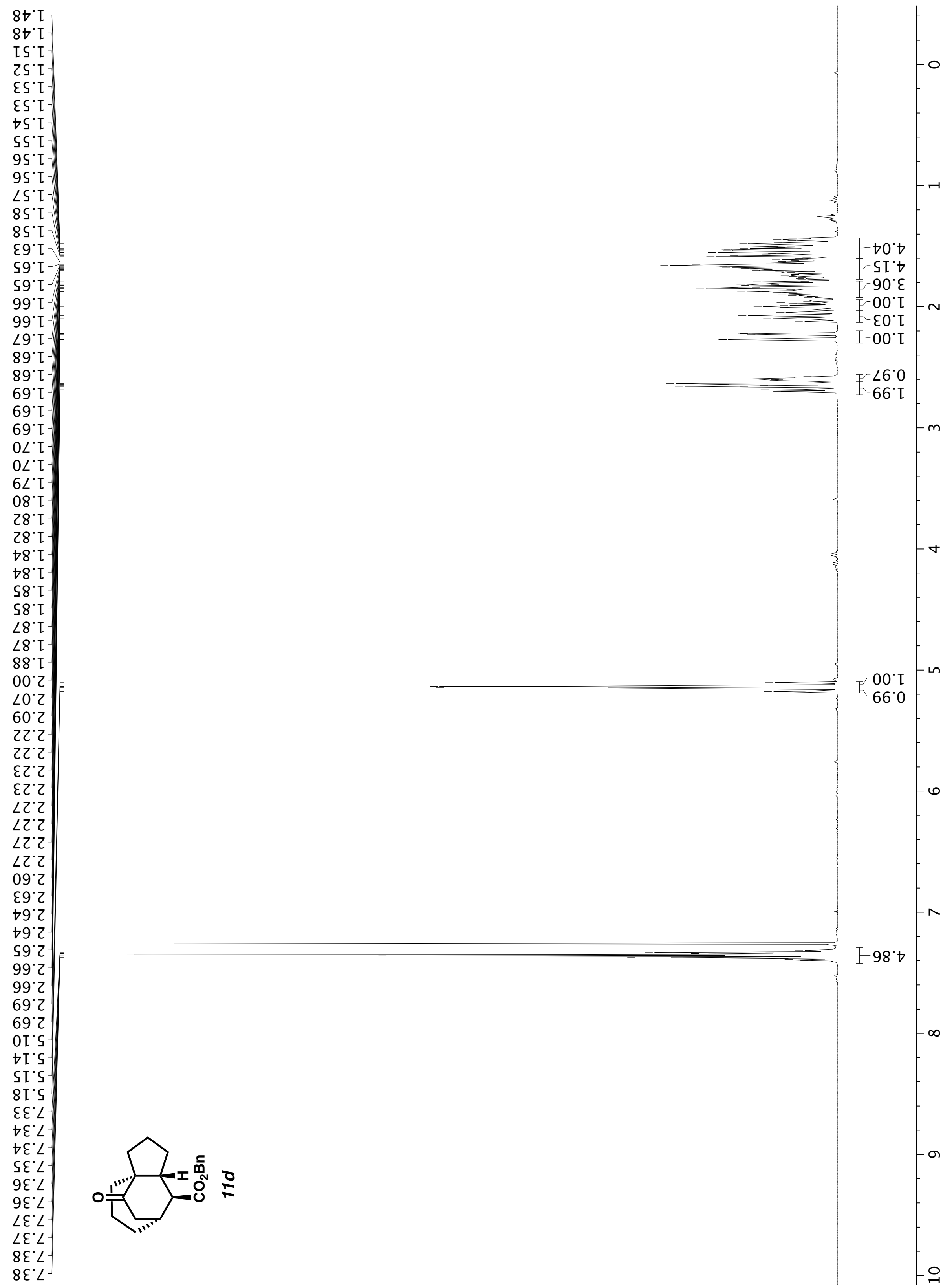

^{1}H NMR (400 MHz, $CDCl_3$) of compound **11d**.

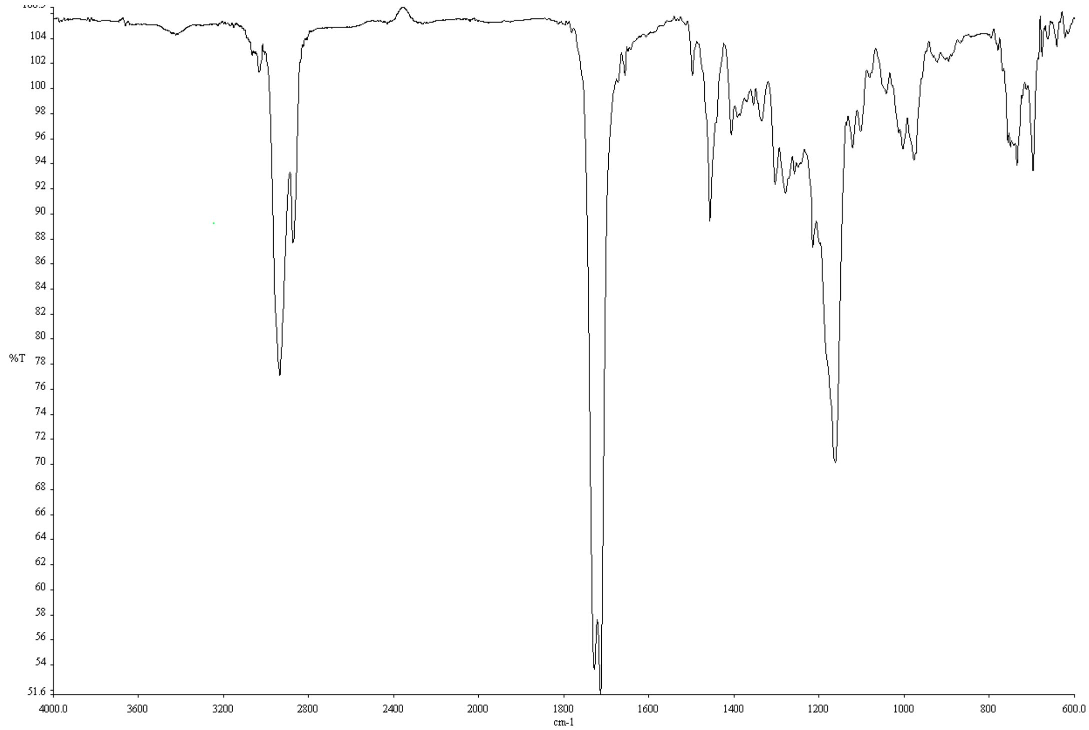

Infrared spectrum (Thin Film, NaCl) of compound **11d**.

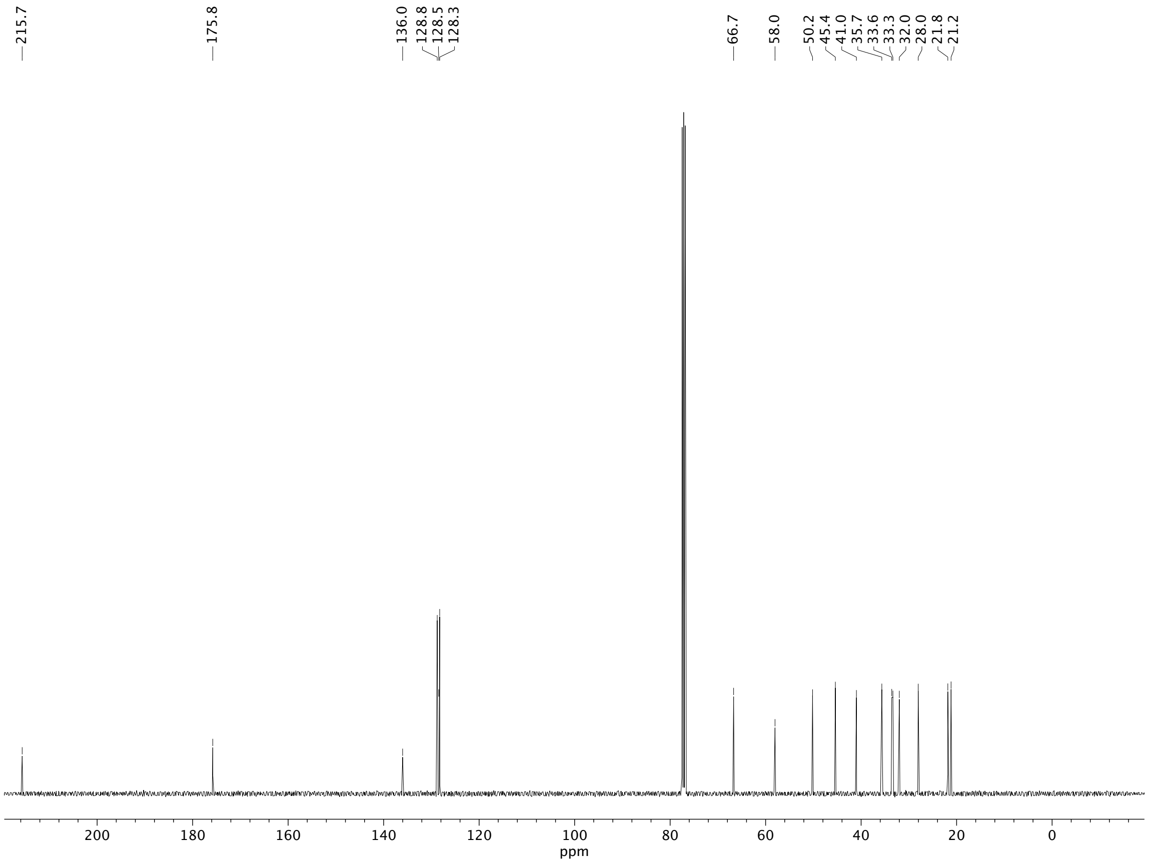

^{13}C NMR (100 MHz, $CDCl_3$) of compound **11d**.

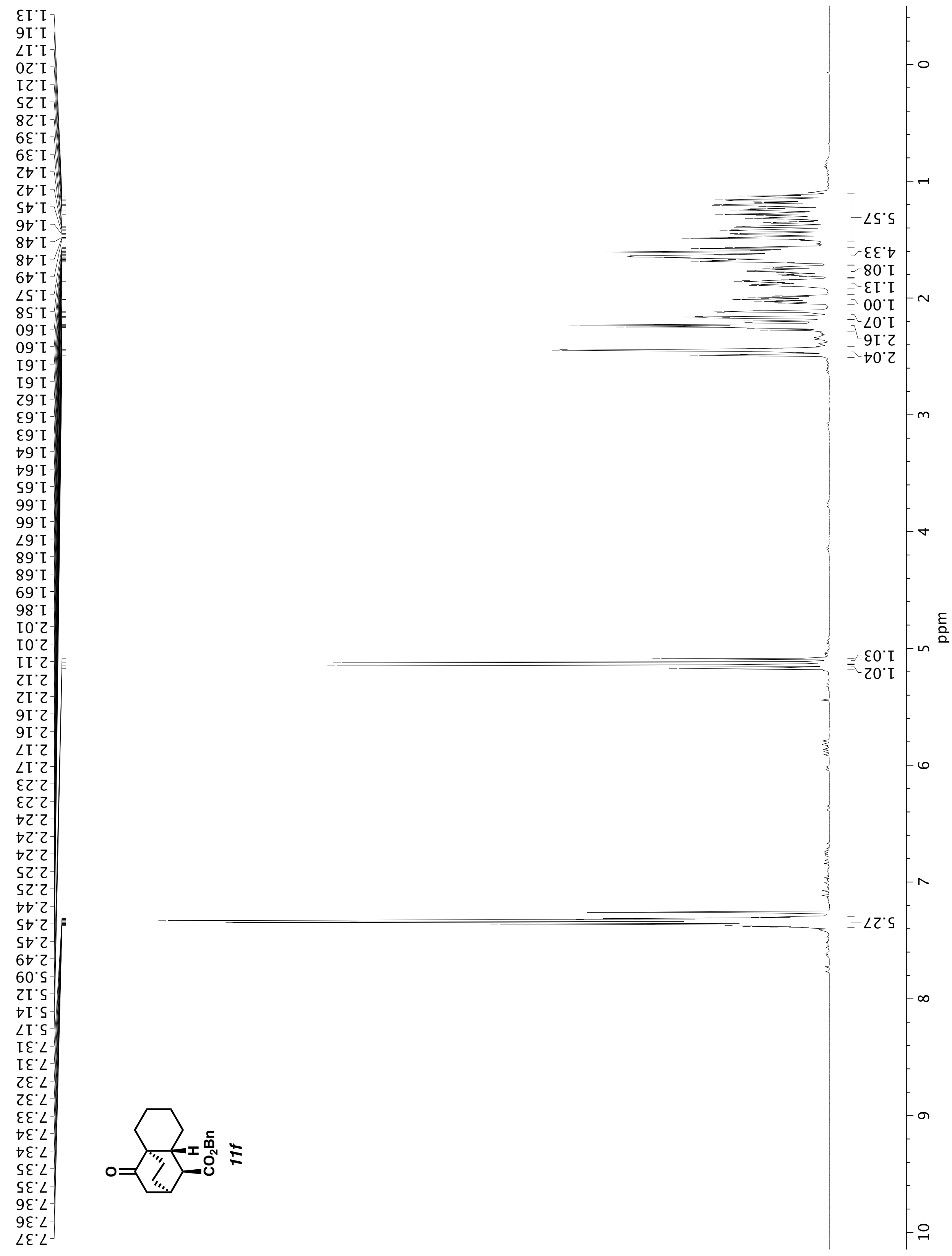

^{1}H NMR (400 MHz, $CDCl_3$) of compound **11f**.

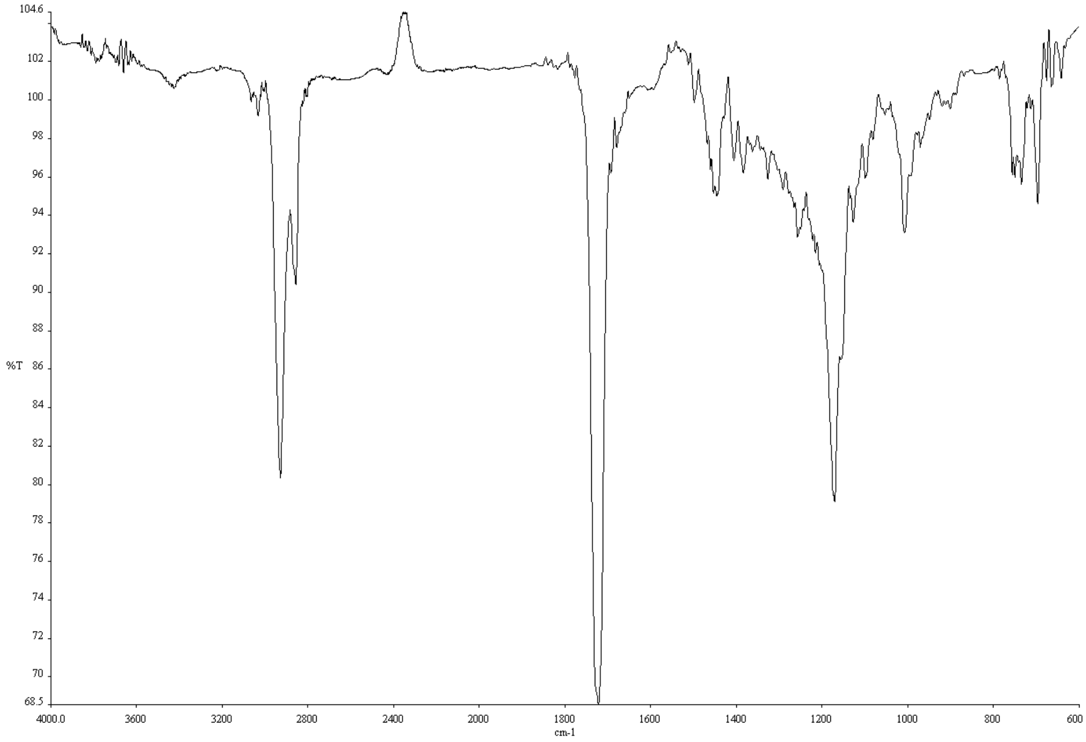

Infrared spectrum (Thin Film, NaCl) of compound **11f**.

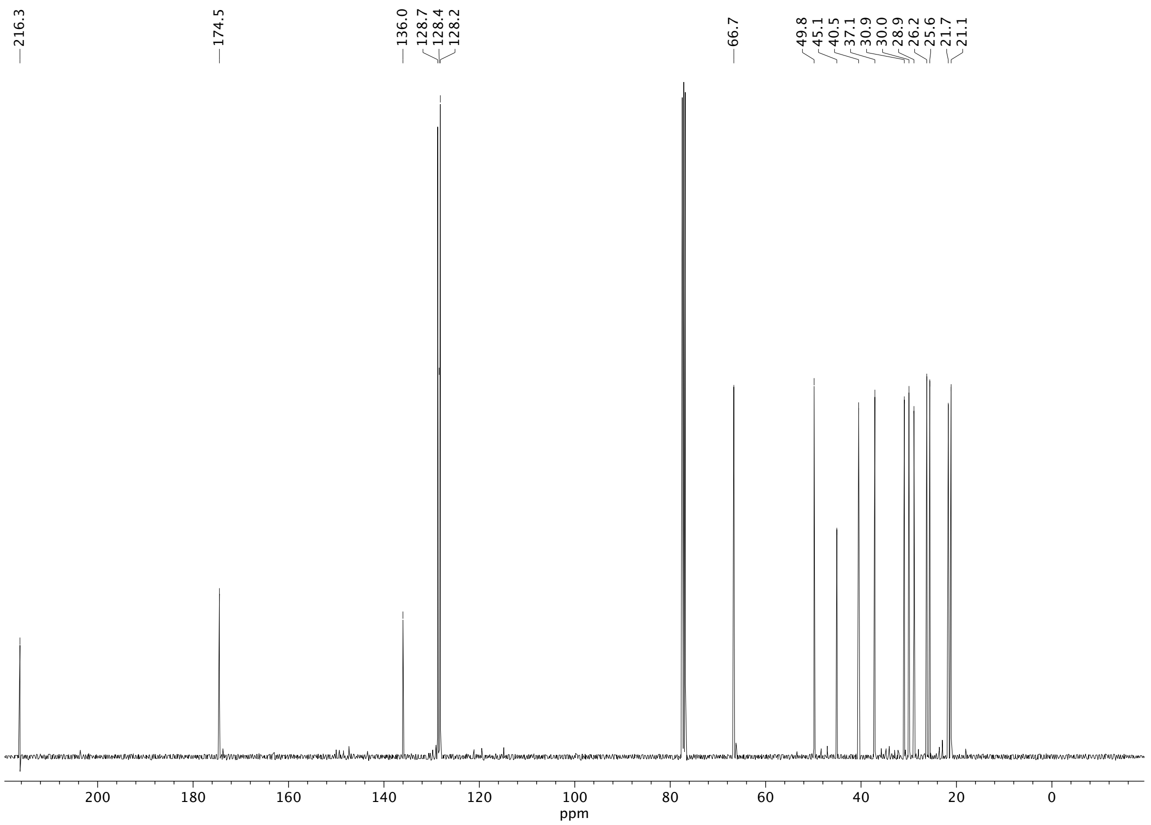

^{13}C NMR (100 MHz, $CDCl_3$) of compound **11f**.

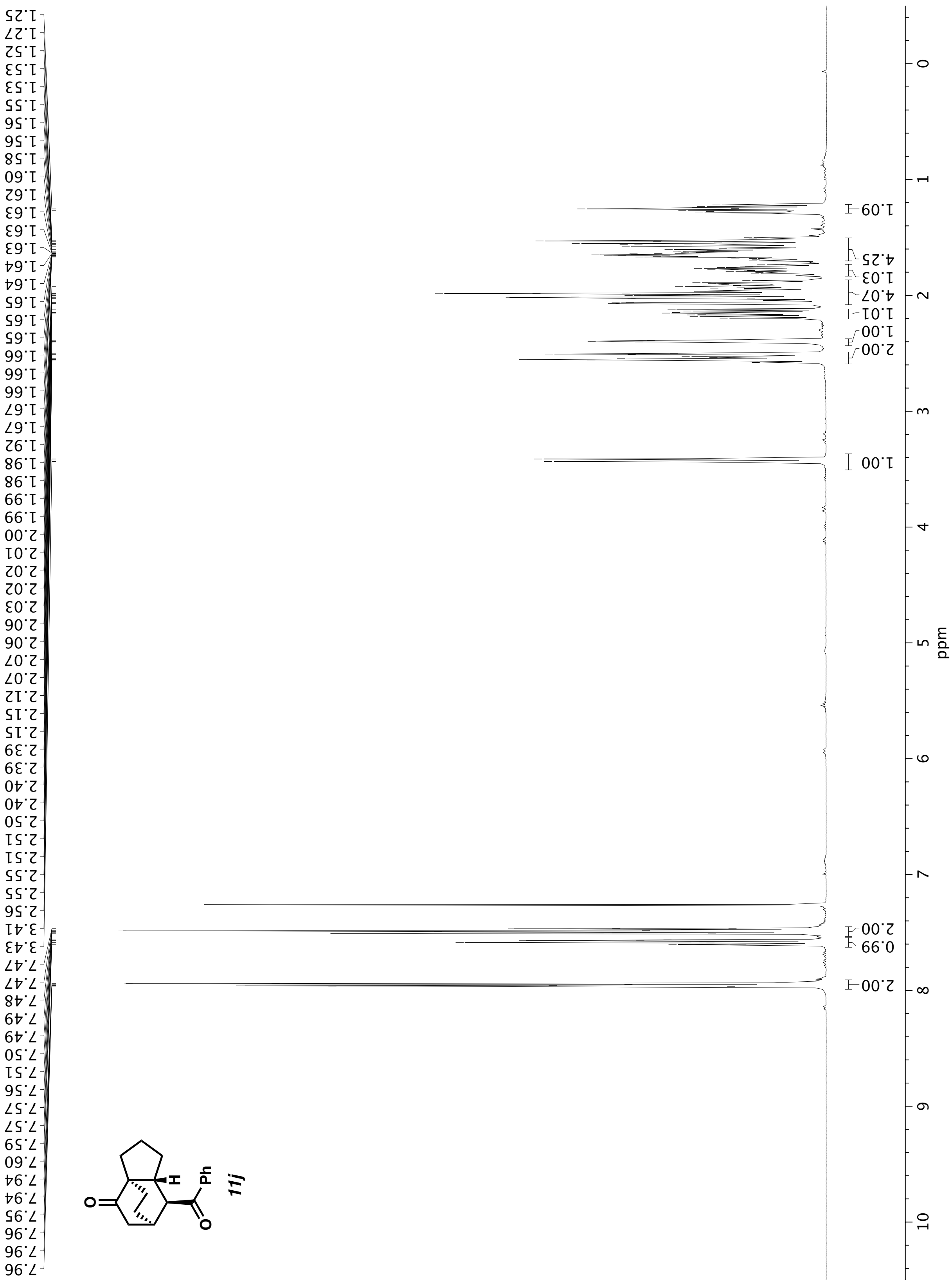

^{1}H NMR (400 MHz, $CDCl_3$) of compound **11j**.

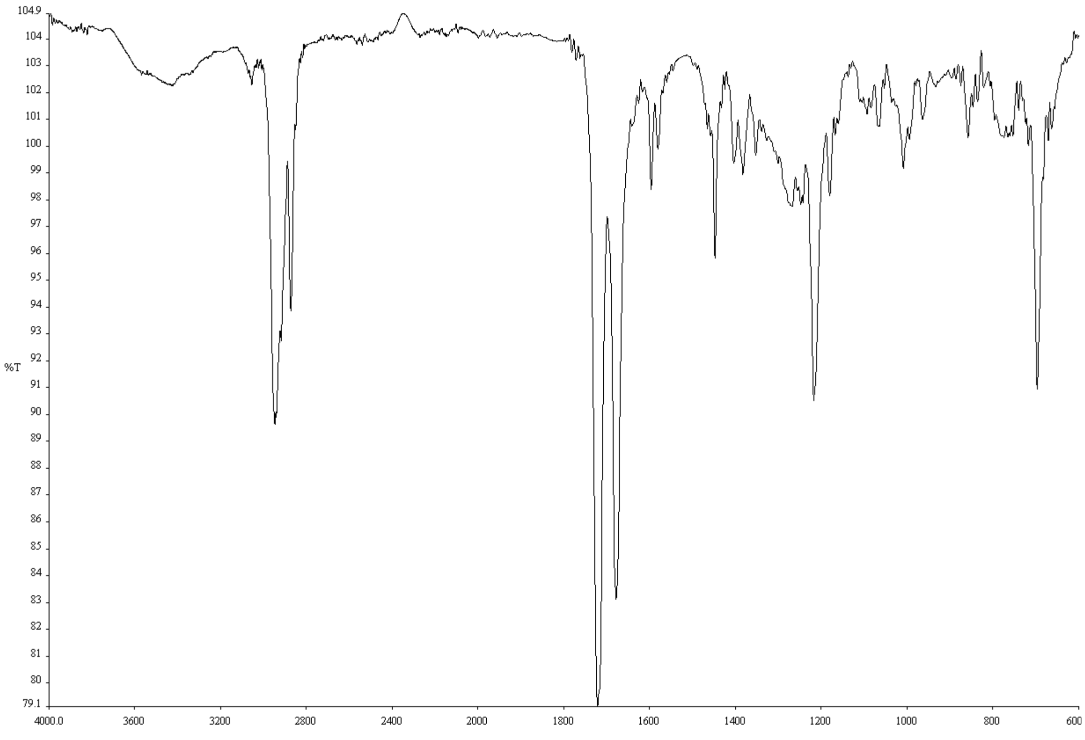

Infrared spectrum (Thin Film, NaCl) of compound **11j**.

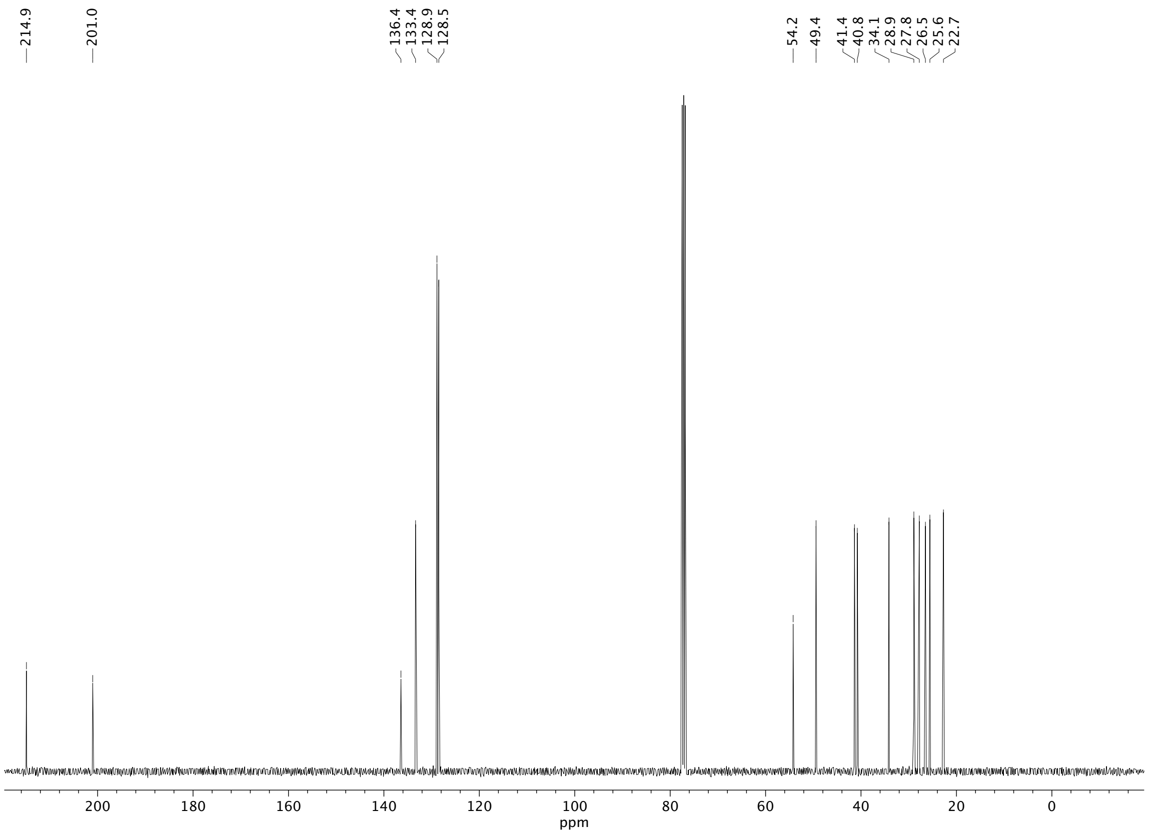

^{13}C NMR (100 MHz, $CDCl_3$) of compound **11j**.

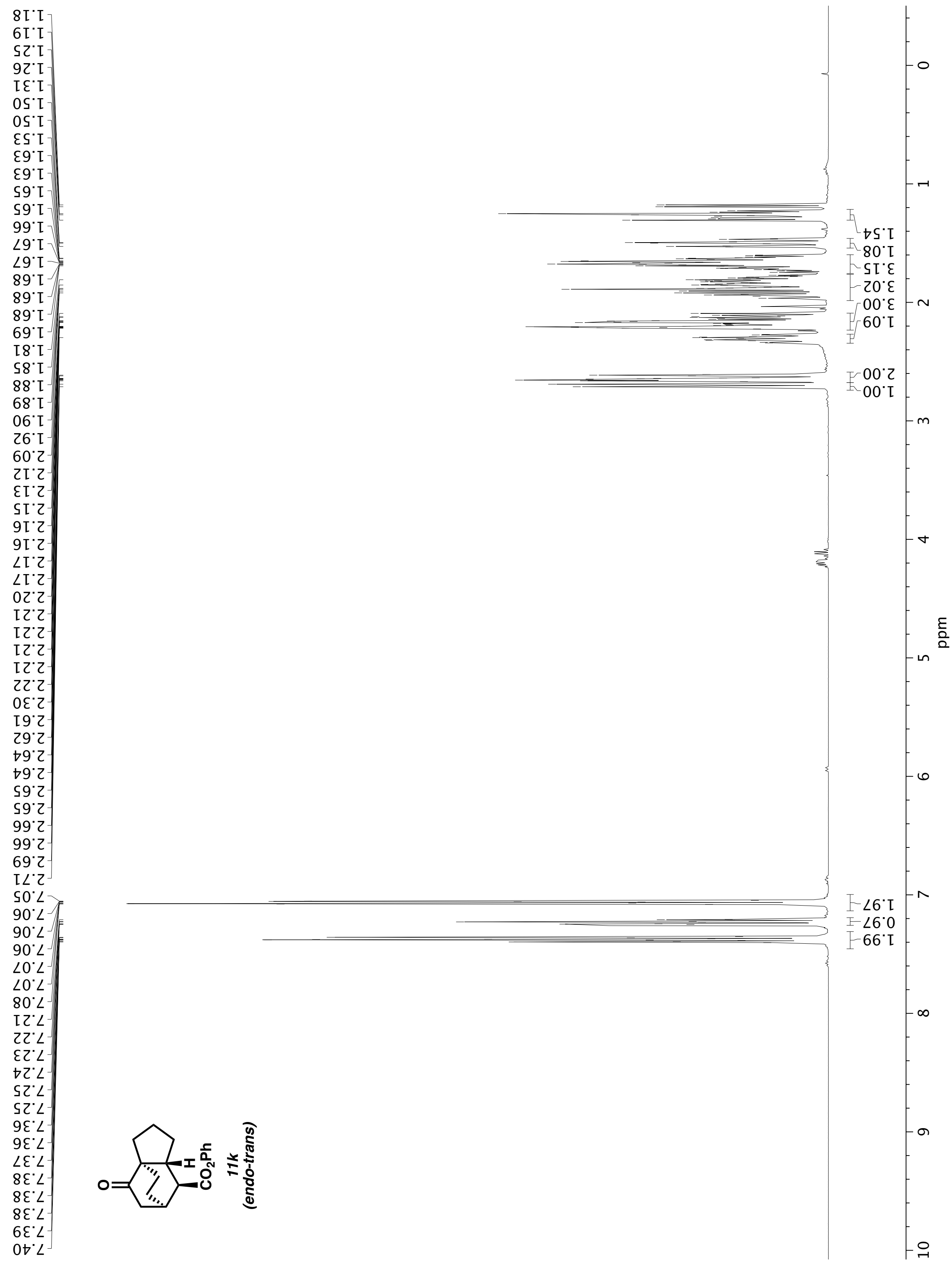

^{1}H NMR (400 MHz, $CDCl_3$) of compound **11k**.

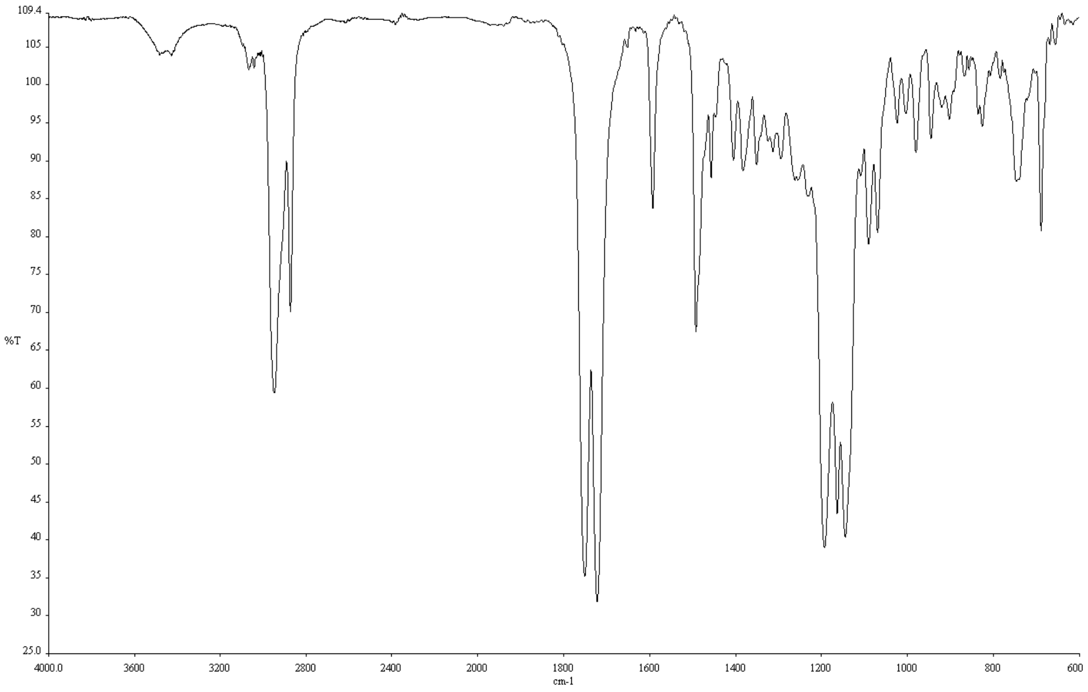

Infrared spectrum (Thin Film, NaCl) of compound **11k**.

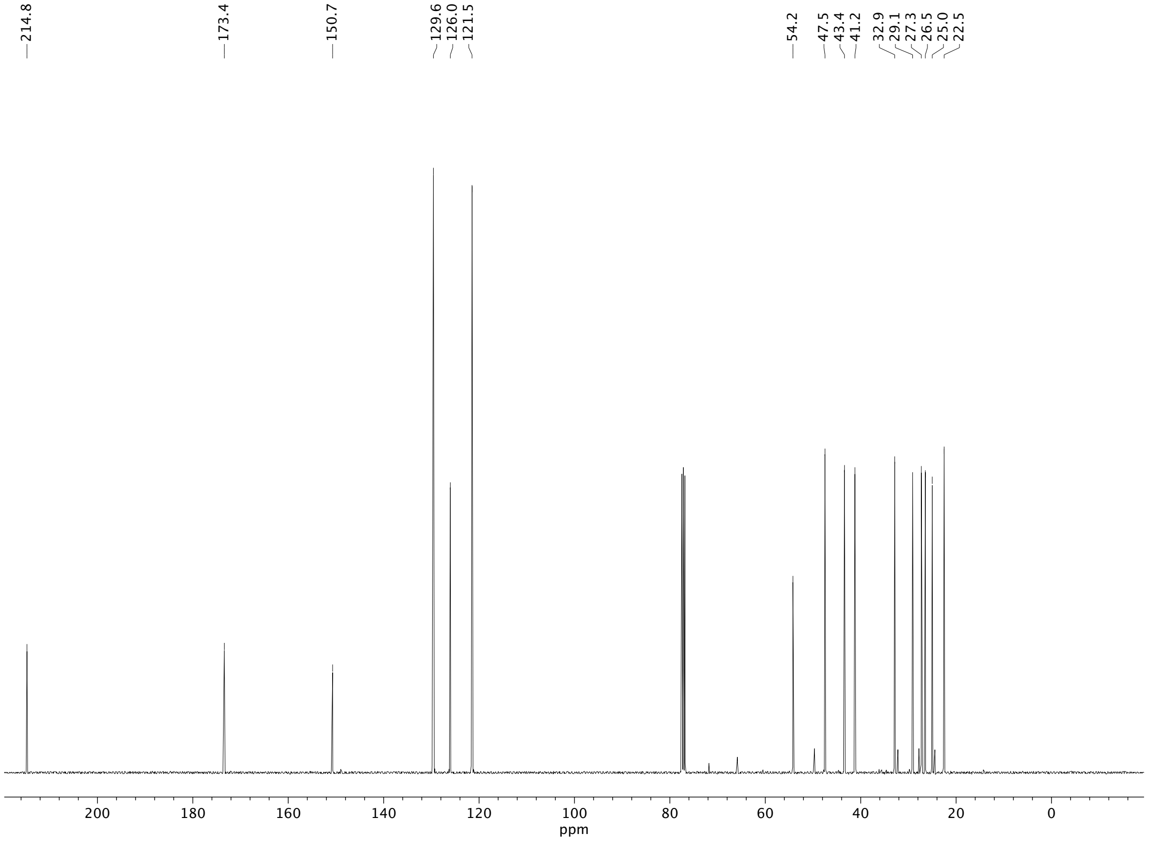

^{13}C NMR (100 MHz, $CDCl_3$) of compound **11k**.

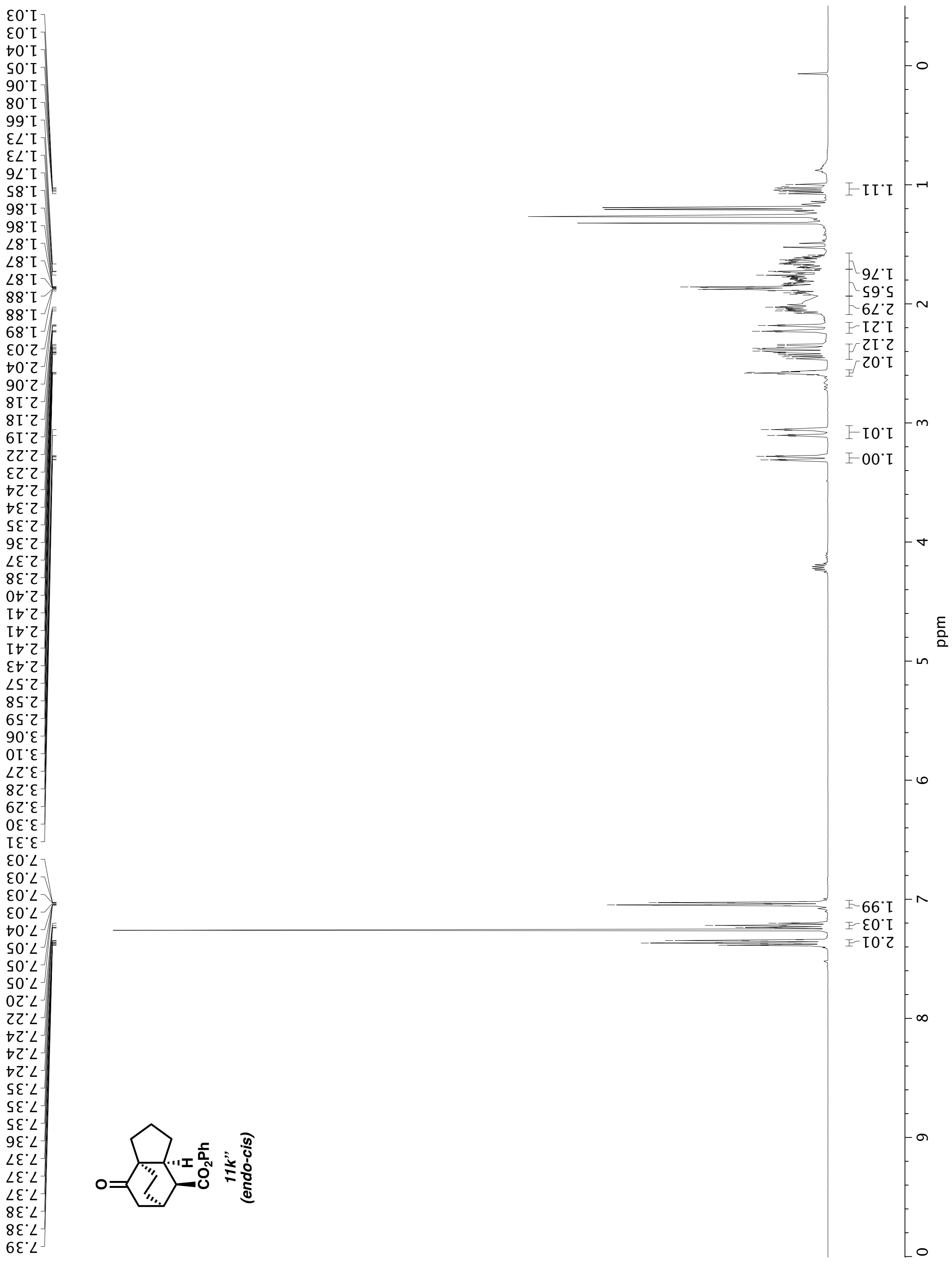

^{1}H NMR (400 MHz, $CDCl_3$) of compound **11k''** .

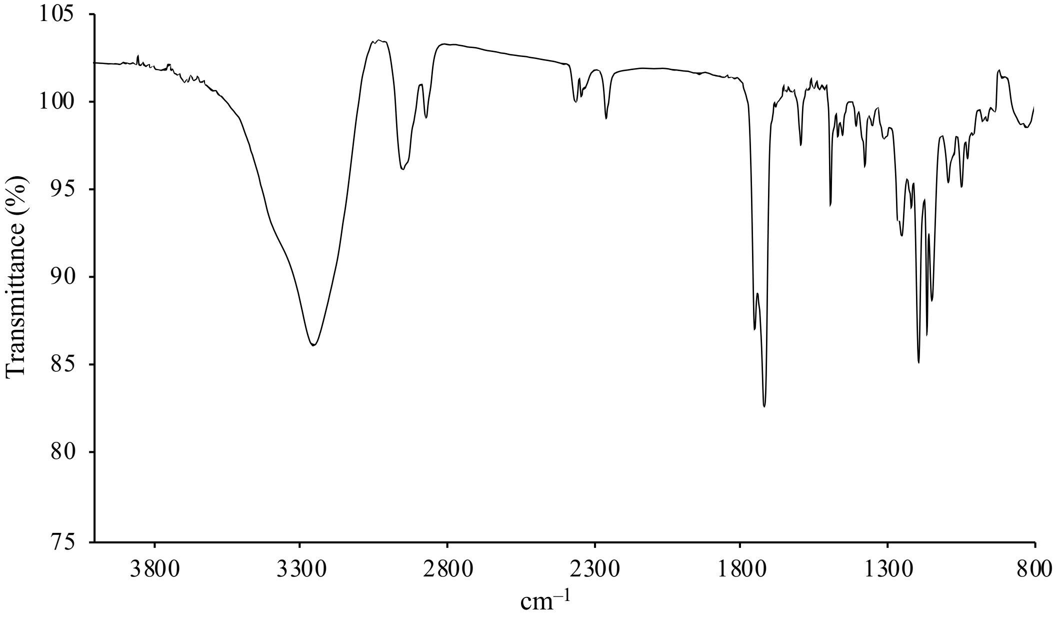

Infrared spectrum ($CDCl_3$ solution) of compound **11k”**.

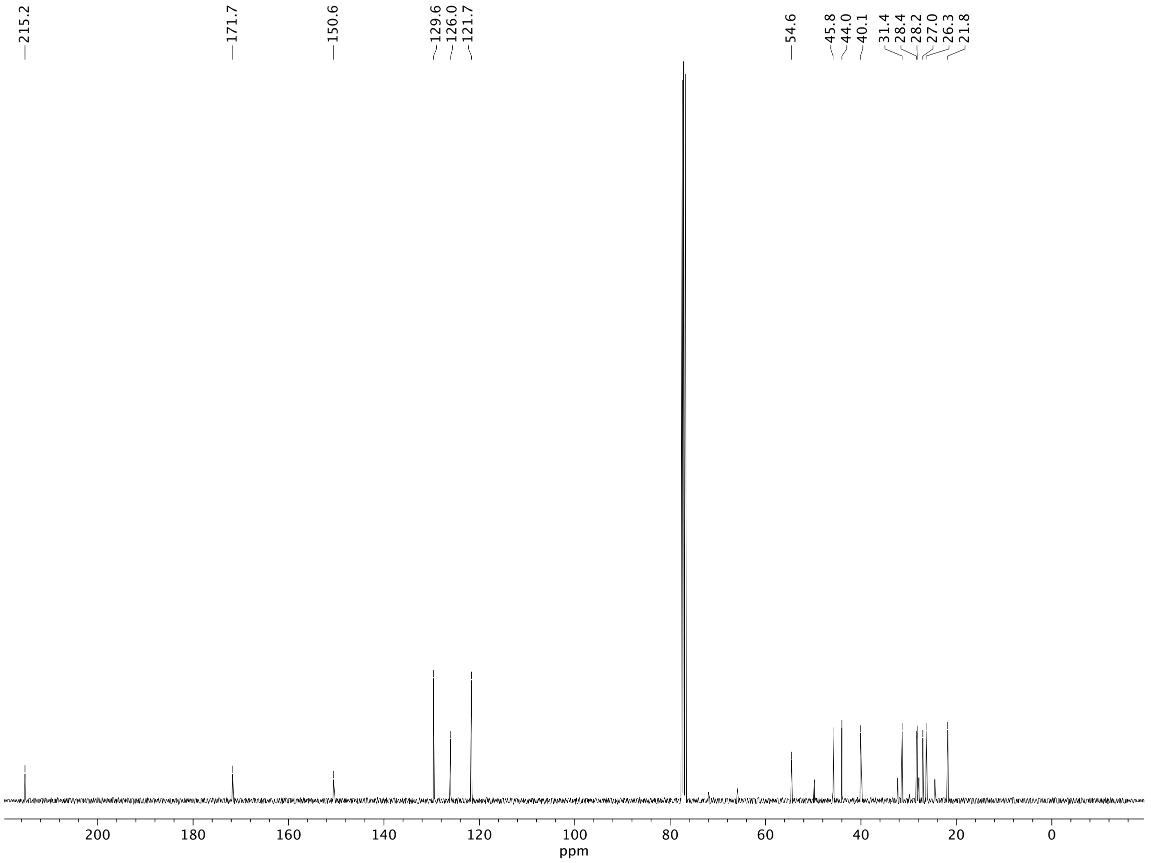

^{13}C NMR (100 MHz, $CDCl_3$) of compound **11k”**.

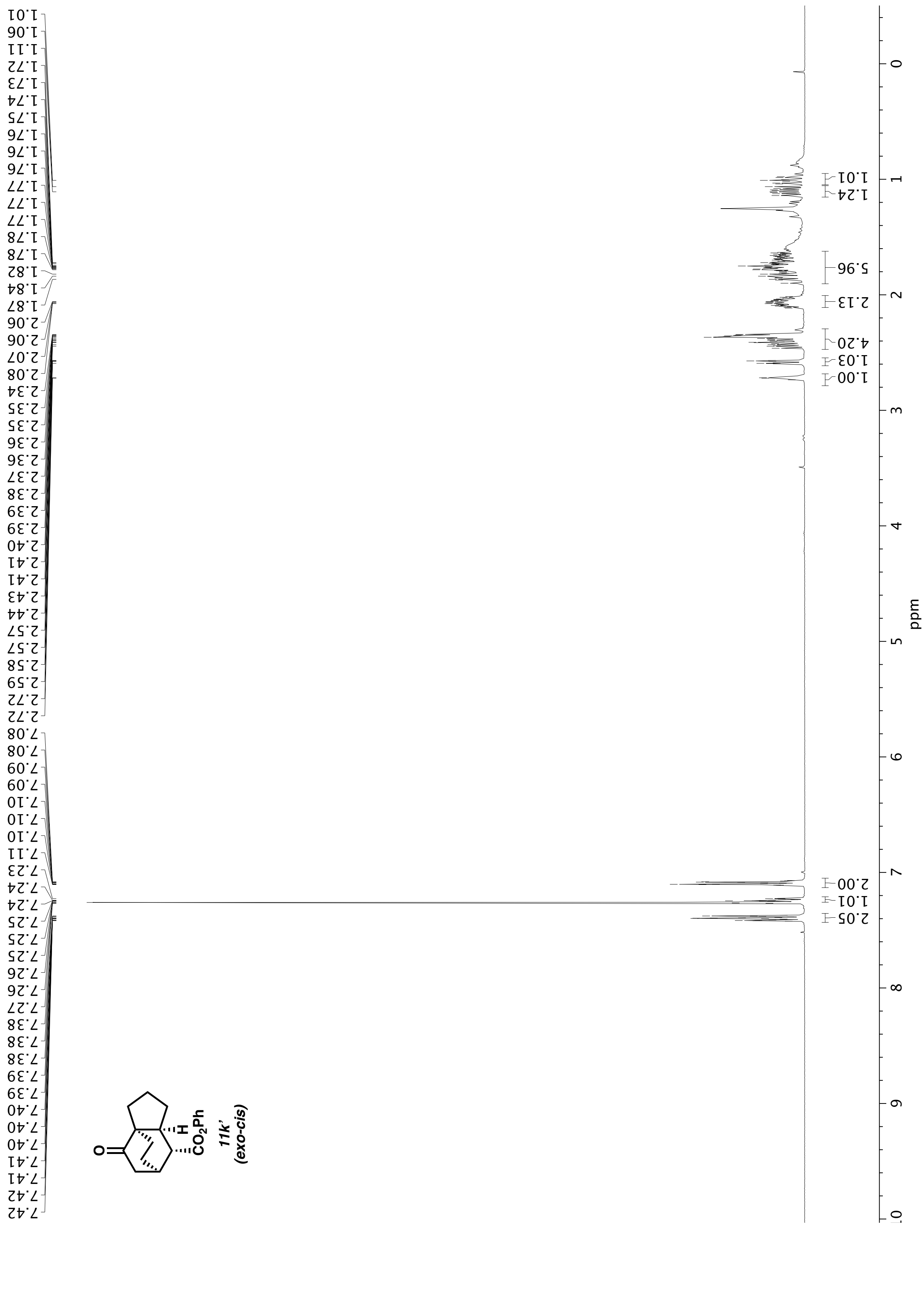

^1H NMR (400 MHz, $CDCl_3$) of compound **11k'**

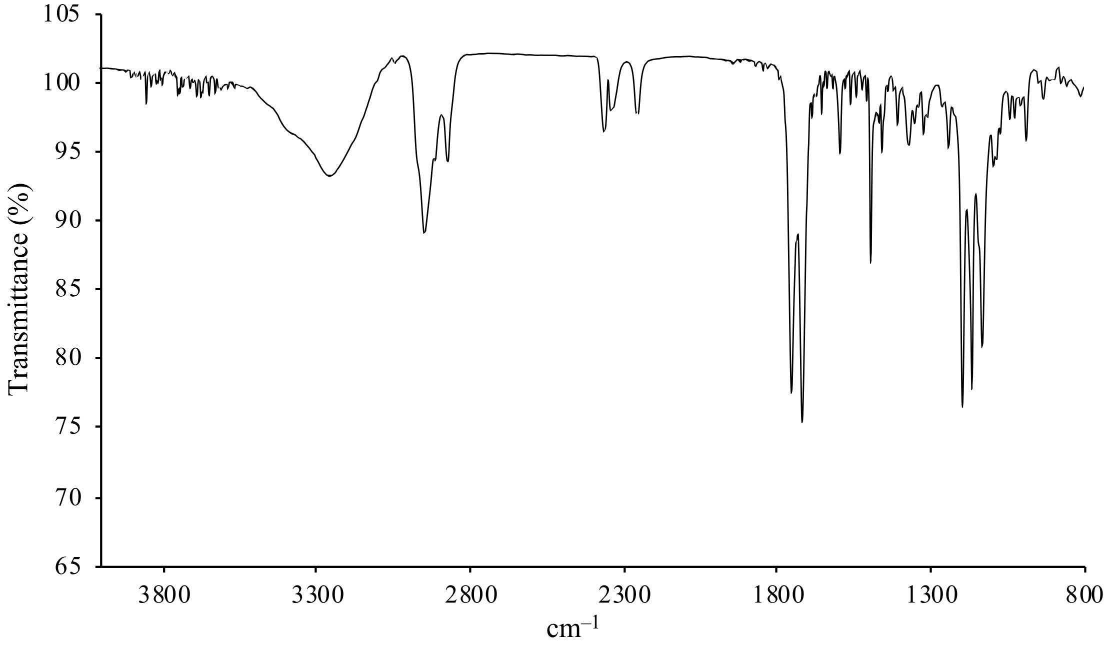

Infrared spectrum ($CDCl_3$ solution) of compound **11k'**.

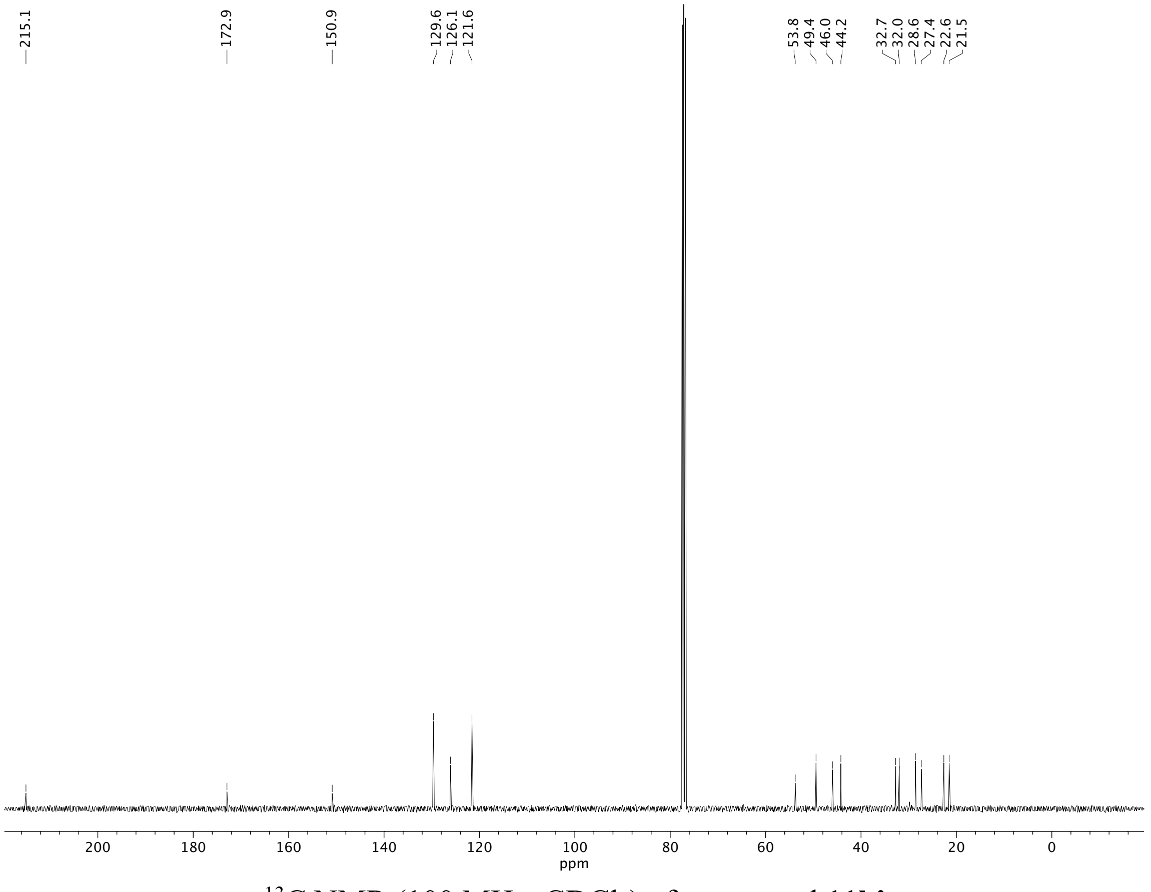

^{13}C NMR (100 MHz, $CDCl_3$) of compound **11k'**.

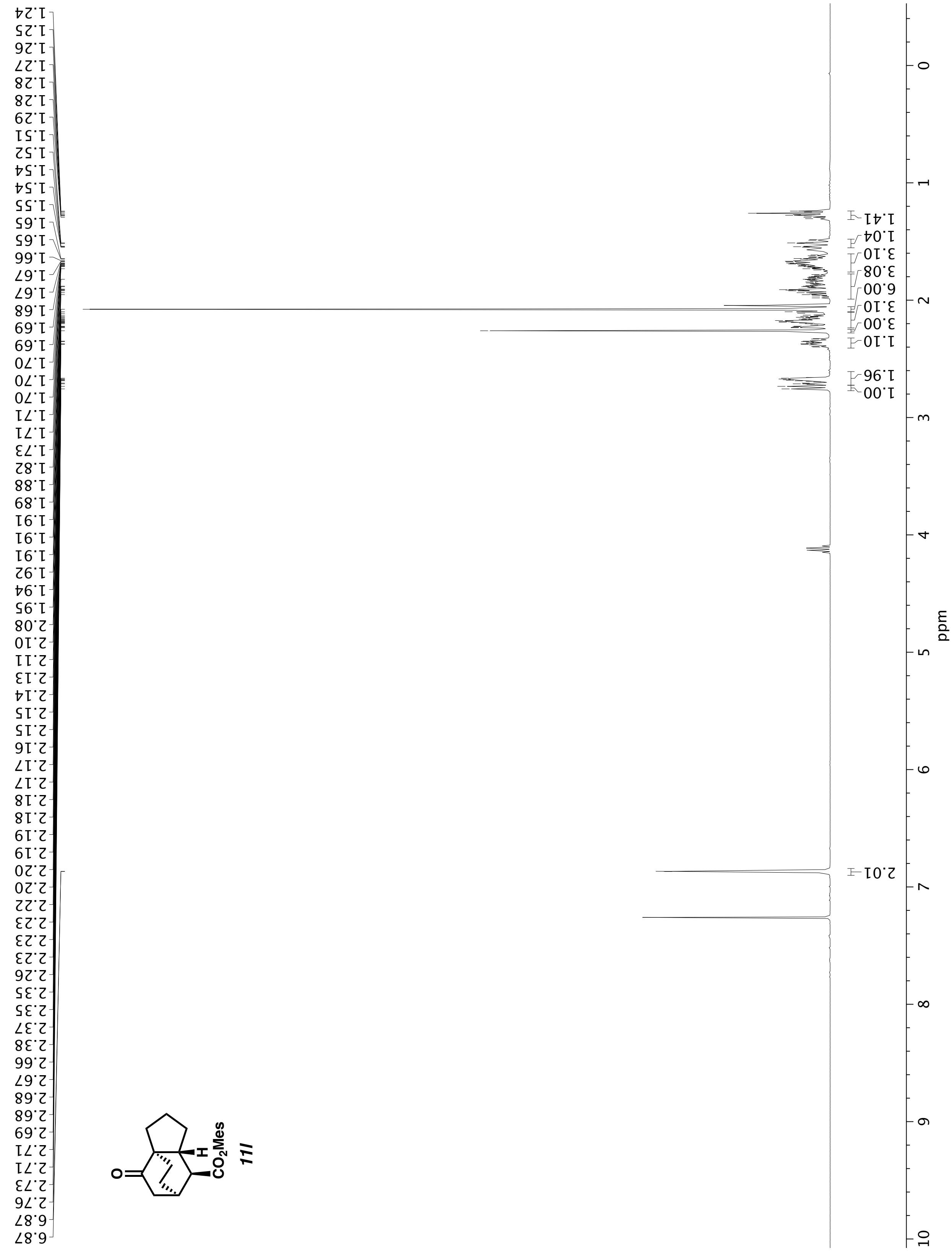

^1H NMR (400 MHz, $CDCl_3$) of compound **11l.**

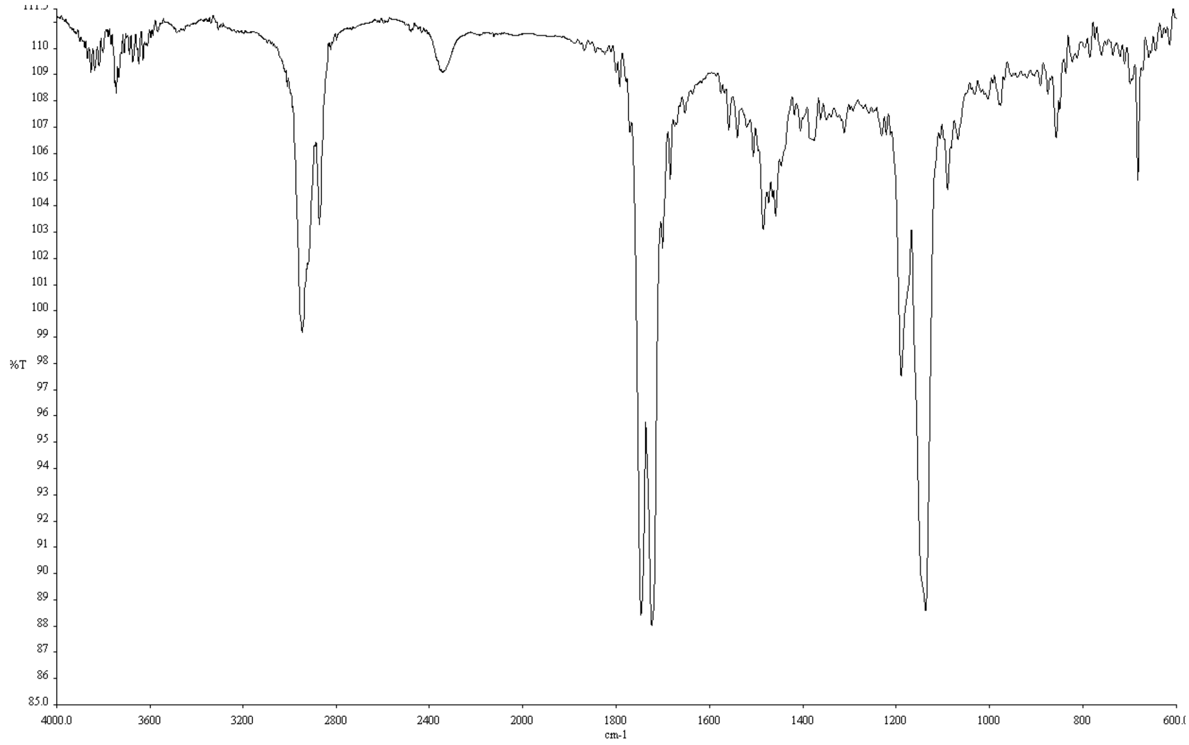

Infrared spectrum (Thin Film, NaCl) of compound **11l**.

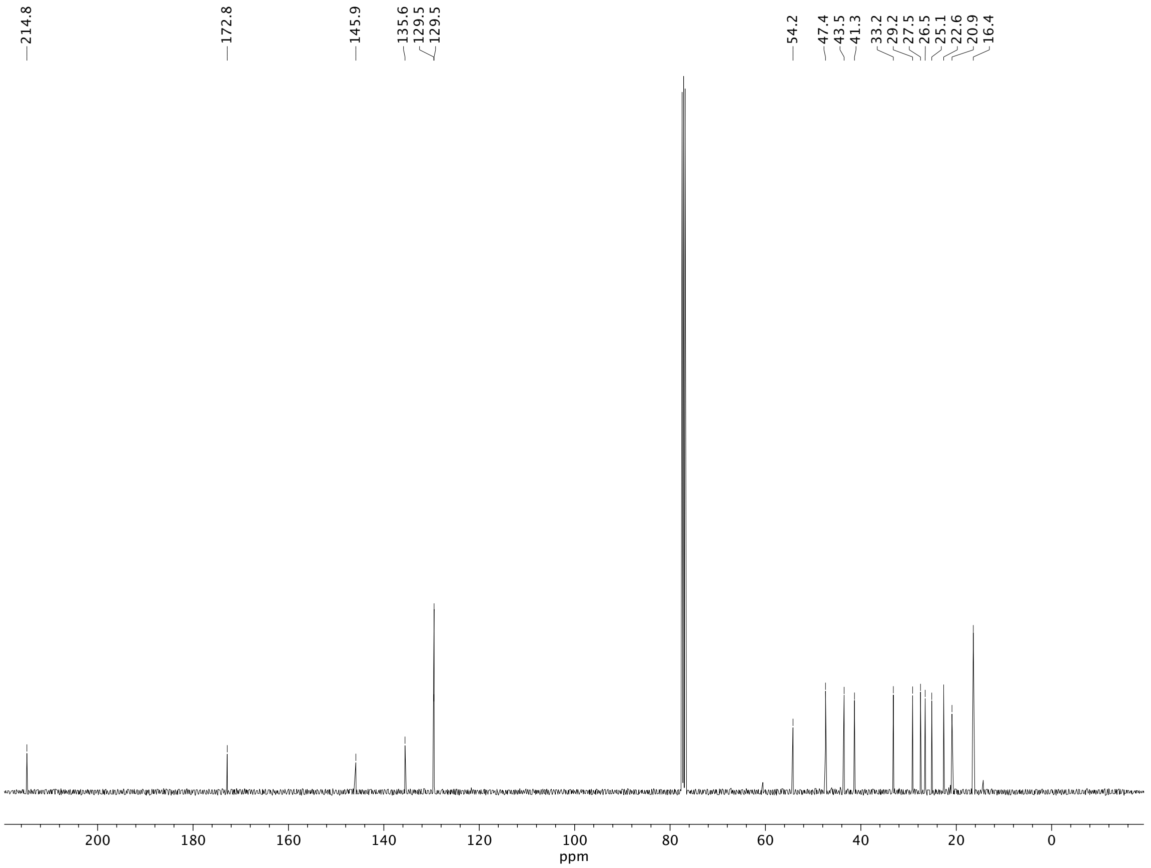

^{13}C NMR (100 MHz, $CDCl_3$) of compound **11l**.

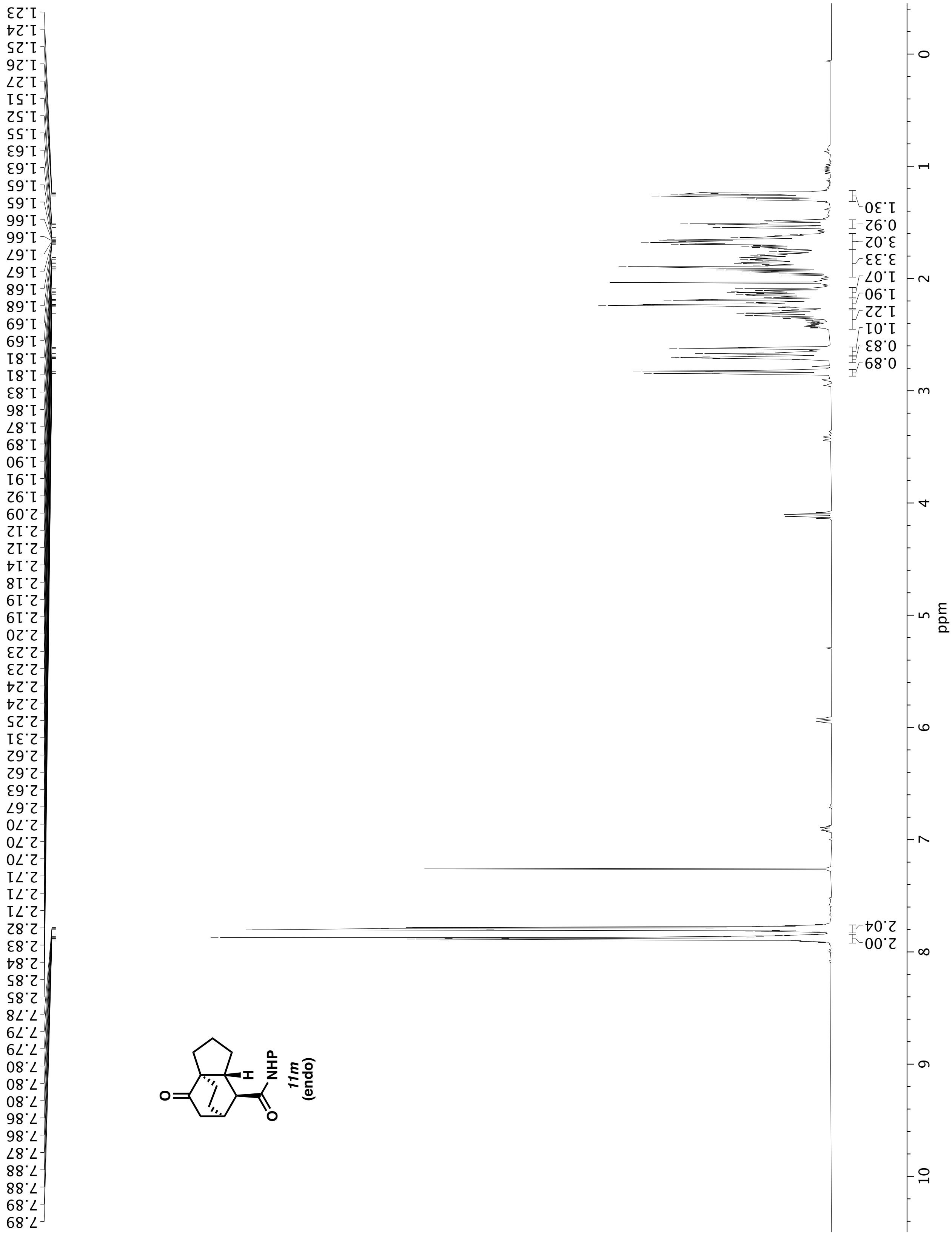

^{1}H NMR (400 MHz, $CDCl_3$) of compound **11m**.

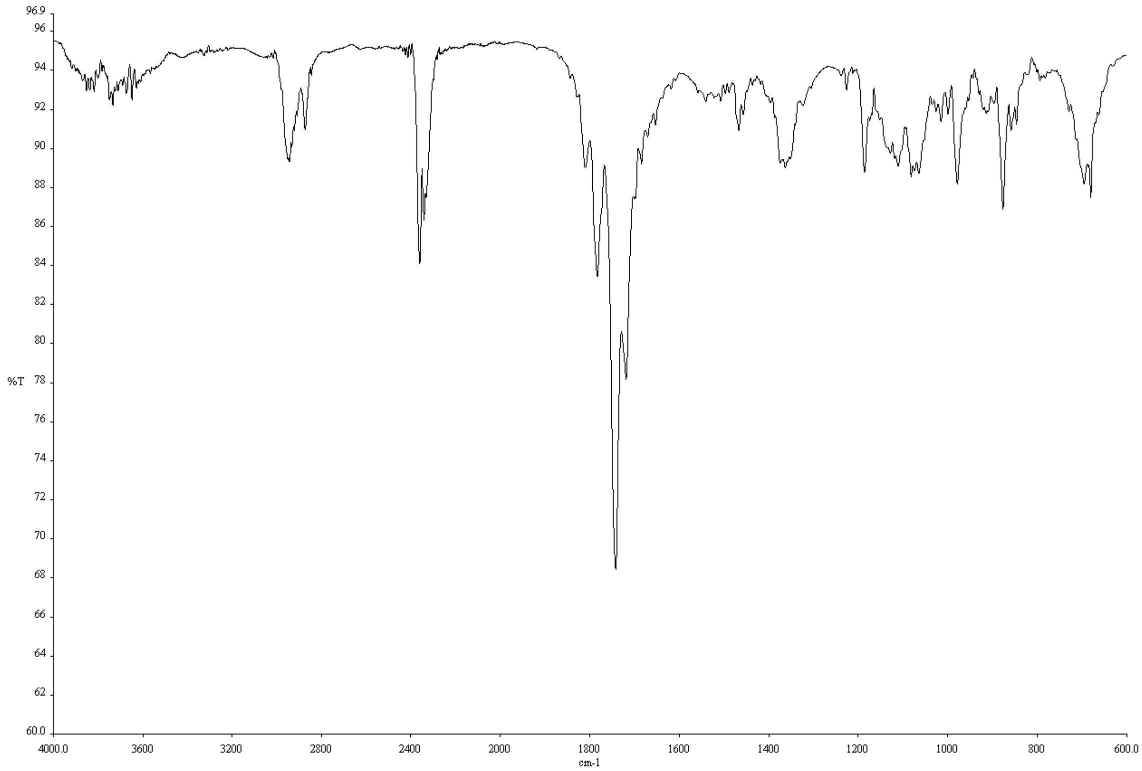

Infrared spectrum (Thin Film, NaCl) of compound **11m**.

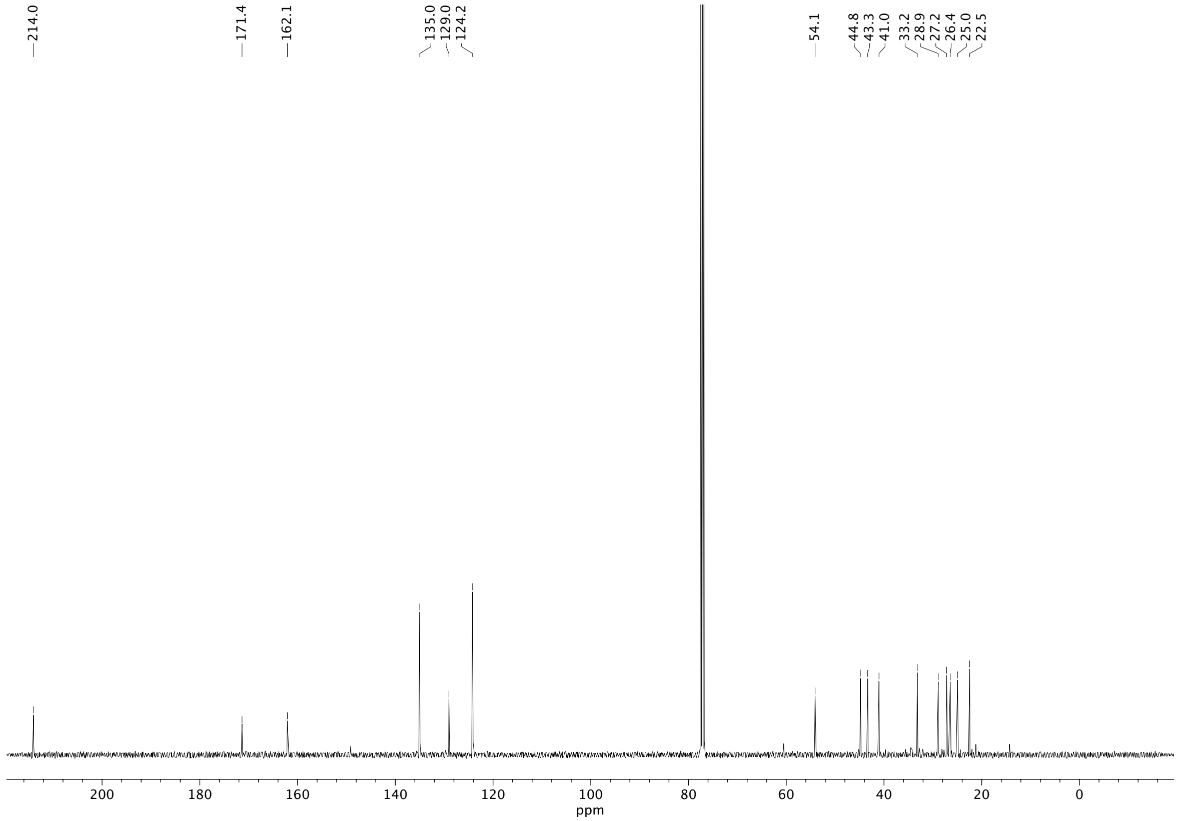

^{13}C NMR (100 MHz, $CDCl_3$) of compound **11m**.

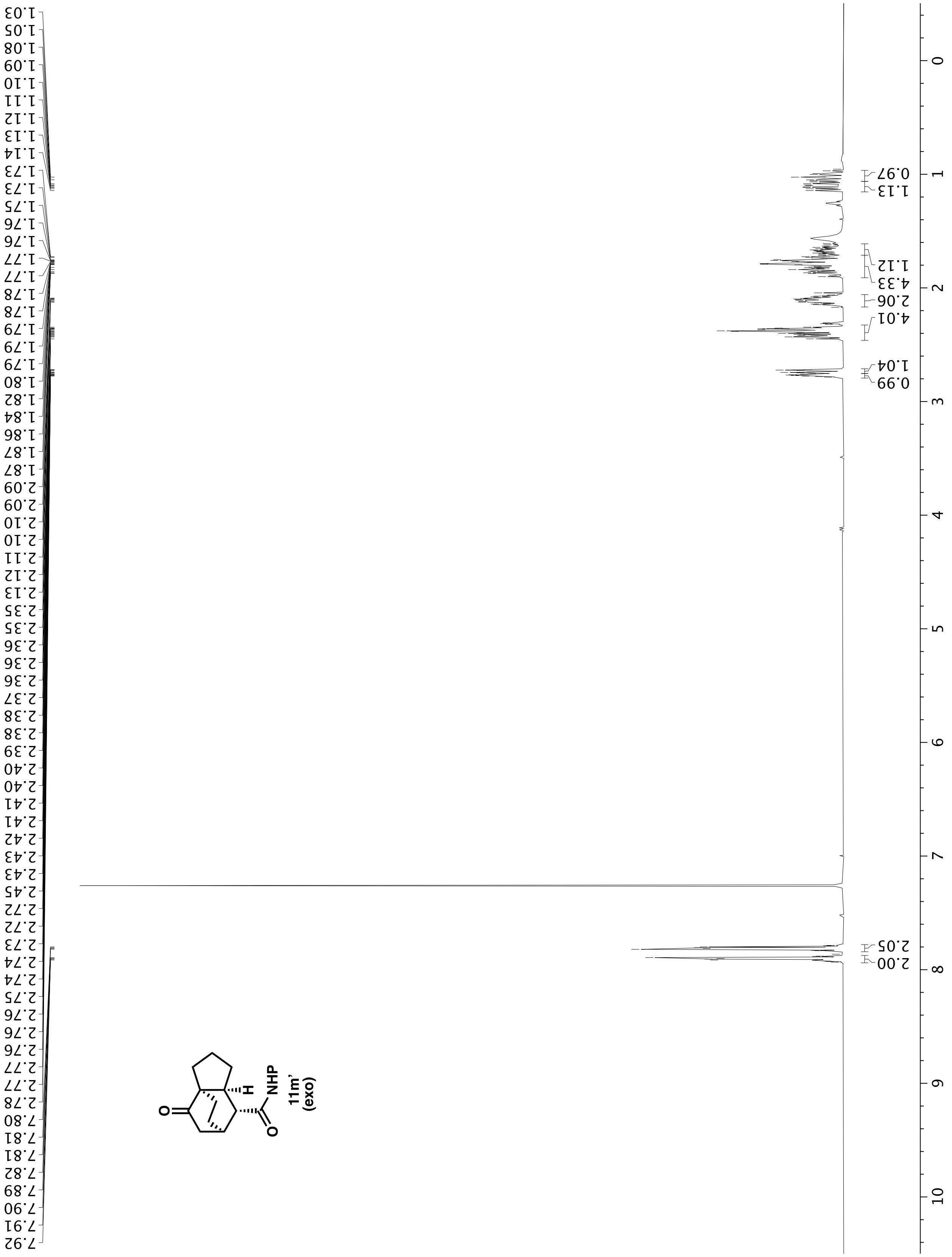

^{1}H NMR (400 MHz, $CDCl_3$) of compound **11m'**

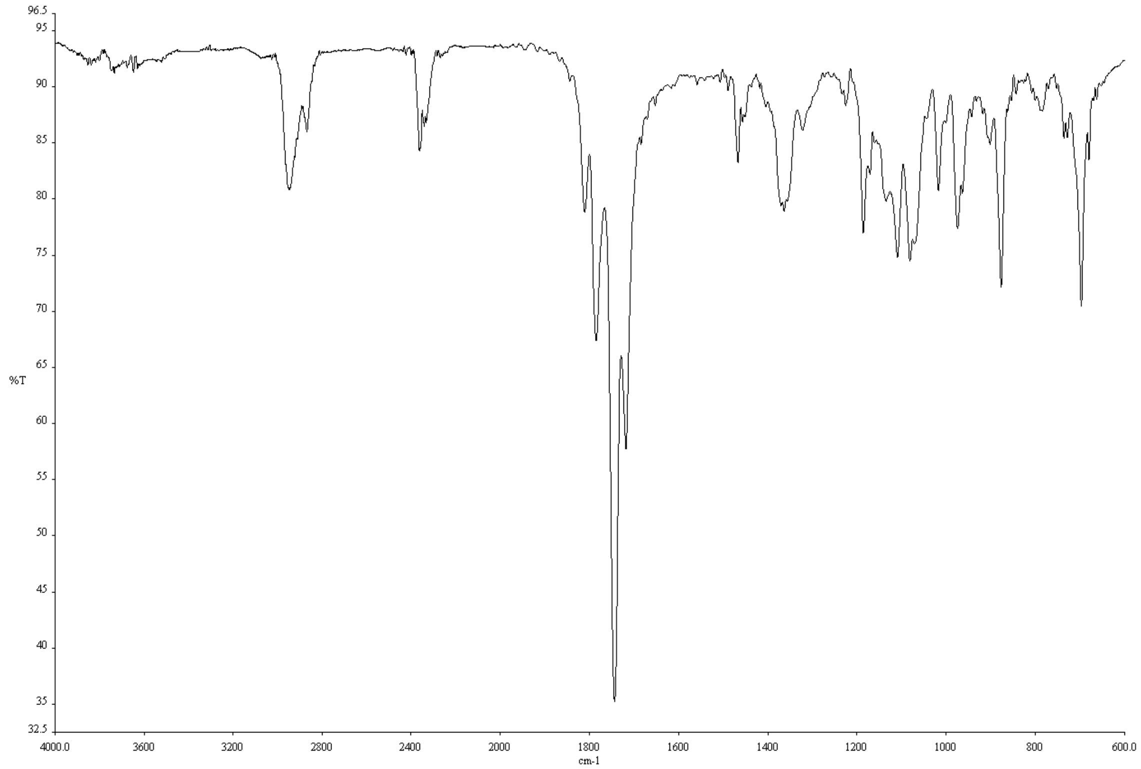

Infrared spectrum (Thin Film, NaCl) of compound **11m’**.

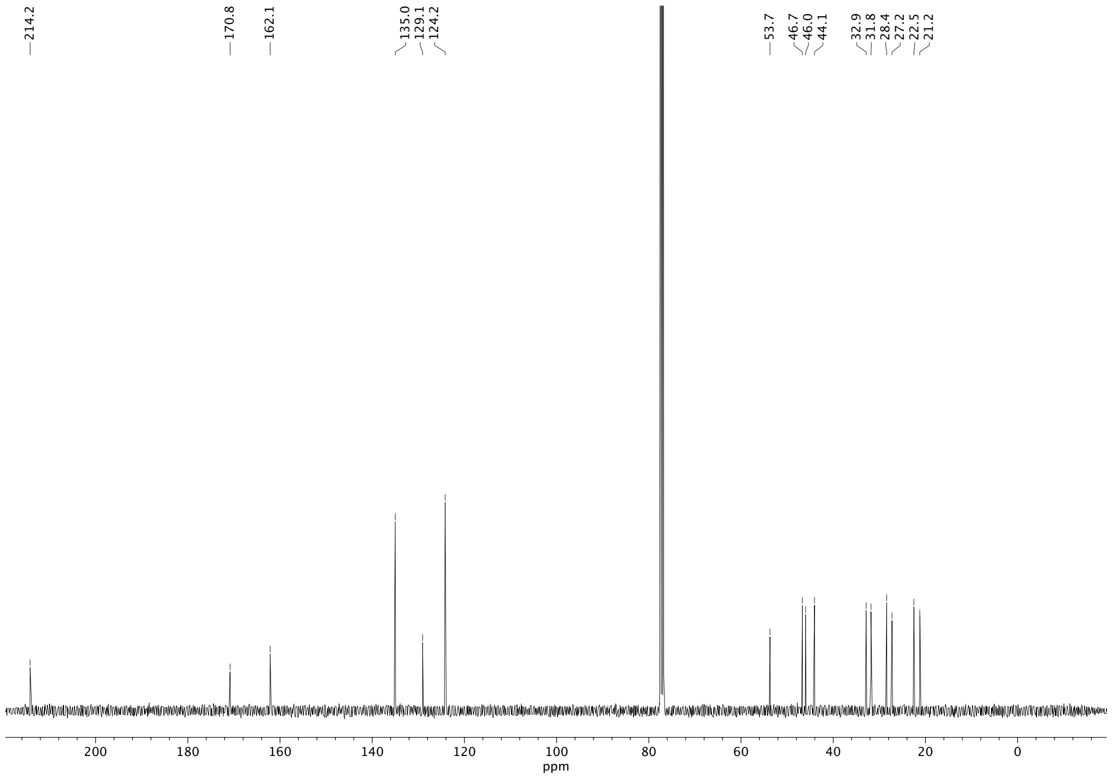

^{13}C NMR (100 MHz, $CDCl_3$) of compound **11m’**.

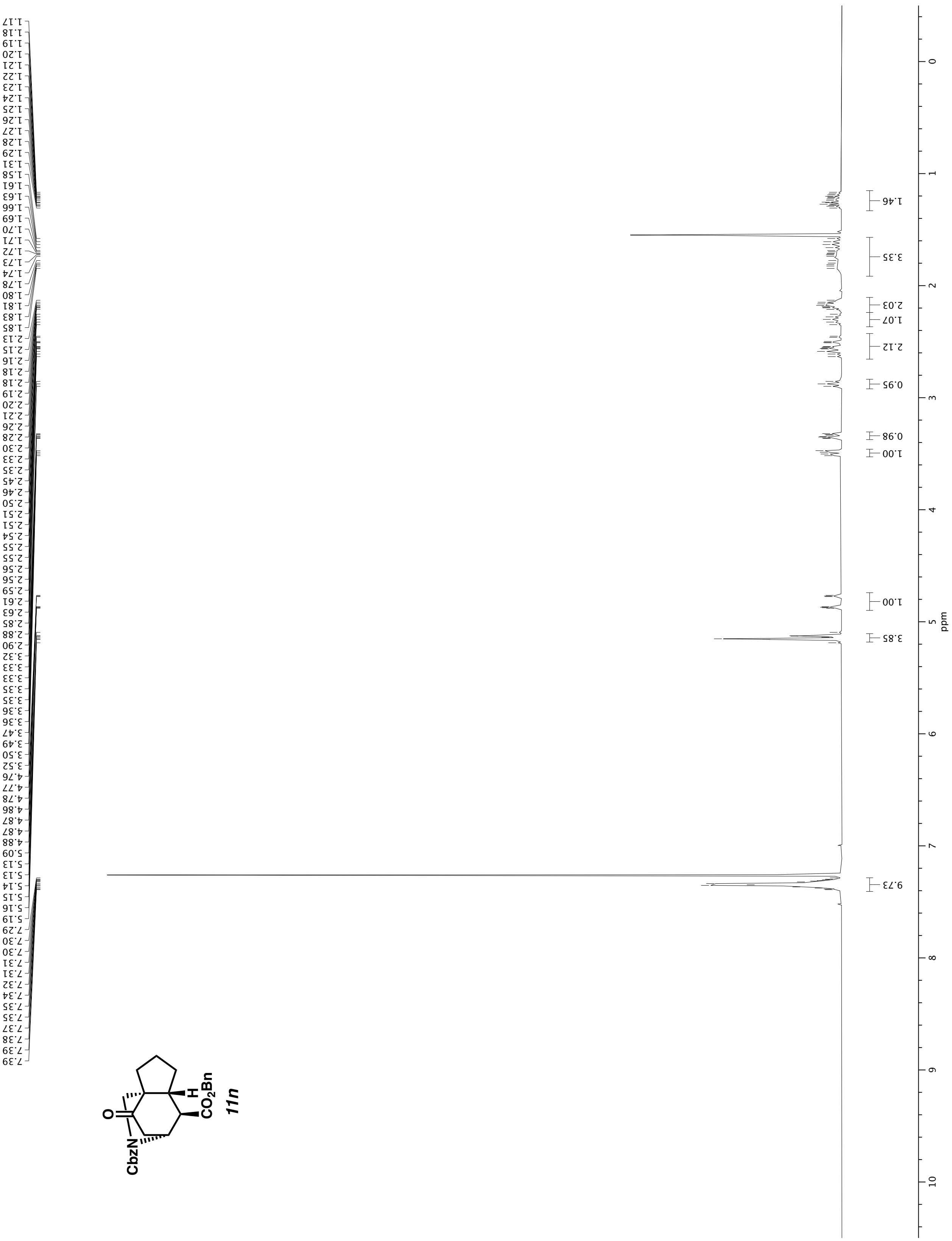

^{1}H NMR (400 MHz, $CDCl_3$) of compound **11n**.

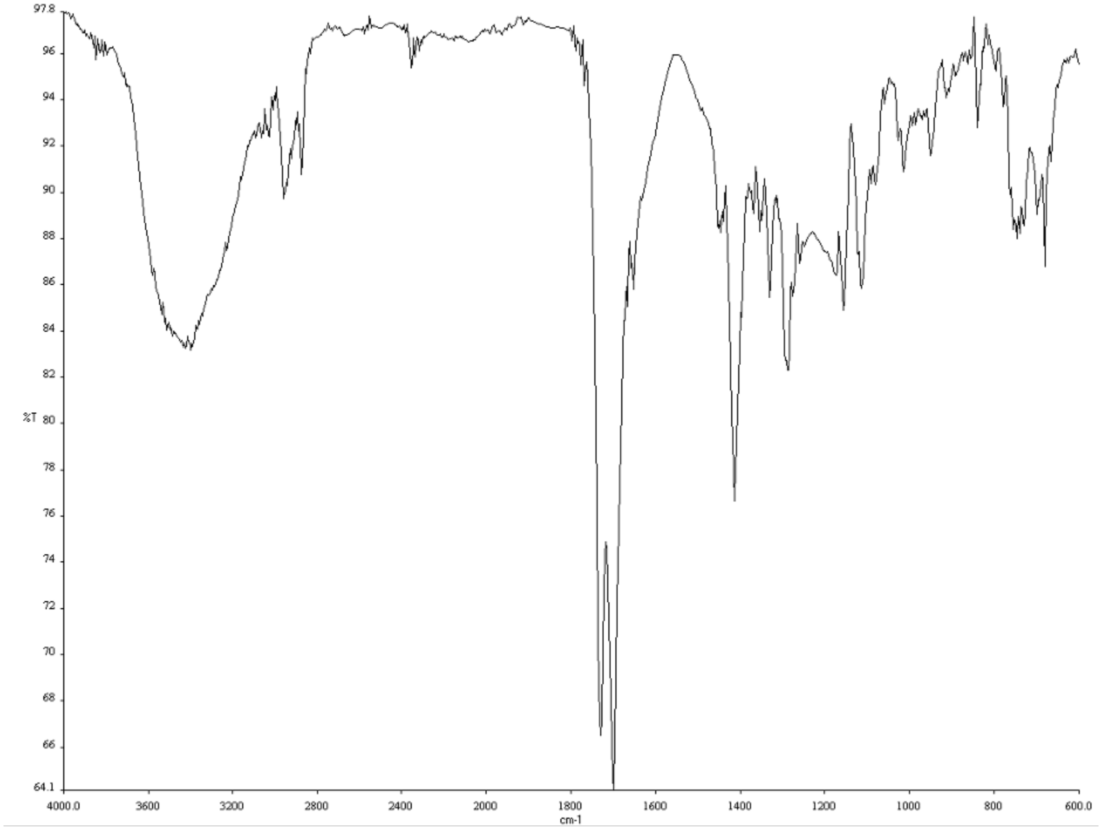

Infrared spectrum (Thin Film, NaCl) of compound **11n**.

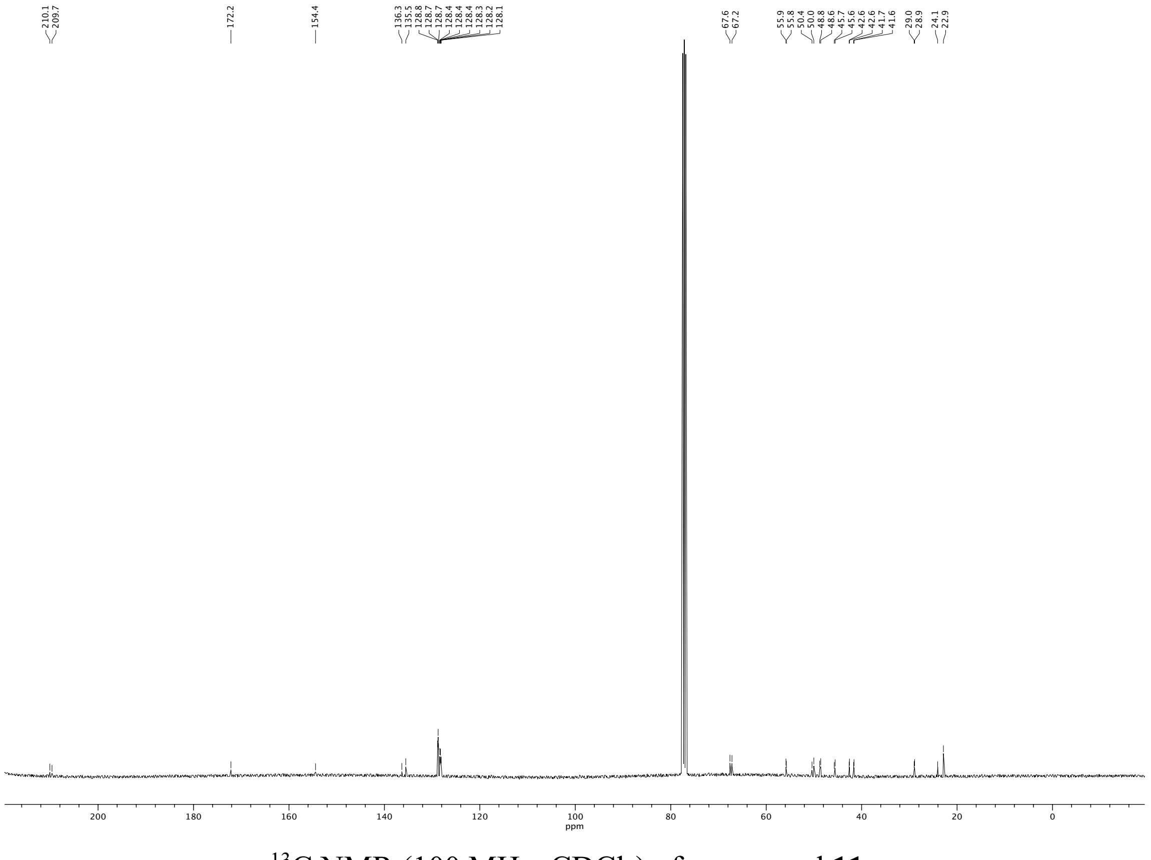

^{13}C NMR (100 MHz, $CDCl_3$) of compound **11n**.

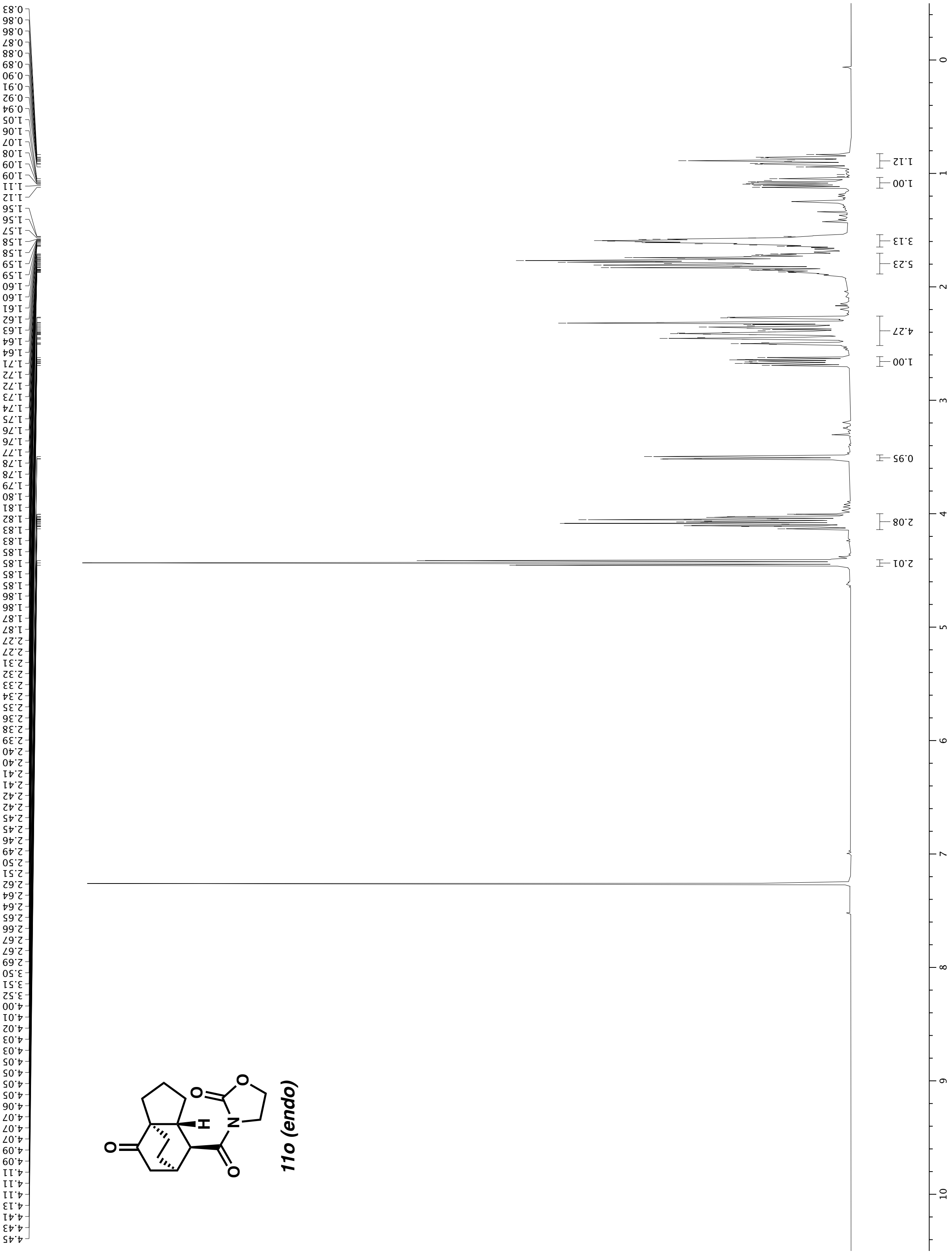

^{1}H NMR (400 MHz, $CDCl_3$) of compound **11o**.

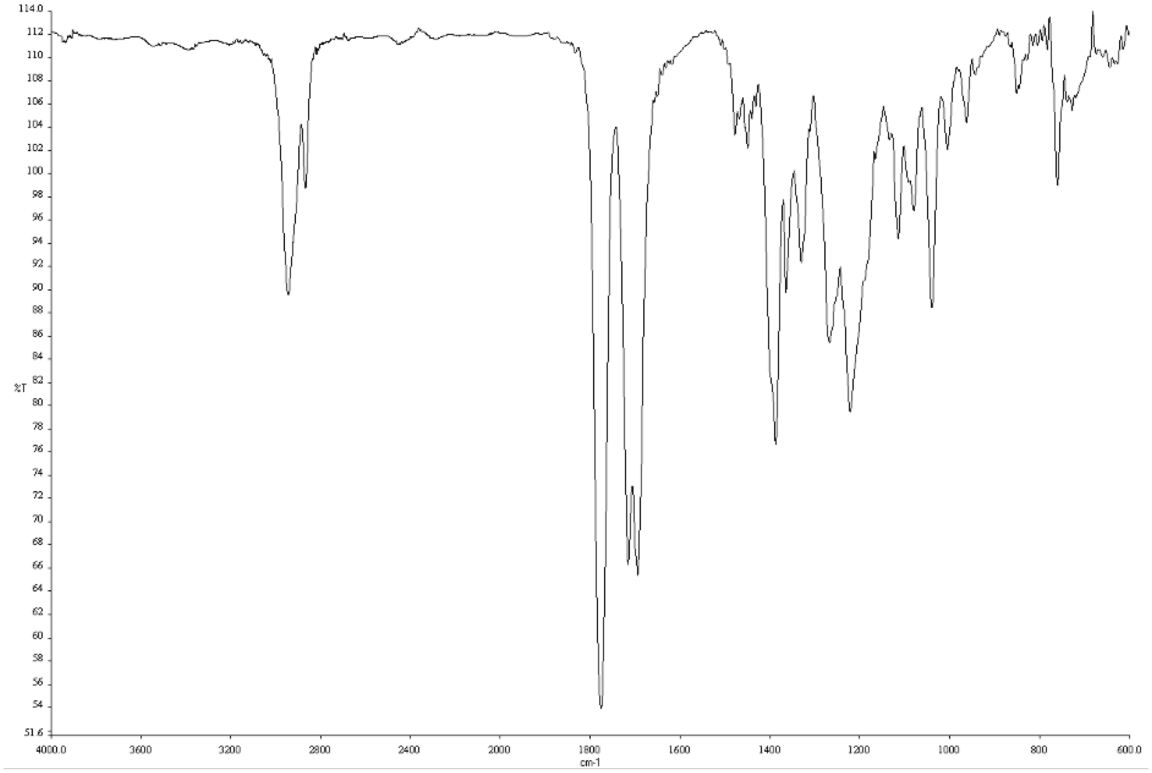

Infrared spectrum (Thin Film, NaCl) of compound **11o**.

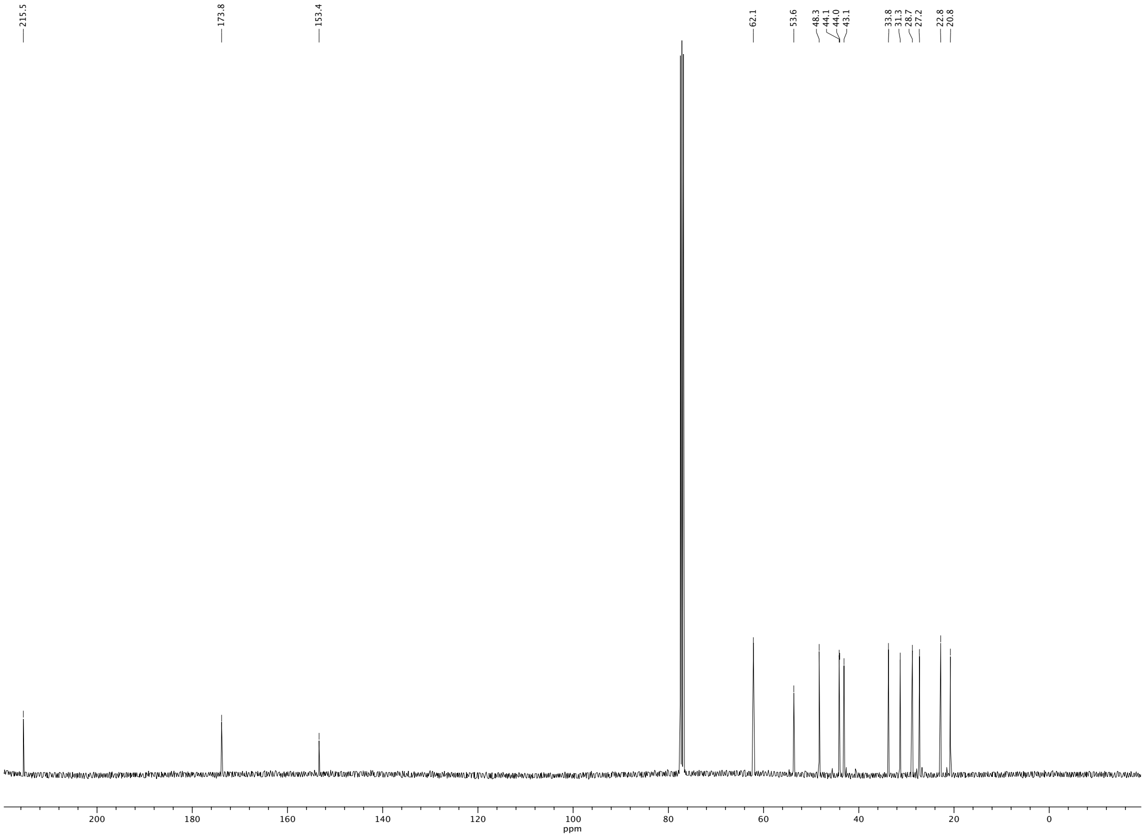

^{13}C NMR (100 MHz, $CDCl_3$) of compound **11o**.

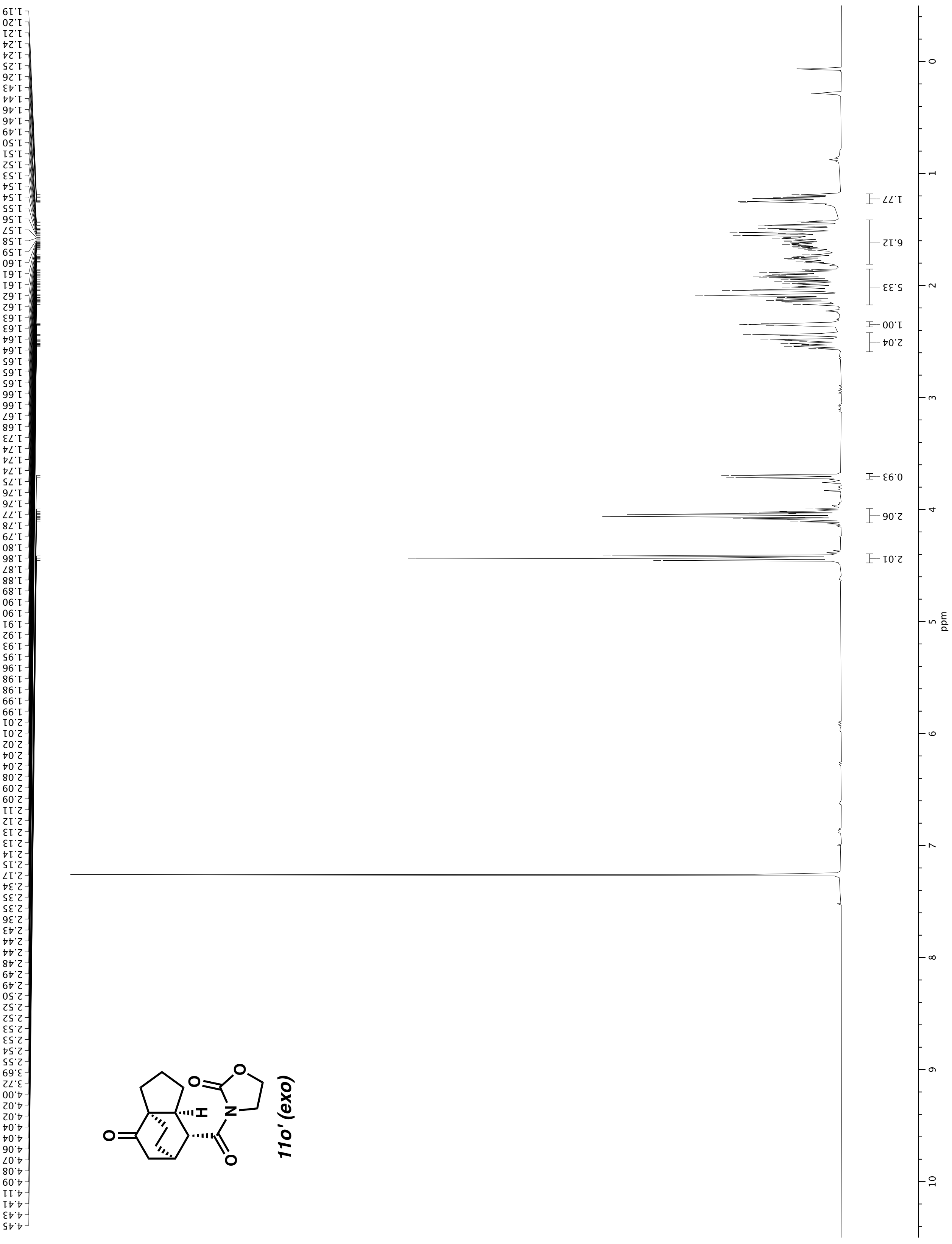

^{1}H NMR (400 MHz, $CDCl_3$) of compound **11o'**.

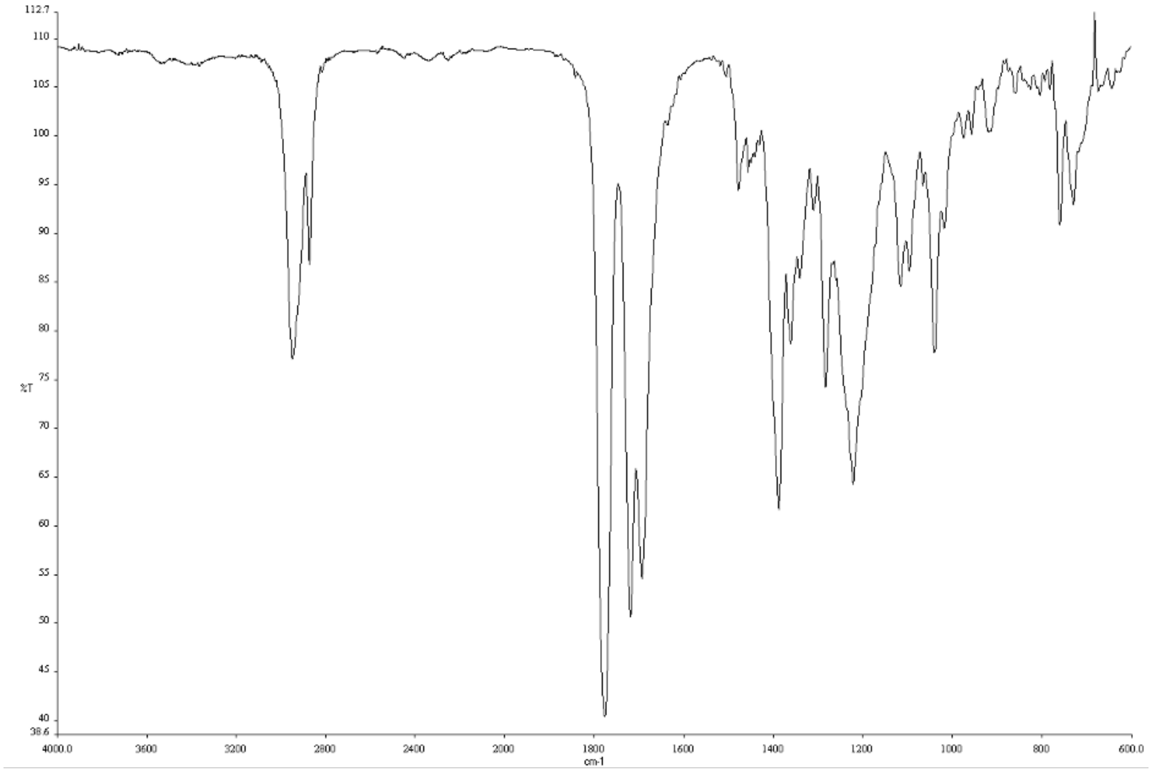

Infrared spectrum (Thin Film, NaCl) of compound **11o'**.

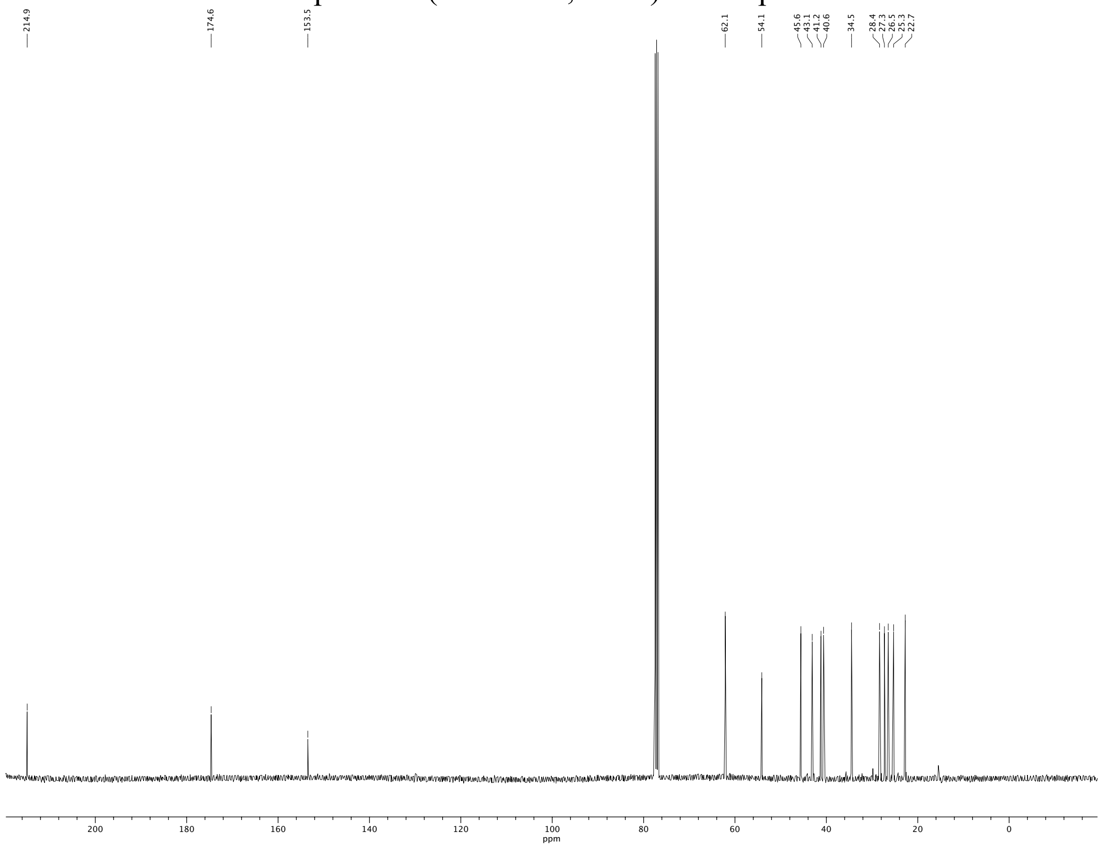

^{13}C NMR (100 MHz, $CDCl_3$) of compound **11o'**.

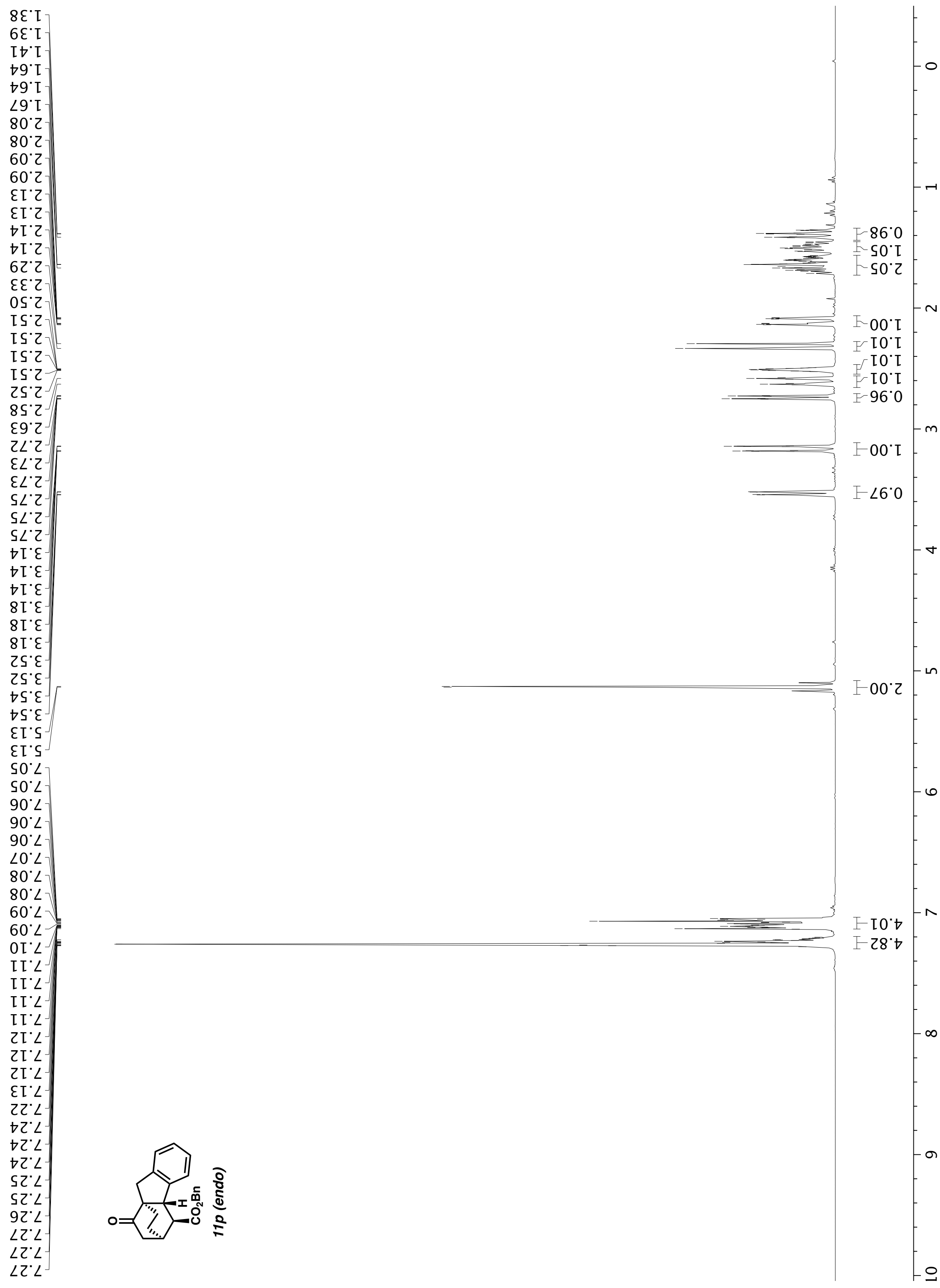

^{1}H NMR (400 MHz, $CDCl_3$) of compound **11p.**

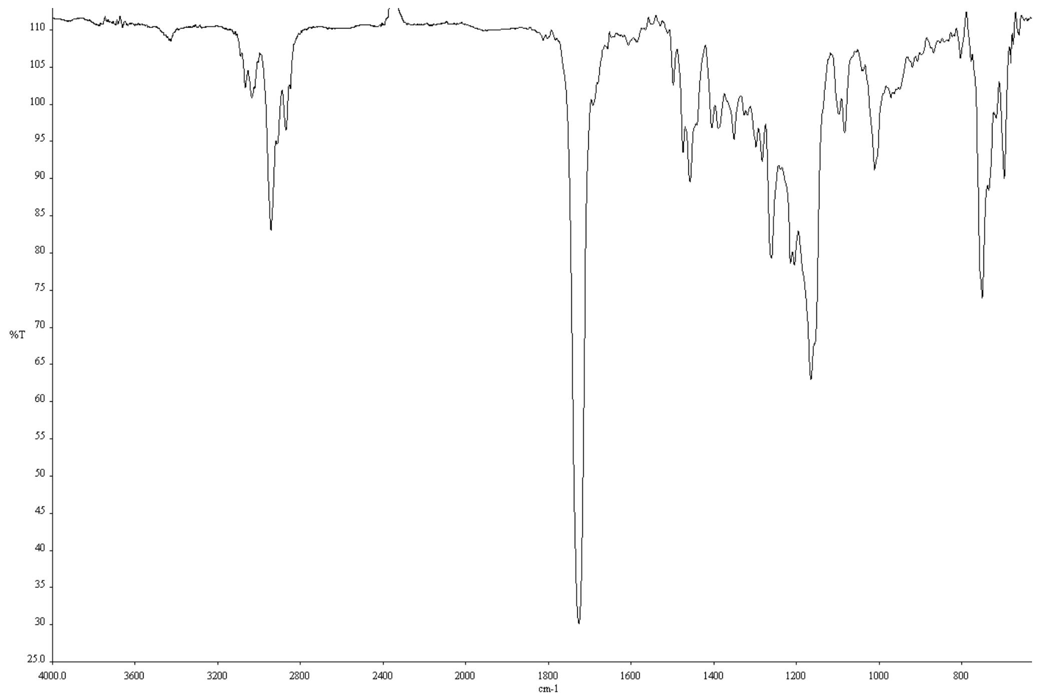

Infrared spectrum (Thin Film, NaCl) of compound **11p**.

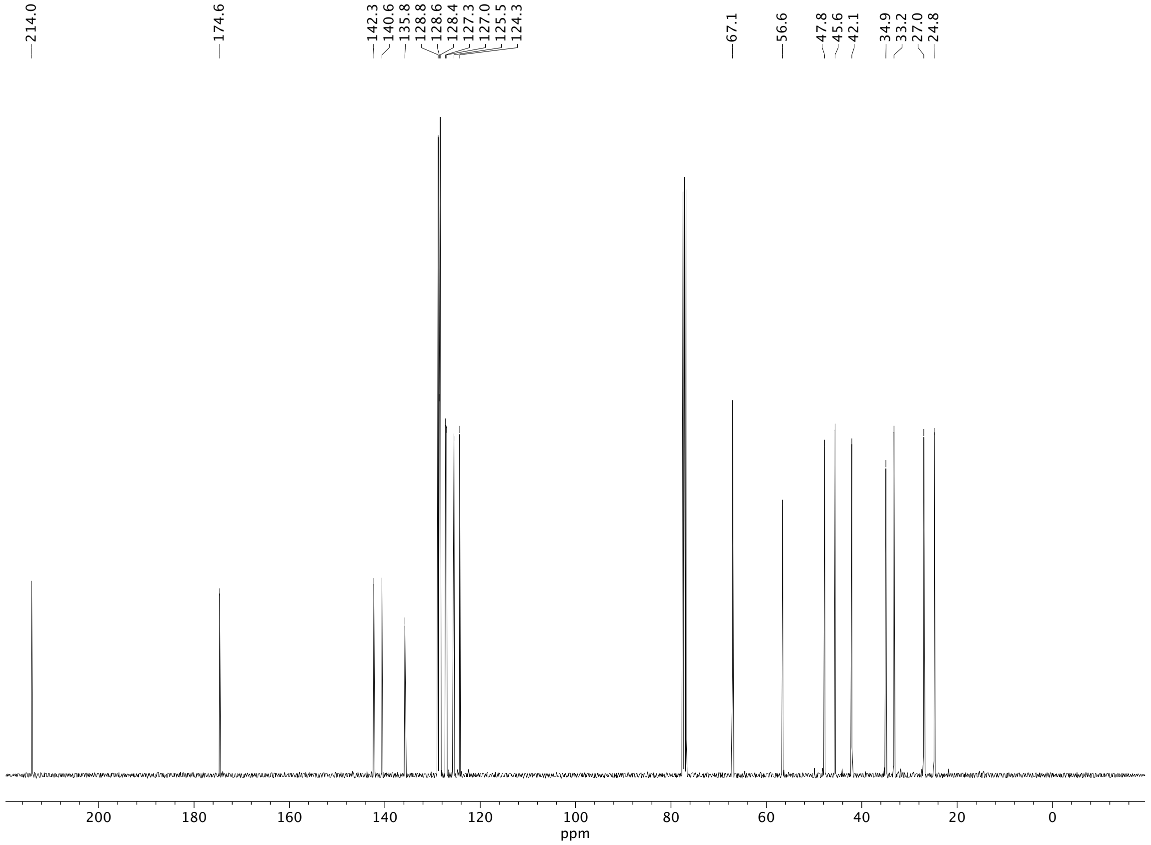

^{13}C NMR (100 MHz, $CDCl_3$) of compound **11p**.

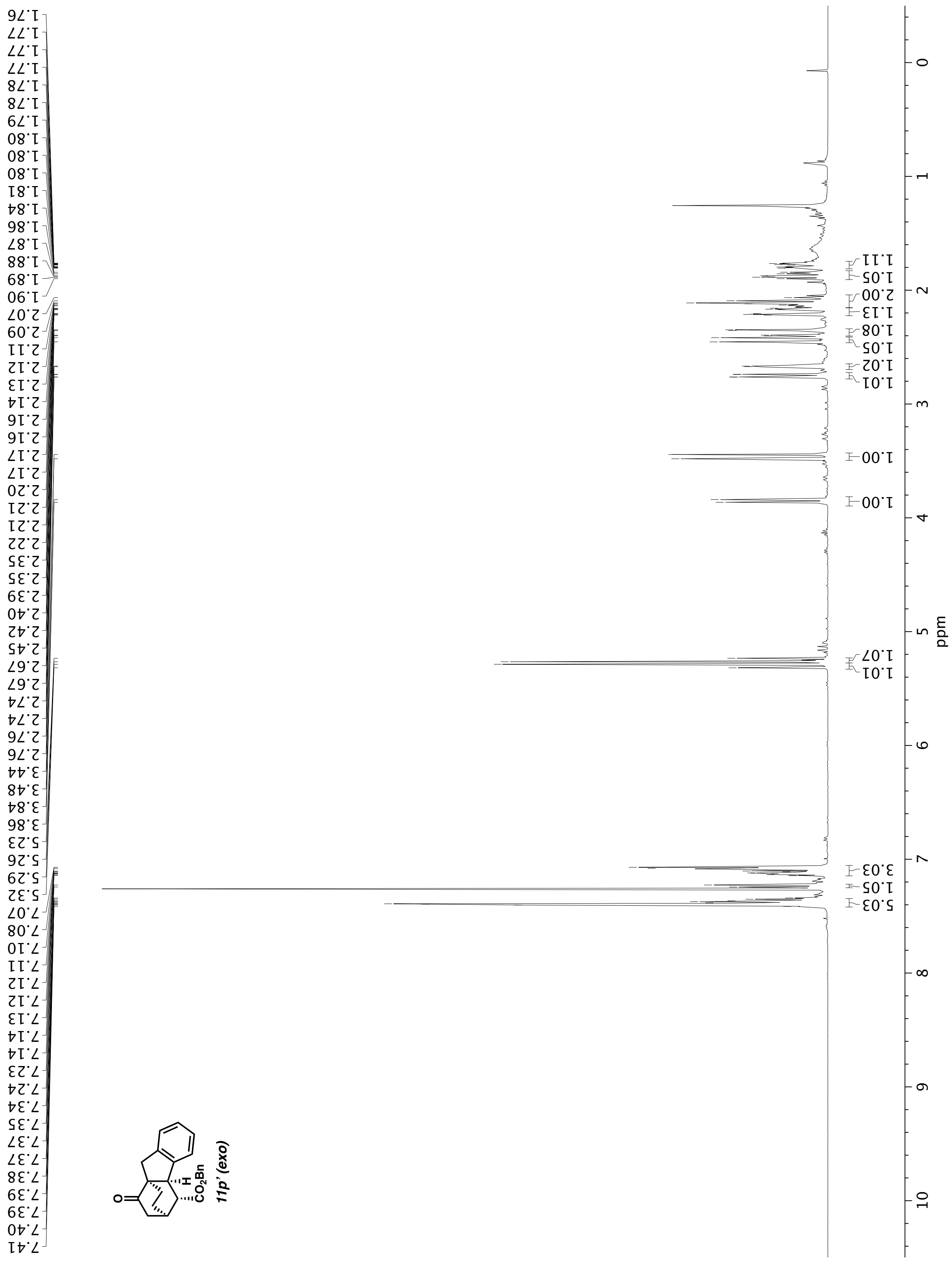

^{1}H NMR (400 MHz, $CDCl_3$) of compound **11p'**

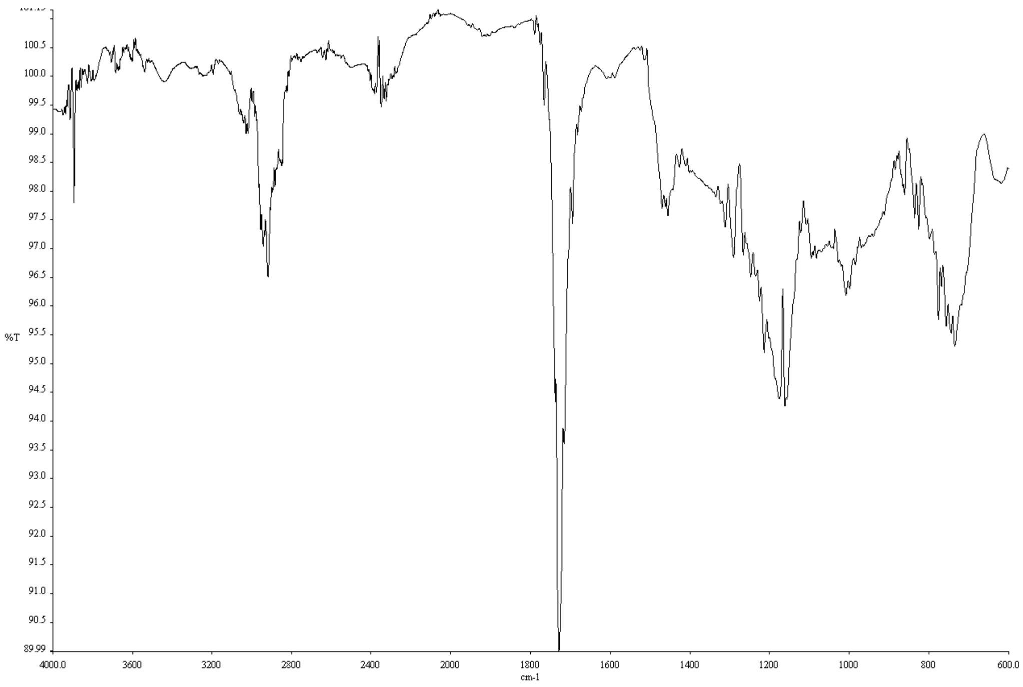

Infrared spectrum (Thin Film, NaCl) of compound **11p'**.

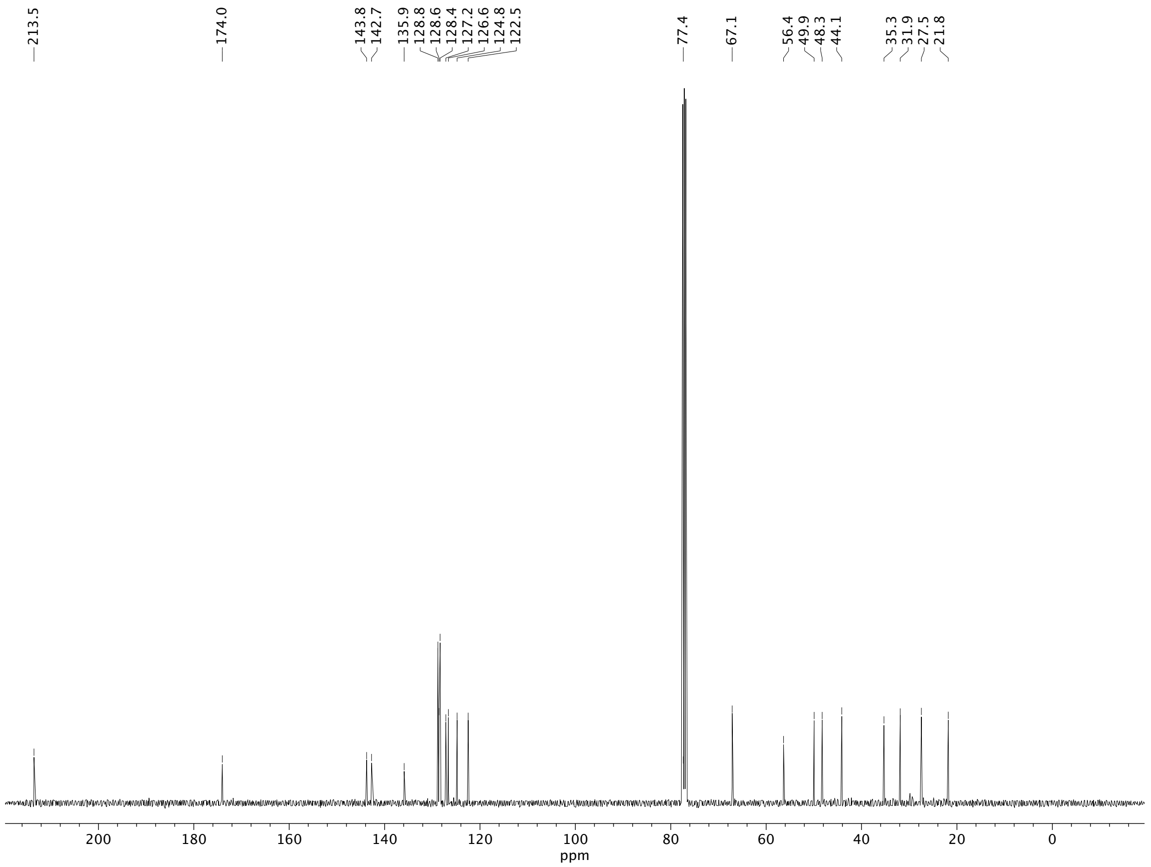

^{13}C NMR (100 MHz, $CDCl_3$) of compound **11p'**.

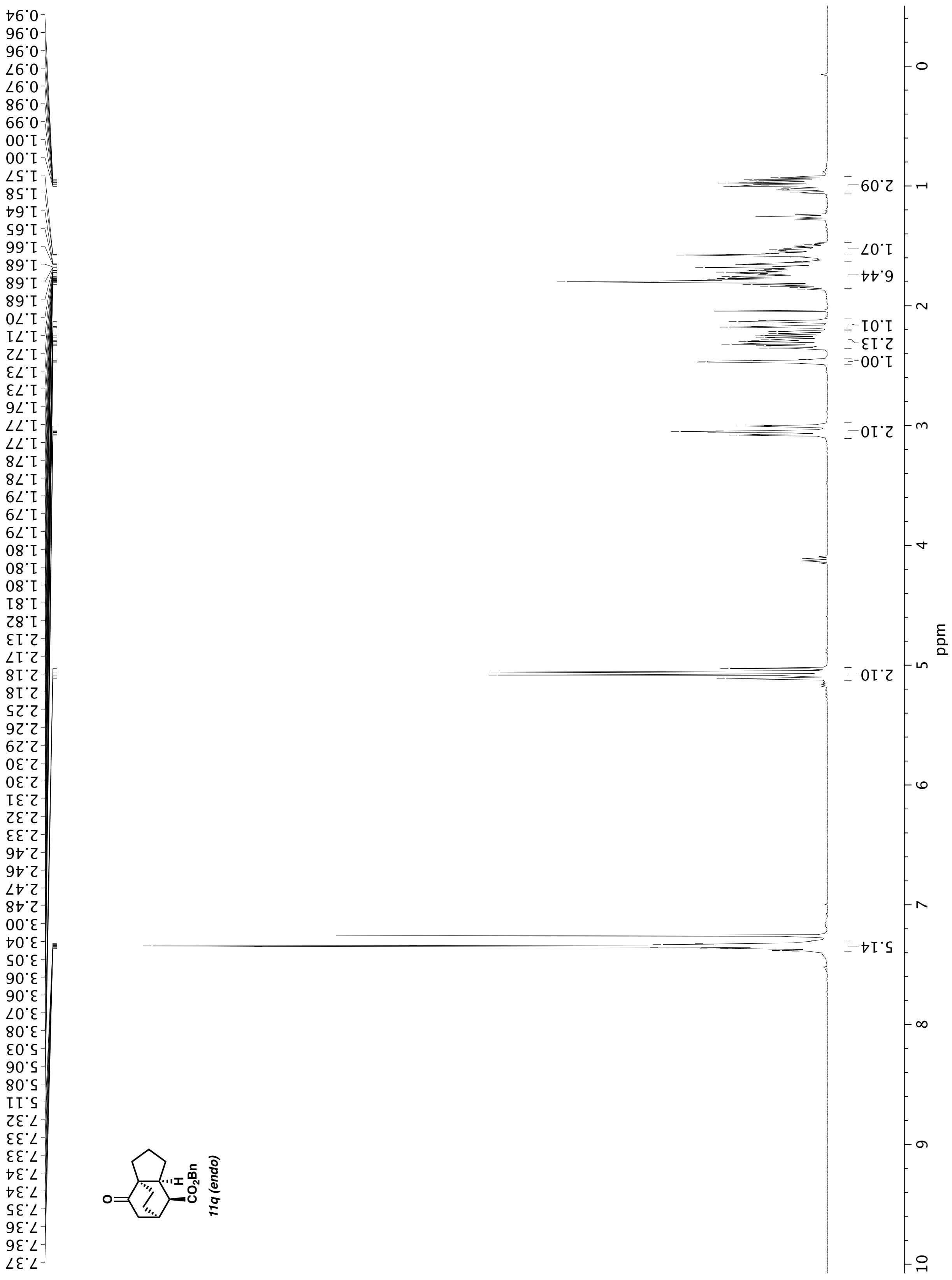

^{1}H NMR (400 MHz, $CDCl_3$) of compound **11q.**

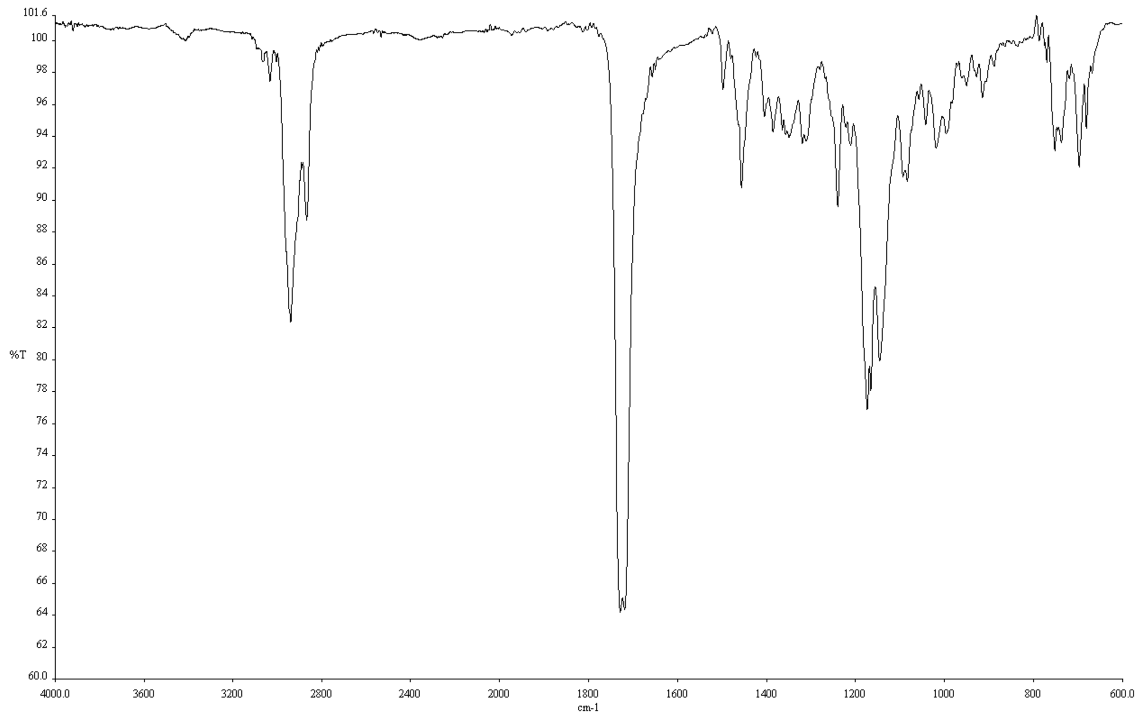

Infrared spectrum (Thin Film, NaCl) of compound **11q**.

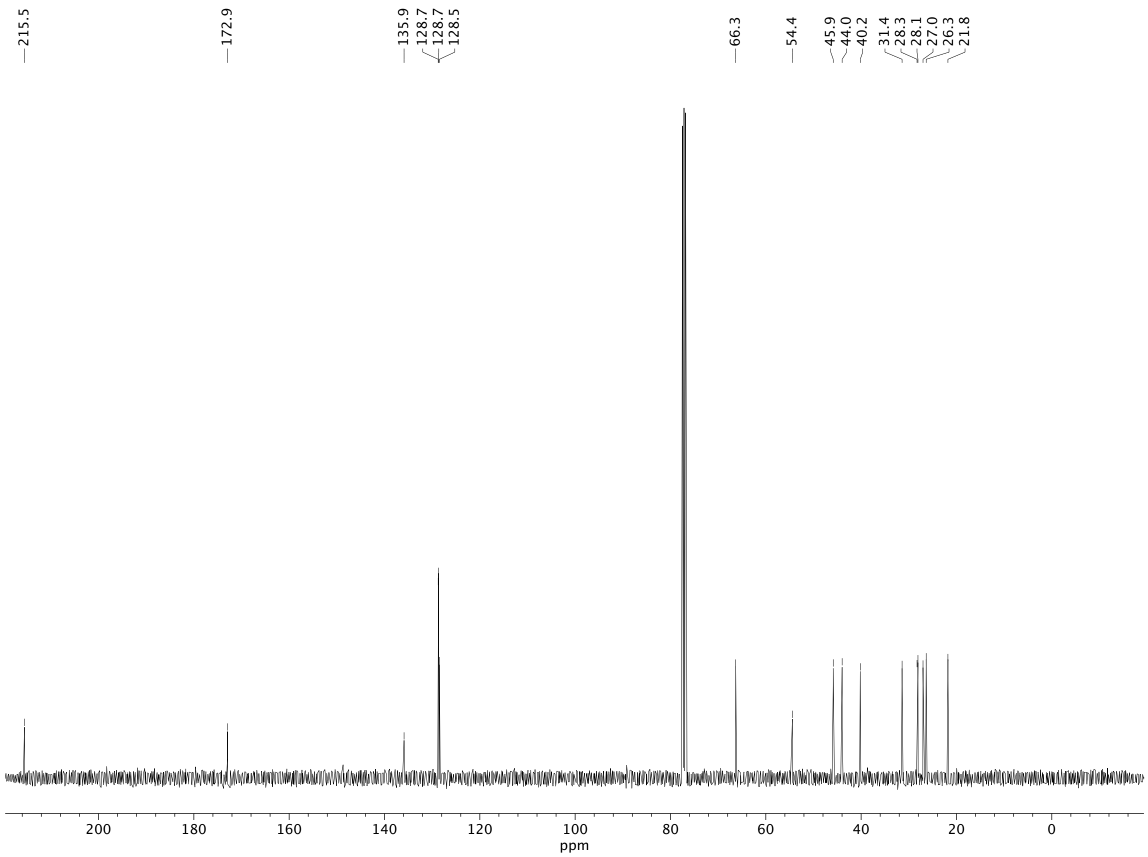

^{13}C NMR (100 MHz, $CDCl_3$) of compound **11q**.

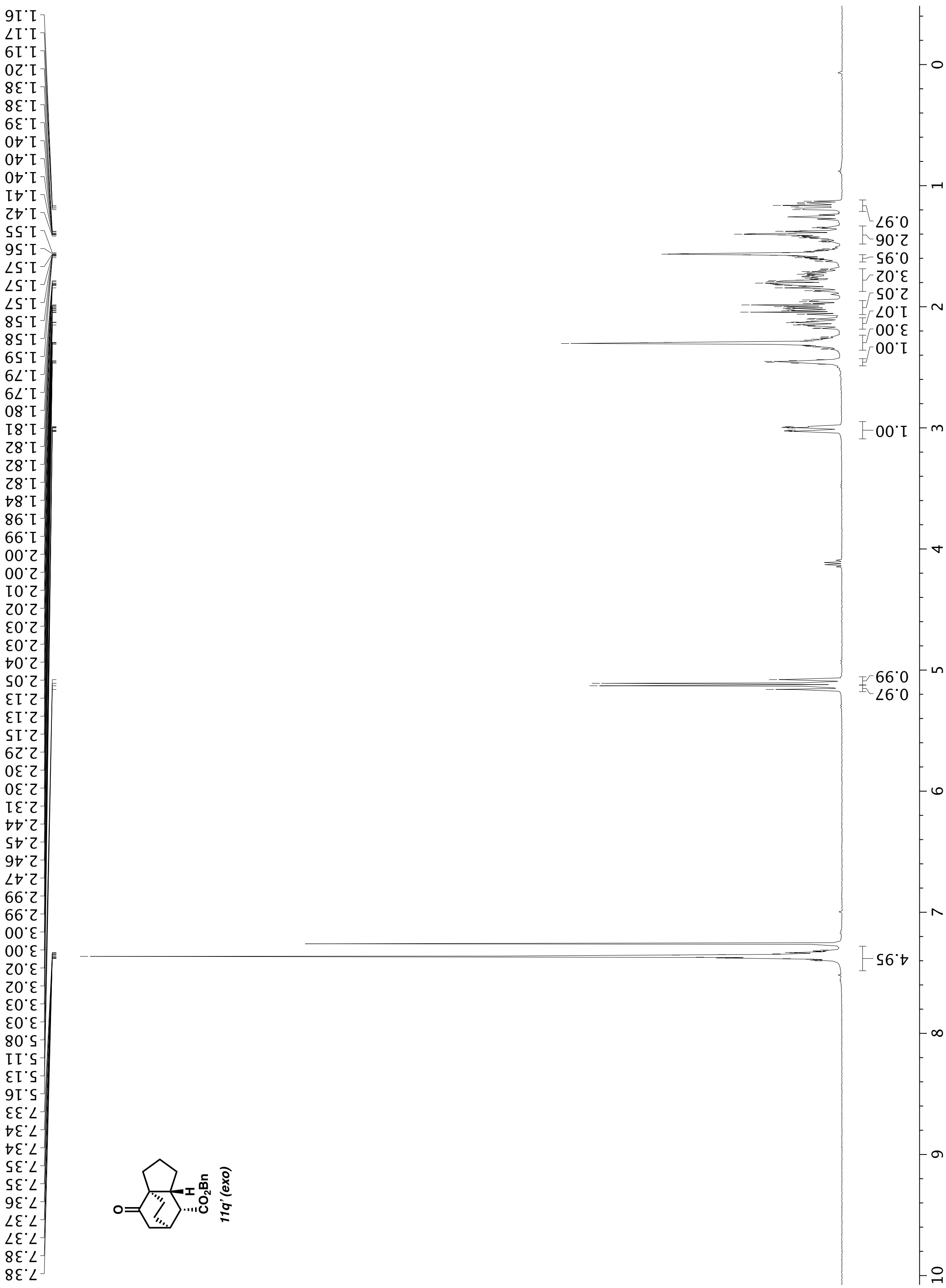

^{1}H NMR (400 MHz, $CDCl_3$) of compound **11q'**

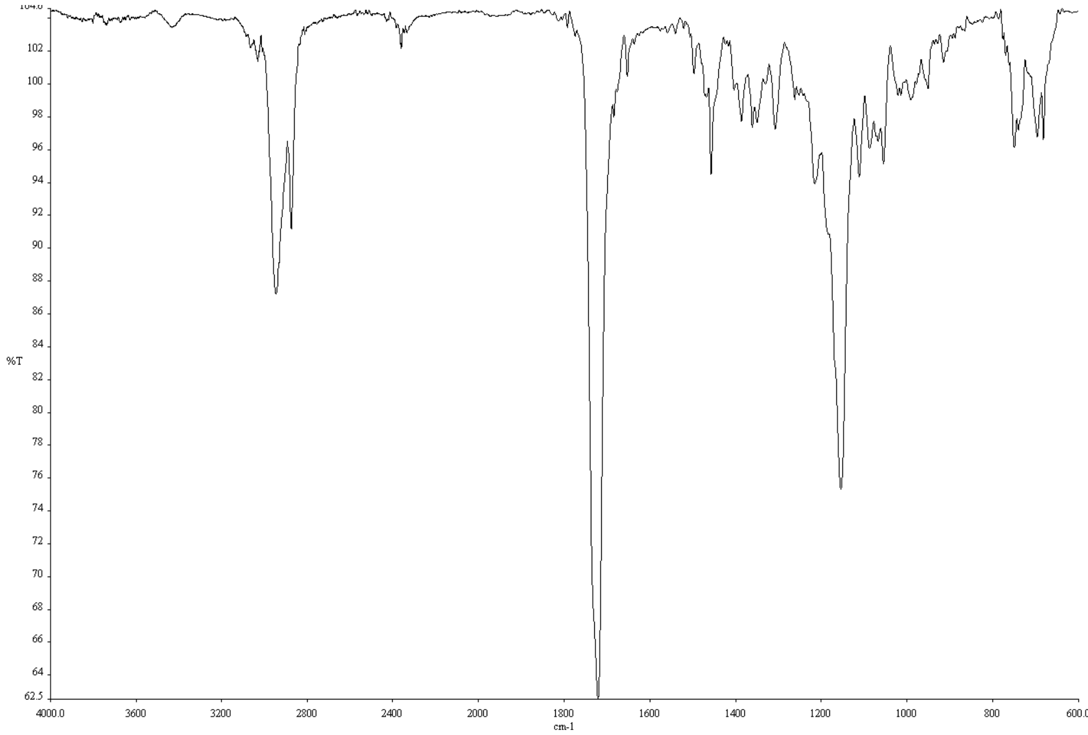

Infrared spectrum (Thin Film, NaCl) of compound **11q'**.

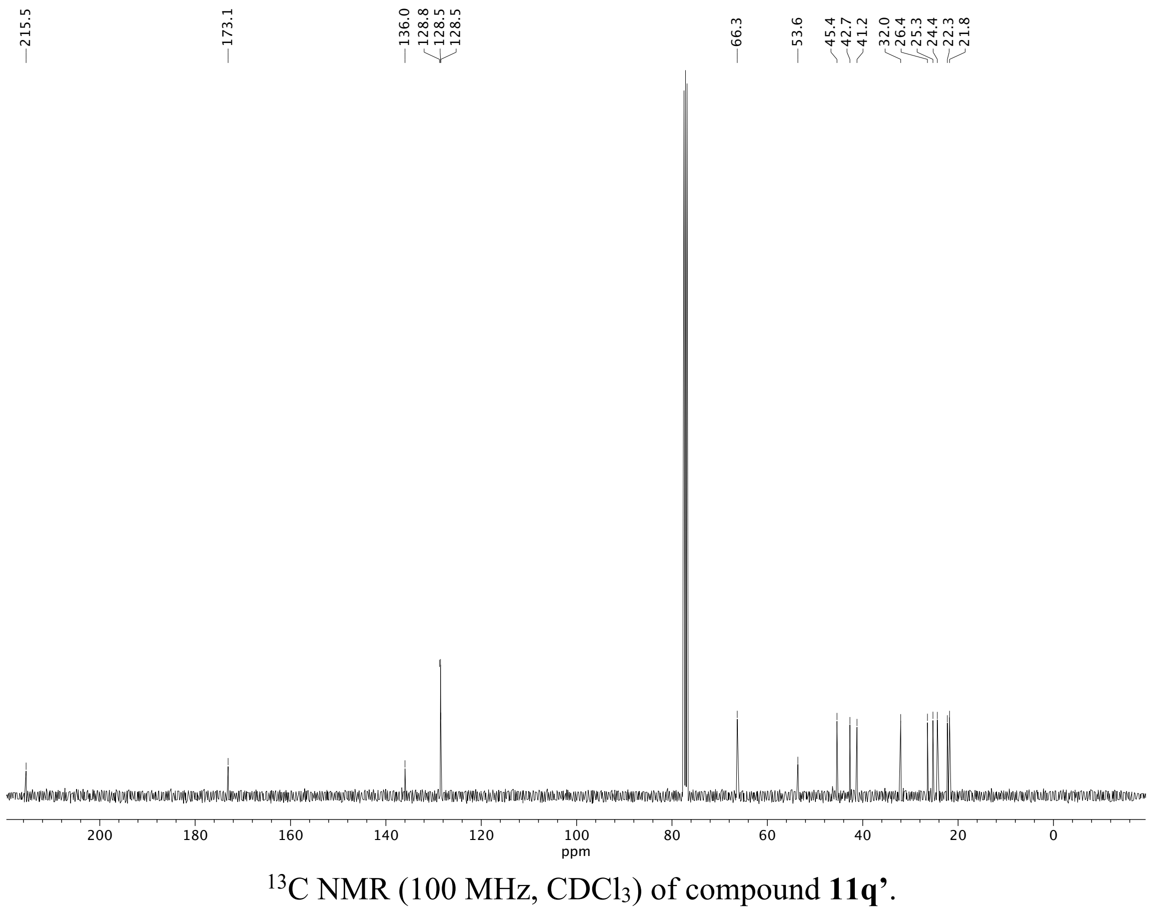

^{13}C NMR (100 MHz, $CDCl_3$) of compound **11q'**.

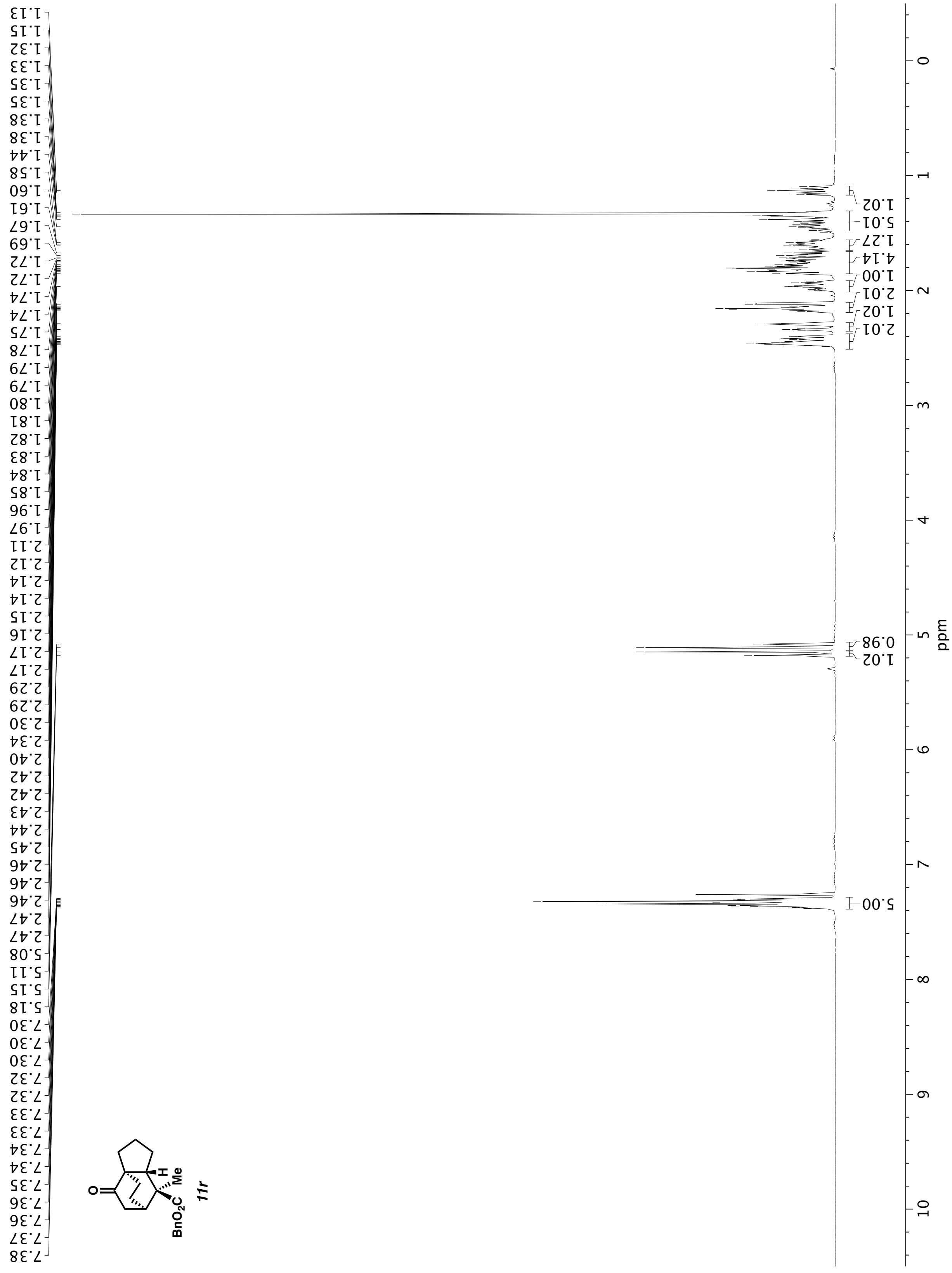

^{1}H NMR (400 MHz, $CDCl_3$) of compound **11r.**

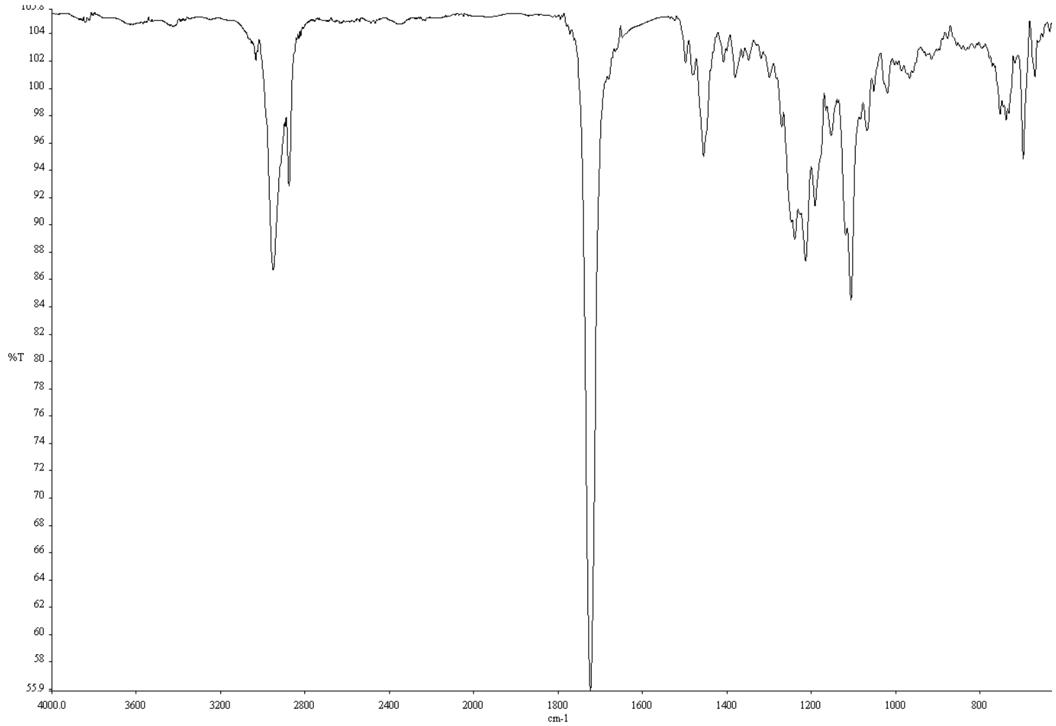

Infrared spectrum (Thin Film, NaCl) of compound **11r**.

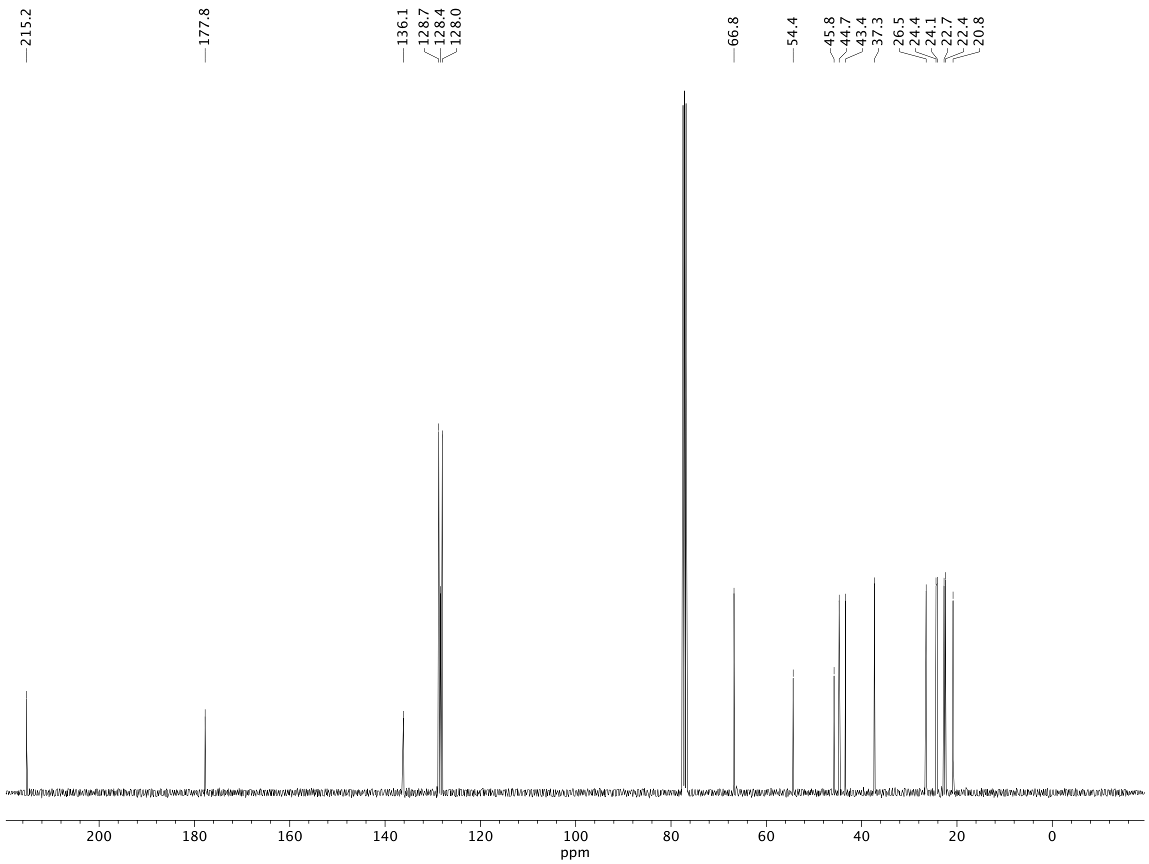

^{13}C NMR (100 MHz, $CDCl_3$) of compound **11r**.

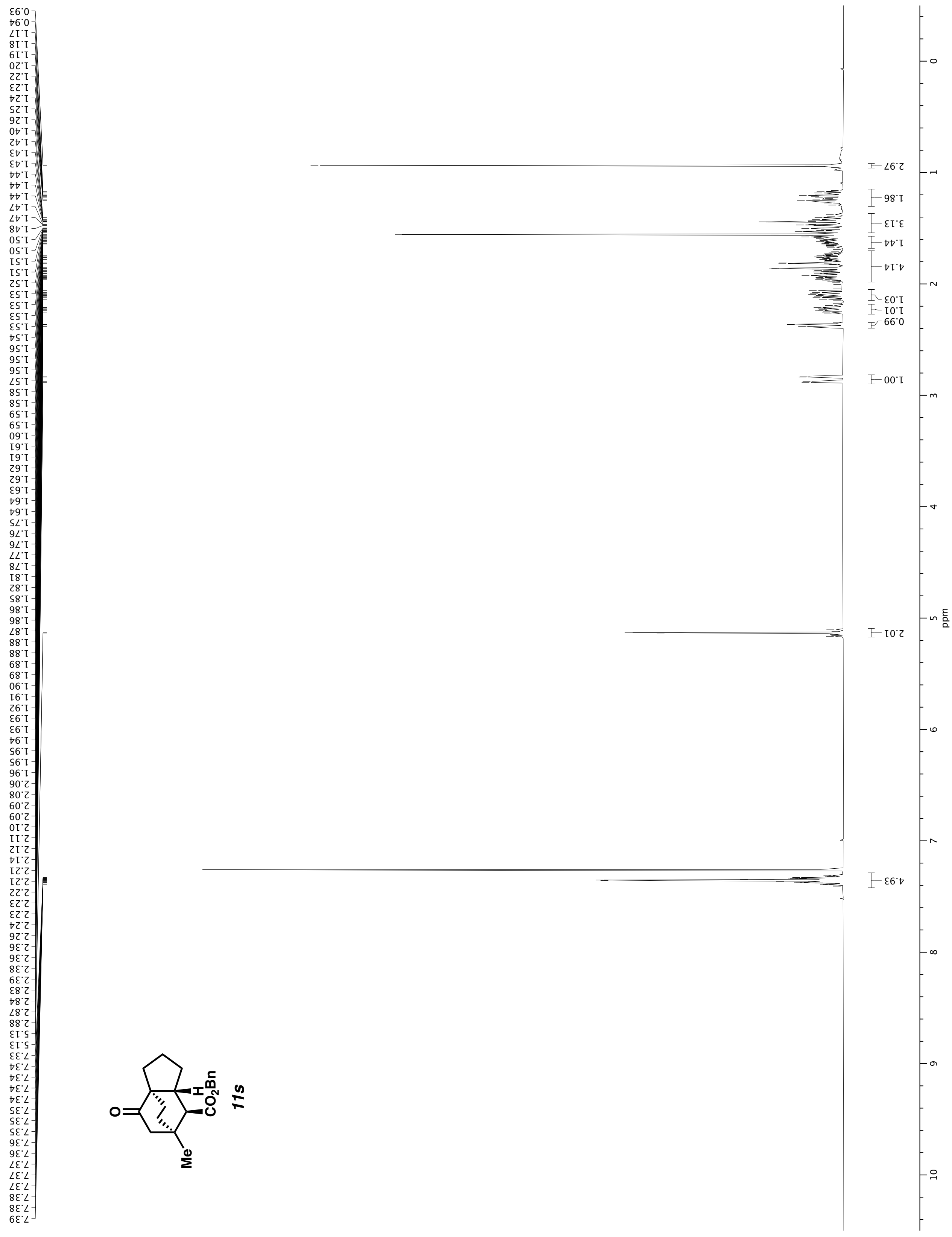

11s

^{1}H NMR (400 MHz, $CDCl_3$) of compound **11s**.

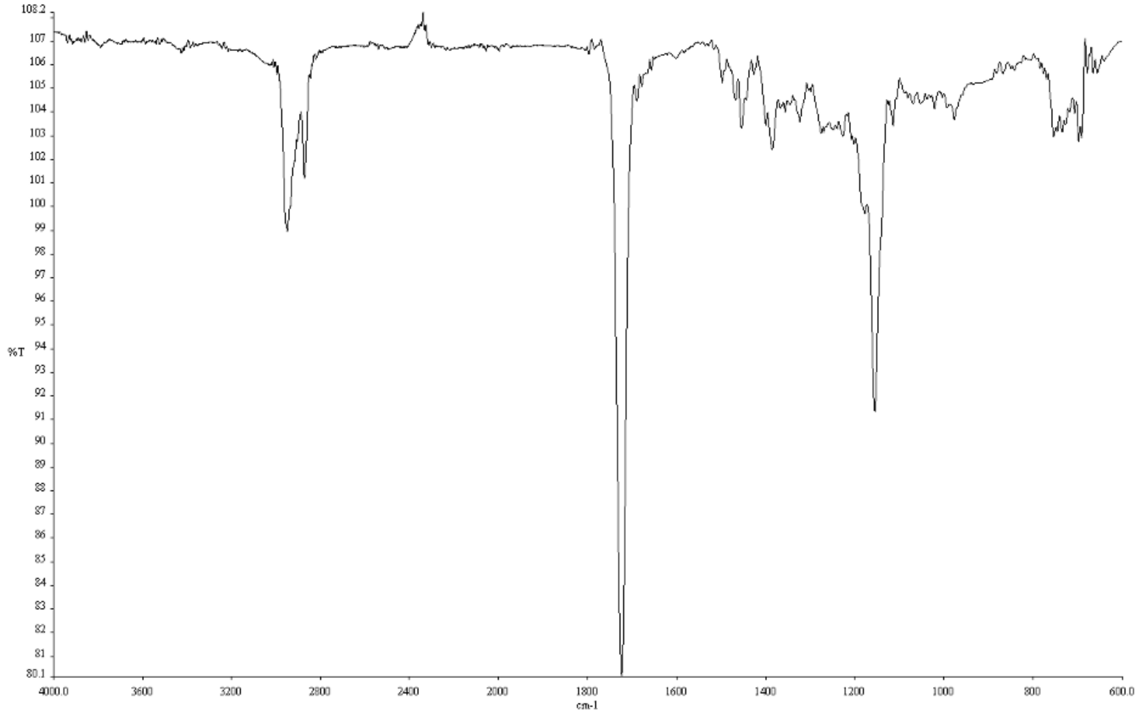

Infrared spectrum (Thin Film, NaCl) of compound **11s**.

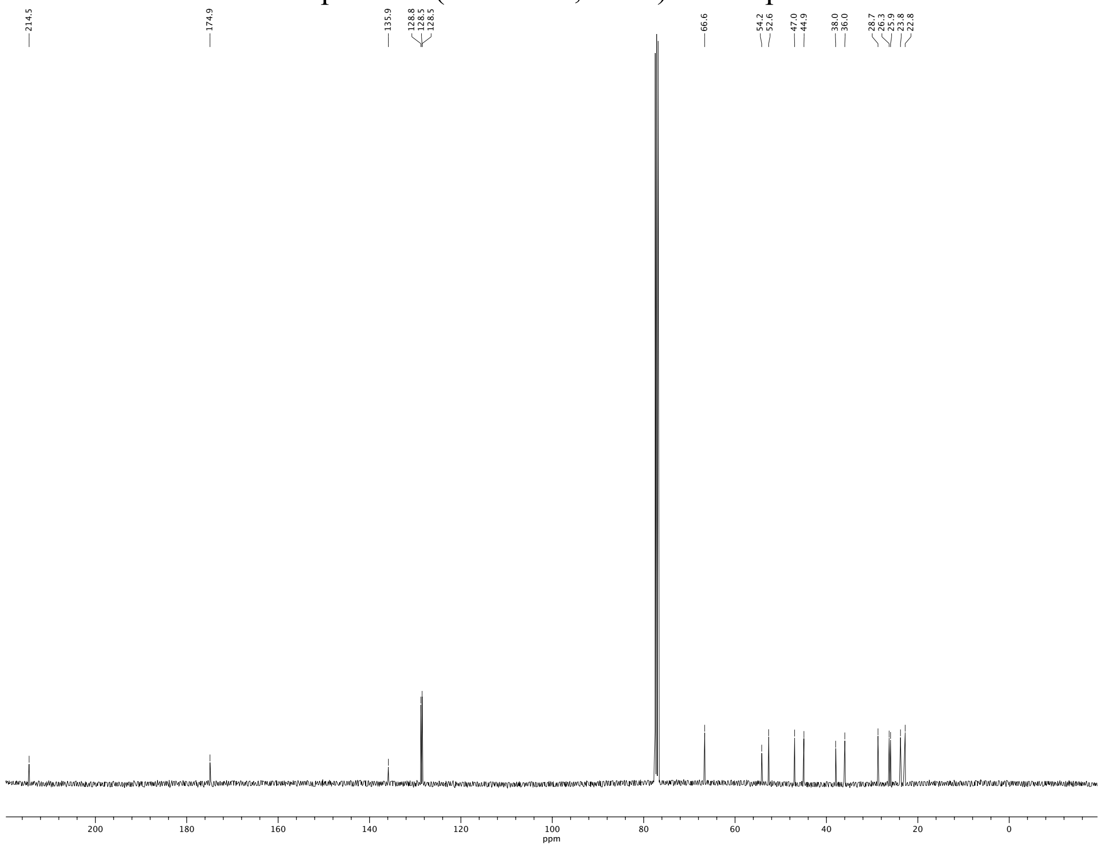

^{13}C NMR (100 MHz, $CDCl_3$) of compound **11s**.

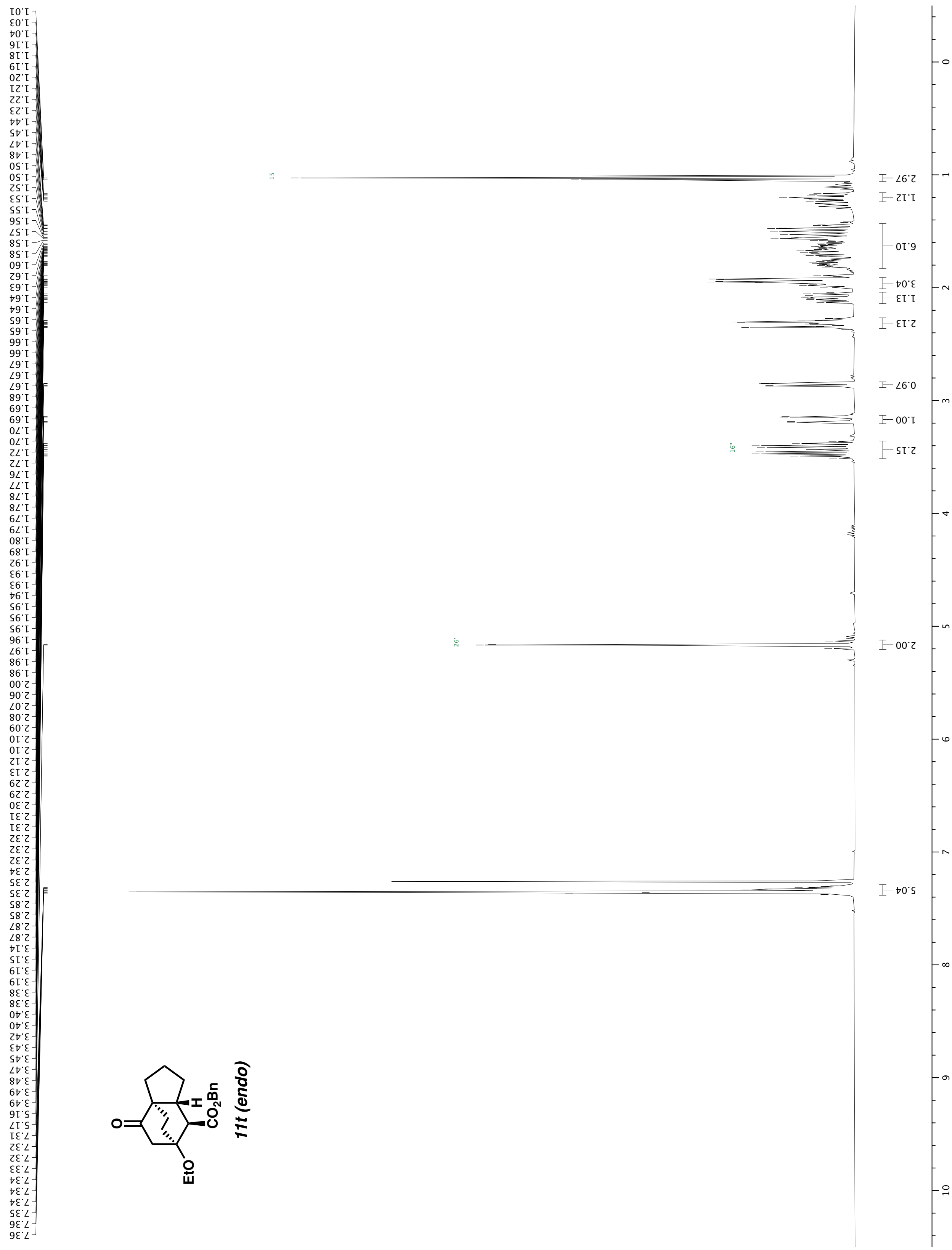

^{1}H NMR (400 MHz, $CDCl_3$) of compound **11t**.

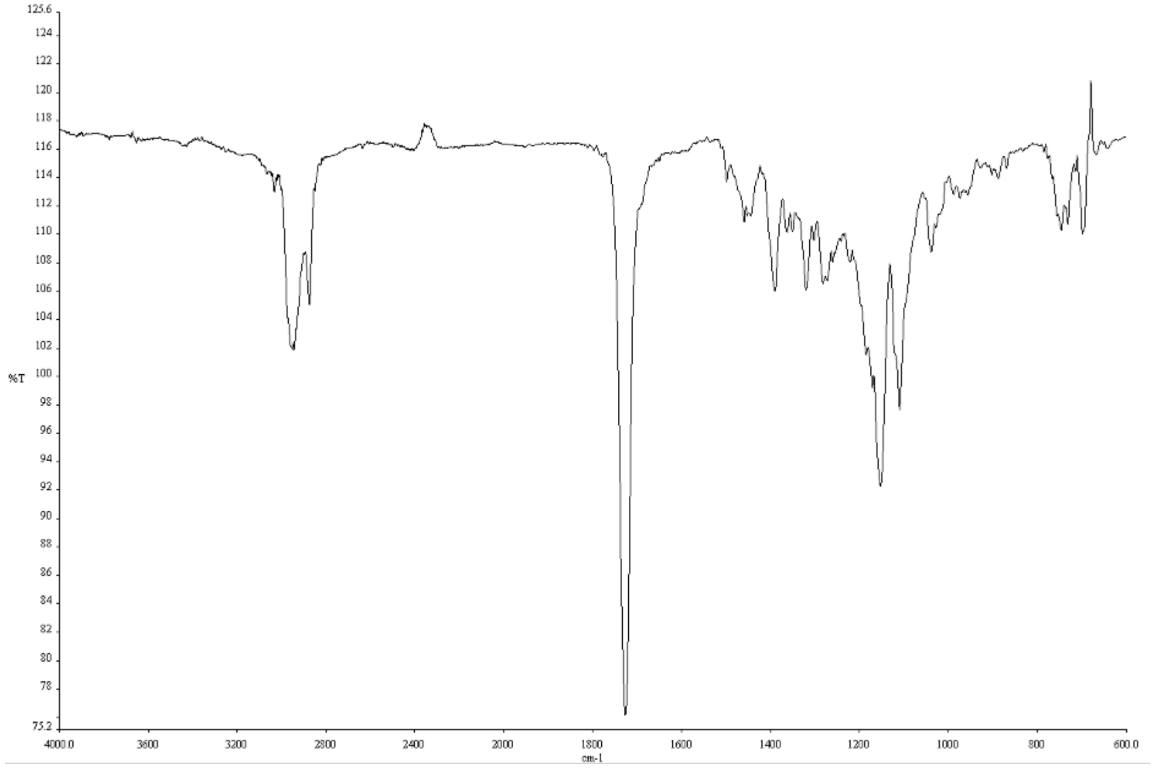

Infrared spectrum (Thin Film, NaCl) of compound **11t**.

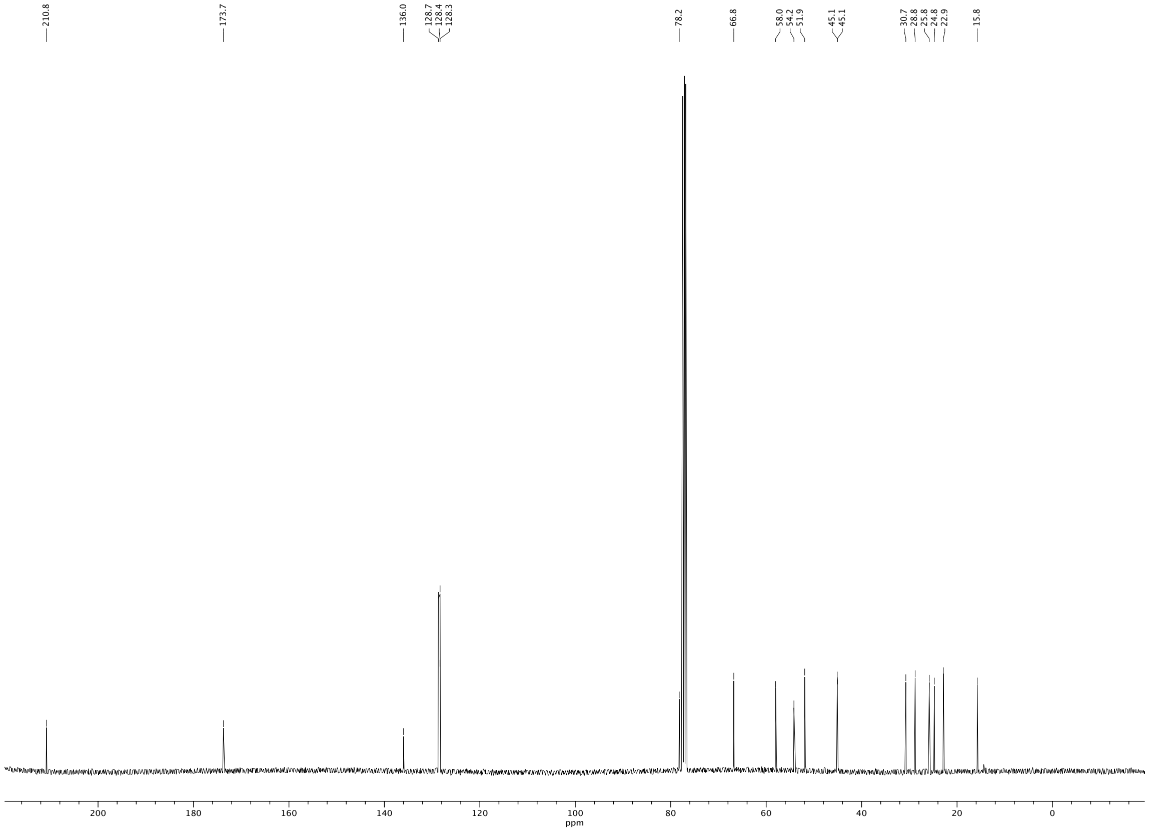

^{13}C NMR (100 MHz, $CDCl_3$) of compound **11t**.

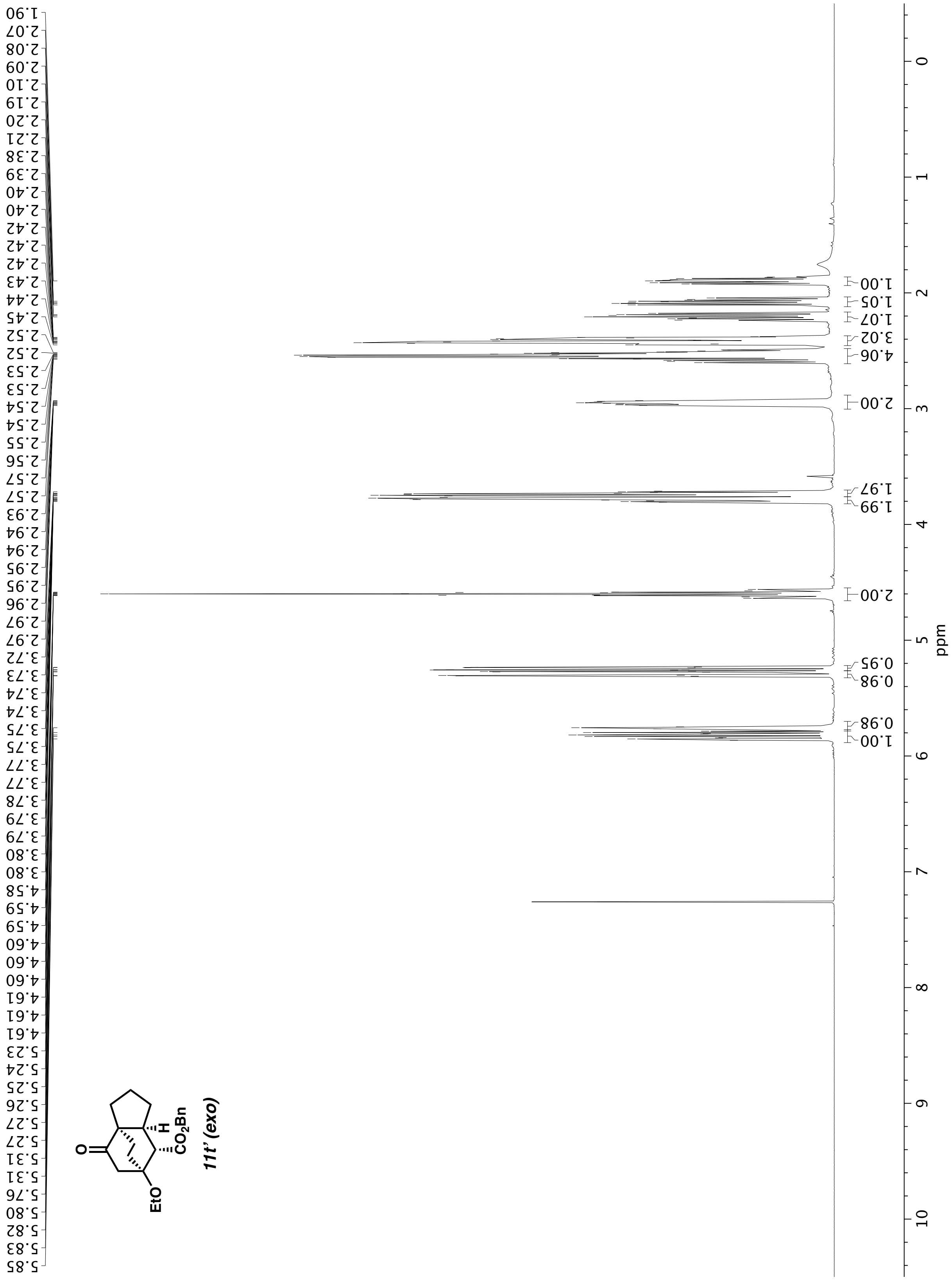

^{1}H NMR (400 MHz, $CDCl_3$) of compound **11t'** .

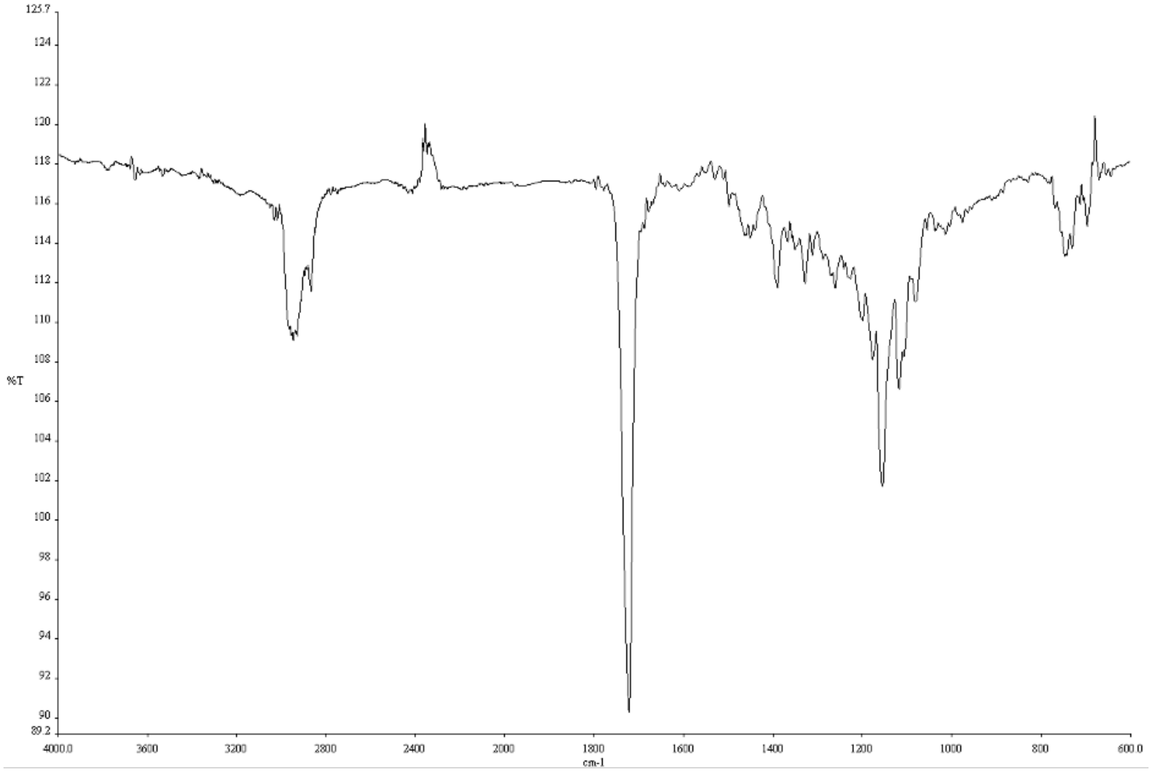

Infrared spectrum (Thin Film, NaCl) of compound **11t'**.

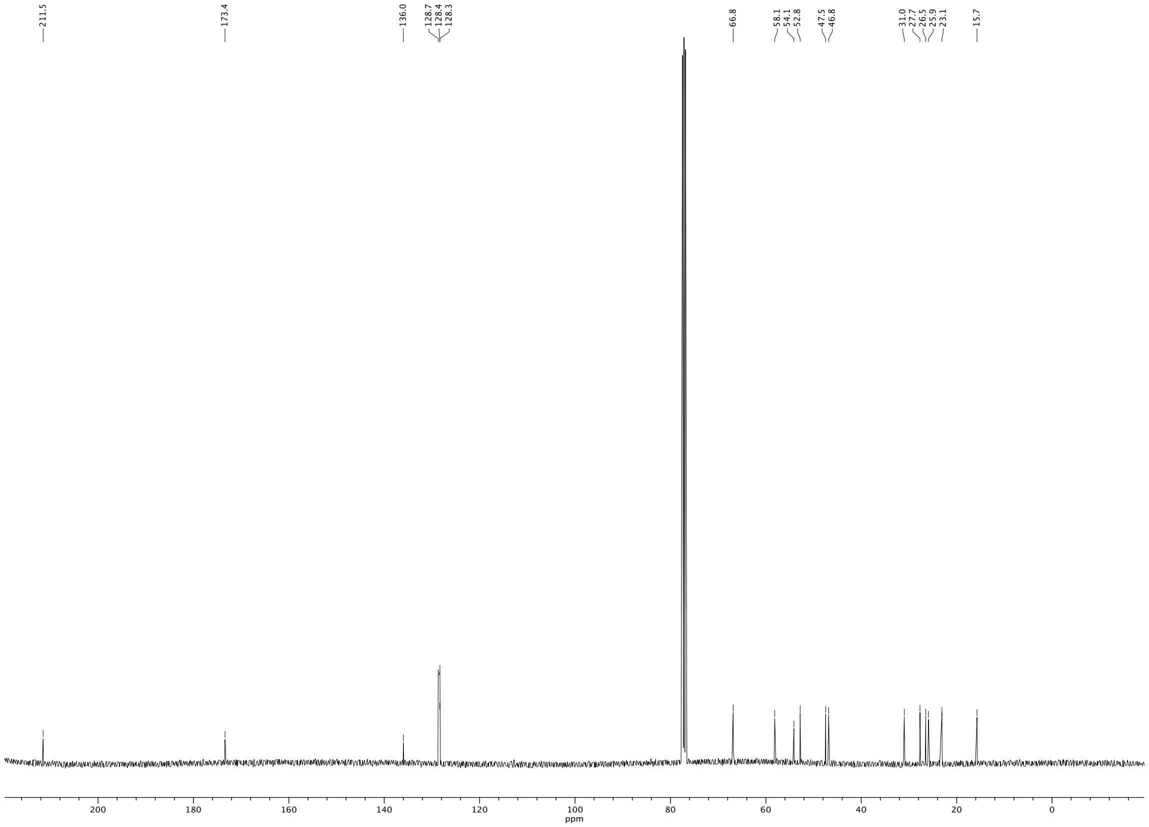

^{13}C NMR (100 MHz, $CDCl_3$) of compound **11t'**.

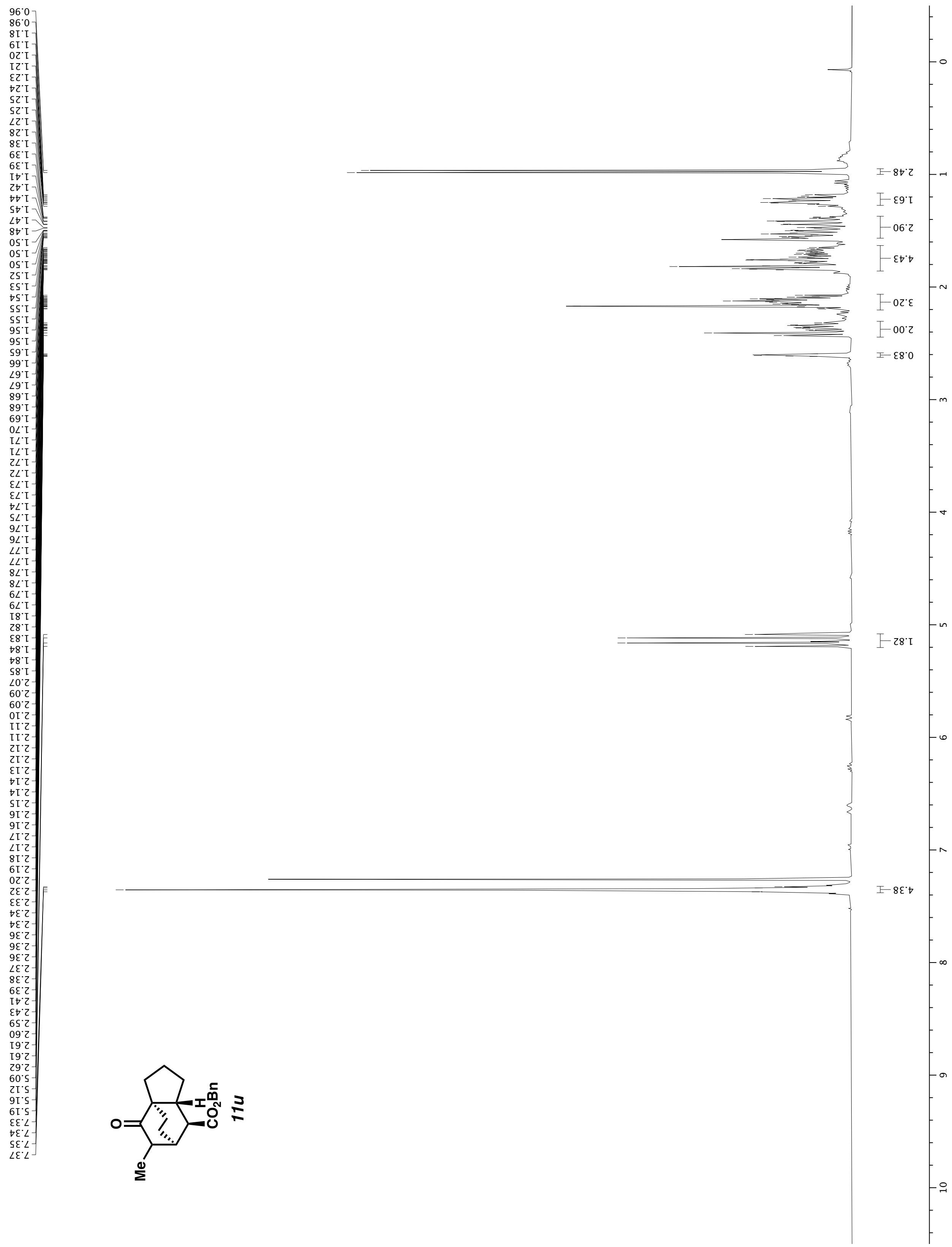

^{1}H NMR (400 MHz, $CDCl_3$) of compound **11u**.

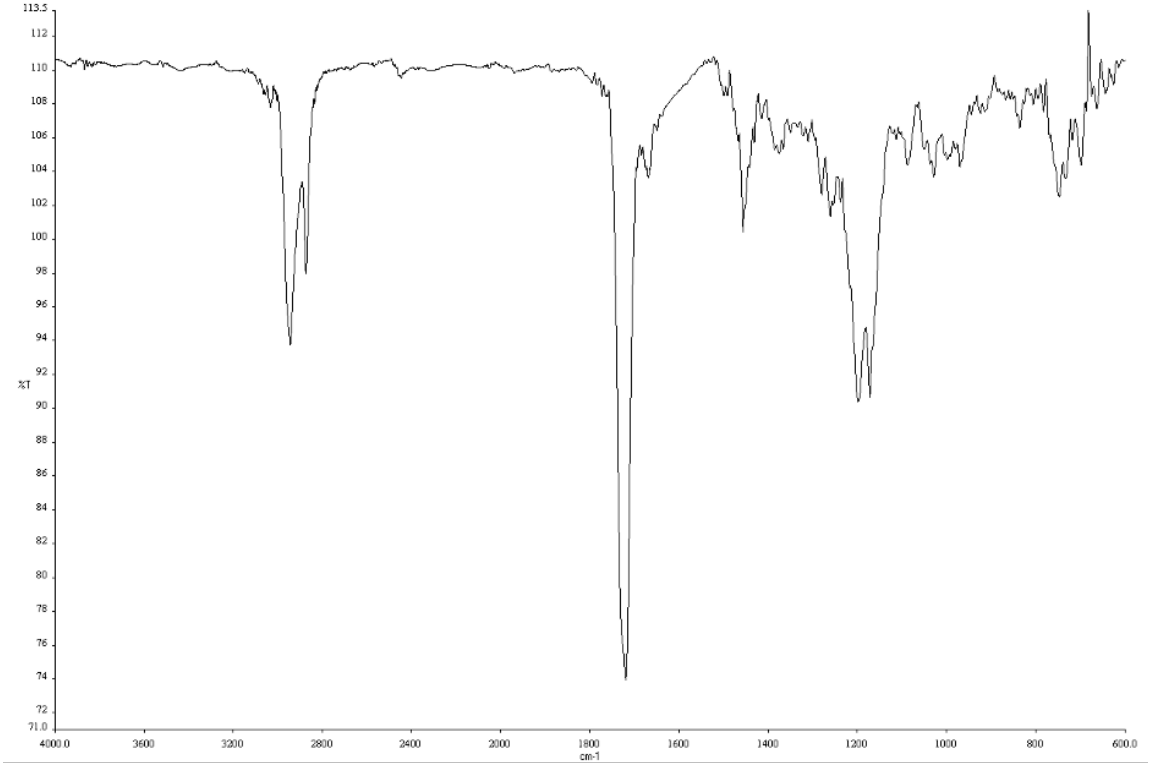

Infrared spectrum (Thin Film, NaCl) of compound **11u**.

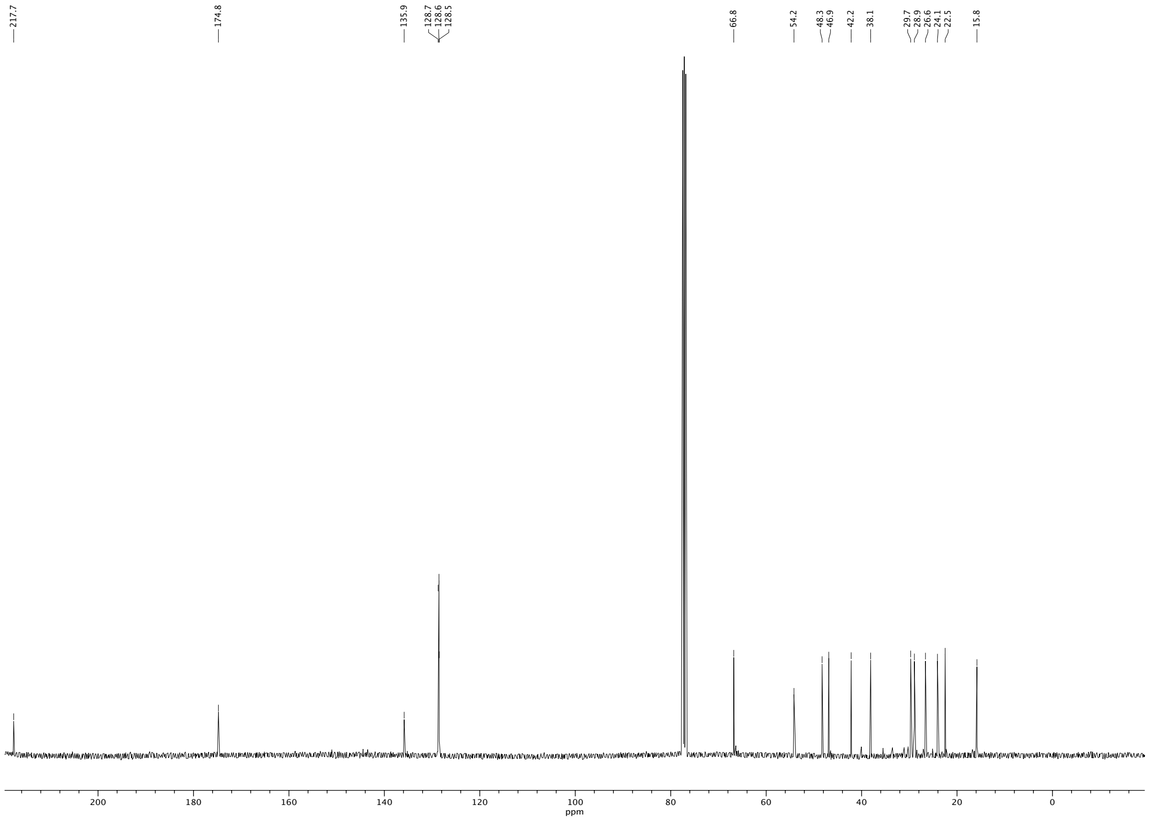

^{13}C NMR (100 MHz, $CDCl_3$) of compound **11u**.

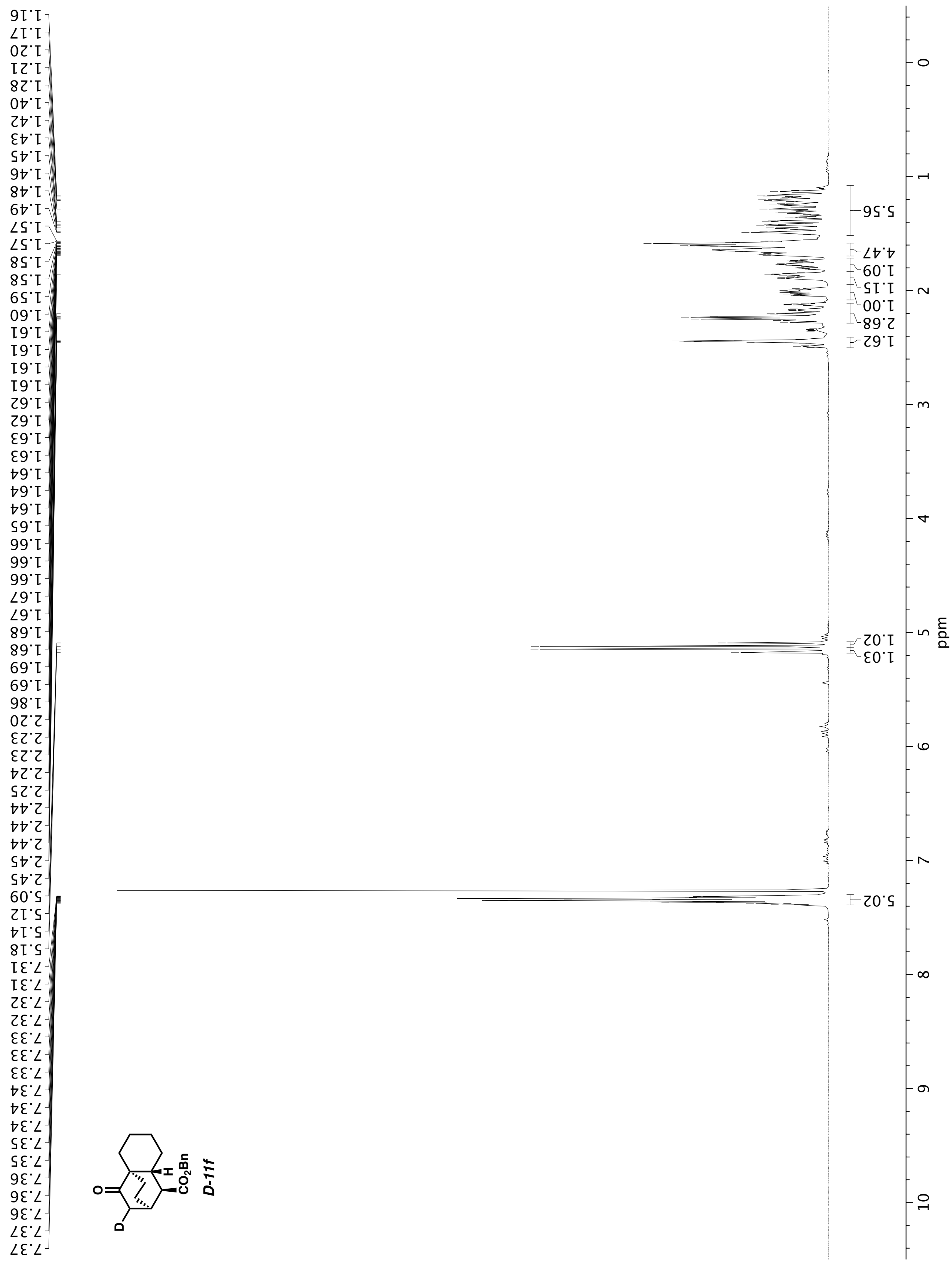

^{1}H NMR (400 MHz, $CDCl_3$) of compound ***D*-11f.**

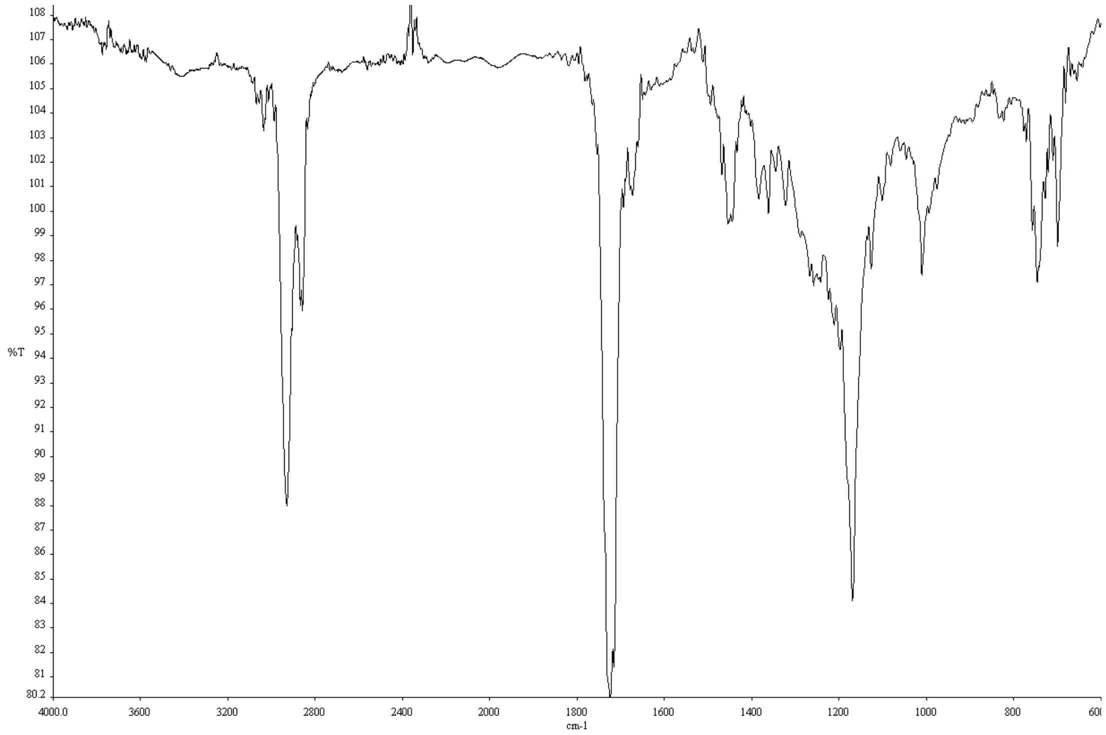

Infrared spectrum (Thin Film, NaCl) of compound ***D*-11f**.

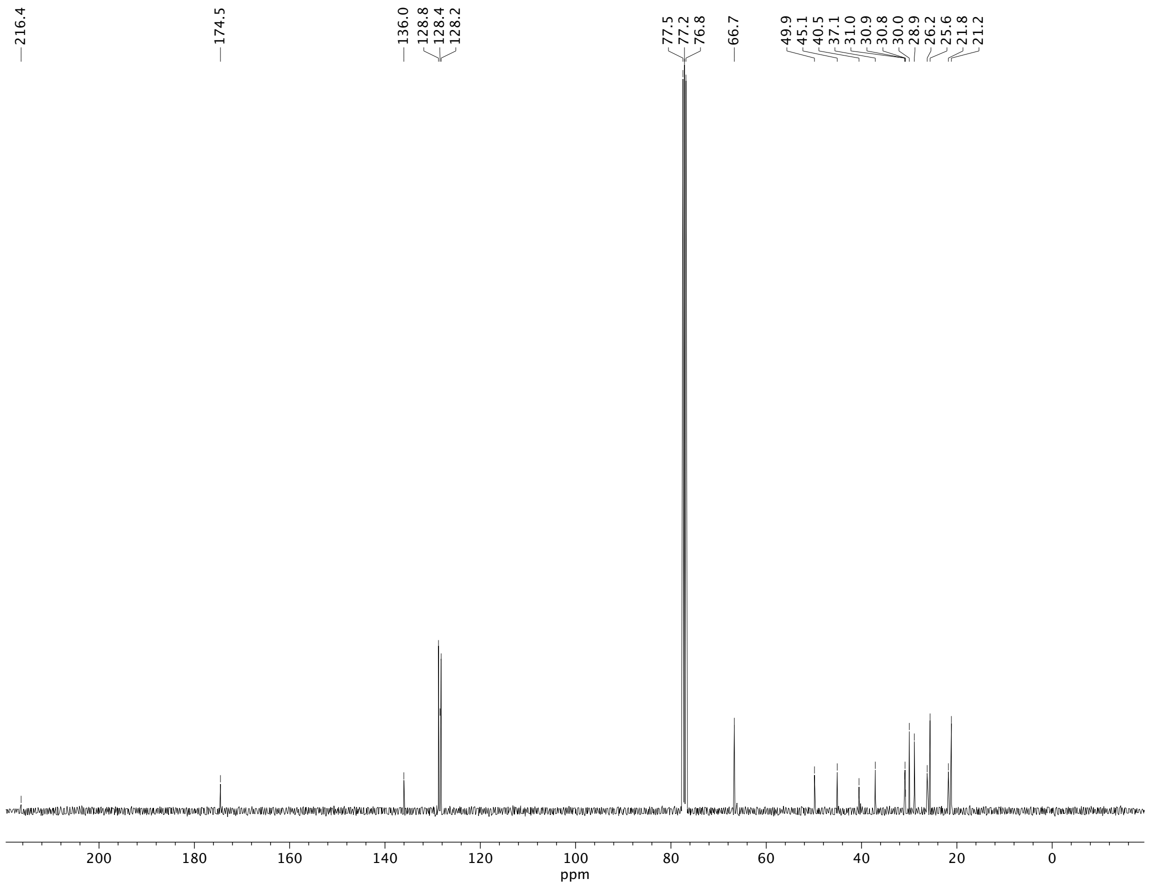

^{13}C NMR (100 MHz, $CDCl_3$) of compound ***D*-11f**.

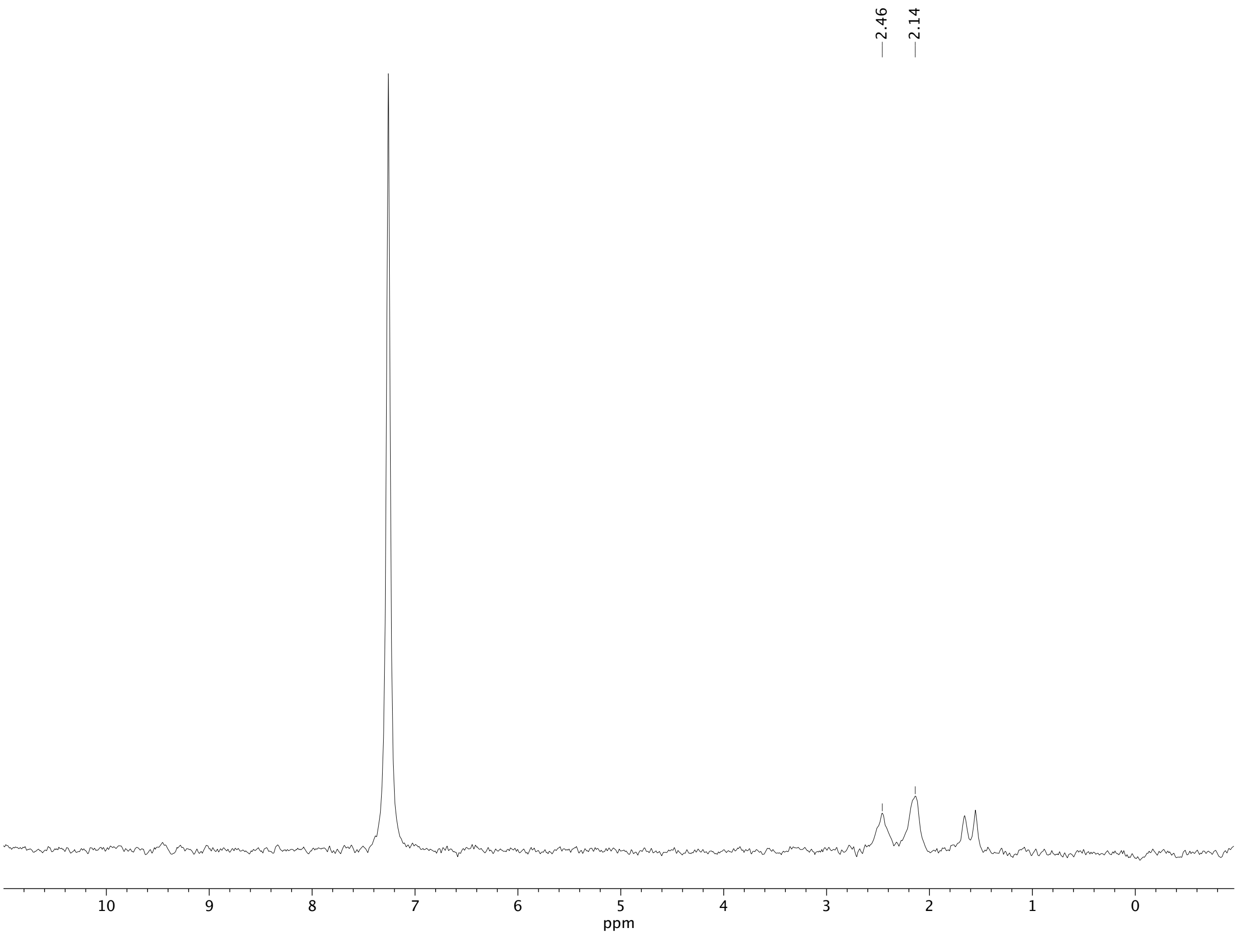

^{2}H NMR (61 MHz, $CHCl_3$) of compound ***D*-11f**.

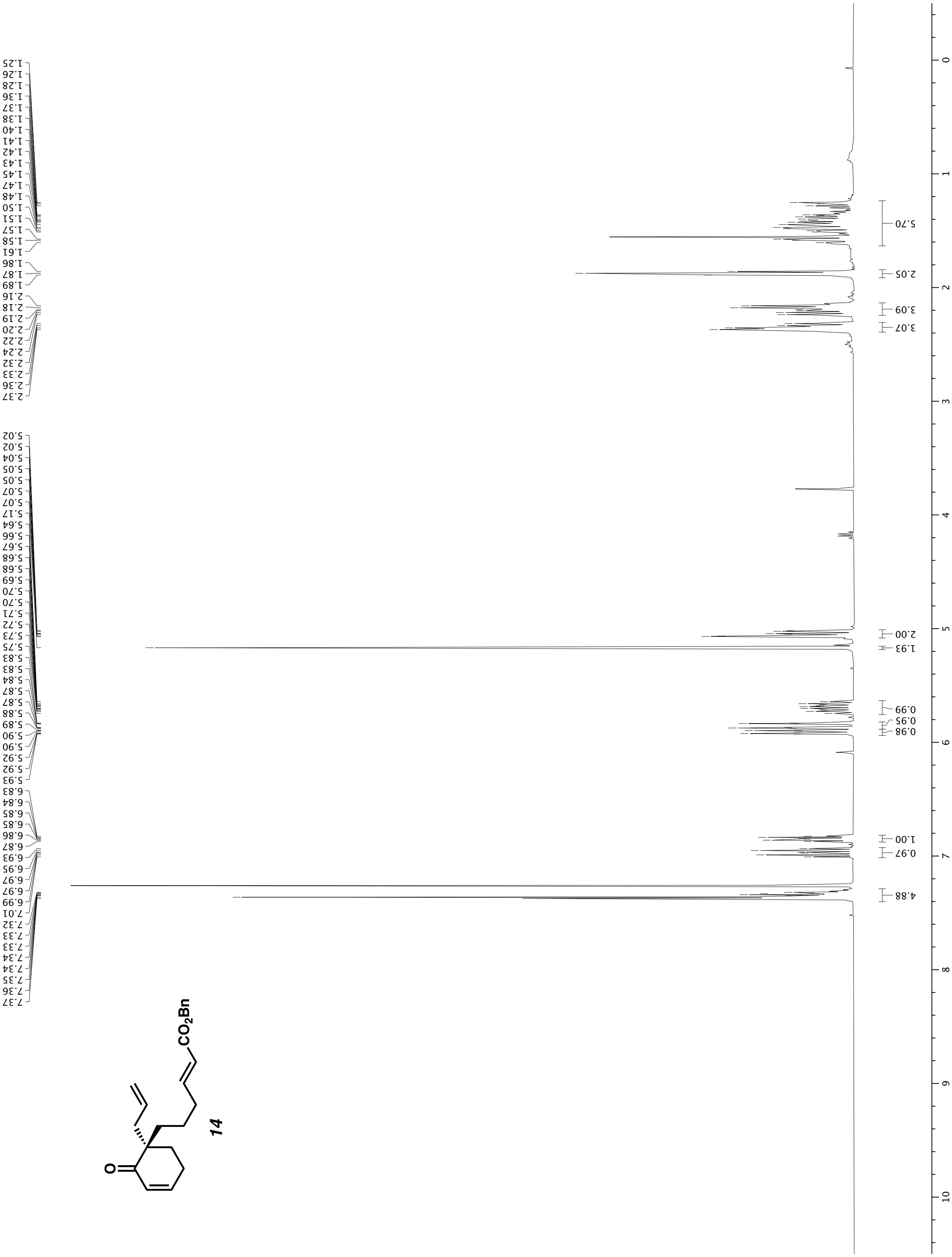

^{1}H NMR (400 MHz, $CDCl_3$) of compound **14**.

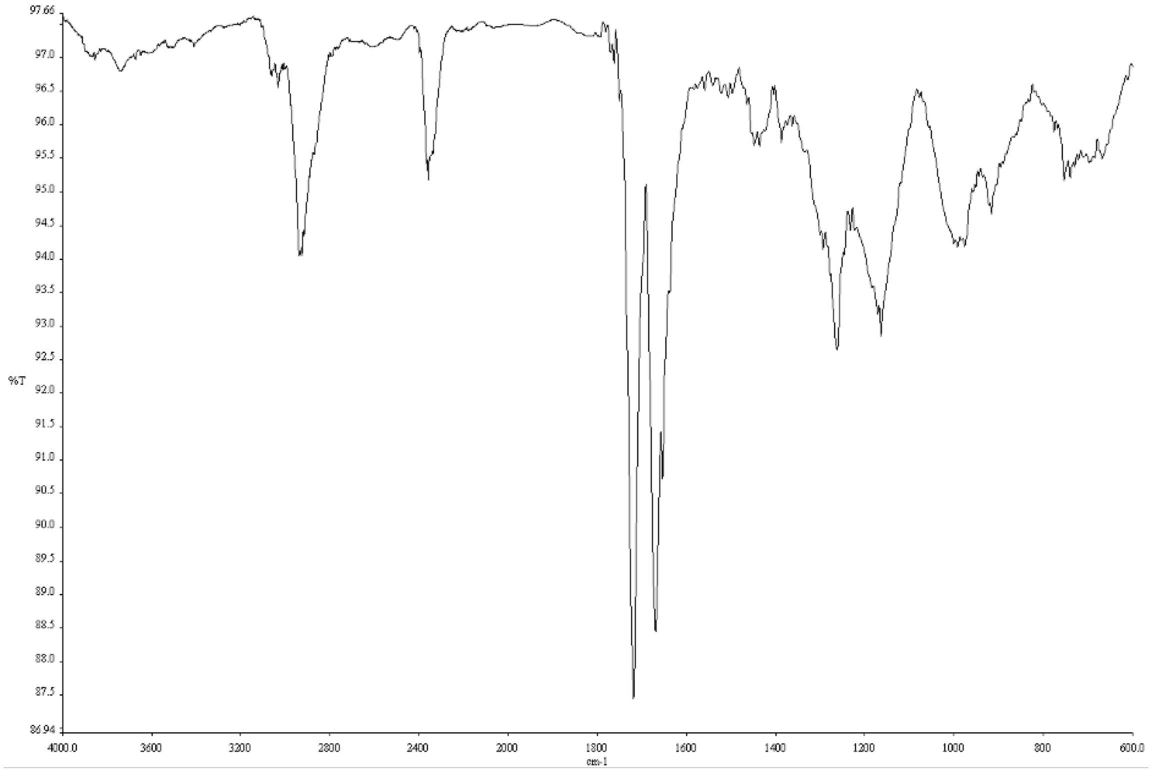

Infrared spectrum (Thin Film, NaCl) of compound **14**.

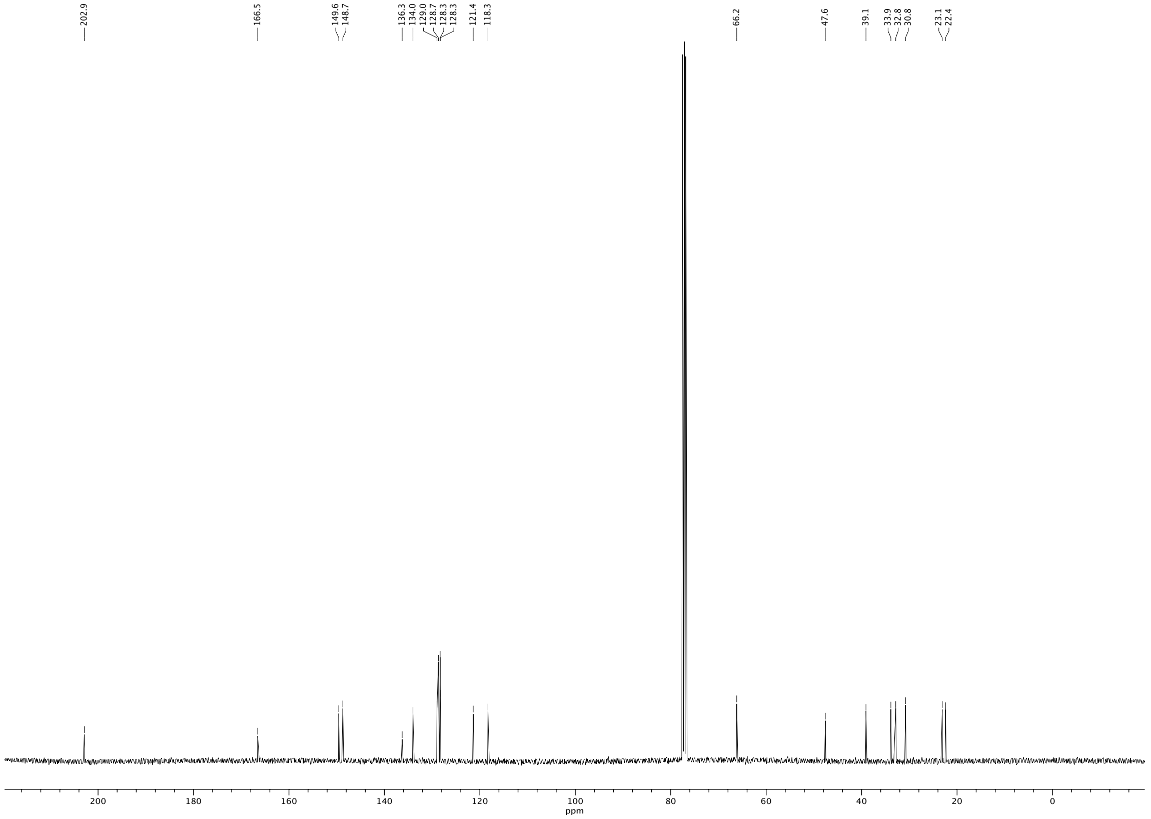

^{13}C NMR (100 MHz, $CDCl_3$) of compound **14**.

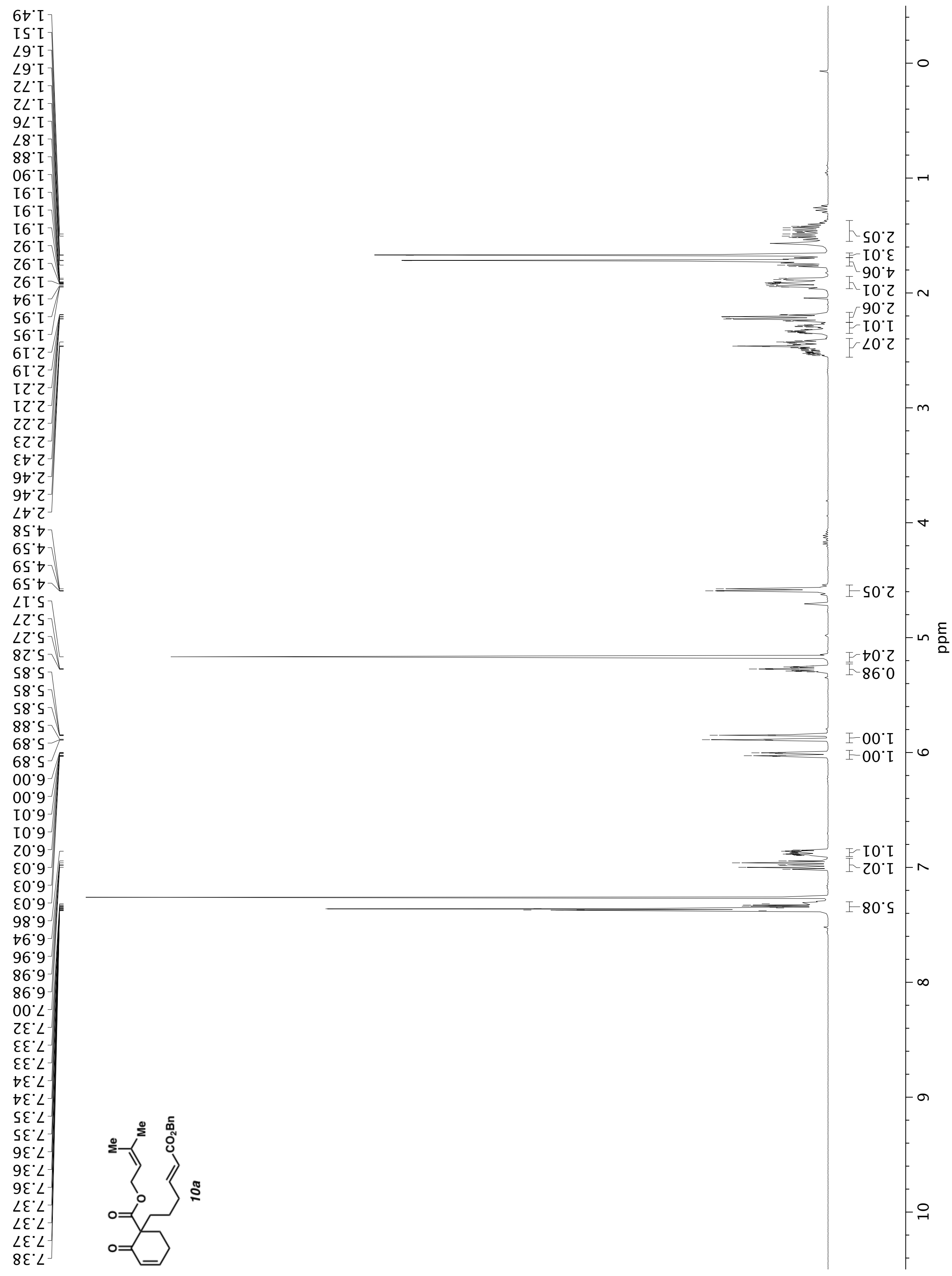

^{1}H NMR (400 MHz, $CDCl_3$) of compound **10a.**

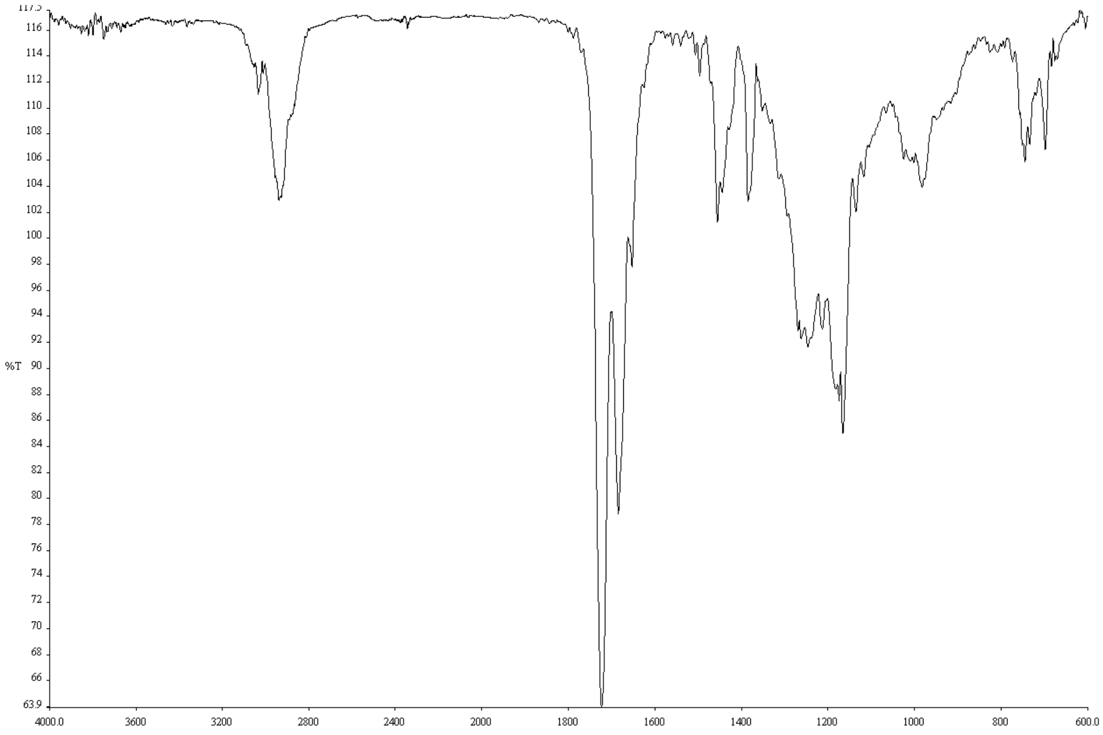

Infrared spectrum (Thin Film, NaCl) of compound **10a**.

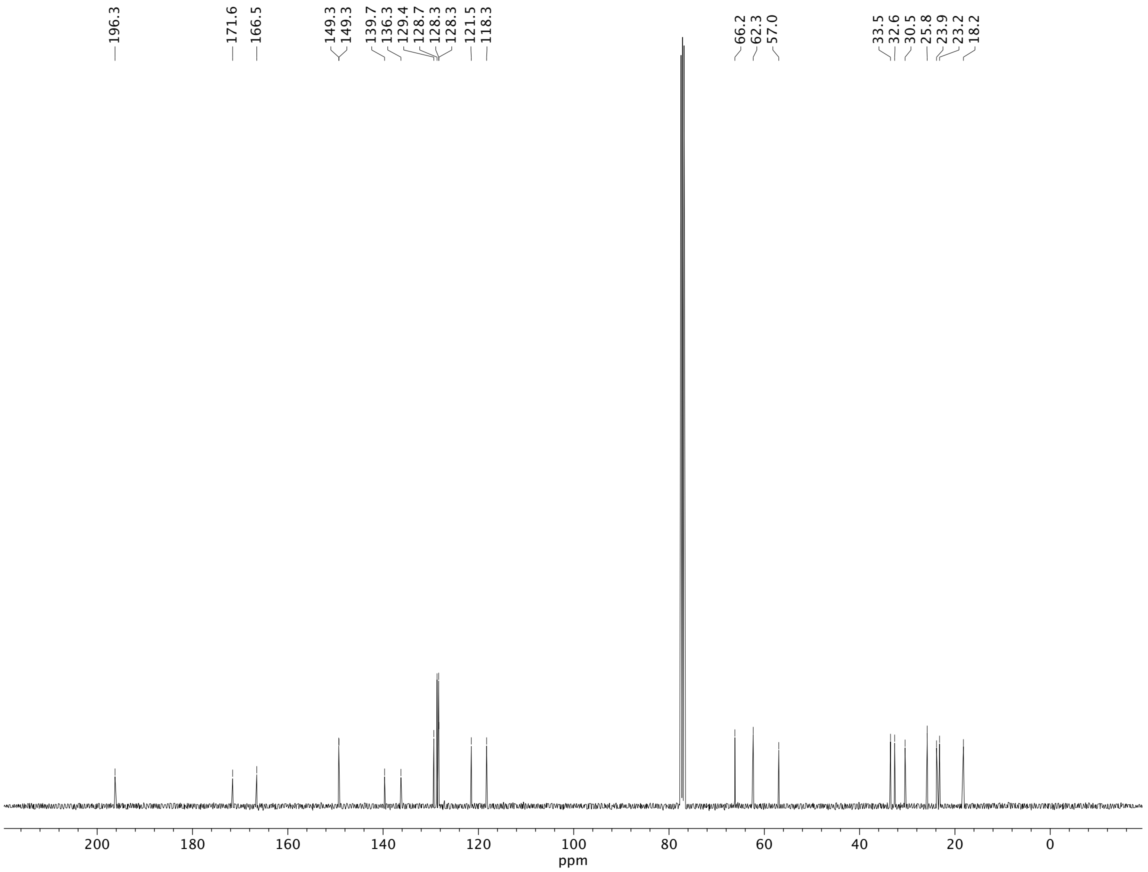

^{13}C NMR (100 MHz, $CDCl_3$) of compound **10a**.

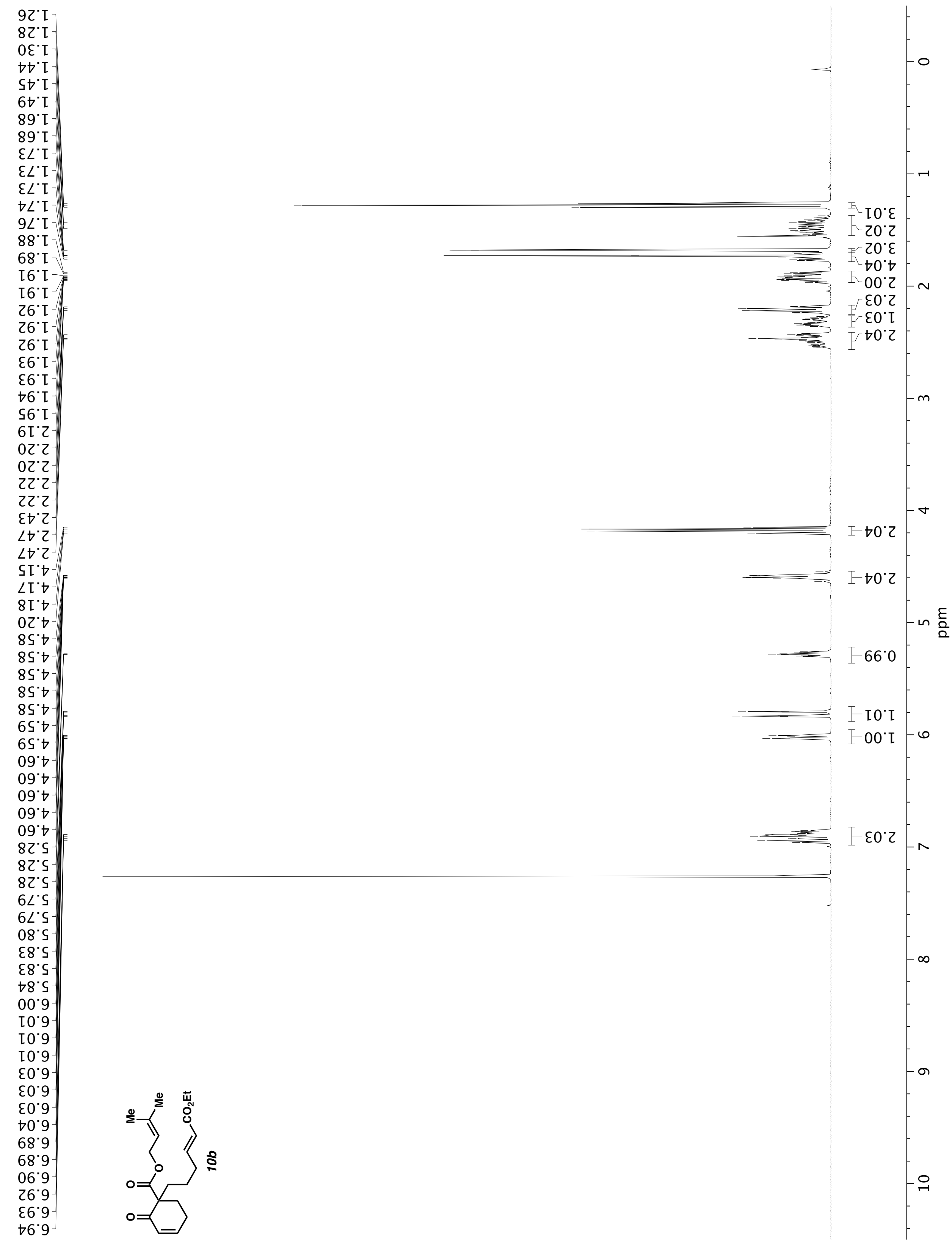

^{1}H NMR (400 MHz, $CDCl_3$) of compound **10b**.

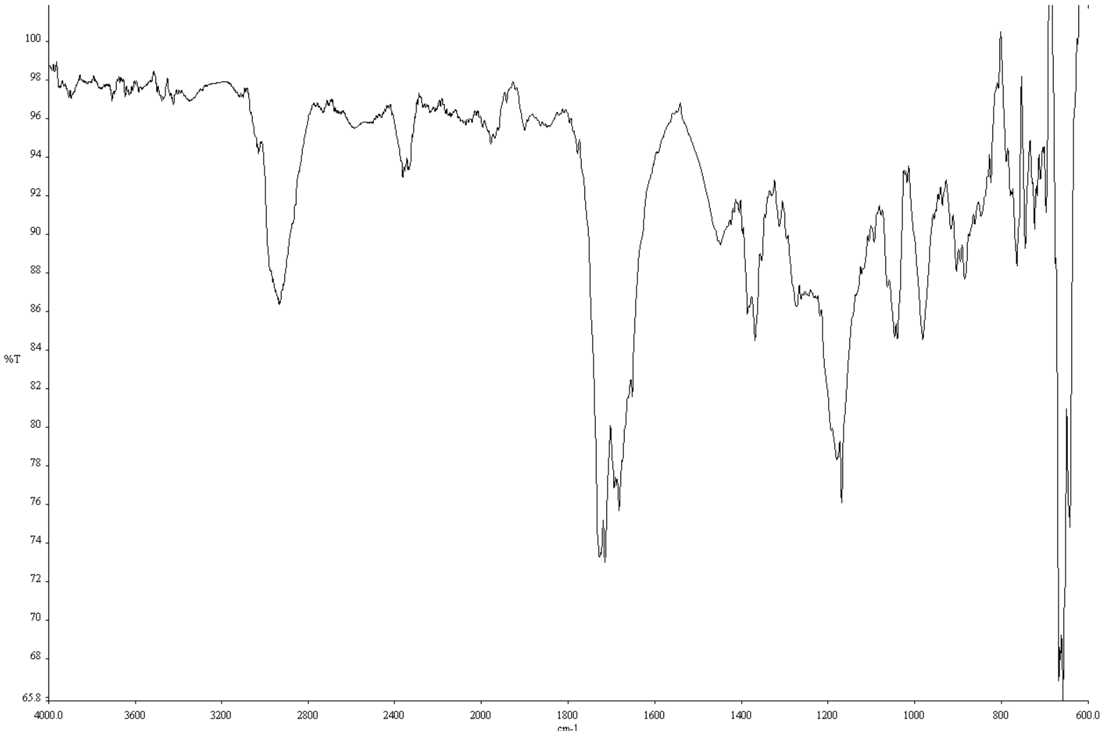

Infrared spectrum (Thin Film, NaCl) of compound **10b**.

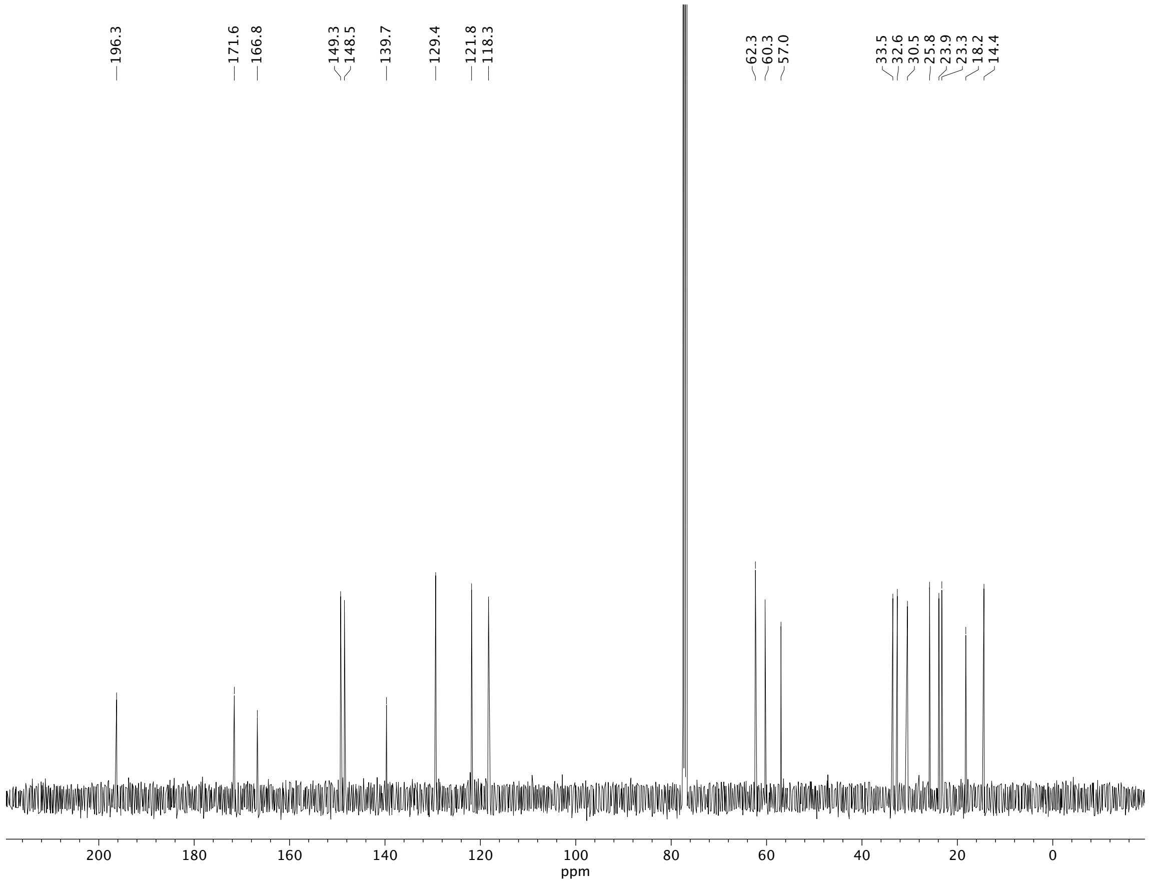

^{13}C NMR (100 MHz, $CDCl_3$) of compound **10b**.

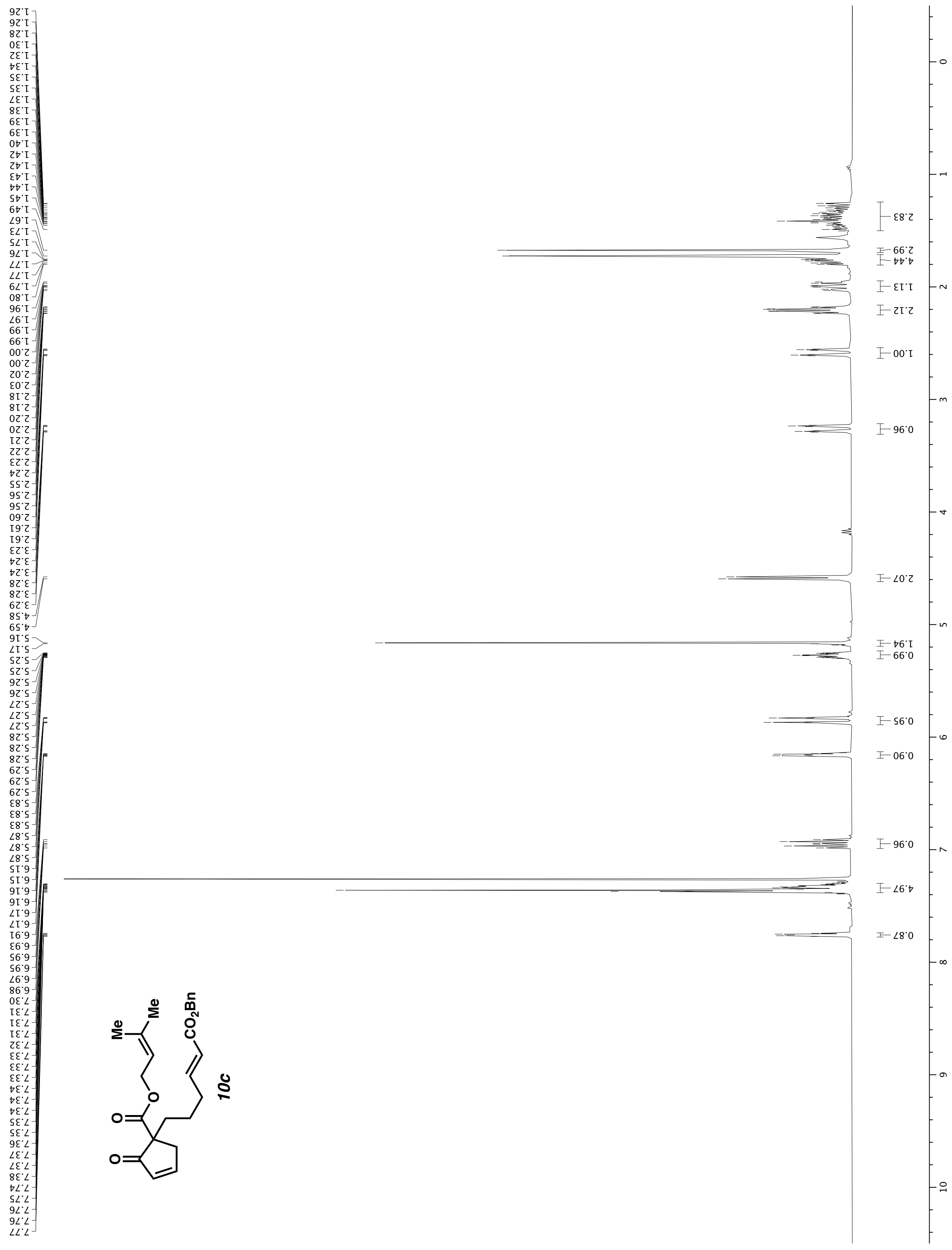

^{1}H NMR (400 MHz, $CDCl_3$) of compound **10c**.

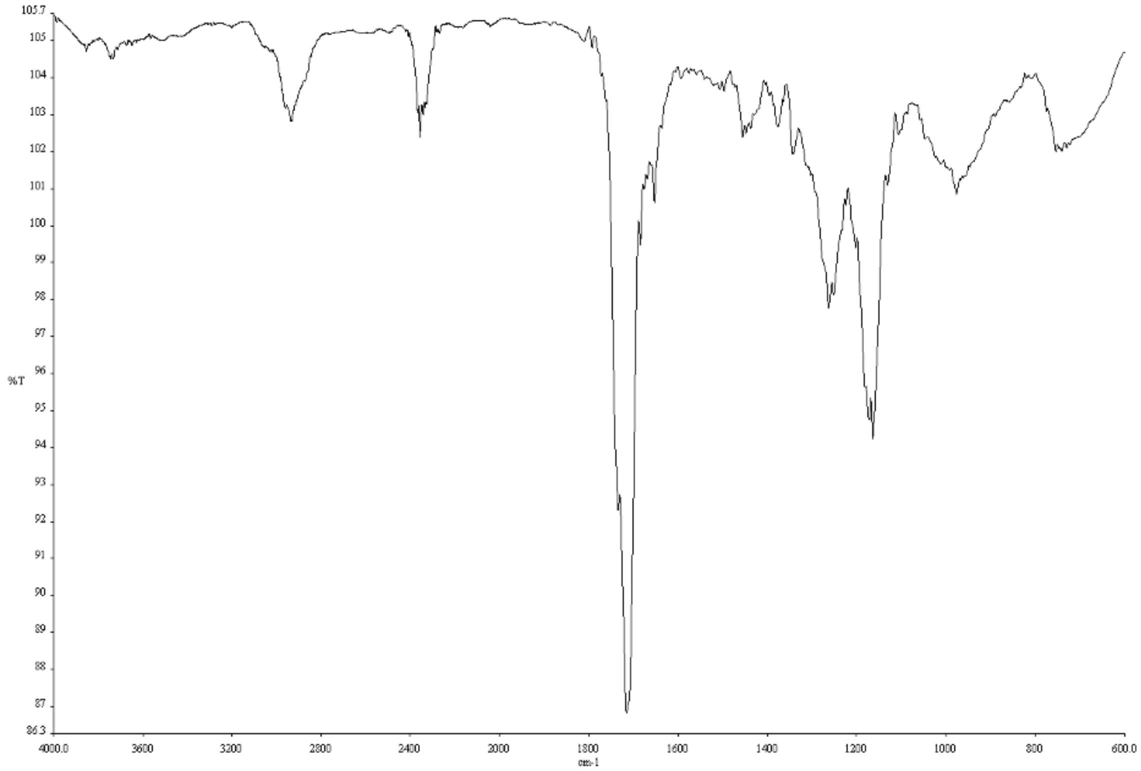

Infrared spectrum (Thin Film, NaCl) of compound **10c**.

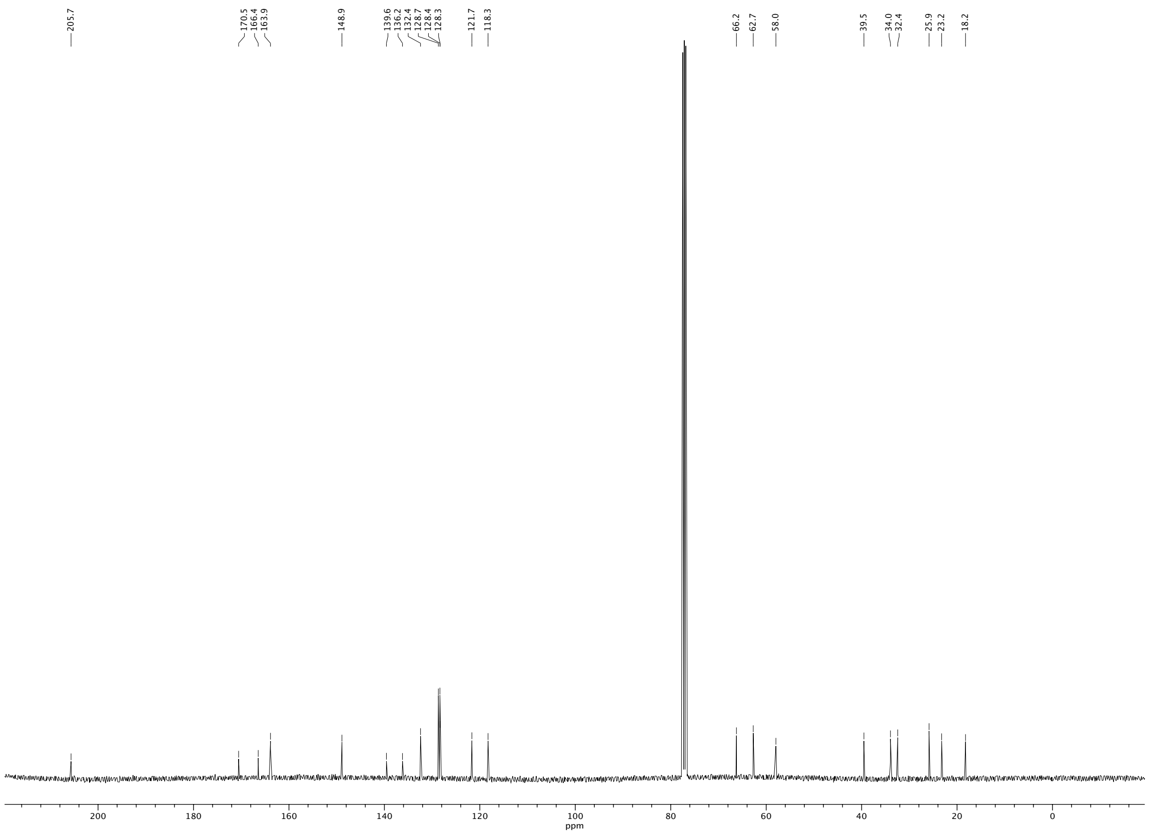

^{13}C NMR (100 MHz, $CDCl_3$) of compound **10c**.

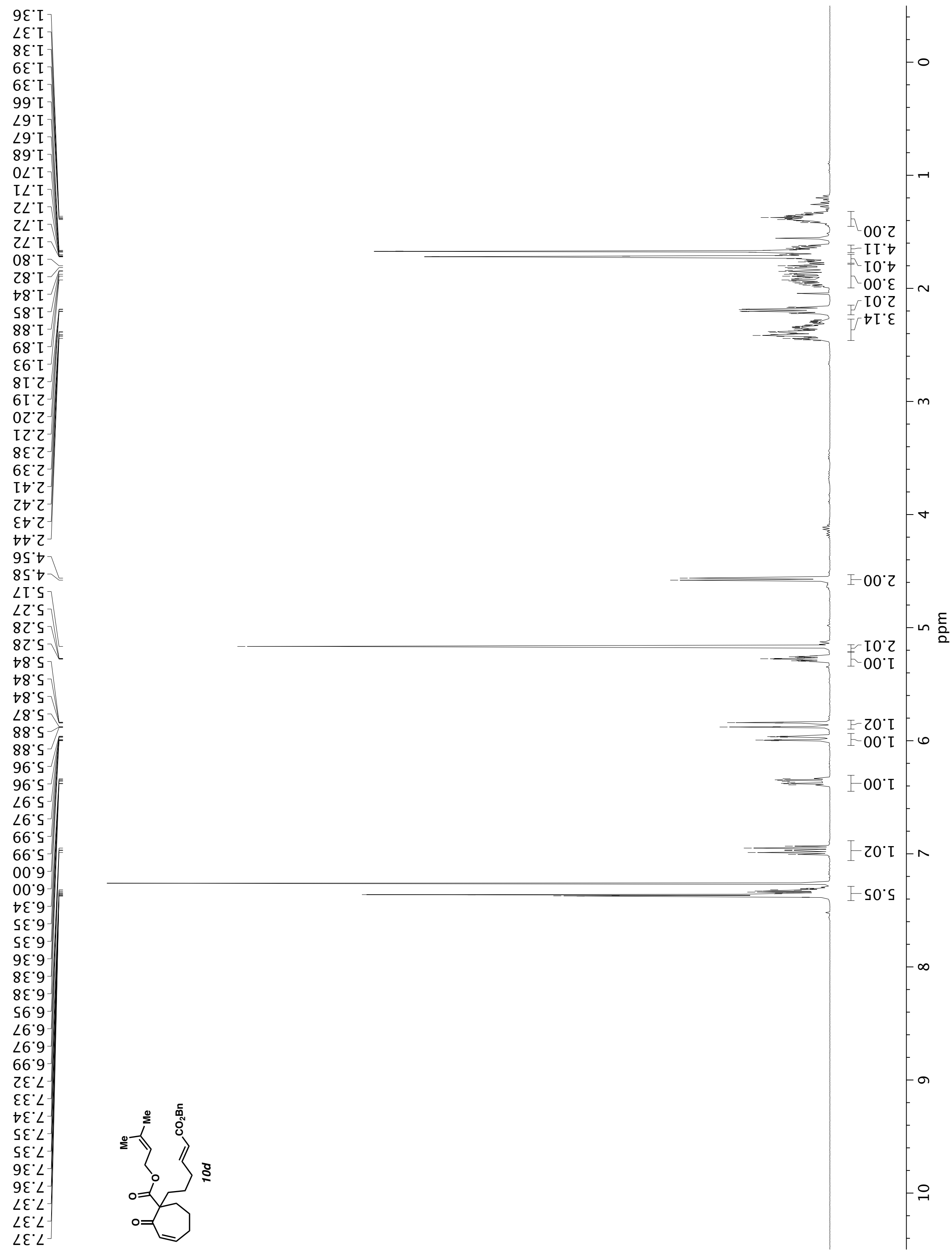

^{1}H NMR (400 MHz, $CDCl_3$) of compound **10d.**

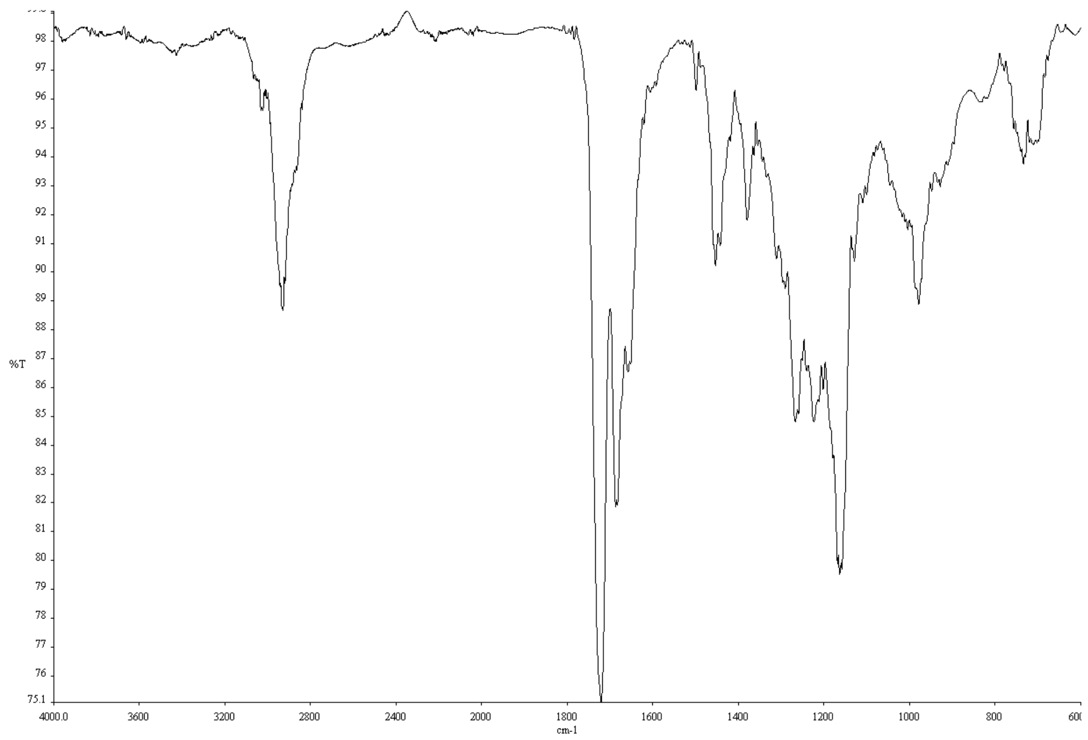

Infrared spectrum (Thin Film, NaCl) of compound **10d**.

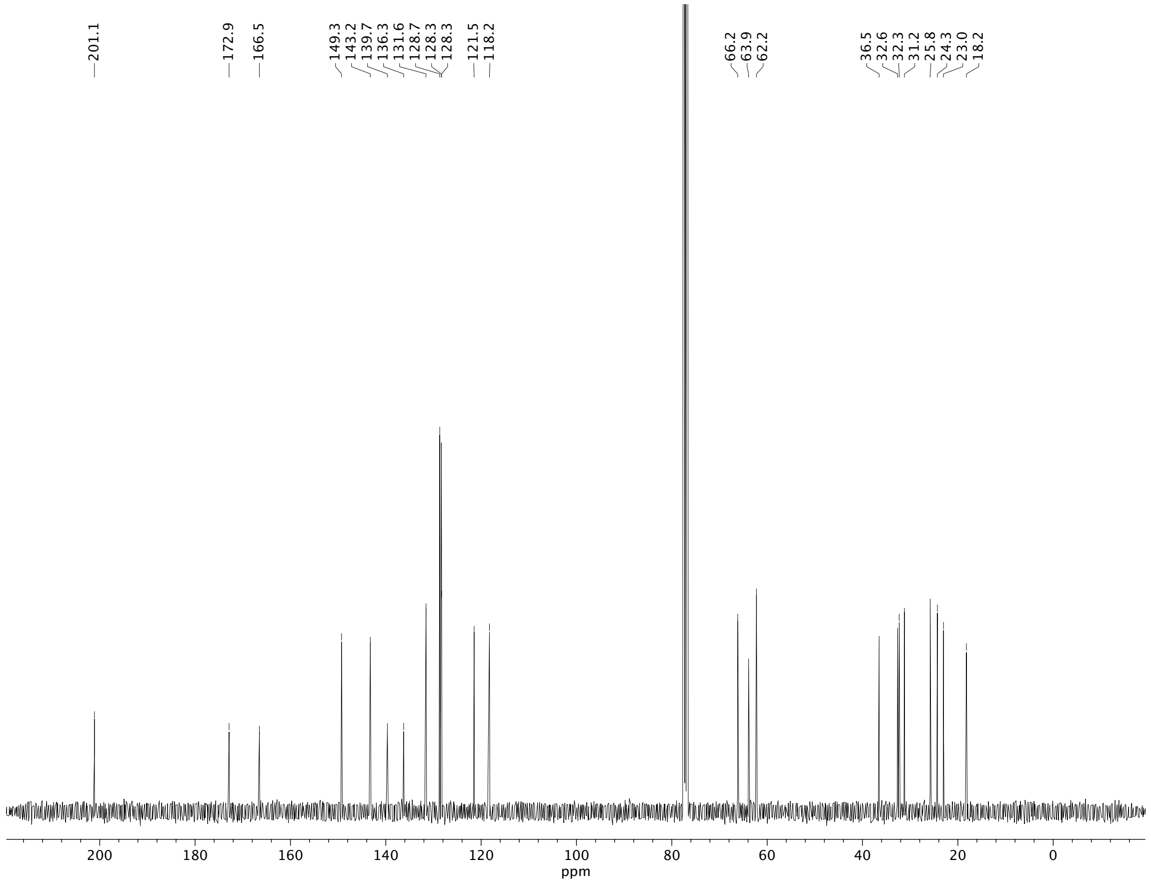

^{13}C NMR (100 MHz, $CDCl_3$) of compound **10d**.

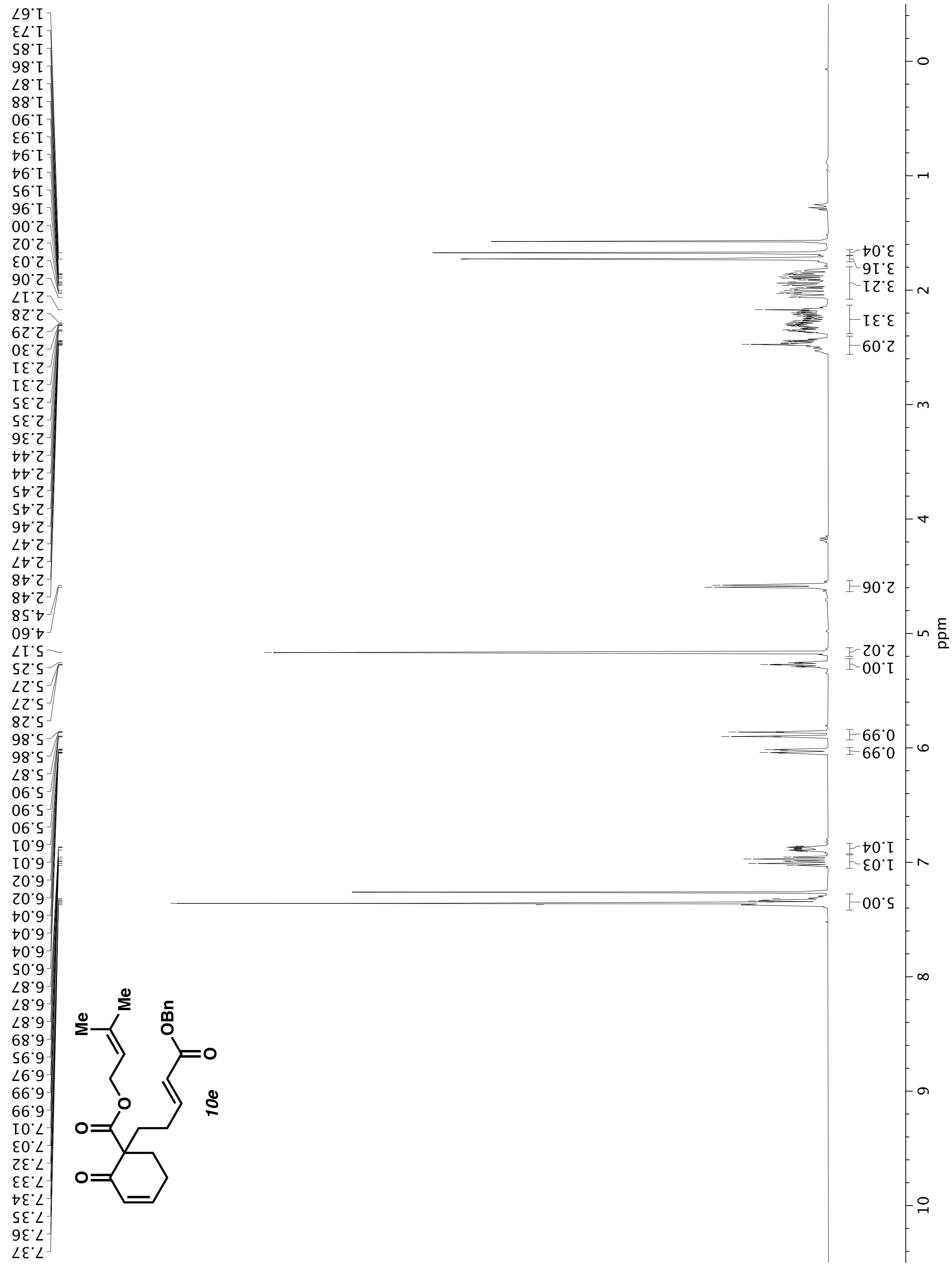

^{1}H NMR (400 MHz, $CDCl_3$) of compound **10e**.

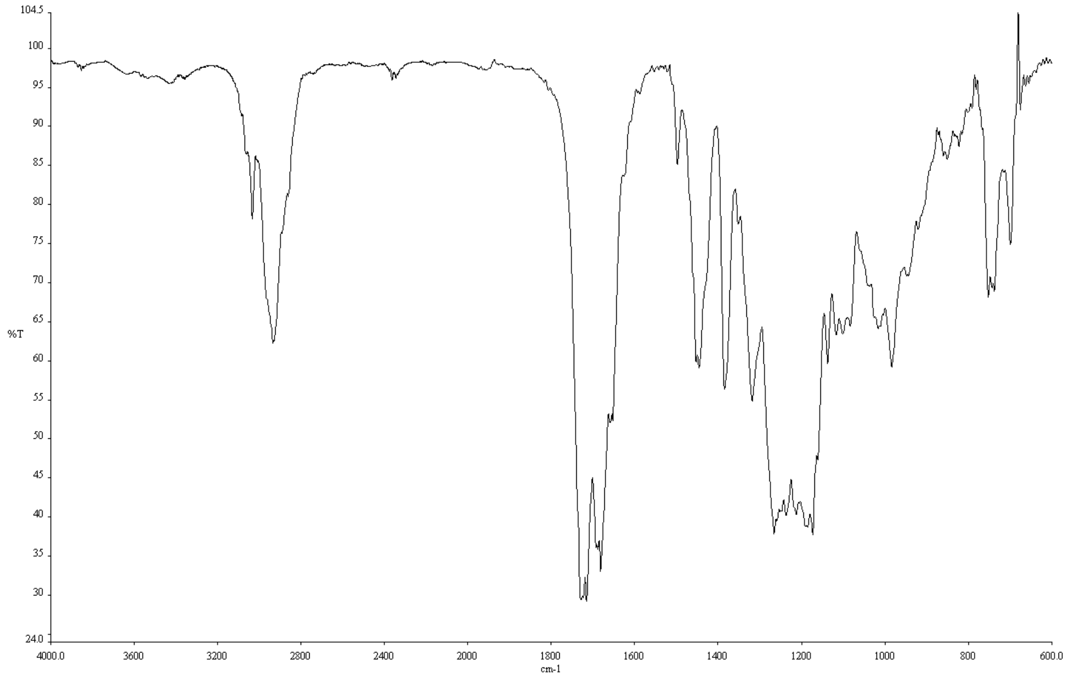

Infrared spectrum (Thin Film, NaCl) of compound **10e**.

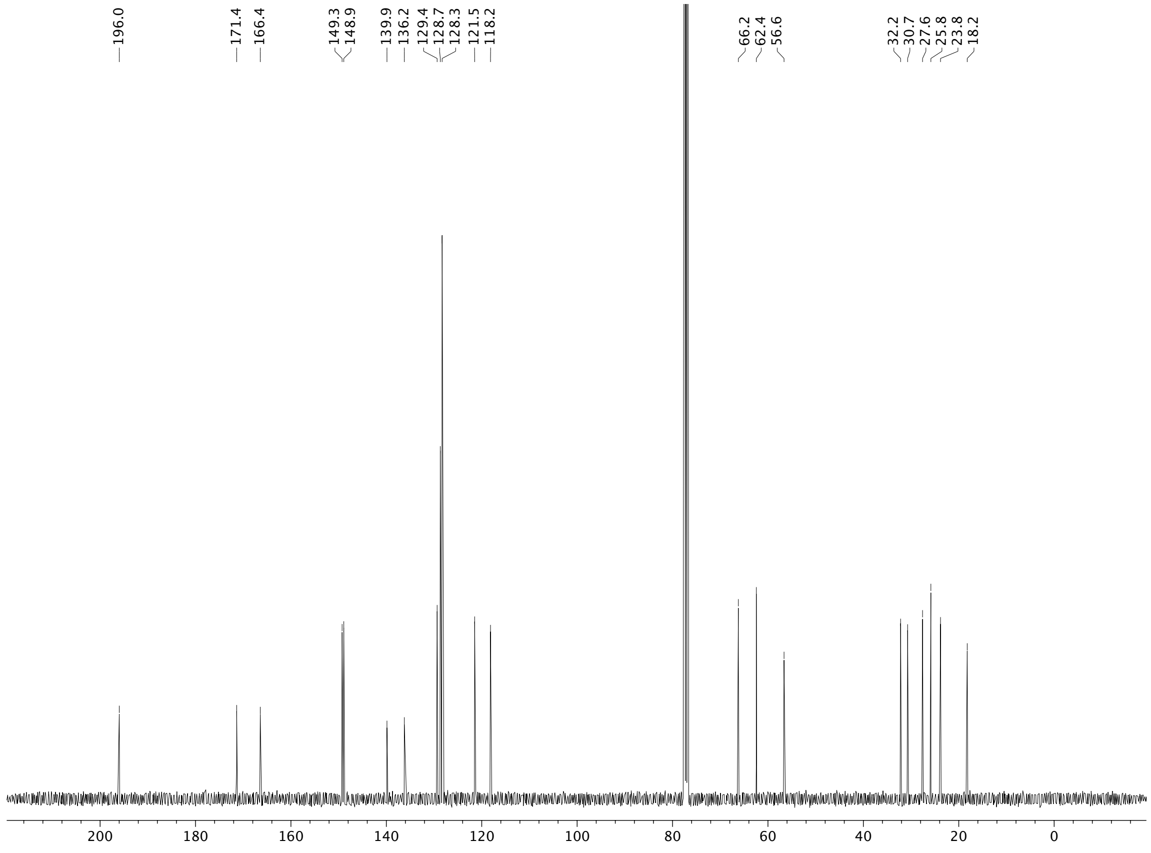

^{13}C NMR (100 MHz, $CDCl_3$) of compound **10e**.

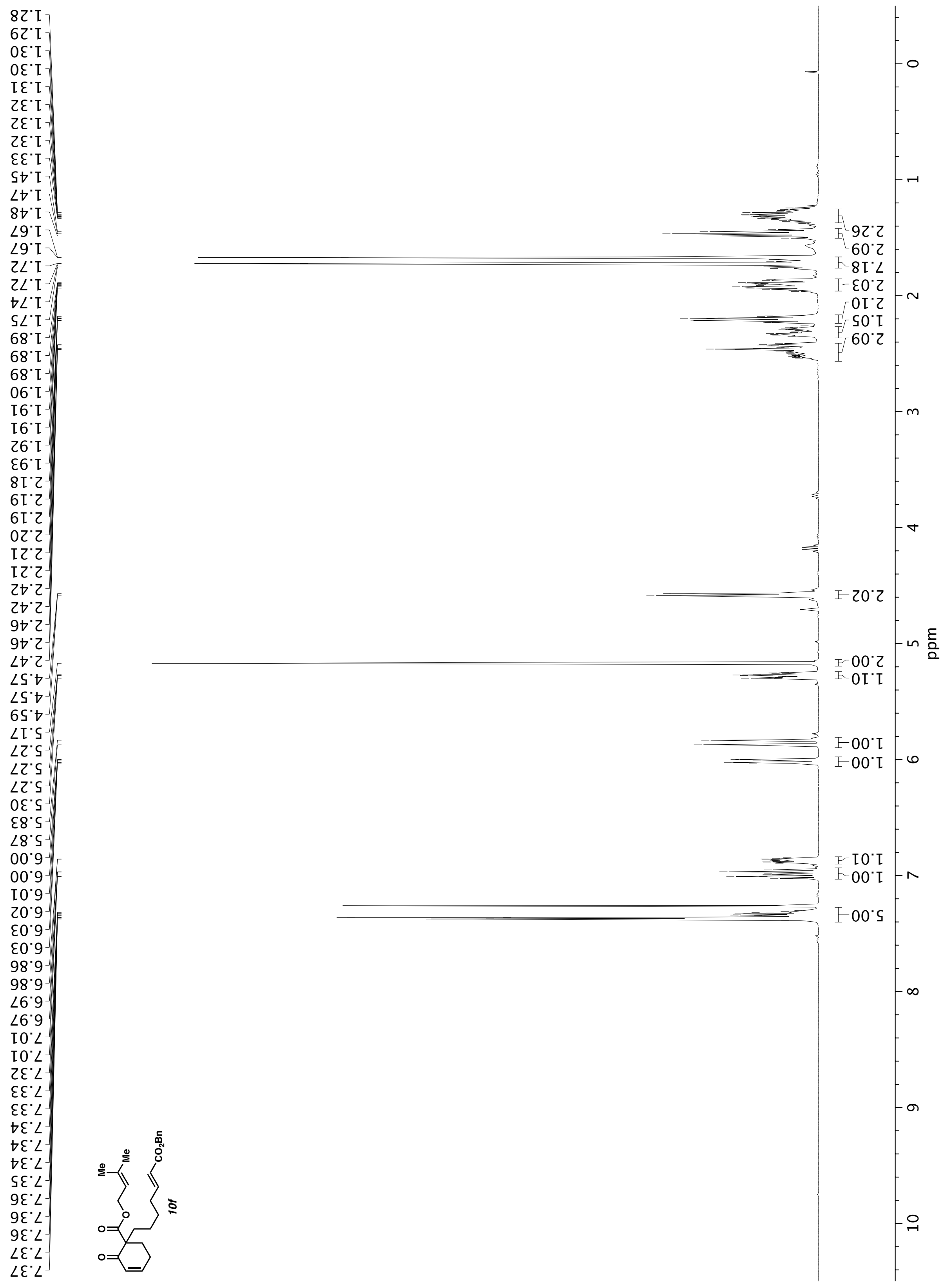

^{1}H NMR (400 MHz, $CDCl_3$) of compound **10f.**

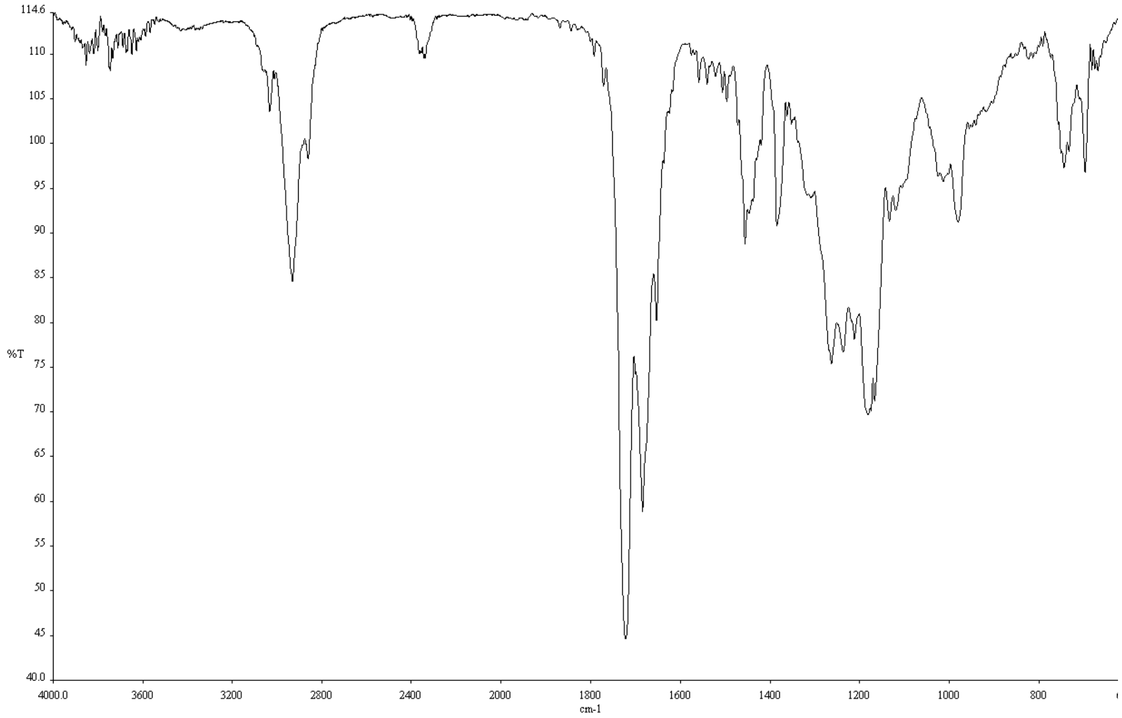

Infrared spectrum (Thin Film, NaCl) of compound **10f**.

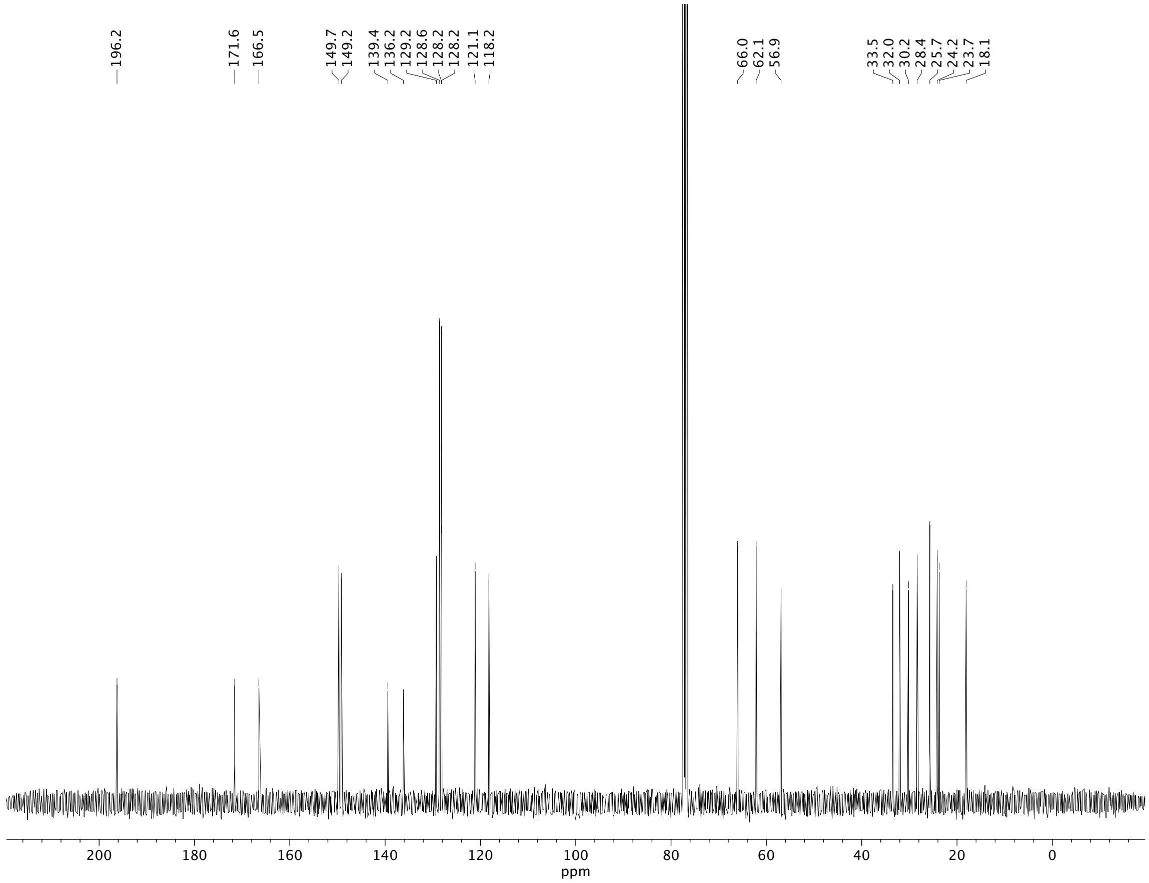

^{13}C NMR (100 MHz, $CDCl_3$) of compound **10f**.

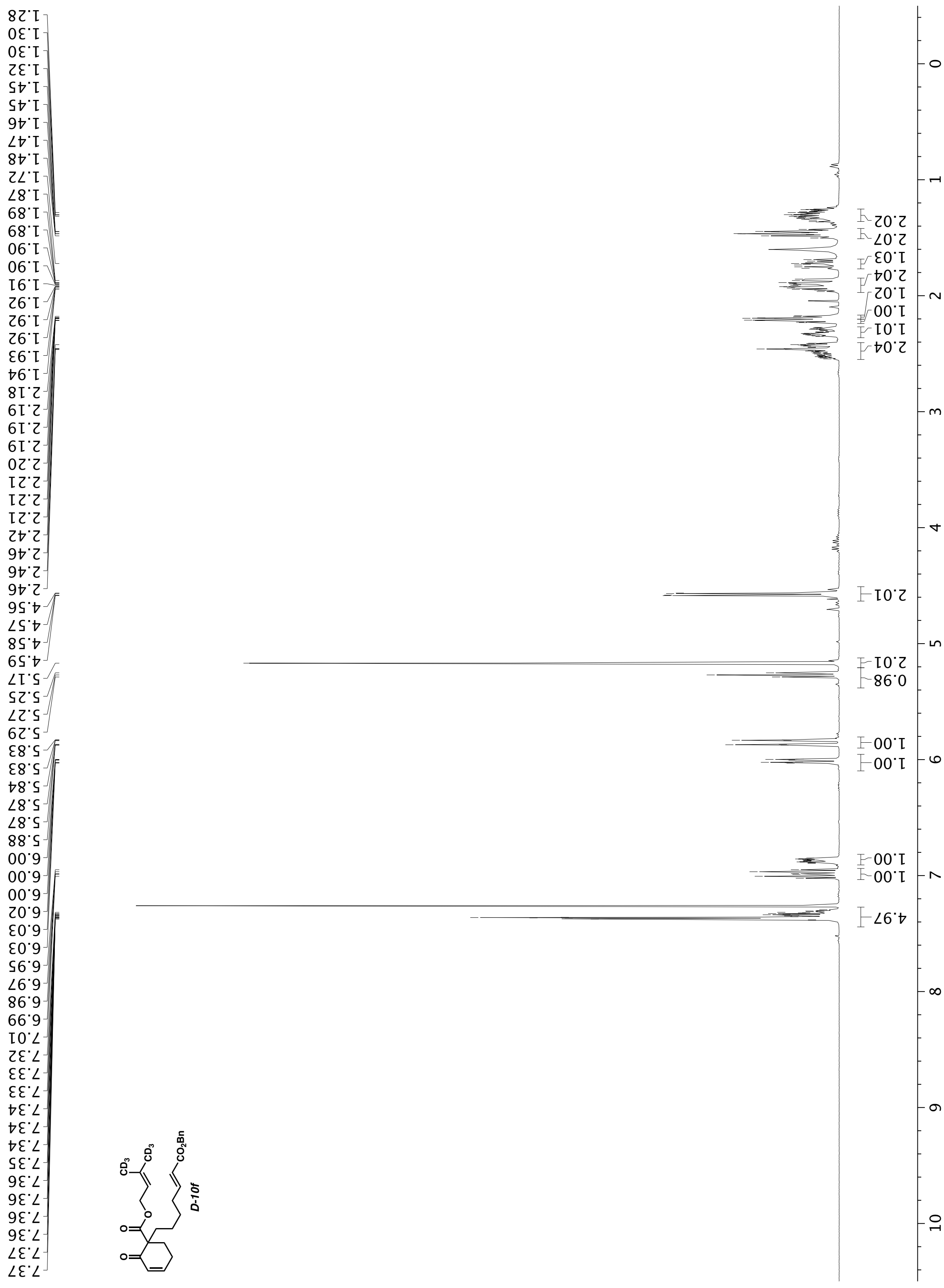

^{1}H NMR (400 MHz, $CDCl_3$) of compound ***D*-10f.**

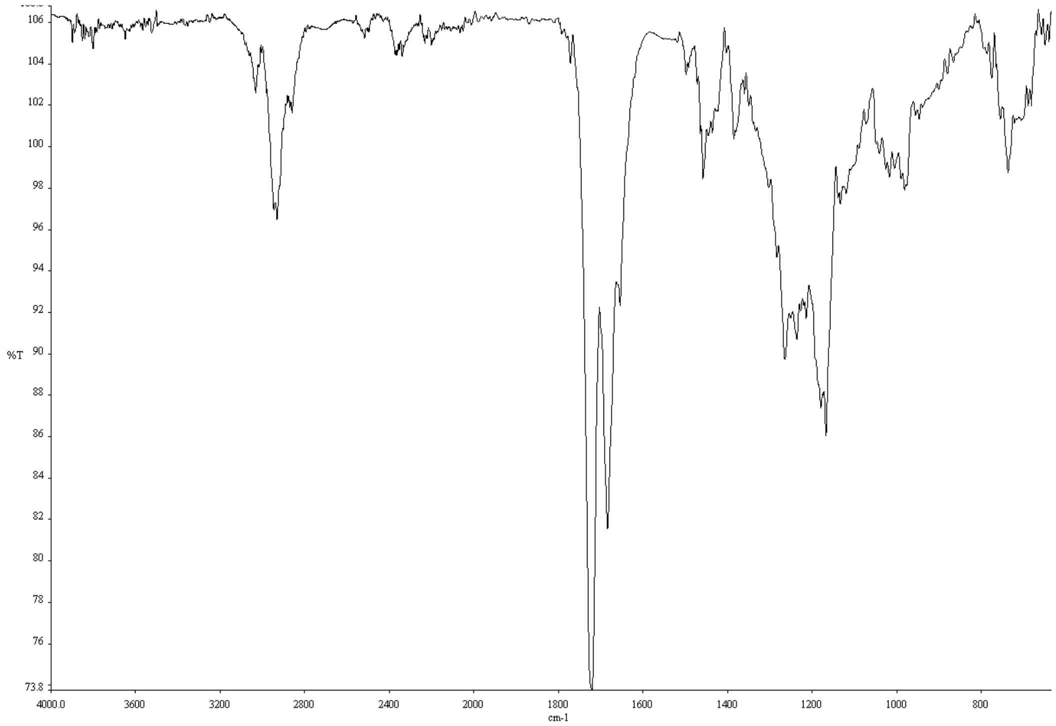

Infrared spectrum (Thin Film, NaCl) of compound ***D*-10f**.

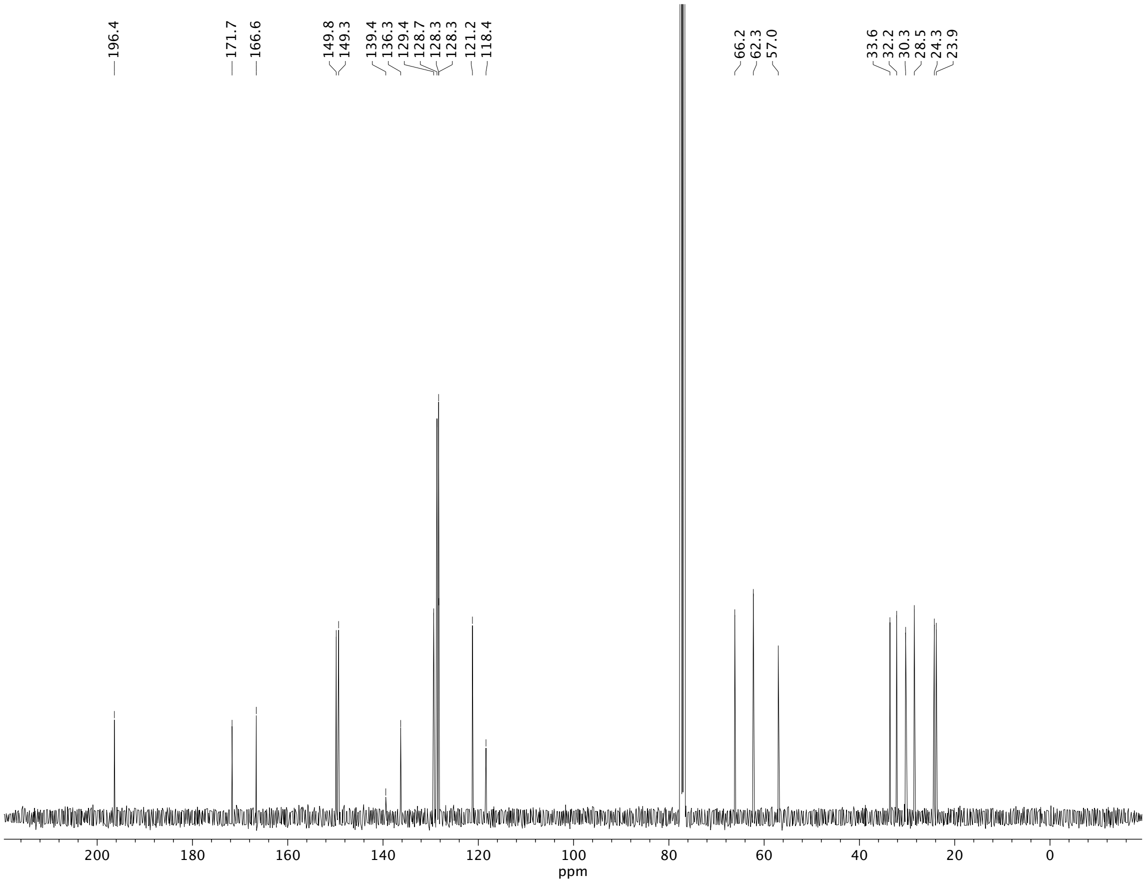

^{13}C NMR (100 MHz, $CDCl_3$) of compound ***D*-10f**.

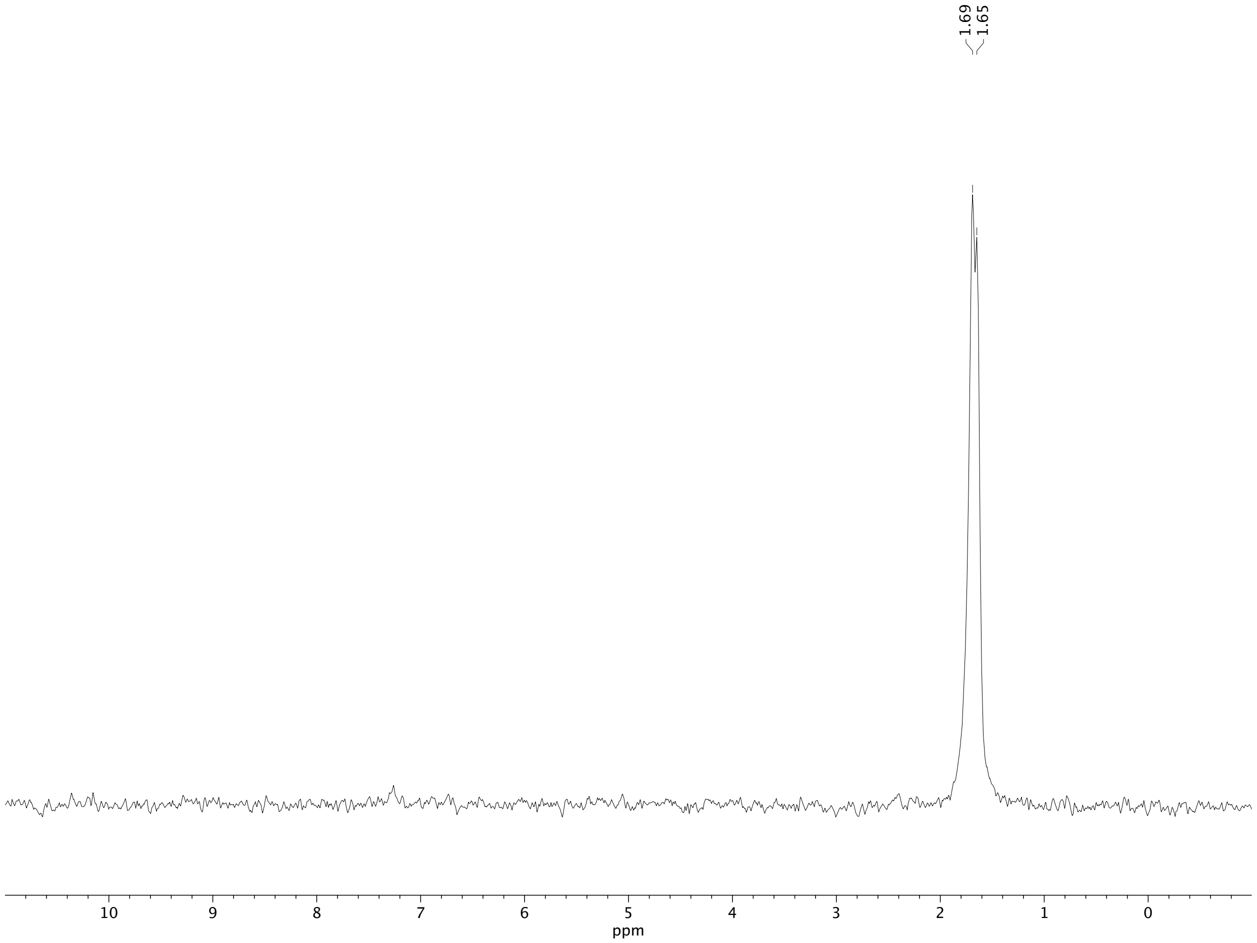

^{2}H NMR (61 MHz, $CHCl_3$) of compound ***D*-10f**.

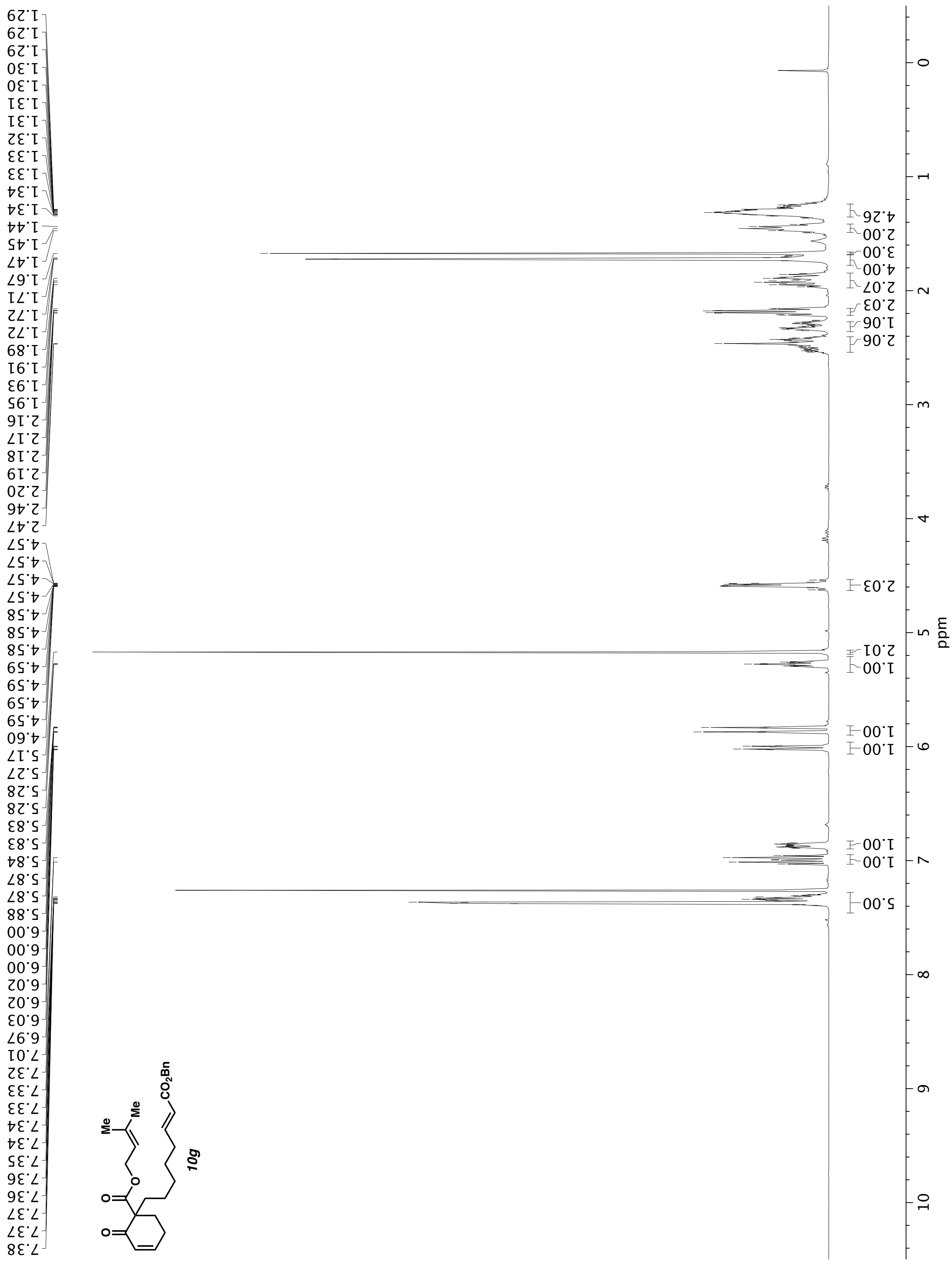

^{1}H NMR (400 MHz, $CDCl_3$) of compound **10g.**

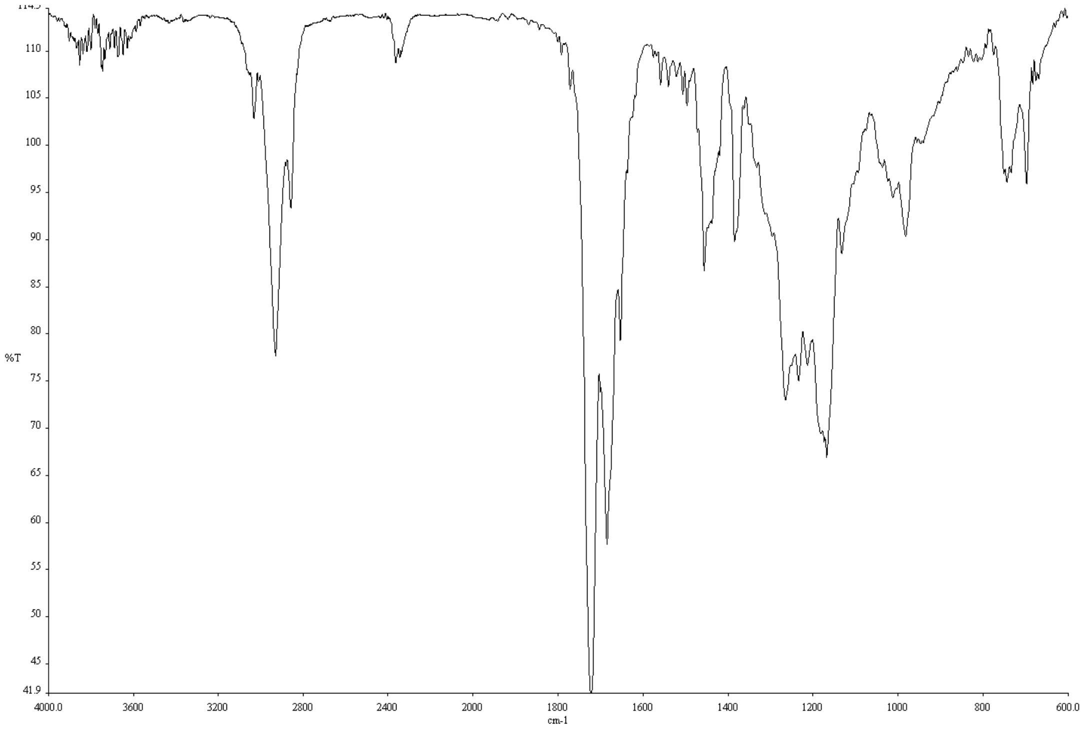

Infrared spectrum (Thin Film, NaCl) of compound **10g**.

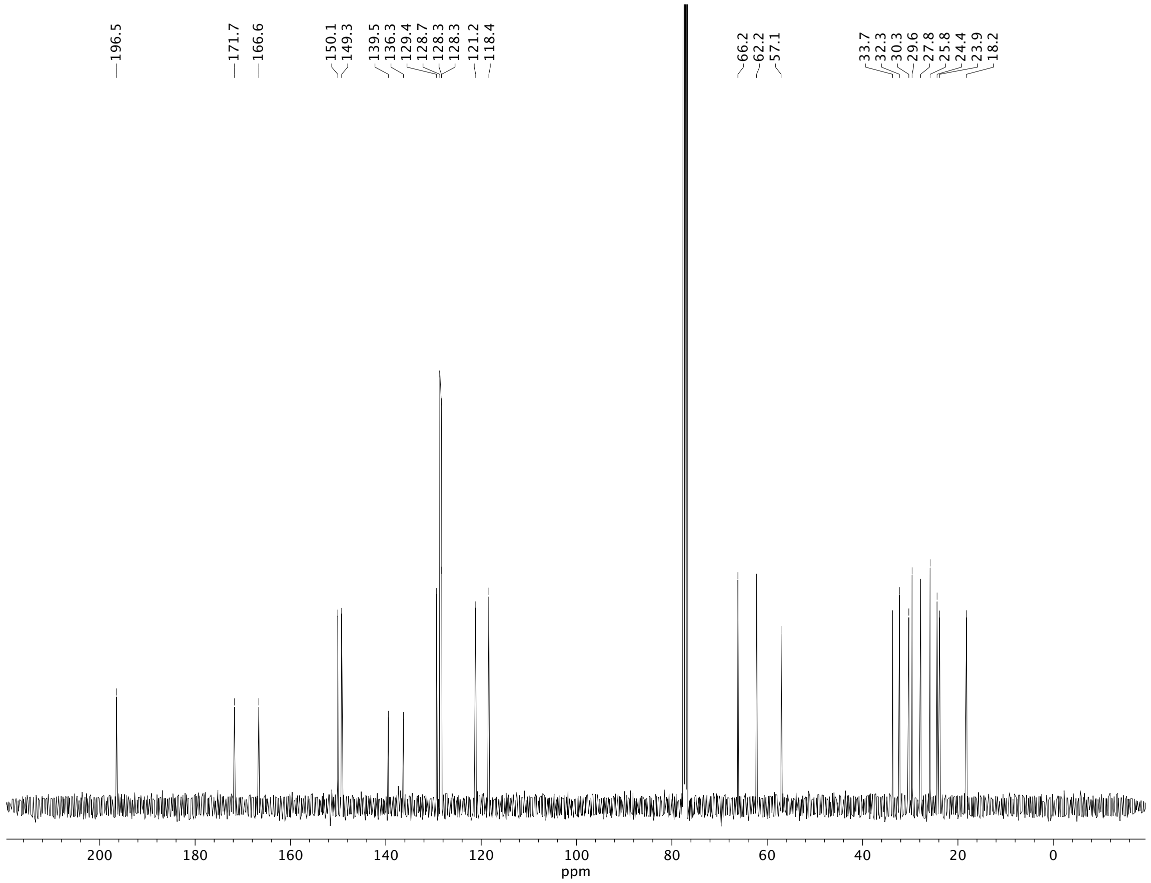

^{13}C NMR (100 MHz, $CDCl_3$) of compound **10g**.

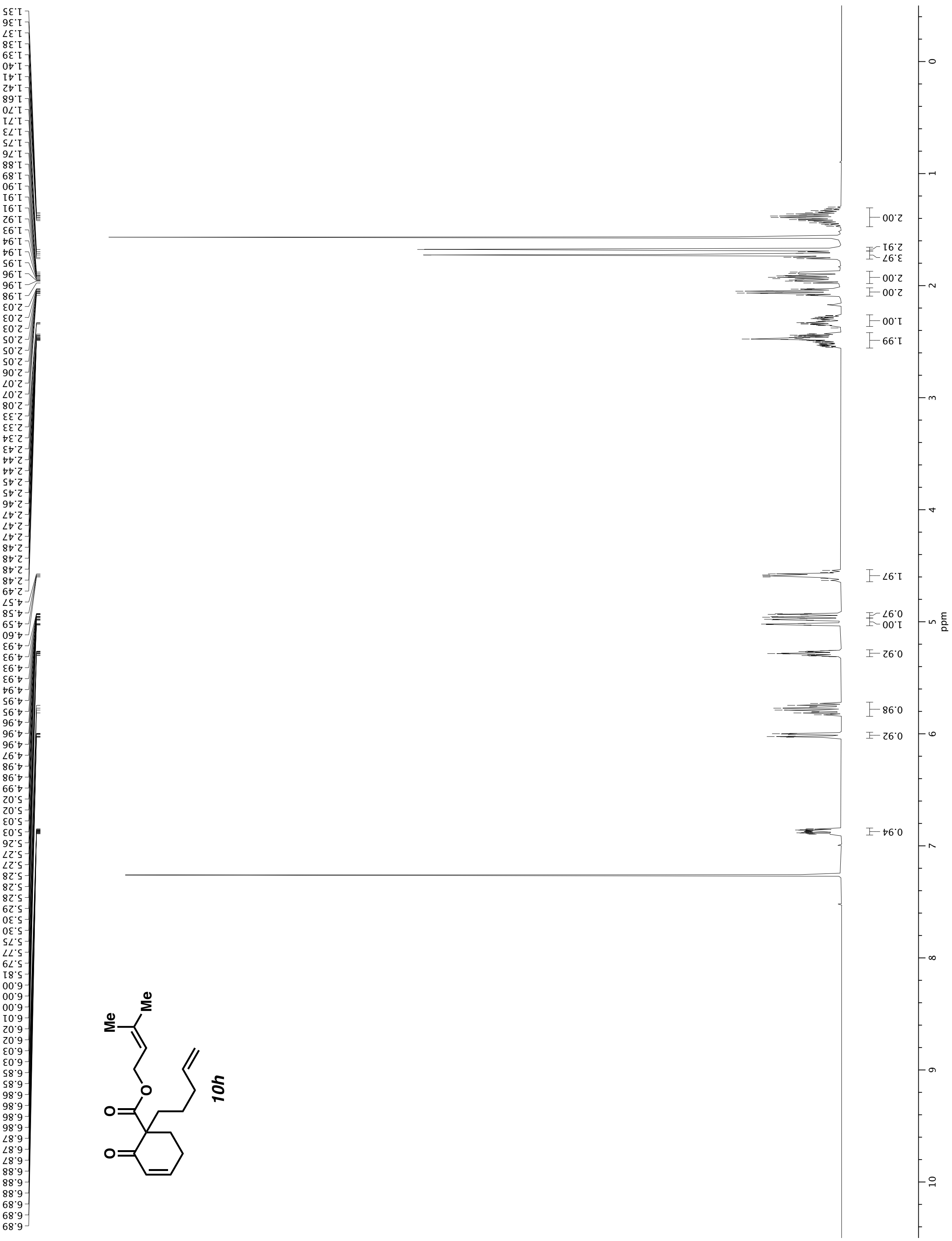

^{1}H NMR (400 MHz, $CDCl_3$) of compound **10h**.

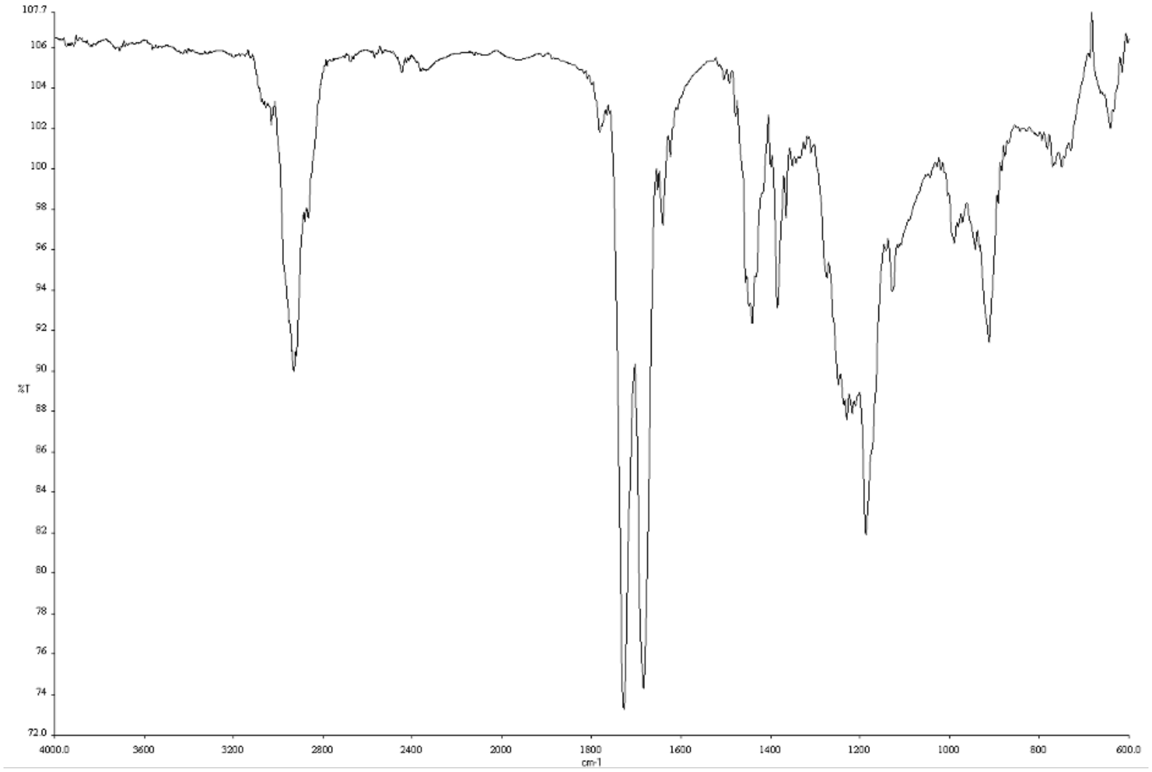

Infrared spectrum (Thin Film, NaCl) of compound **10h**.

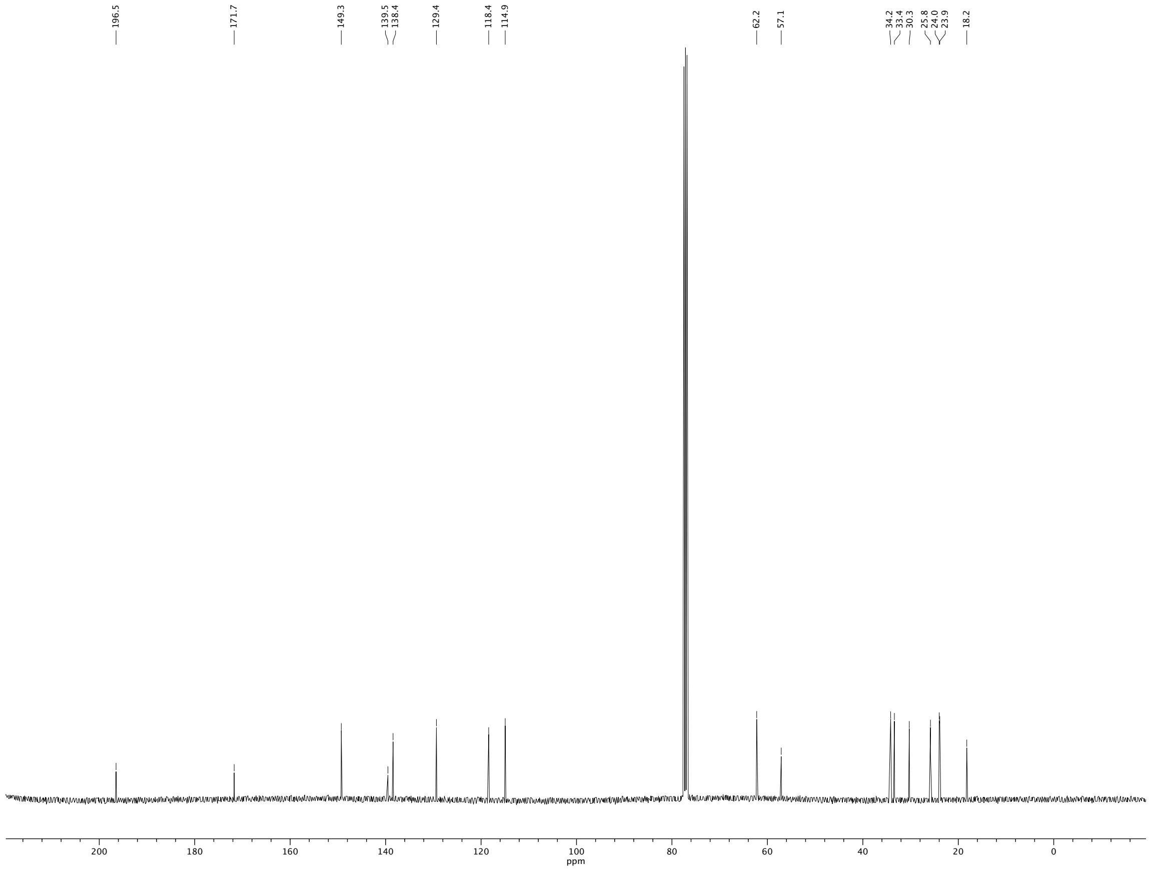

^{13}C NMR (100 MHz, $CDCl_3$) of compound **10h**.

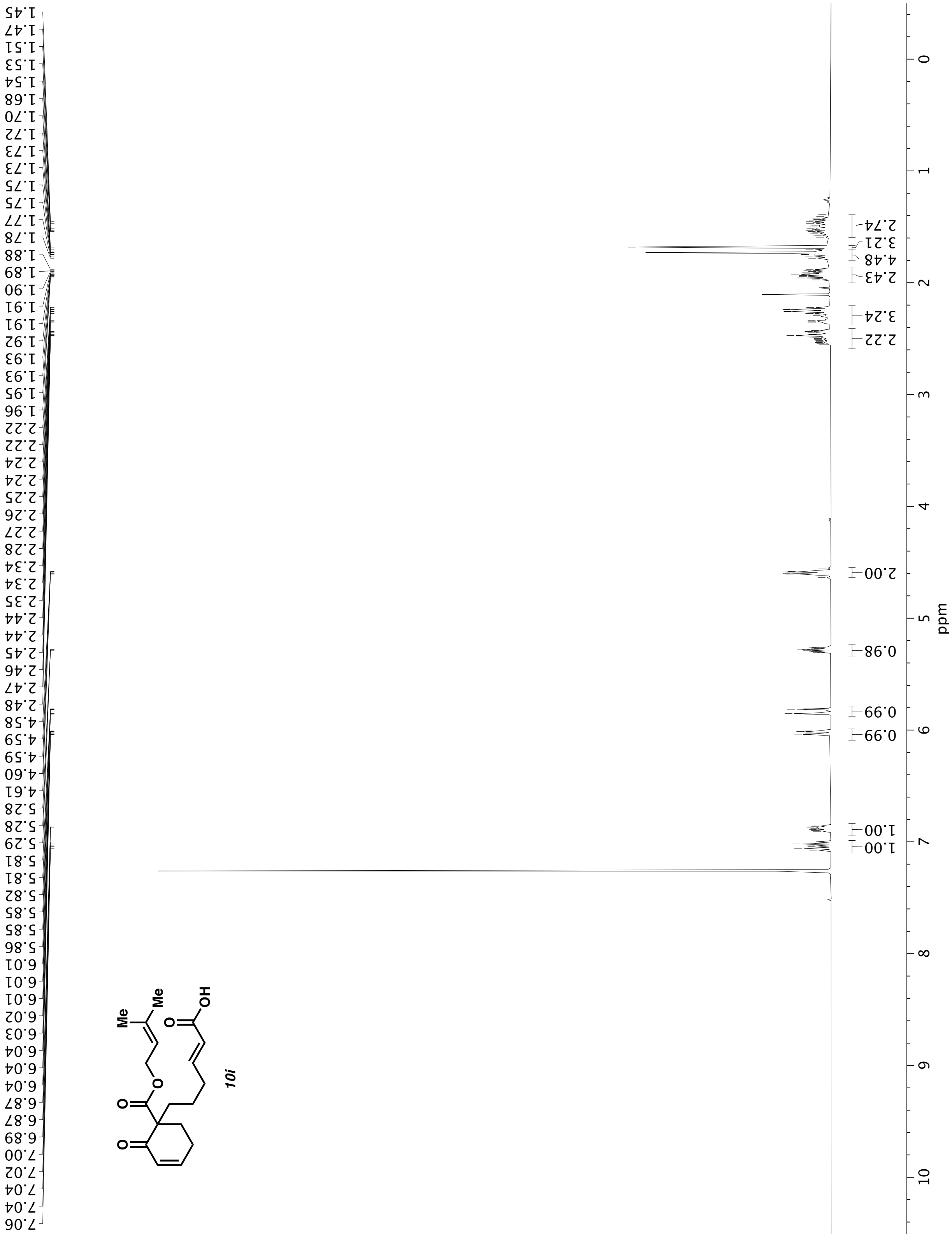

^{1}H NMR (400 MHz, $CDCl_3$) of compound **10i**.

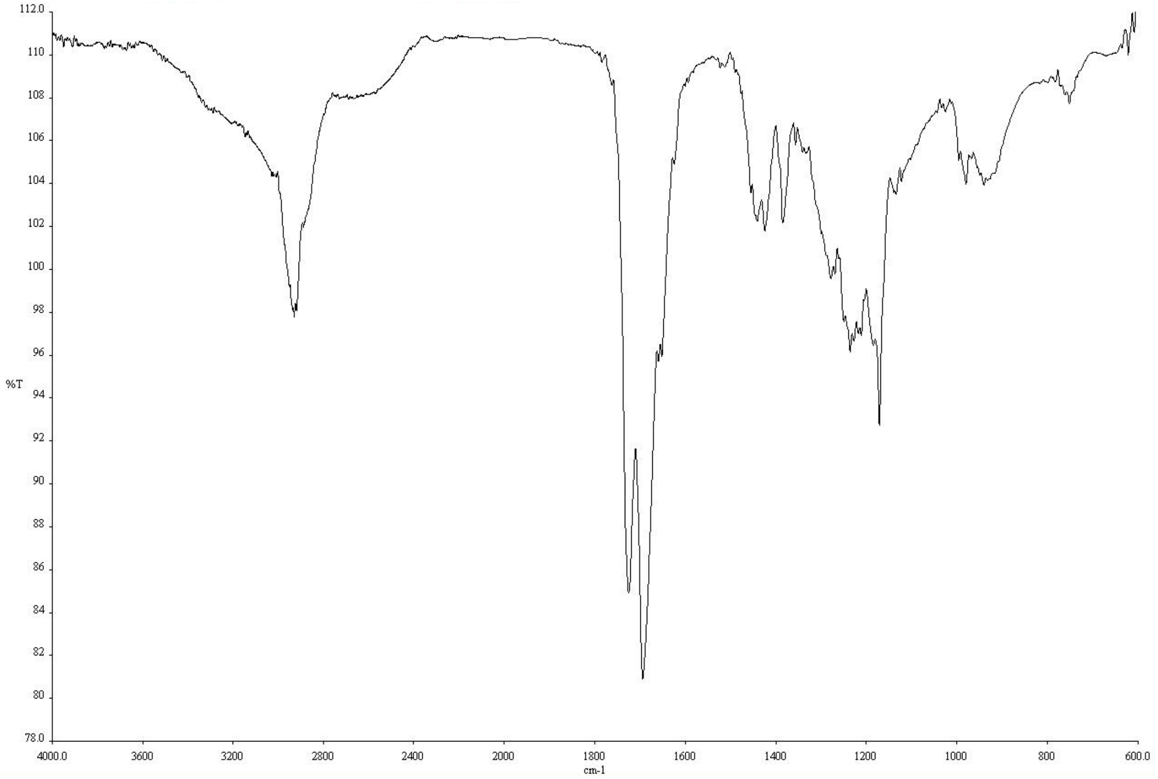

Infrared spectrum (Thin Film, NaCl) of compound **10i**.

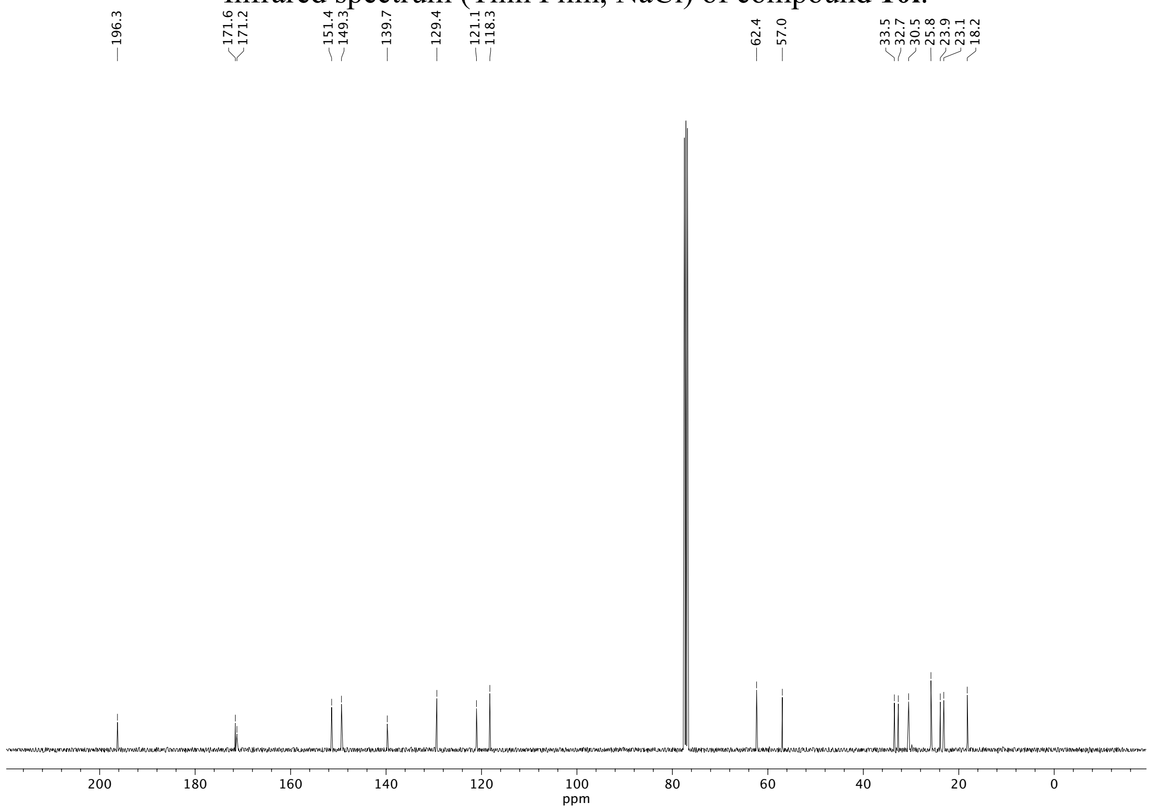

^{13}C NMR (100 MHz, $CDCl_3$) of compound **10i**.

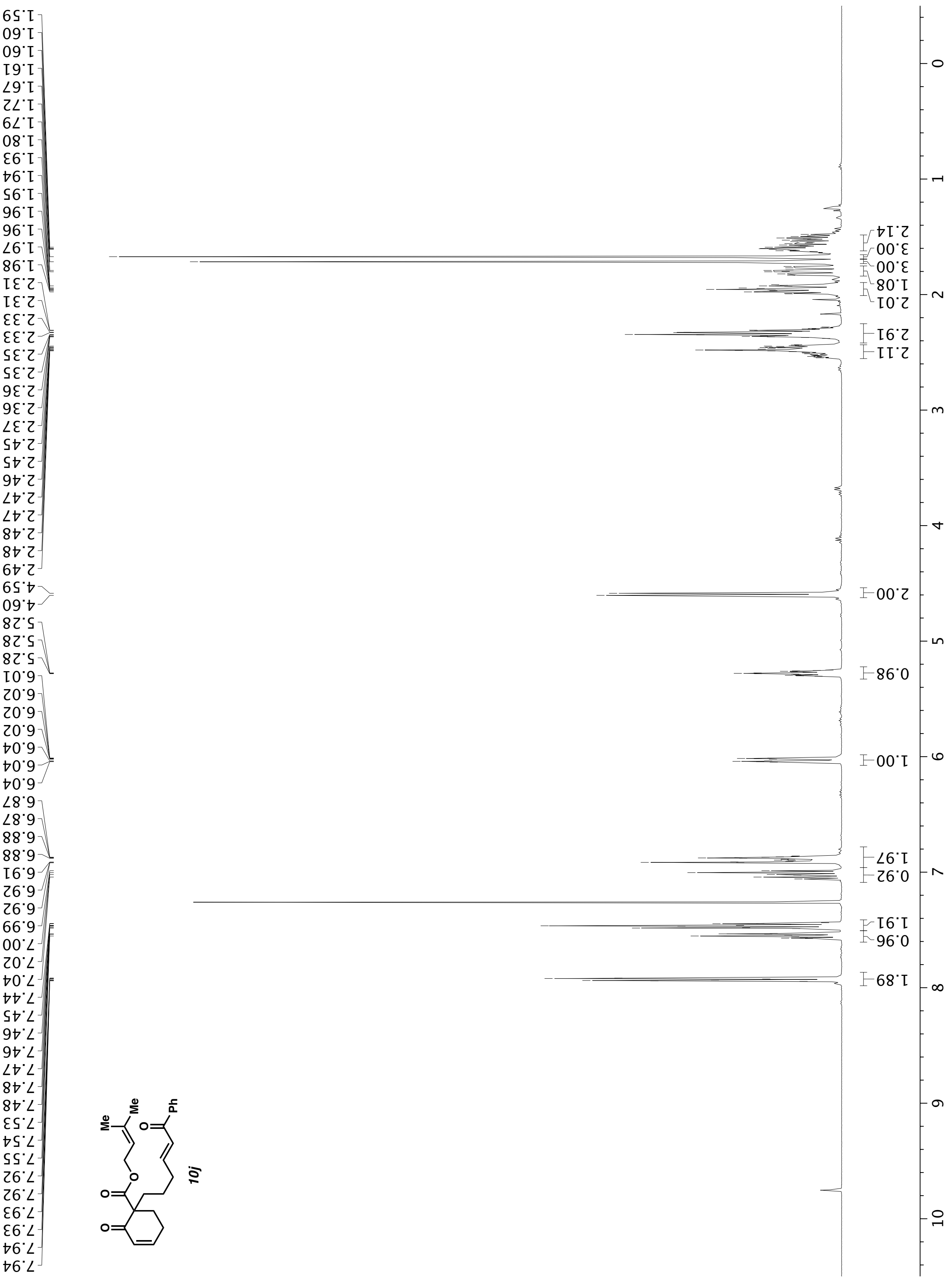

^{1}H NMR (400 MHz, $CDCl_3$) of compound **10j.**

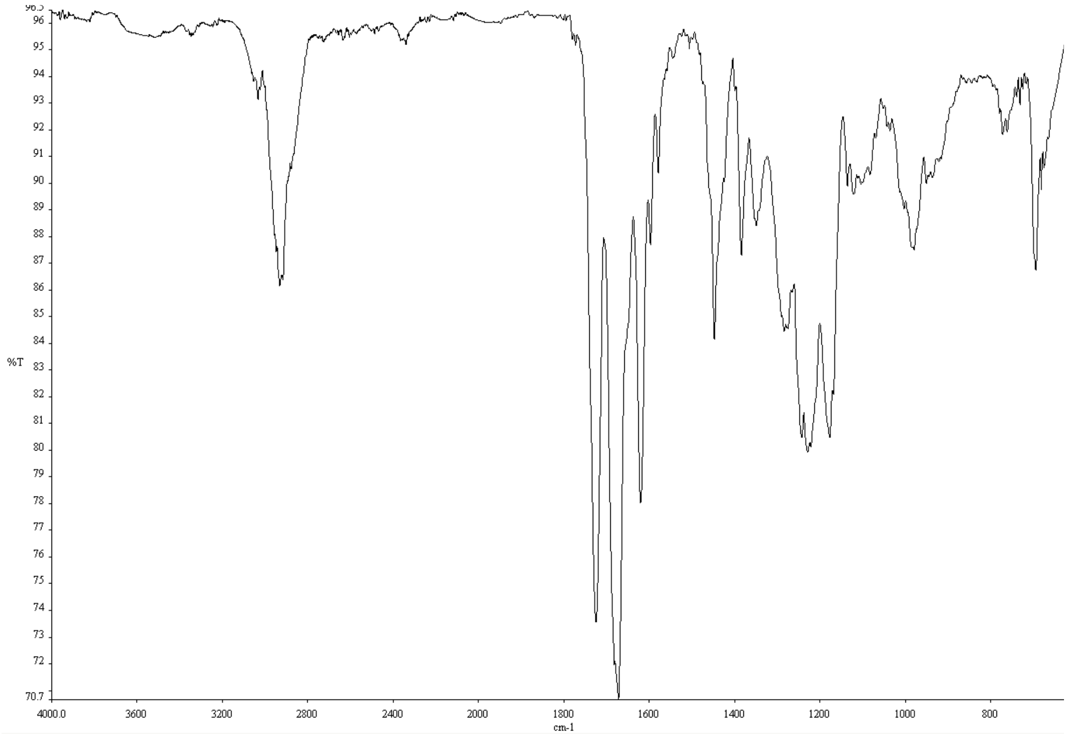

Infrared spectrum (Thin Film, NaCl) of compound **10j**.

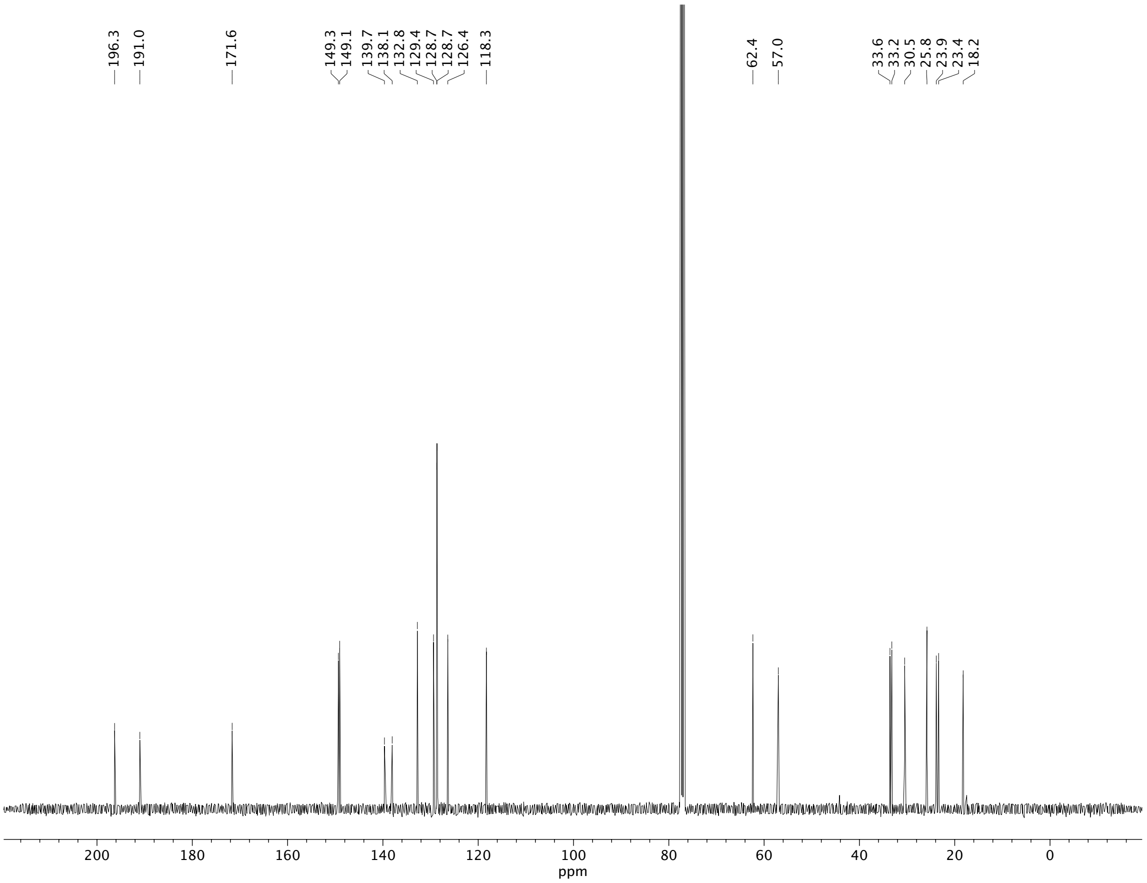

^{13}C NMR (100 MHz, CDCl$_3$) of compound **10j**.

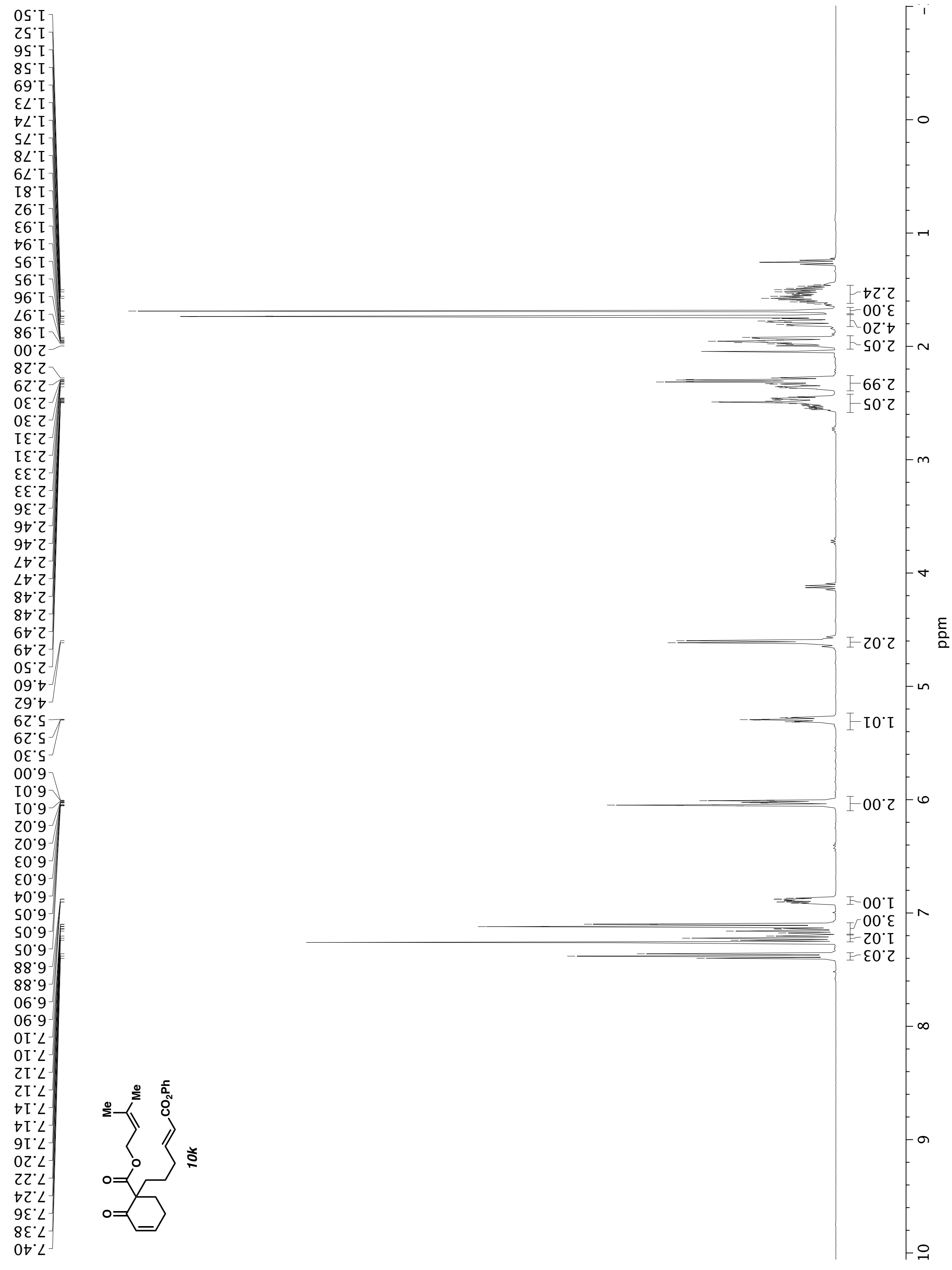

^{1}H NMR (400 MHz, $CDCl_3$) of compound **10k.**

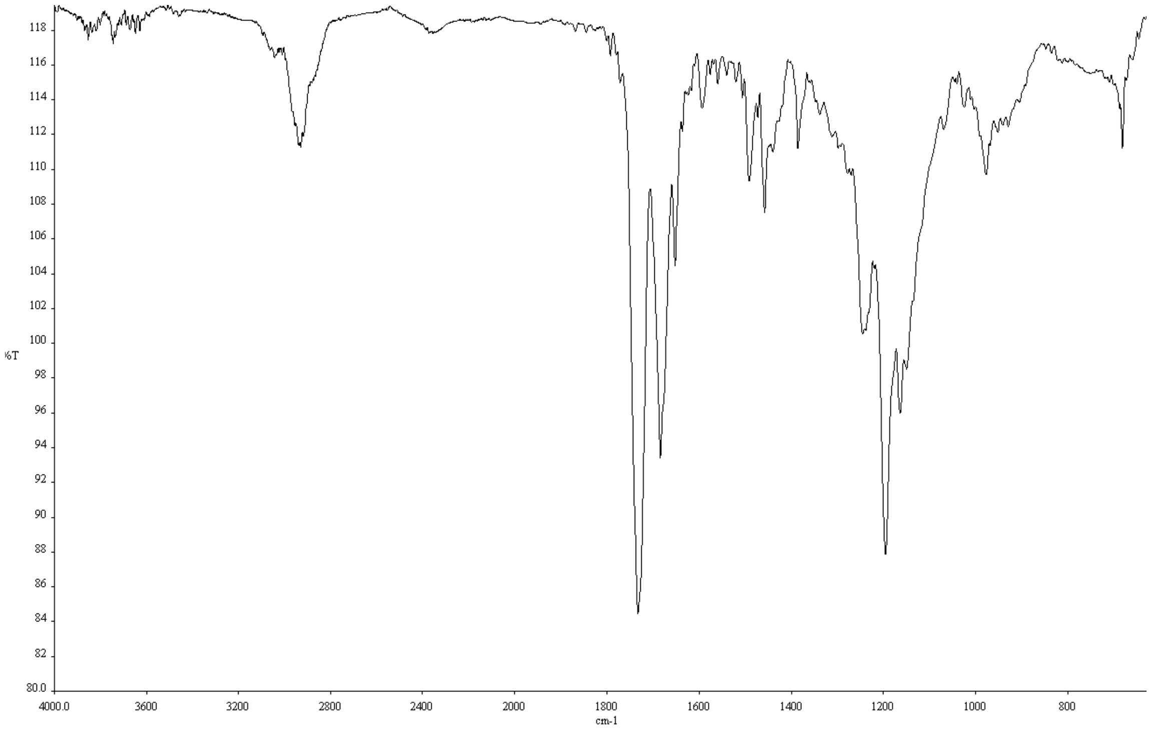

Infrared spectrum (Thin Film, NaCl) of compound **10k**.

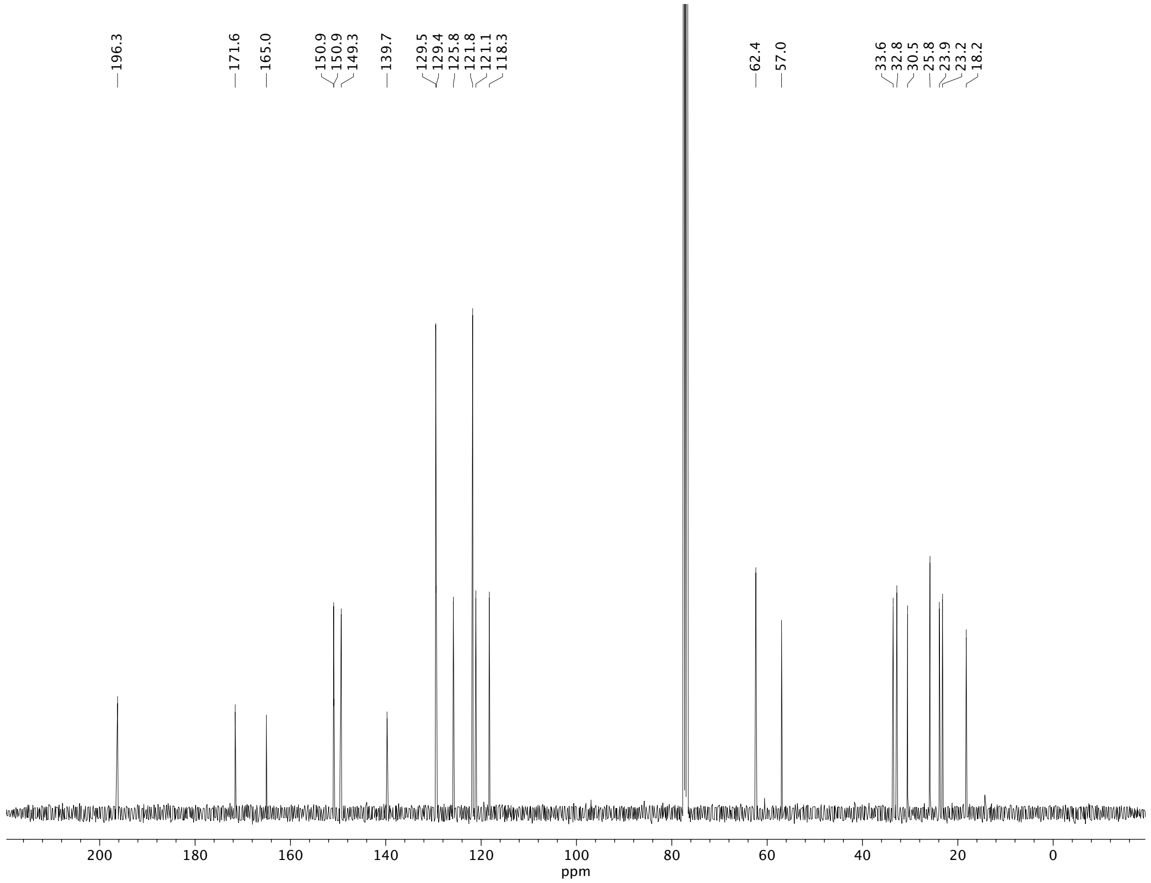

^{13}C NMR (100 MHz, $CDCl_3$) of compound **10k**.

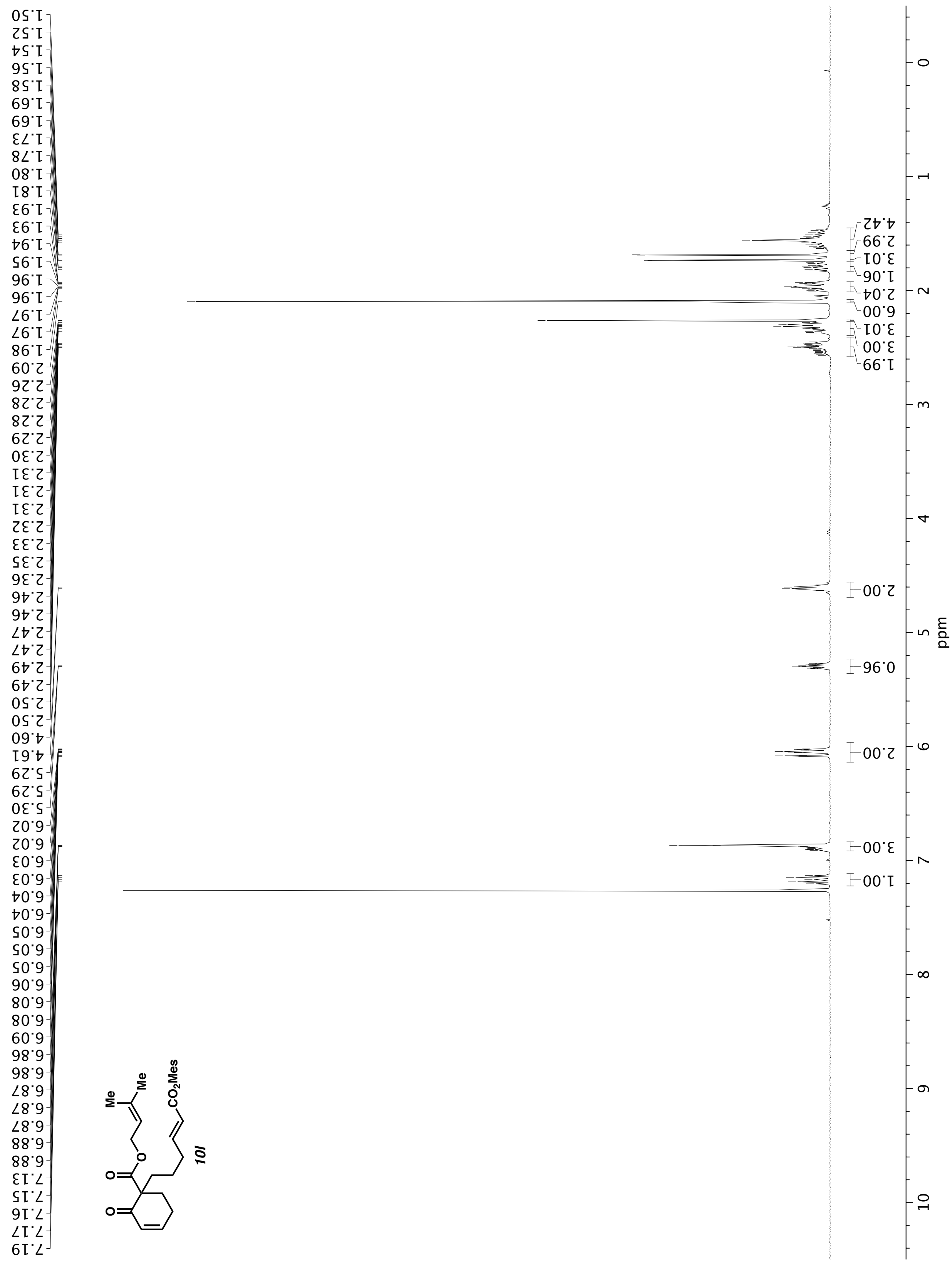

^{1}H NMR (400 MHz, $CDCl_3$) of compound **10l.**

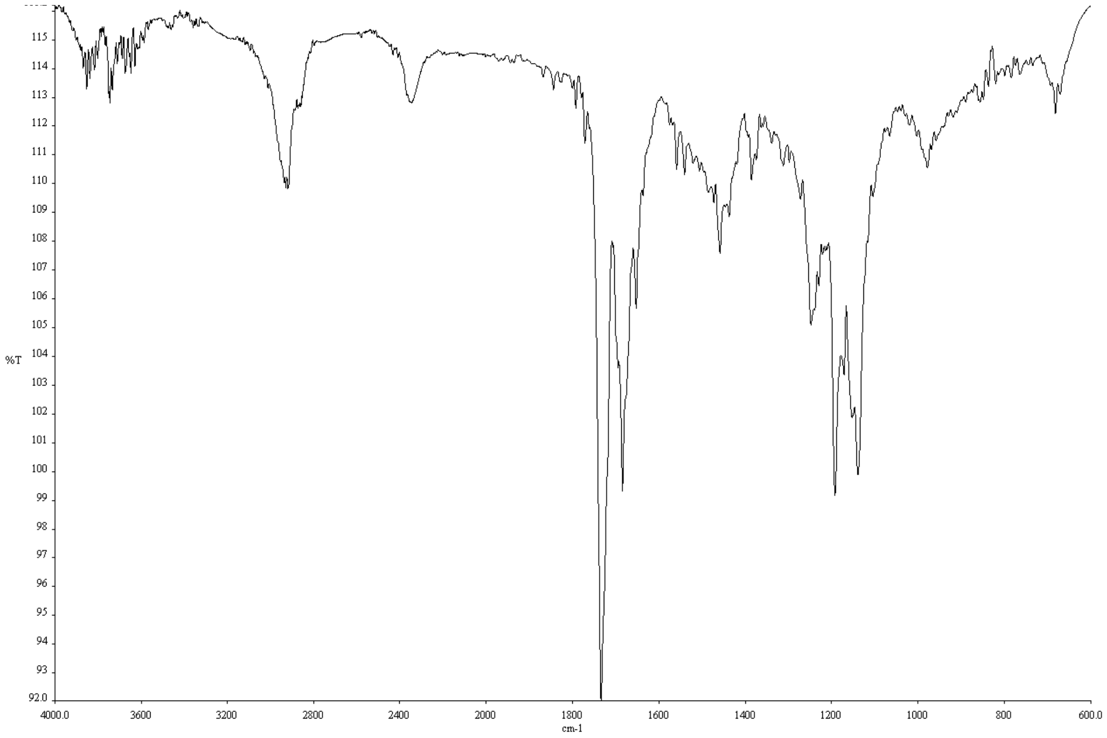

Infrared spectrum (Thin Film, NaCl) of compound **10l**.

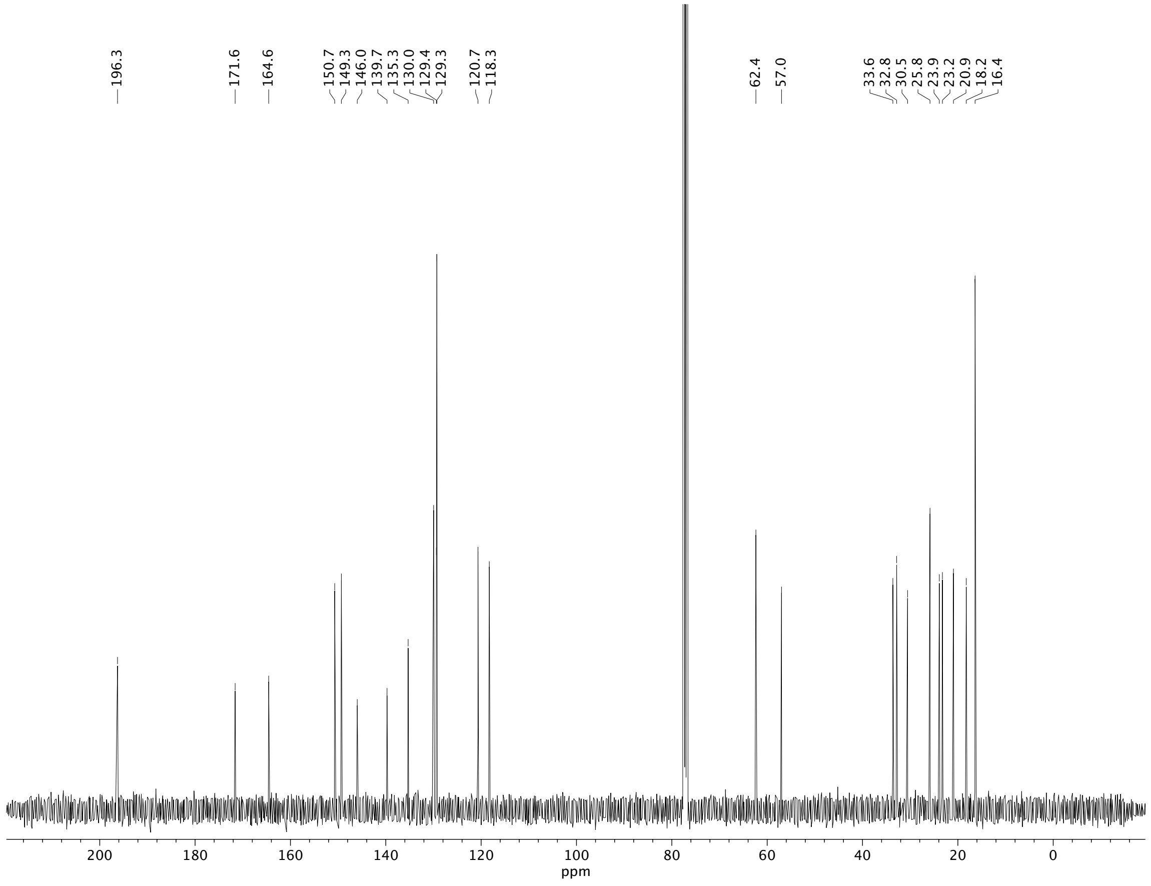

^{13}C NMR (100 MHz, $CDCl_3$) of compound **10l**.

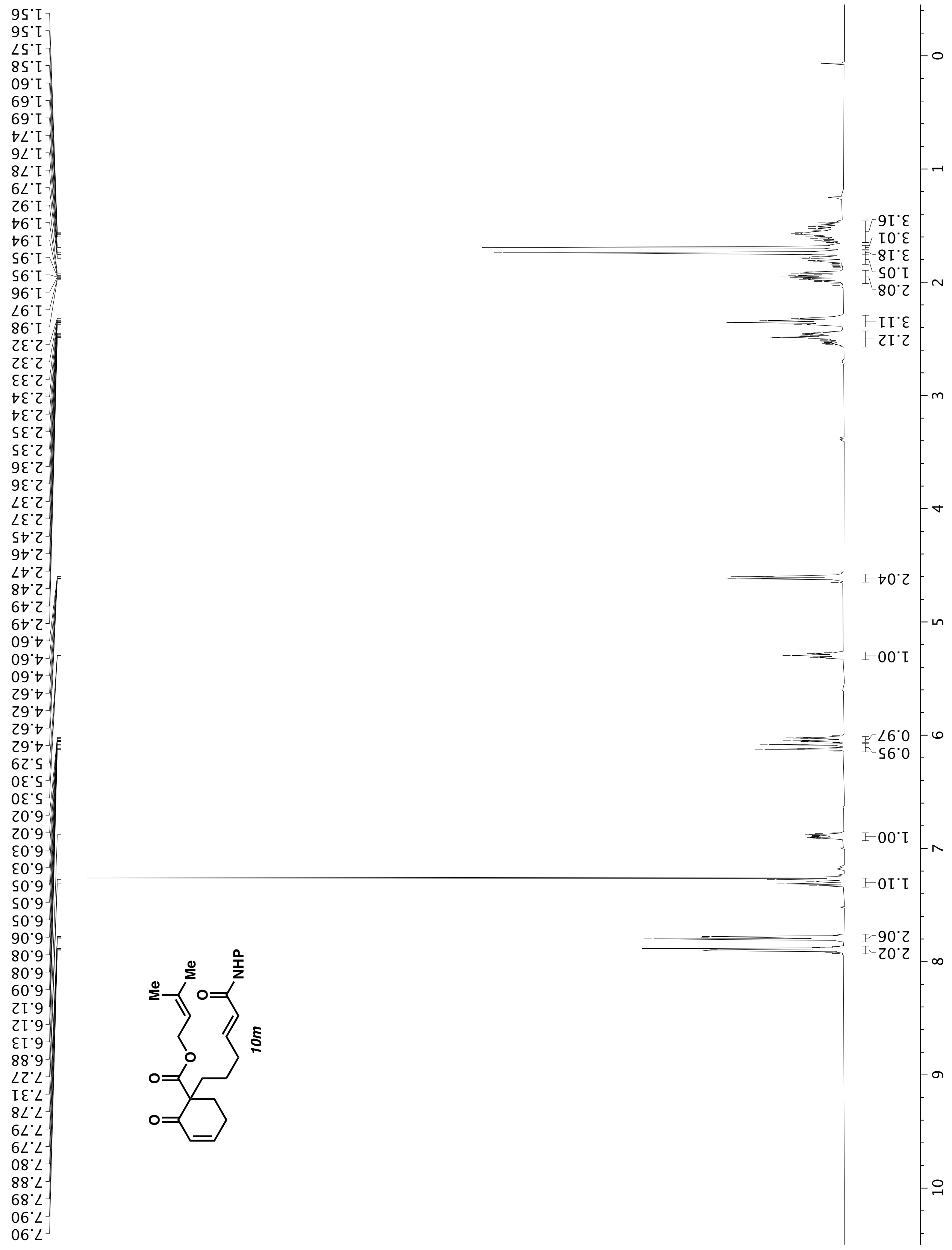

^{1}H NMR (400 MHz, $CDCl_3$) of compound **10m**.

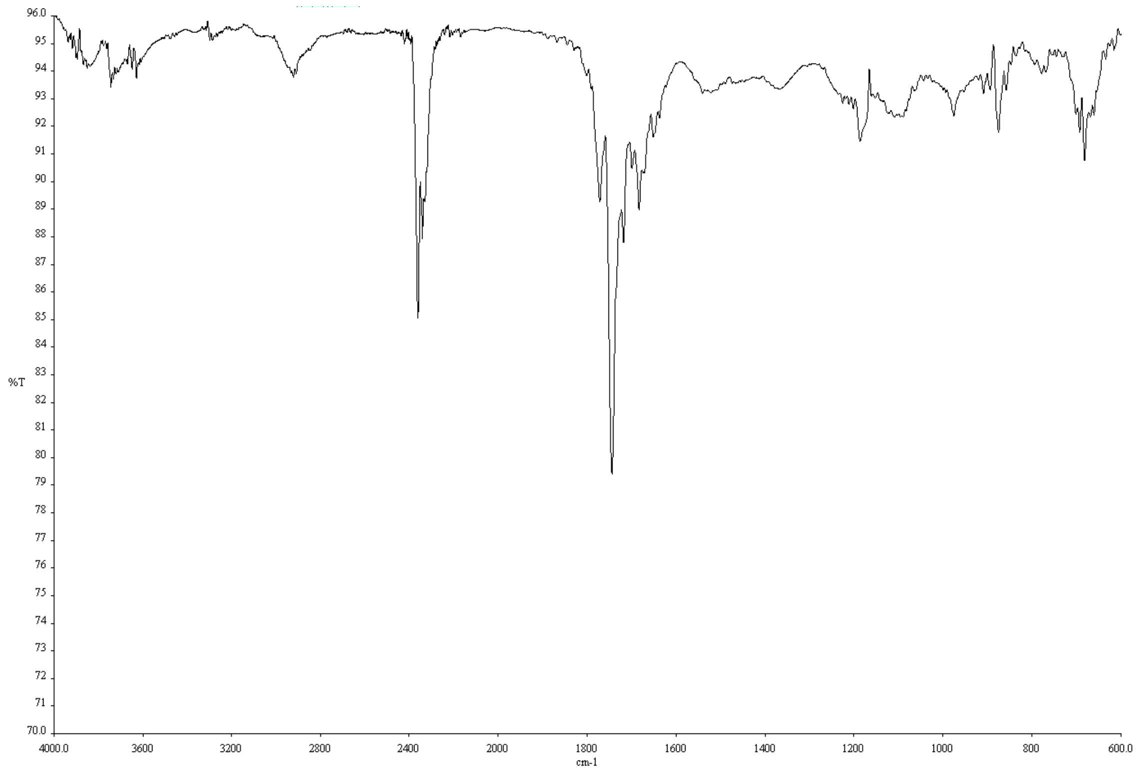

Infrared spectrum (Thin Film, NaCl) of compound **10m**.

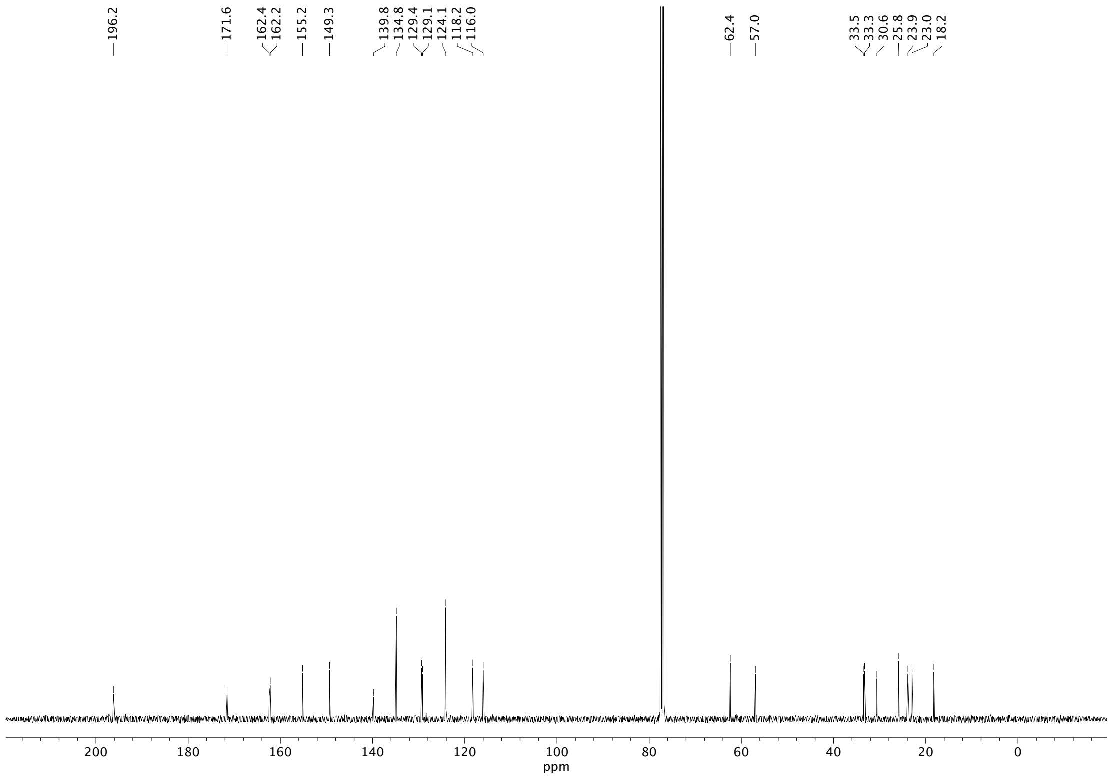

^{13}C NMR (100 MHz, $CDCl_3$) of compound **10m**.

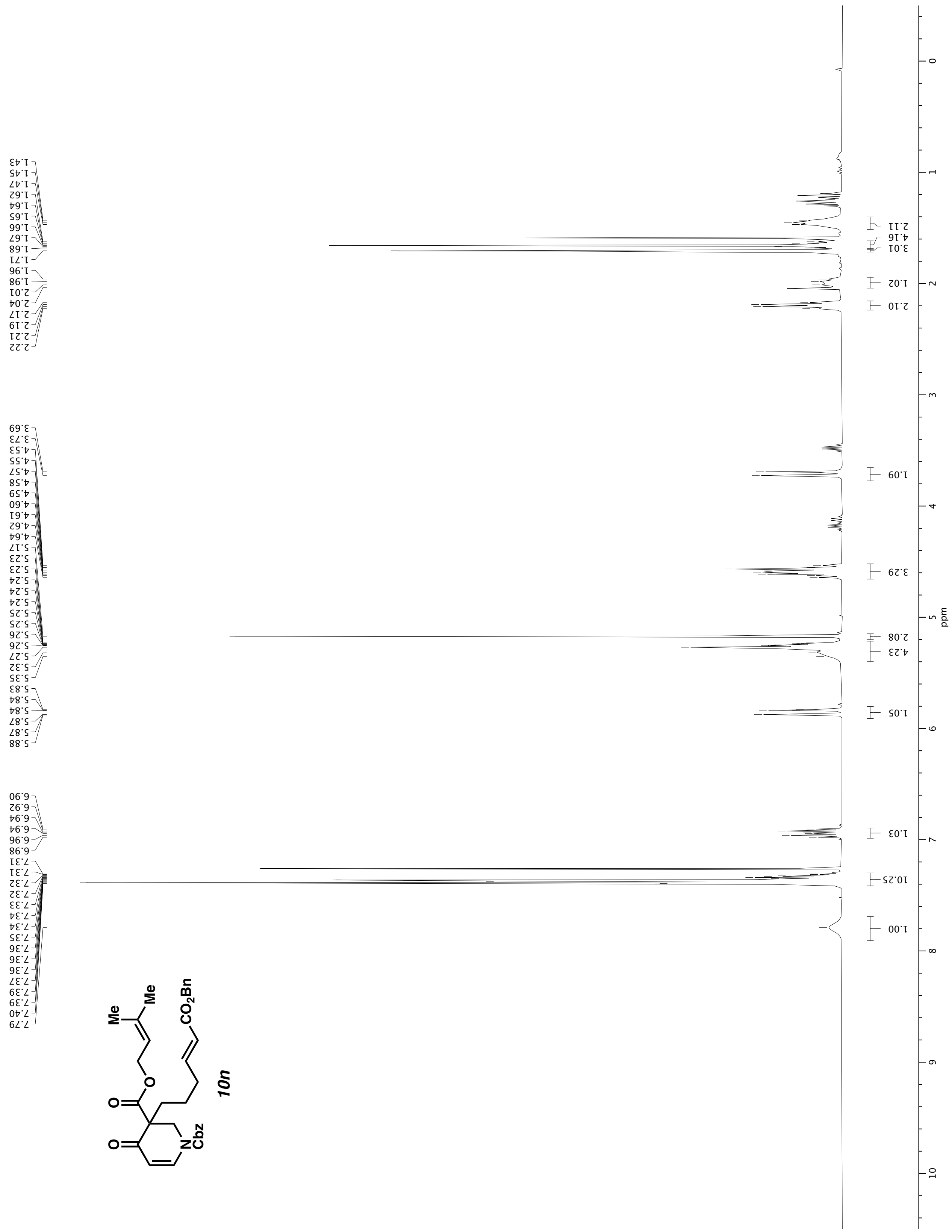

^1H NMR (400 MHz, $CDCl_3$) of compound **10n**.

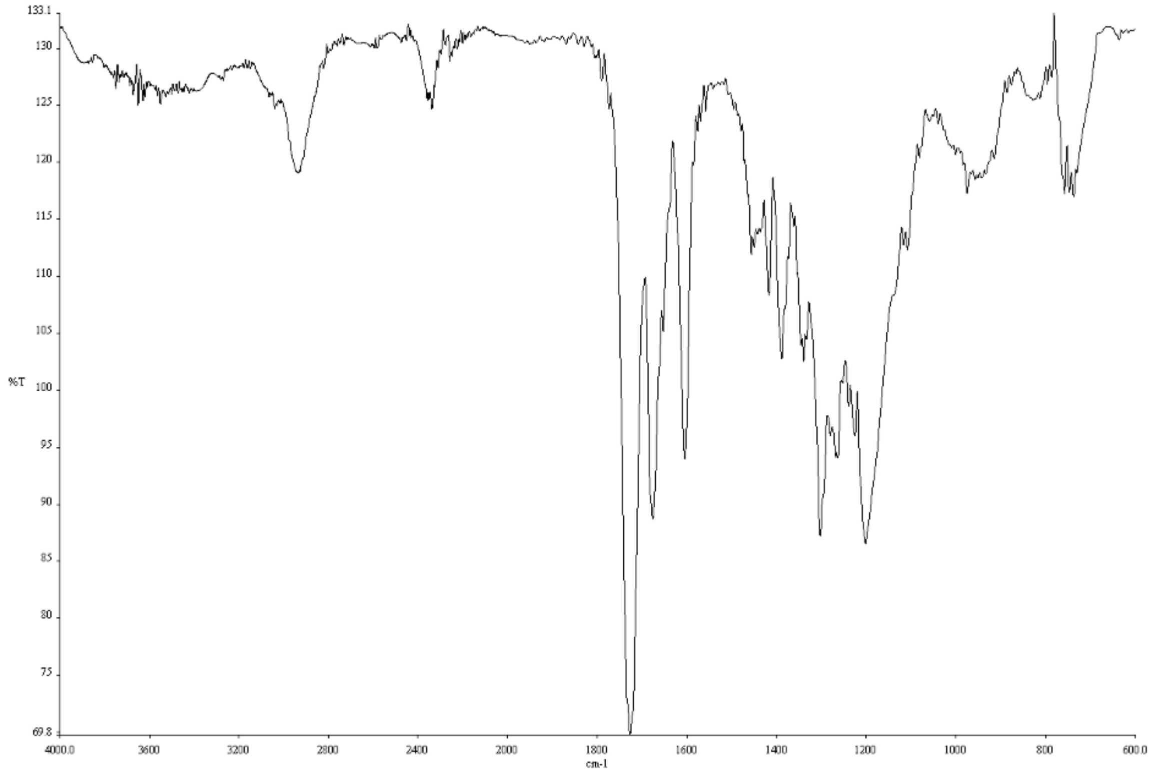

Infrared spectrum (Thin Film, NaCl) of compound **10n**.

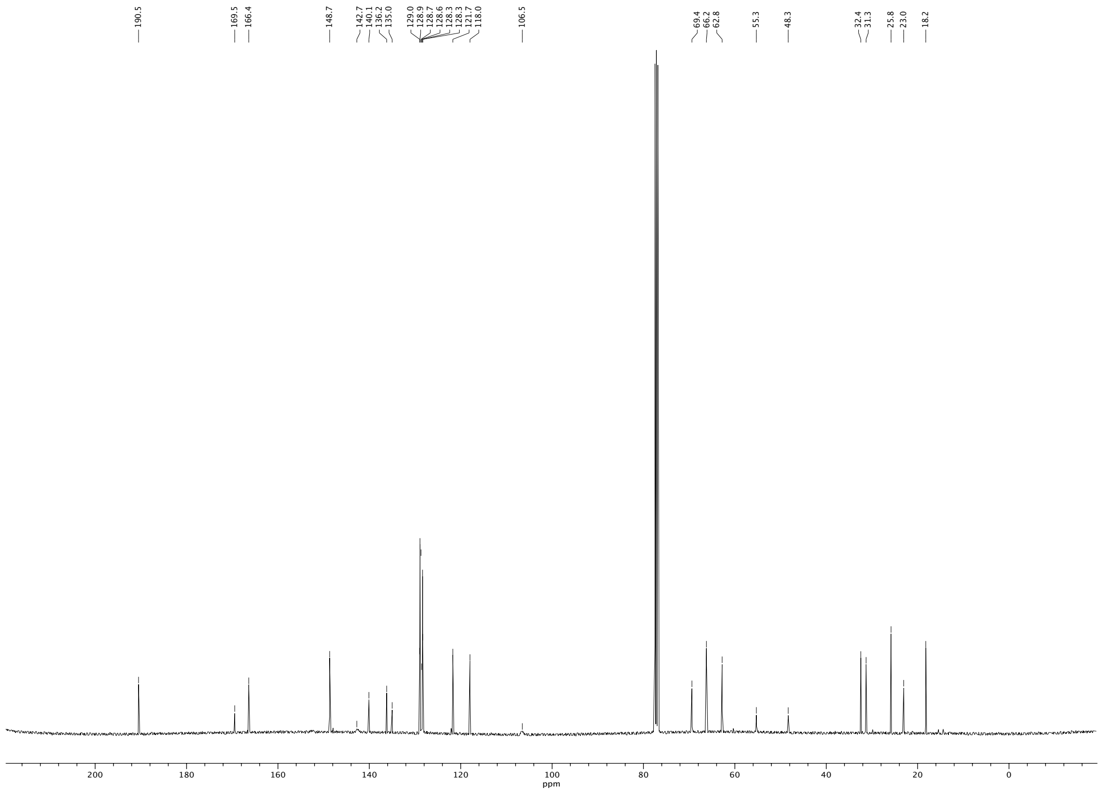

^{13}C NMR (100 MHz, $CDCl_3$) of compound **10n**.

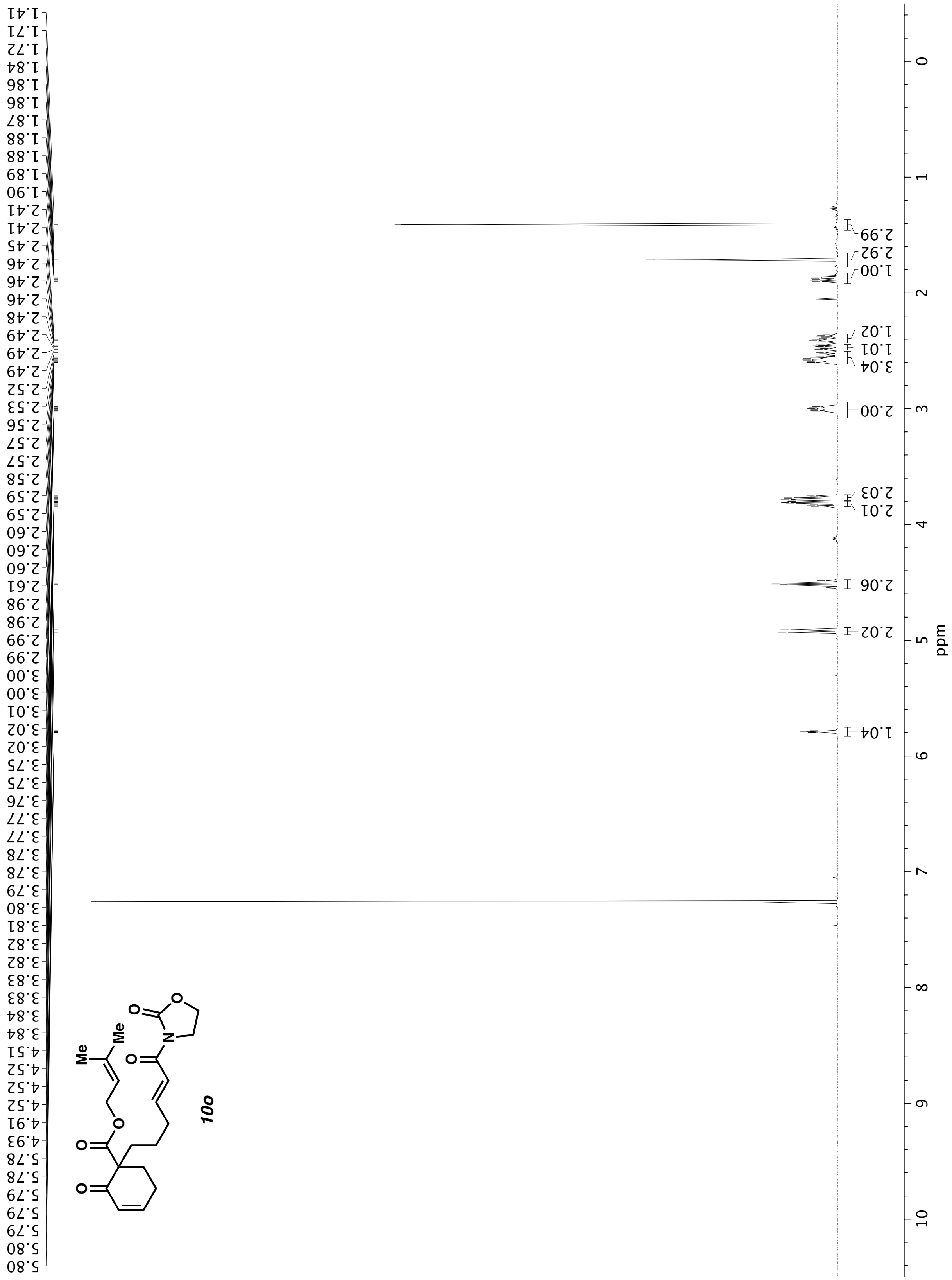

^{1}H NMR (400 MHz, $CDCl_3$) of compound **10o**.

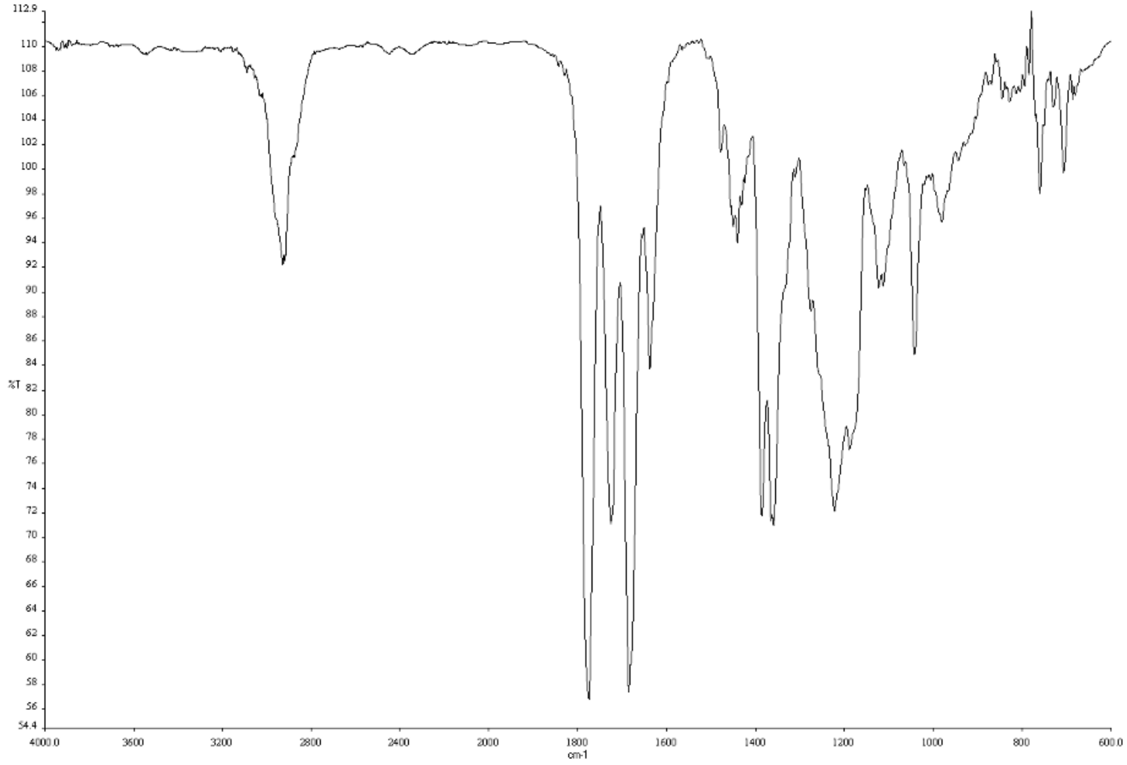

Infrared spectrum (Thin Film, NaCl) of compound **10o.**

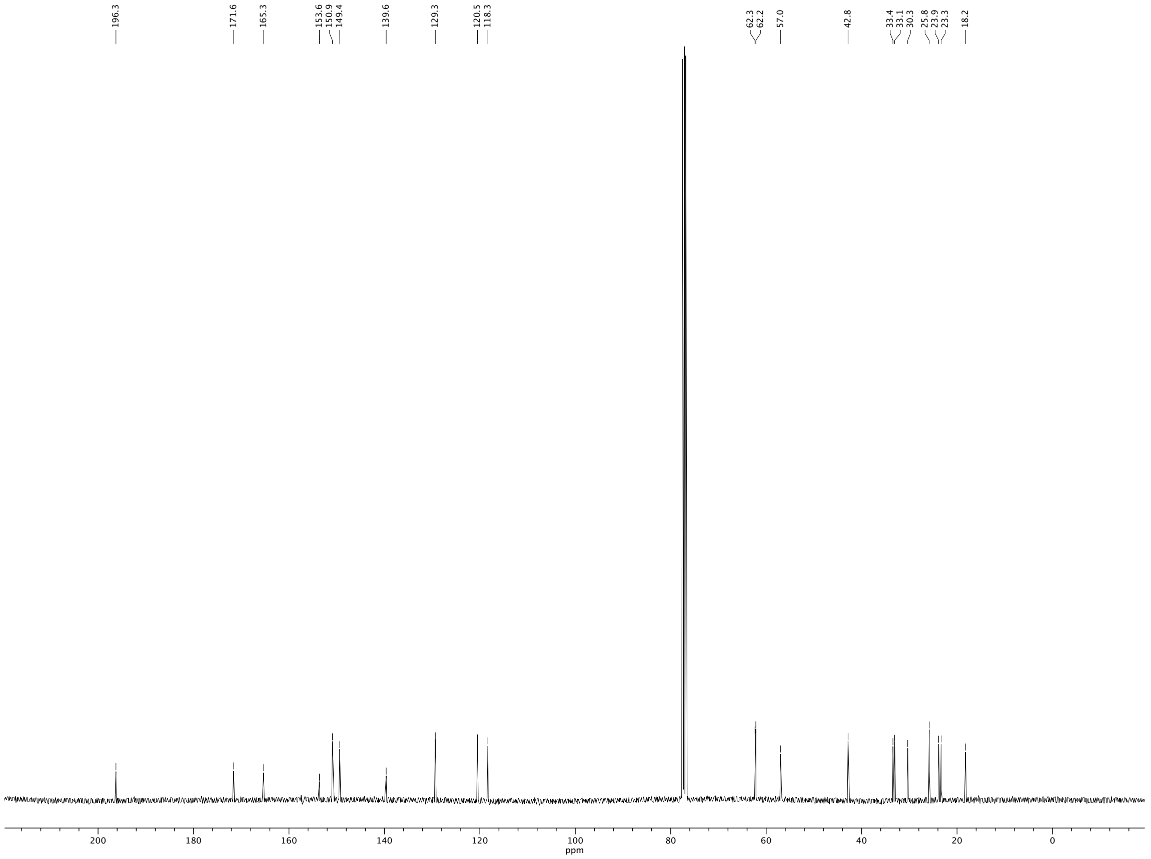

^{13}C NMR (100 MHz, $CDCl_3$) of compound **10o**.

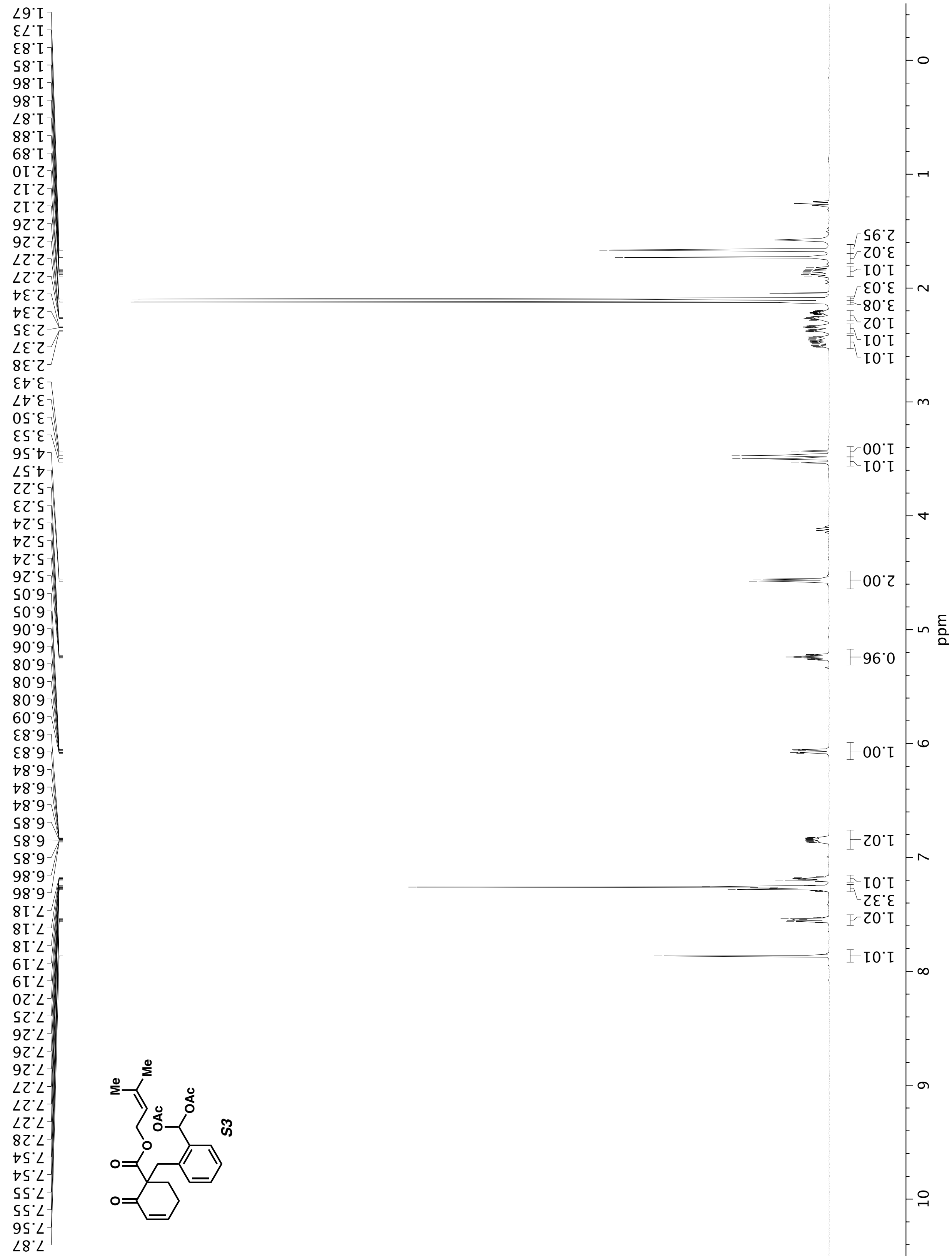

^{1}H NMR (400 MHz, $CDCl_3$) of compound **S3.**

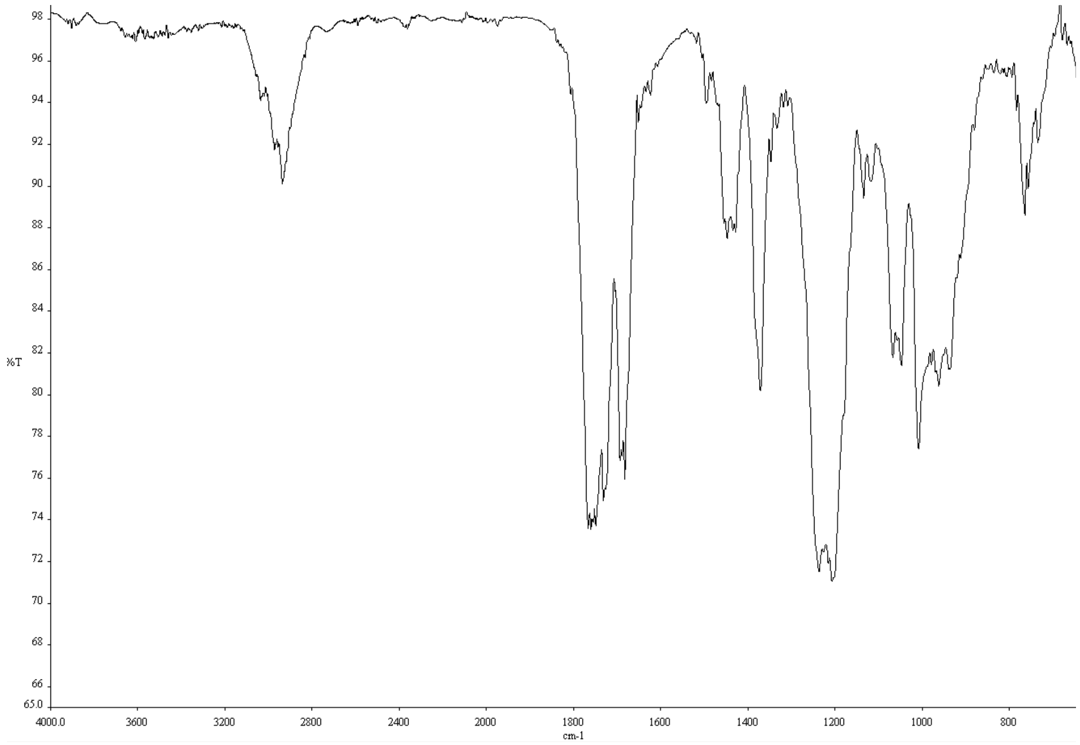

Infrared spectrum (Thin Film, NaCl) of compound **S3**.

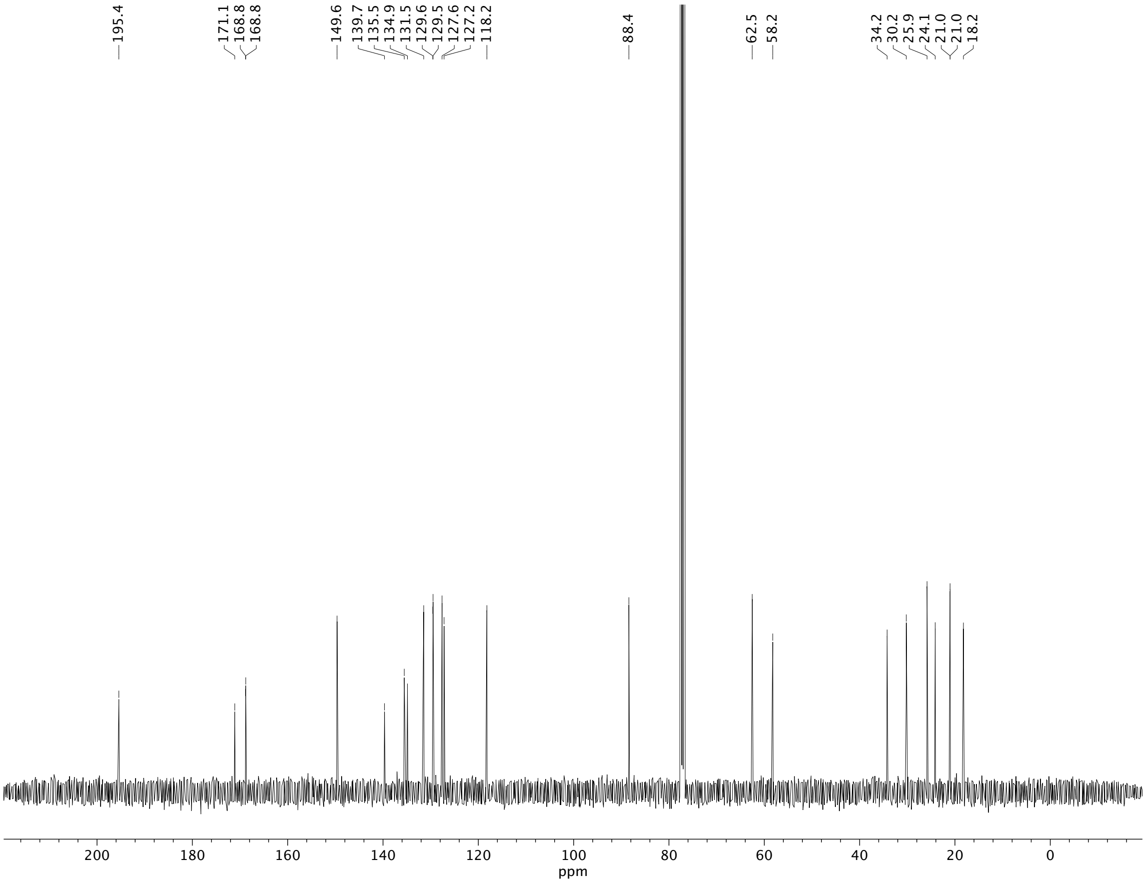

^{13}C NMR (100 MHz, $CDCl_3$) of compound **S3**.

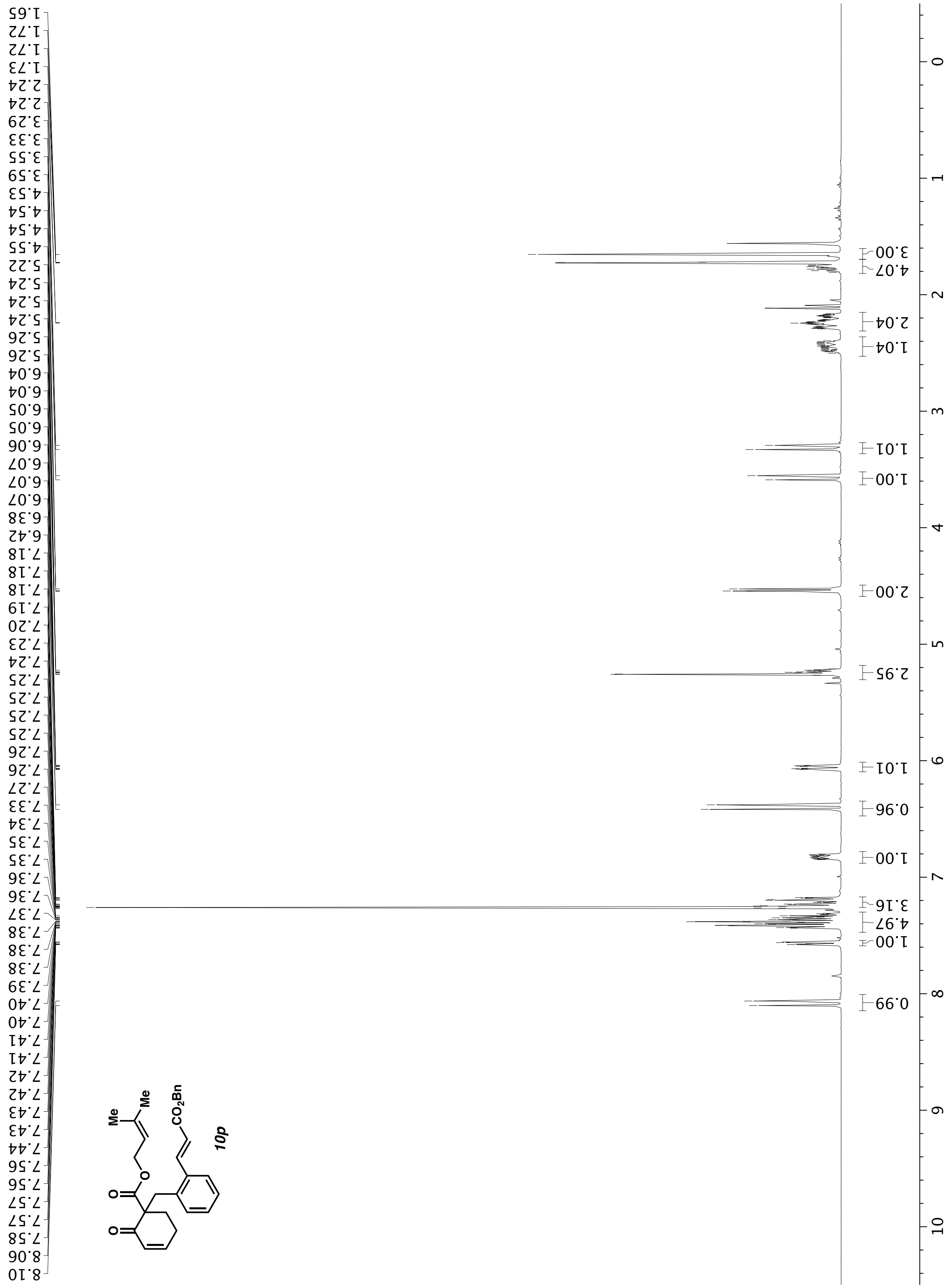

^{1}H NMR (400 MHz, $CDCl_3$) of compound **10p.**

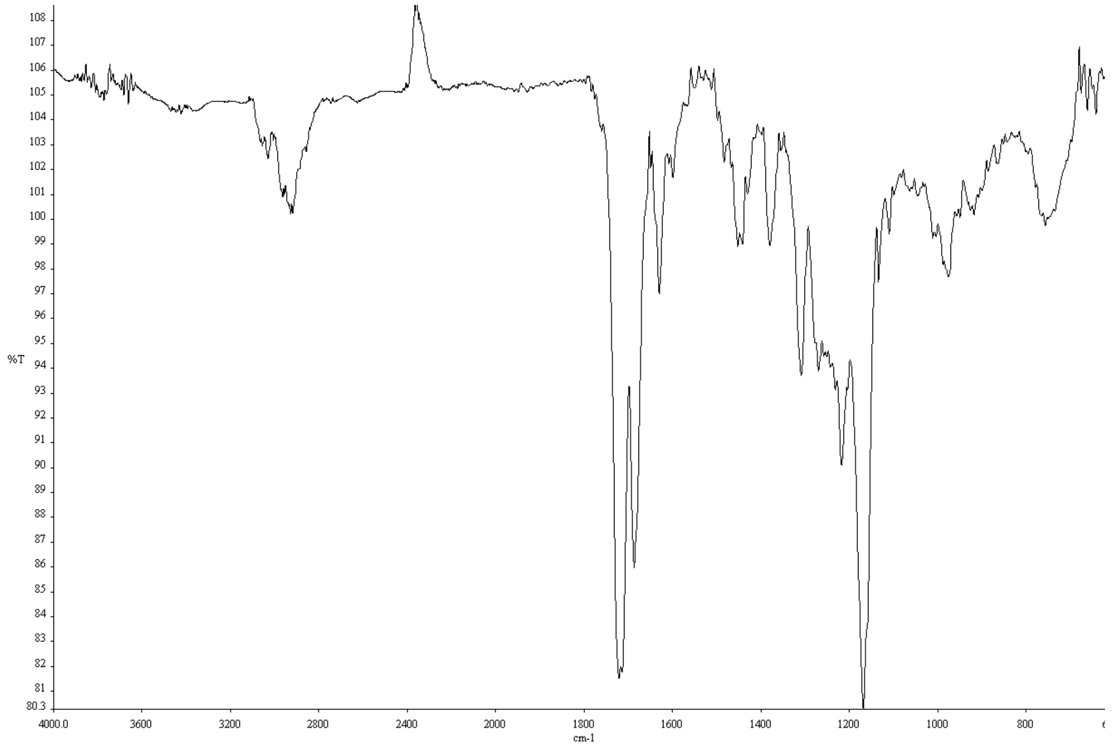

Infrared spectrum (Thin Film, NaCl) of compound **10p**.

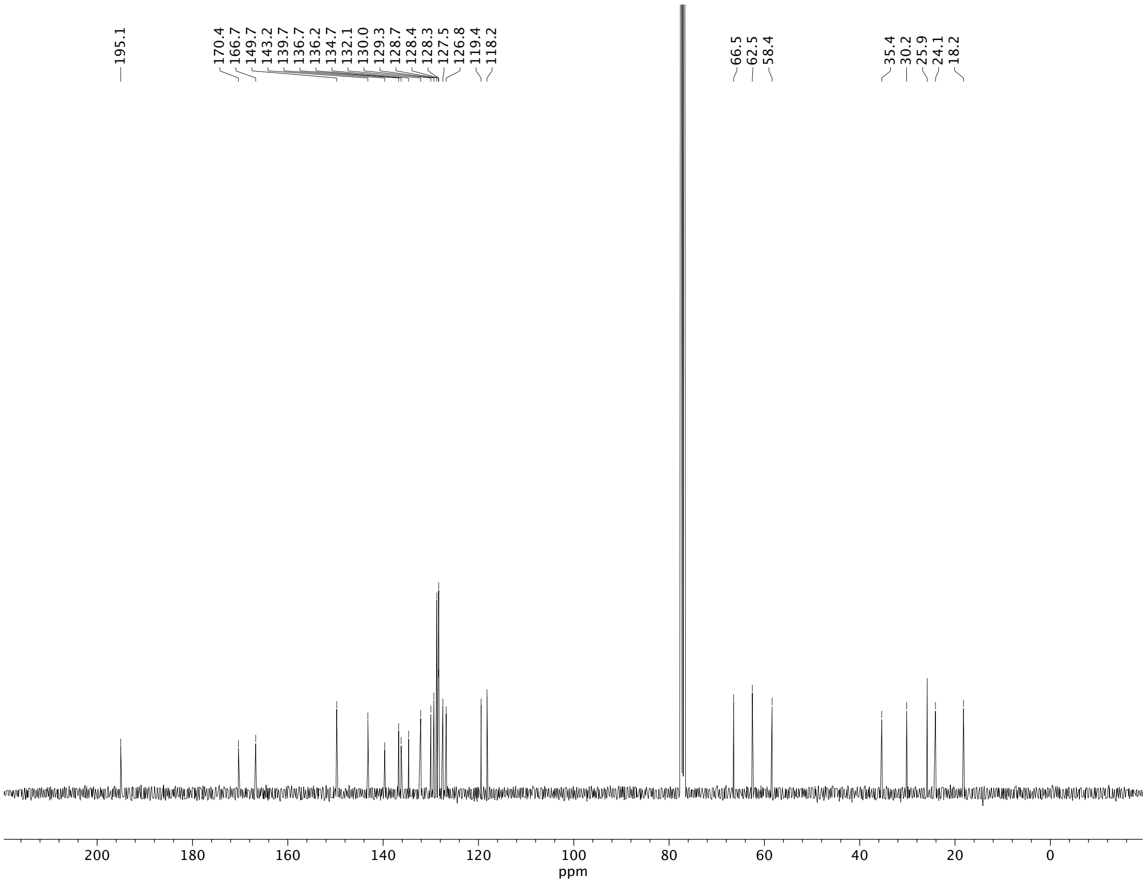

^{13}C NMR (100 MHz, $CDCl_3$) of compound **10p**.

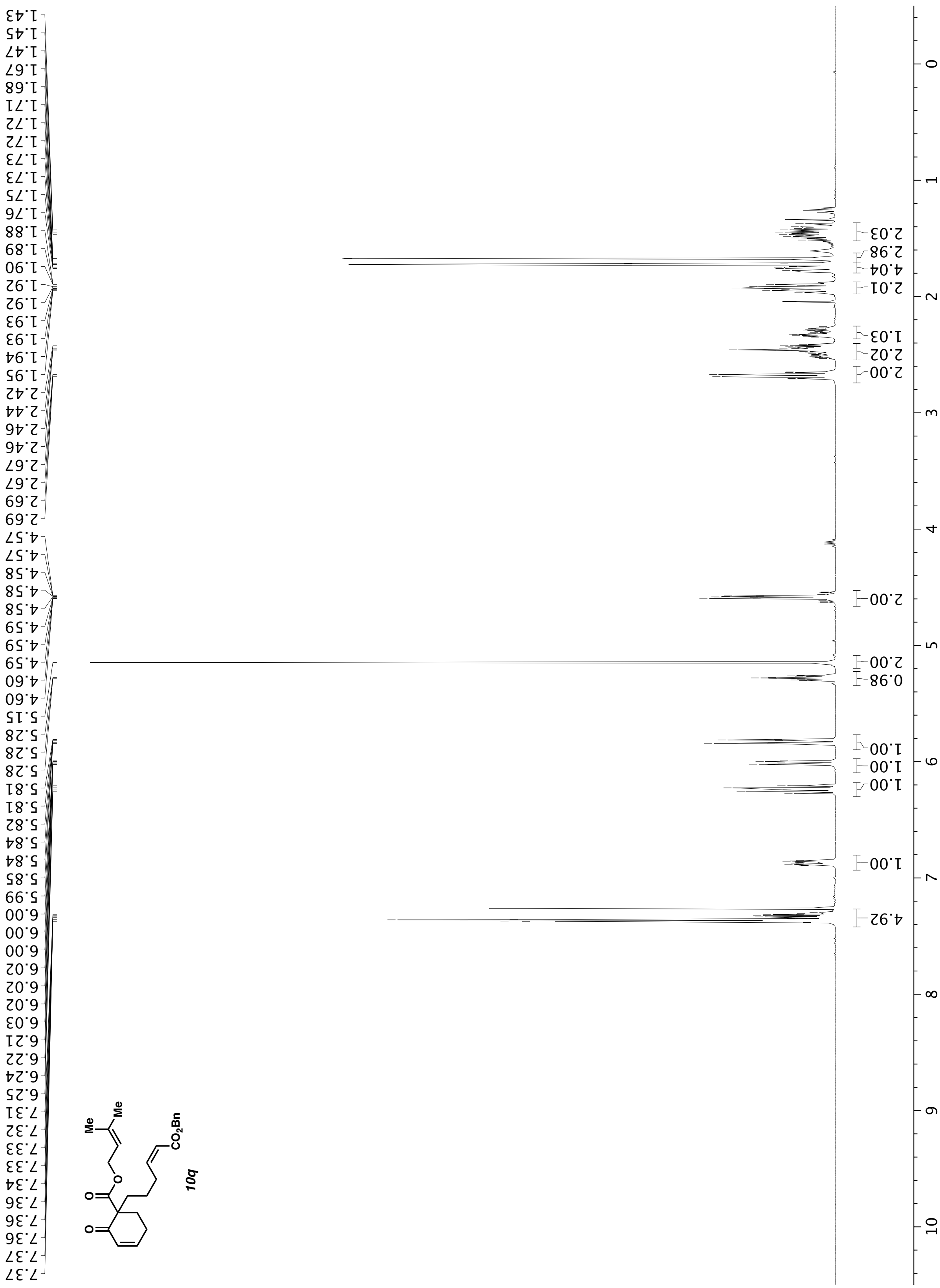

^{1}H NMR (400 MHz, $CDCl_3$) of compound **10q.**

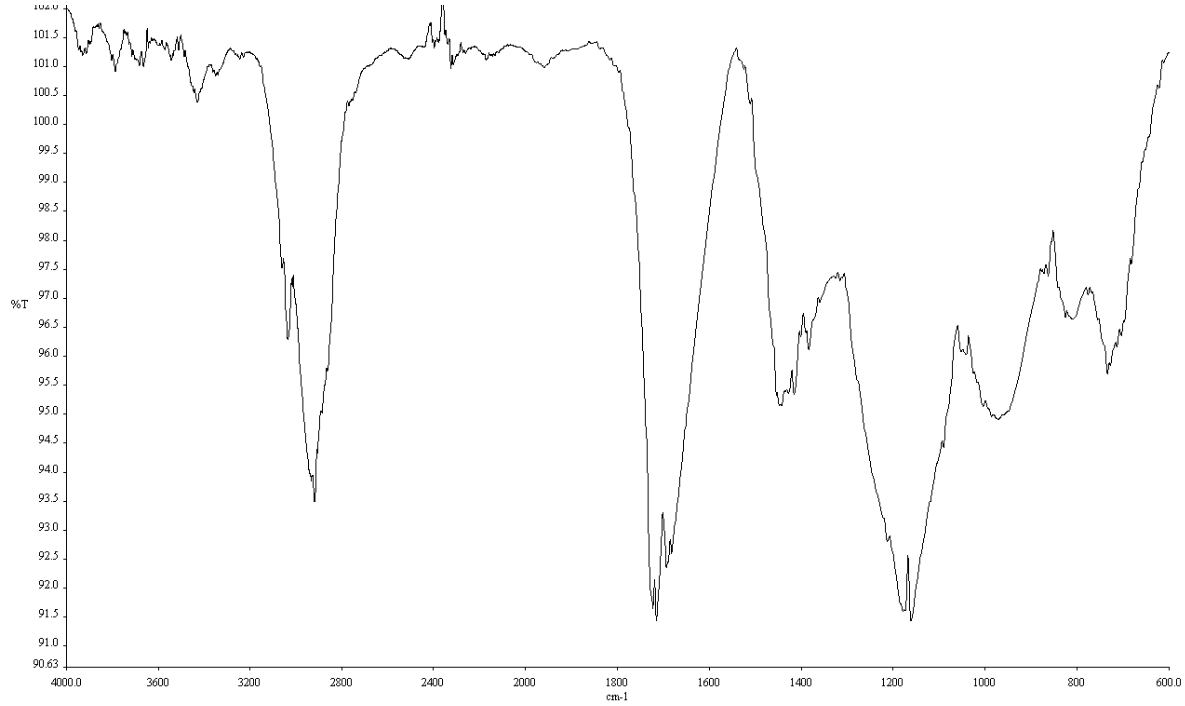

Infrared spectrum (Thin Film, NaCl) of compound **10q**.

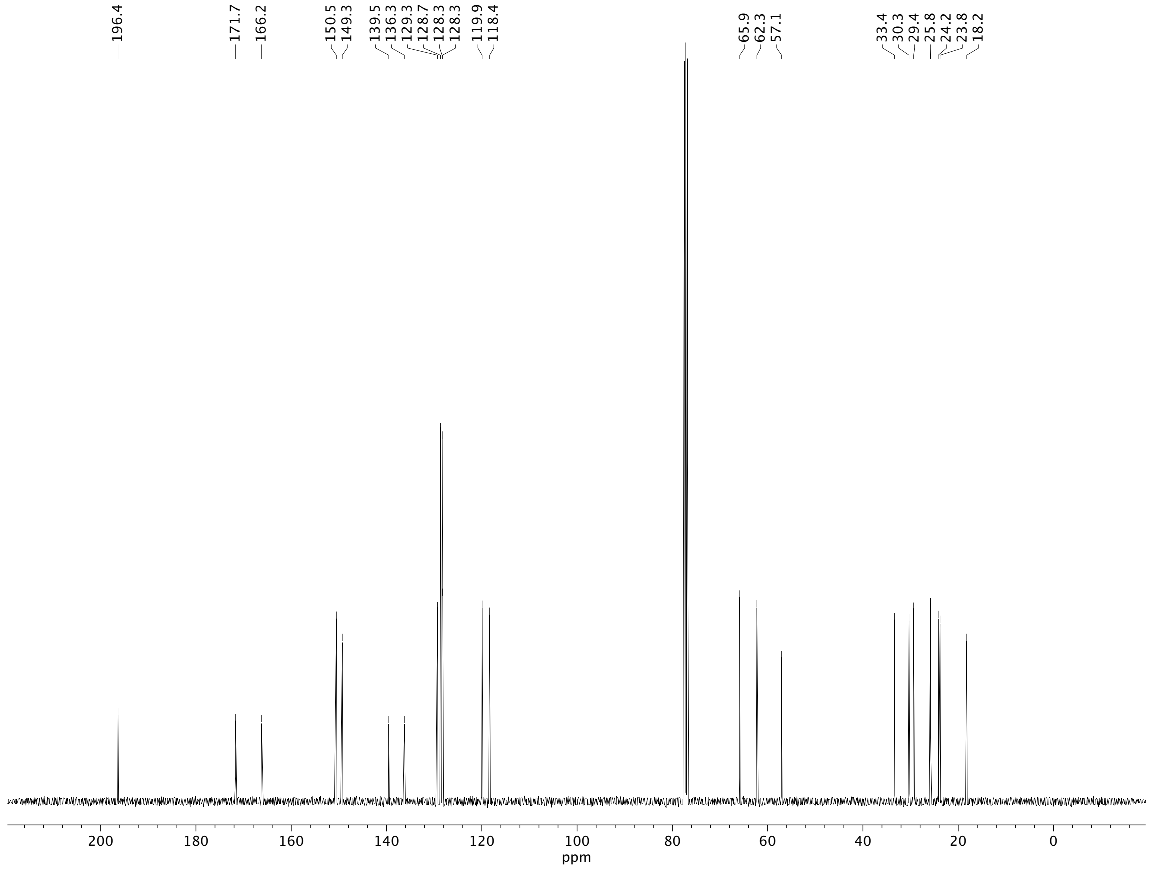

^{13}C NMR (100 MHz, $CDCl_3$) of compound **10q**.

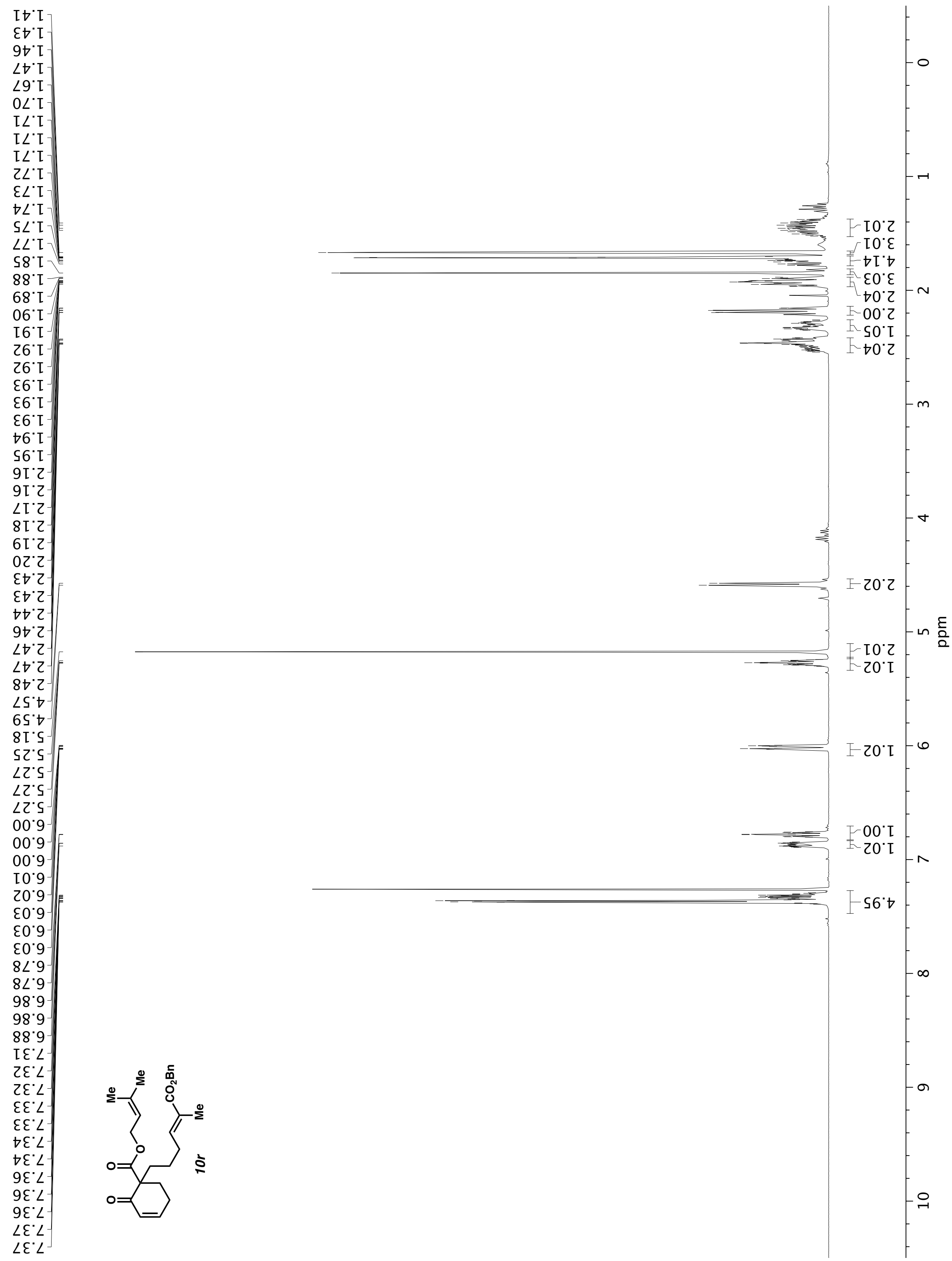

^{1}H NMR (400 MHz, $CDCl_3$) of compound **10r.**

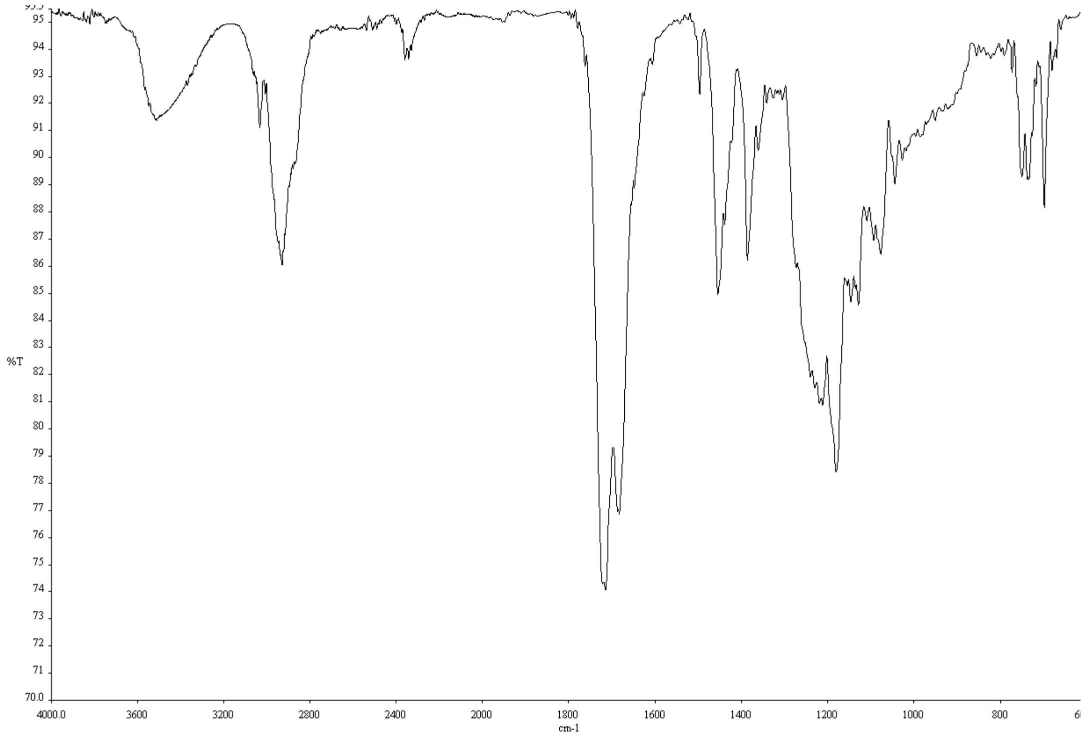

Infrared spectrum (Thin Film, NaCl) of compound **10r**.

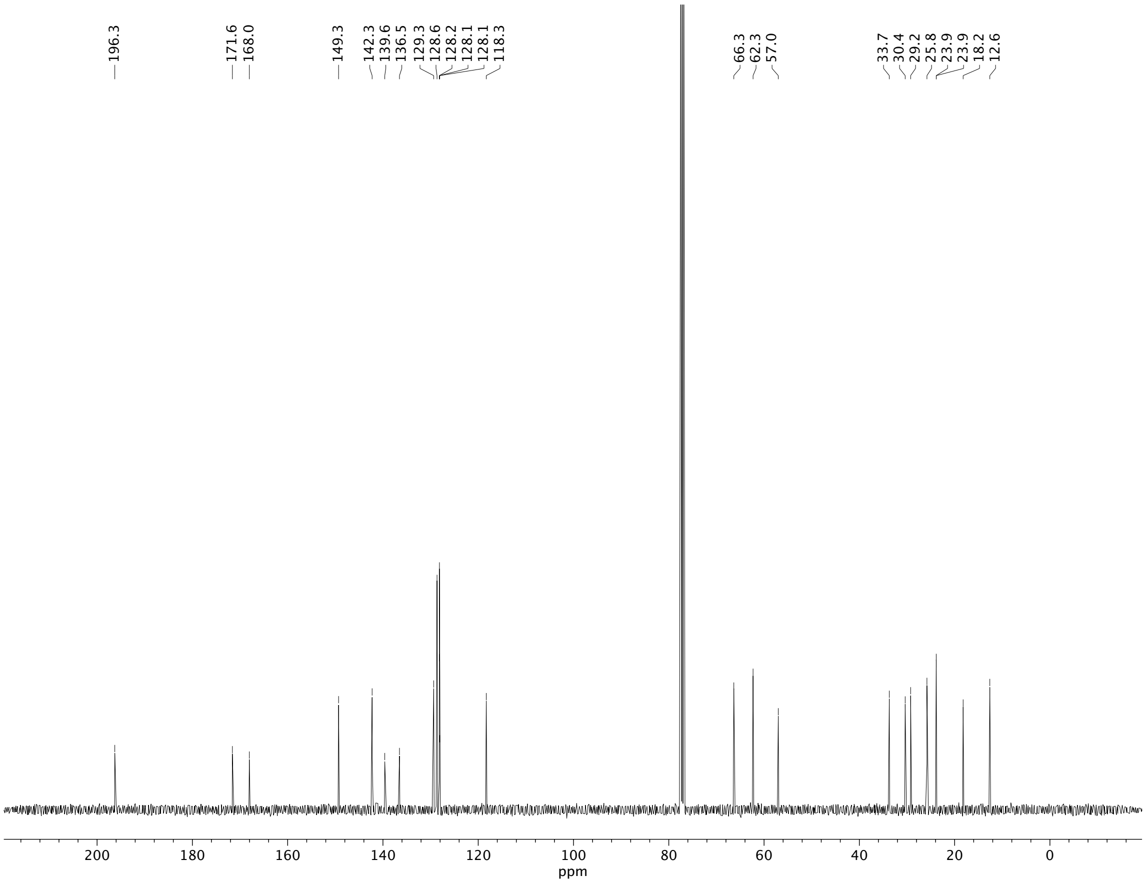

^{13}C NMR (100 MHz, $CDCl_3$) of compound **10r**.

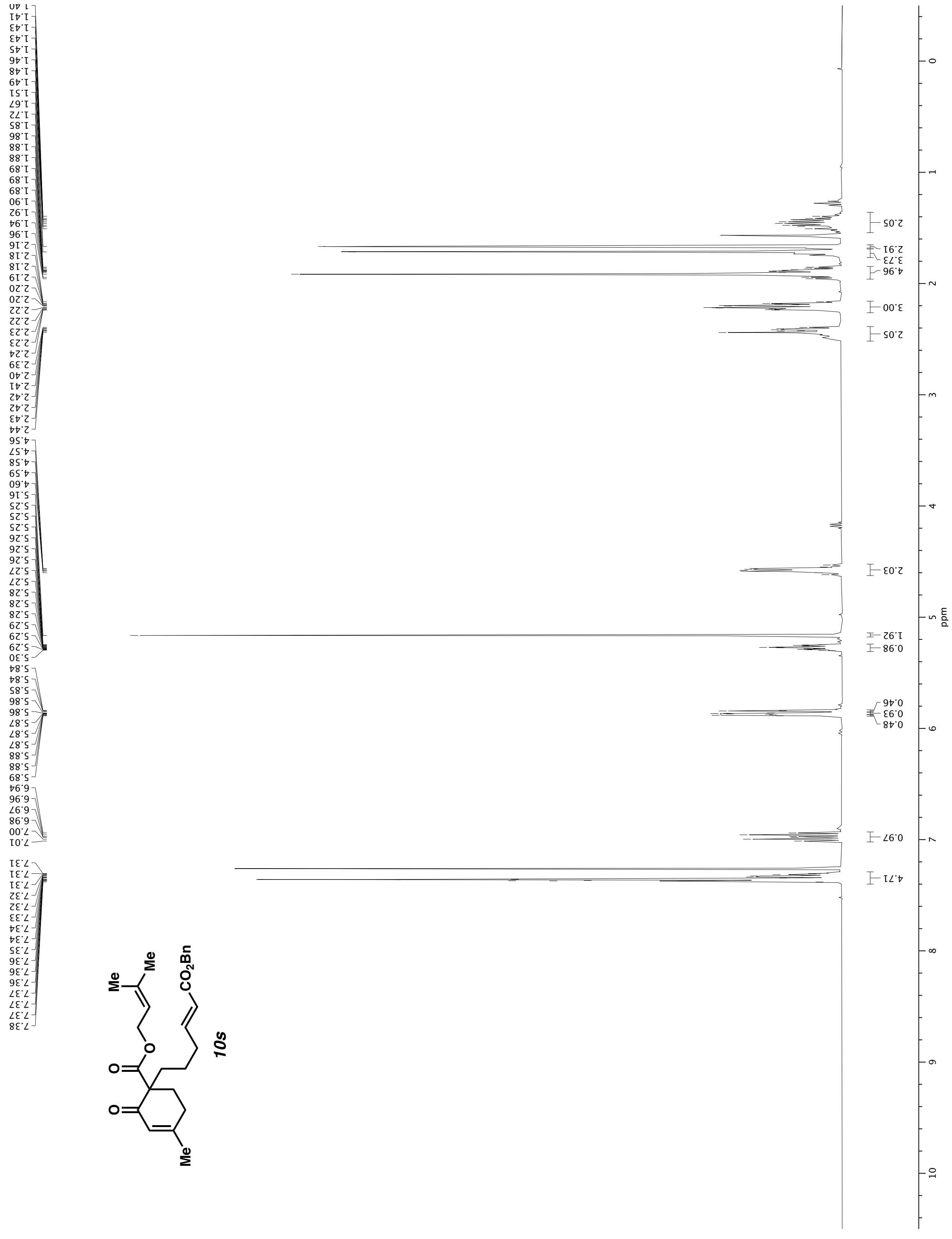

^{1}H NMR (400 MHz, $CDCl_3$) of compound **10s**.

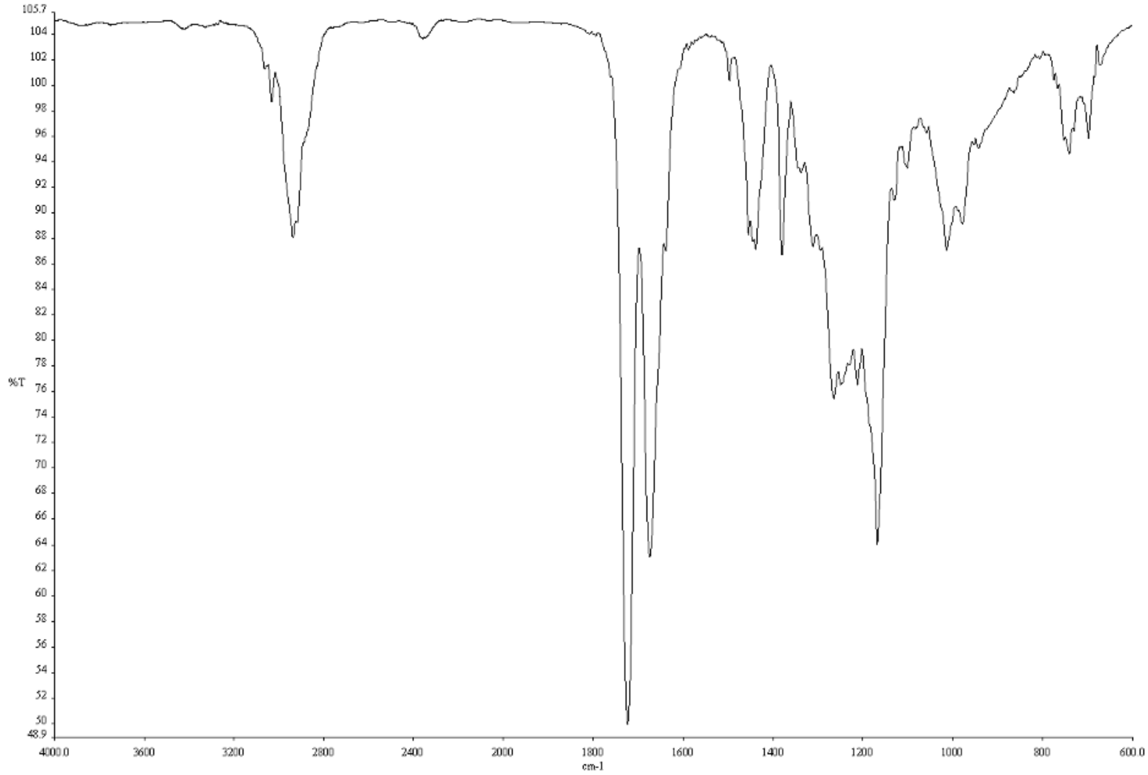

Infrared spectrum (Thin Film, NaCl) of compound **10s**.

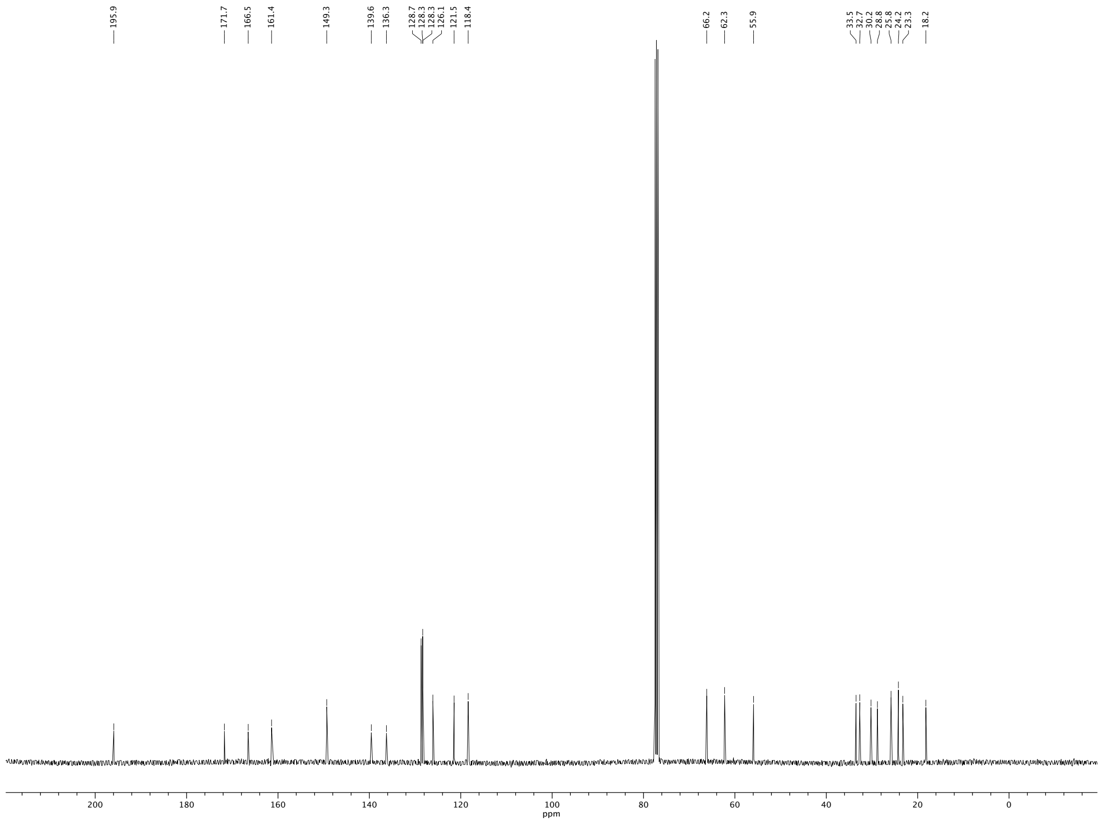

^{13}C NMR (100 MHz, $CDCl_3$) of compound **10s**.

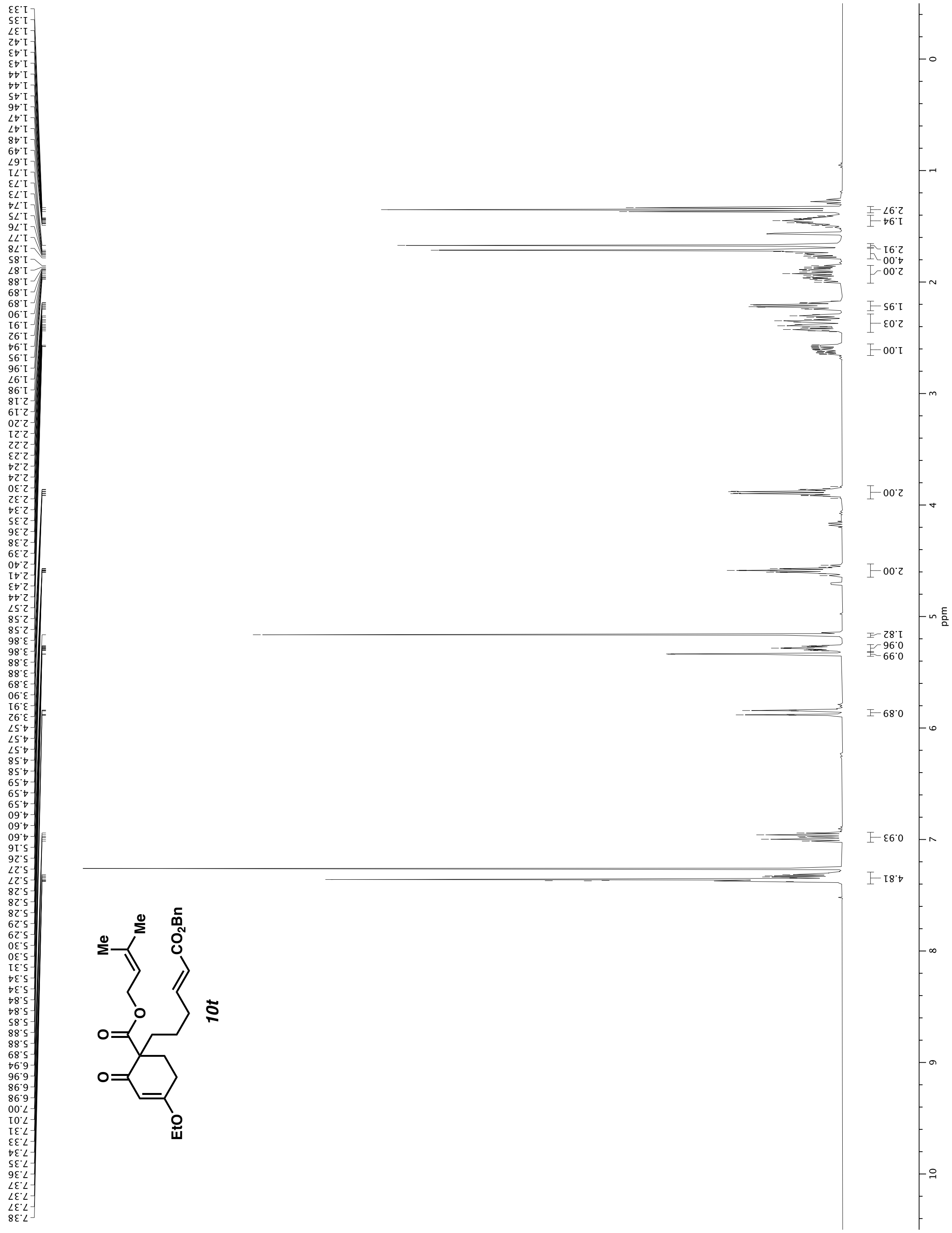

^{1}H NMR (400 MHz, CDCl$_3$) of compound **10t**.

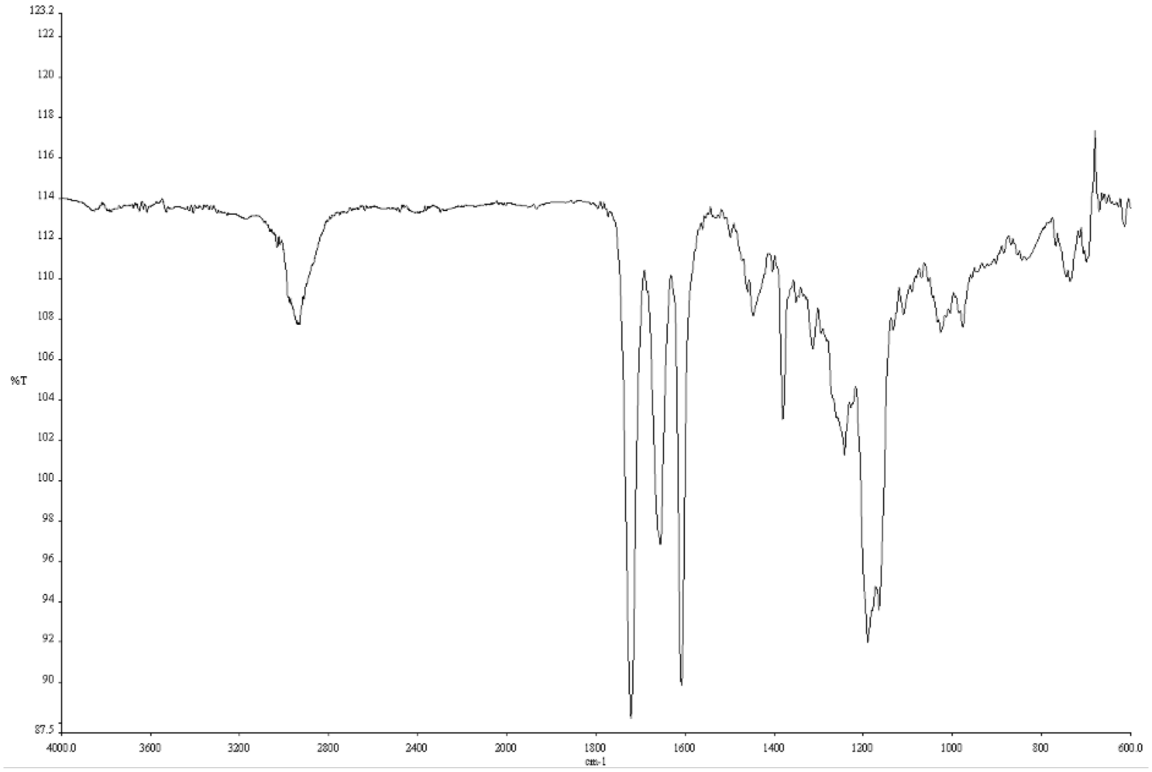

Infrared spectrum (Thin Film, NaCl) of compound **10t**.

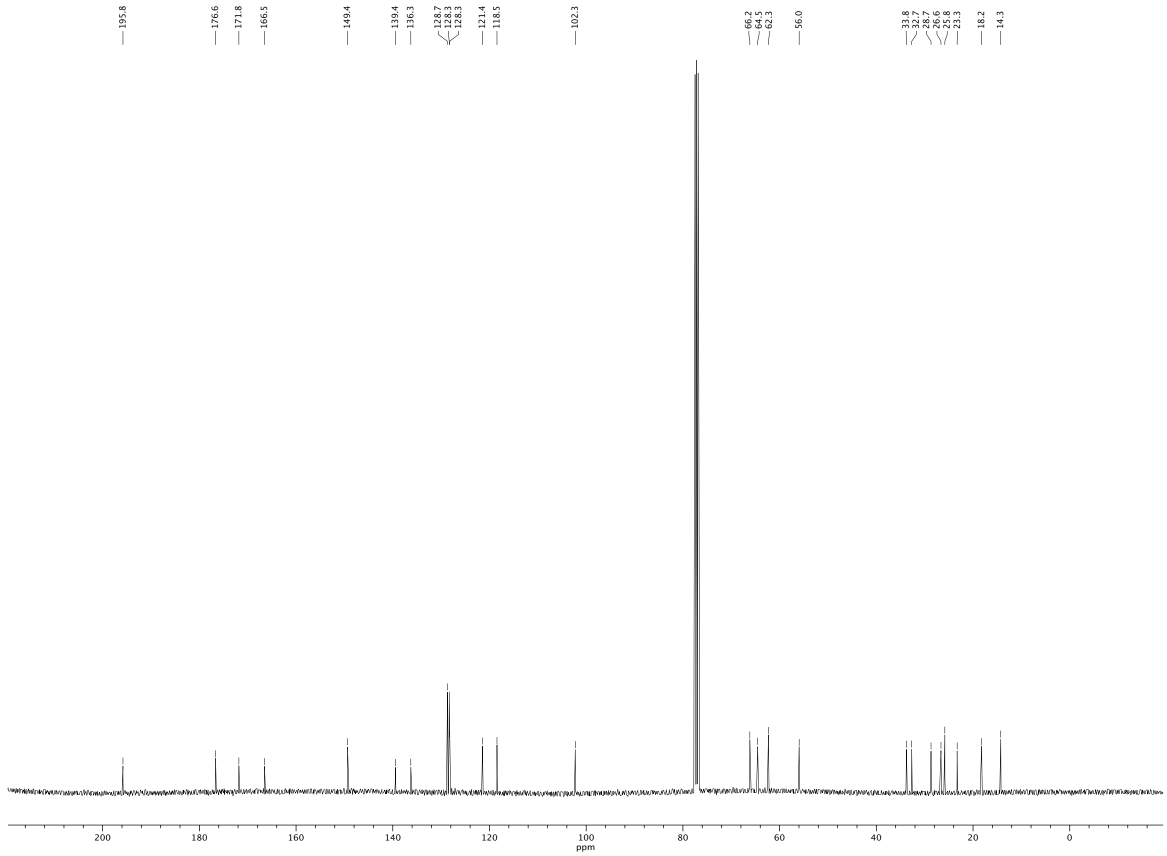

^{13}C NMR (100 MHz, $CDCl_3$) of compound **10t**.

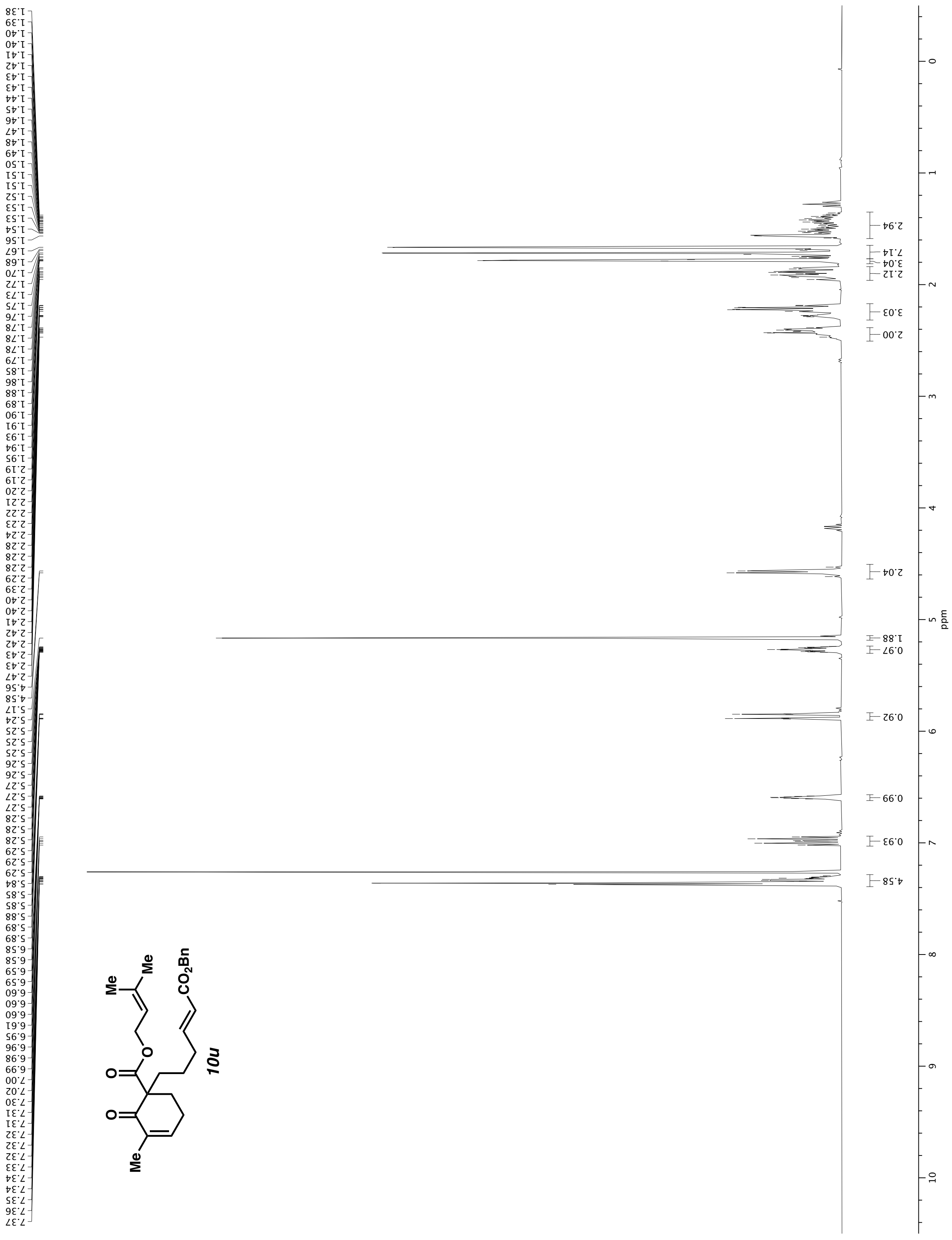

^{1}H NMR (400 MHz, $CDCl_3$) of compound **10u**.

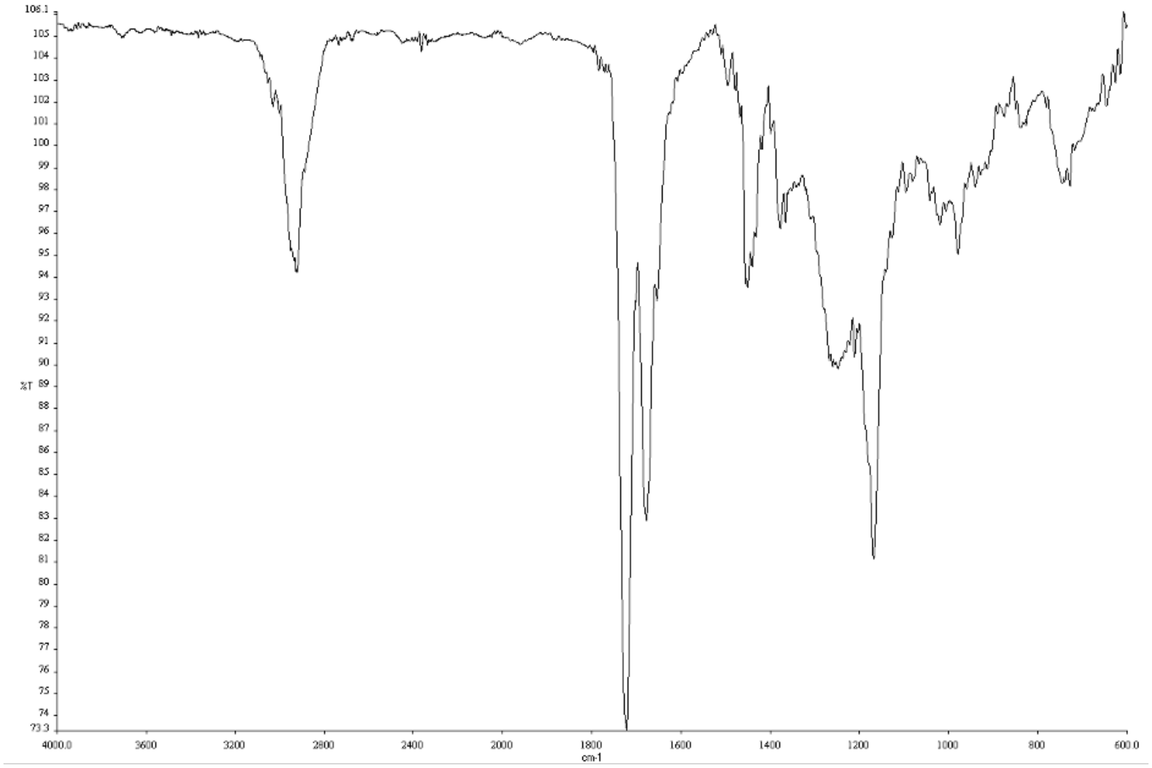

Infrared spectrum (Thin Film, NaCl) of compound **10u**.

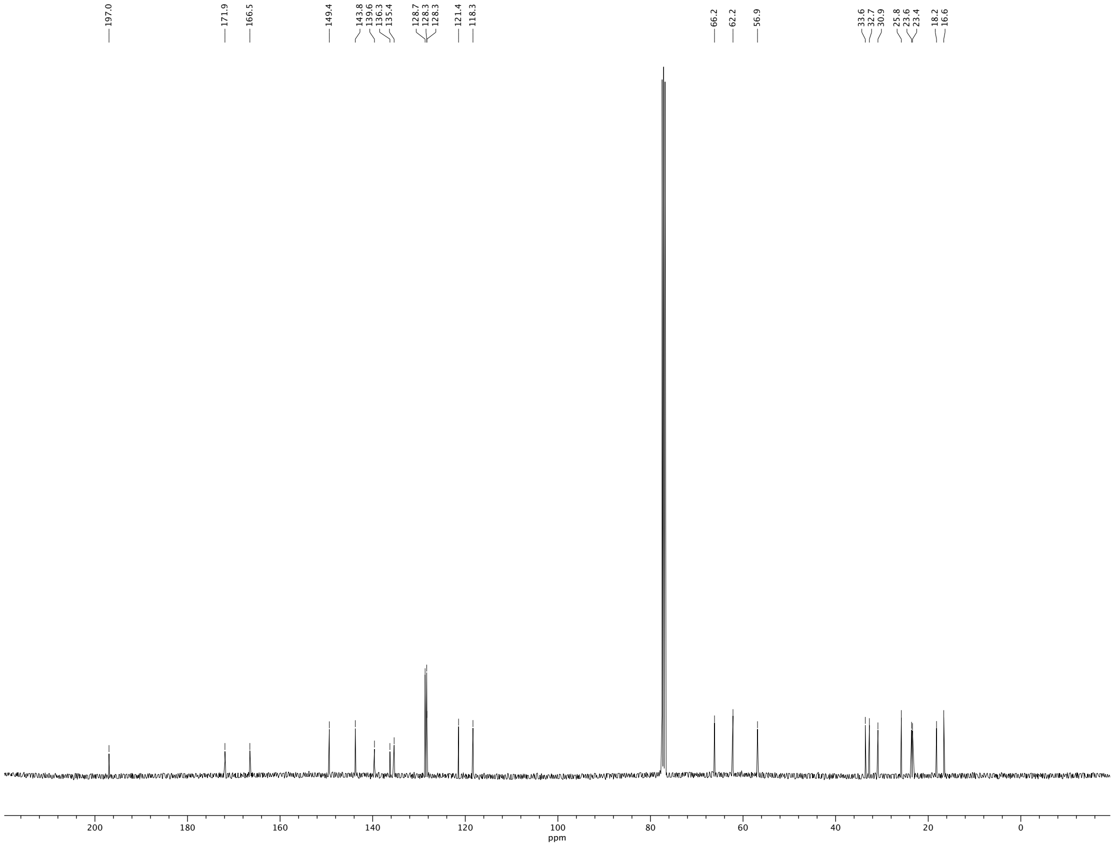

^{13}C NMR (100 MHz, $CDCl_3$) of compound **10u**.

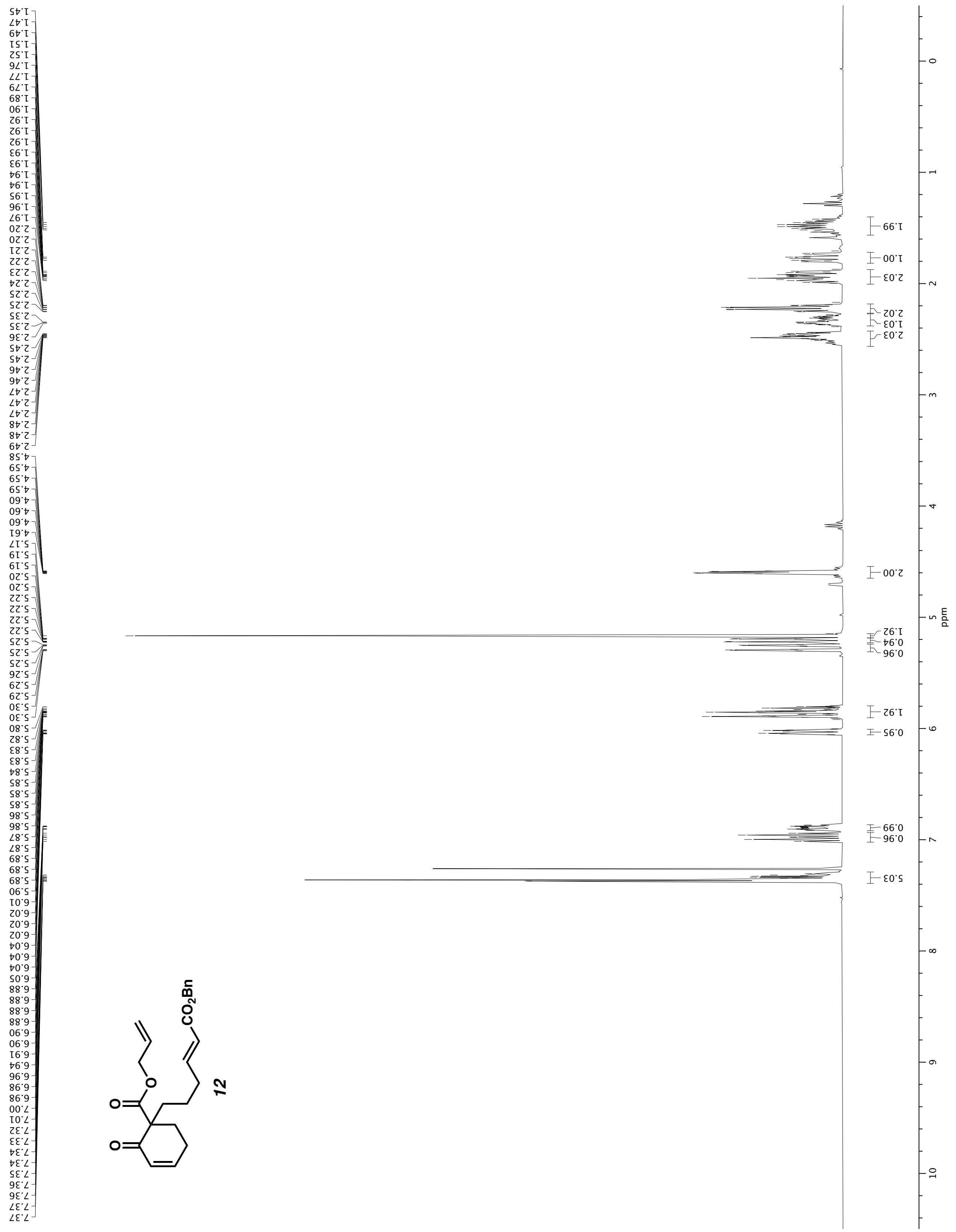

^{1}H NMR (400 MHz, $CDCl_3$) of compound **12**.

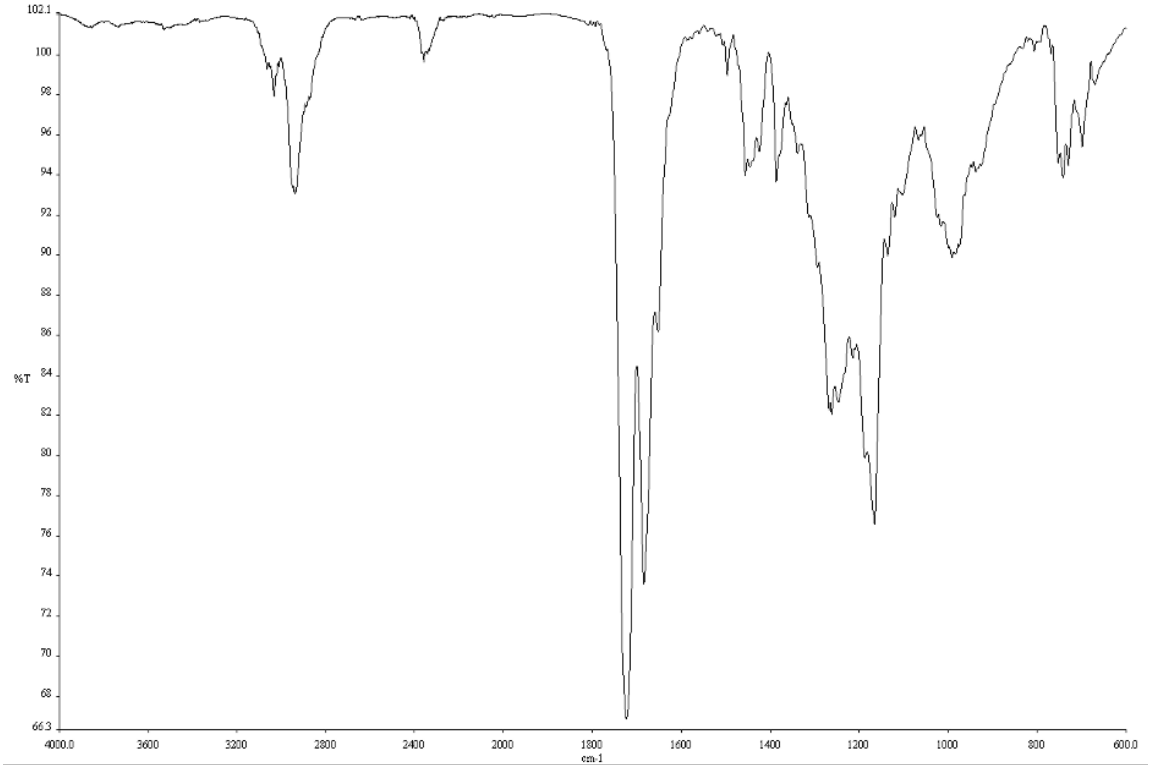

Infrared spectrum (Thin Film, NaCl) of compound **12**.

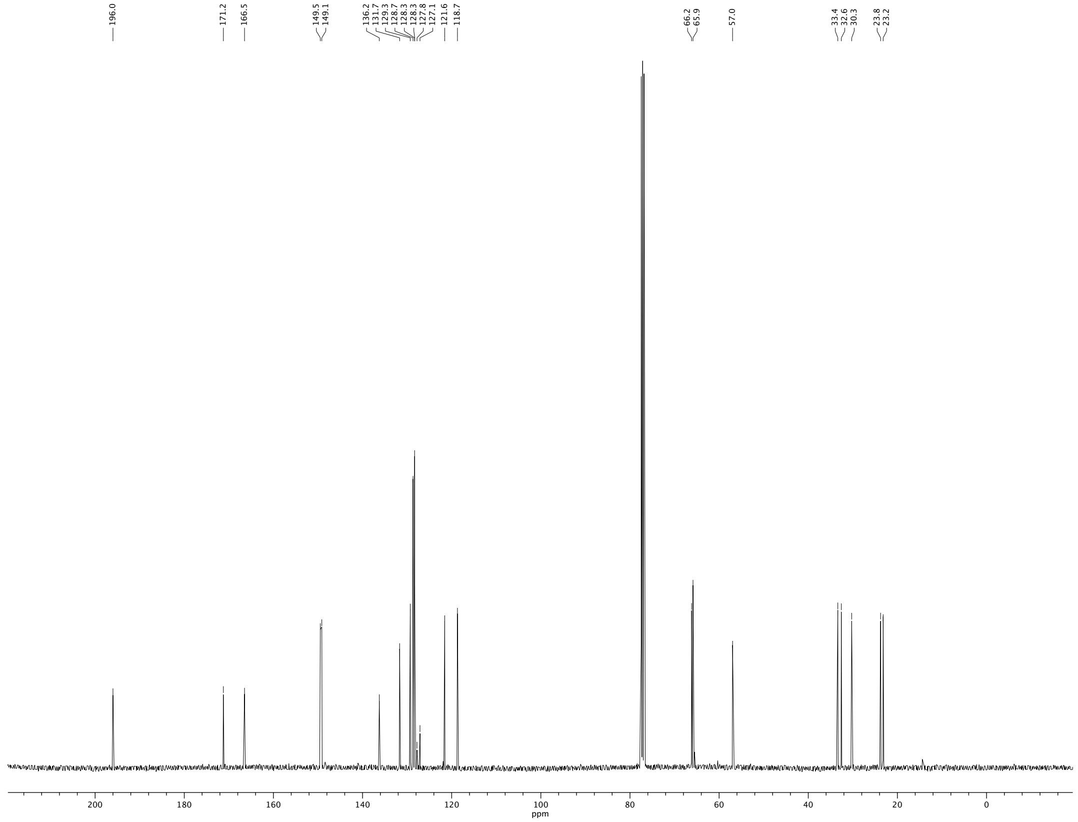

^{13}C NMR (100 MHz, $CDCl_3$) of compound **12**.

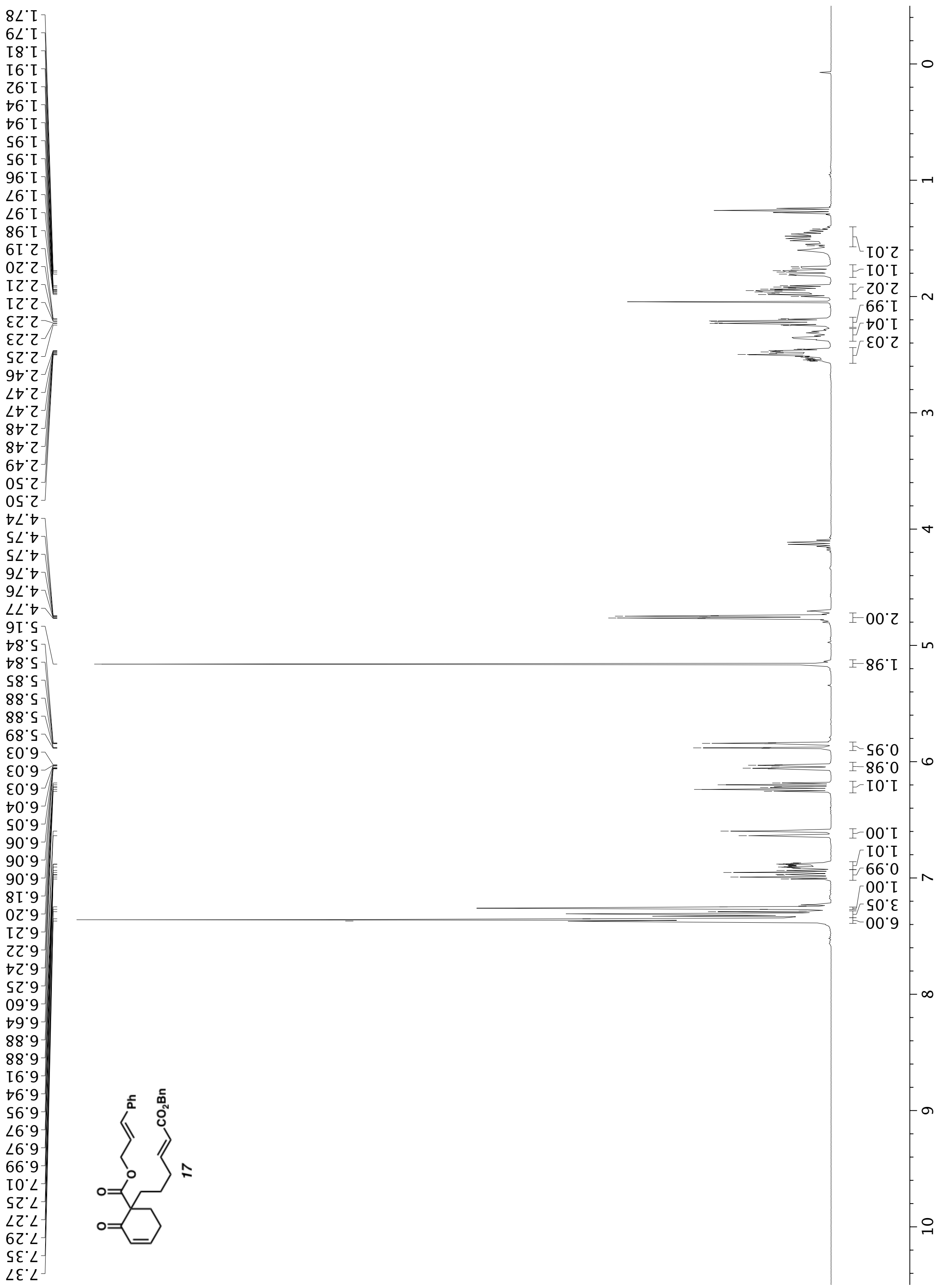

^{1}H NMR (400 MHz, $CDCl_3$) of compound **17.**

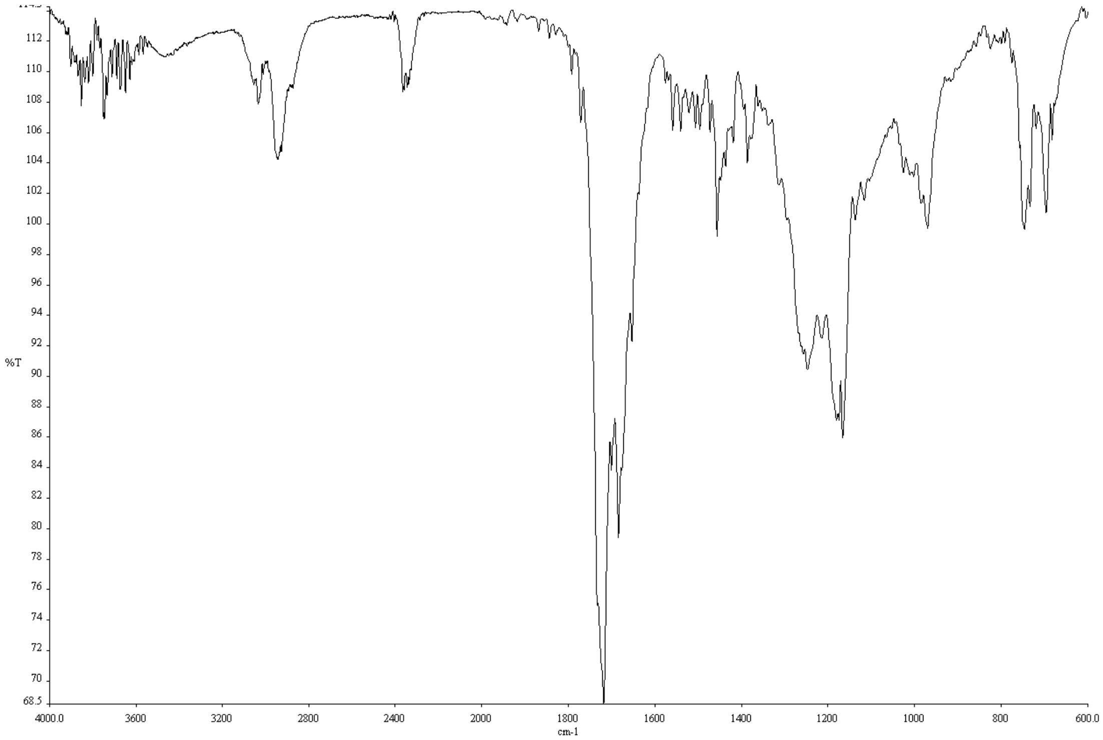

Infrared spectrum (Thin Film, NaCl) of compound **17**.

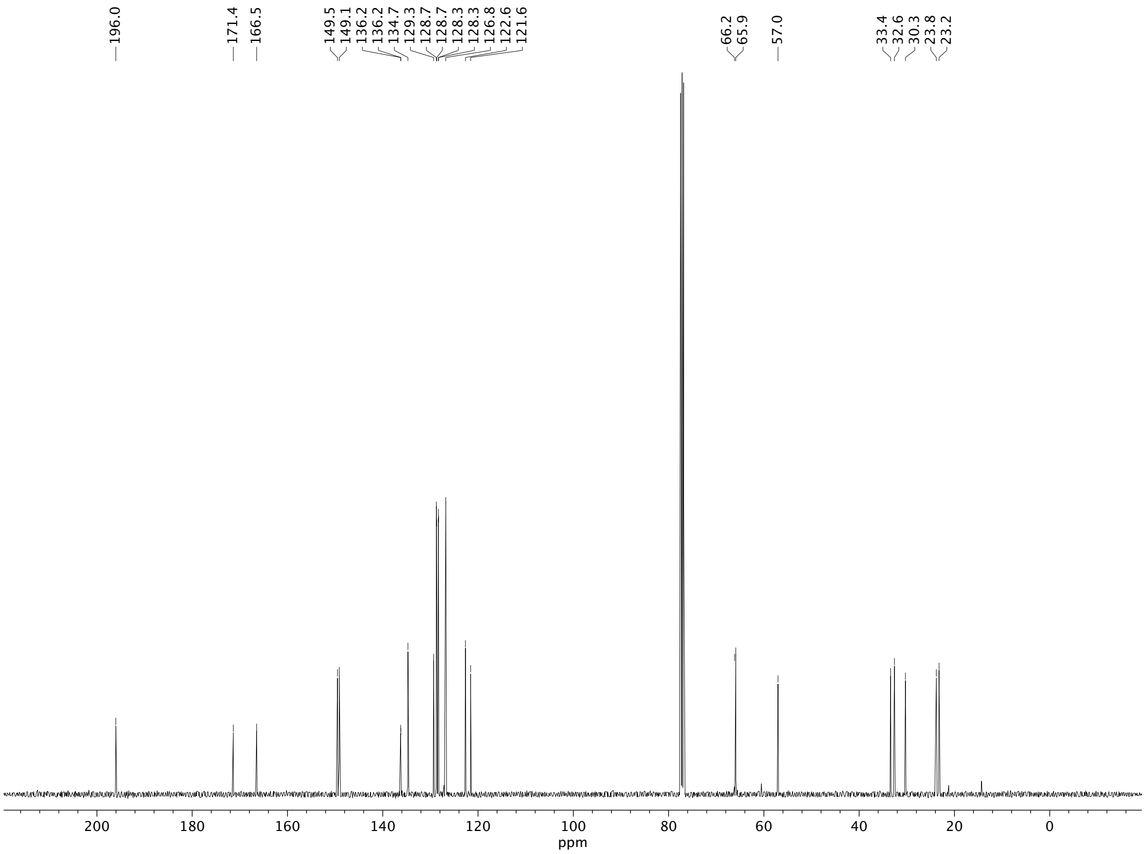

^{13}C NMR (100 MHz, $CDCl_3$) of compound **17**.

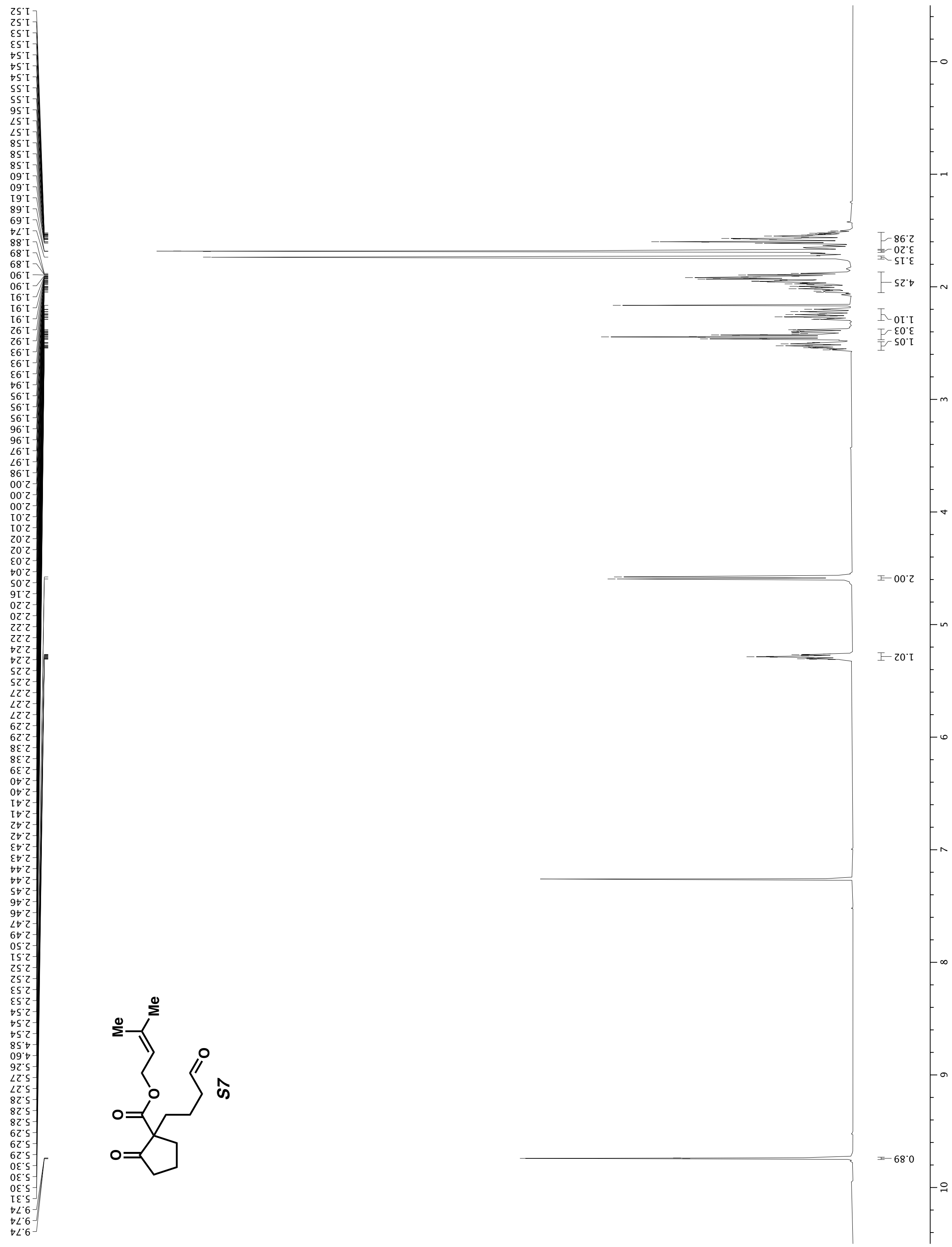

^{1}H NMR (400 MHz, $CDCl_3$) of compound **S7**.

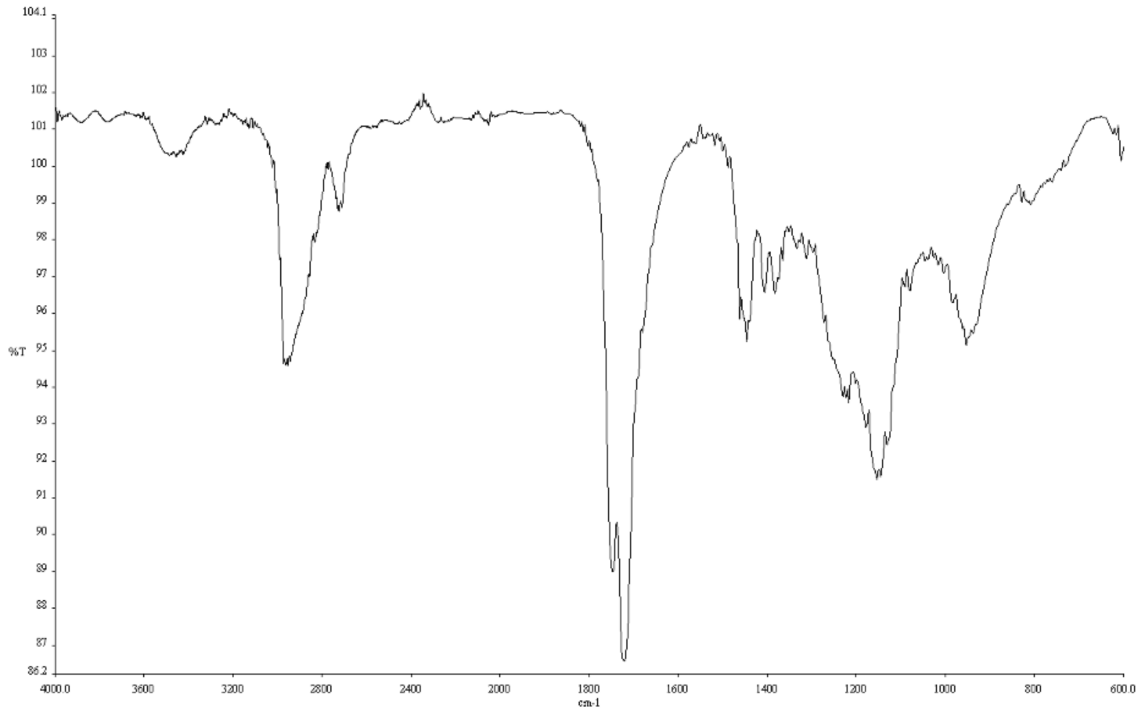

Infrared spectrum (Thin Film, NaCl) of compound **S7**.

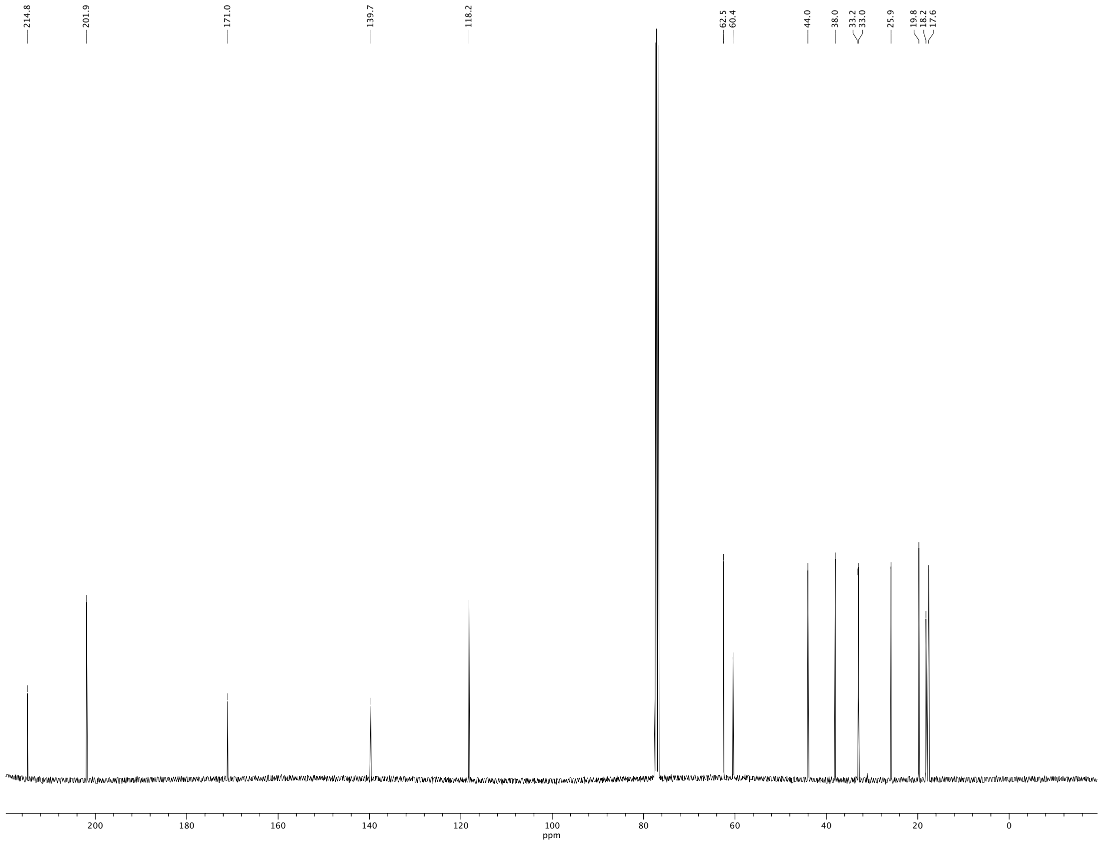

^{13}C NMR (100 MHz, $CDCl_3$) of compound **S7**.

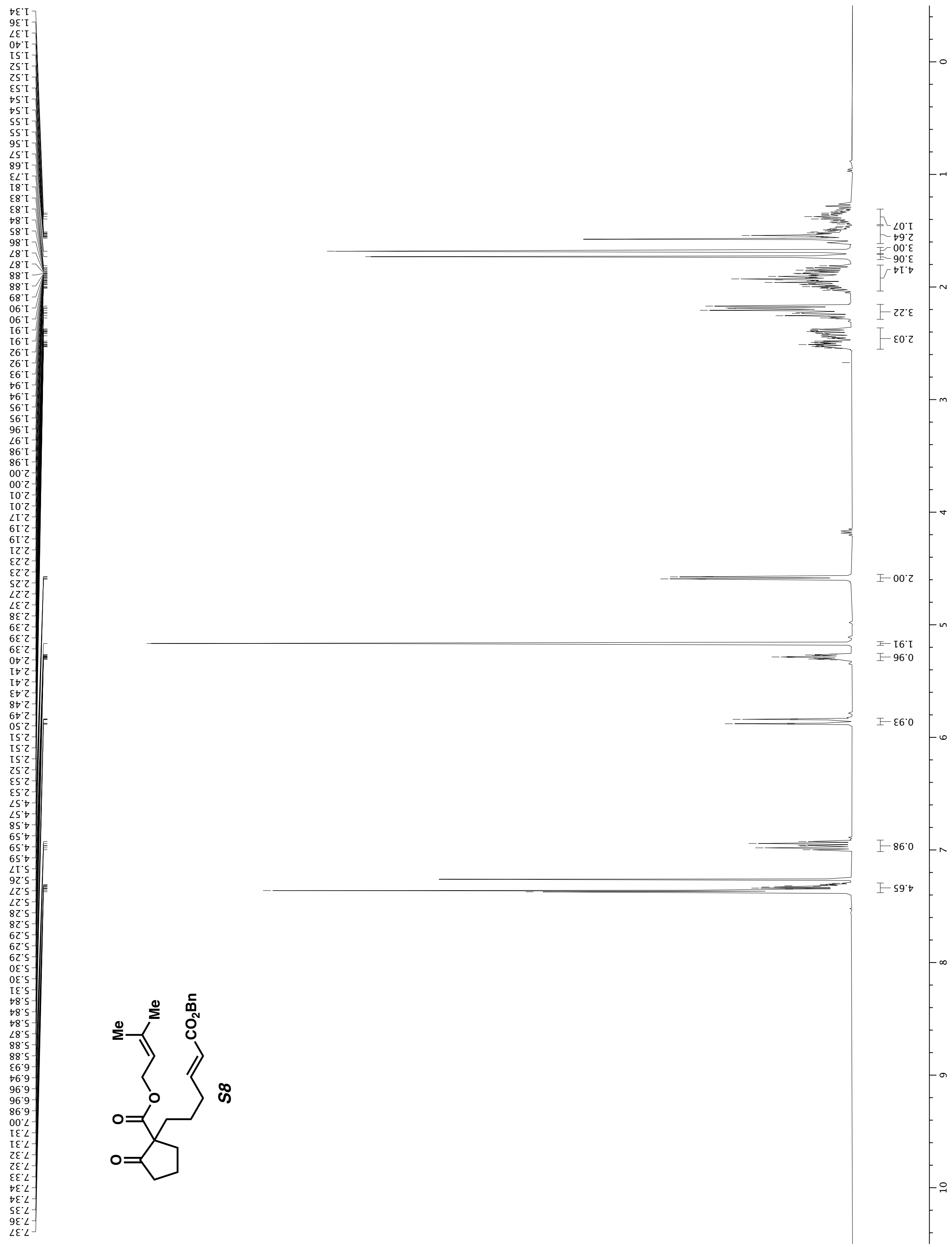

^{1}H NMR (400 MHz, $CDCl_3$) of compound **S8**.

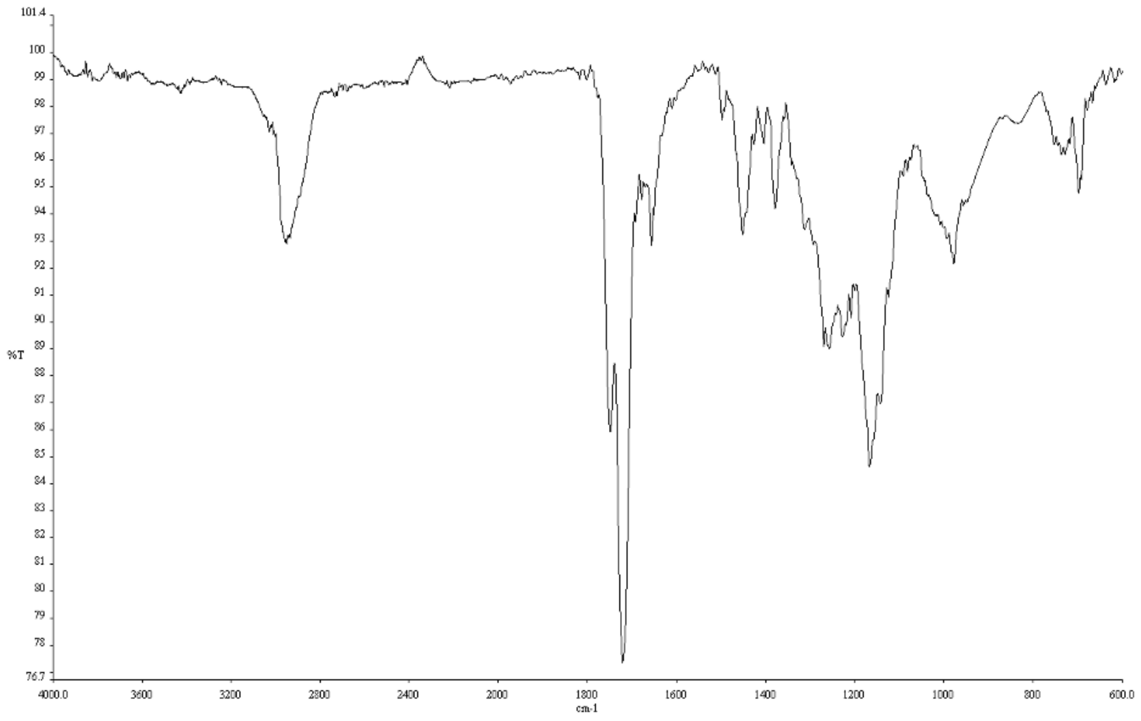

Infrared spectrum (Thin Film, NaCl) of compound **S8**.

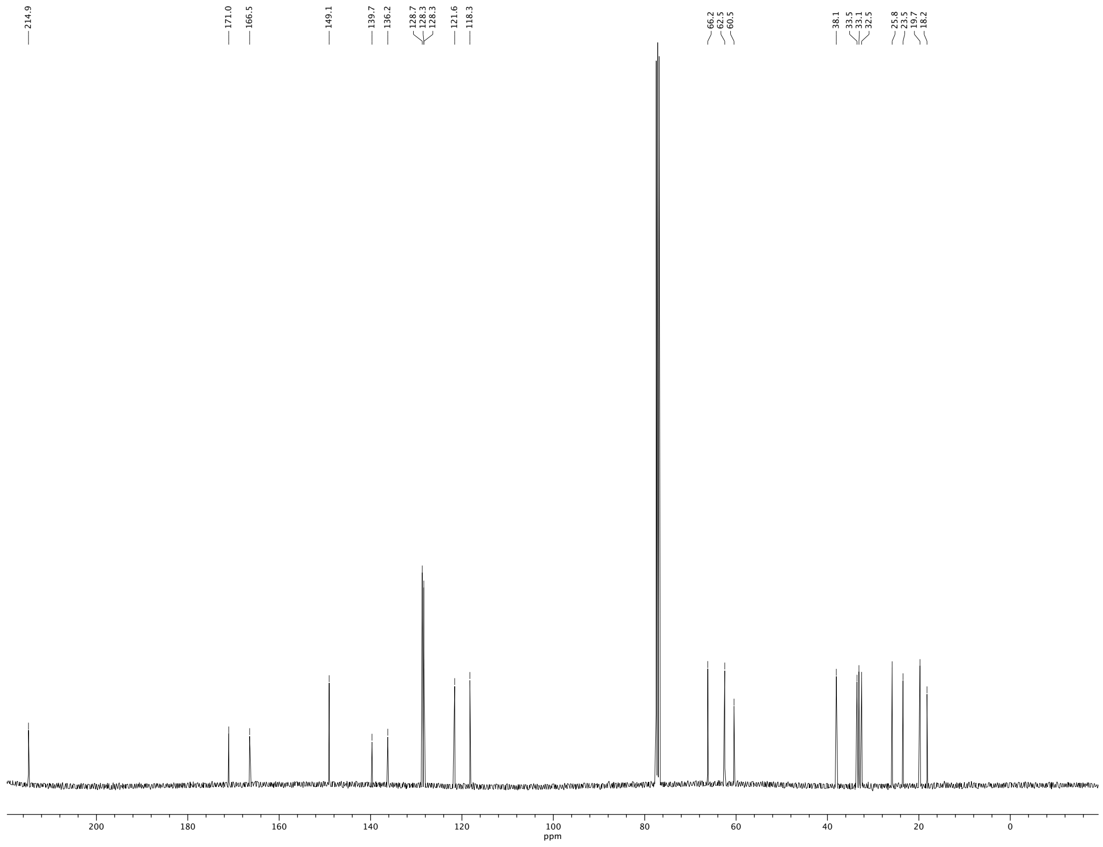

^{13}C NMR (100 MHz, $CDCl_3$) of compound **S8**.

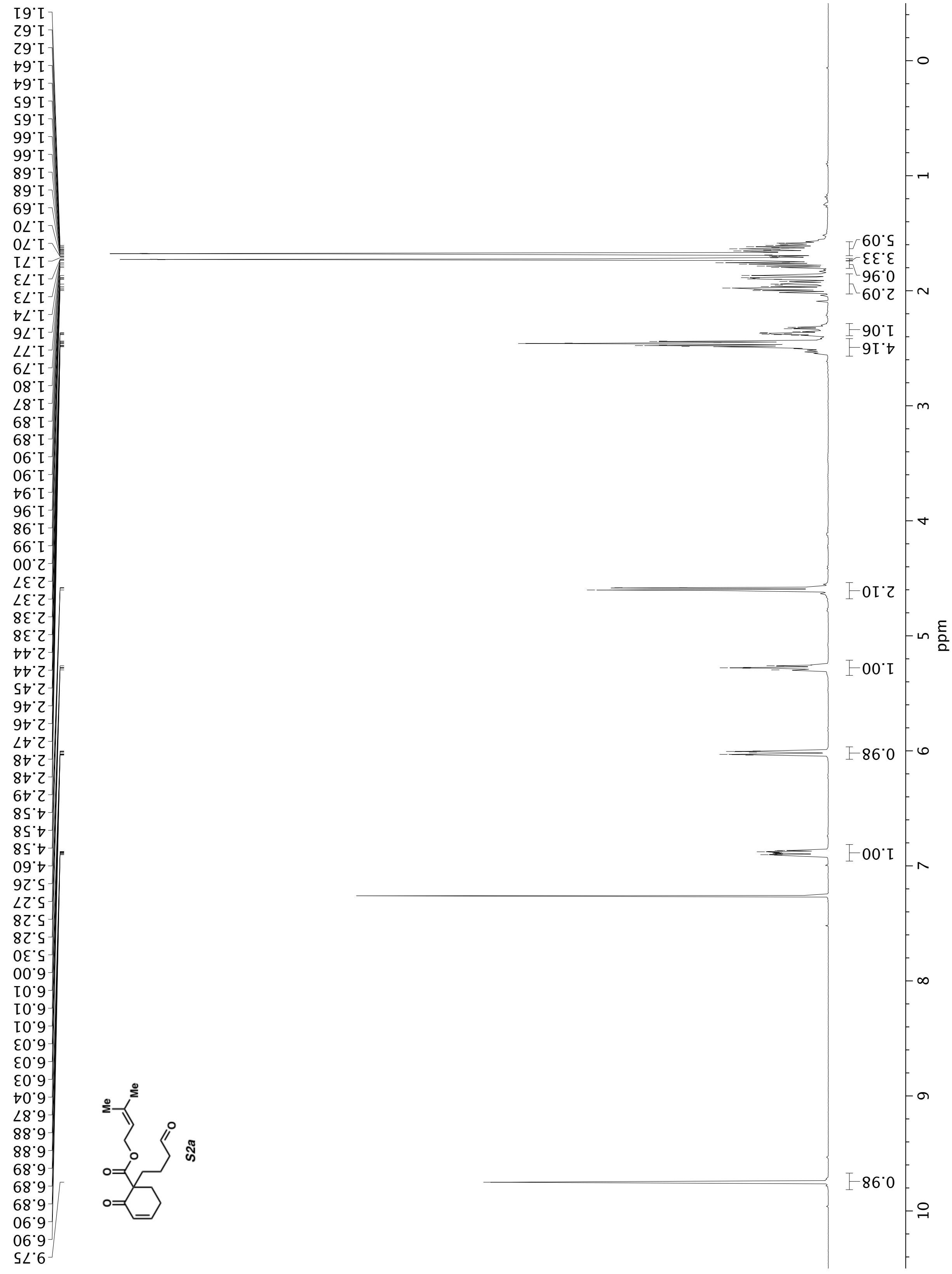

^{1}H NMR (400 MHz, $CDCl_3$) of compound **S2a.**

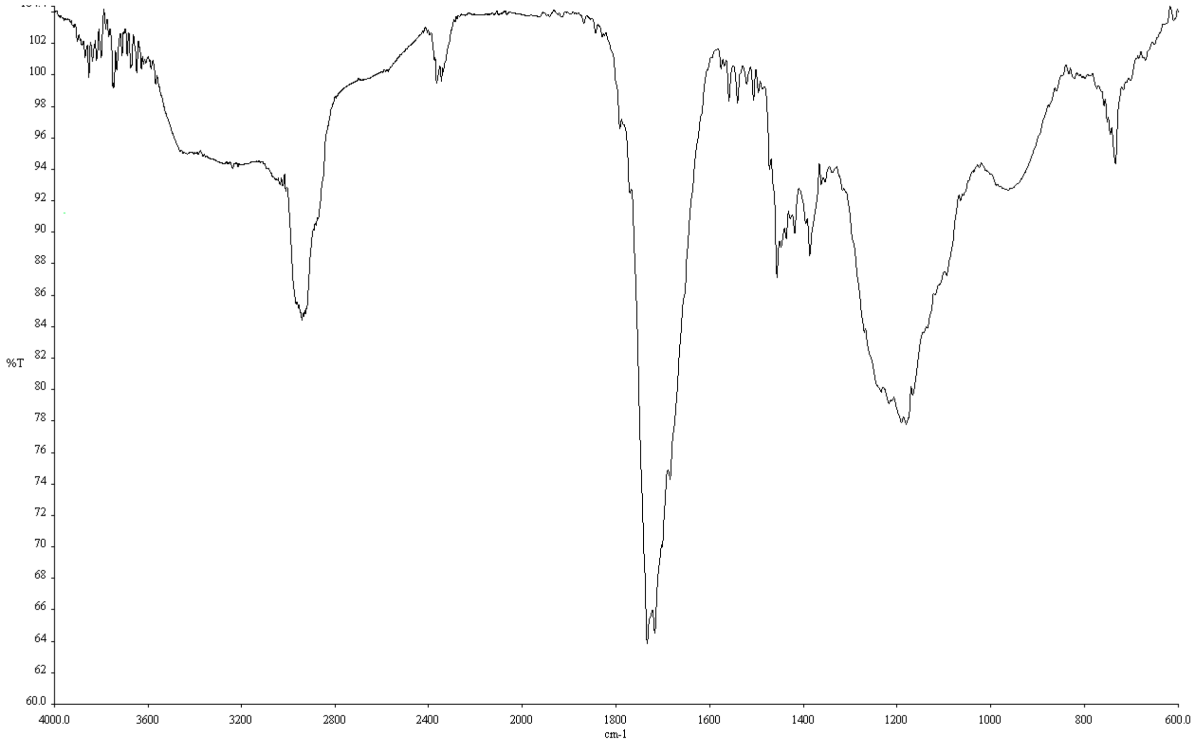

Infrared spectrum (Thin Film, NaCl) of compound **S2a**.

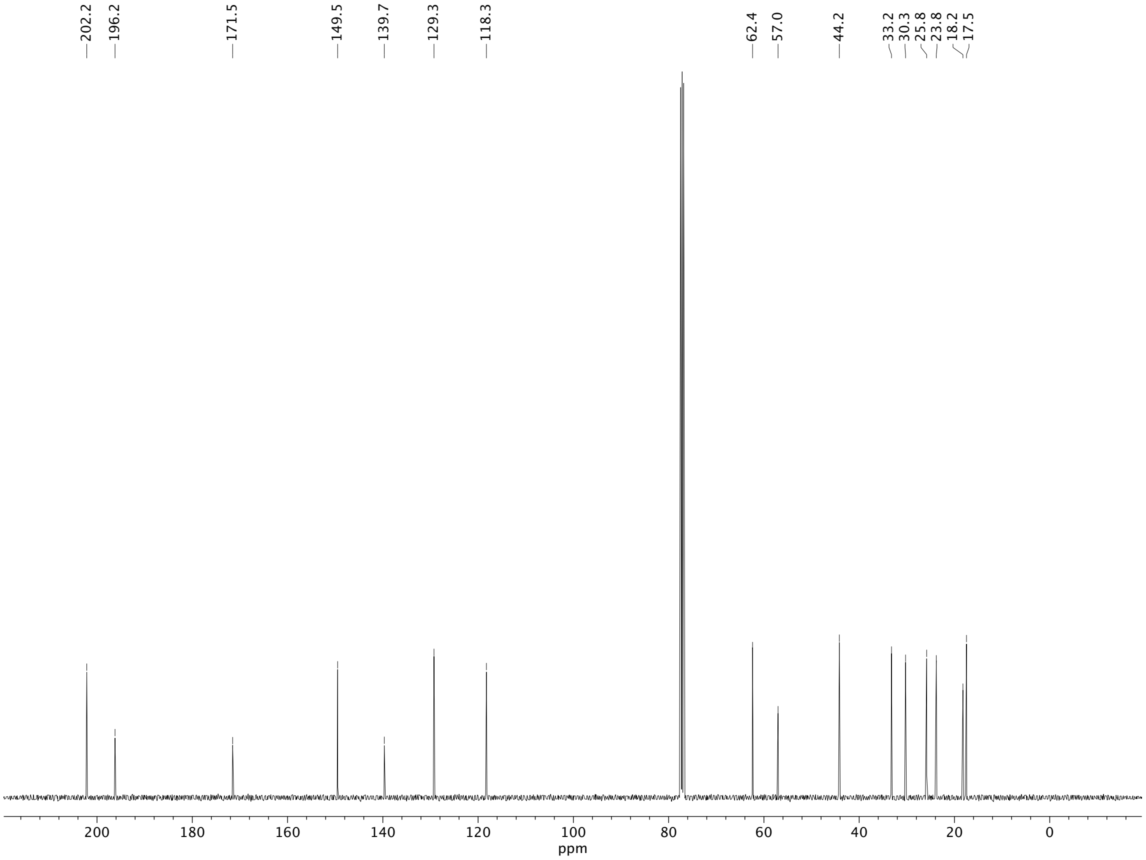

^{13}C NMR (100 MHz, $CDCl_3$) of compound **S2a**.

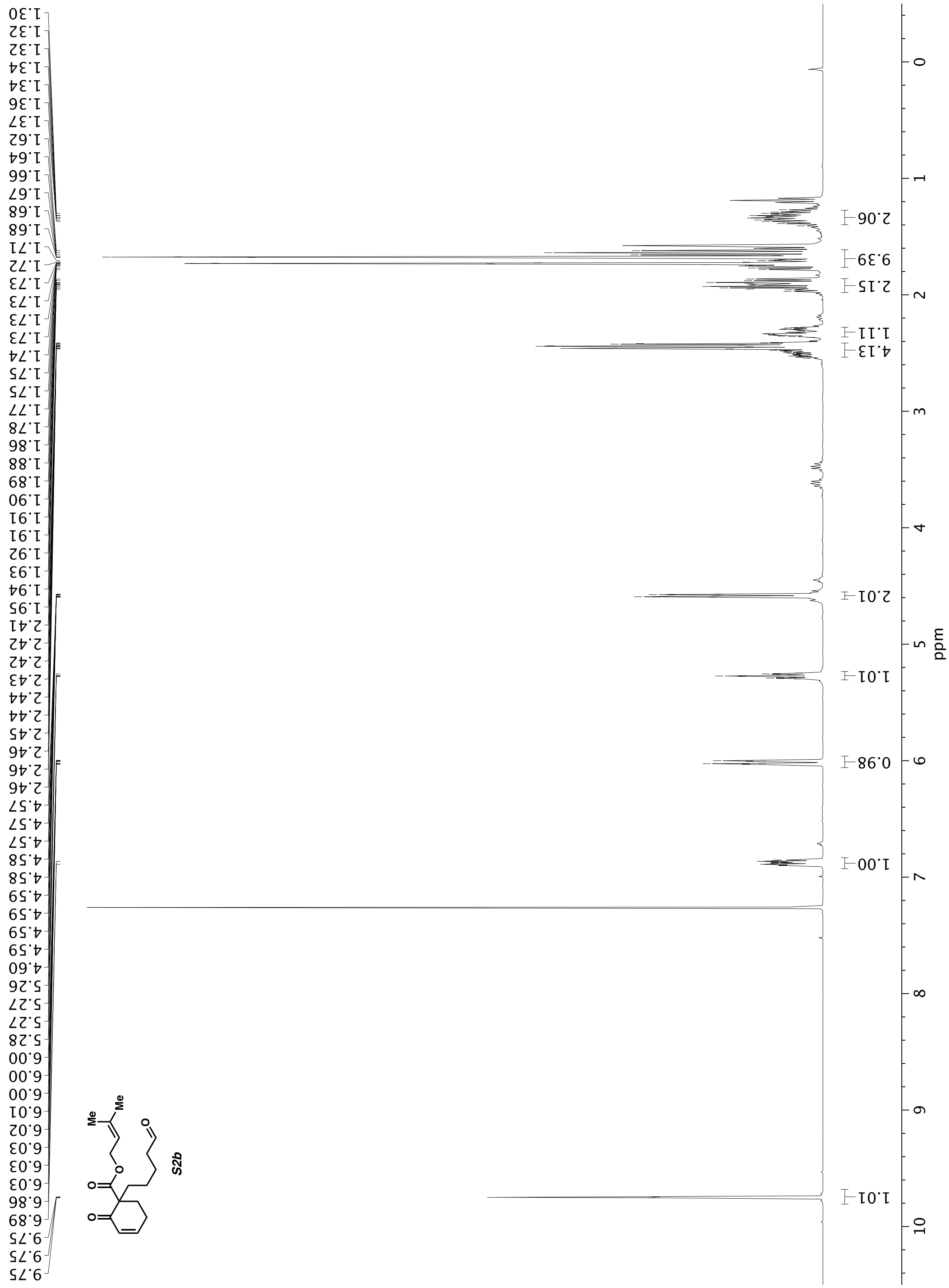

^{1}H NMR (400 MHz, $CDCl_3$) of compound **S2b.**

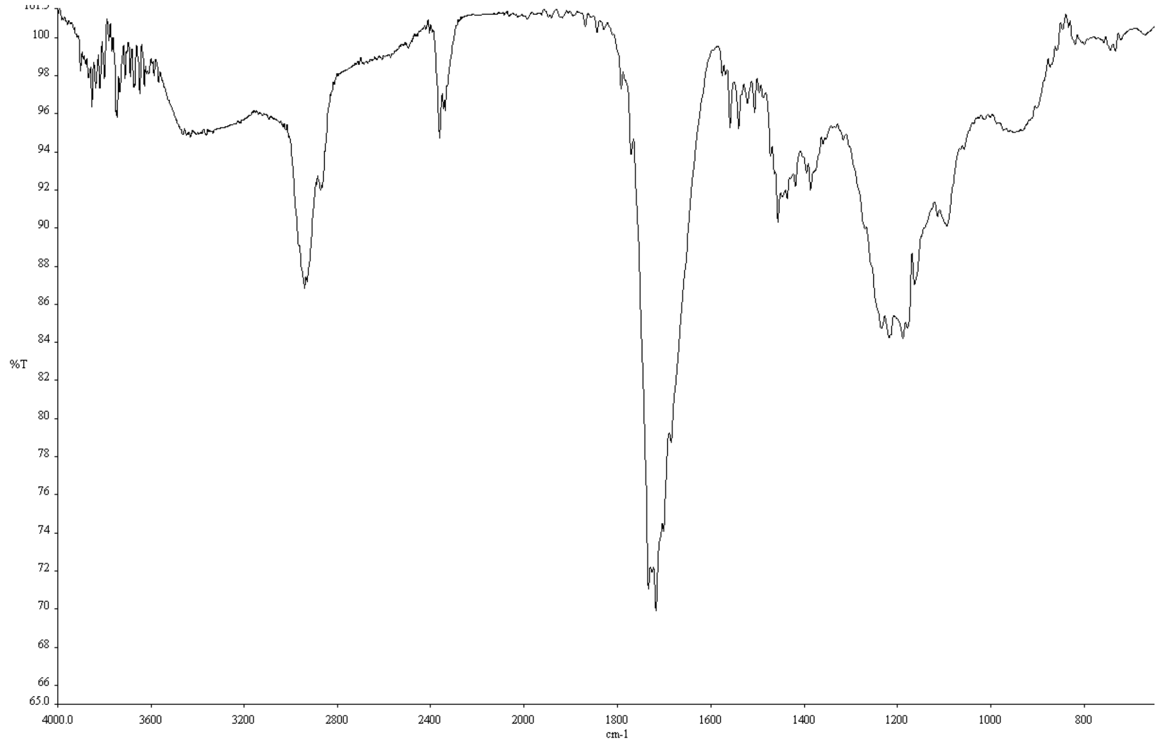

Infrared spectrum (Thin Film, NaCl) of compound **S2b**.

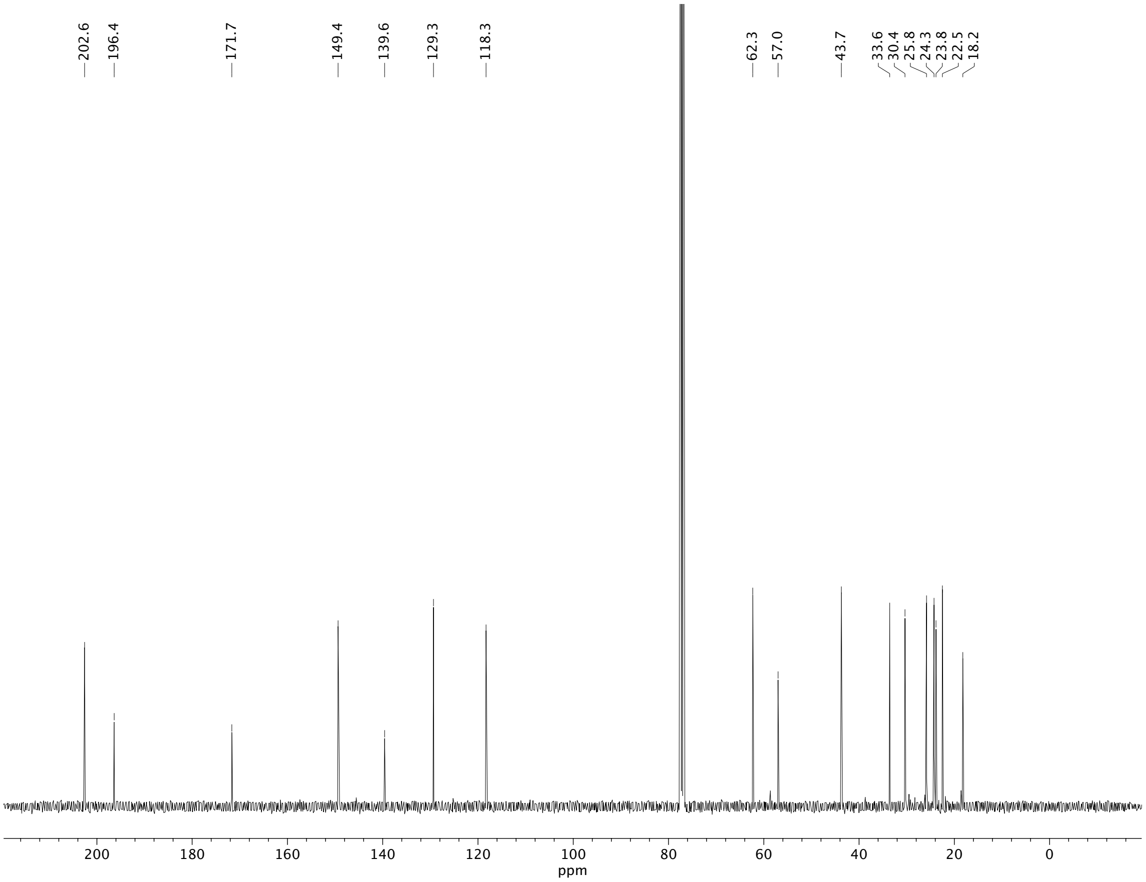

^{13}C NMR (100 MHz, $CDCl_3$) of compound **S2b**.

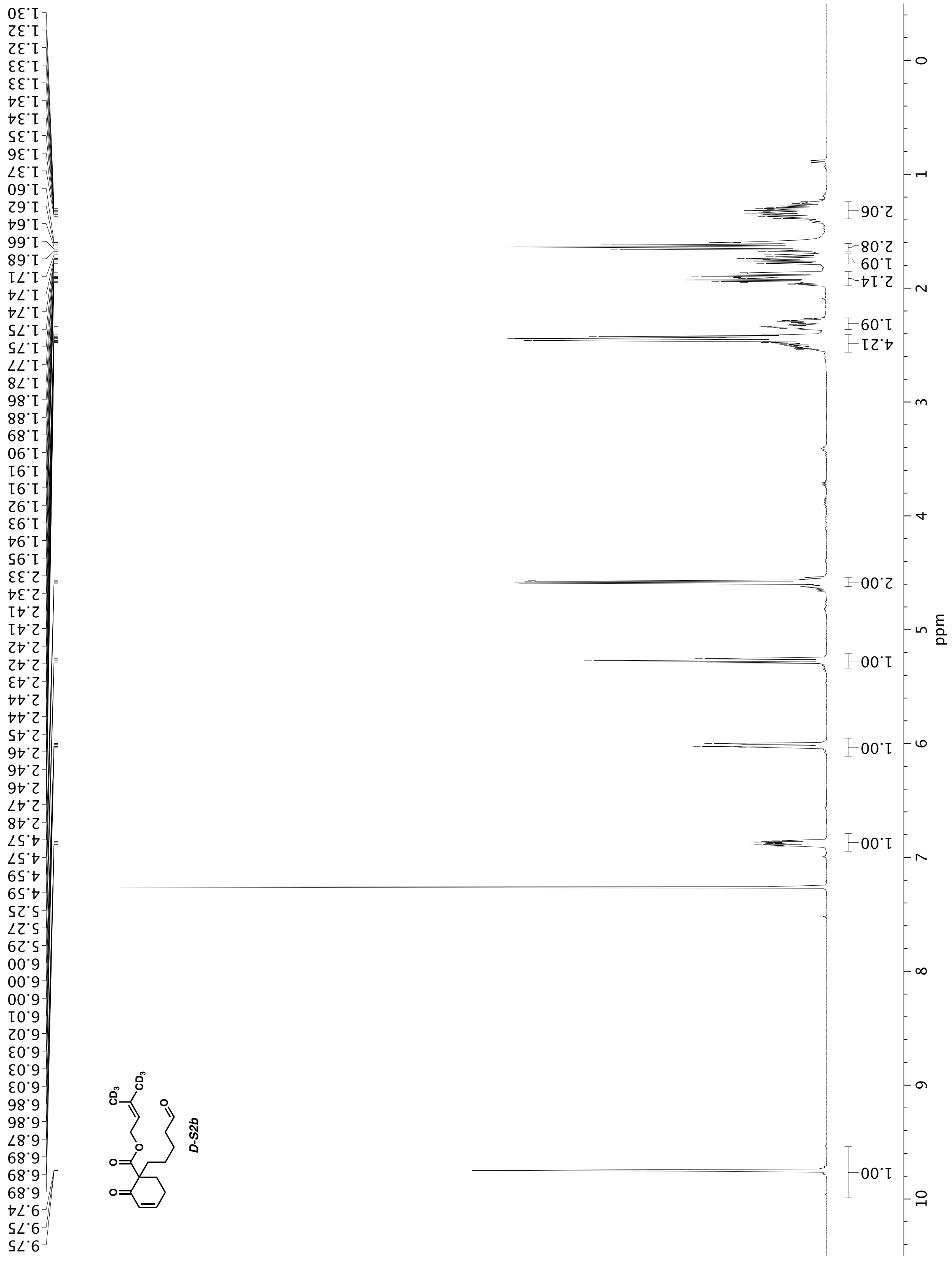

^{1}H NMR (400 MHz, $CDCl_3$) of compound ***D*-S2b.**

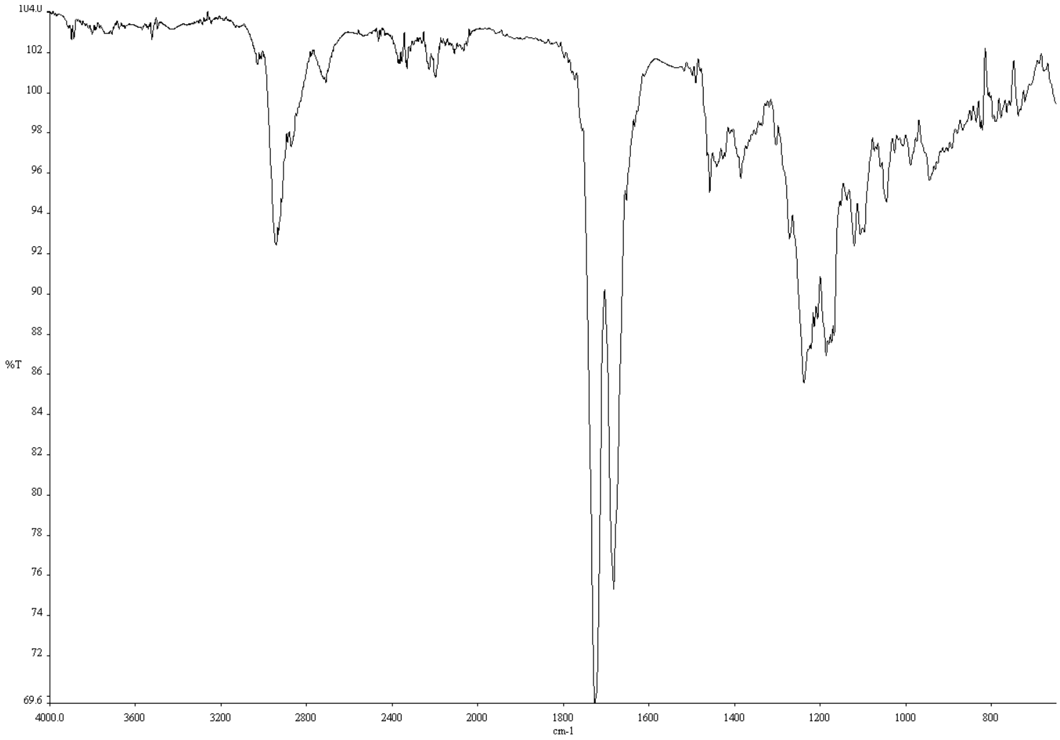

Infrared spectrum (Thin Film, NaCl) of compound ***D*-S2b**.

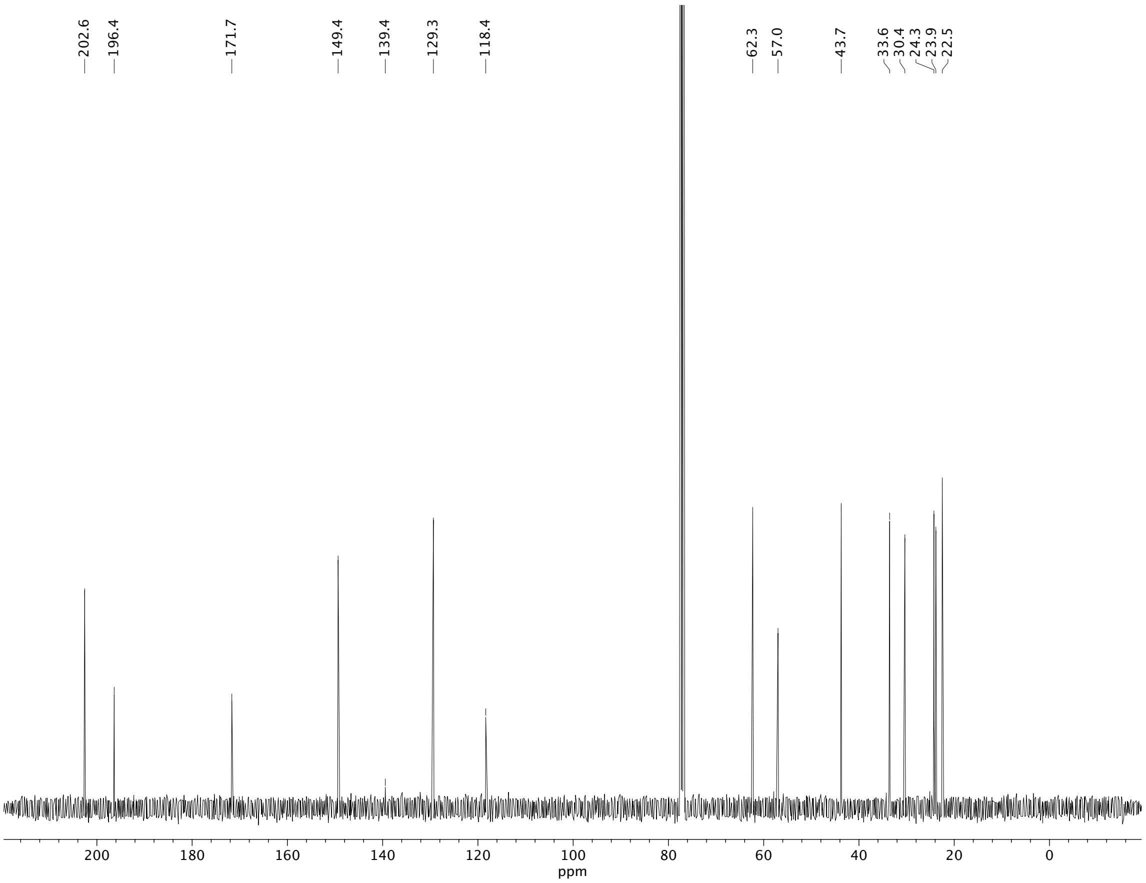

^{13}C NMR (100 MHz, $CDCl_3$) of compound ***D*-S2b**.

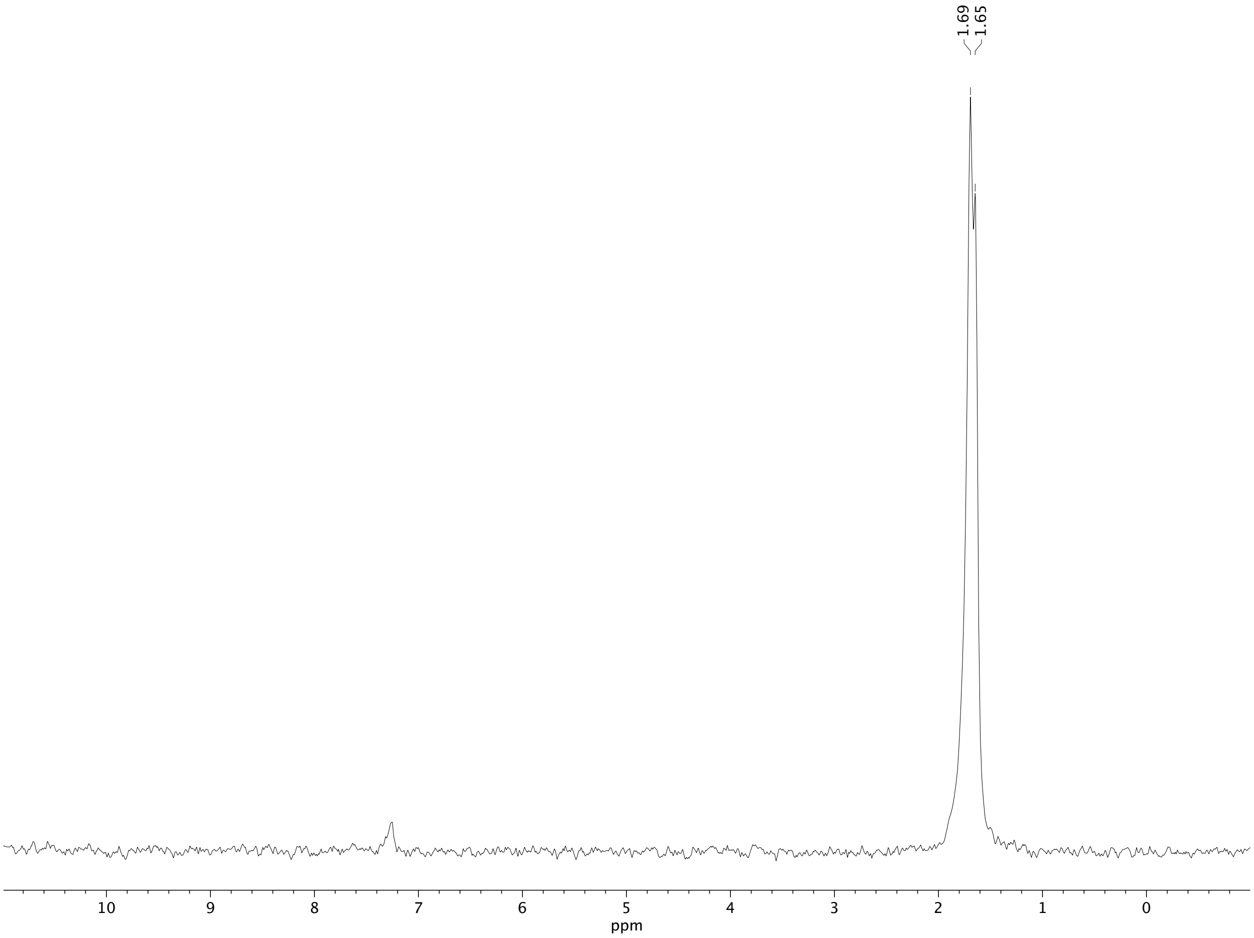

^{2}H NMR (61 MHz, $CHCl_3$) of compound ***D*-S2b**.

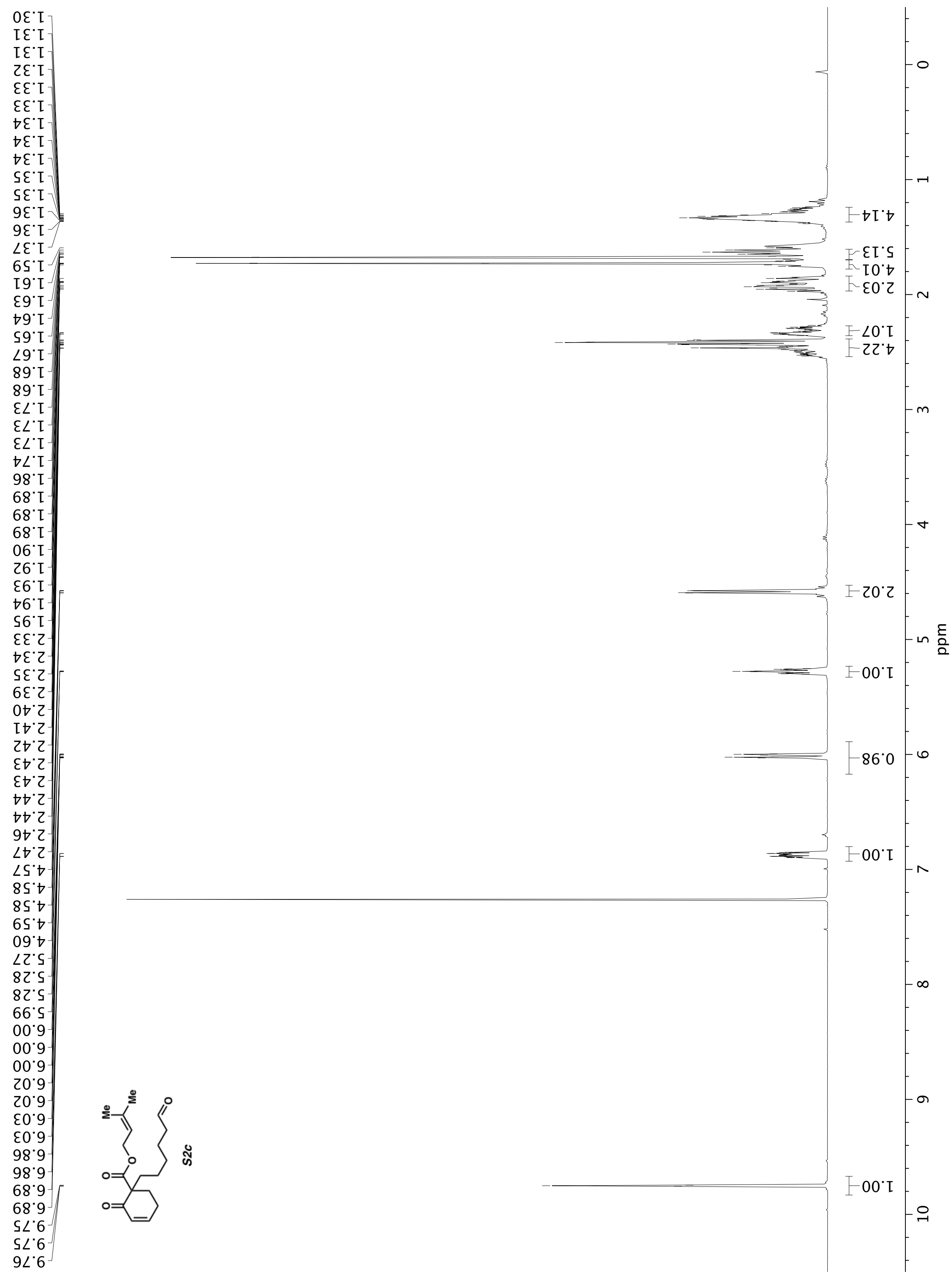

^{1}H NMR (400 MHz, CDCl$_3$) of compound **S2c.**

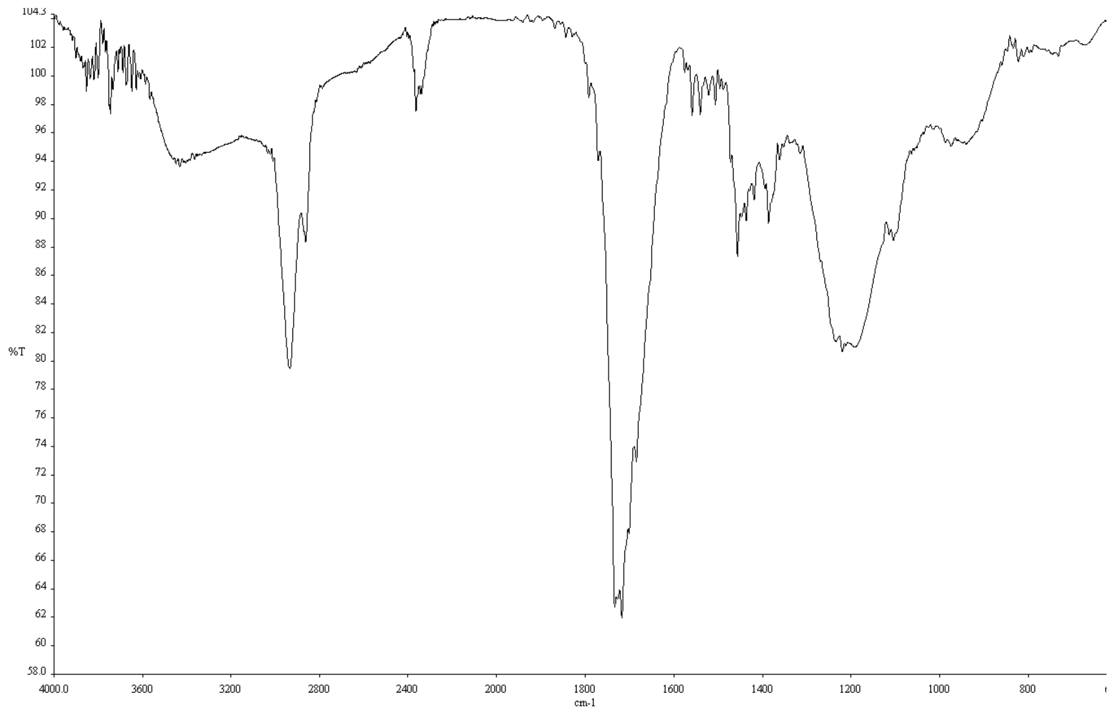

Infrared spectrum (Thin Film, NaCl) of compound **S2c**.

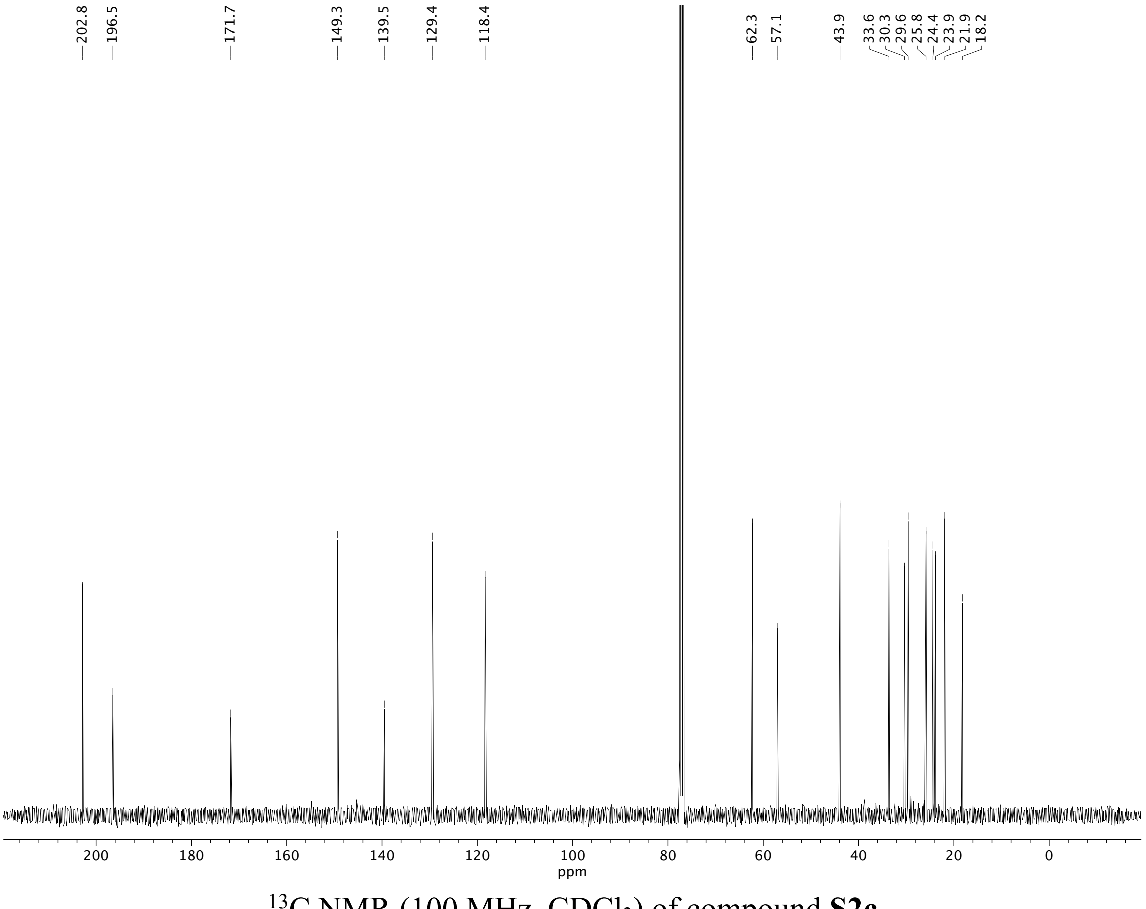

^{13}C NMR (100 MHz, $CDCl_3$) of compound **S2c**.

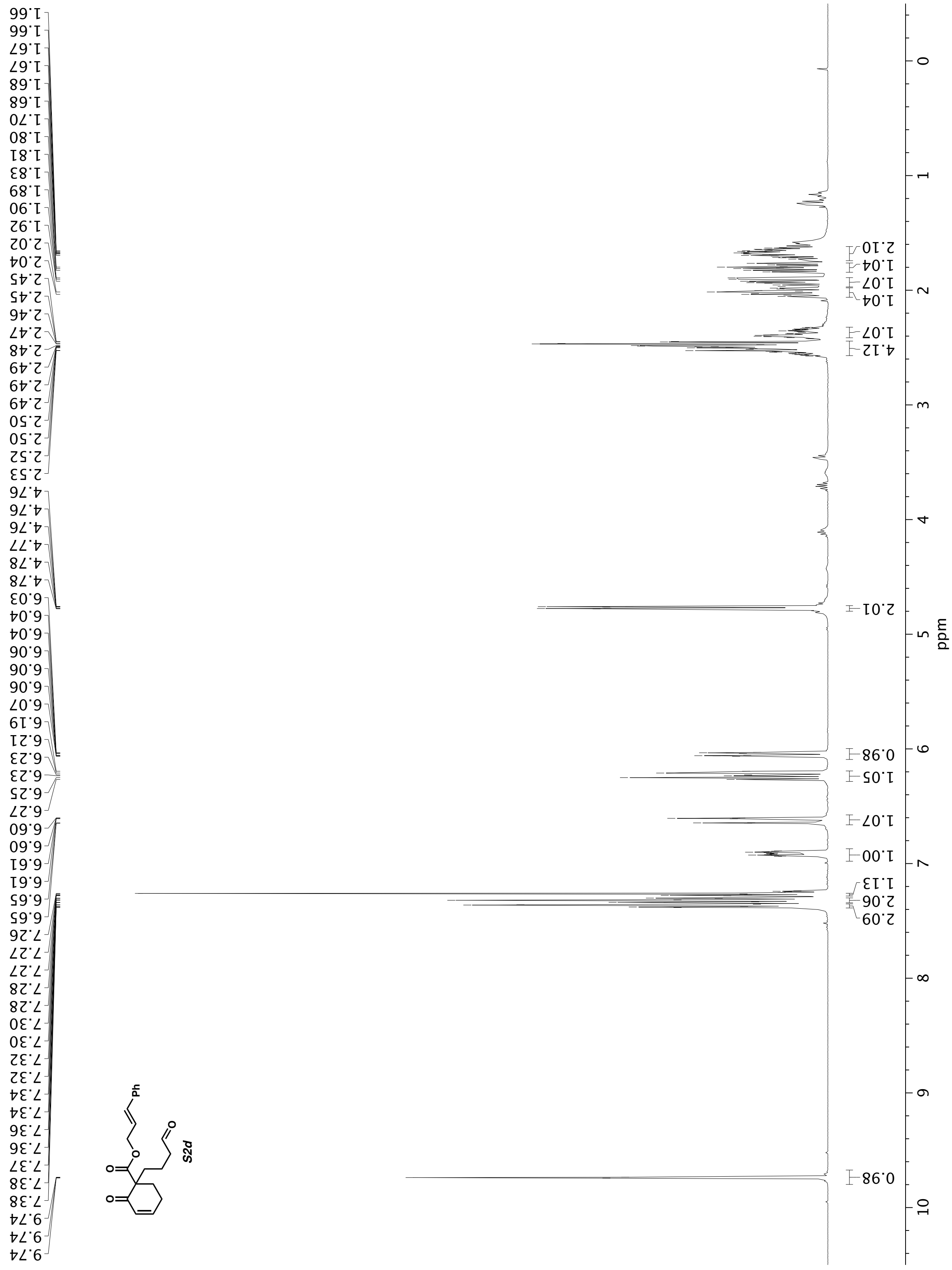

^{1}H NMR (400 MHz, $CDCl_3$) of compound **S2d.**

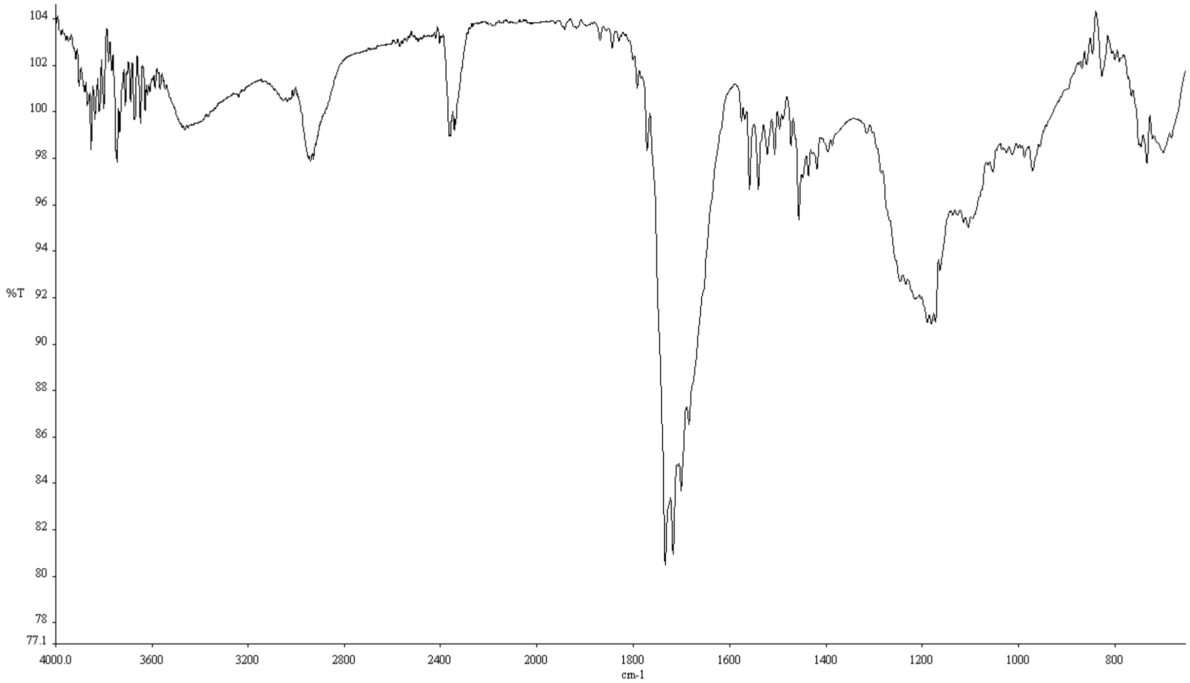

Infrared spectrum (Thin Film, NaCl) of compound **S2d**.

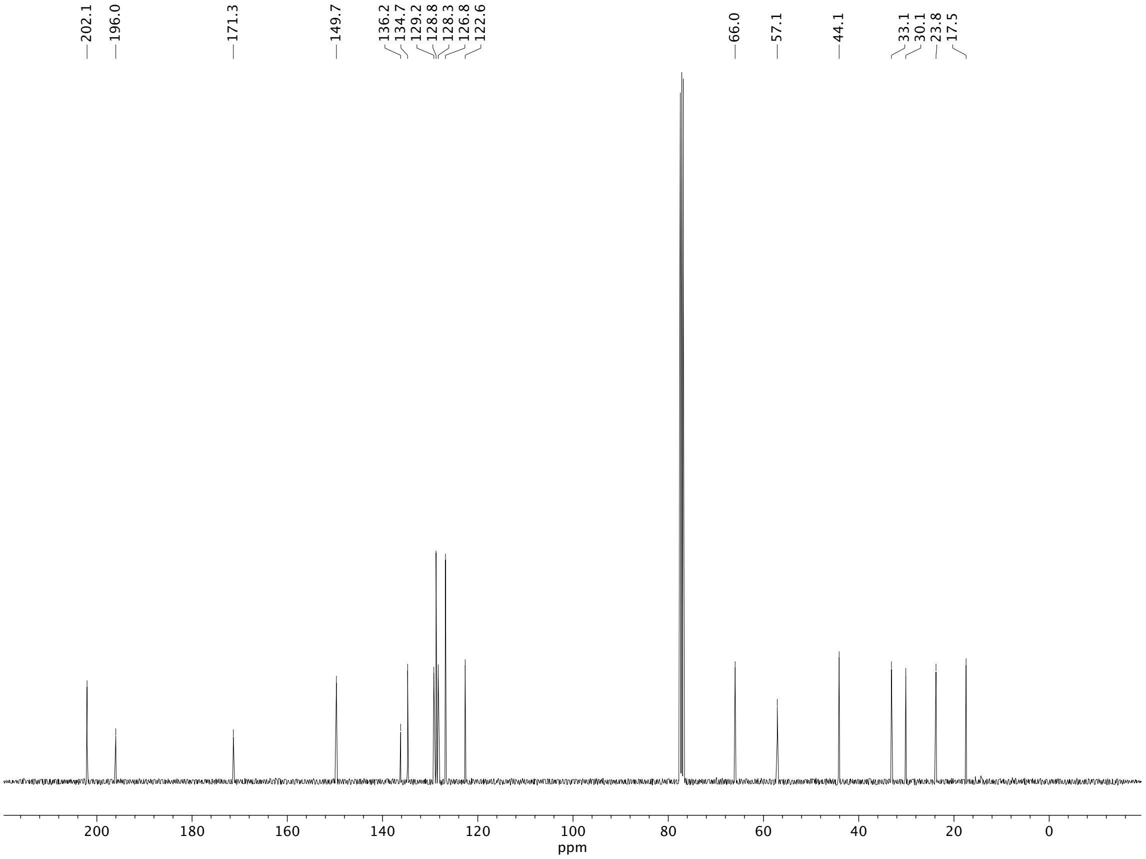

^{13}C NMR (100 MHz, $CDCl_3$) of compound **S2d**.

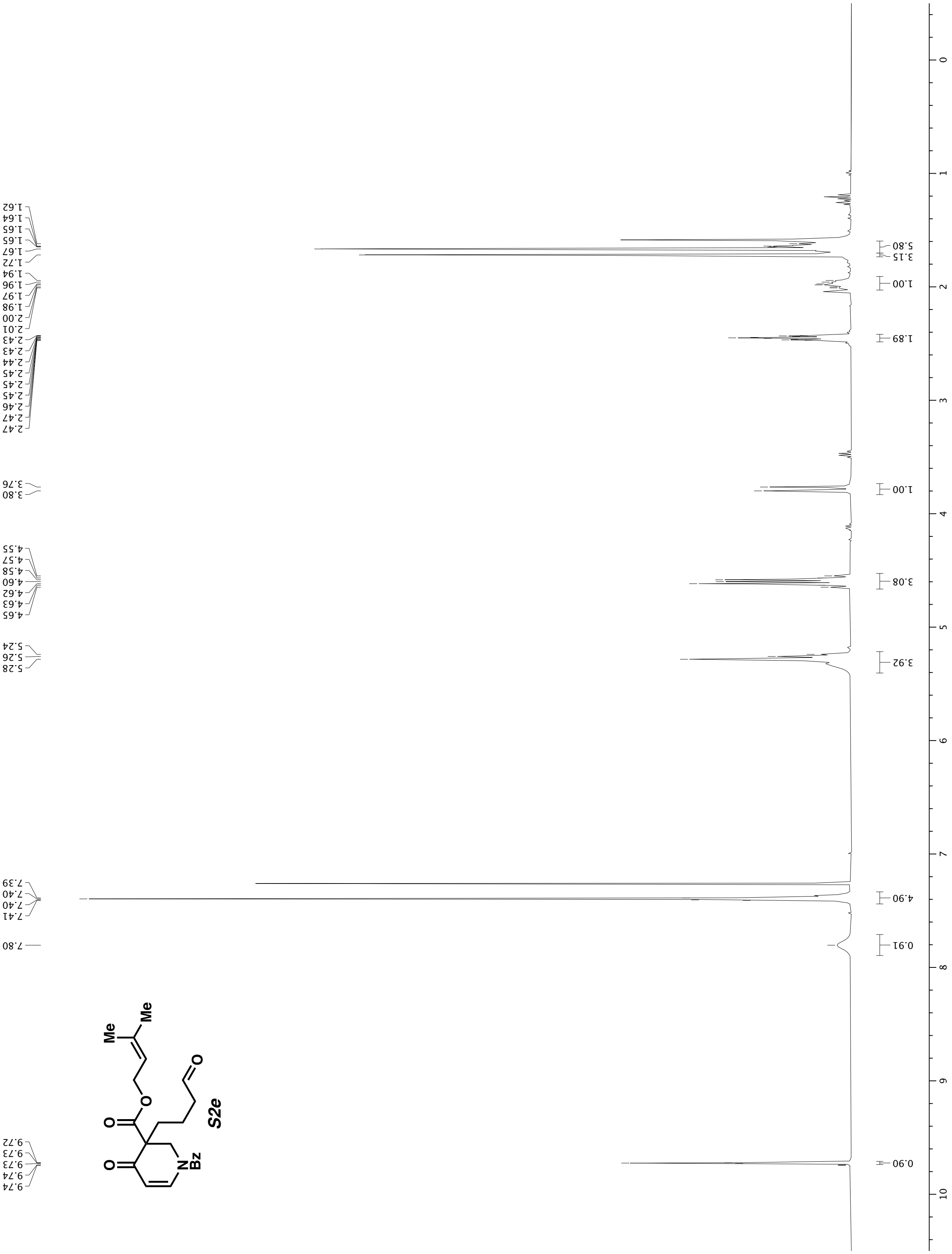

^{1}H NMR (400 MHz, $CDCl_3$) of compound **S2e**.

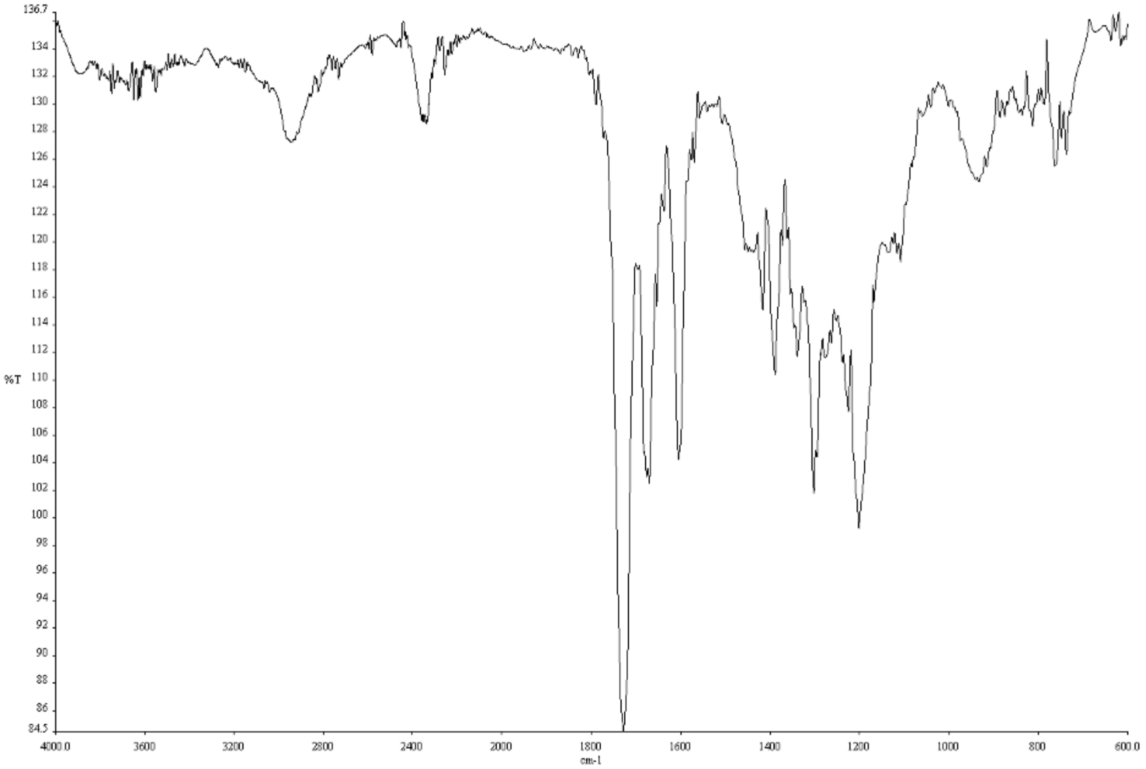

Infrared spectrum (Thin Film, NaCl) of compound **S2e**.

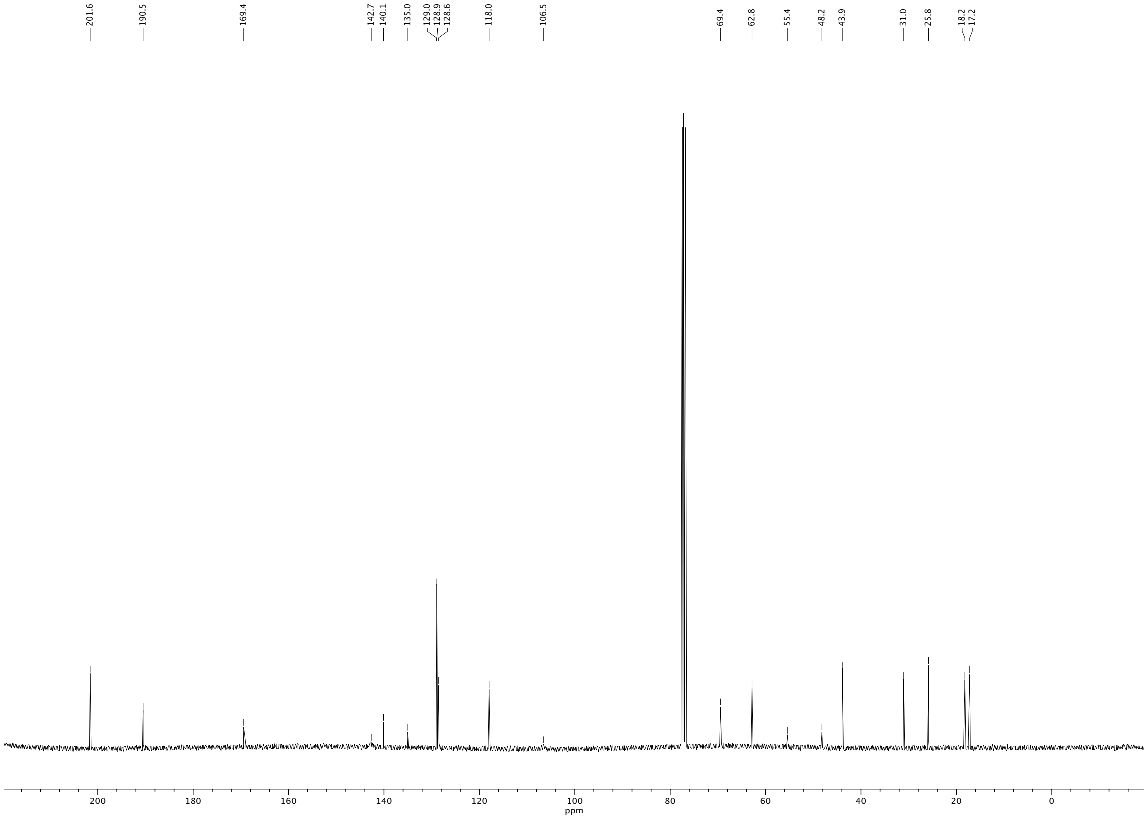

^{13}C NMR (100 MHz, $CDCl_3$) of compound **S2e**.

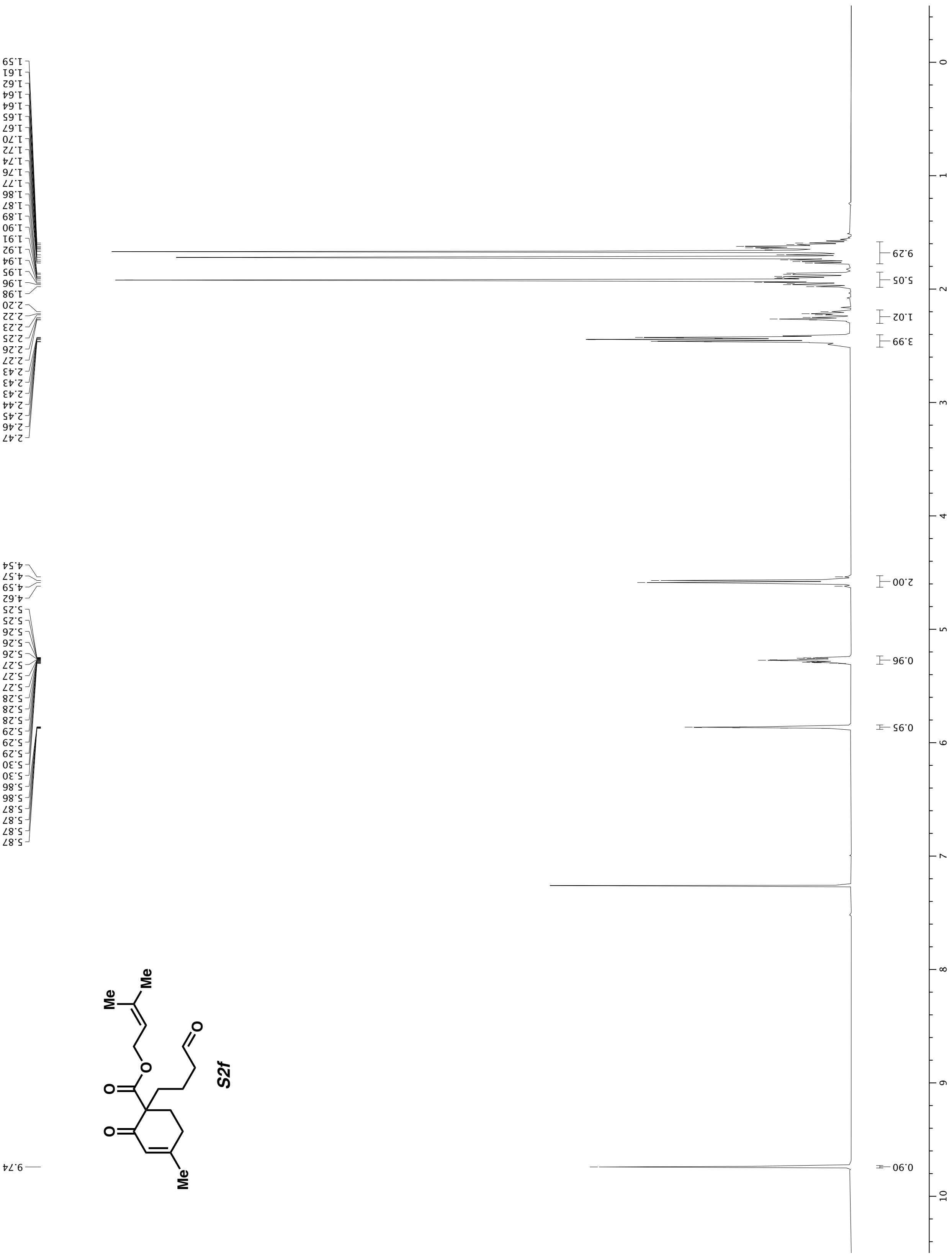

^{1}H NMR (400 MHz, $CDCl_3$) of compound **S2f**.

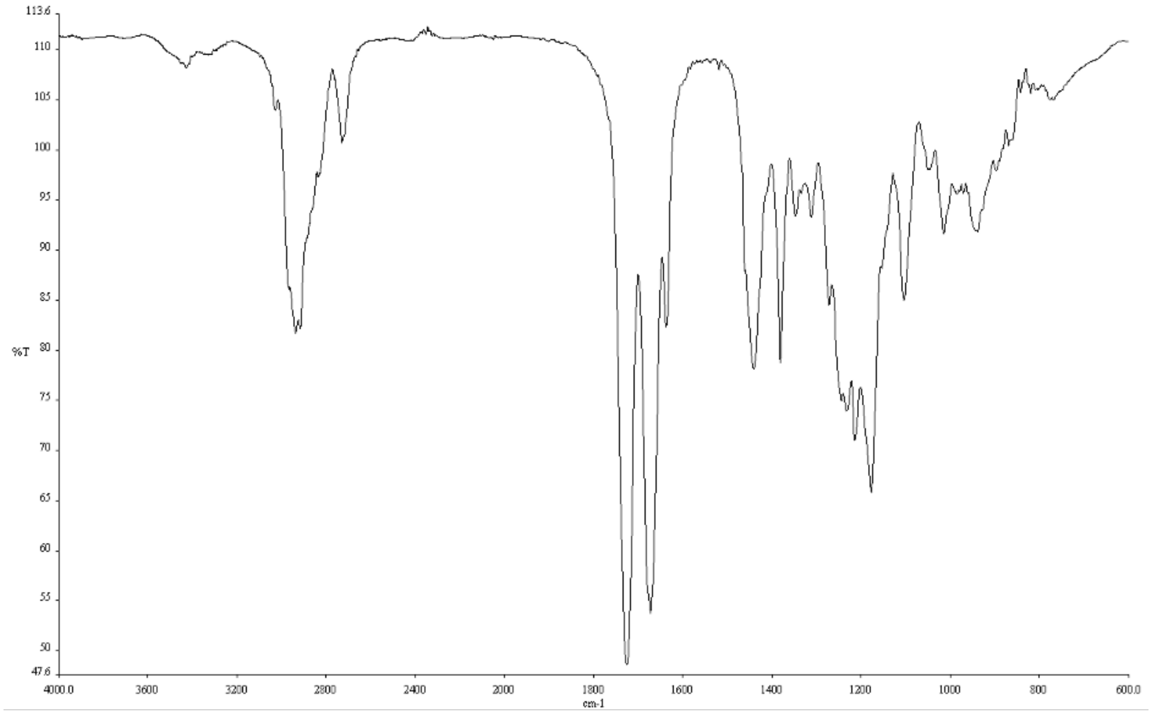

Infrared spectrum (Thin Film, NaCl) of compound **S2f**.

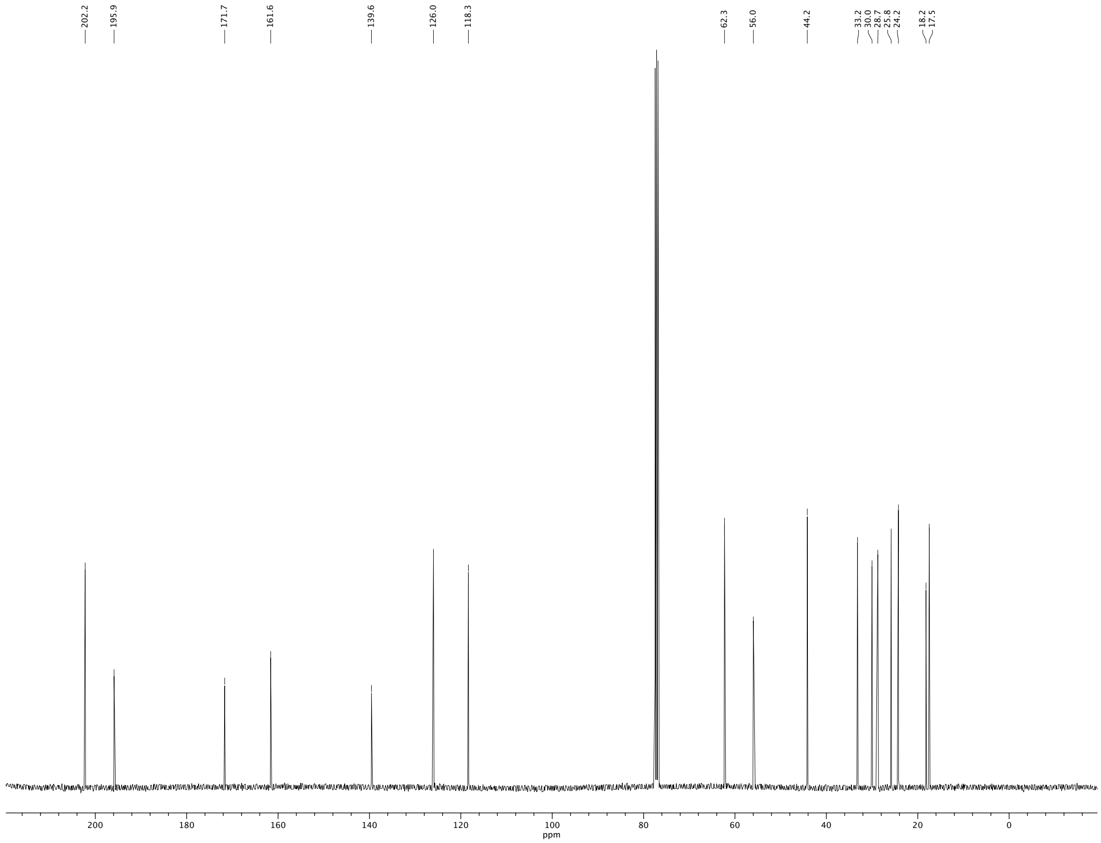

^{13}C NMR (100 MHz, $CDCl_3$) of compound **S2f**.

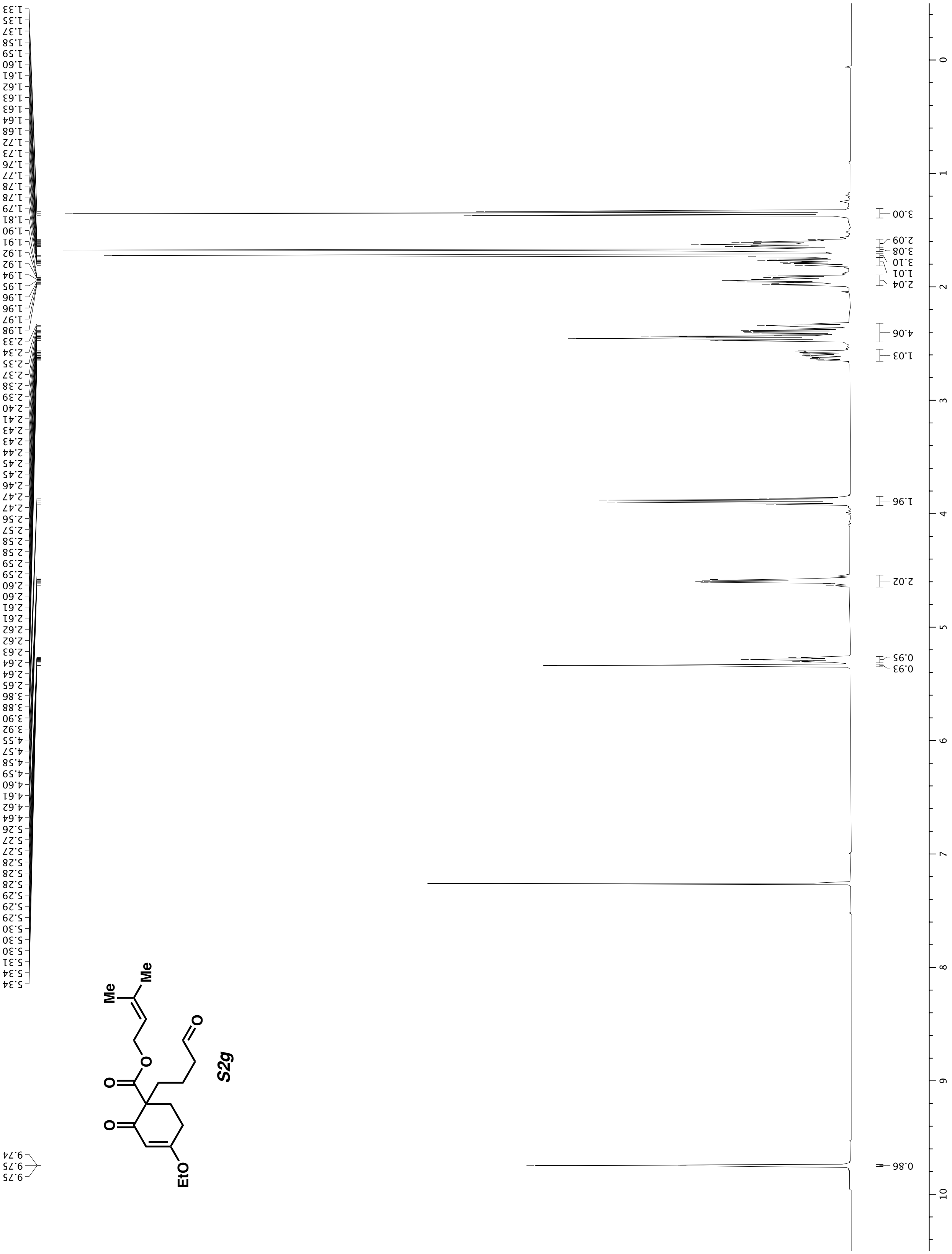

^1H NMR (400 MHz, $CDCl_3$) of compound **S2g**.

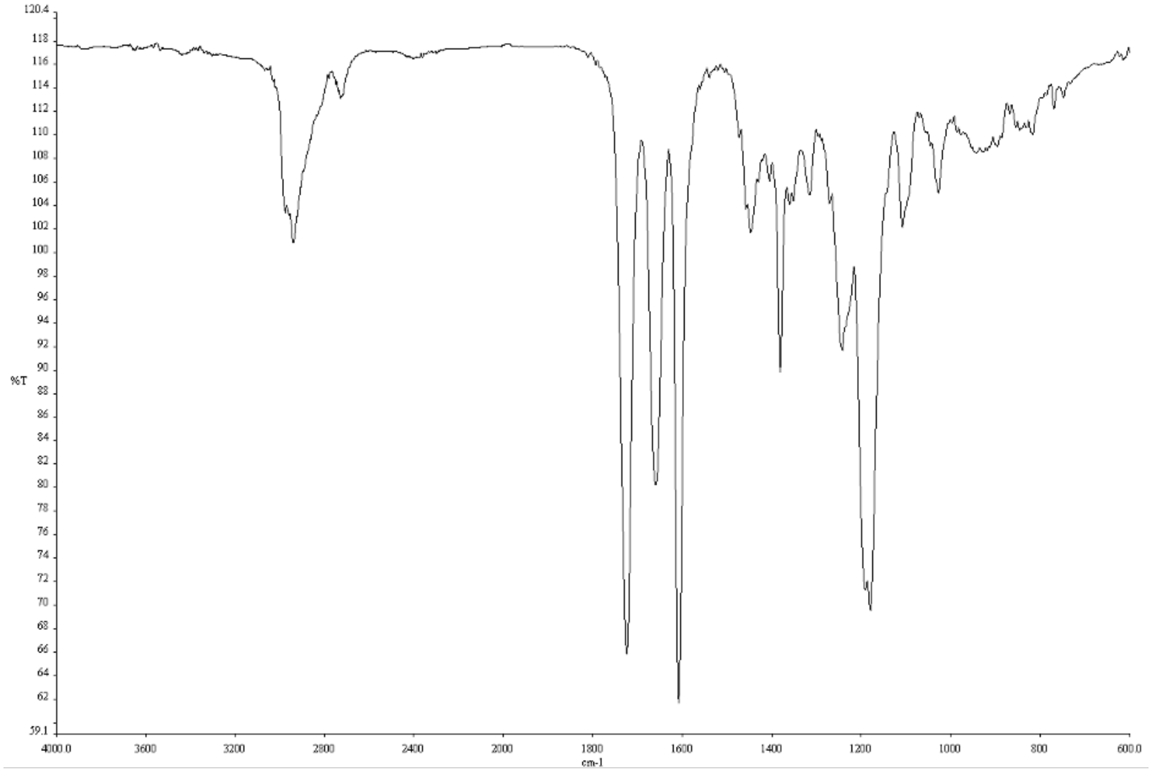

Infrared spectrum (Thin Film, NaCl) of compound **S2g**.

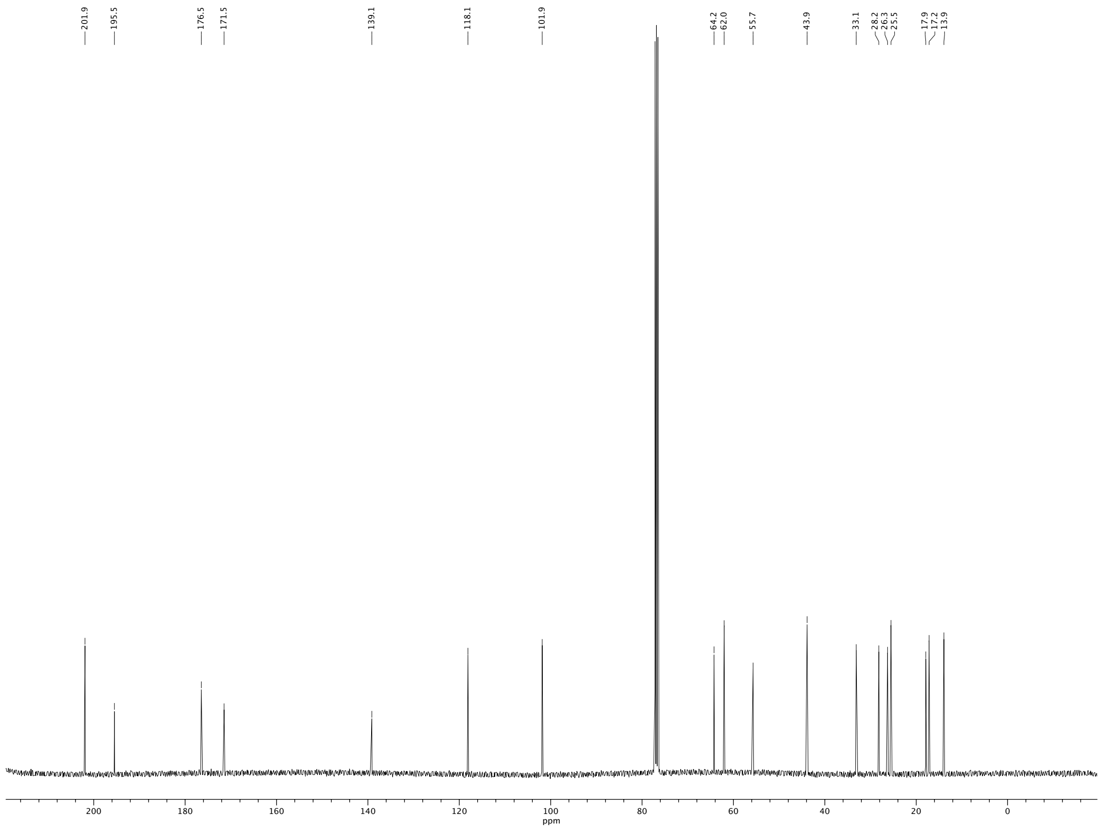

^{13}C NMR (100 MHz, $CDCl_3$) of compound **S2g**.

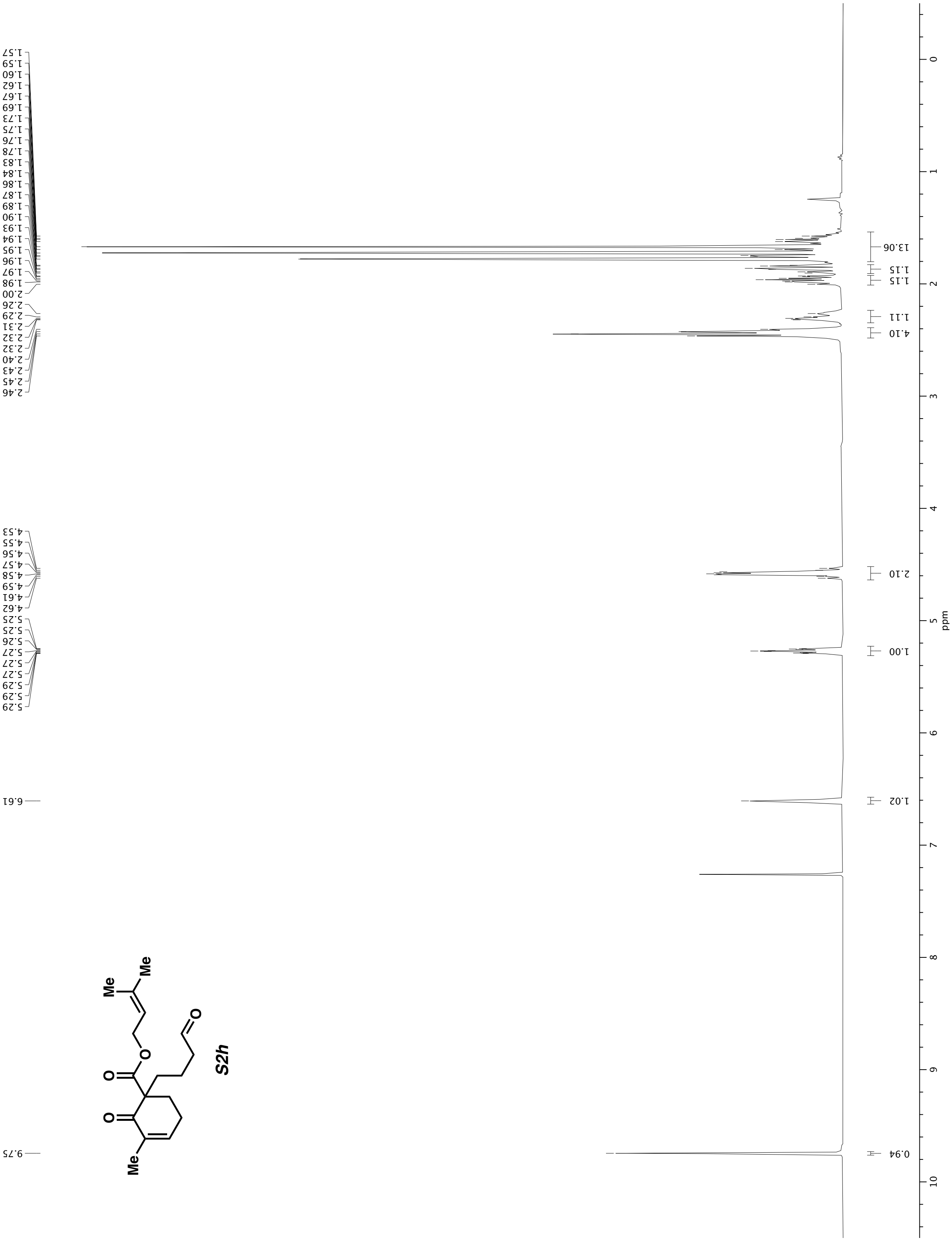

^{1}H NMR (400 MHz, $CDCl_3$) of compound **S2h**.

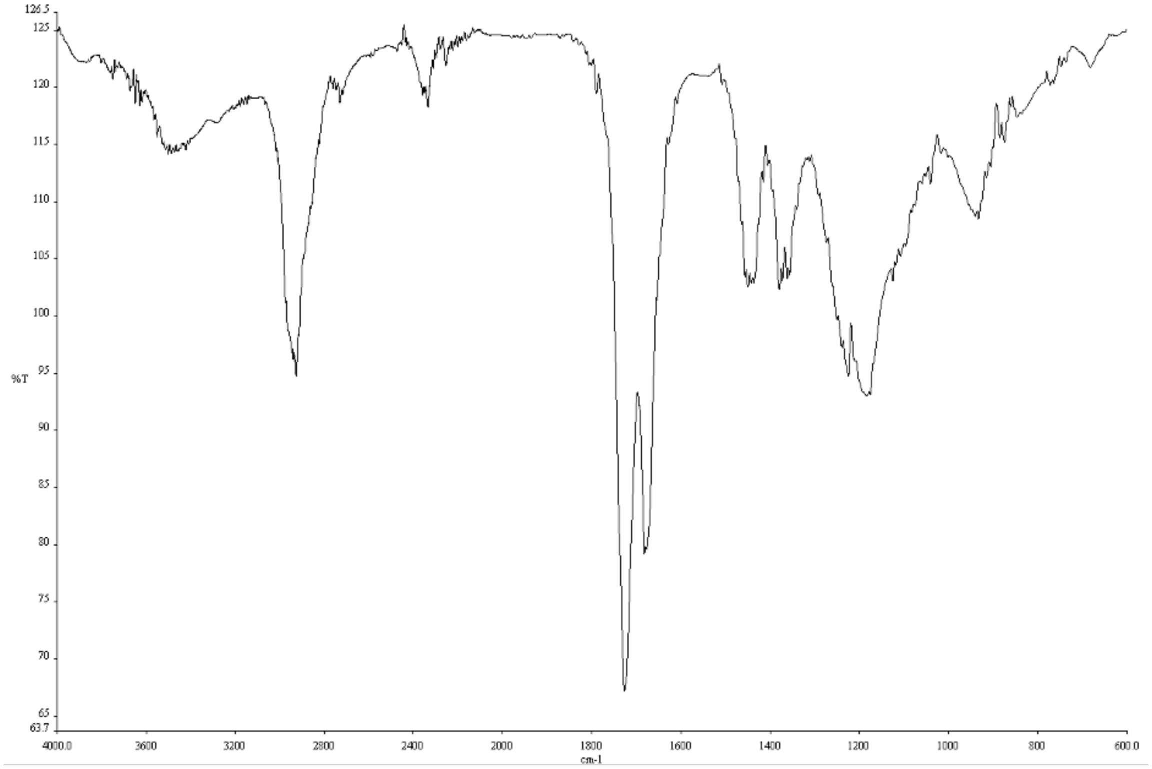

Infrared spectrum (Thin Film, NaCl) of compound **S2h**.

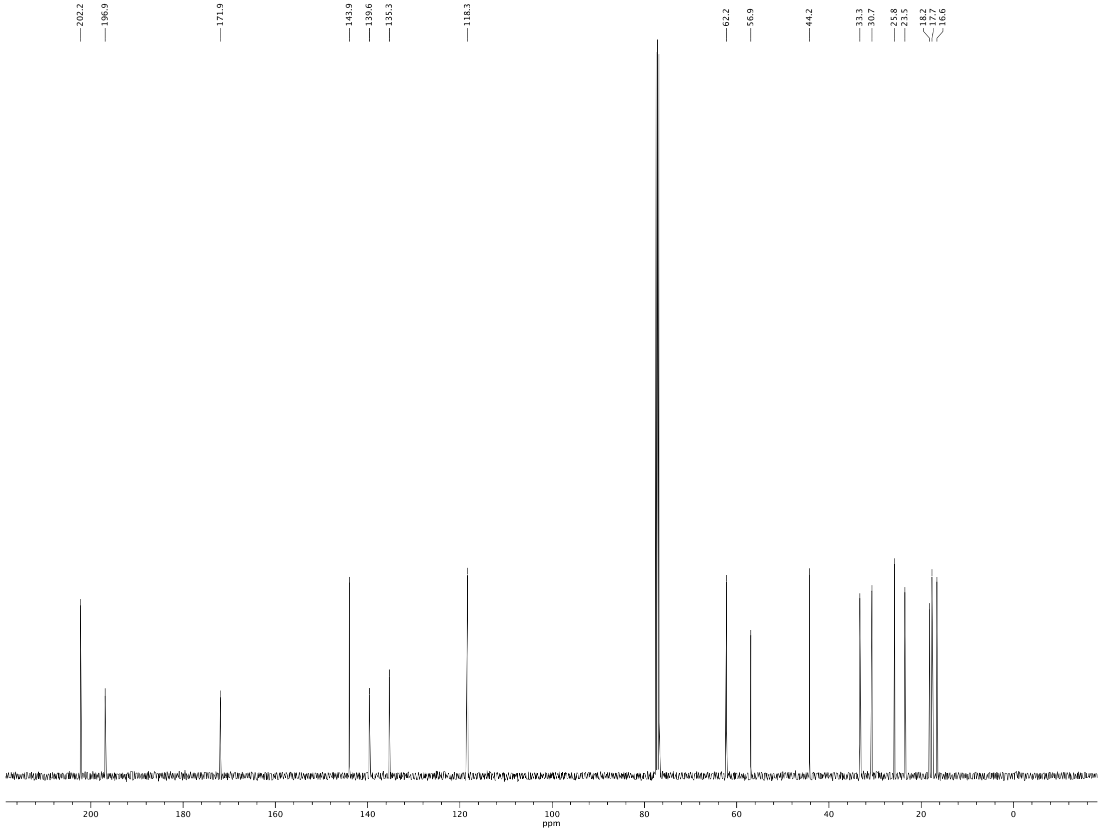

^{13}C NMR (100 MHz, $CDCl_3$) of compound **S2h**.

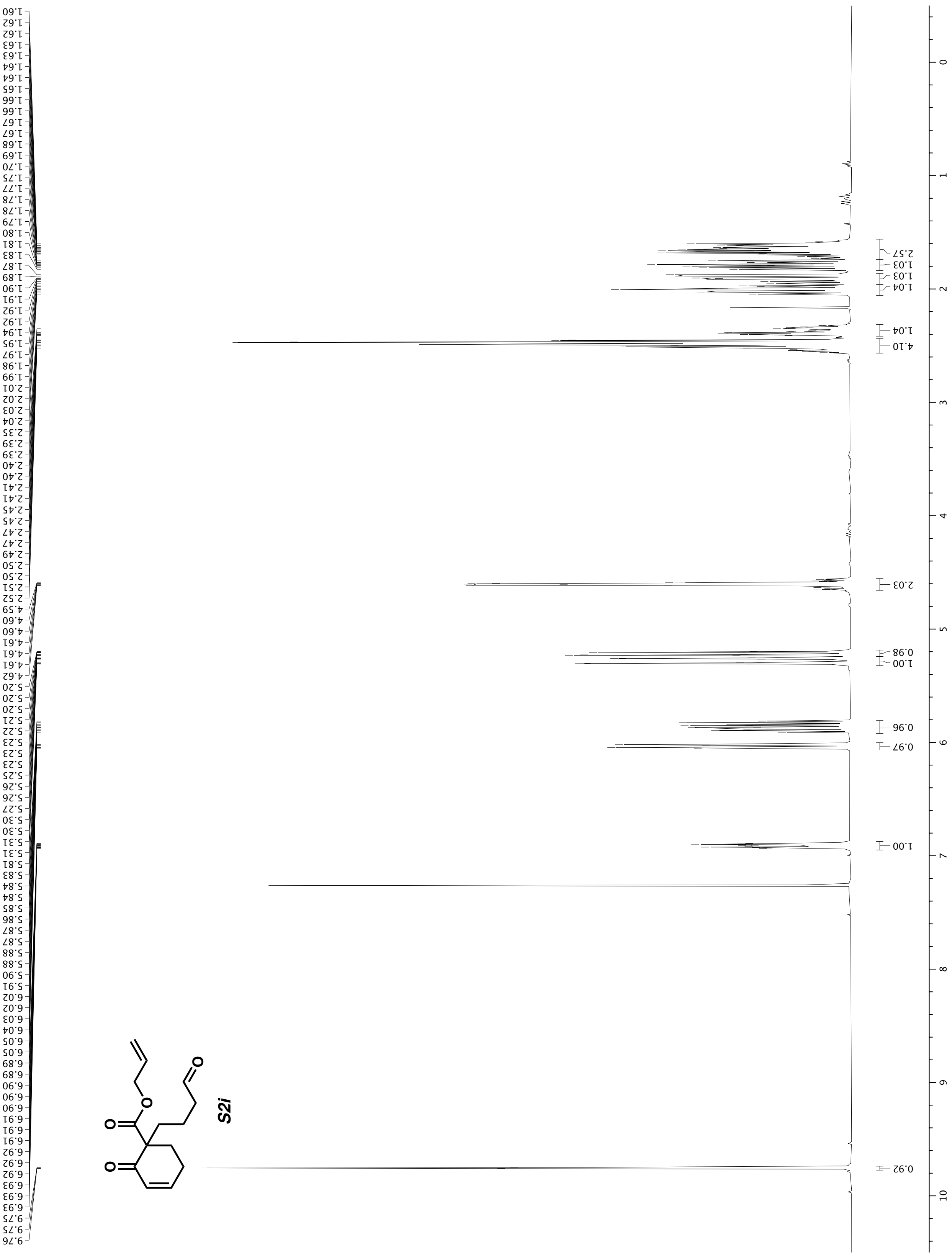

^{1}H NMR (400 MHz, $CDCl_3$) of compound **S2i**.

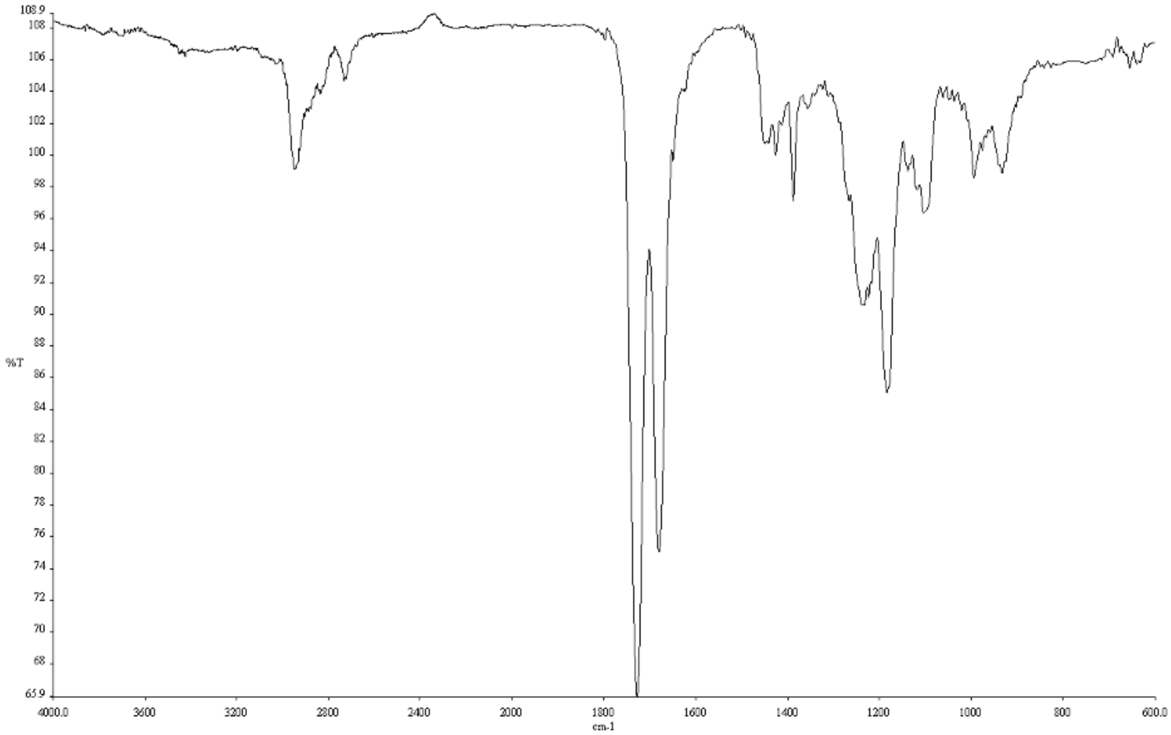

Infrared spectrum (Thin Film, NaCl) of compound **S2i**.

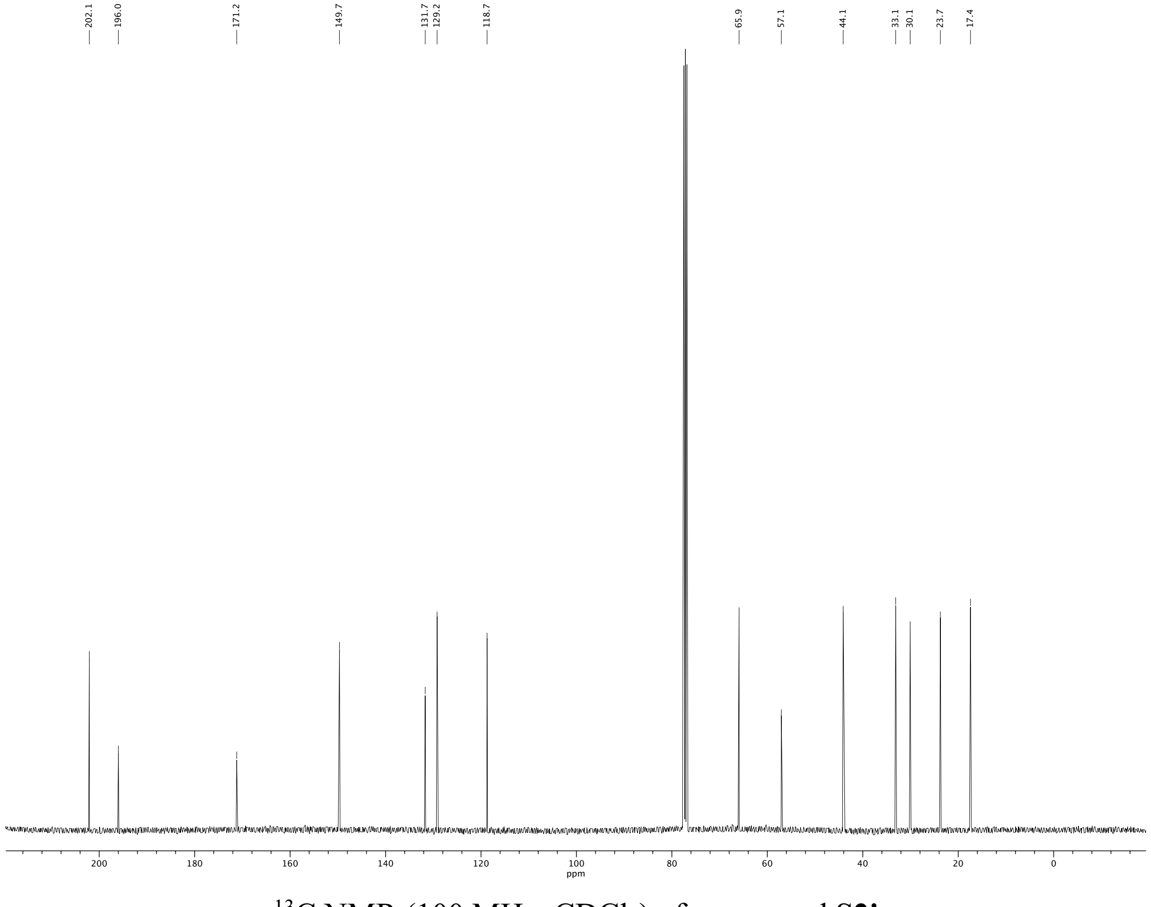

^{13}C NMR (100 MHz, $CDCl_3$) of compound **S2i**.

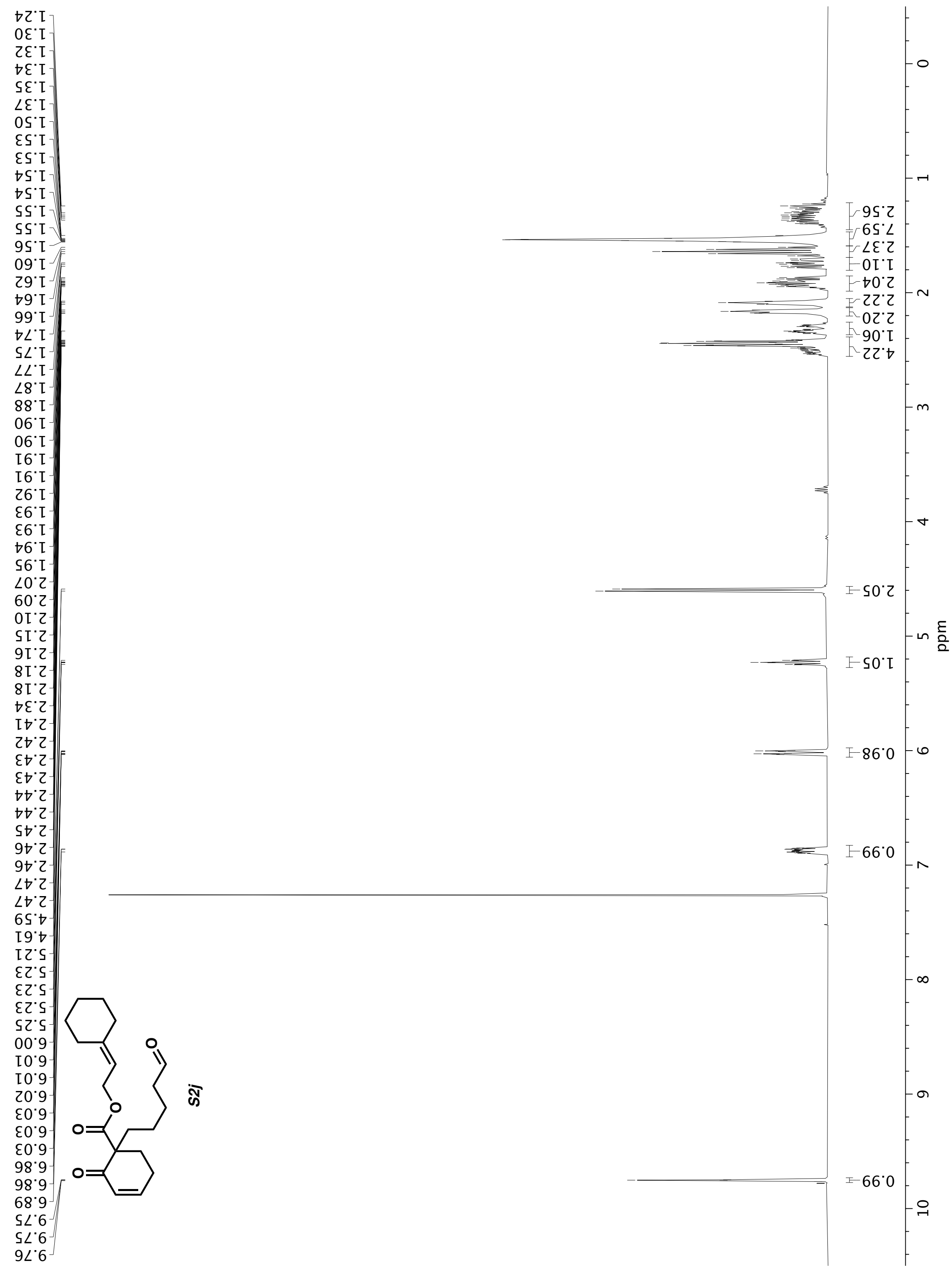

^{1}H NMR (400 MHz, $CDCl_3$) of compound **S2j**.

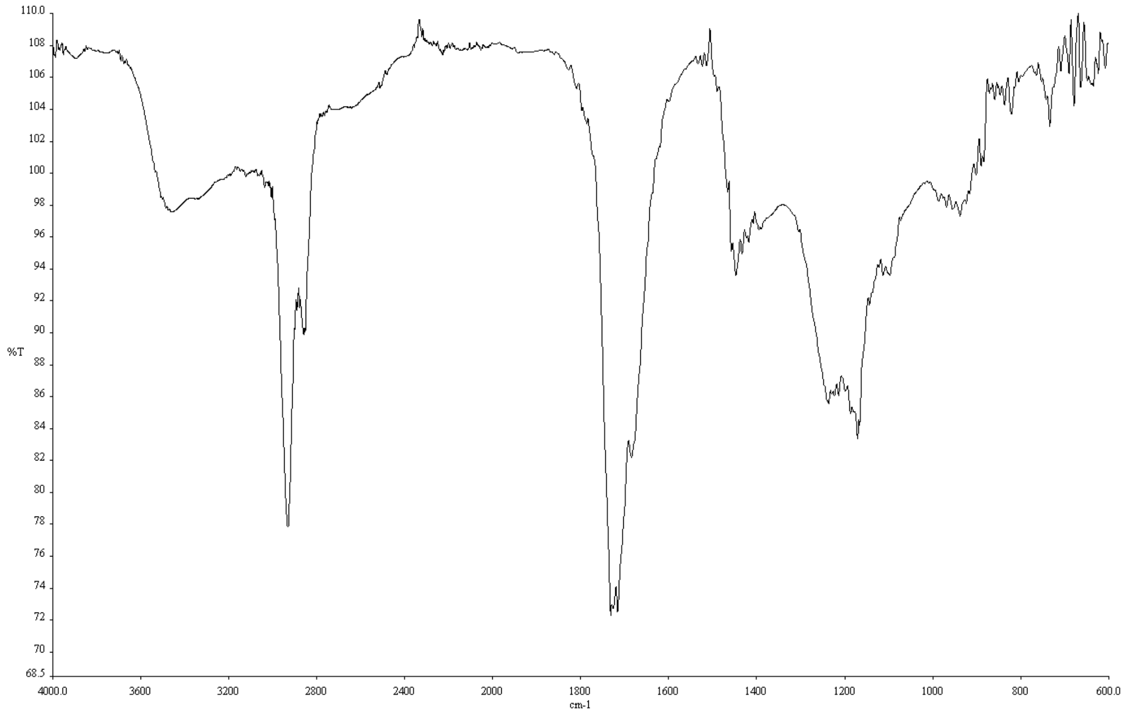

Infrared spectrum (Thin Film, NaCl) of compound **S2j**.

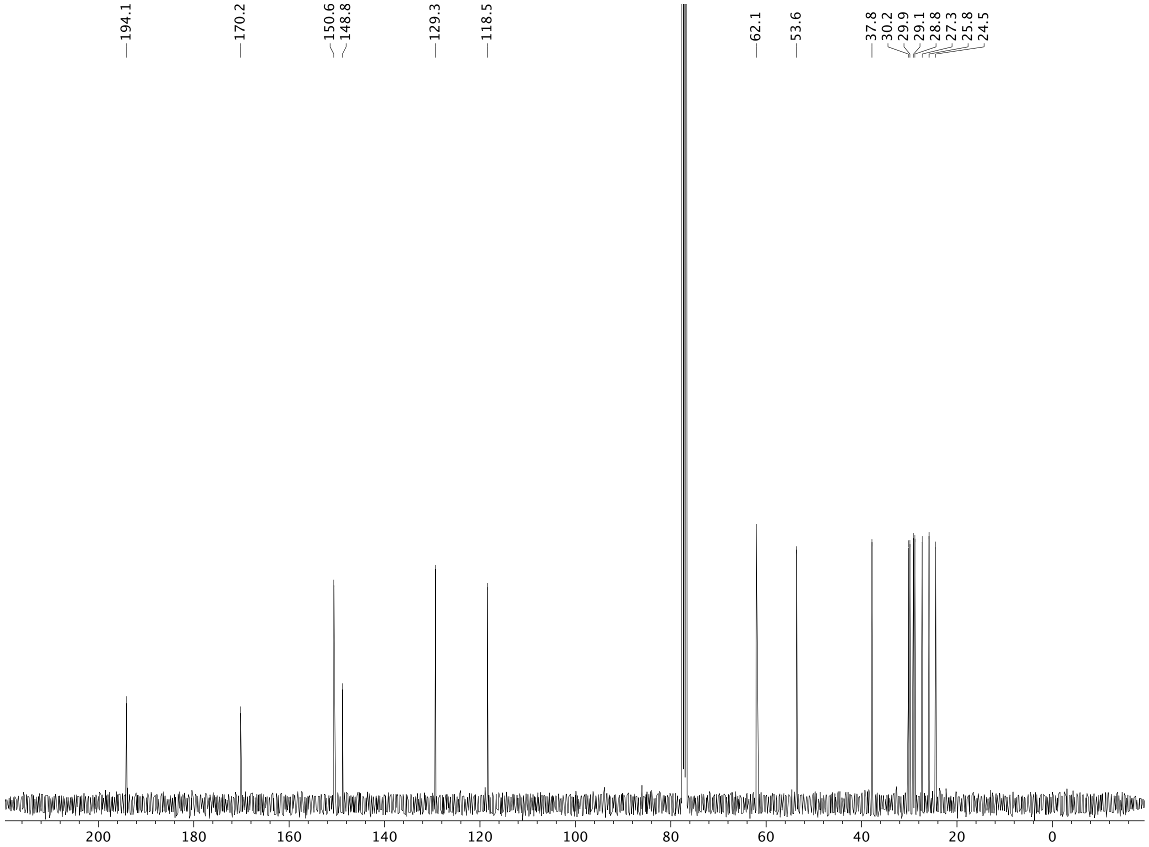

^{13}C NMR (100 MHz, $CDCl_3$) of compound **S2j**.

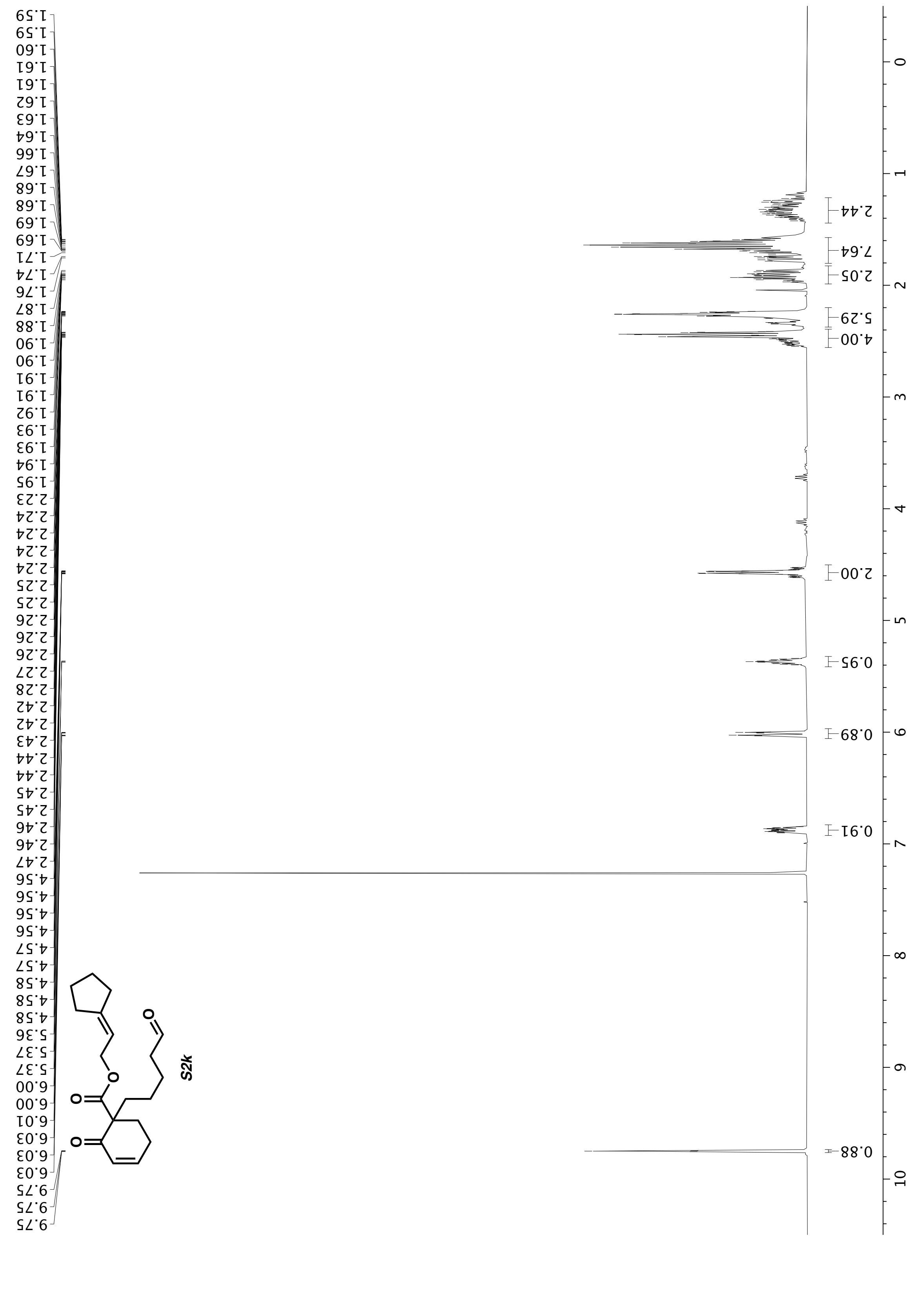

^1H NMR (400 MHz, $CDCl_3$) of compound **S2k**.

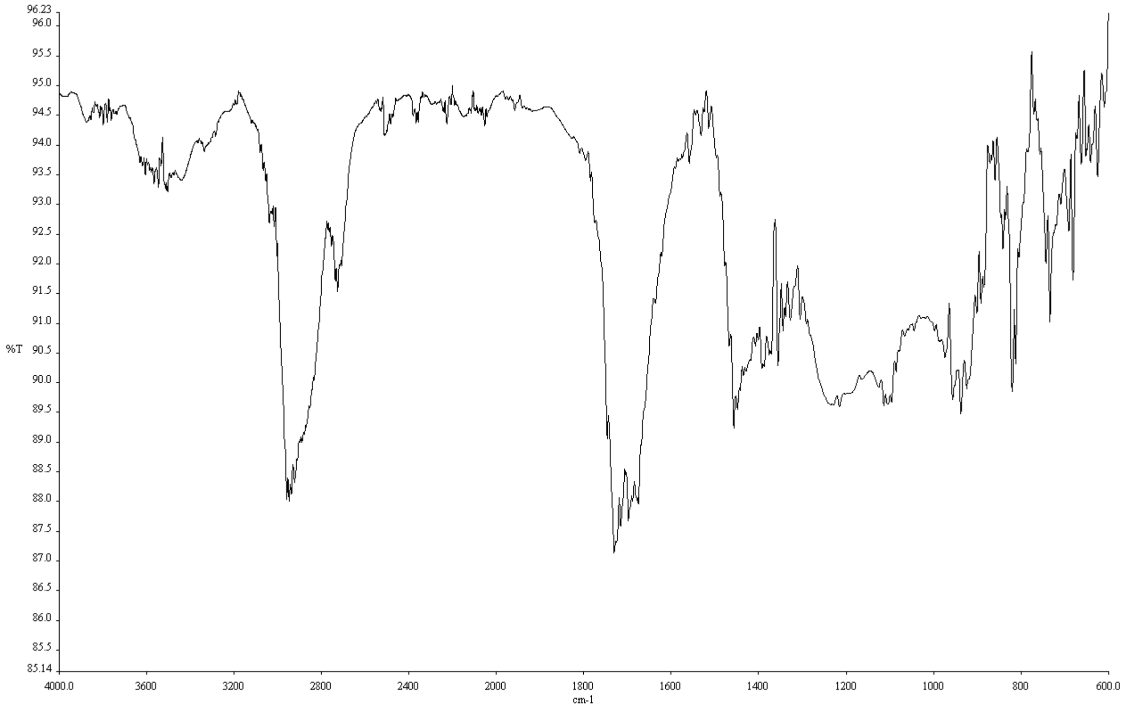

Infrared spectrum (Thin Film, NaCl) of compound **S2k**.

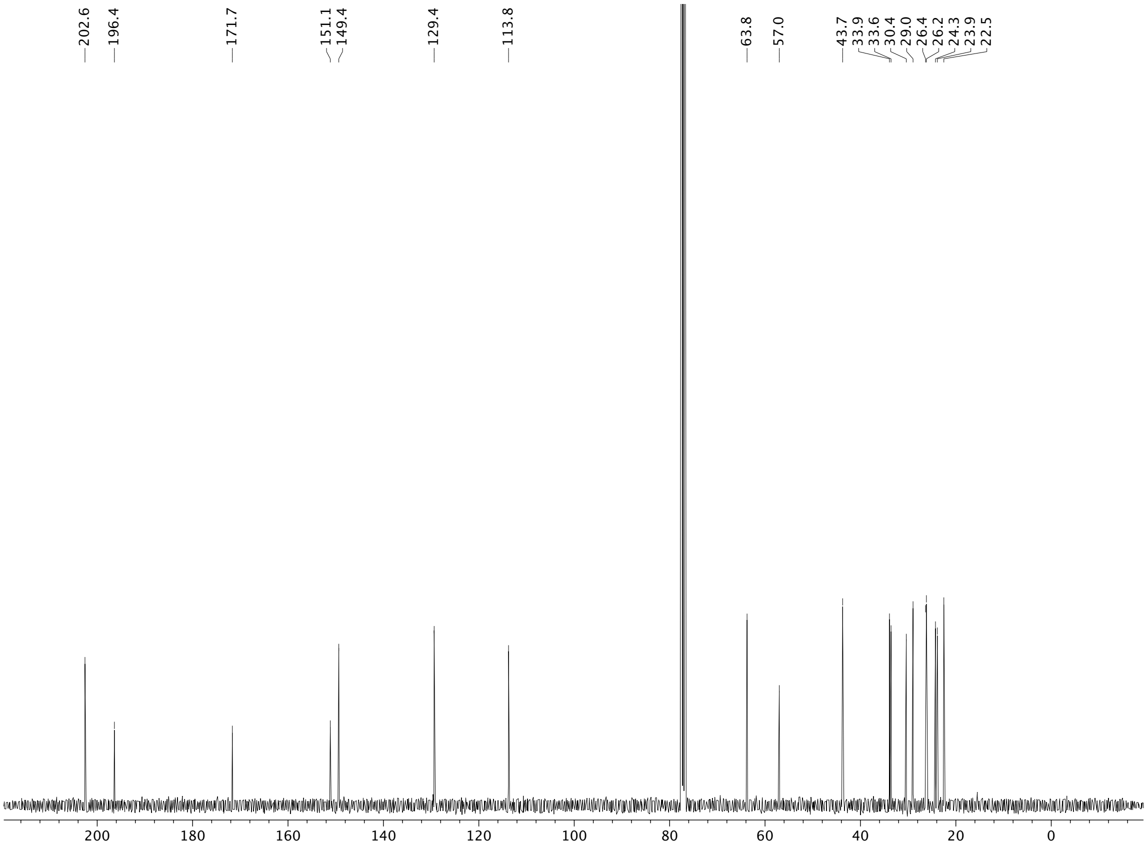

^{13}C NMR (100 MHz, $CDCl_3$) of compound **S2k**.

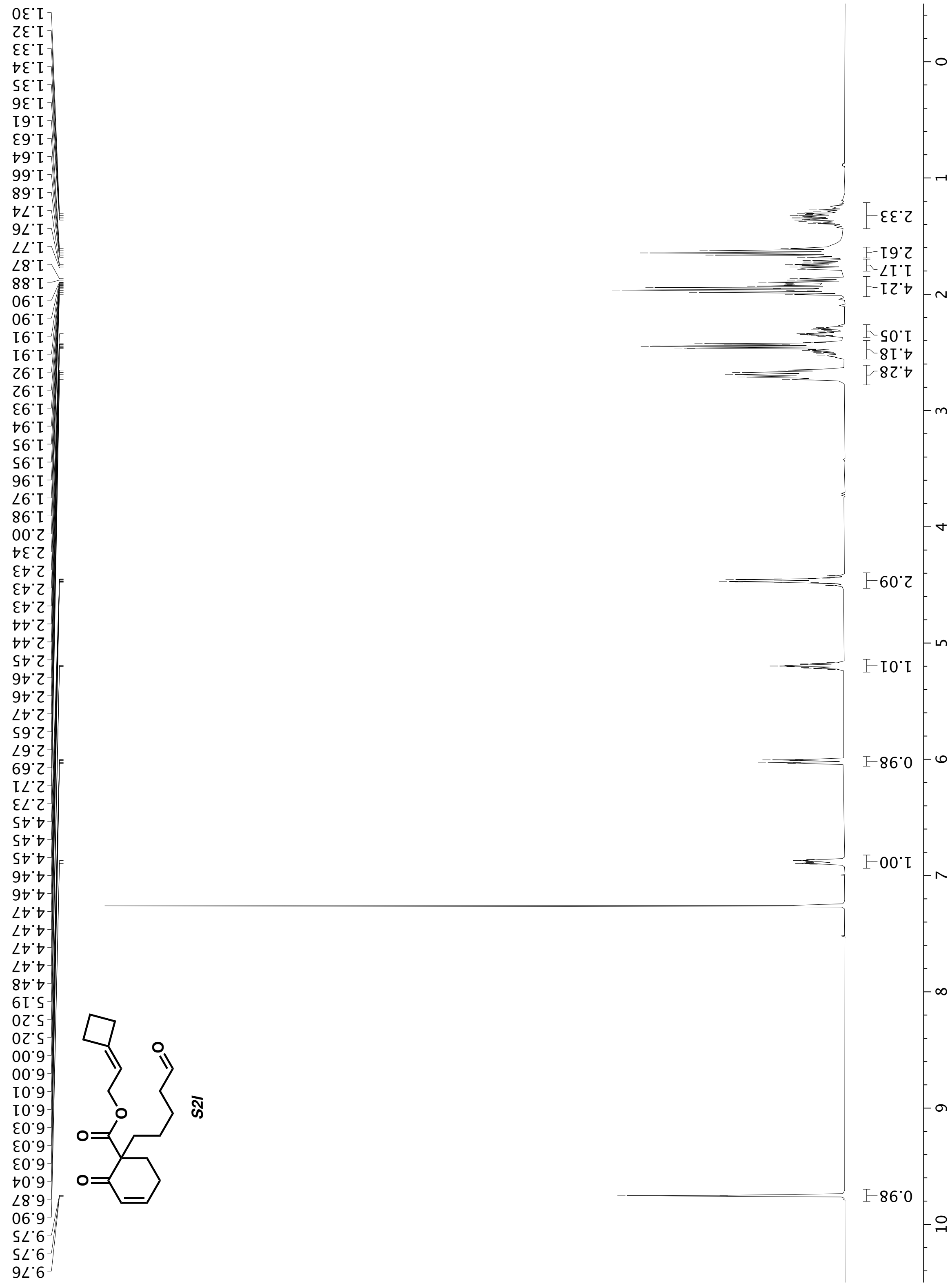

^{1}H NMR (400 MHz, $CDCl_3$) of compound **S21**.

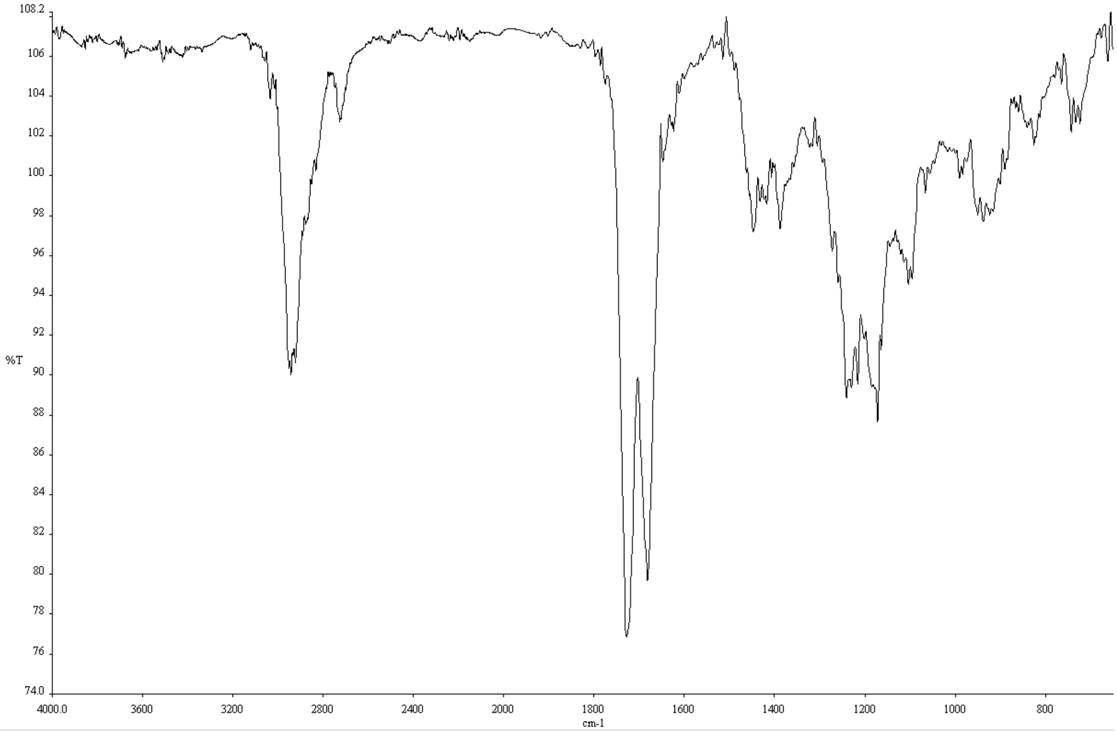

Infrared spectrum (Thin Film, NaCl) of compound **S2l**.

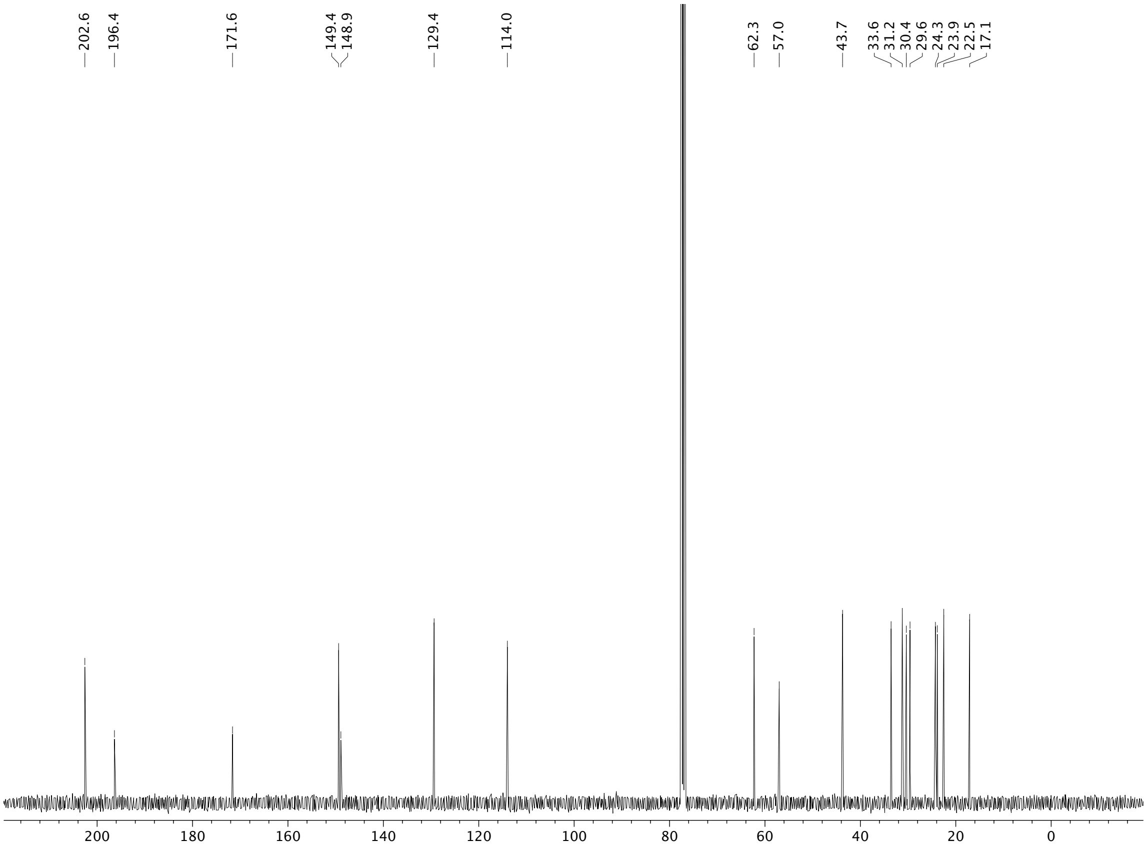

^{13}C NMR (100 MHz, $CDCl_3$) of compound **S2l**.

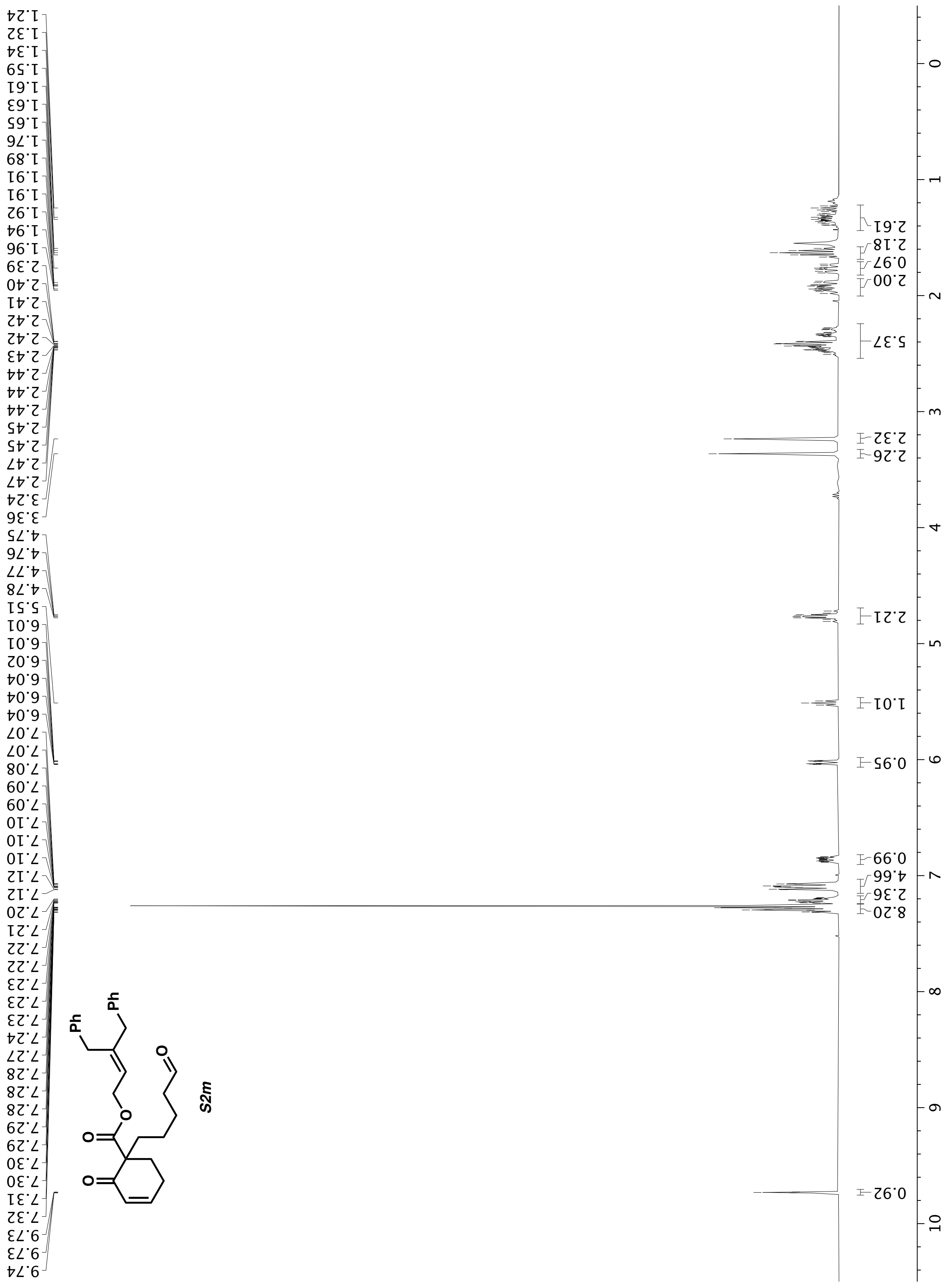

^{1}H NMR (400 MHz, CDCl$_3$) of compound **S2m**.

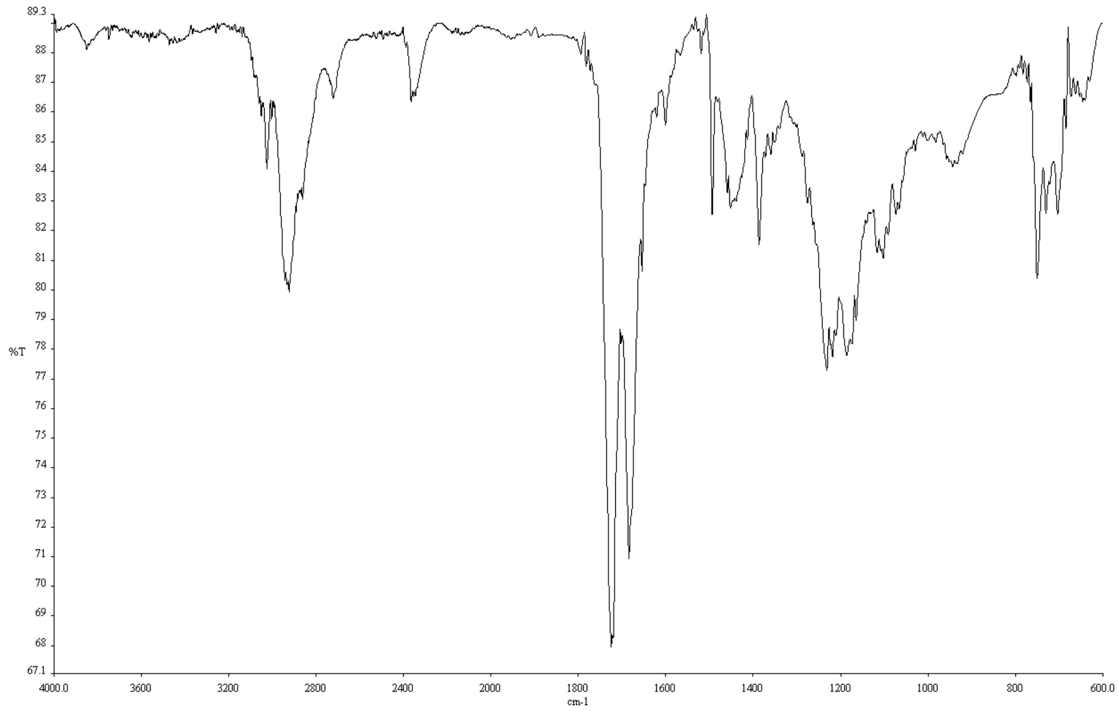

Infrared spectrum (Thin Film, NaCl) of compound **S2m**.

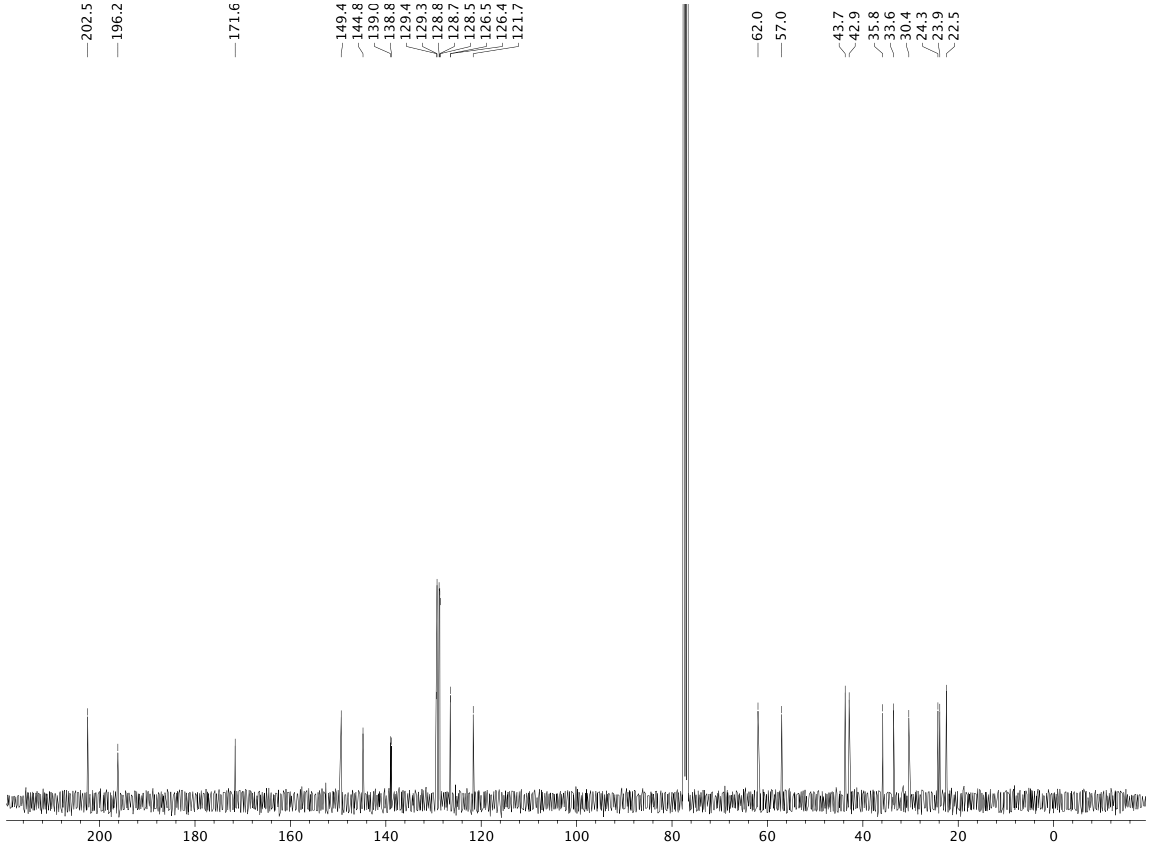

^{13}C NMR (100 MHz, $CDCl_3$) of compound **S2m**.

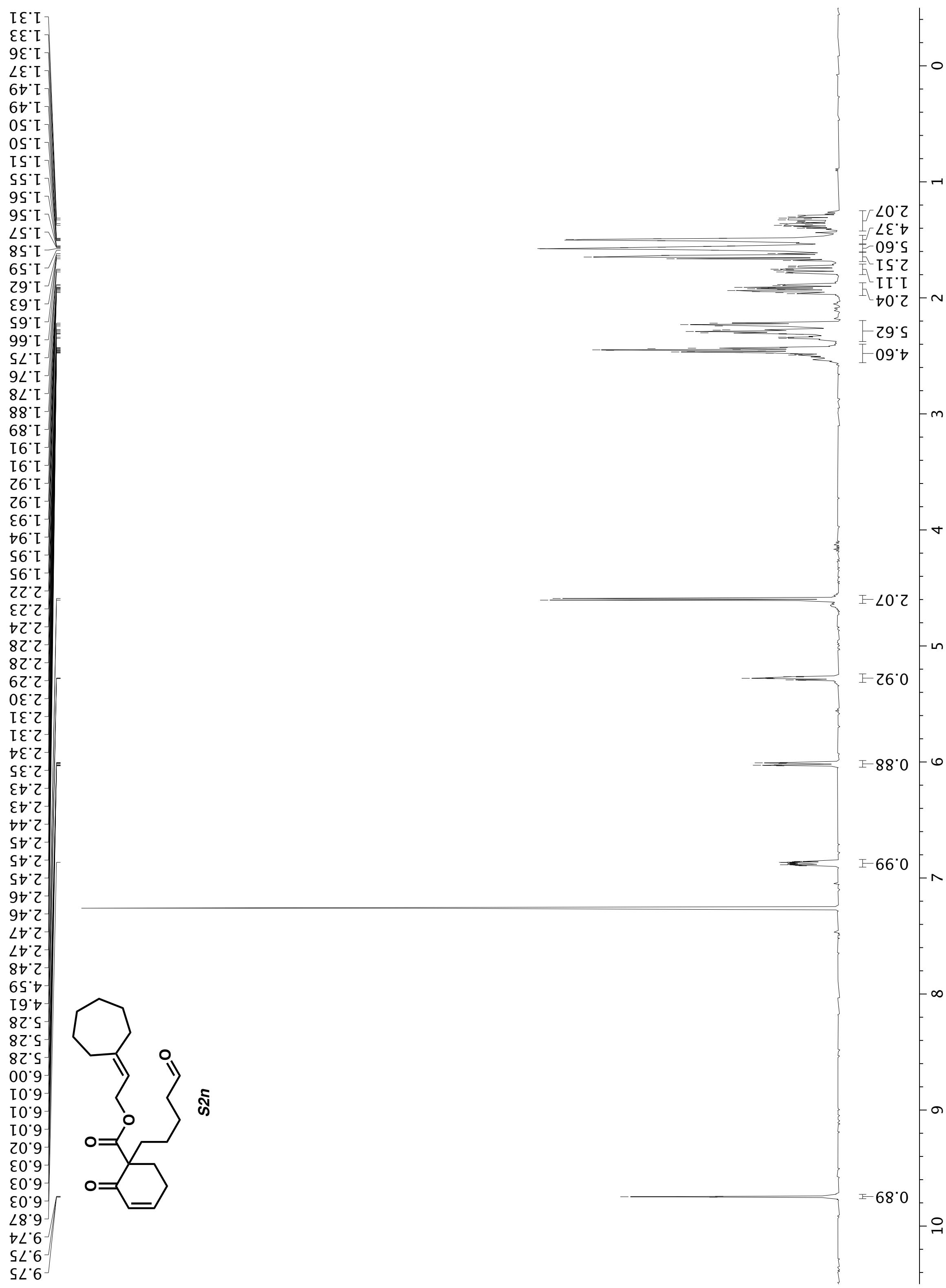

^{1}H NMR (400 MHz, $CDCl_3$) of compound **S2n**.

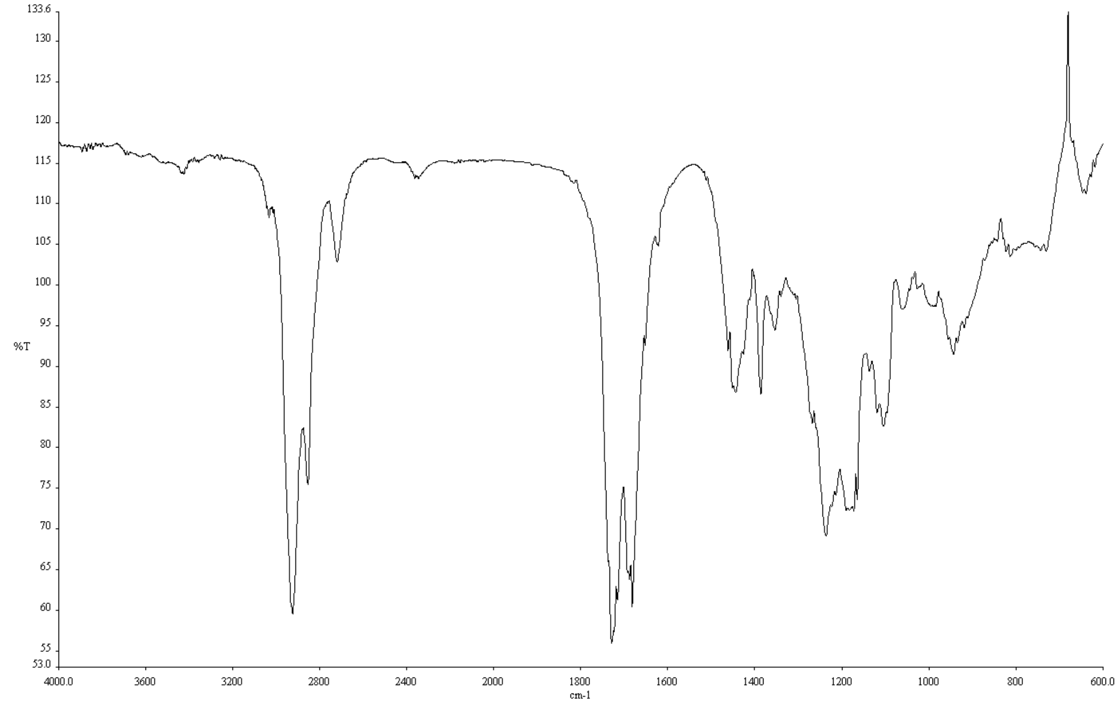

Infrared spectrum (Thin Film, NaCl) of compound **S2n**.

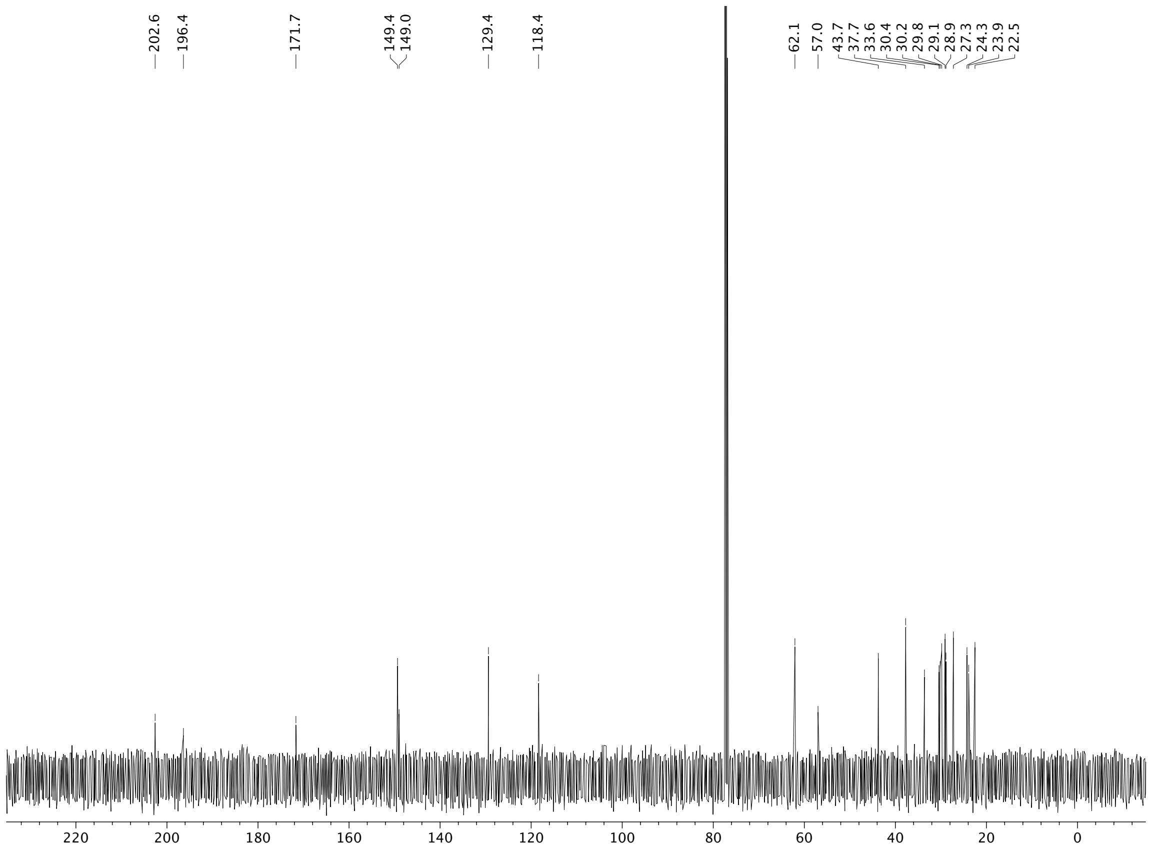

^{13}C NMR (100 MHz, $CDCl_3$) of compound **S2n**.

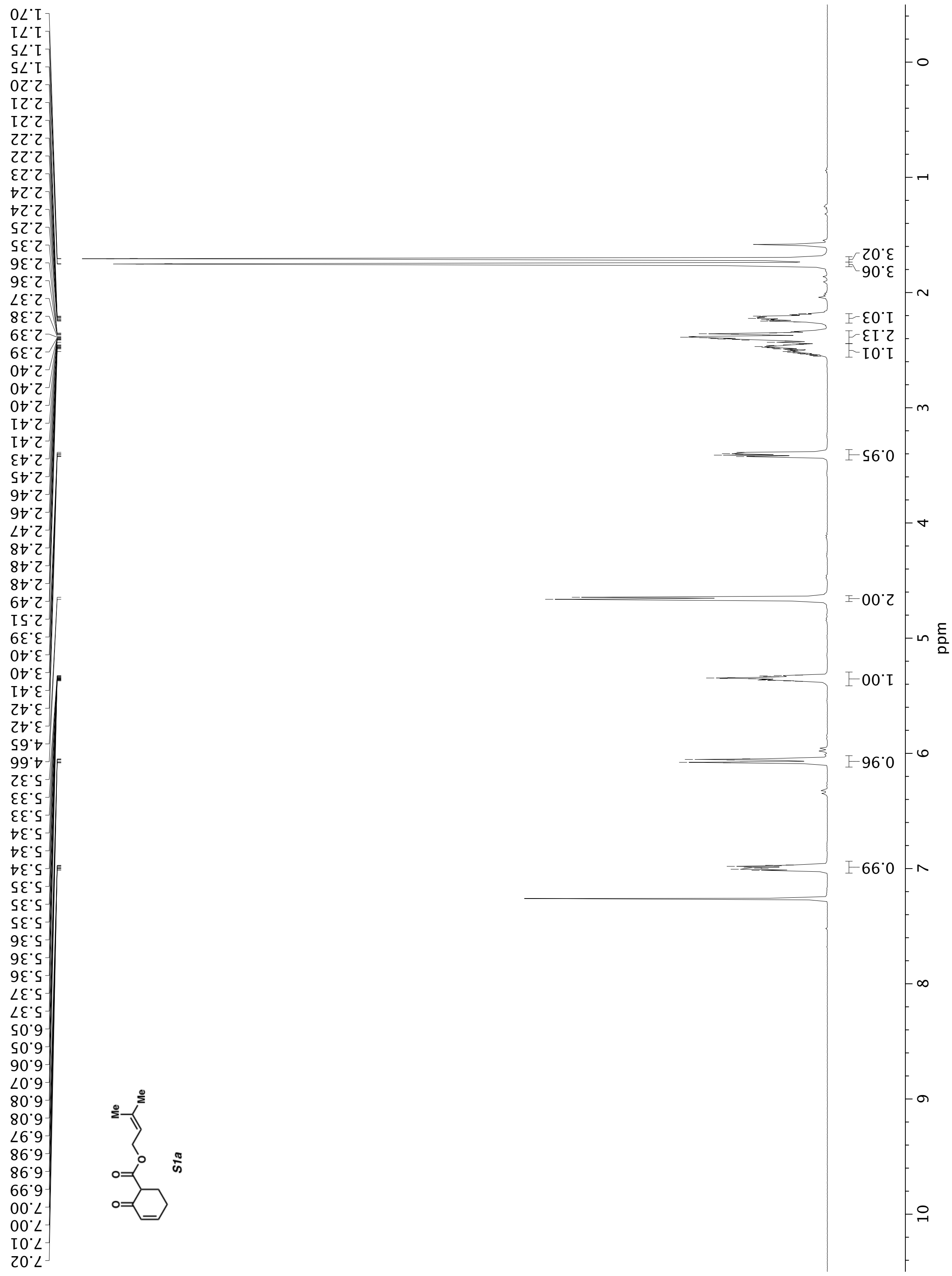

^{1}H NMR (400 MHz, $CDCl_3$) of compound **S1a.**

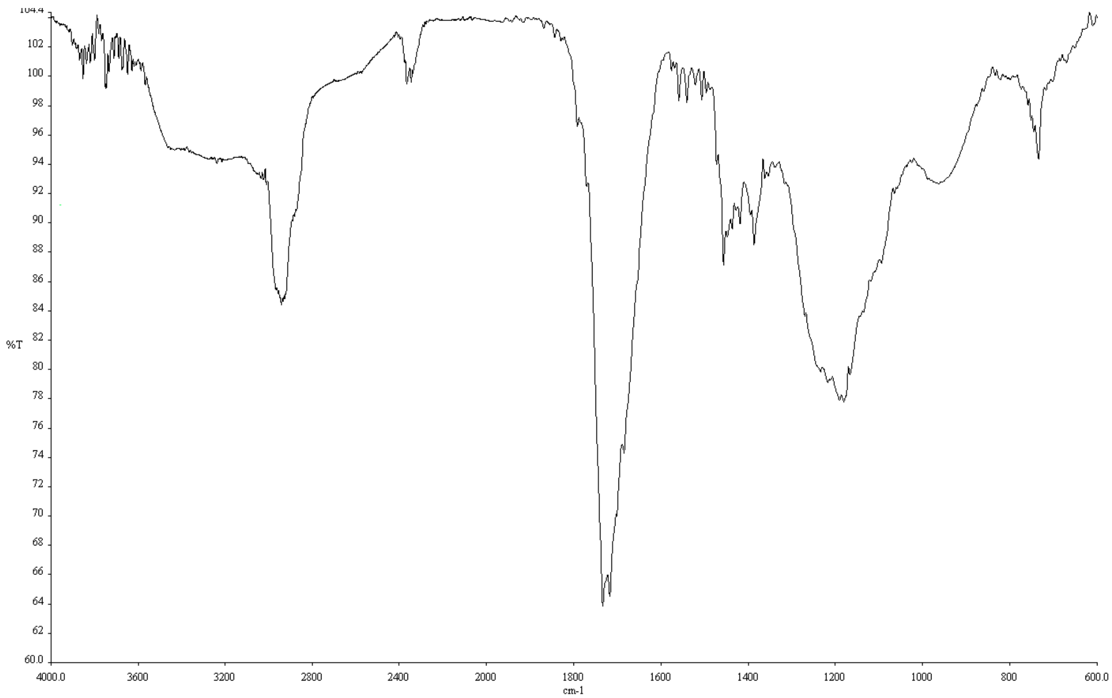

Infrared spectrum (Thin Film, NaCl) of compound **S1a**.

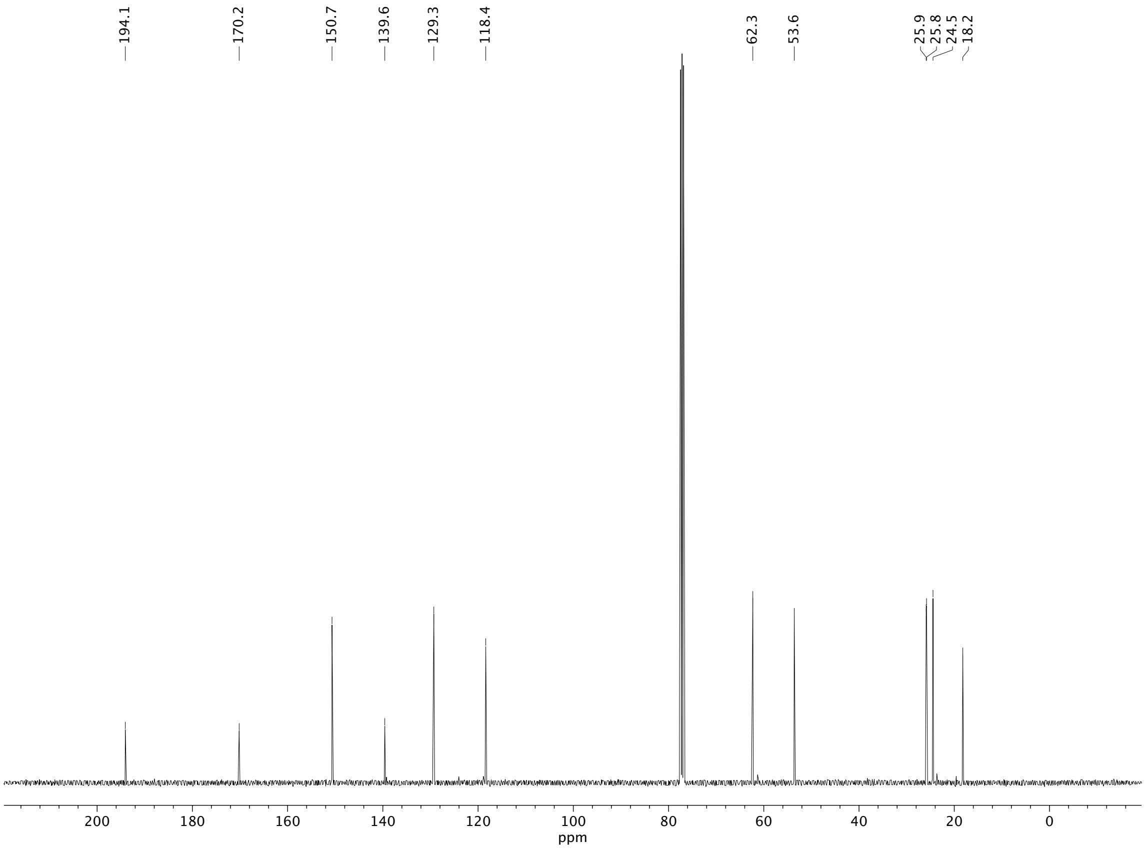

^{13}C NMR (100 MHz, $CDCl_3$) of compound **S1a**.

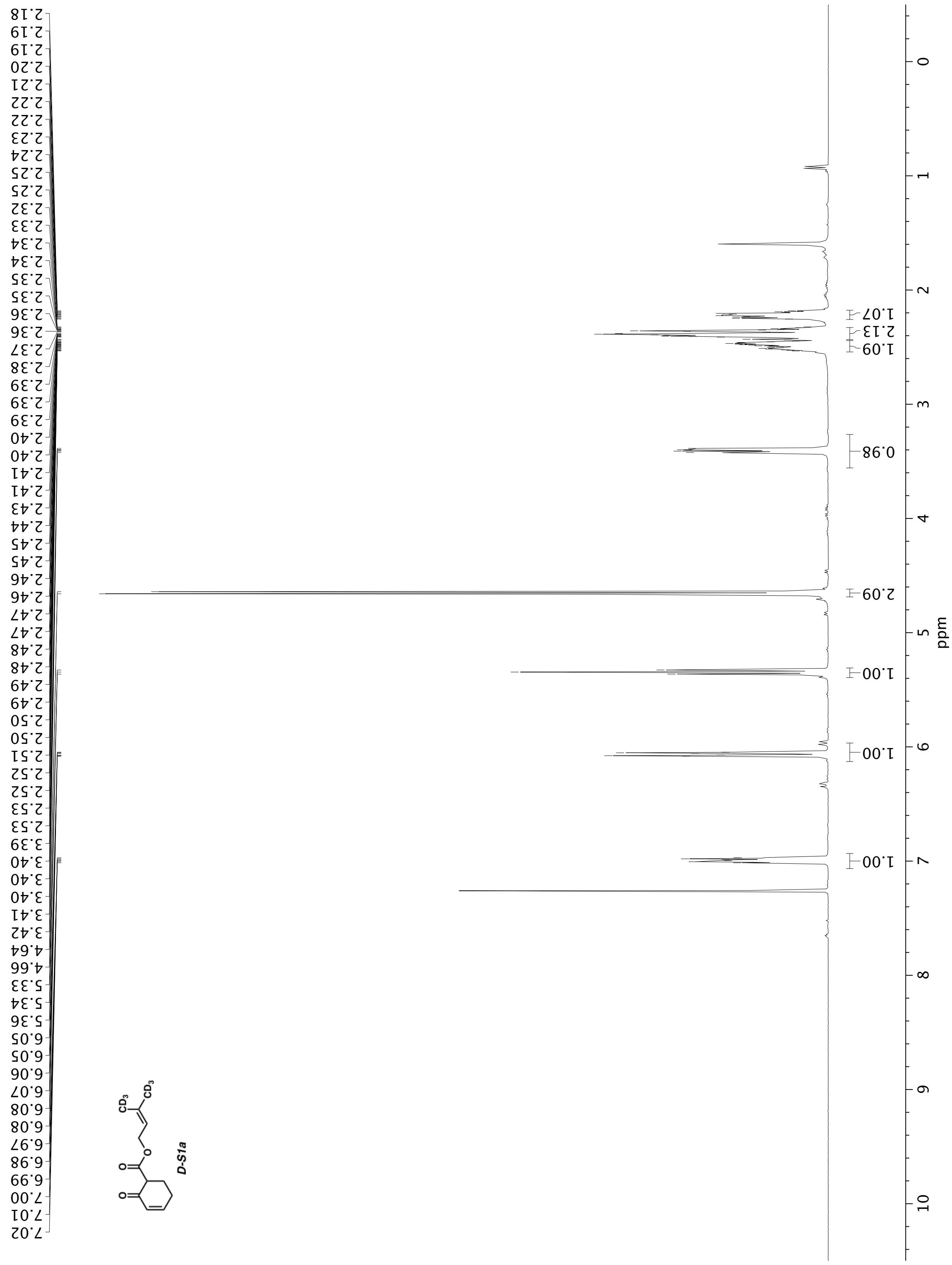

^{1}H NMR (400 MHz, $CDCl_3$) of compound ***D*-S1a.**

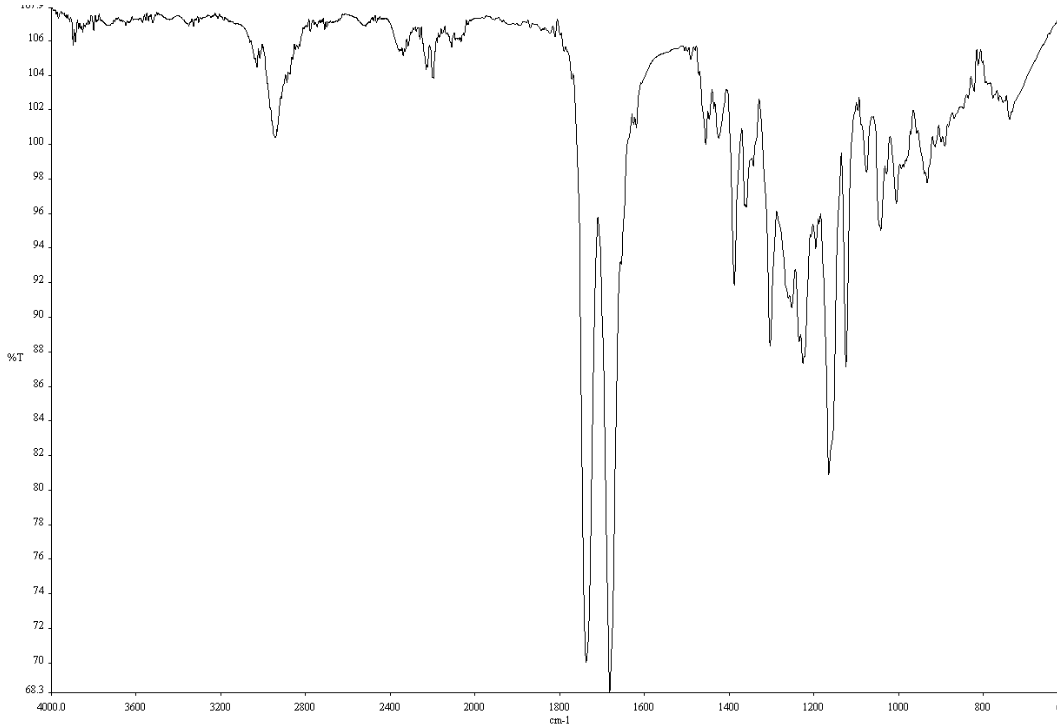

Infrared spectrum (Thin Film, NaCl) of compound ***D*-S1a**.

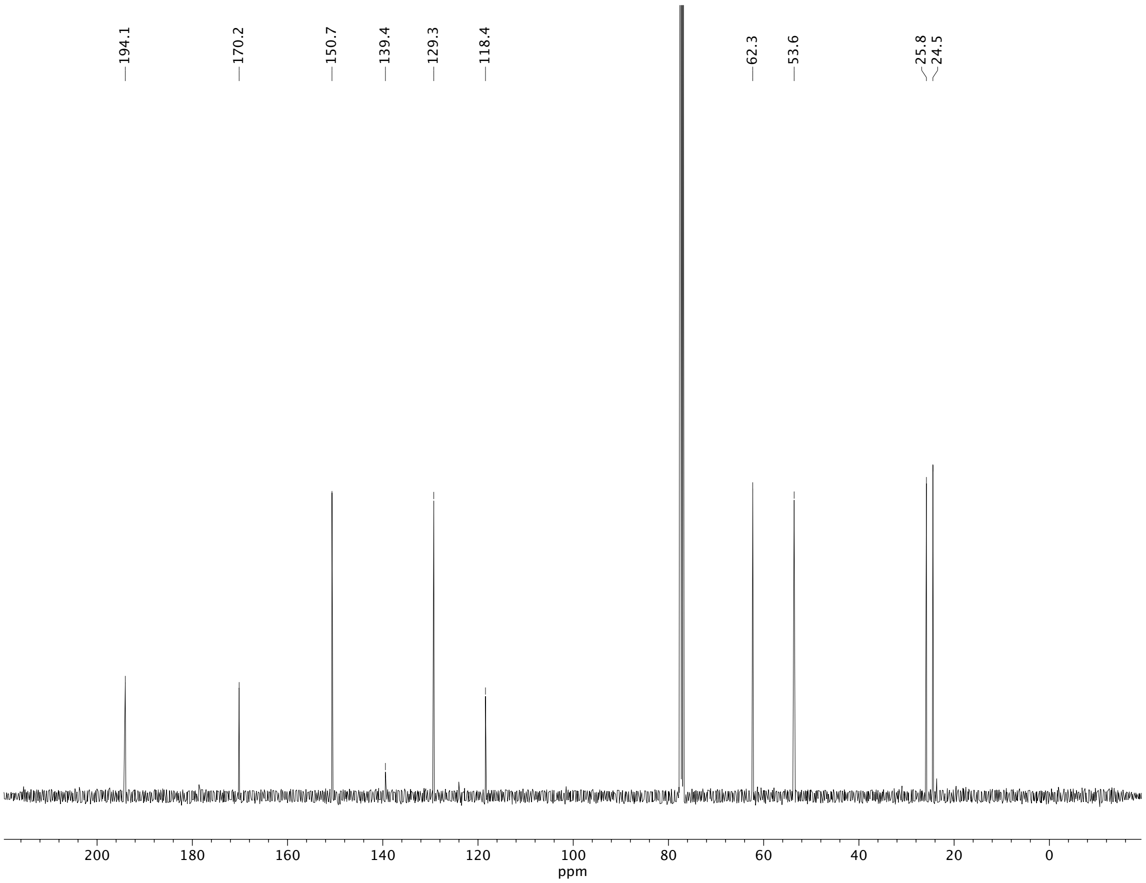

^{13}C NMR (100 MHz, $CDCl_3$) of compound ***D*-S1a**.

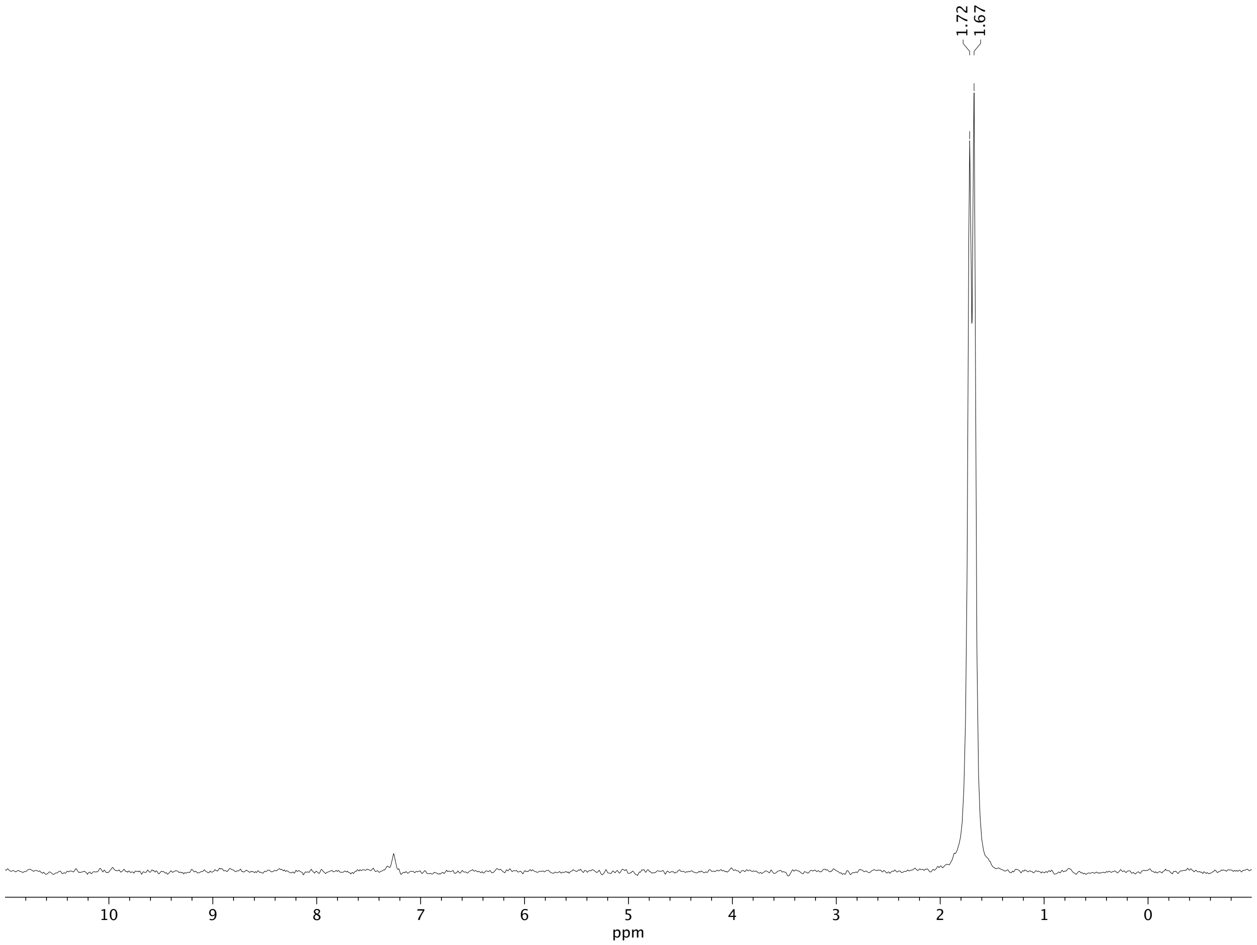

^{2}H NMR (61 MHz, $CHCl_3$) of compound ***D*-S1a**.

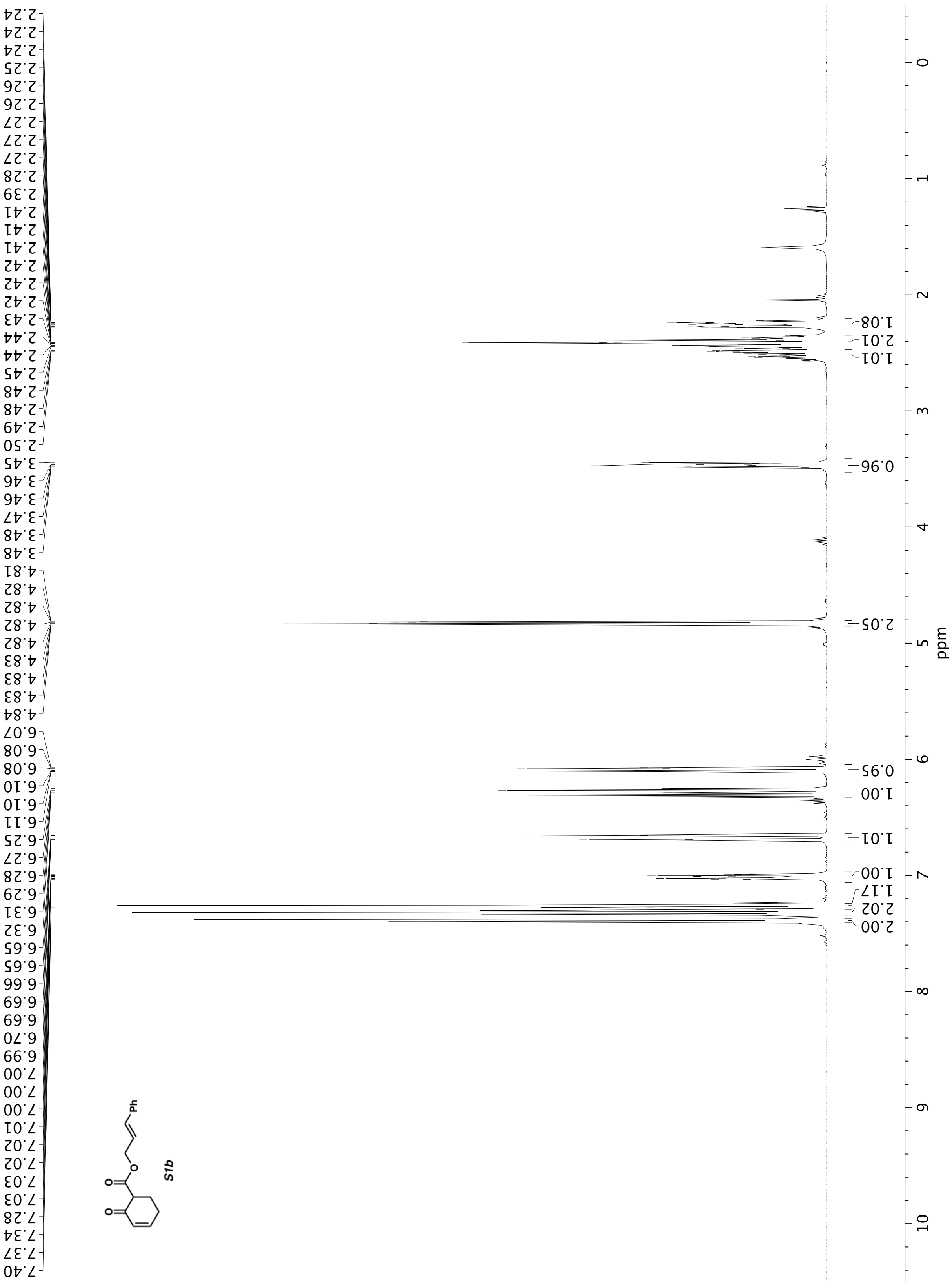

^{1}H NMR (400 MHz, $CDCl_3$) of compound **S1b.**

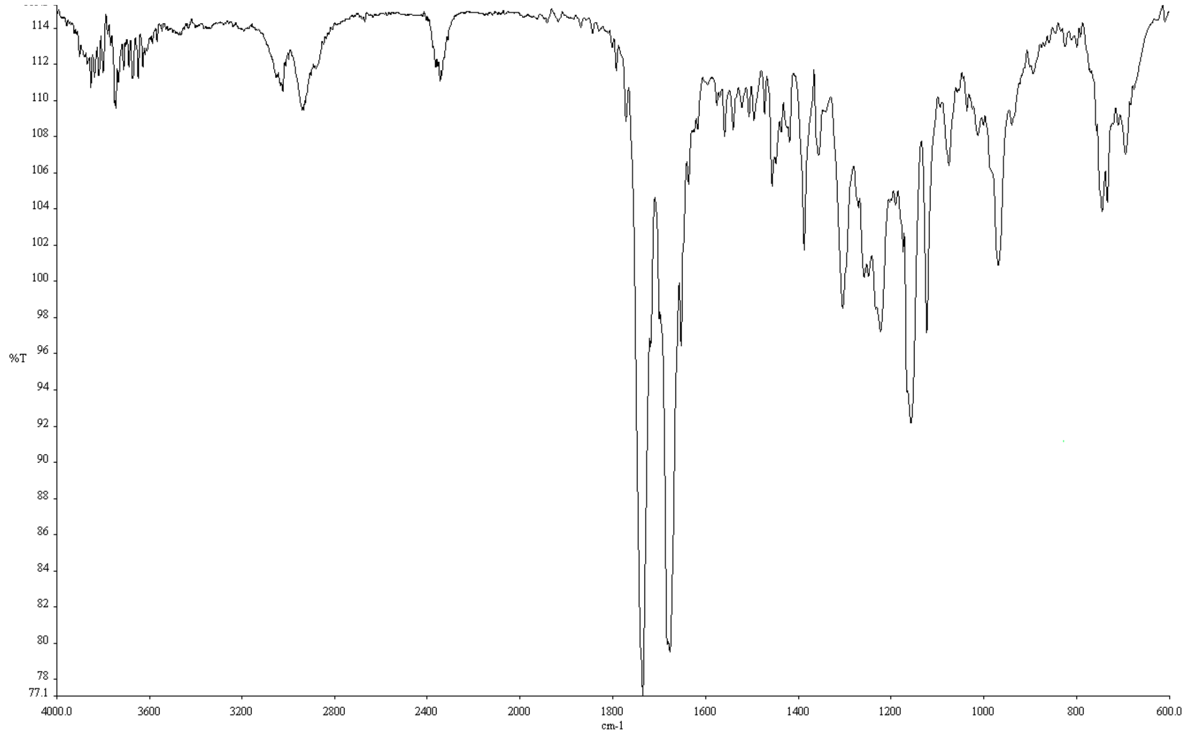

Infrared spectrum (Thin Film, NaCl) of compound **S1b**.

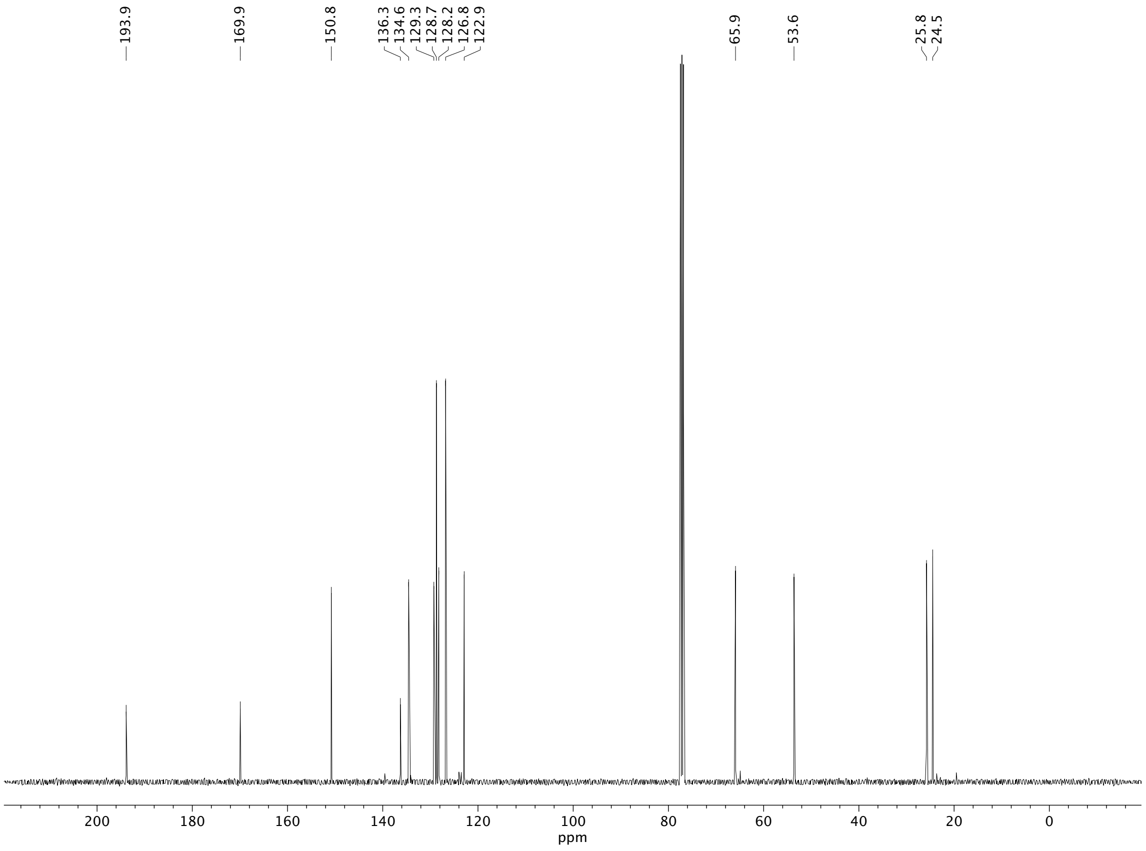

^{13}C NMR (100 MHz, $CDCl_3$) of compound **S1b**.

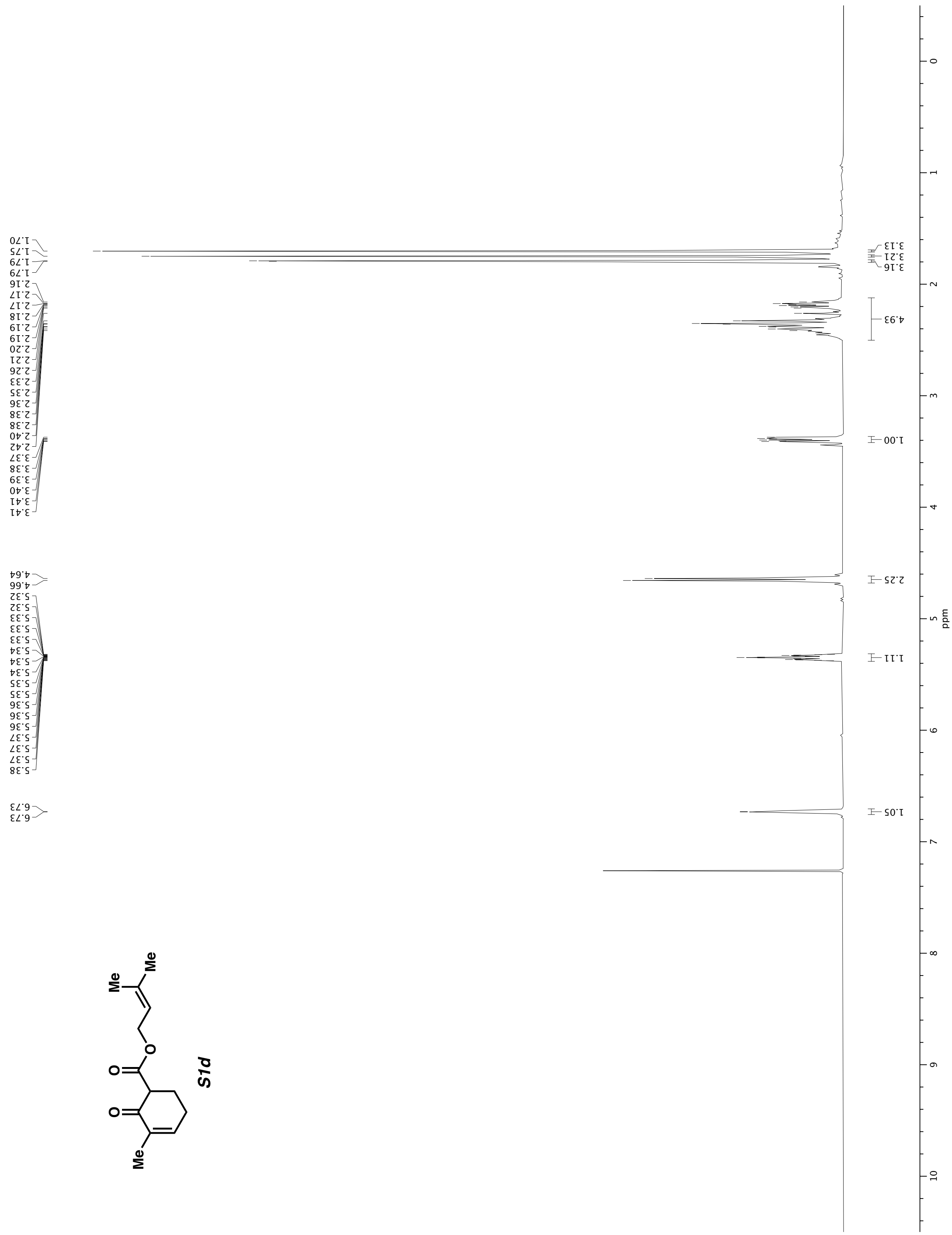

^{1}H NMR (400 MHz, $CDCl_3$) of compound **S1d**.

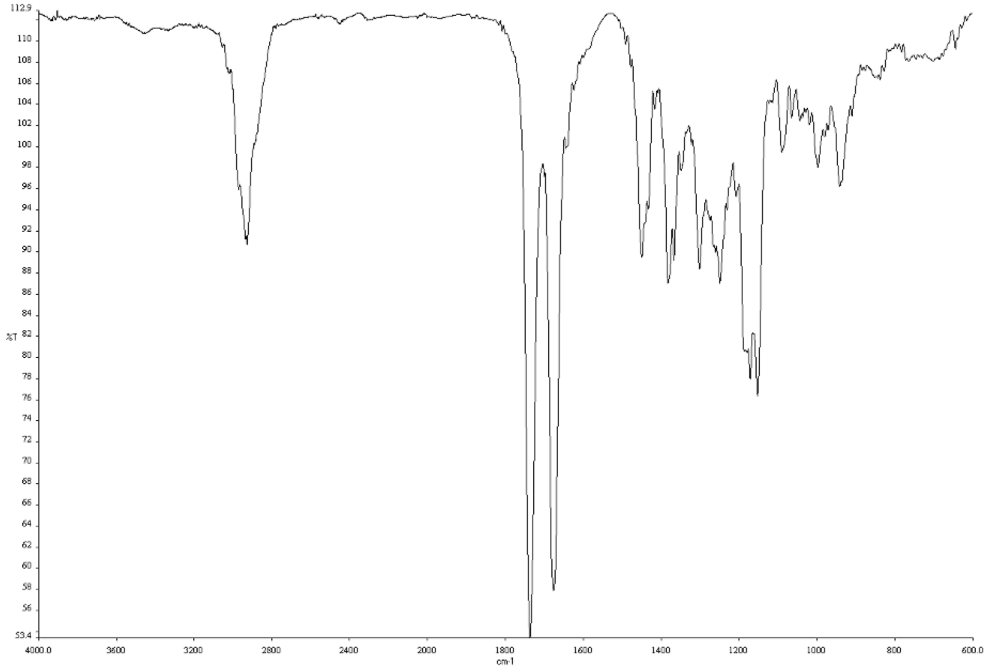

Infrared spectrum (Thin Film, NaCl) of compound **S1d**.

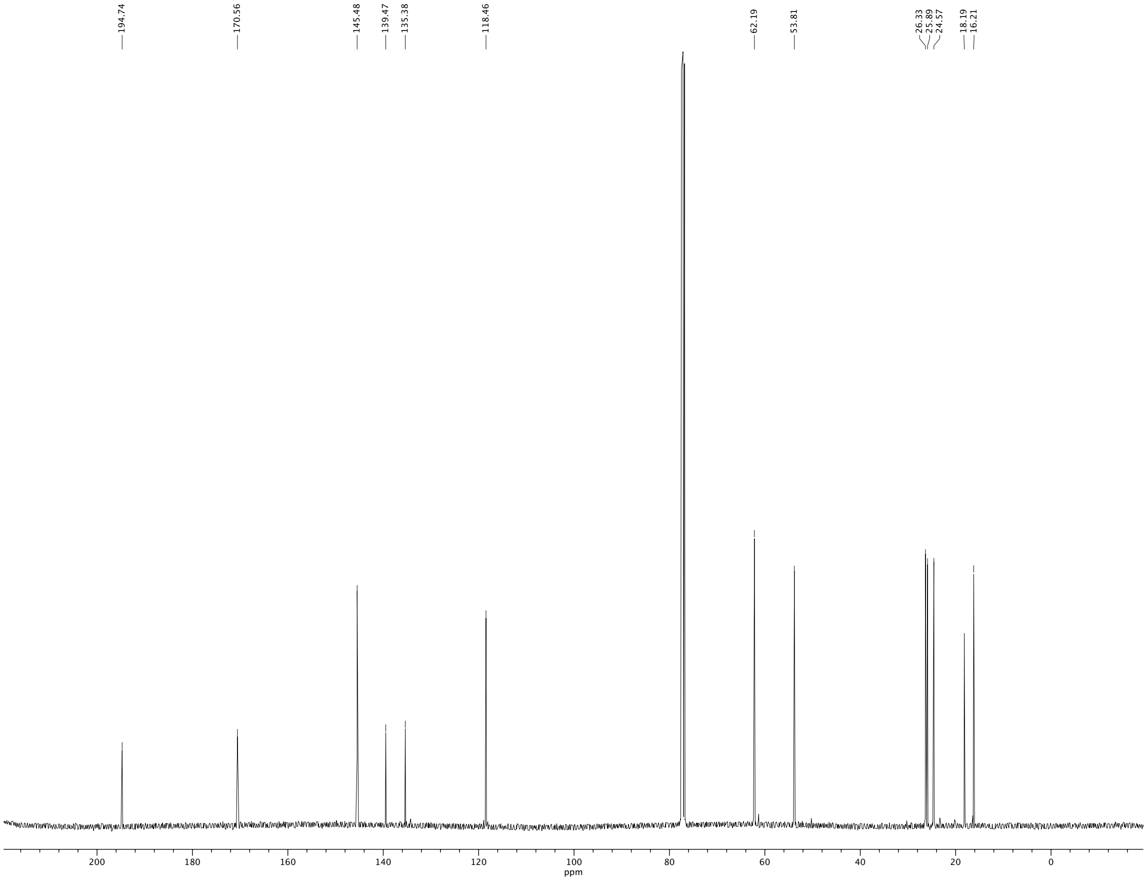

^{13}C NMR (100 MHz, $CDCl_3$) of compound **S1d**.

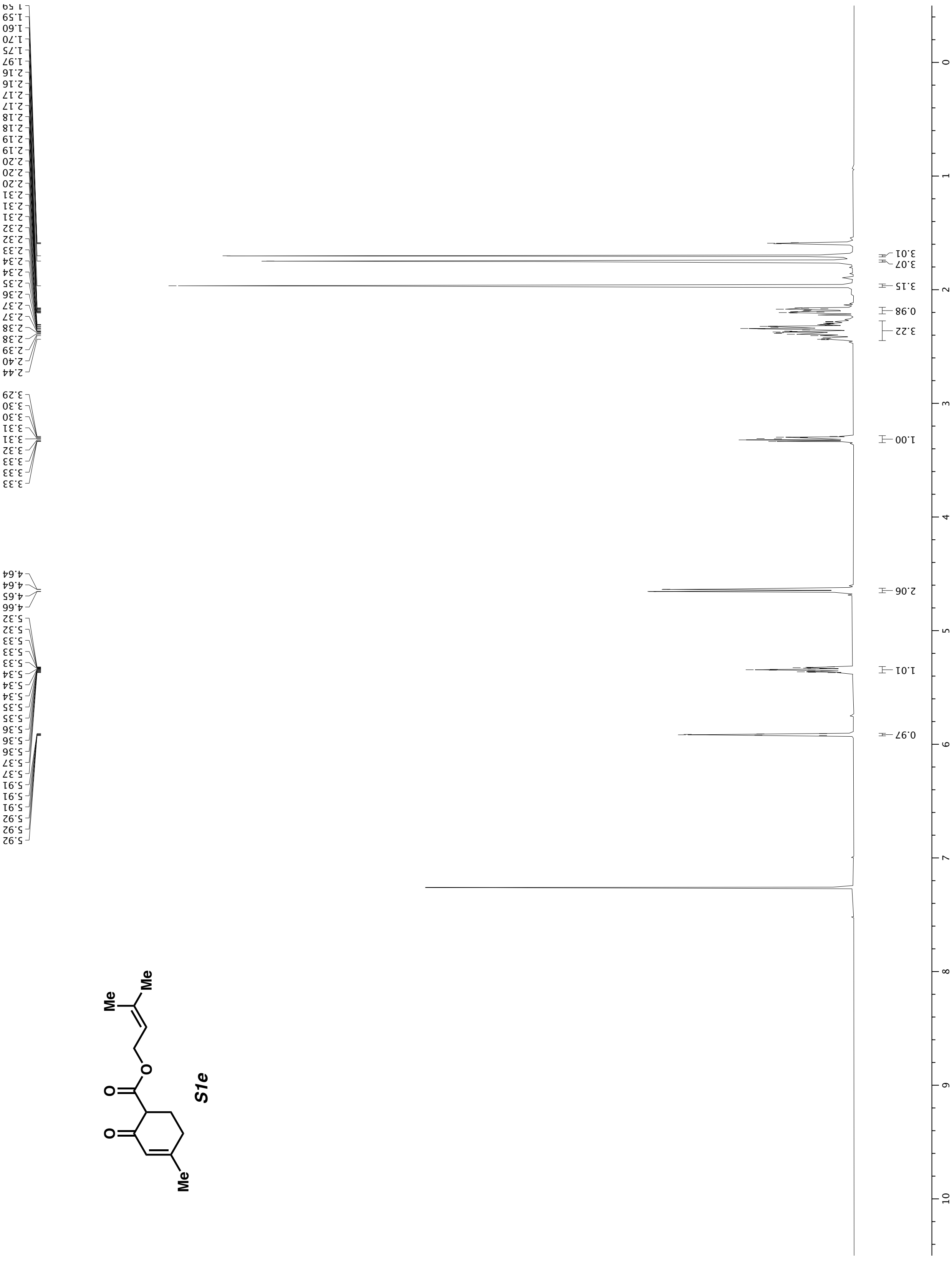

^{1}H NMR (400 MHz, $CDCl_3$) of compound **S1e**.

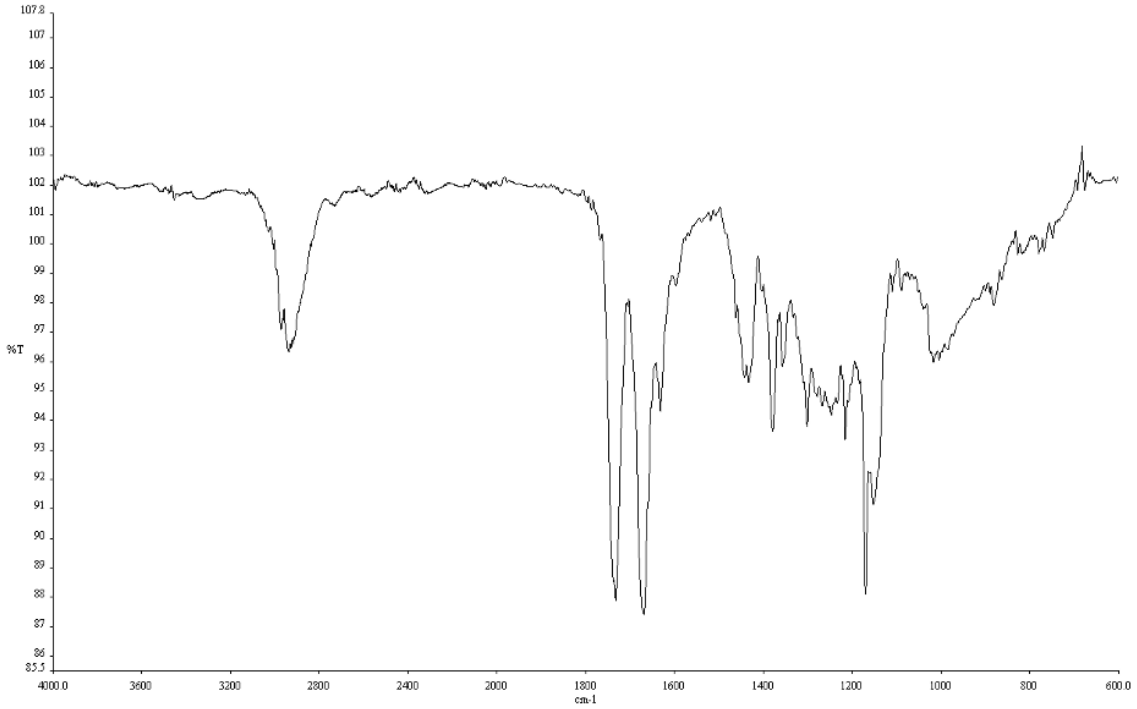

Infrared spectrum (Thin Film, NaCl) of compound **S1e**.

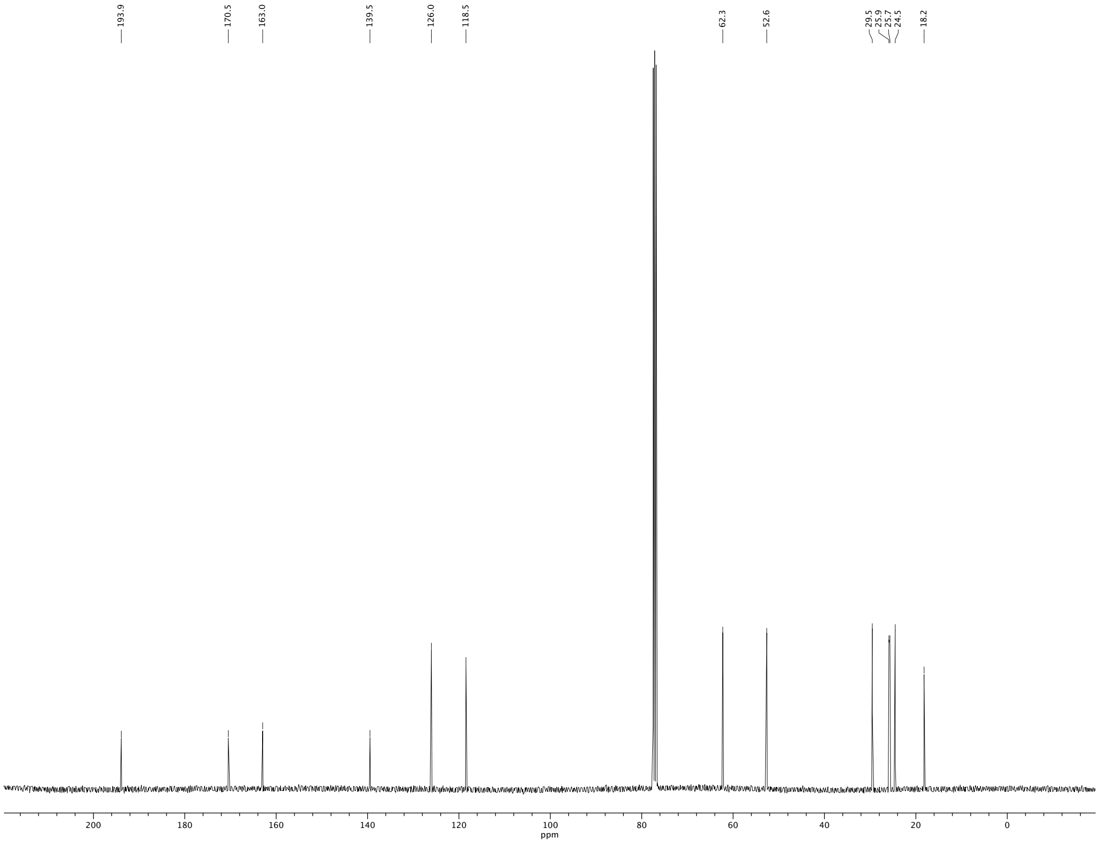

^{13}C NMR (100 MHz, $CDCl_3$) of compound **S1e**.

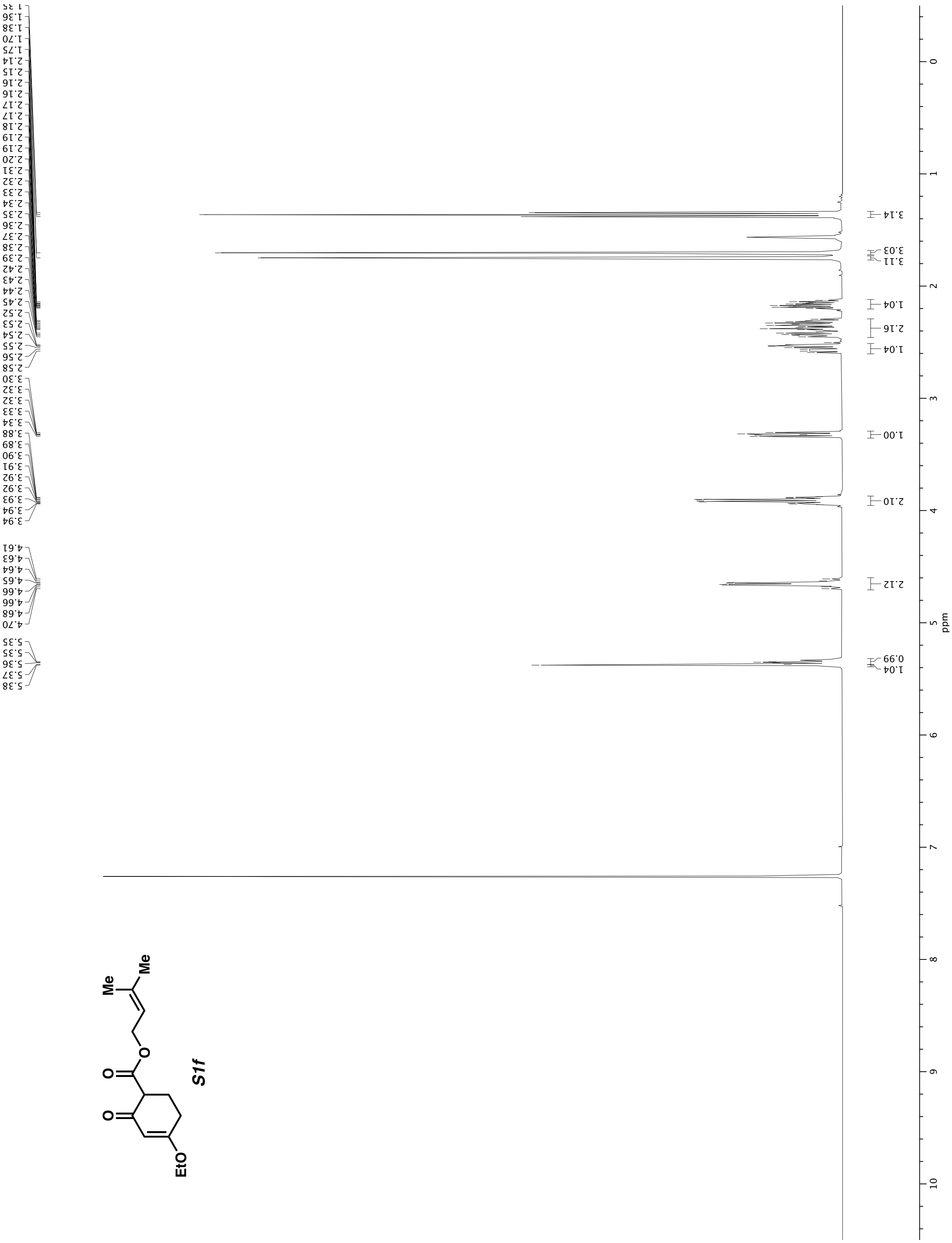

^{1}H NMR (400 MHz, $CDCl_3$) of compound **S1f**.

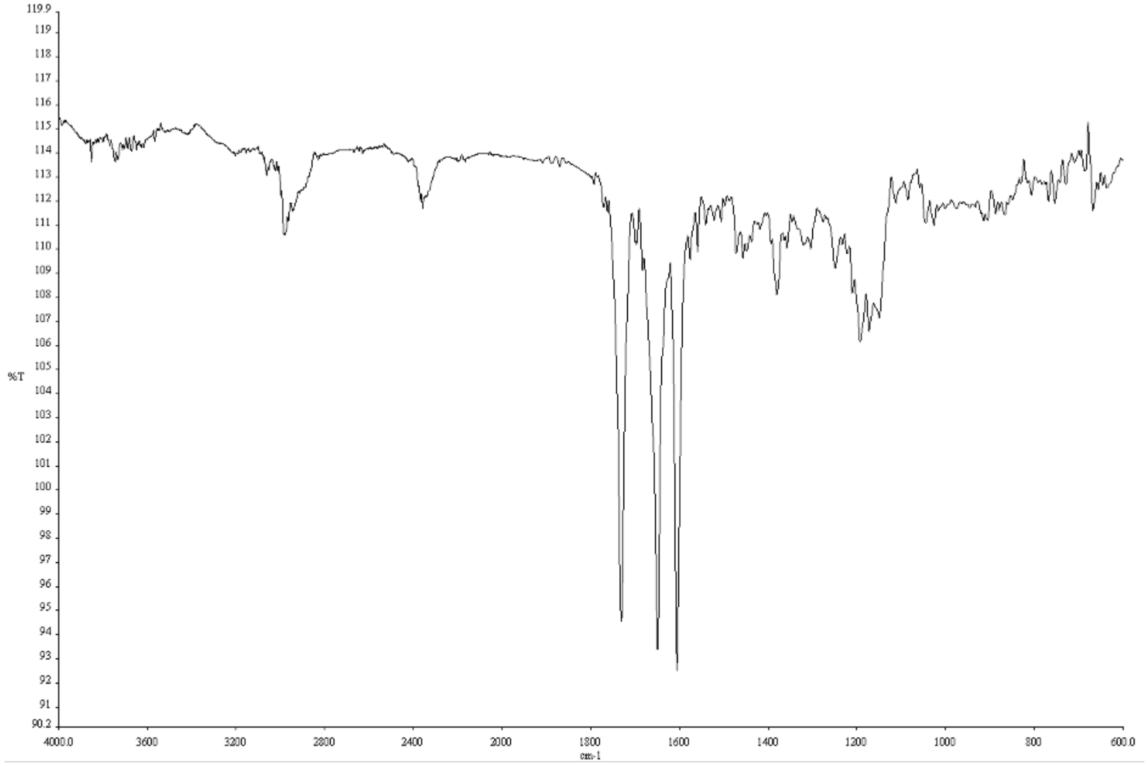

Infrared spectrum (Thin Film, NaCl) of compound **S1f**.

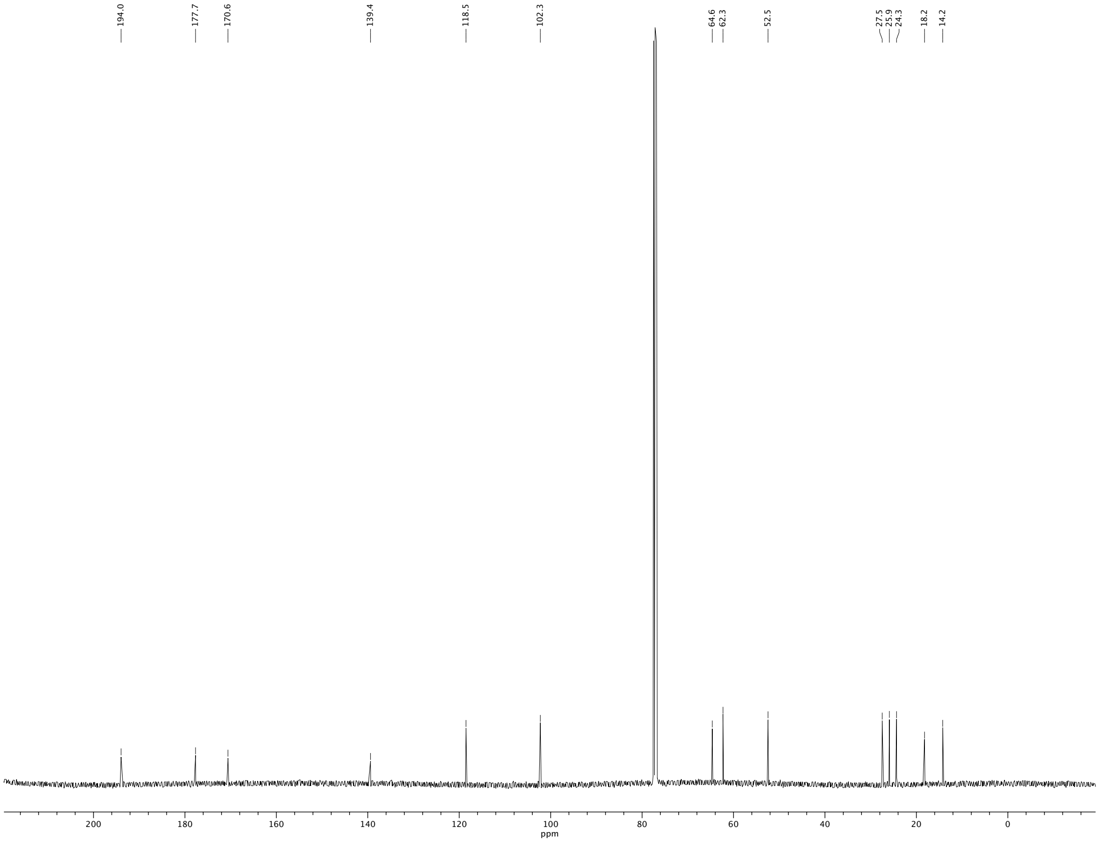

^{13}C NMR (100 MHz, $CDCl_3$) of compound **S1f**.

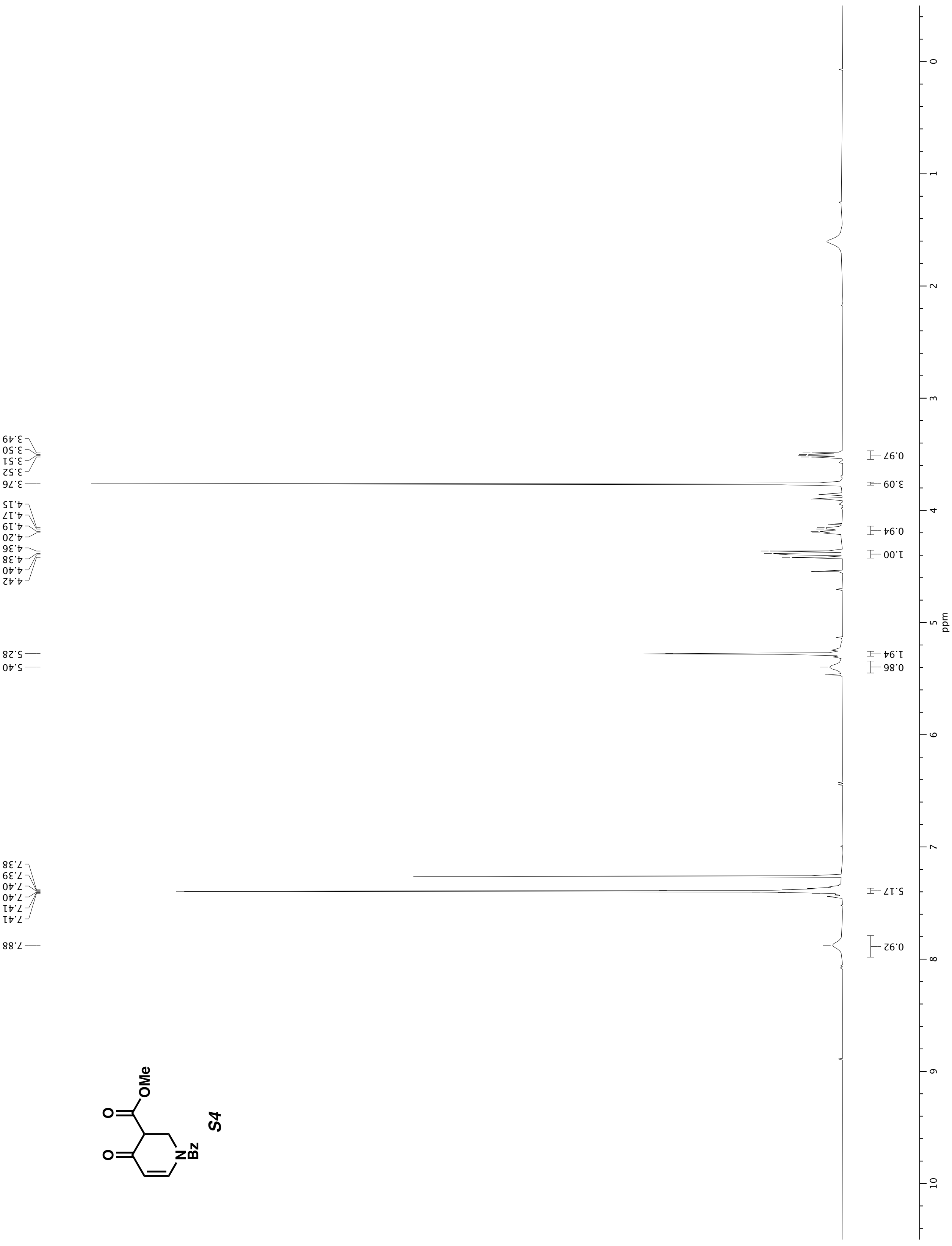

^{1}H NMR (400 MHz, $CDCl_3$) of compound **S4**.

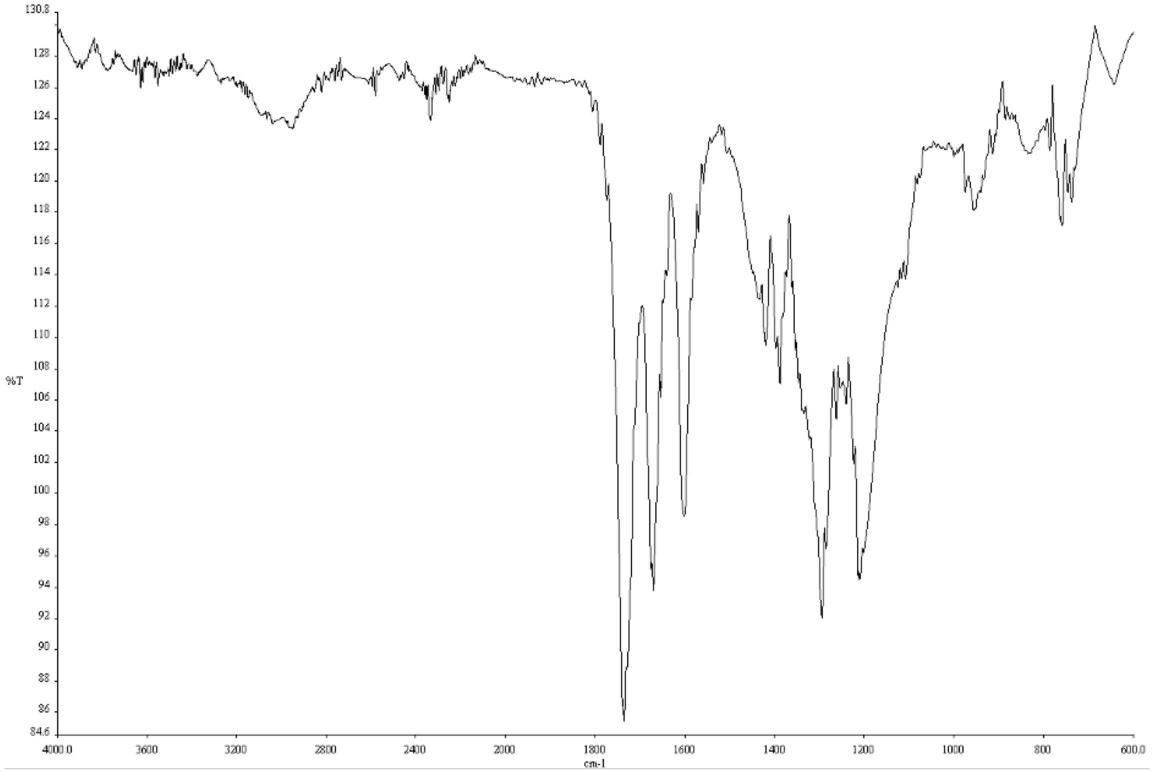

Infrared spectrum (Thin Film, NaCl) of compound **S4**.

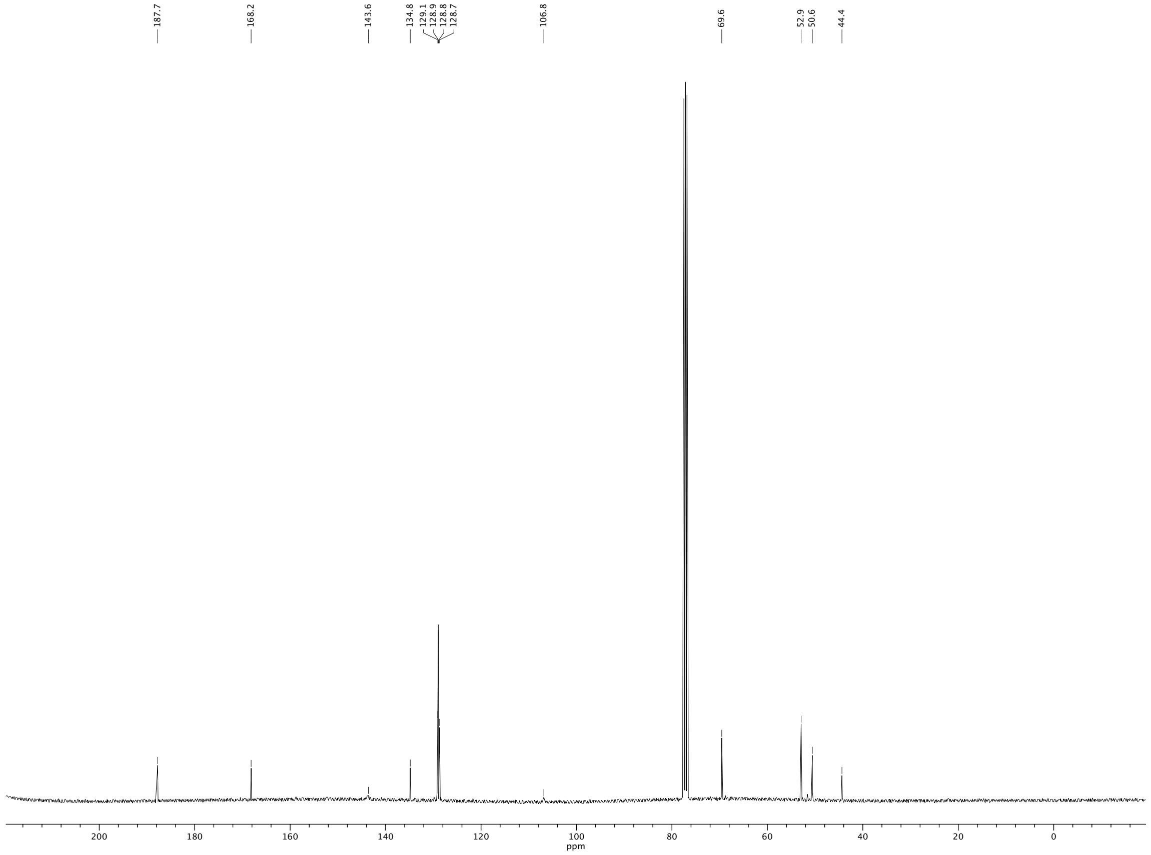

^{13}C NMR (100 MHz, $CDCl_3$) of compound **S4**.

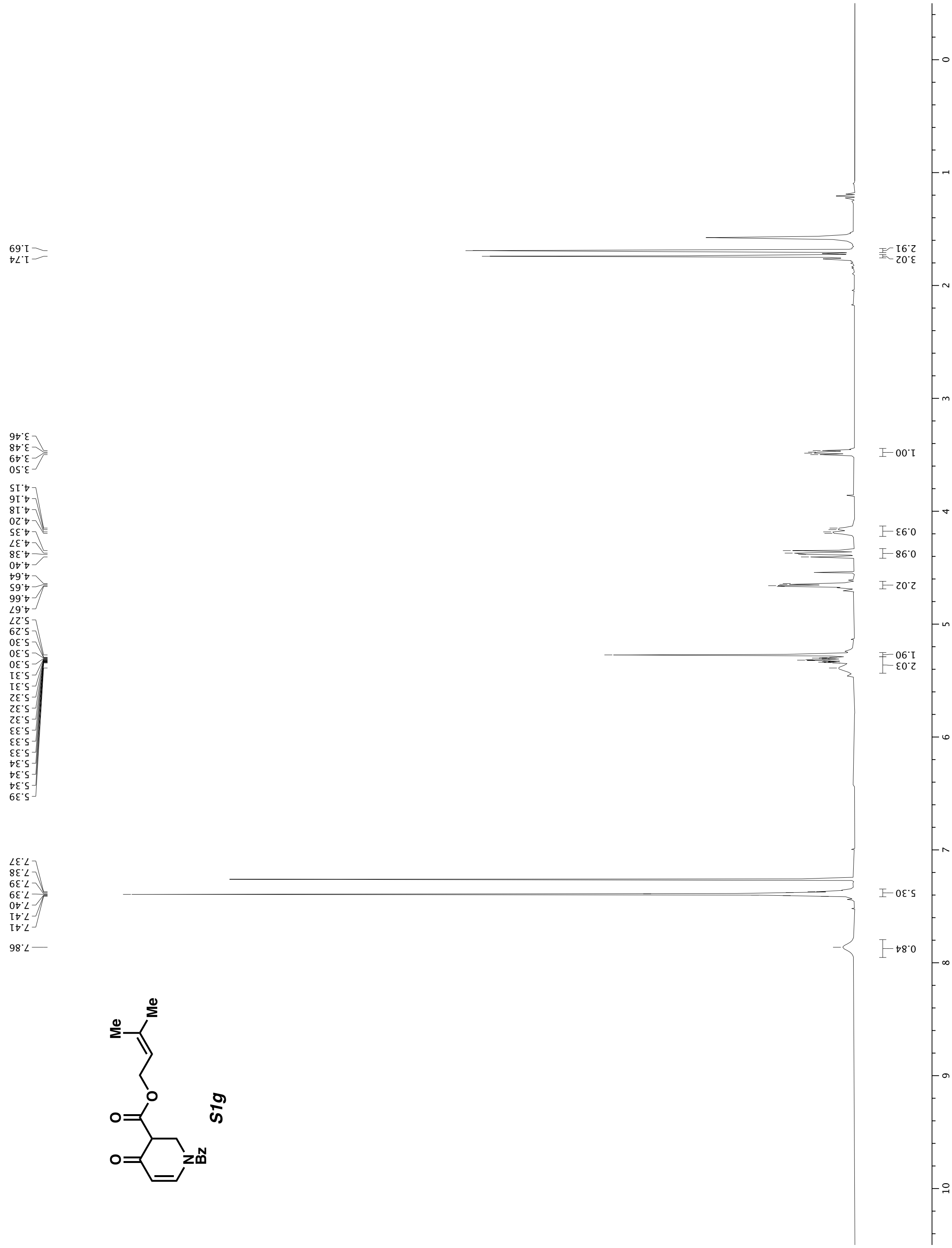

^{1}H NMR (400 MHz, $CDCl_3$) of compound **S1g**.

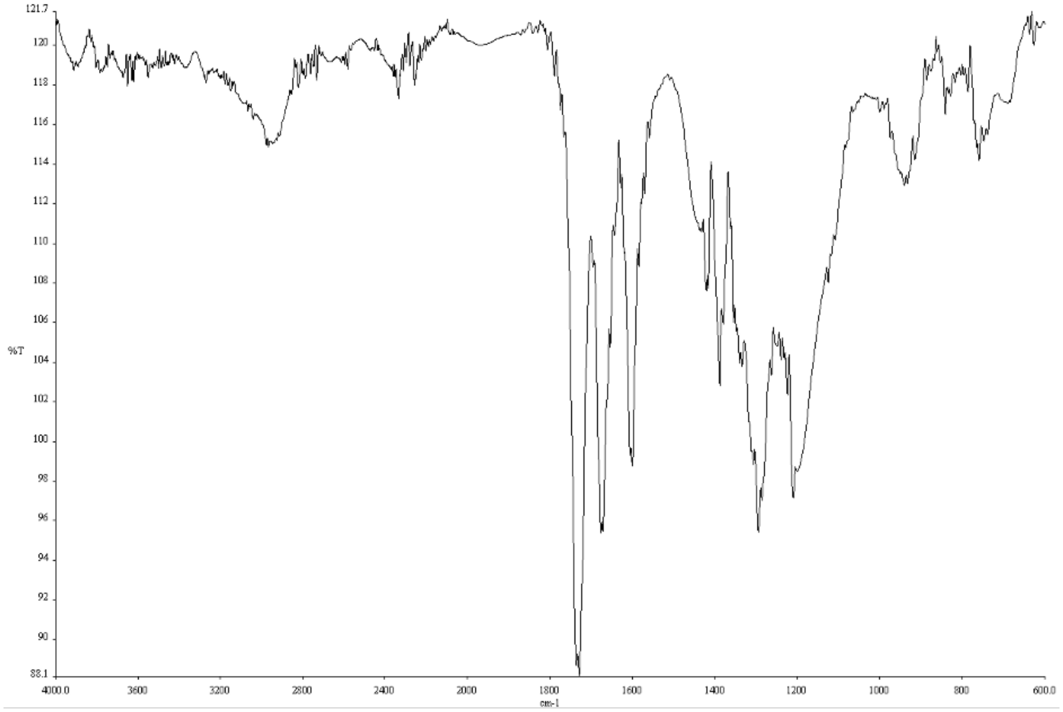

Infrared spectrum (Thin Film, NaCl) of compound **S1g**.

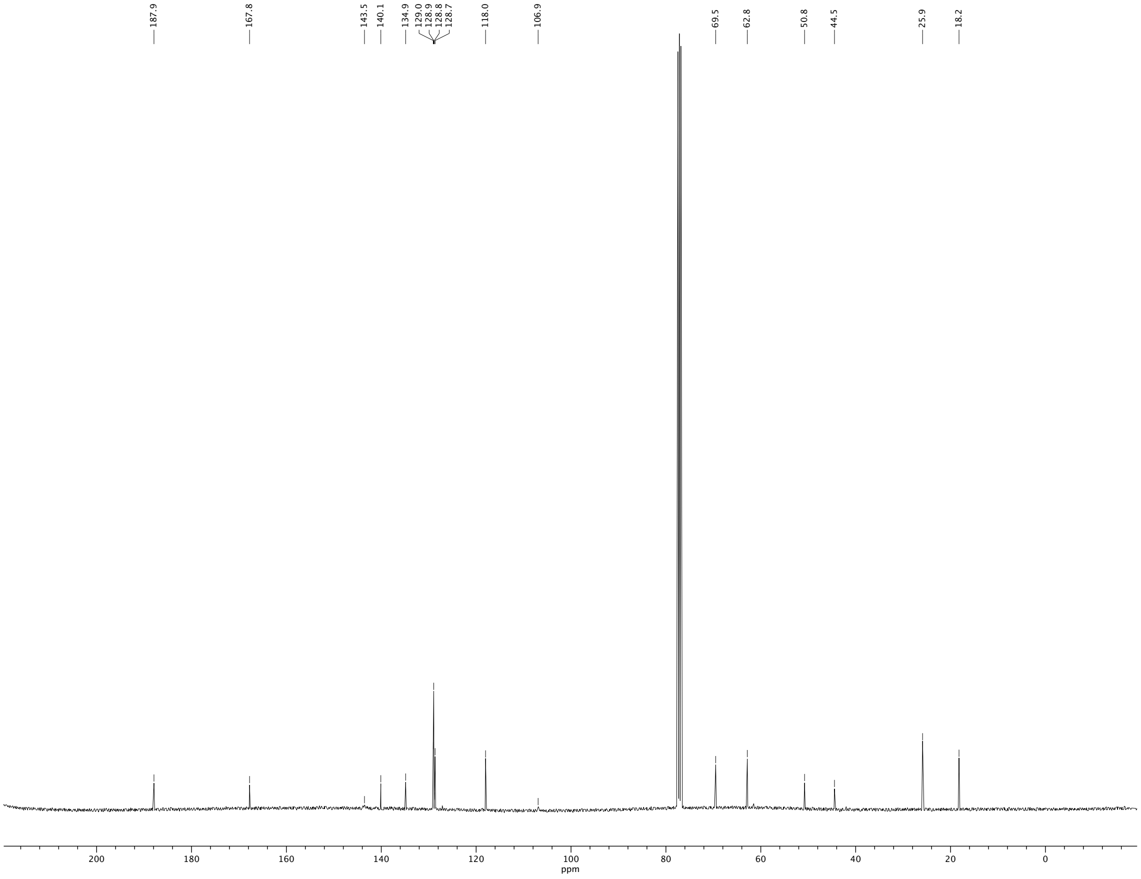

^{13}C NMR (100 MHz, $CDCl_3$) of compound **S1g**.

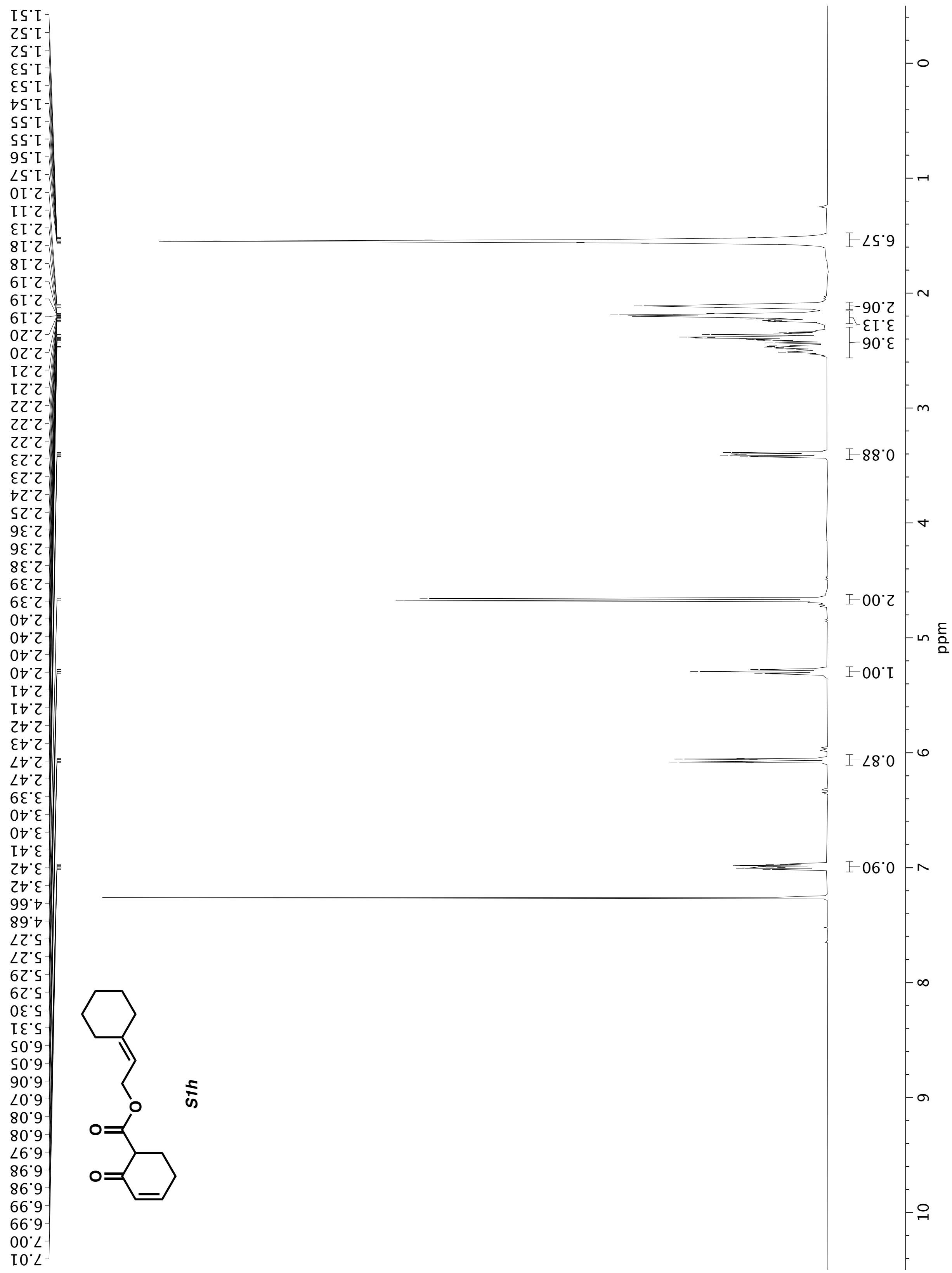

^{1}H NMR (400 MHz, $CDCl_3$) of compound **S1h**.

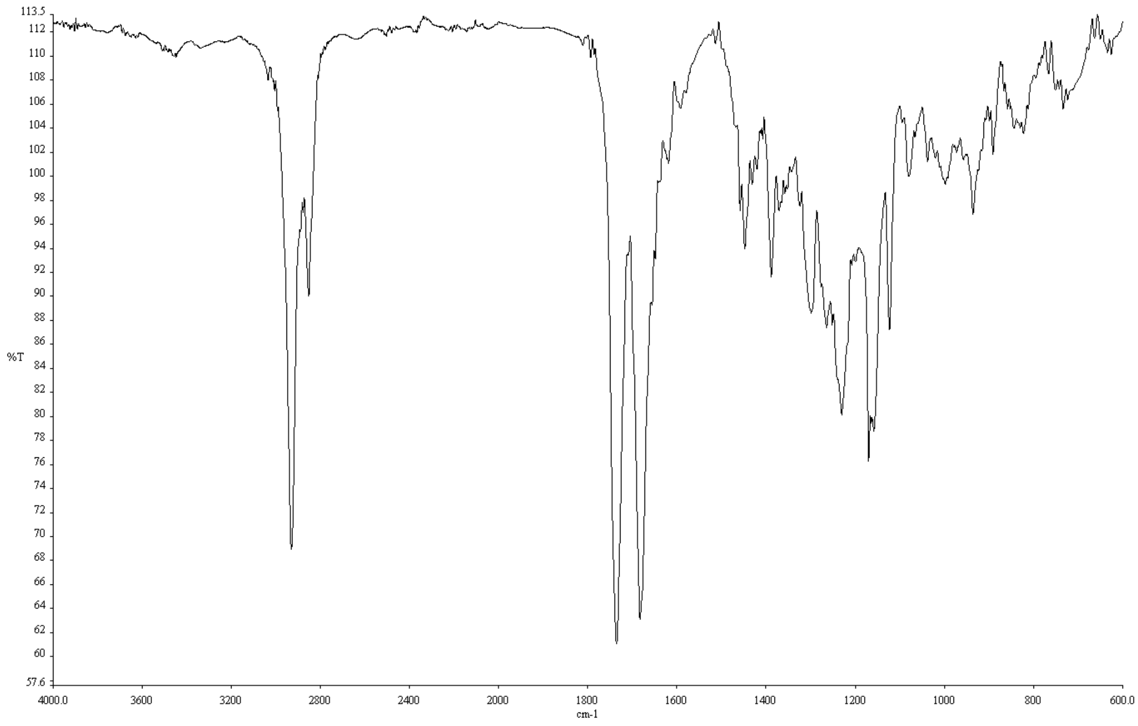

Infrared spectrum (Thin Film, NaCl) of compound **S1h**.

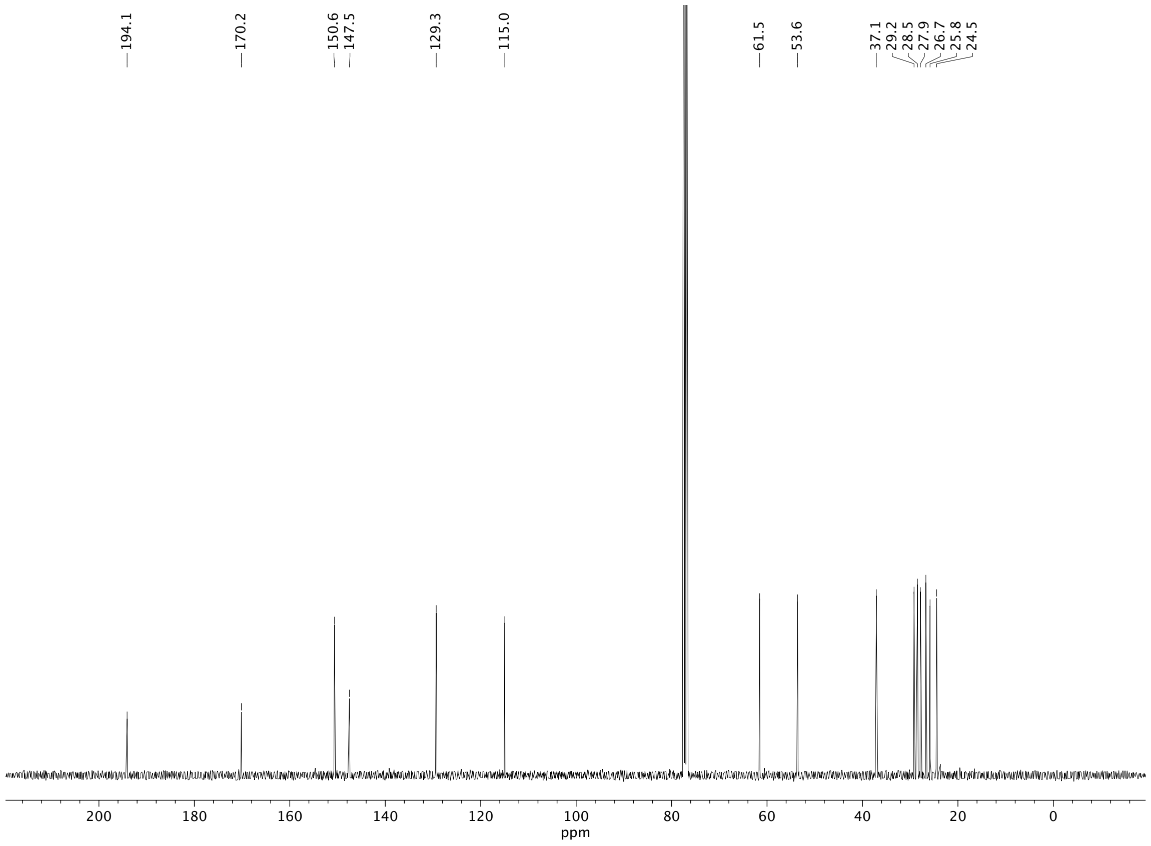

^{13}C NMR (100 MHz, $CDCl_3$) of compound **S1h**.

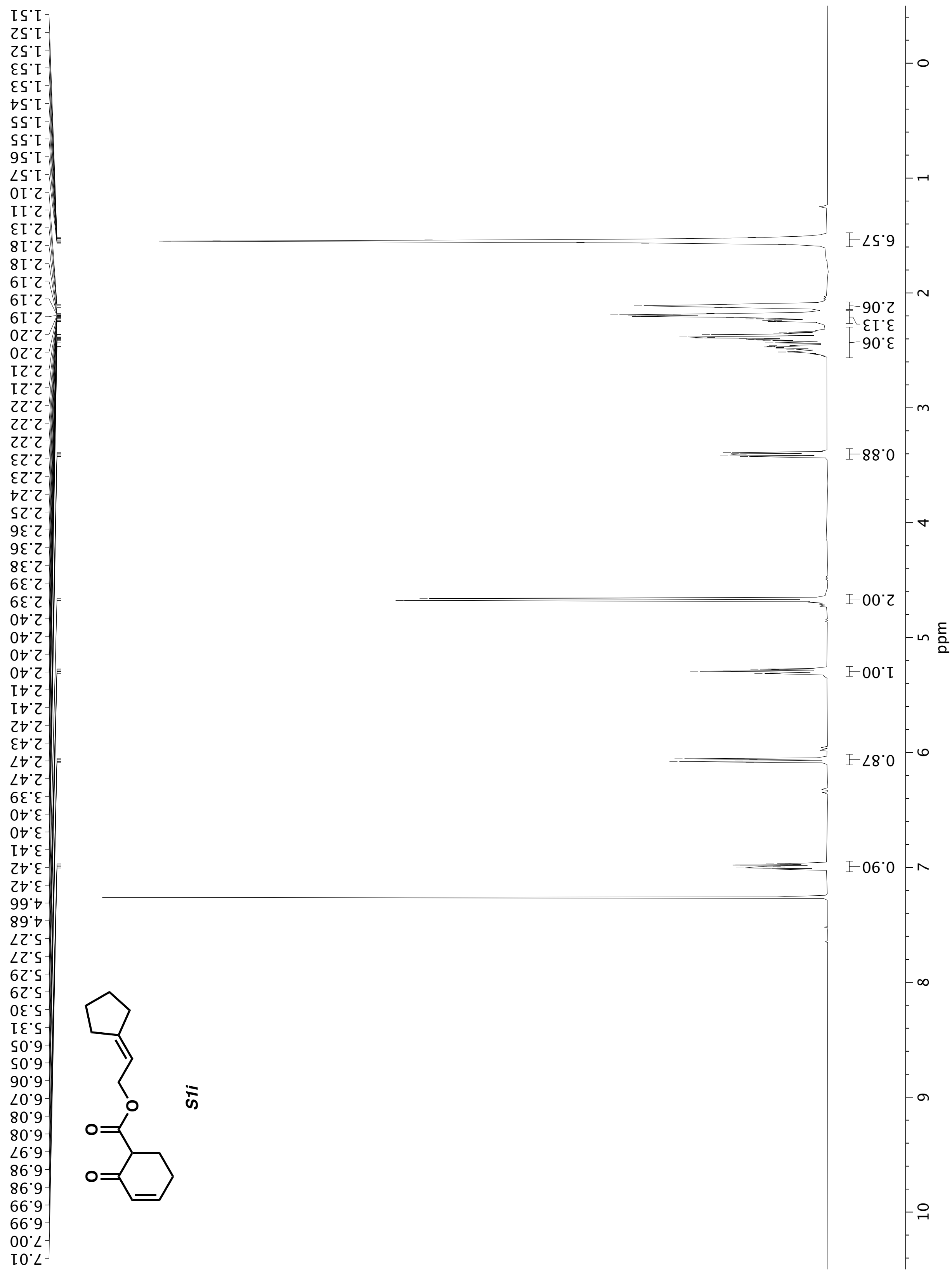

^{1}H NMR (400 MHz, $CDCl_3$) of compound **S1i.**

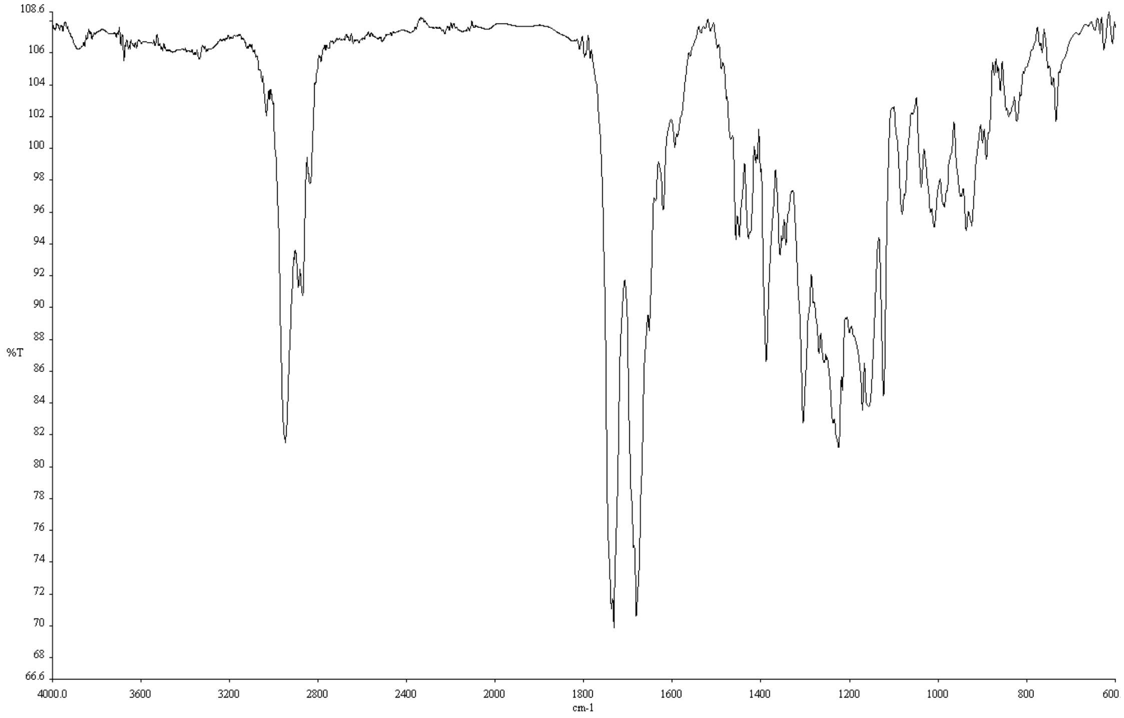

Infrared spectrum (Thin Film, NaCl) of compound **S1i**.

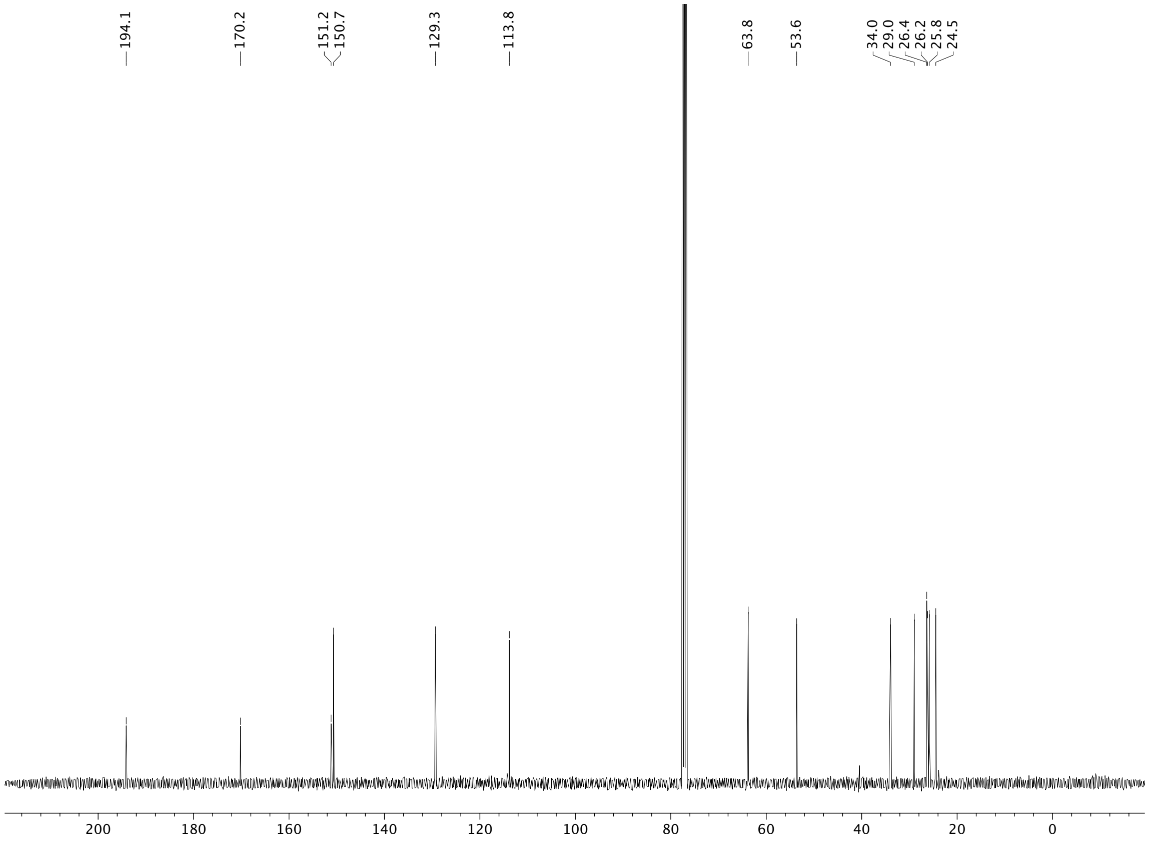

^{13}C NMR (100 MHz, $CDCl_3$) of compound **S1i**.

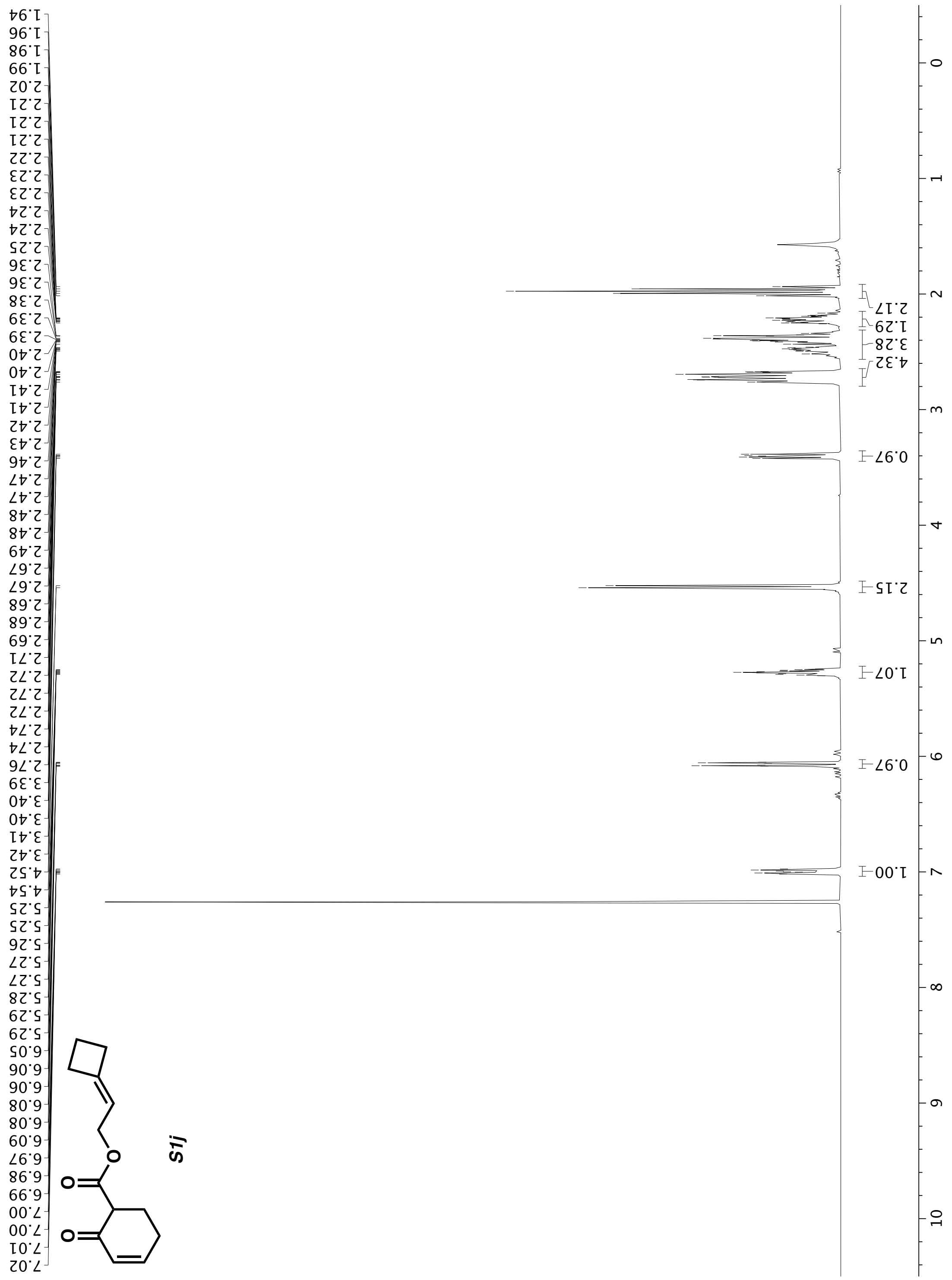

^{1}H NMR (400 MHz, $CDCl_3$) of compound **S1j**.

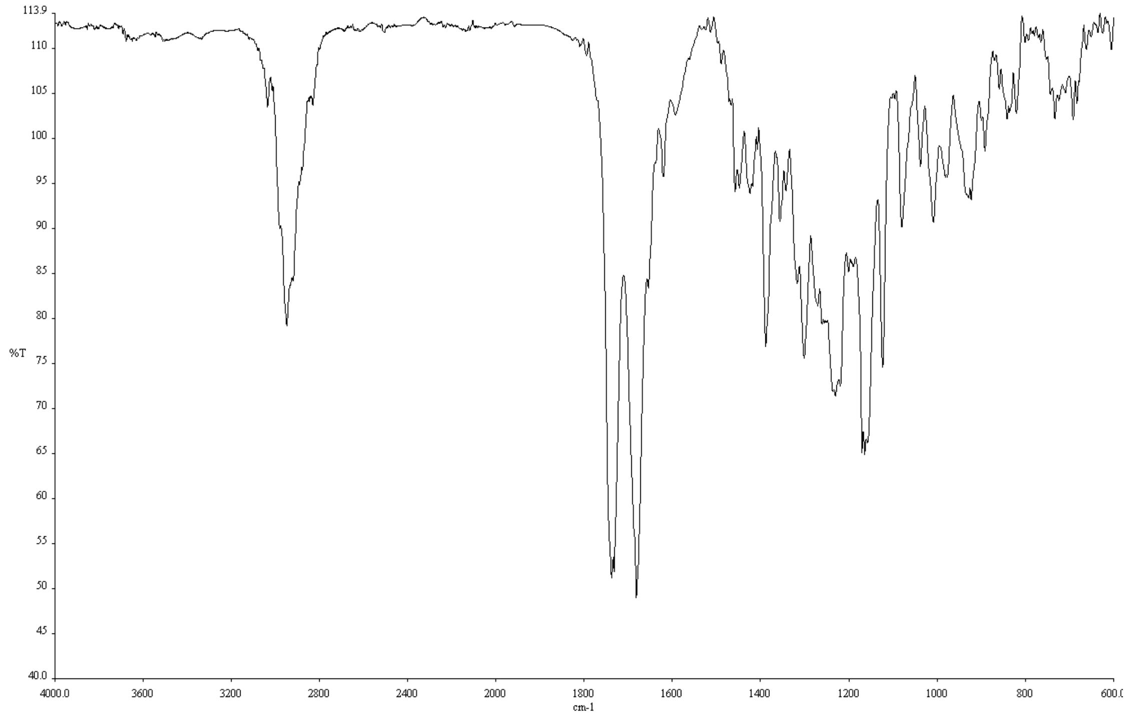

Infrared spectrum (Thin Film, NaCl) of compound **S1j**.

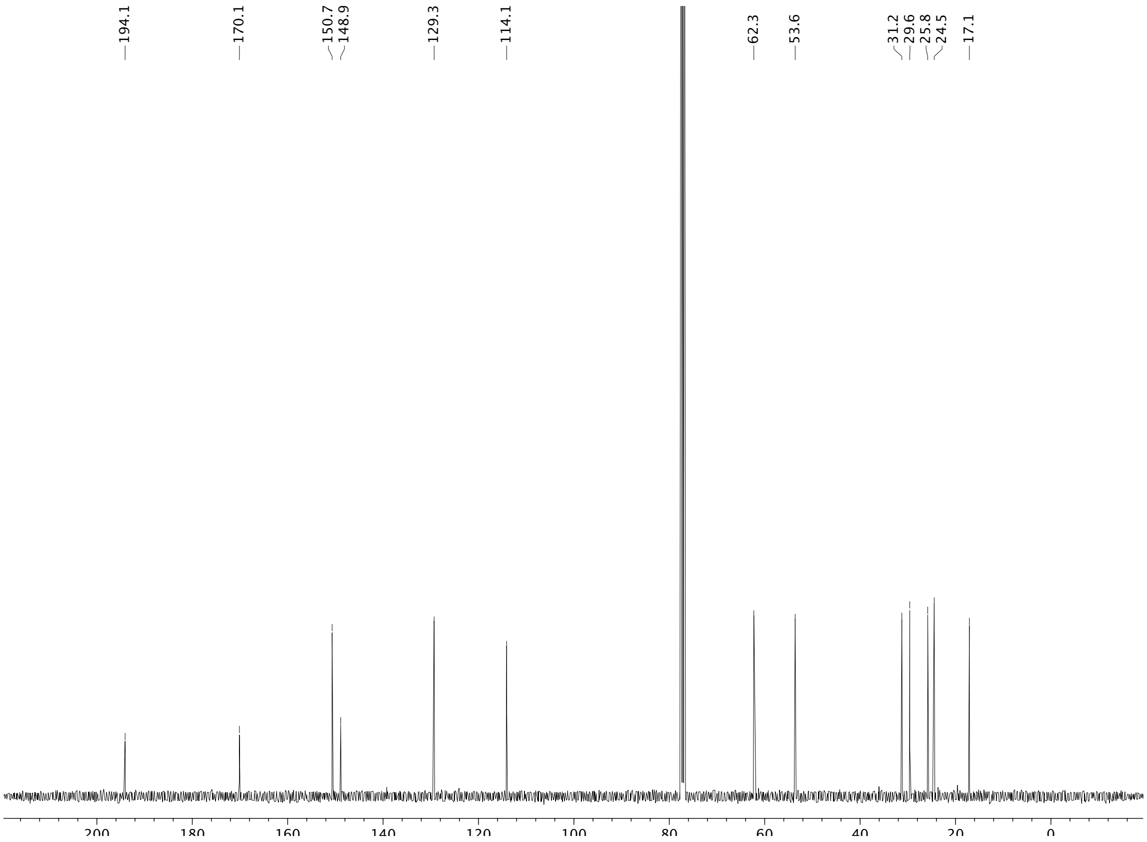

^{13}C NMR (100 MHz, $CDCl_3$) of compound **S1j**.

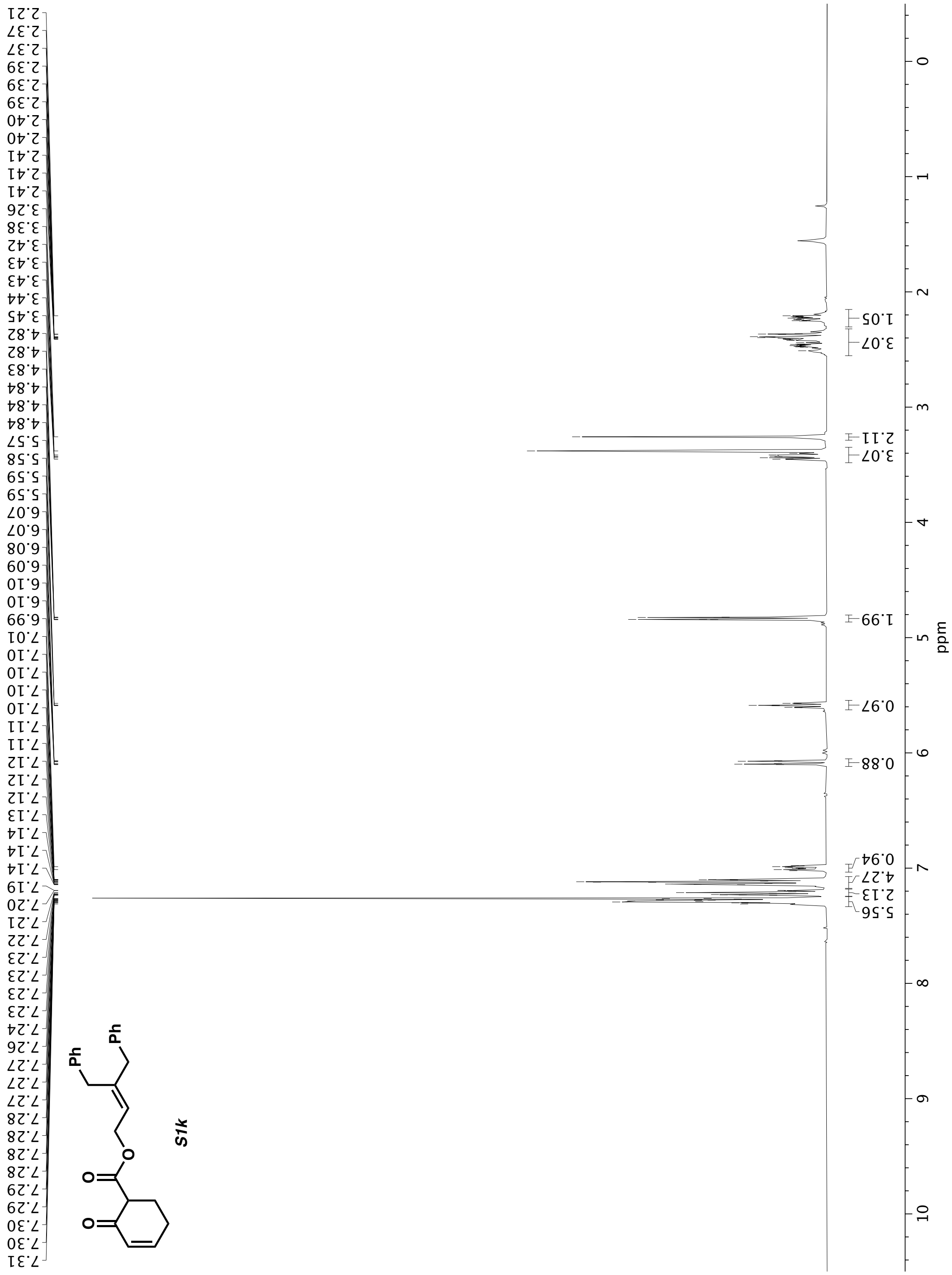

S1k

^{1}H NMR (400 MHz, $CDCl_3$) of compound **S1k**.

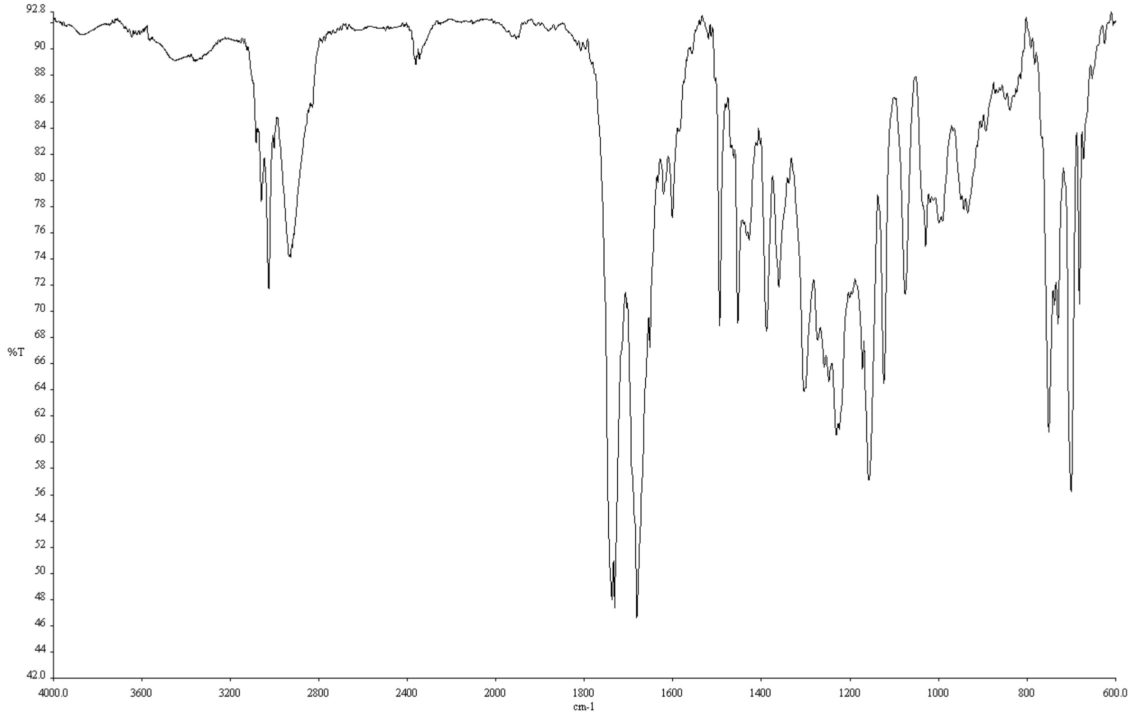

Infrared spectrum (Thin Film, NaCl) of compound **S1k**.

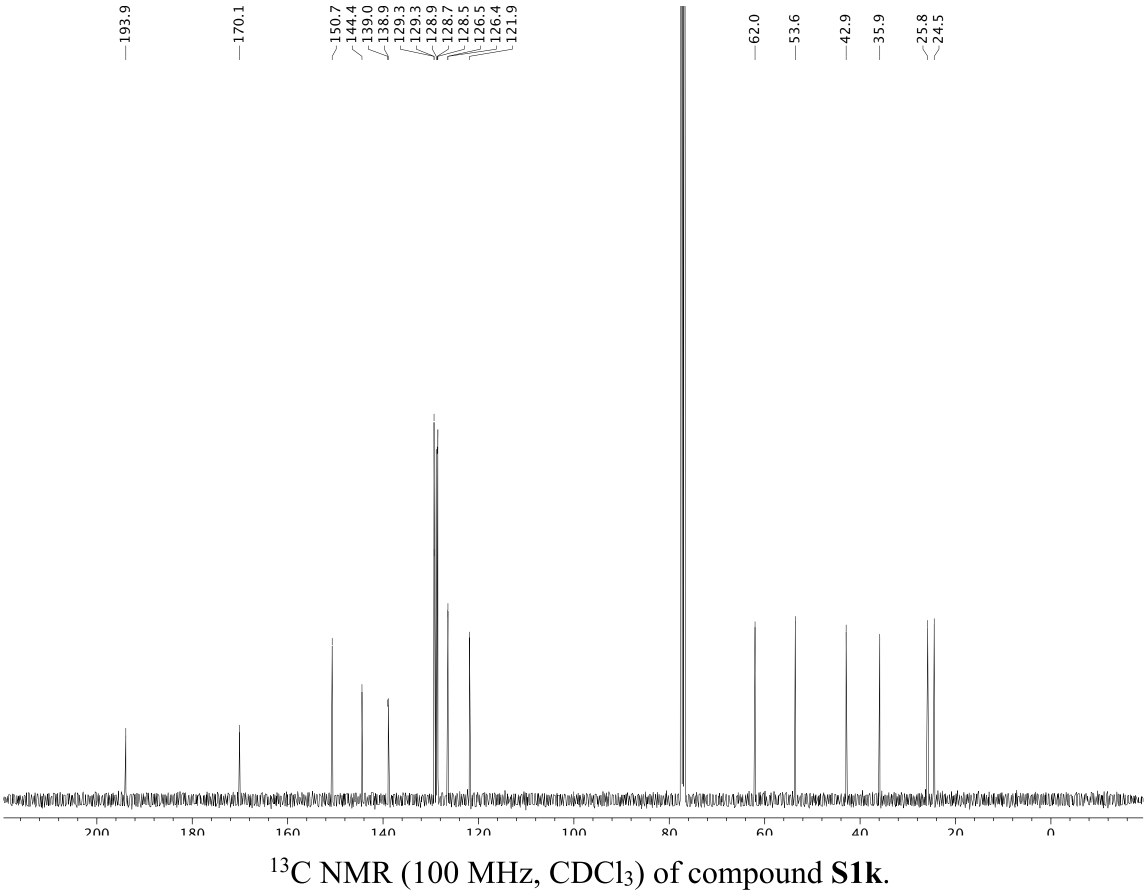

^{13}C NMR (100 MHz, $CDCl_3$) of compound **S1k**.

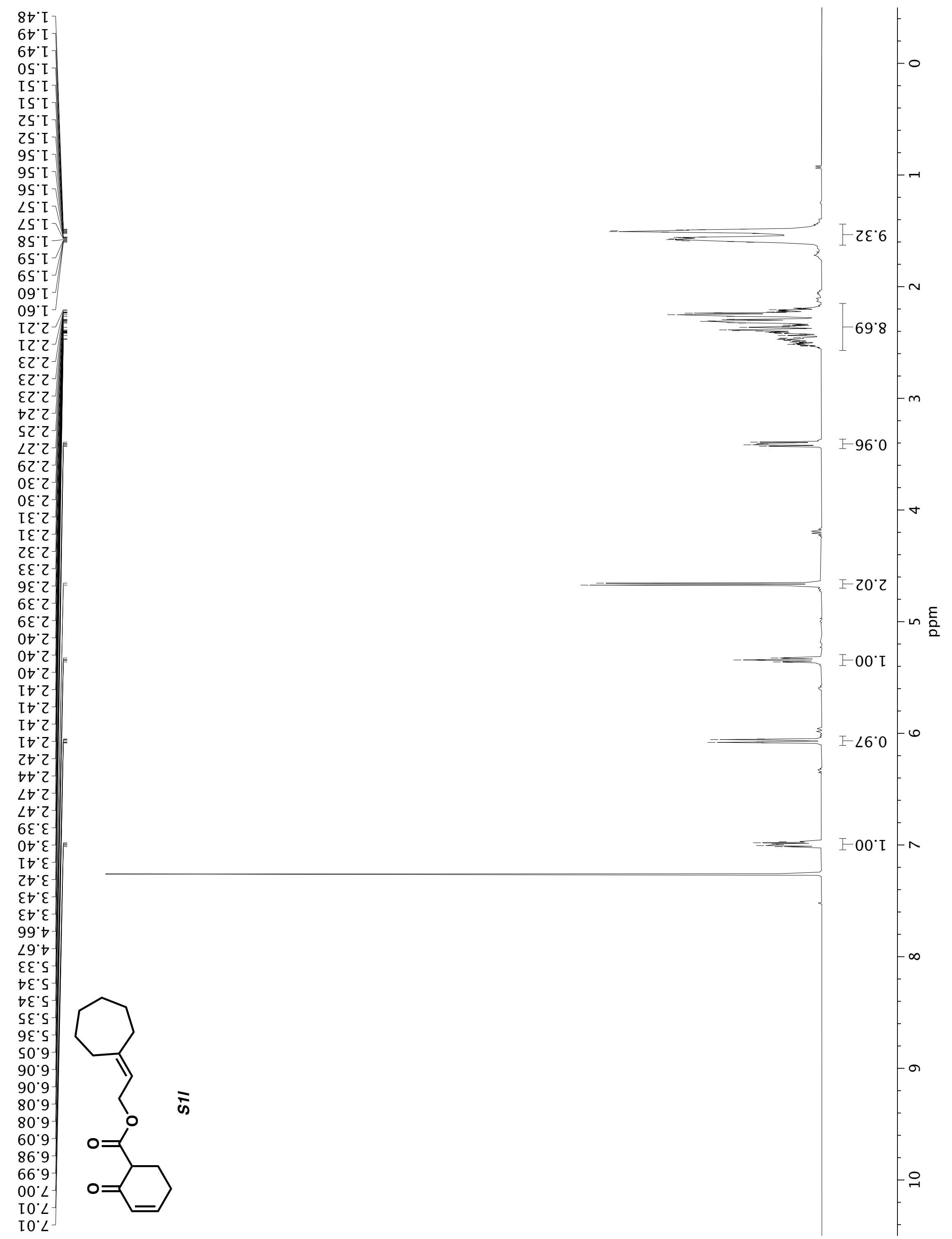

^{1}H NMR (400 MHz, $CDCl_3$) of compound **S11**.

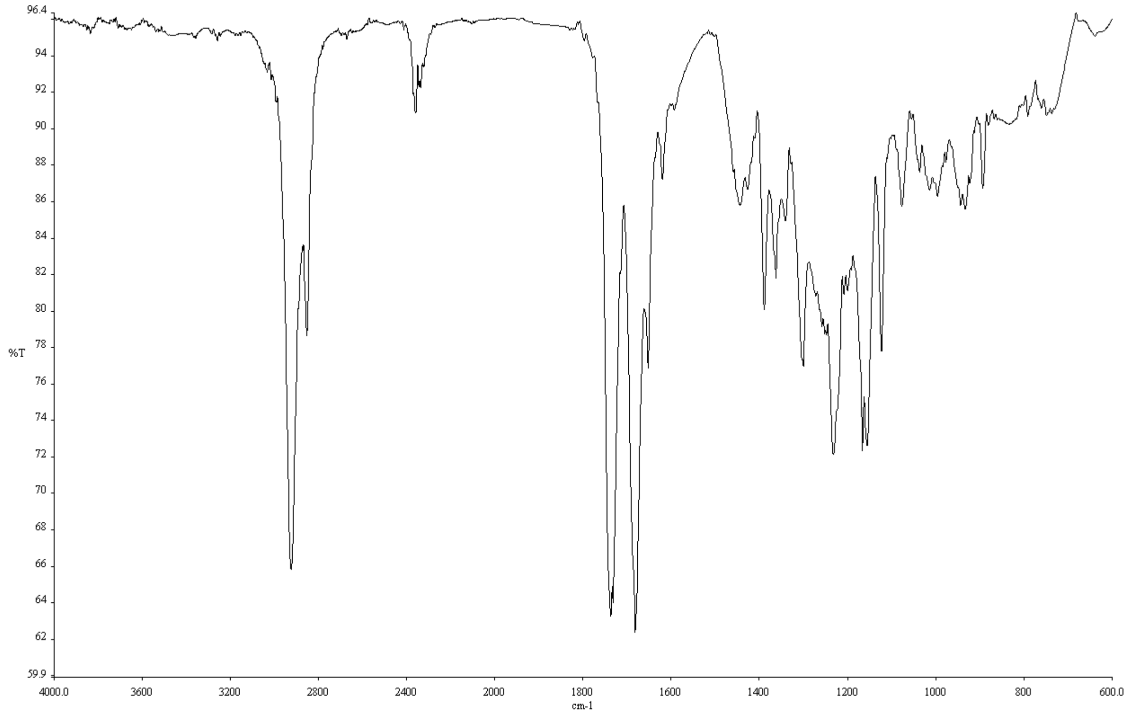

Infrared spectrum (Thin Film, NaCl) of compound **S1l**.

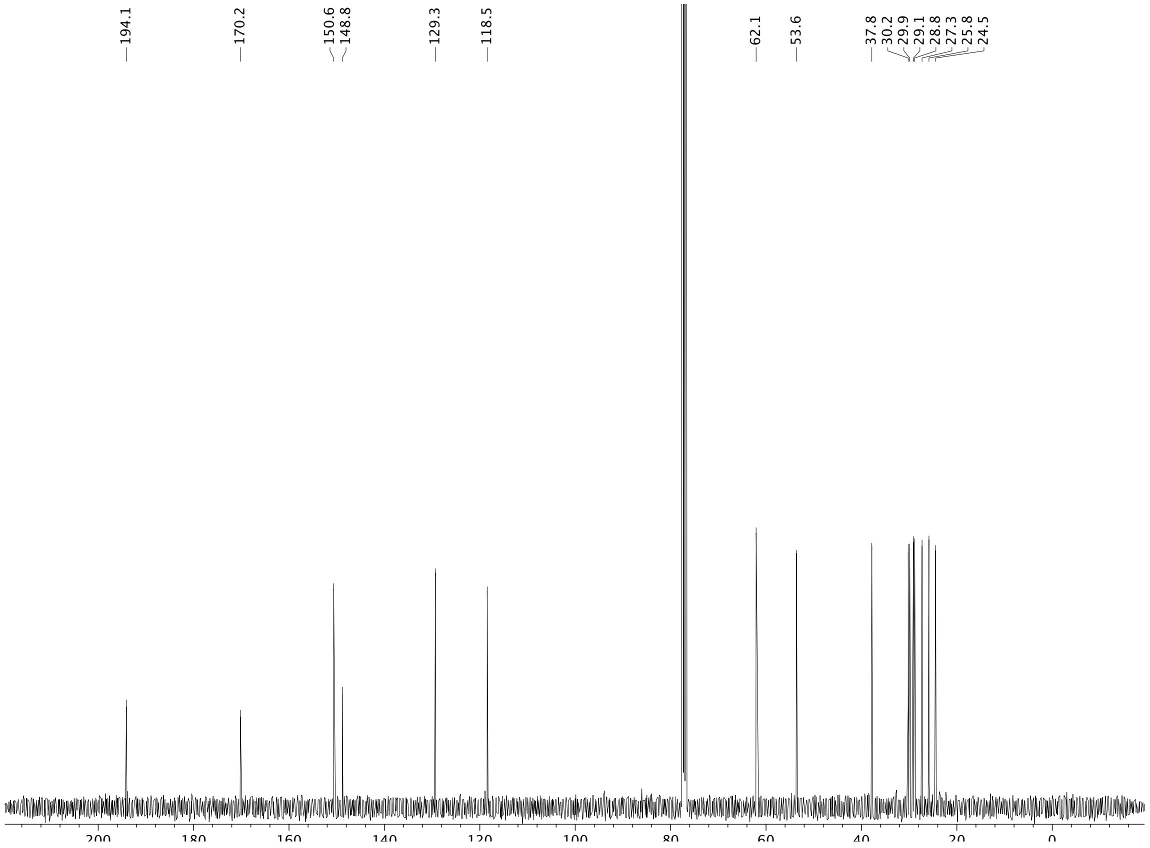

^{13}C NMR (100 MHz, $CDCl_3$) of compound **S1l**.

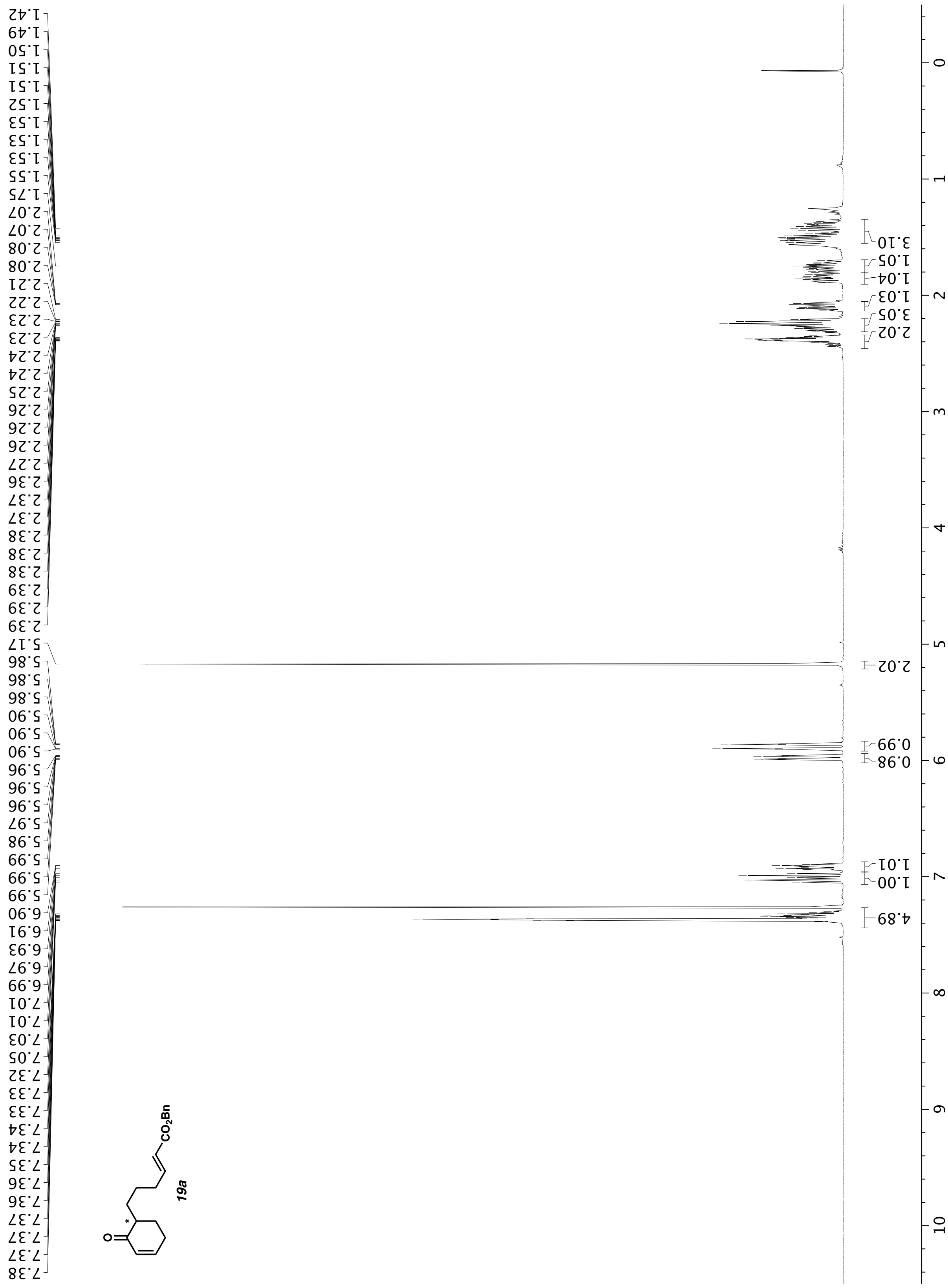

^{1}H NMR (400 MHz, $CDCl_3$) of compound **19a.**

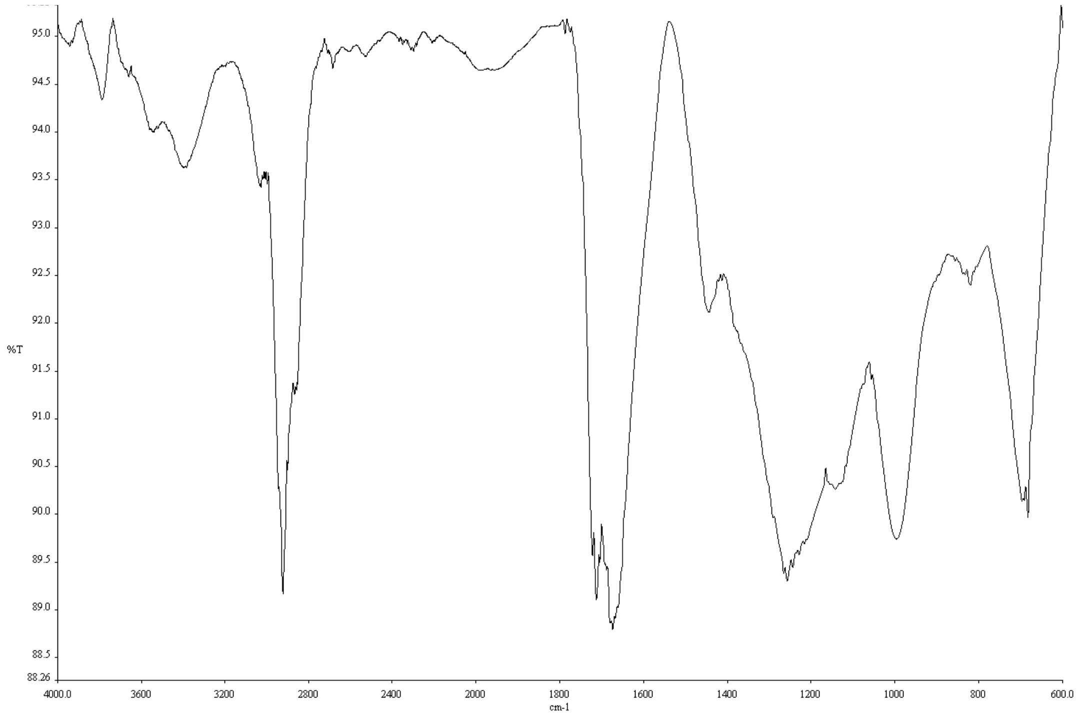

Infrared spectrum (Thin Film, NaCl) of compound **19a**.

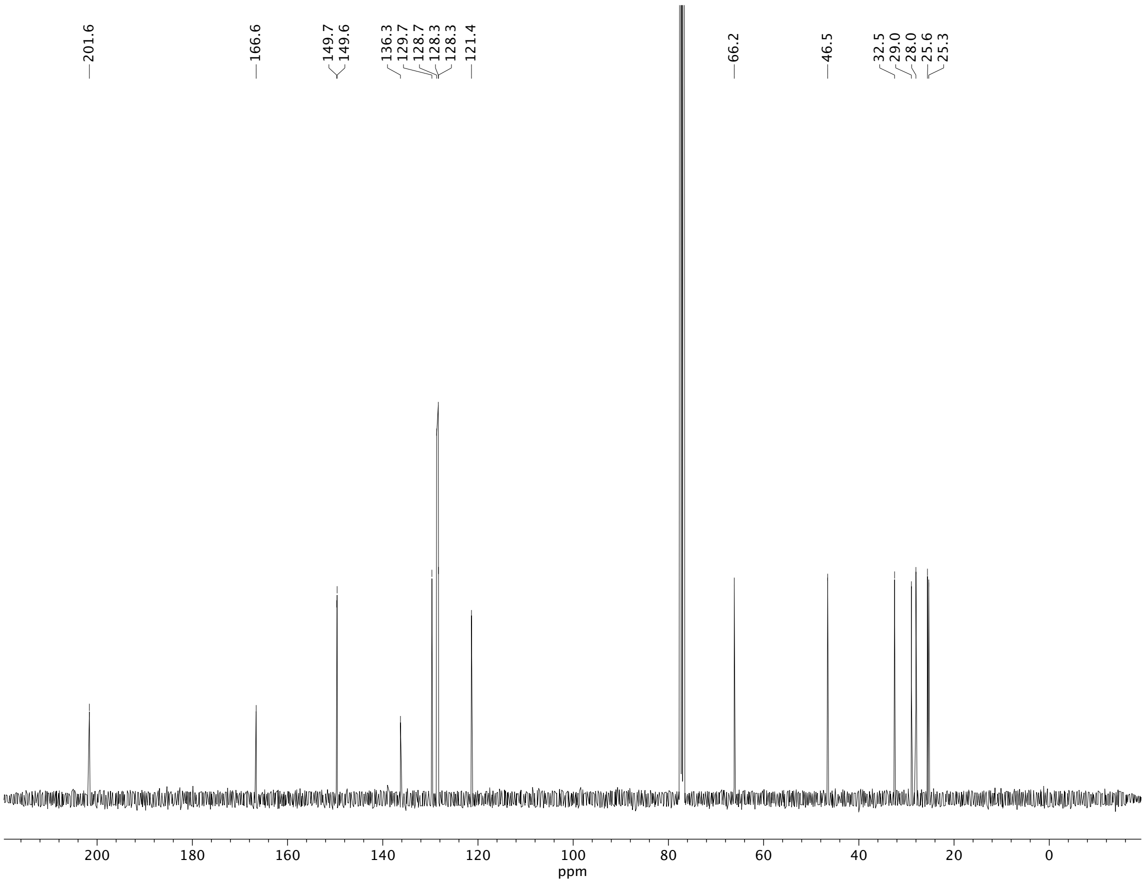

^{13}C NMR (100 MHz, $CDCl_3$) of compound **19a**.

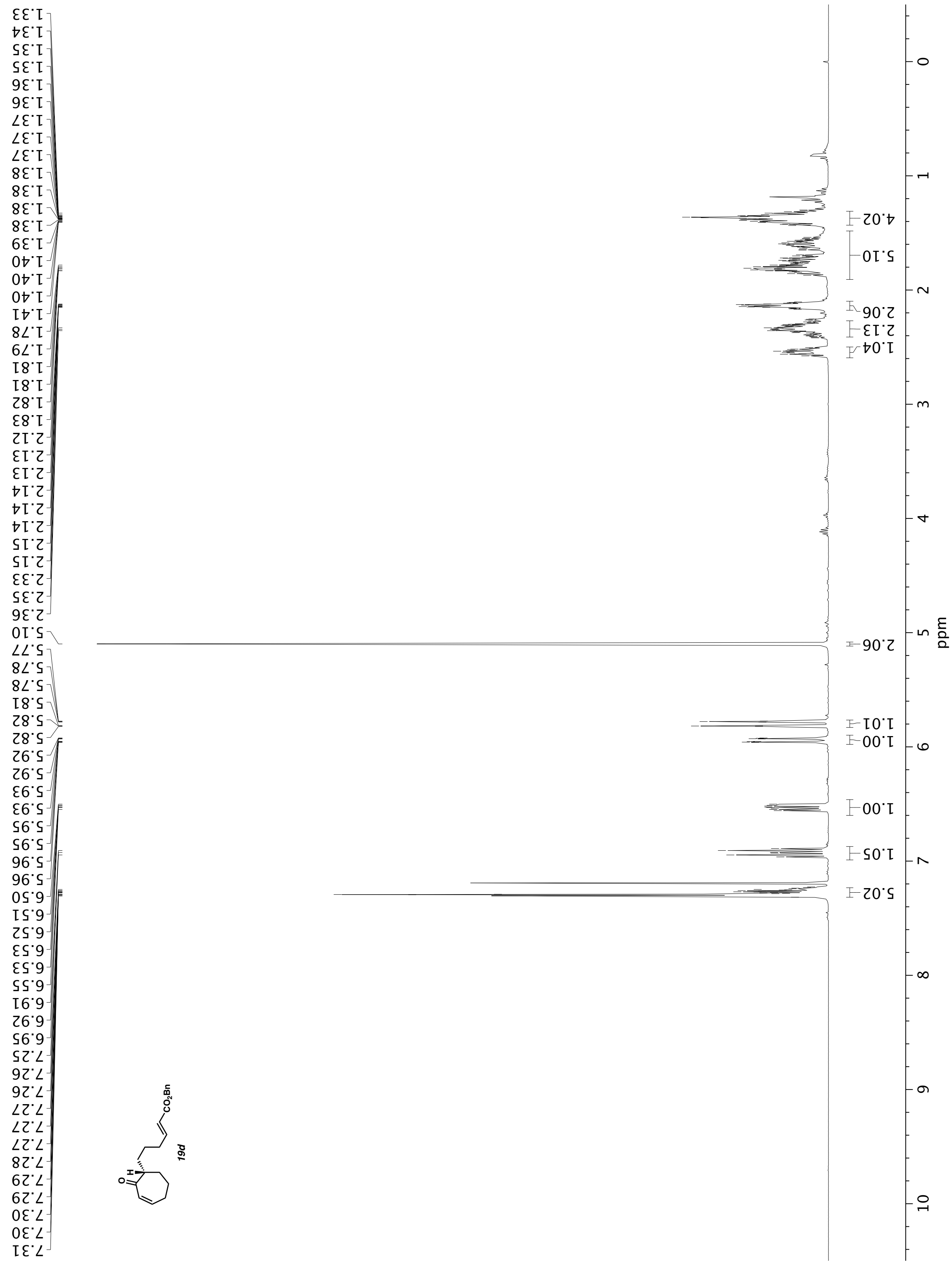

^{1}H NMR (400 MHz, $CDCl_3$) of compound **19d.**

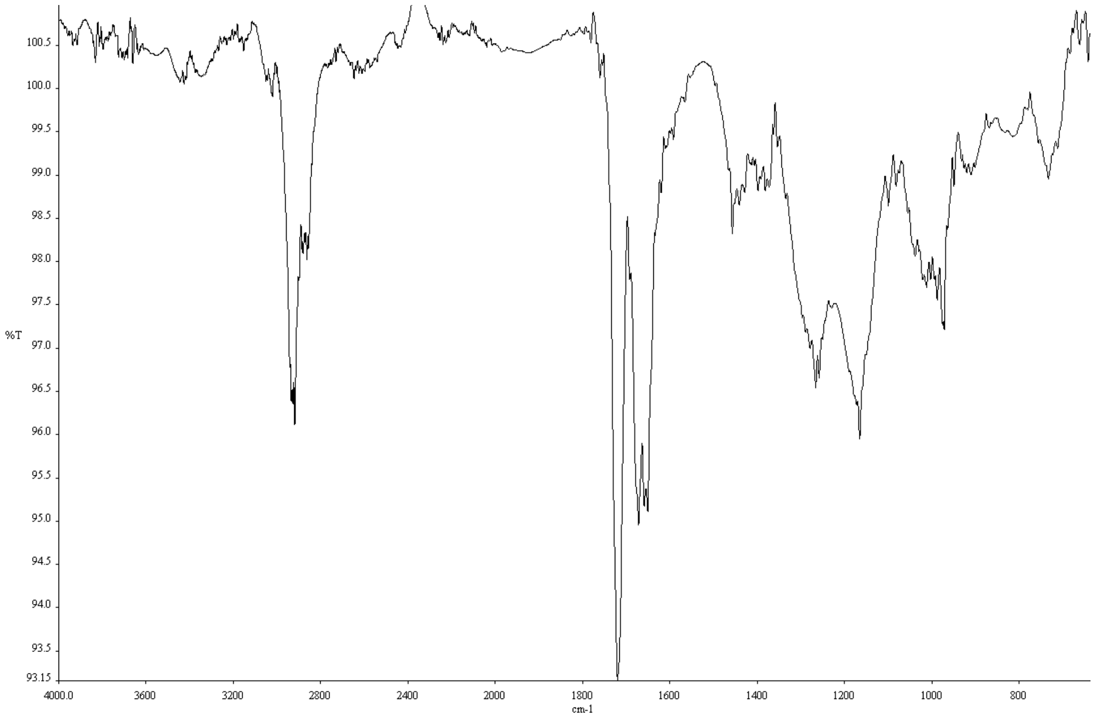

Infrared spectrum (Thin Film, NaCl) of compound **19d**.

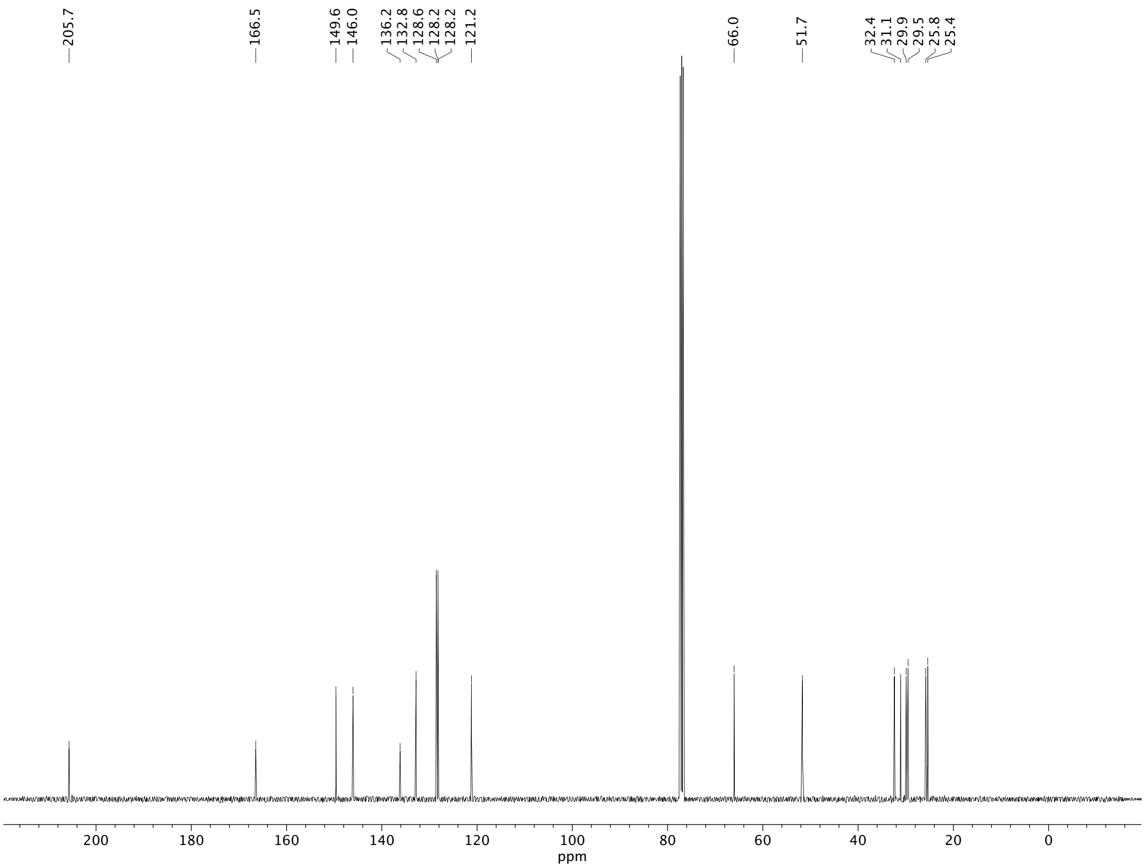

^{13}C NMR (100 MHz, $CDCl_3$) of compound **19d**.

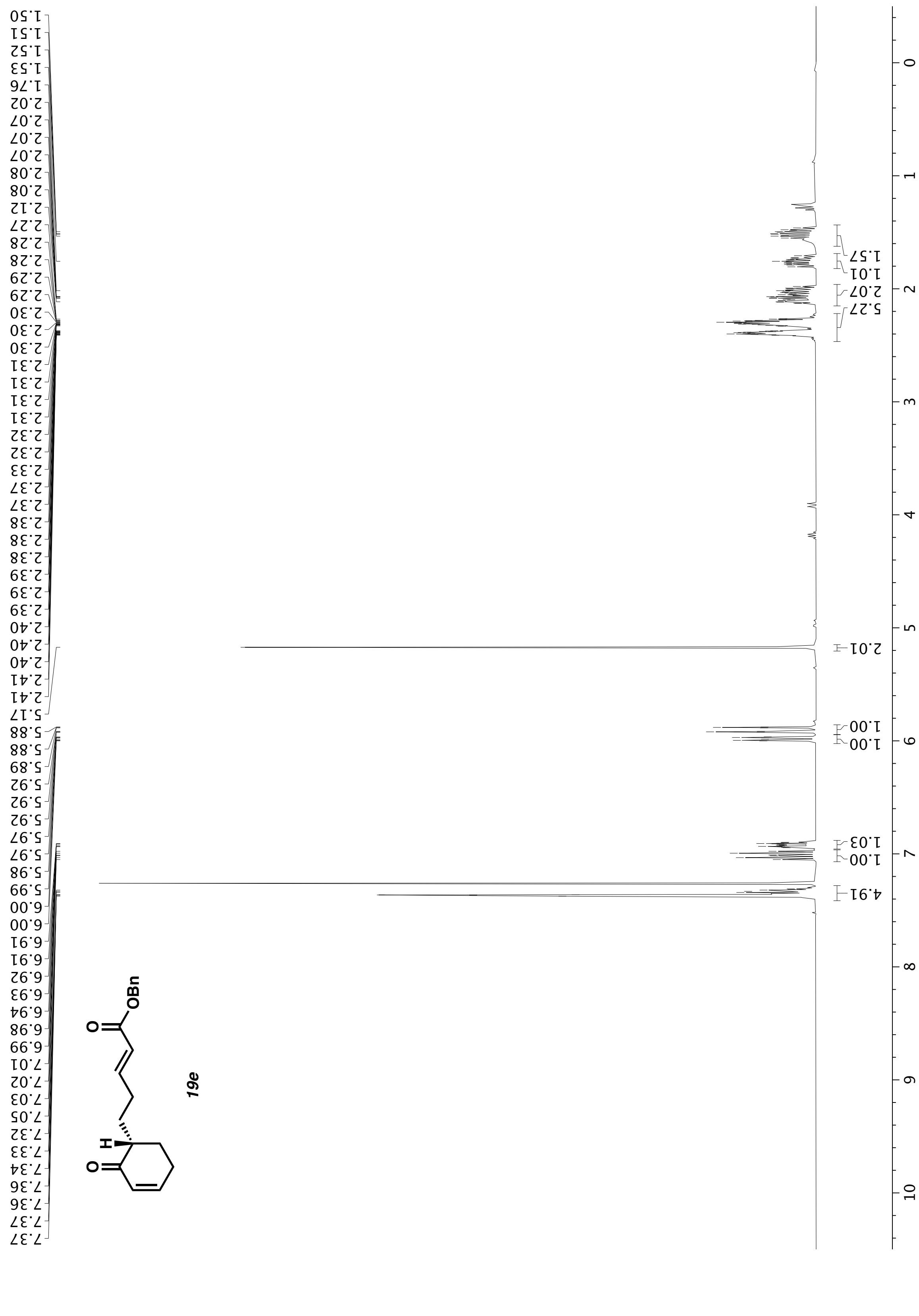

^{1}H NMR (400 MHz, $CDCl_3$) of compound **19e**.

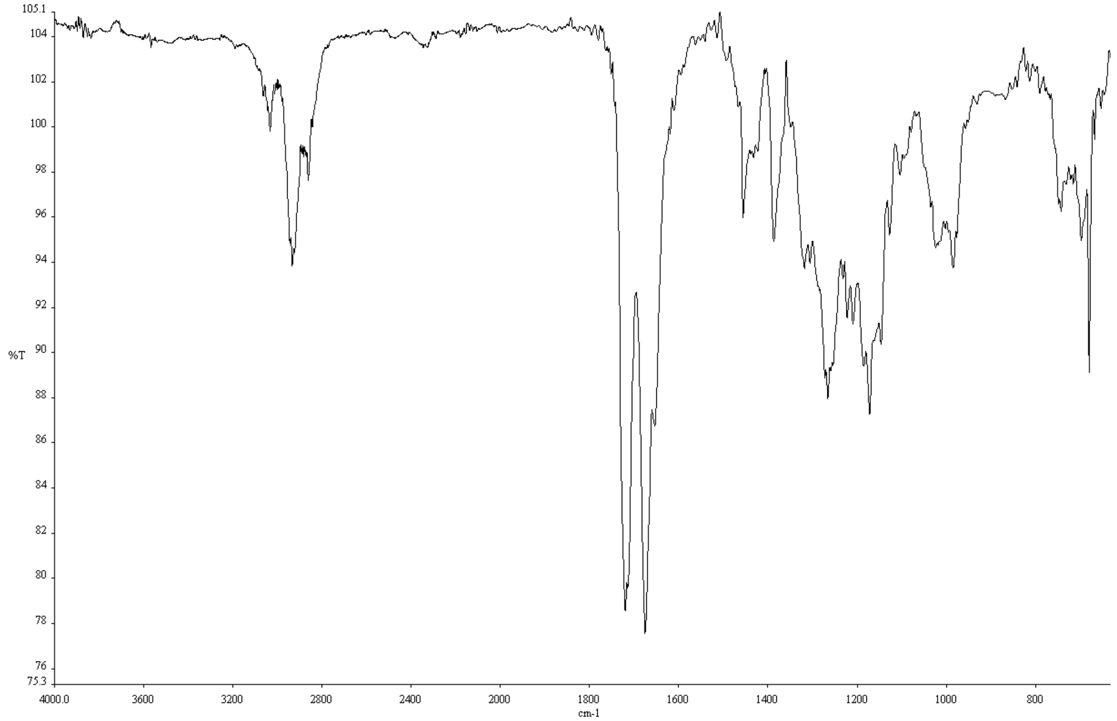

Infrared spectrum (Thin Film, NaCl) of compound **19e**.

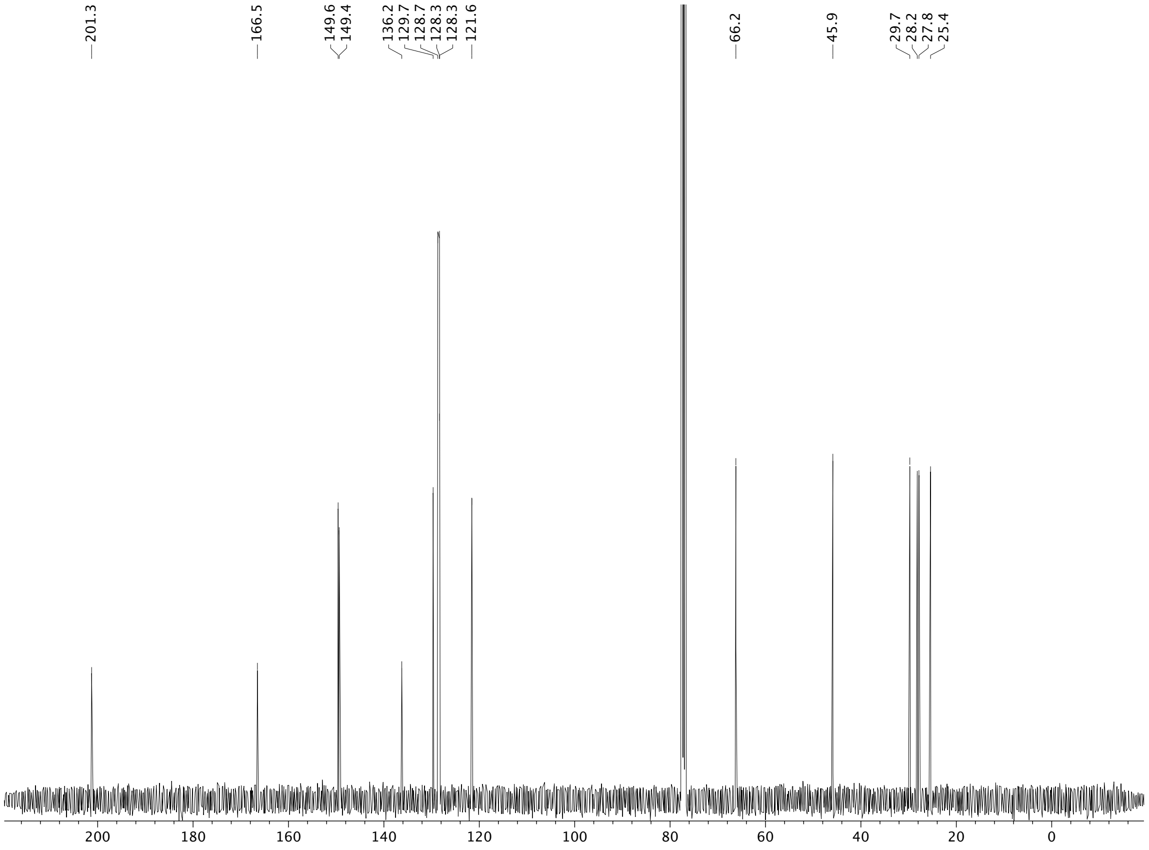

^{13}C NMR (100 MHz, $CDCl_3$) of compound **19e**.

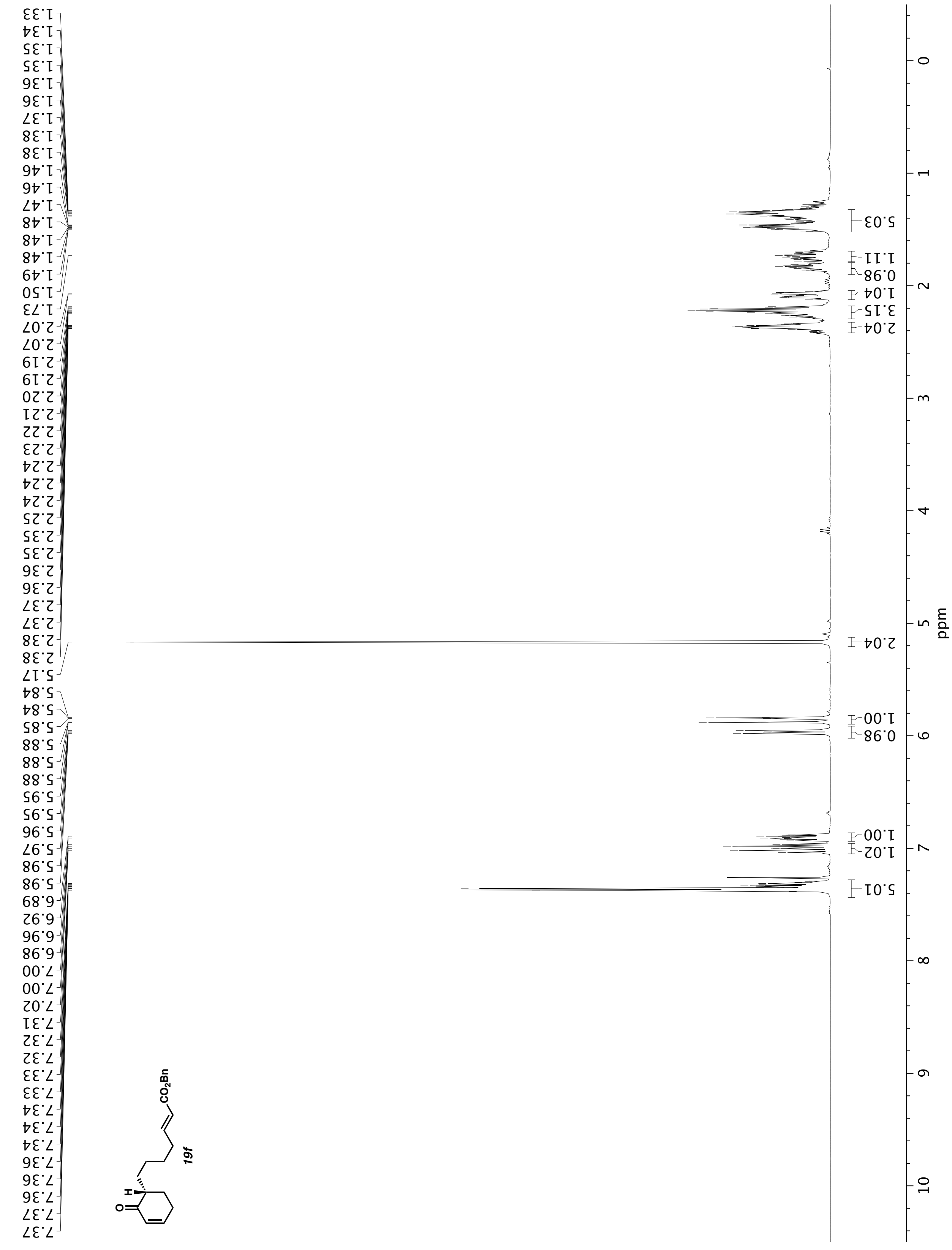

^{1}H NMR (400 MHz, $CDCl_3$) of compound **19f.**

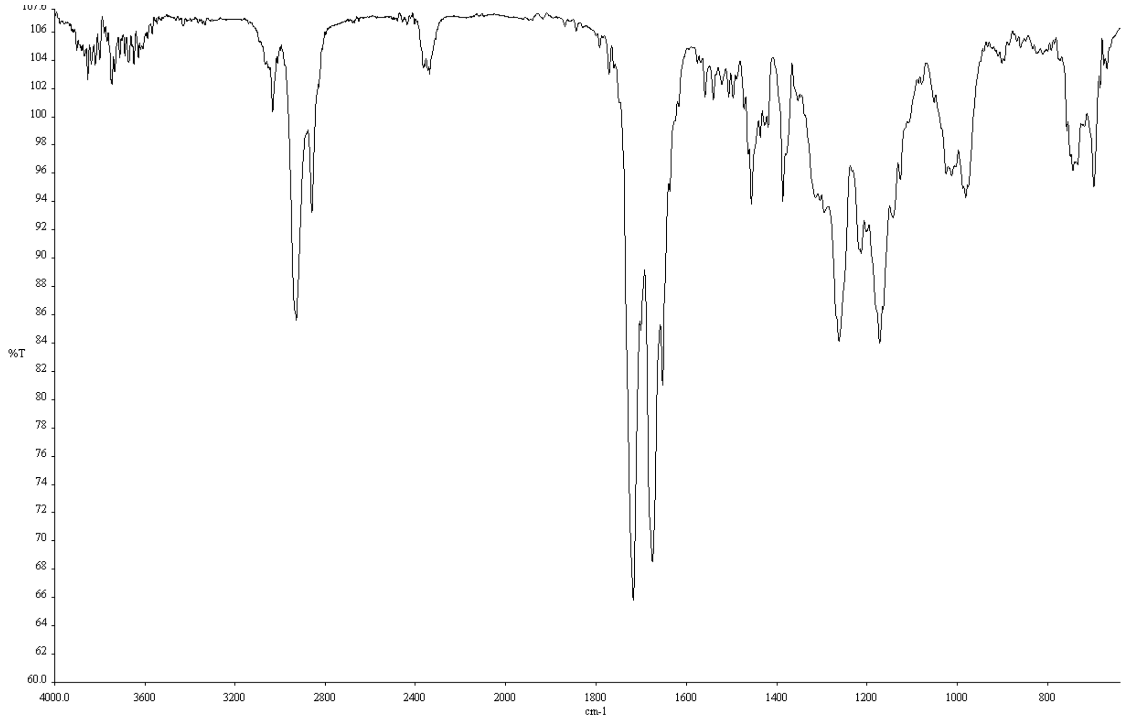

Infrared spectrum (Thin Film, NaCl) of compound **19f**.

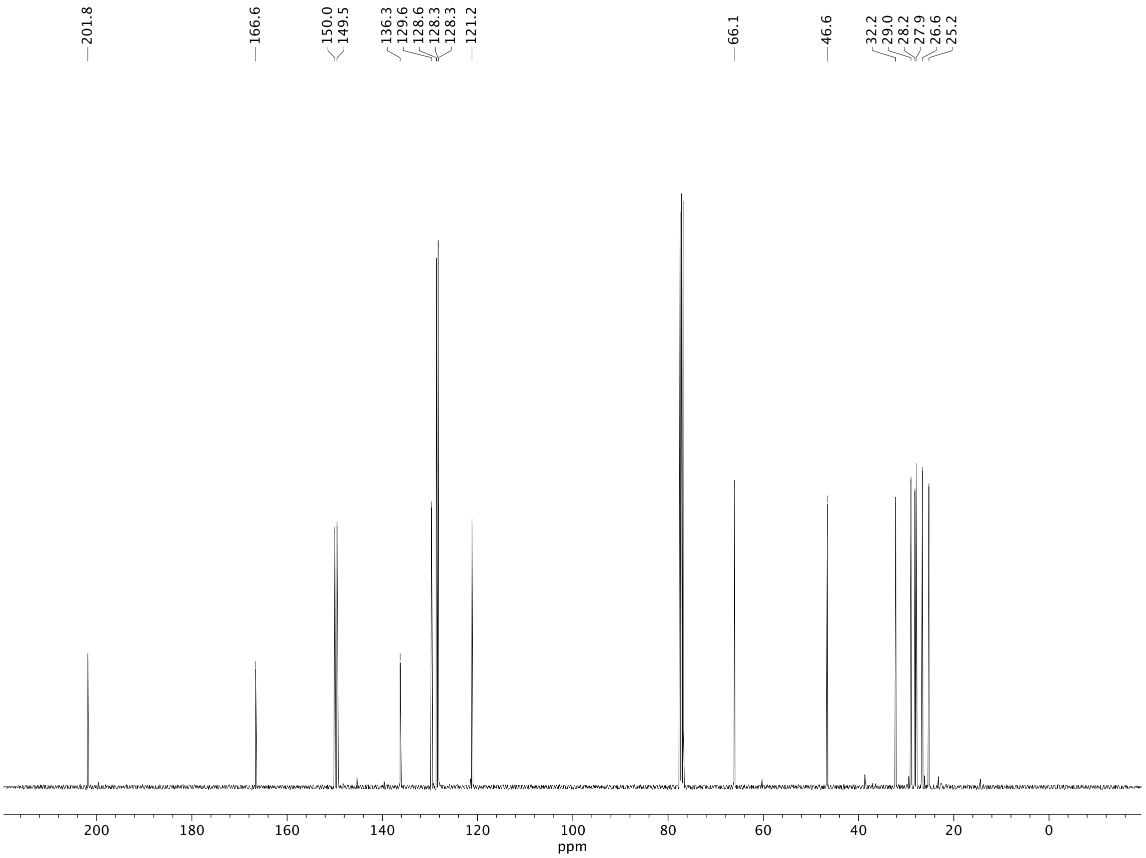

^{13}C NMR (100 MHz, $CDCl_3$) of compound **19f**.

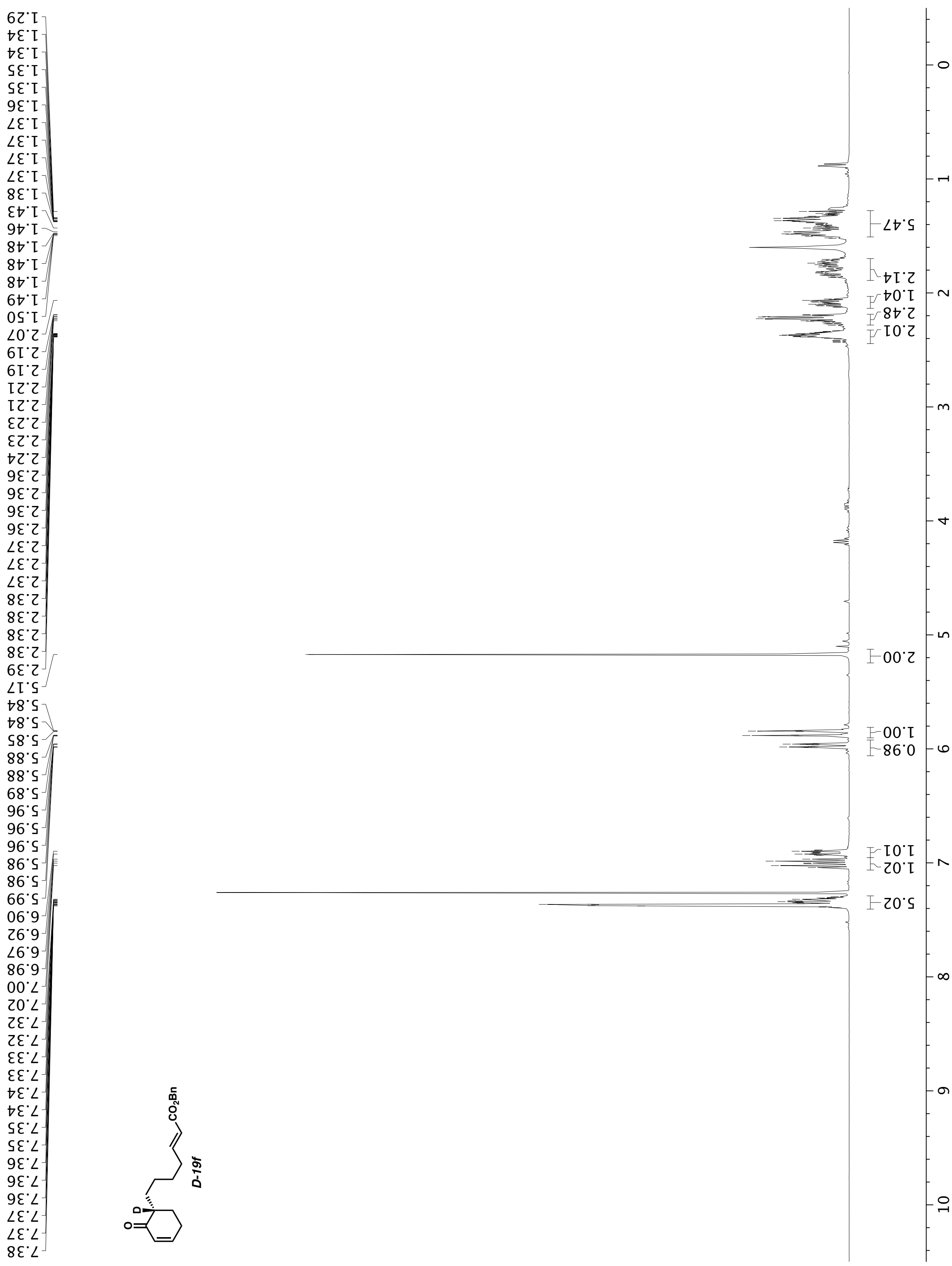

^{1}H NMR (400 MHz, $CDCl_3$) of compound ***D*-19f.**

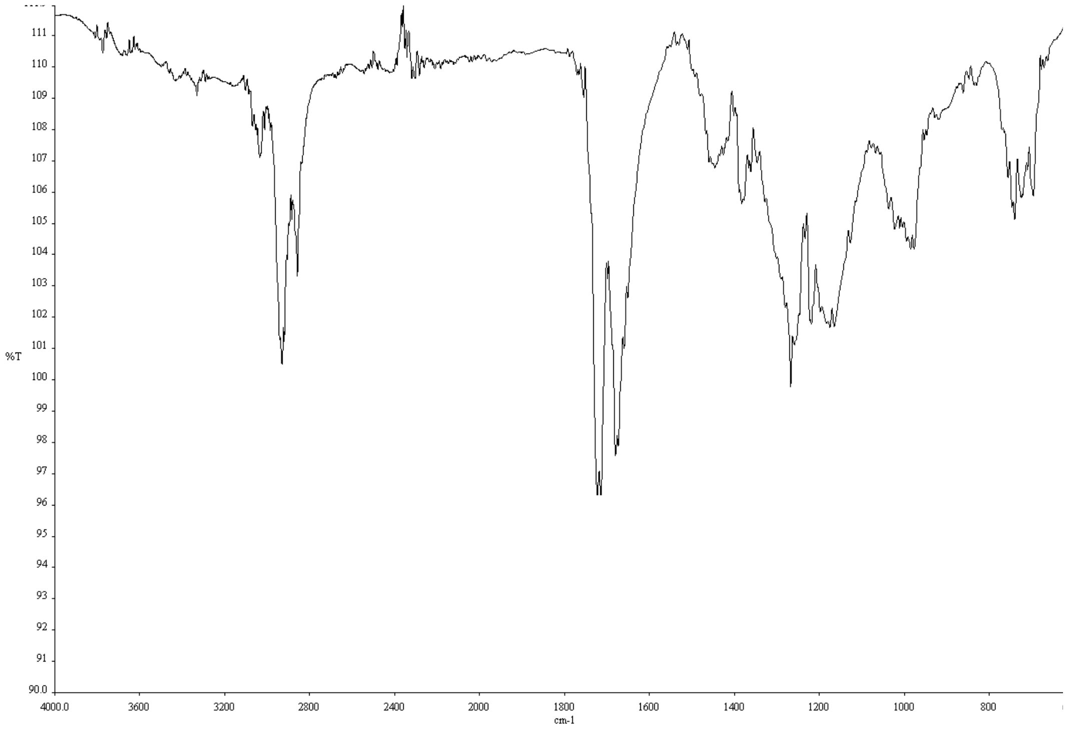

Infrared spectrum (Thin Film, NaCl) of compound ***D*-19f**.

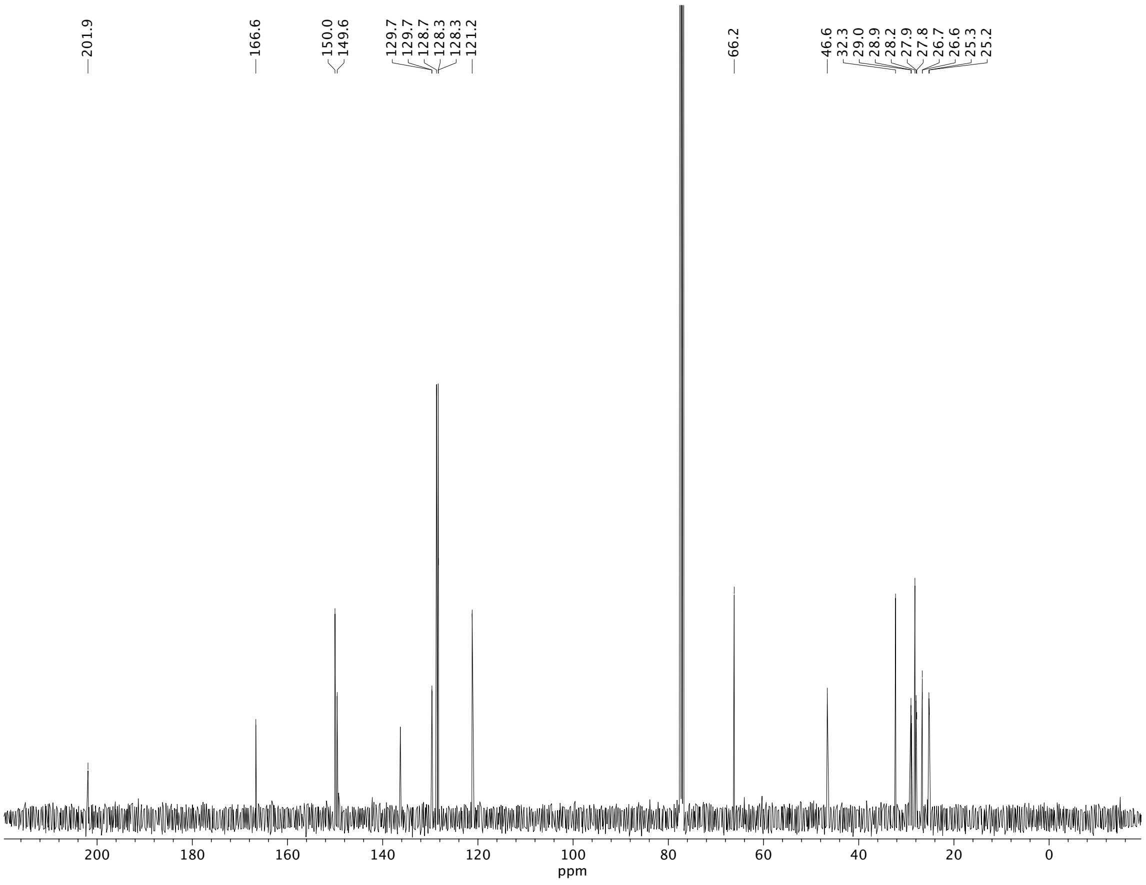

^{13}C NMR (100 MHz, $CDCl_3$) of compound ***D*-19f**.

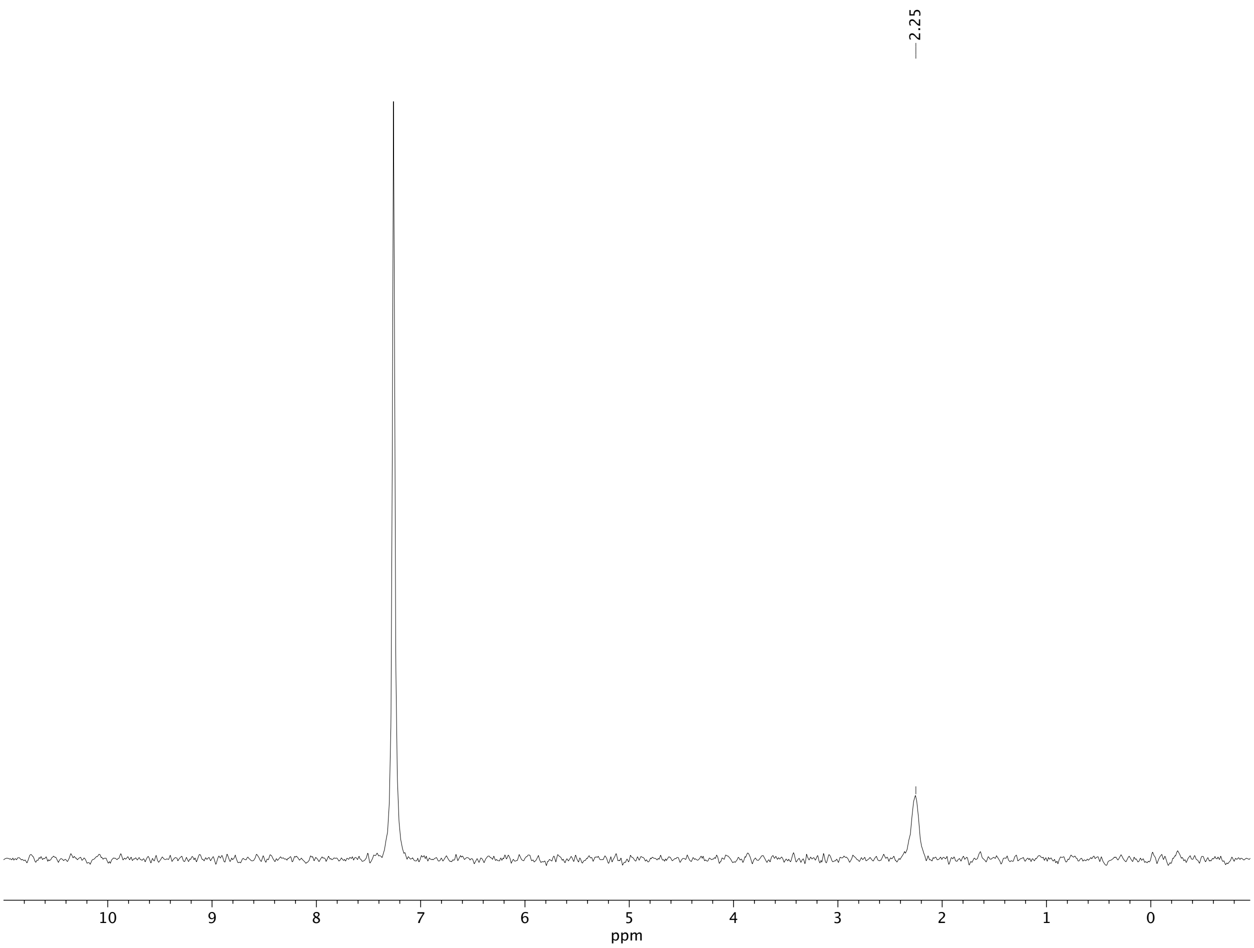

^{2}H NMR (61 MHz, $CHCl_3$) of compound ***D*-19f**.

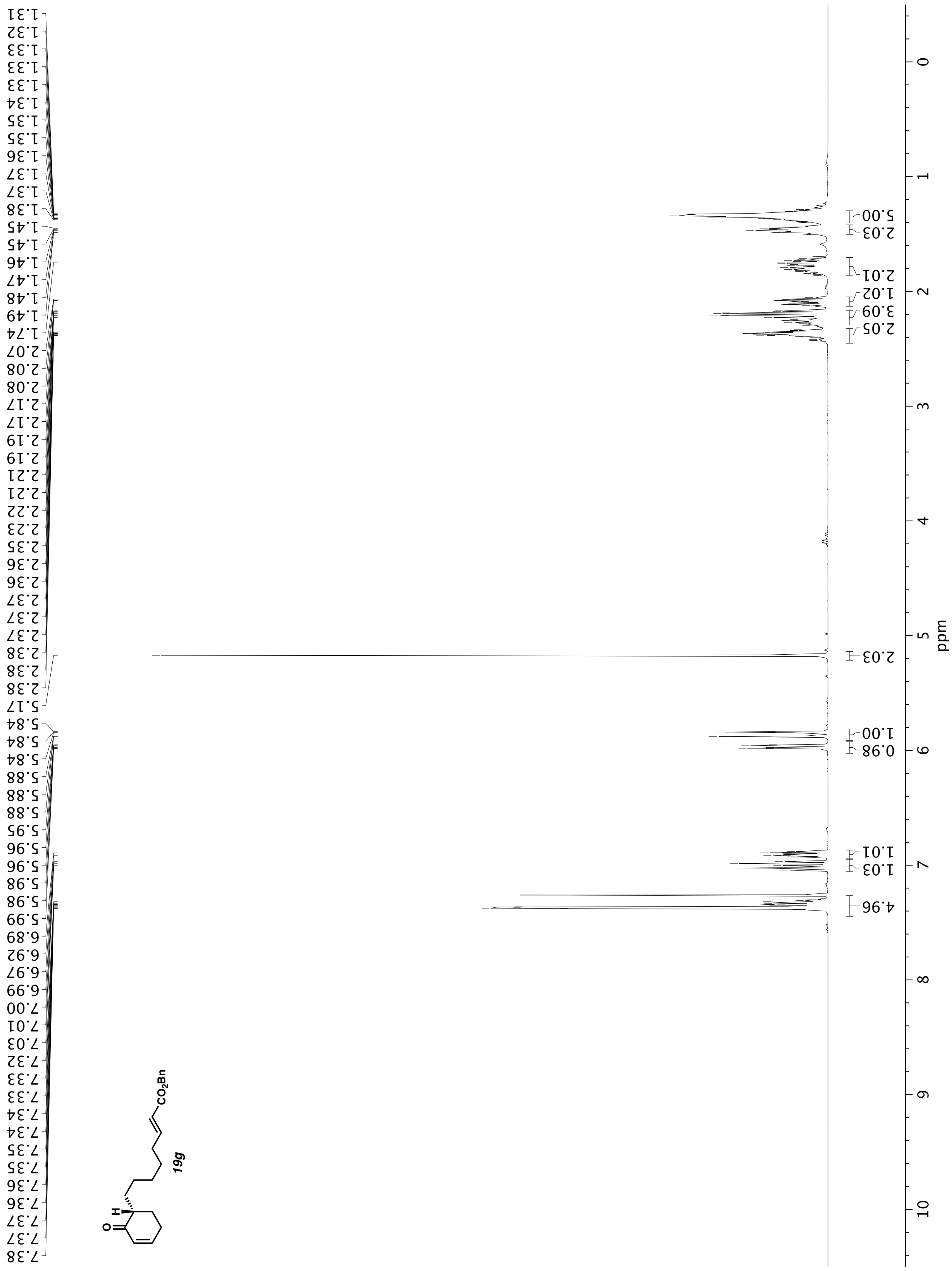

^{1}H NMR (400 MHz, $CDCl_3$) of compound **19g.**

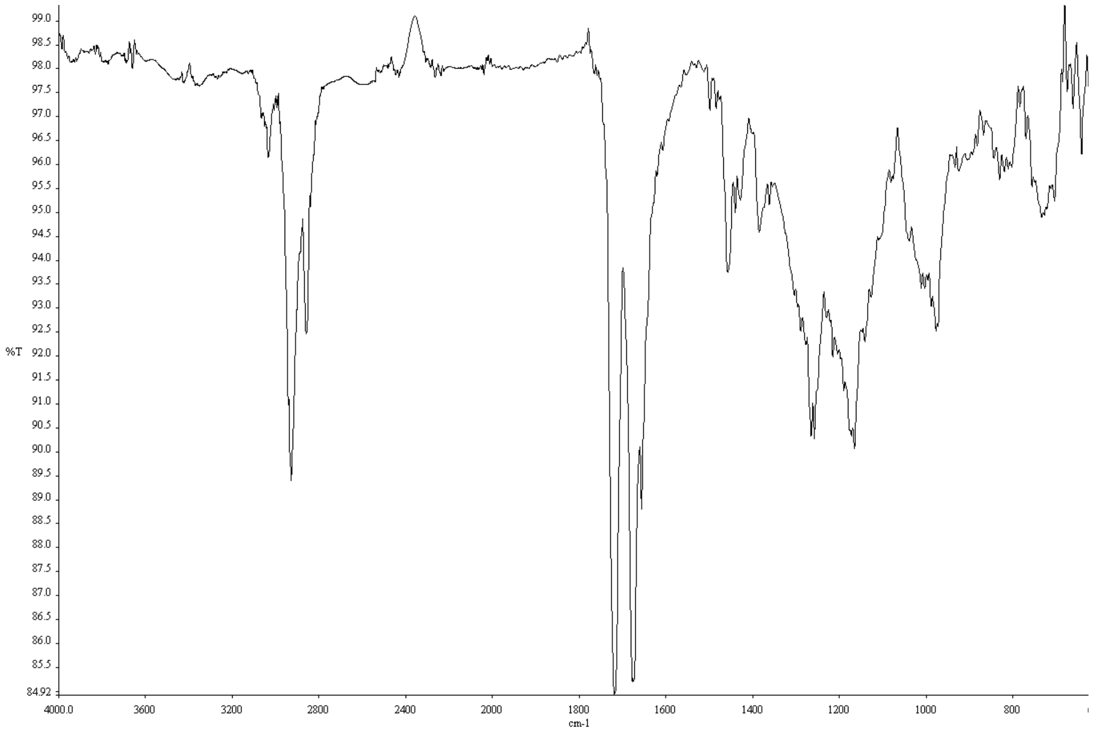

Infrared spectrum (Thin Film, NaCl) of compound **19g**.

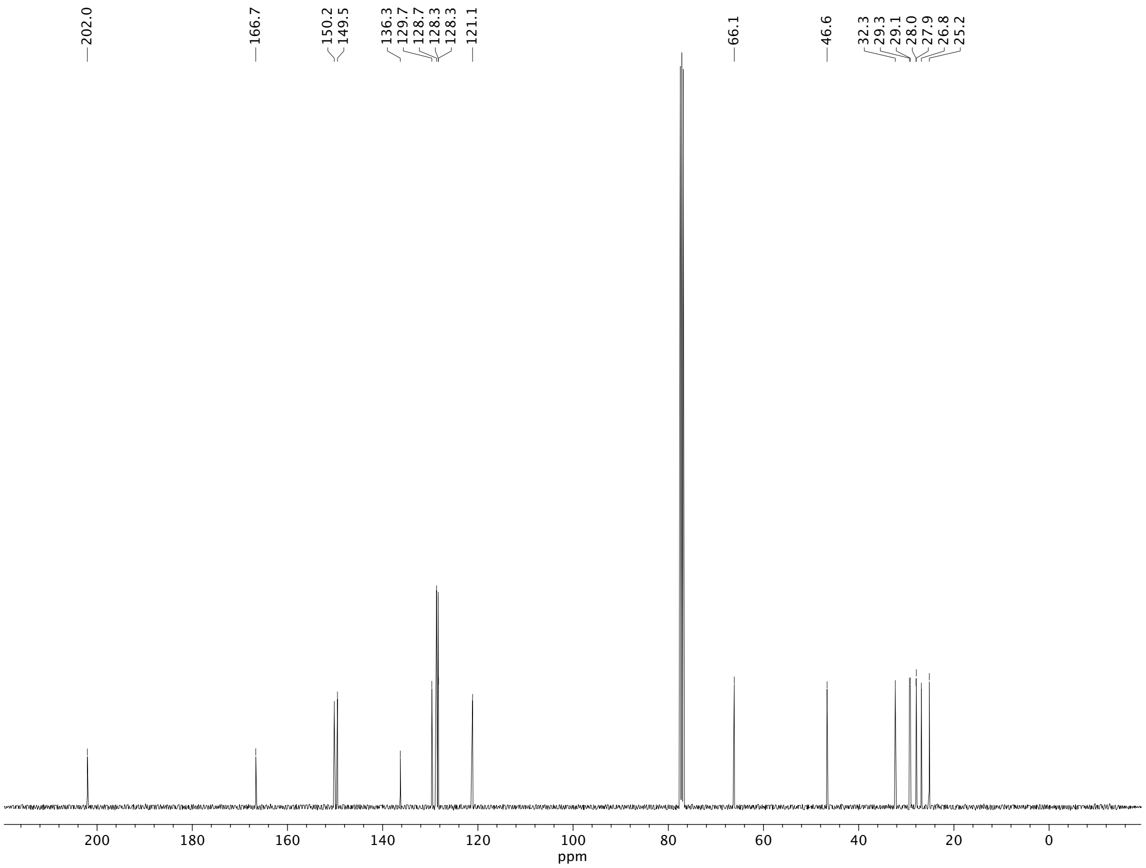

^{13}C NMR (100 MHz, $CDCl_3$) of compound **19g**.

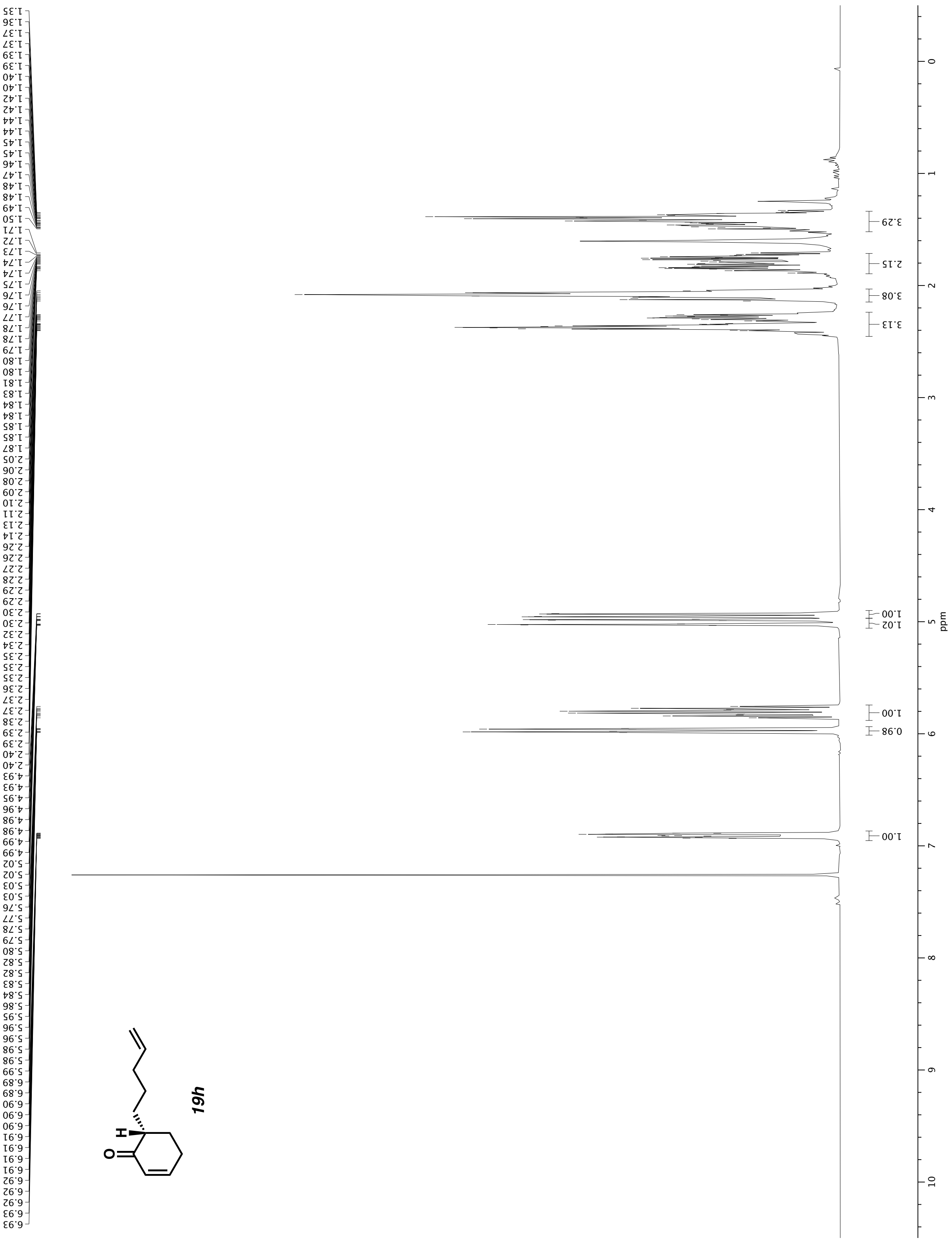

^{1}H NMR (400 MHz, $CDCl_3$) of compound **19h**.

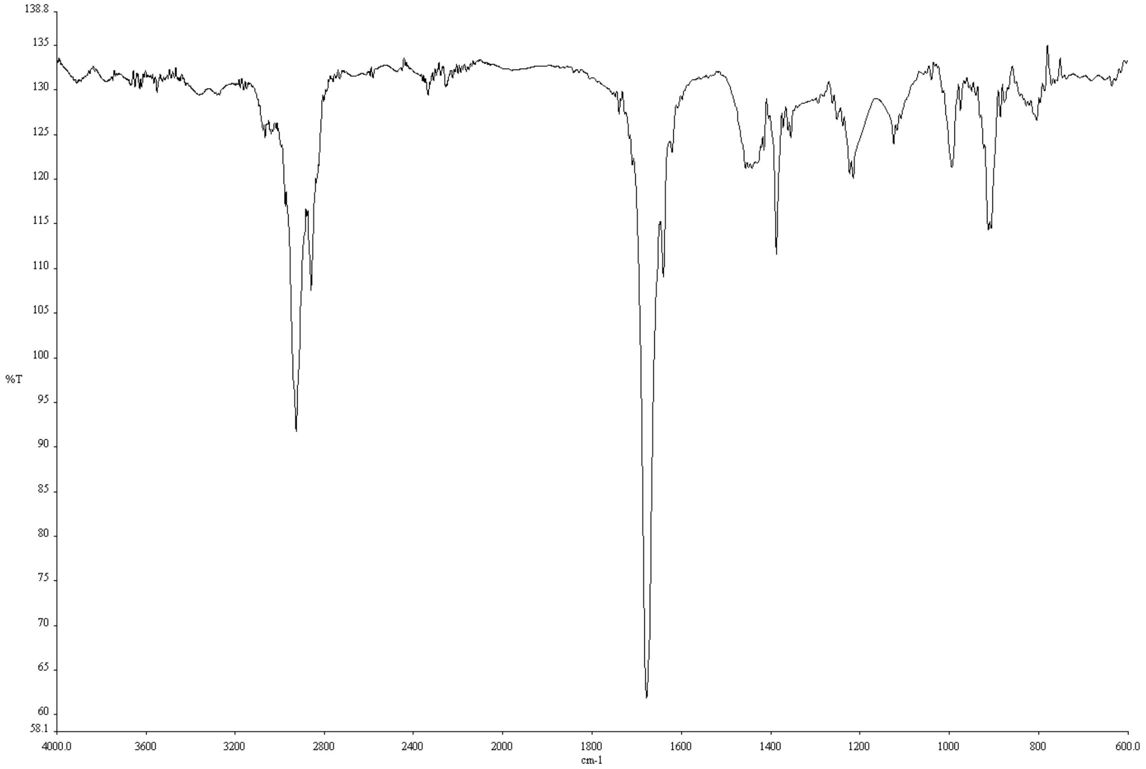

Infrared spectrum (Thin Film, NaCl) of compound **19h**.

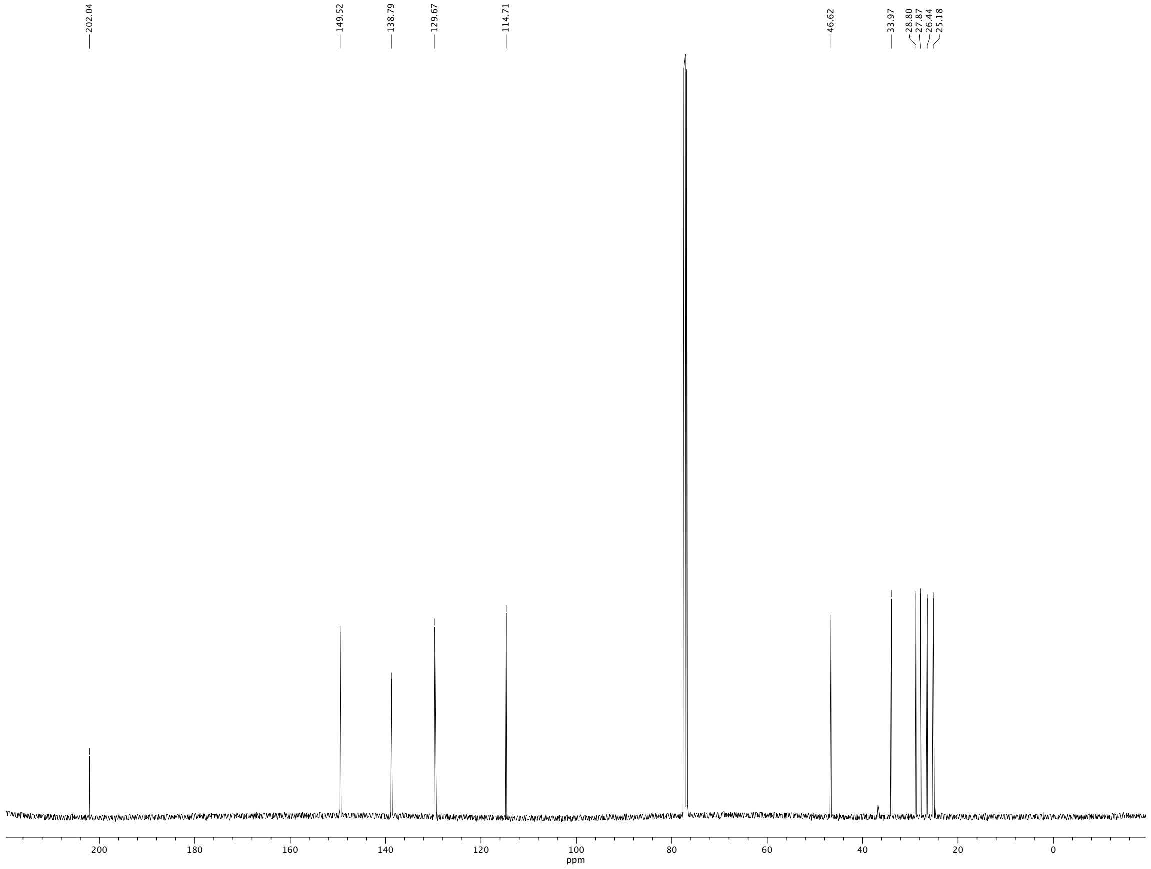

^{13}C NMR (100 MHz, $CDCl_3$) of compound **19h**.

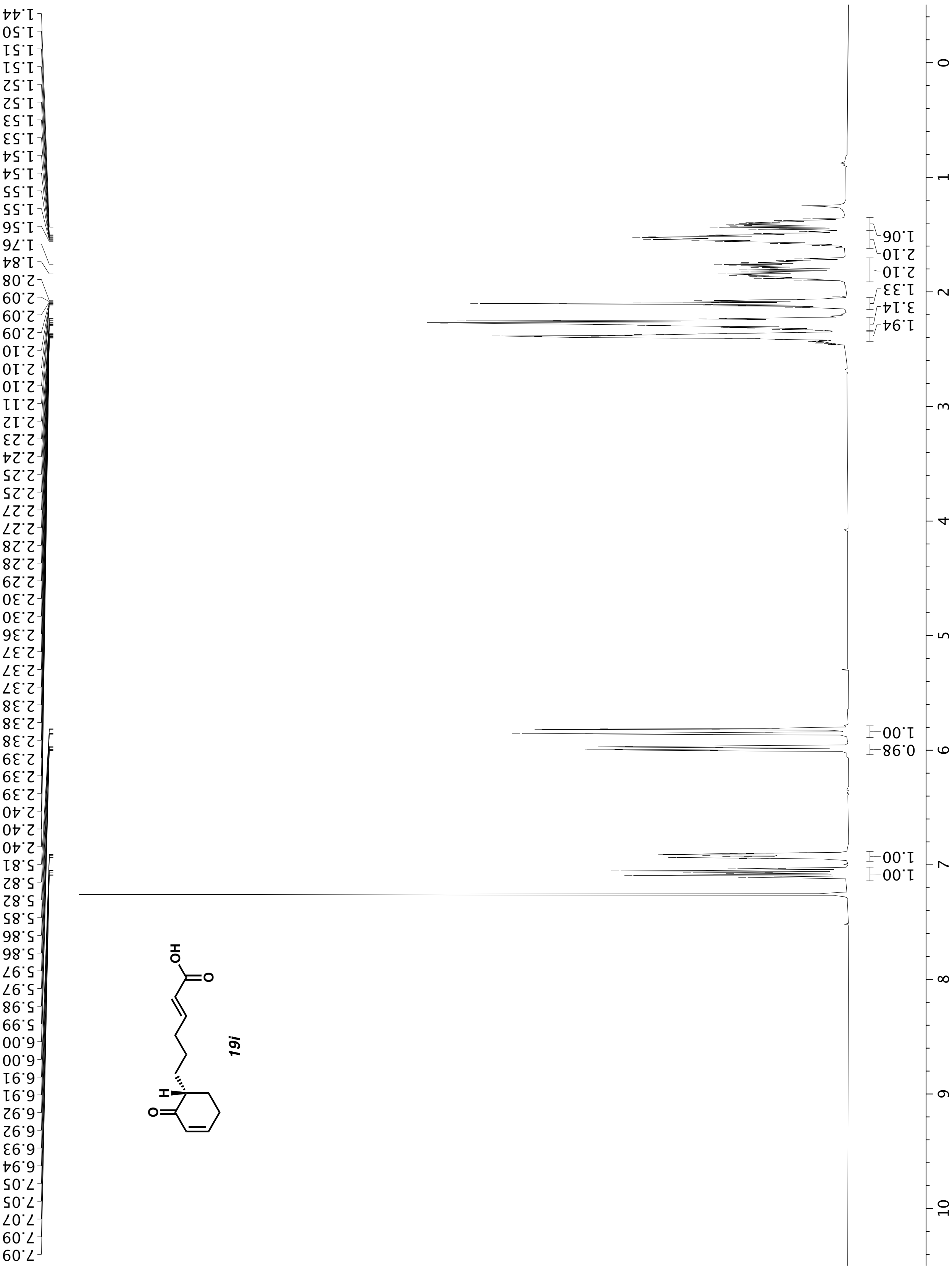

^{1}H NMR (400 MHz, $CDCl_3$) of compound **19i**.

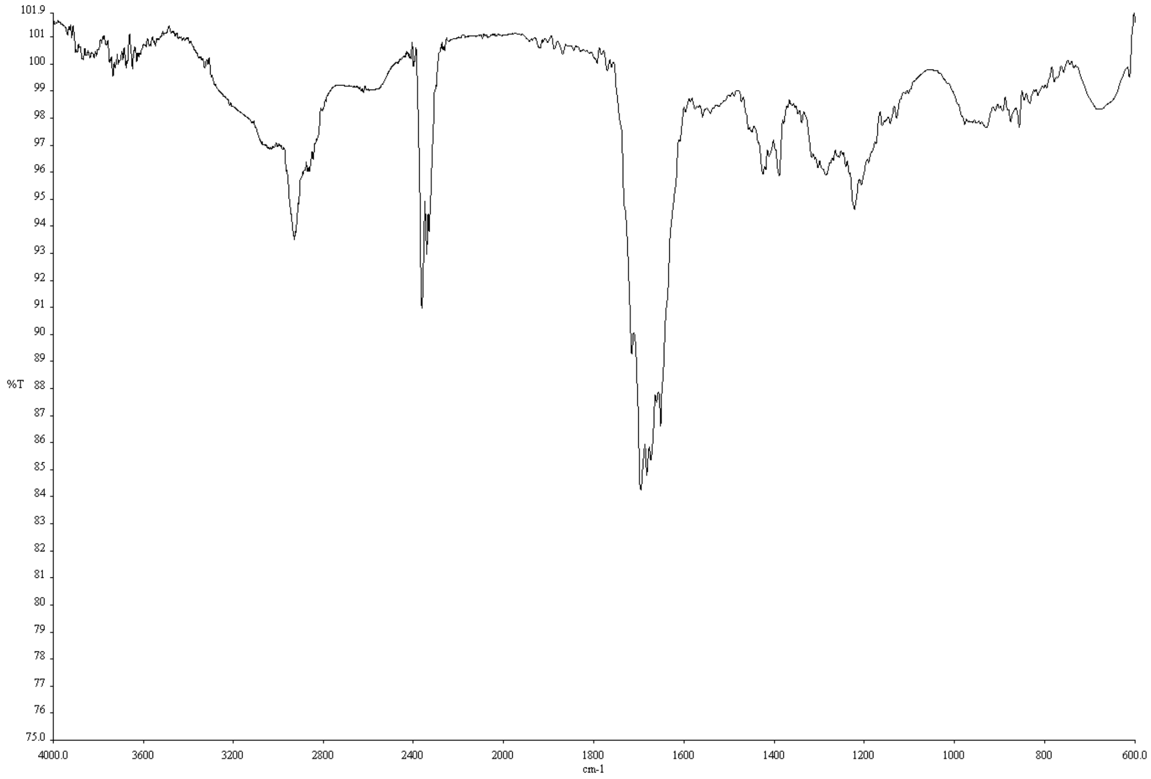

Infrared spectrum (Thin Film, NaCl) of compound **19i**.

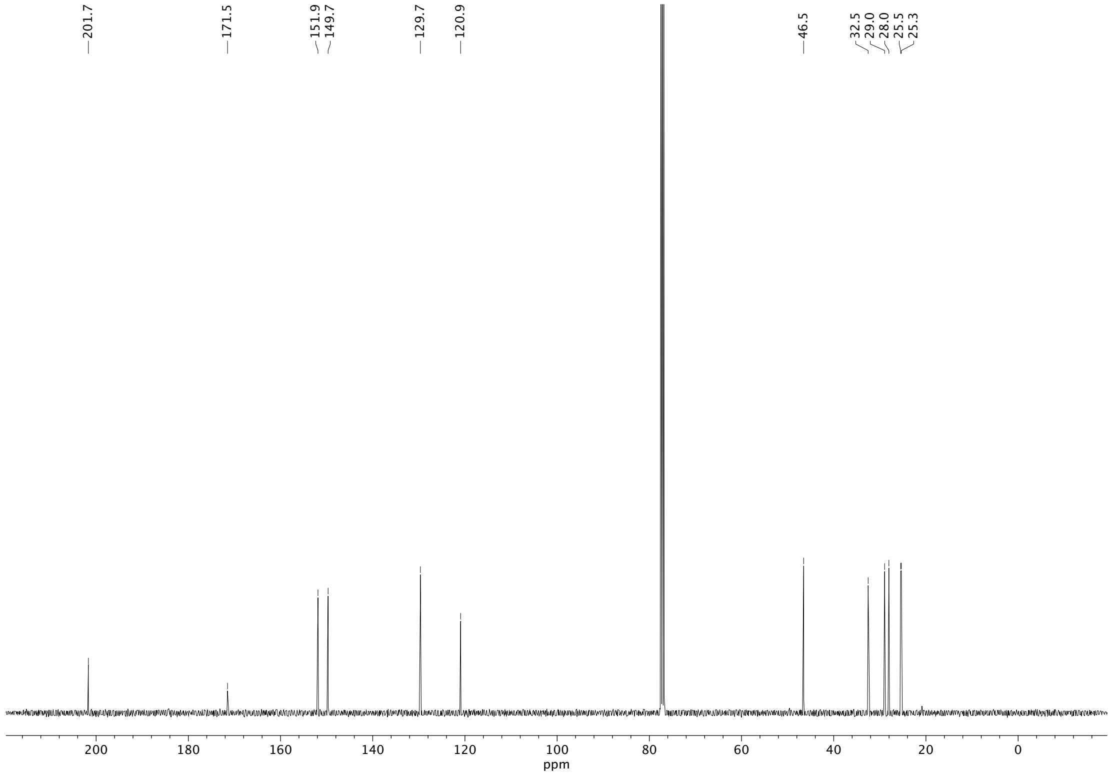

^{13}C NMR (100 MHz, $CDCl_3$) of compound **19i**.

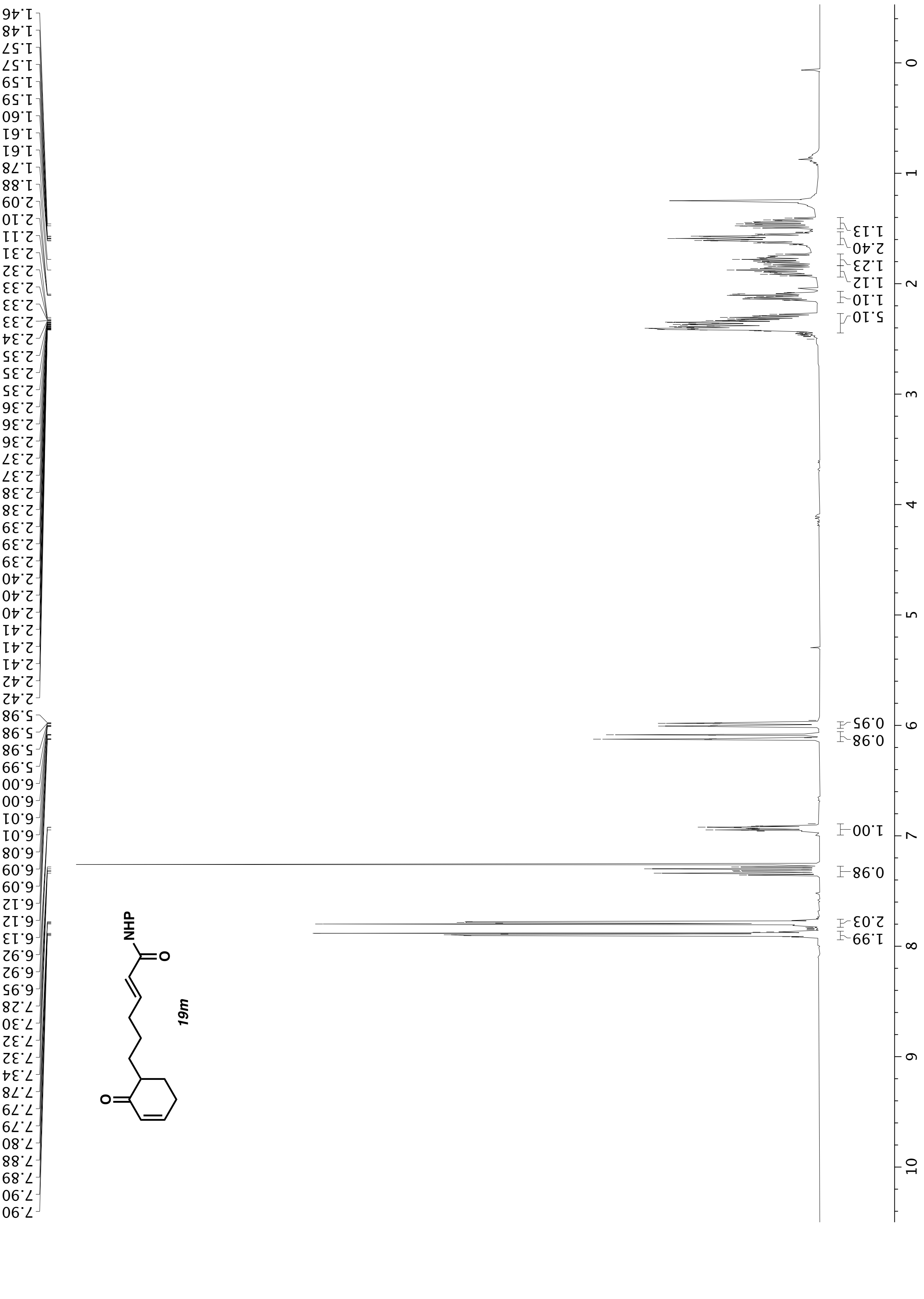

^{1}H NMR (400 MHz, $CDCl_3$) of compound **19m**.

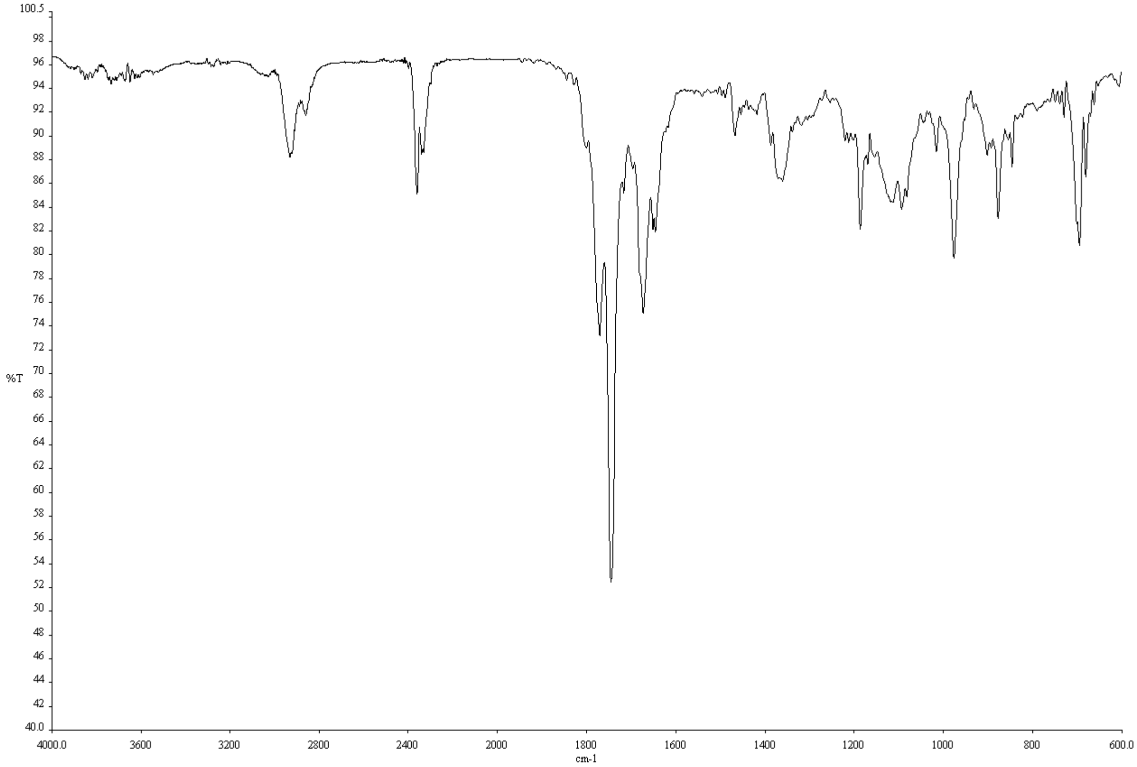

Infrared spectrum (Thin Film, NaCl) of compound **19m**.

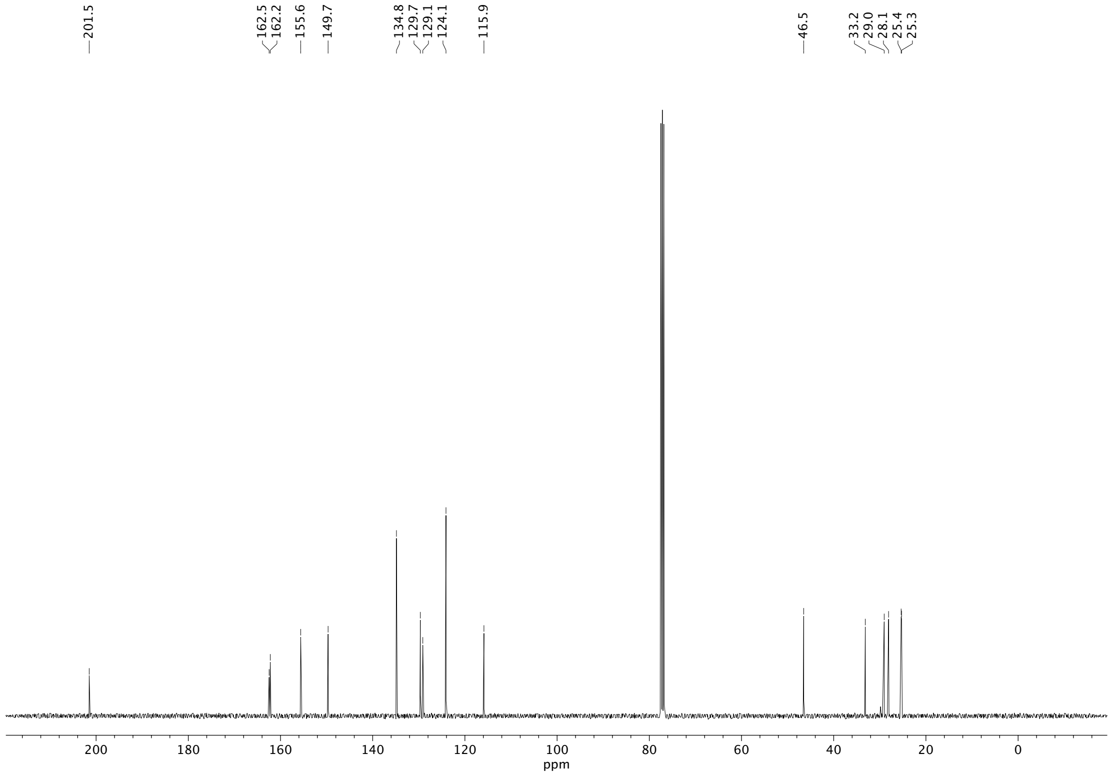

^{13}C NMR (100 MHz, $CDCl_3$) of compound **19m**.

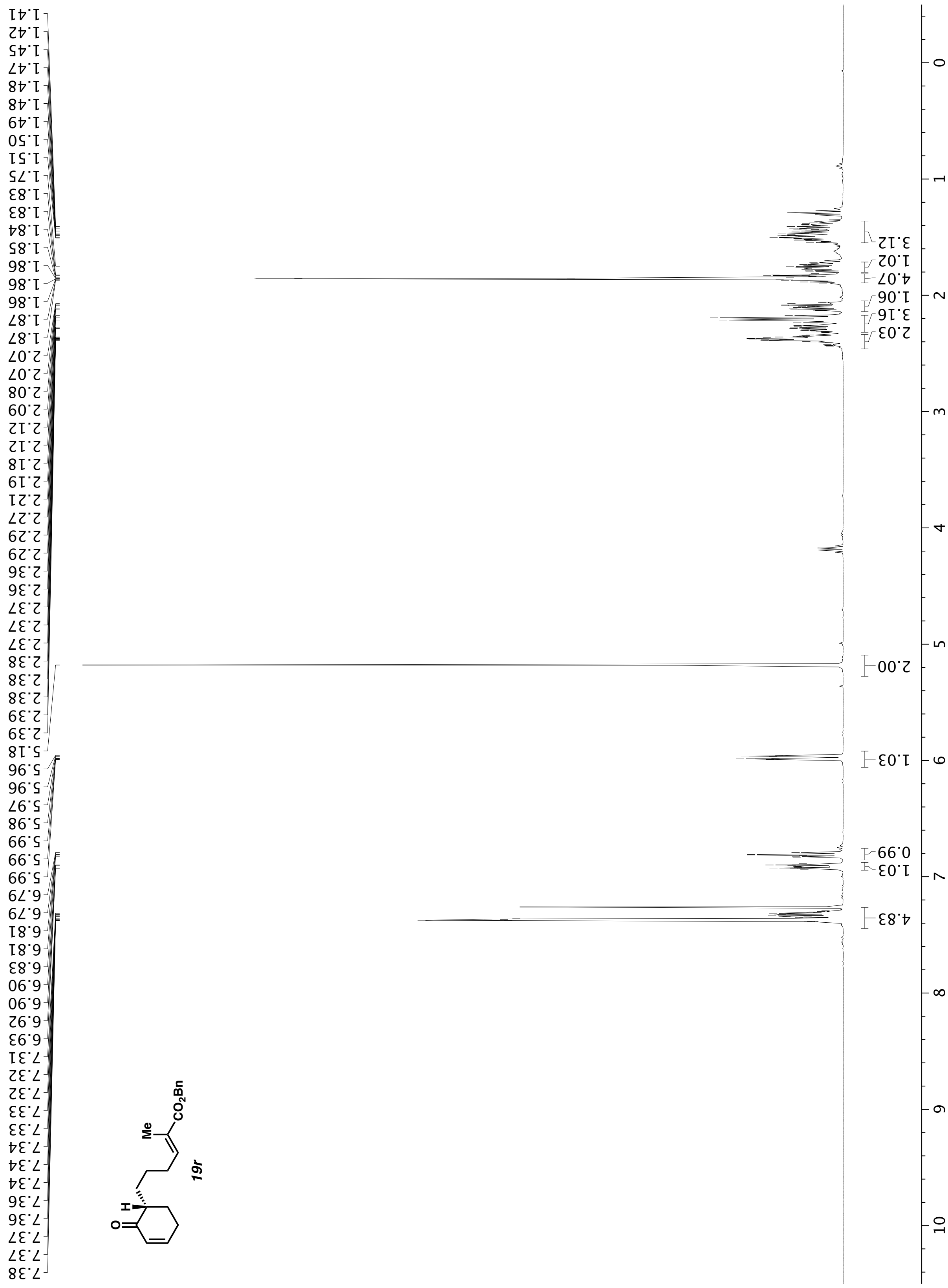

^{1}H NMR (400 MHz, $CDCl_3$) of compound **19r**.

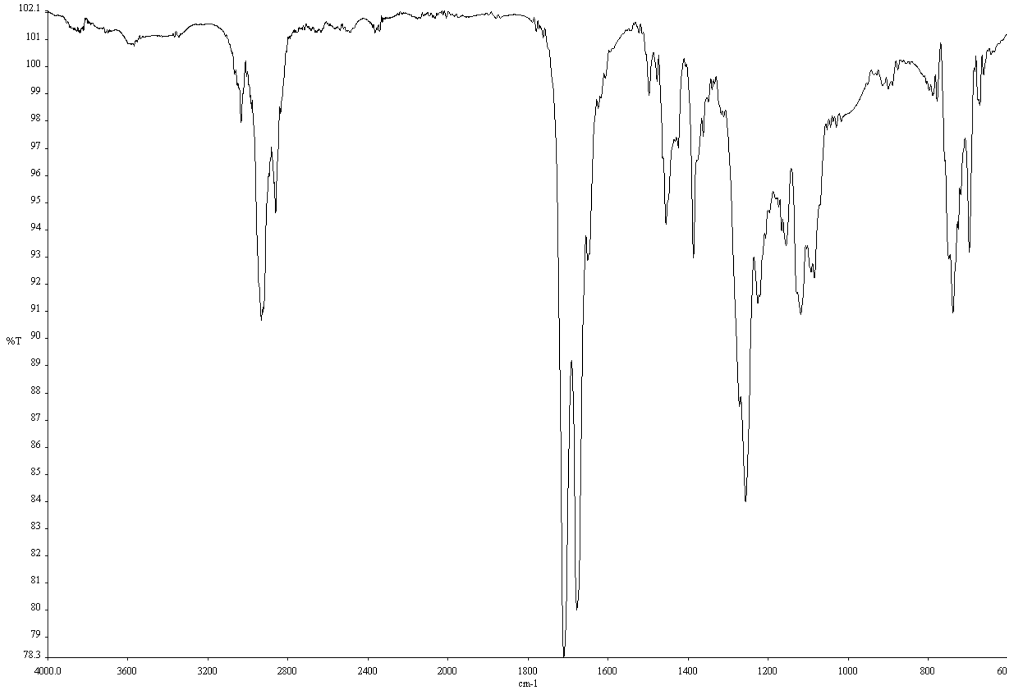

Infrared spectrum (Thin Film, NaCl) of compound **19r**.

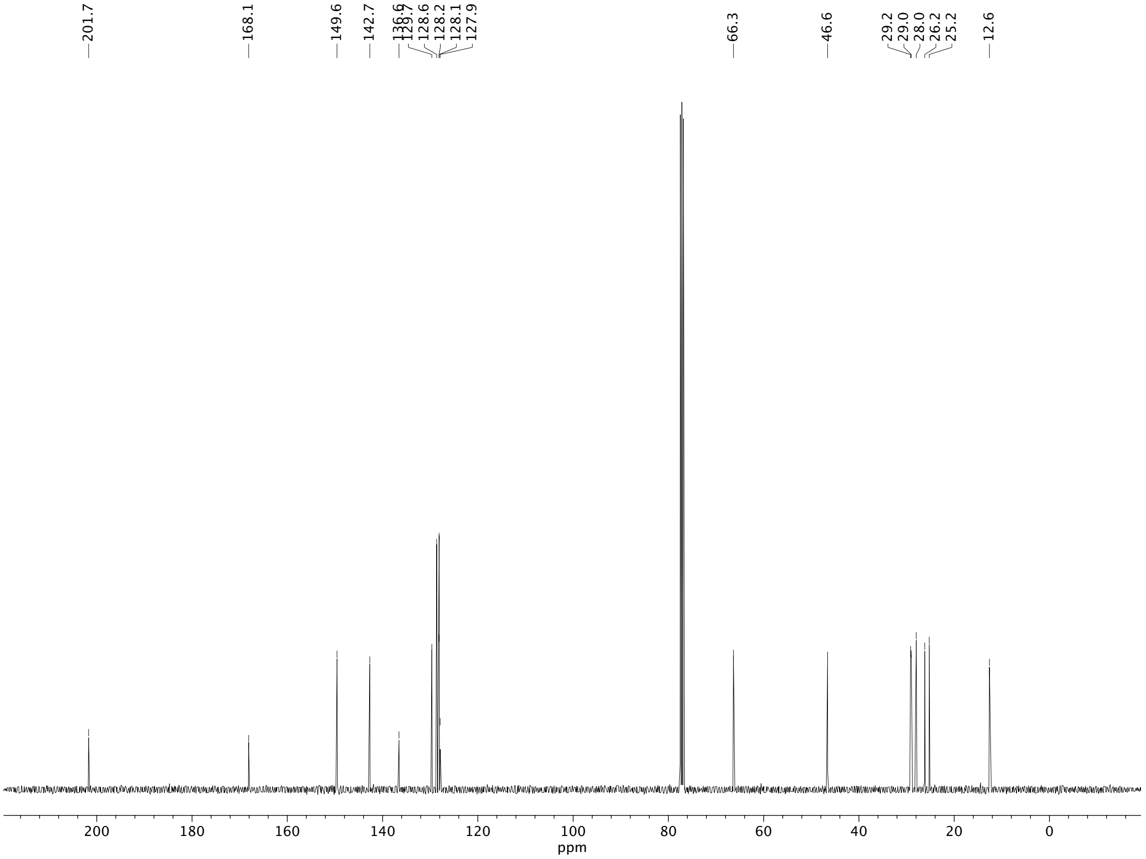

^{13}C NMR (100 MHz, $CDCl_3$) of compound **19r**.

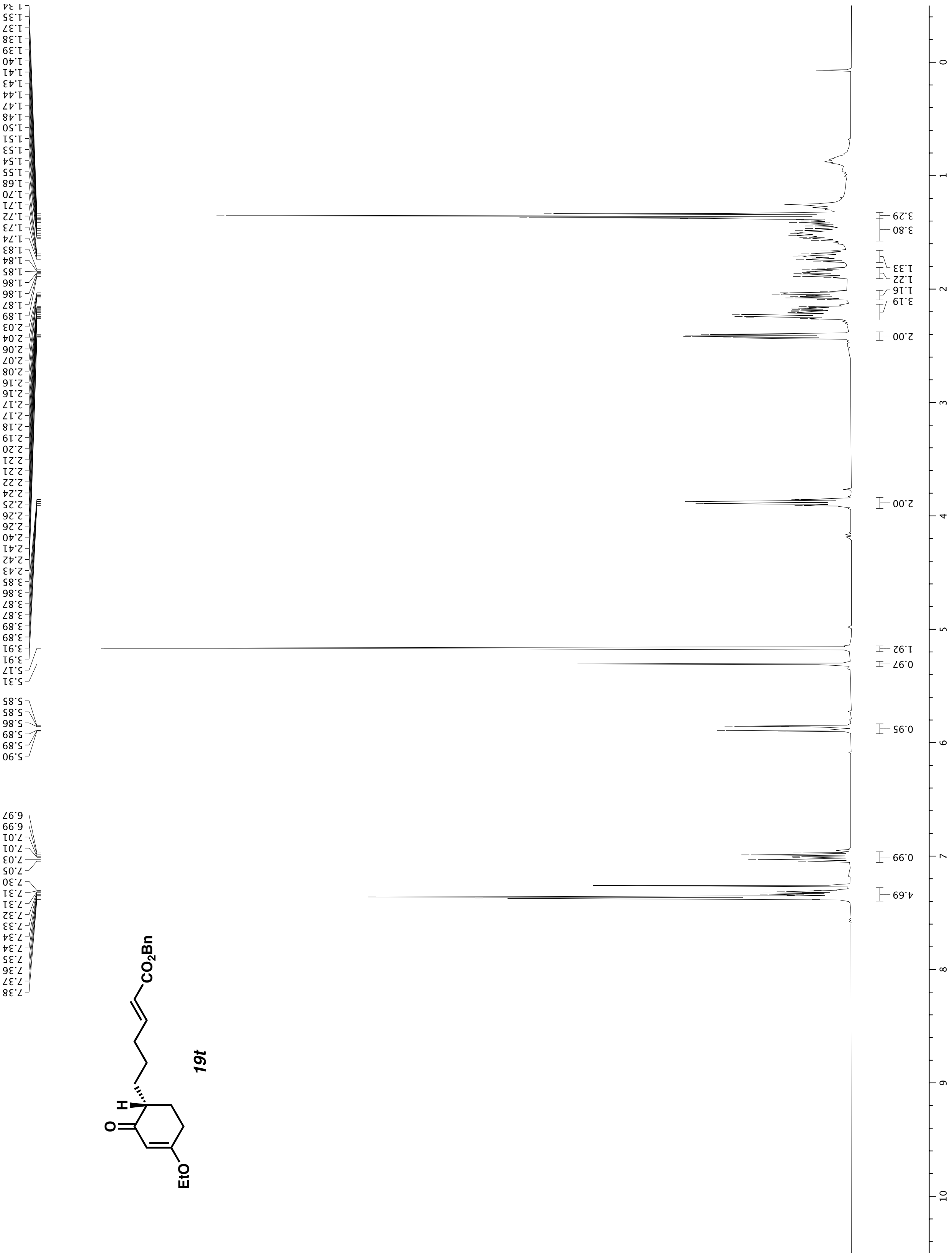

^{1}H NMR (400 MHz, $CDCl_3$) of compound **19t**.

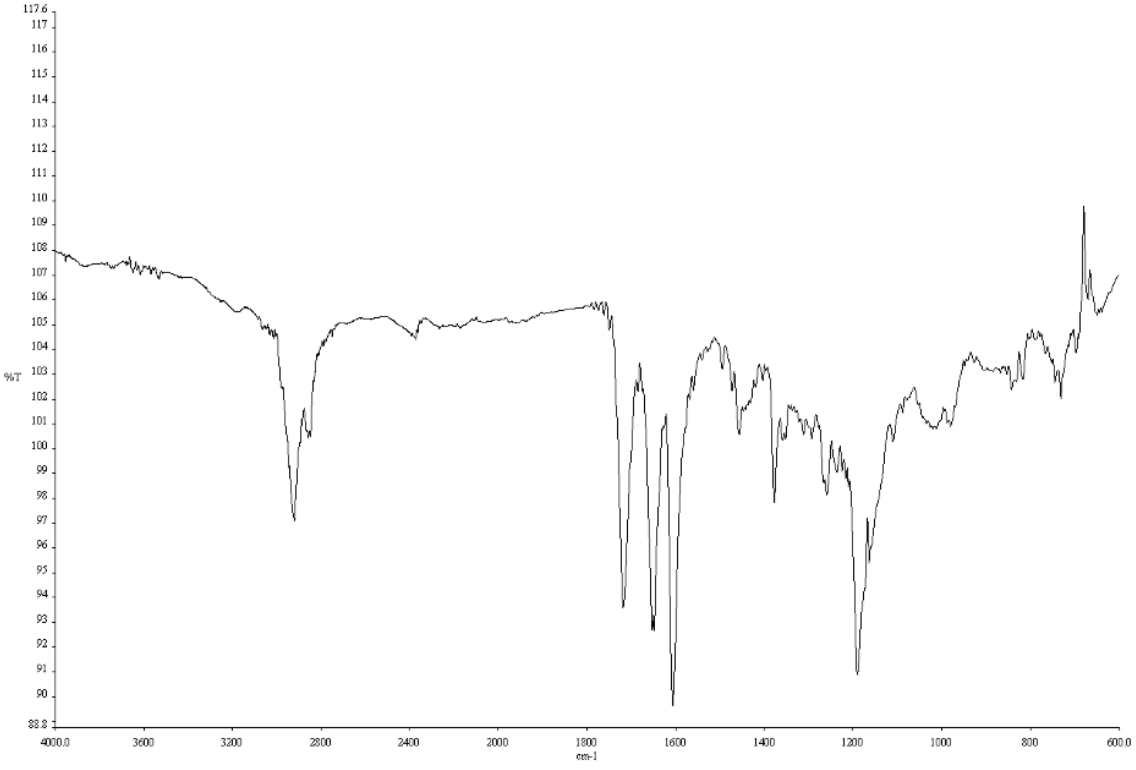

Infrared spectrum (Thin Film, NaCl) of compound **19t**.

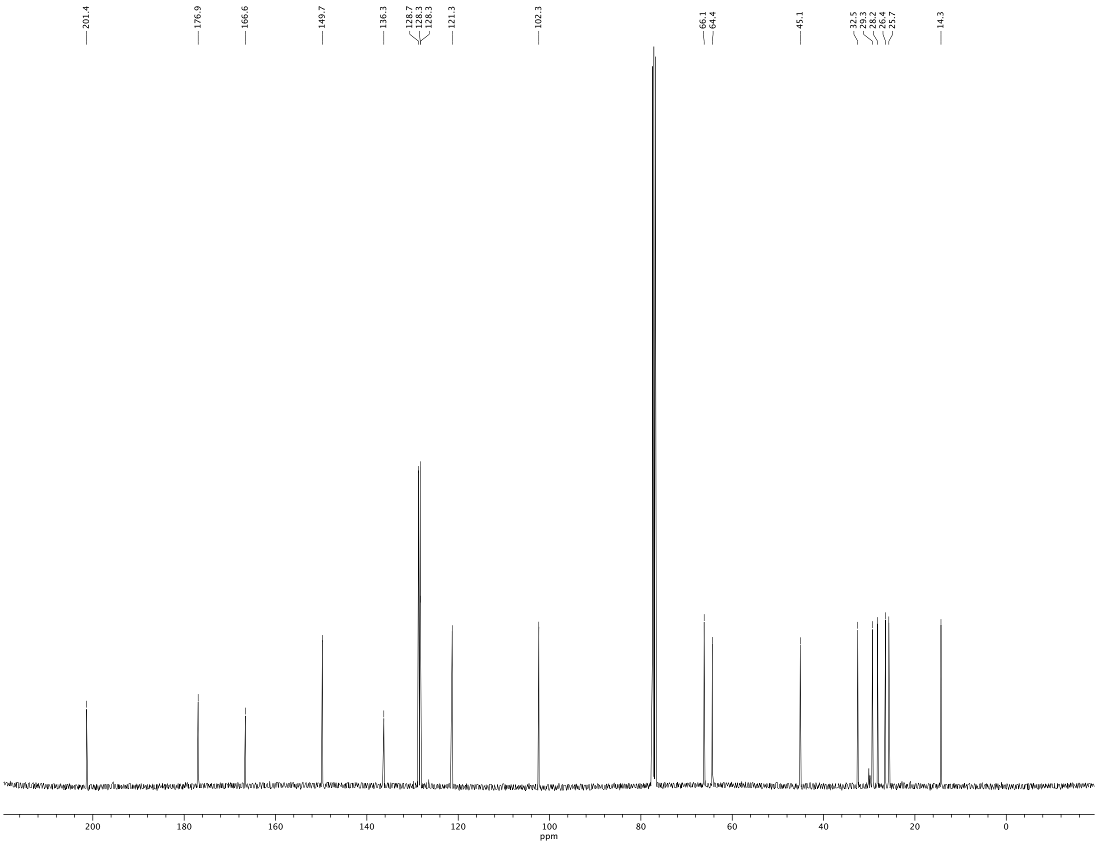

^{13}C NMR (100 MHz, $CDCl_3$) of compound **19t**.

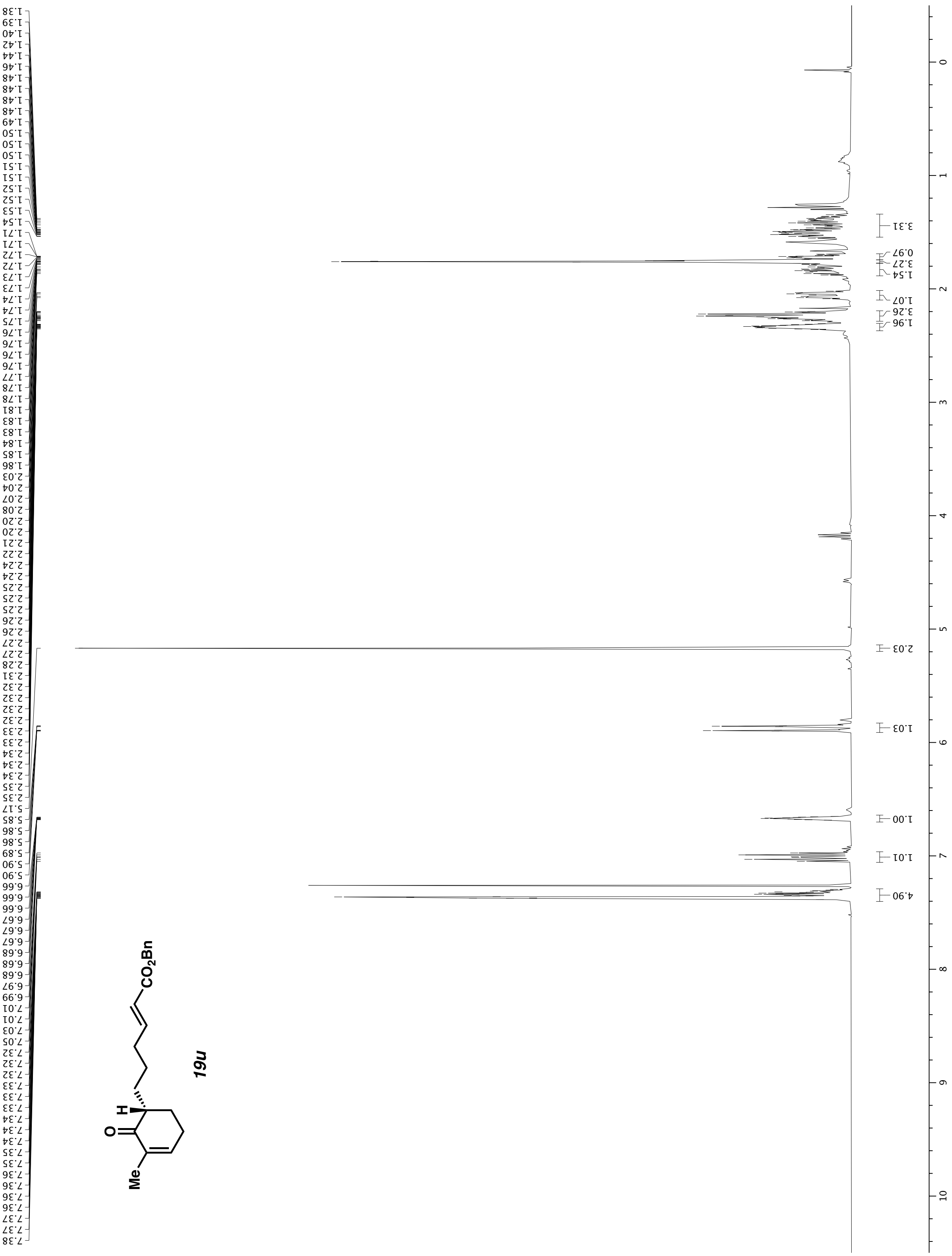

^{1}H NMR (400 MHz, $CDCl_3$) of compound **19u**.

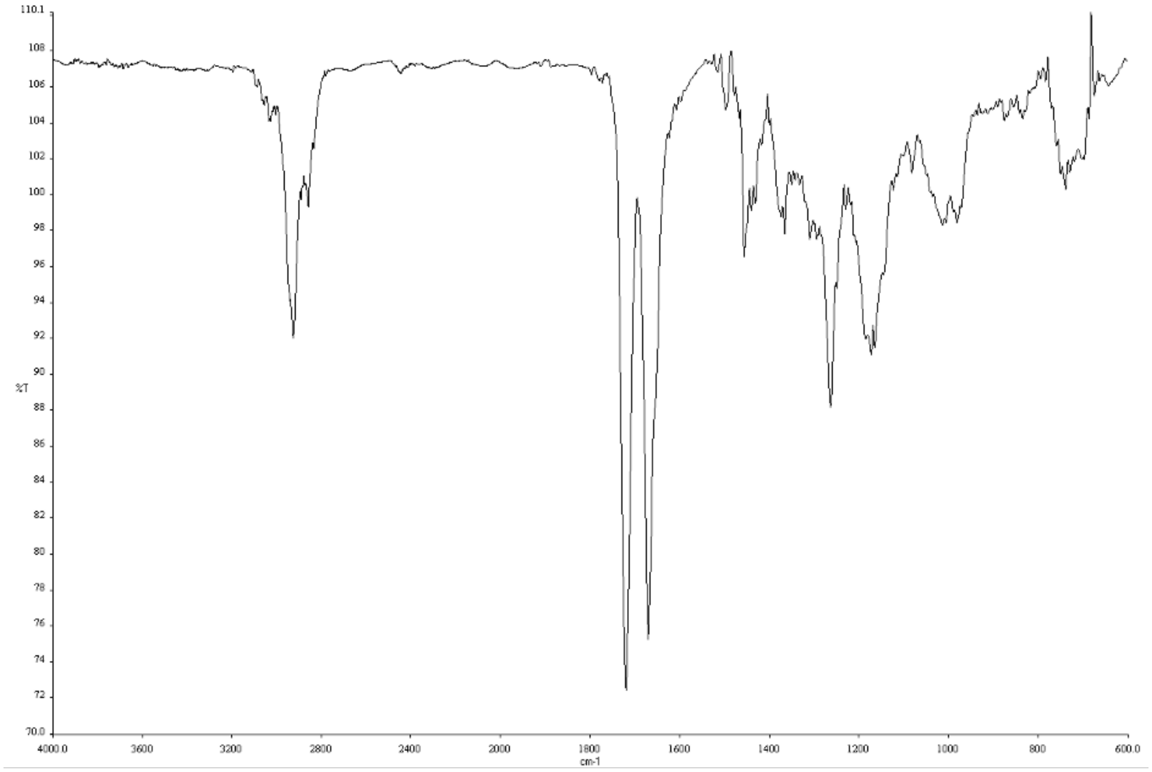

Infrared spectrum (Thin Film, NaCl) of compound **19u**.

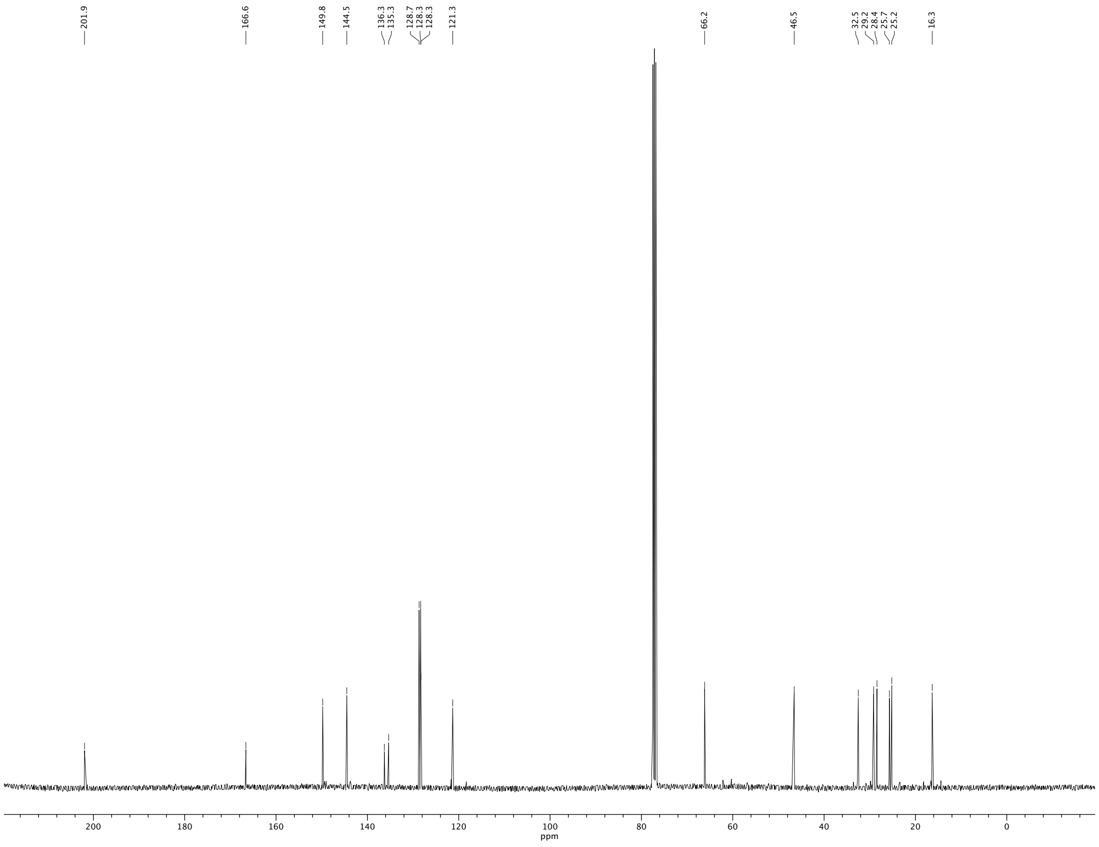

^{13}C NMR (100 MHz, $CDCl_3$) of compound **19u**.

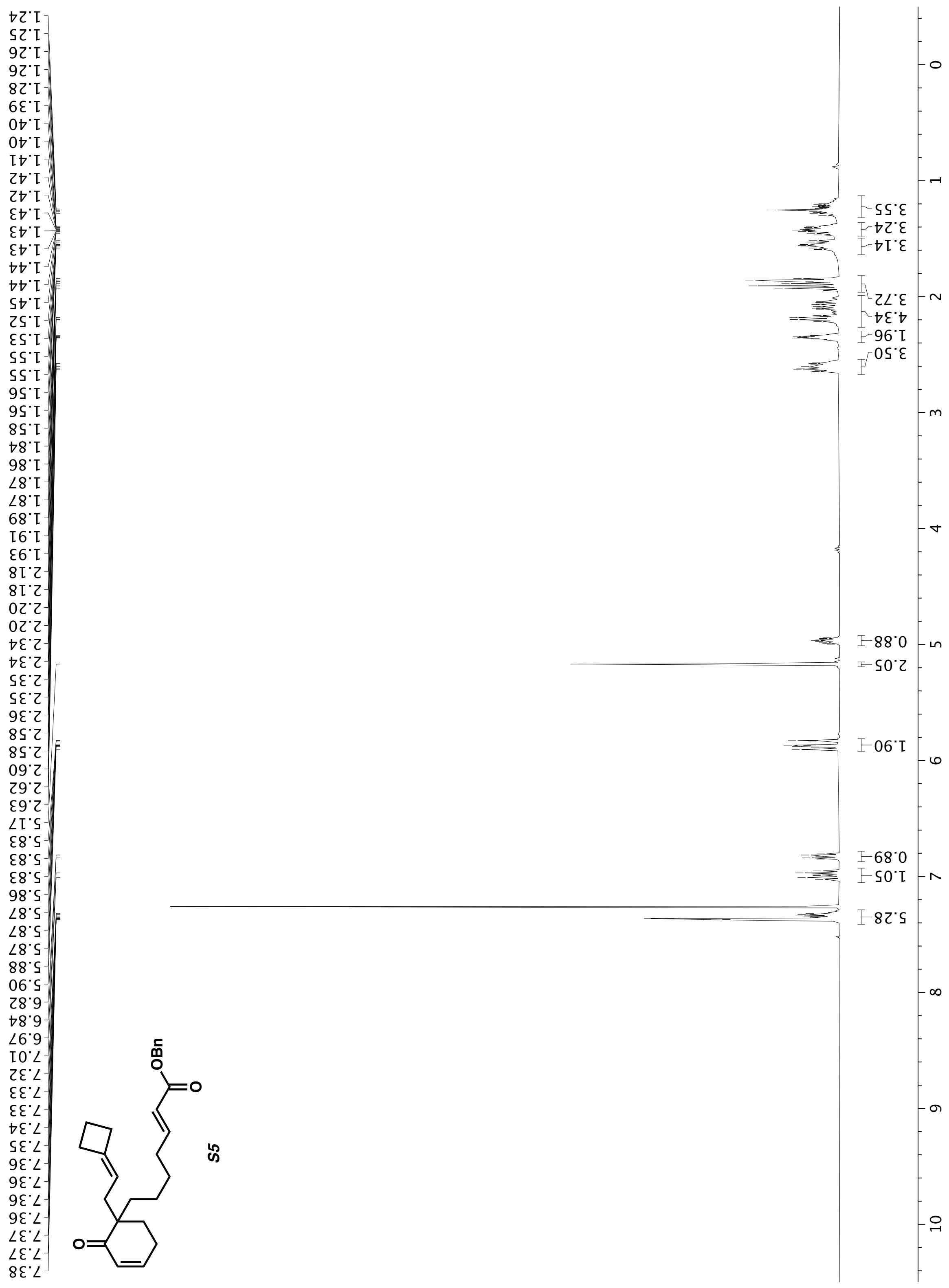

^{1}H NMR (400 MHz, $CDCl_3$) of compound **S5**.

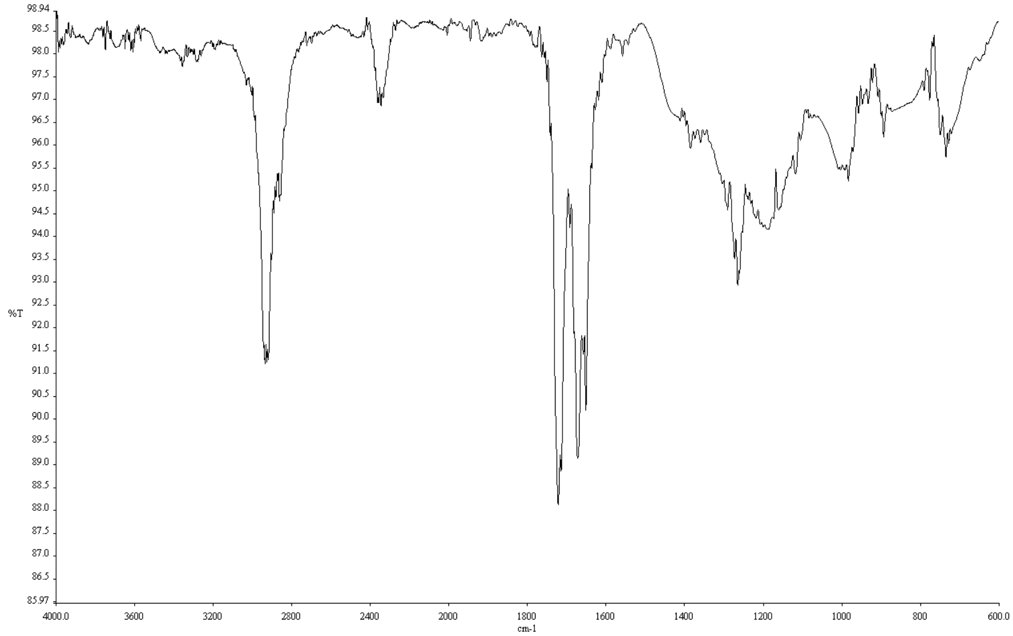

Infrared spectrum (Thin Film, NaCl) of compound **S5**.

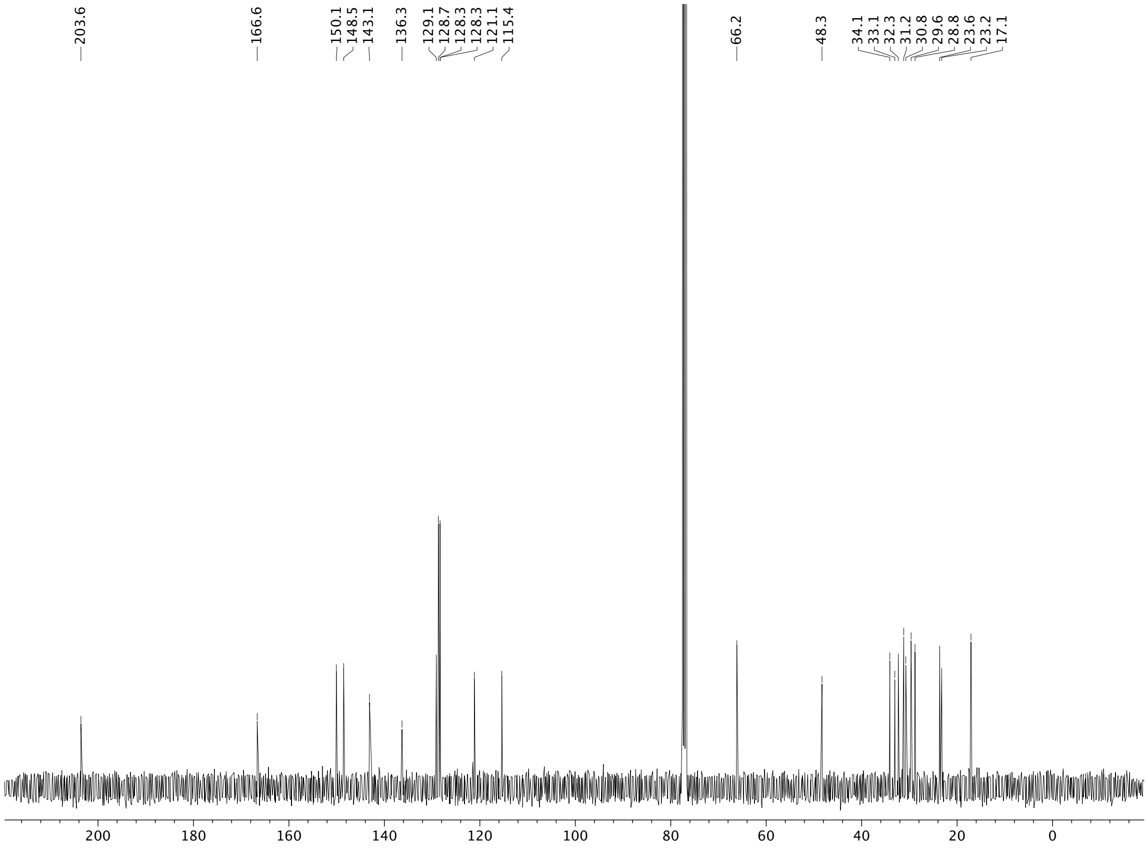

^{13}C NMR (100 MHz, $CDCl_3$) of compound **S5**.

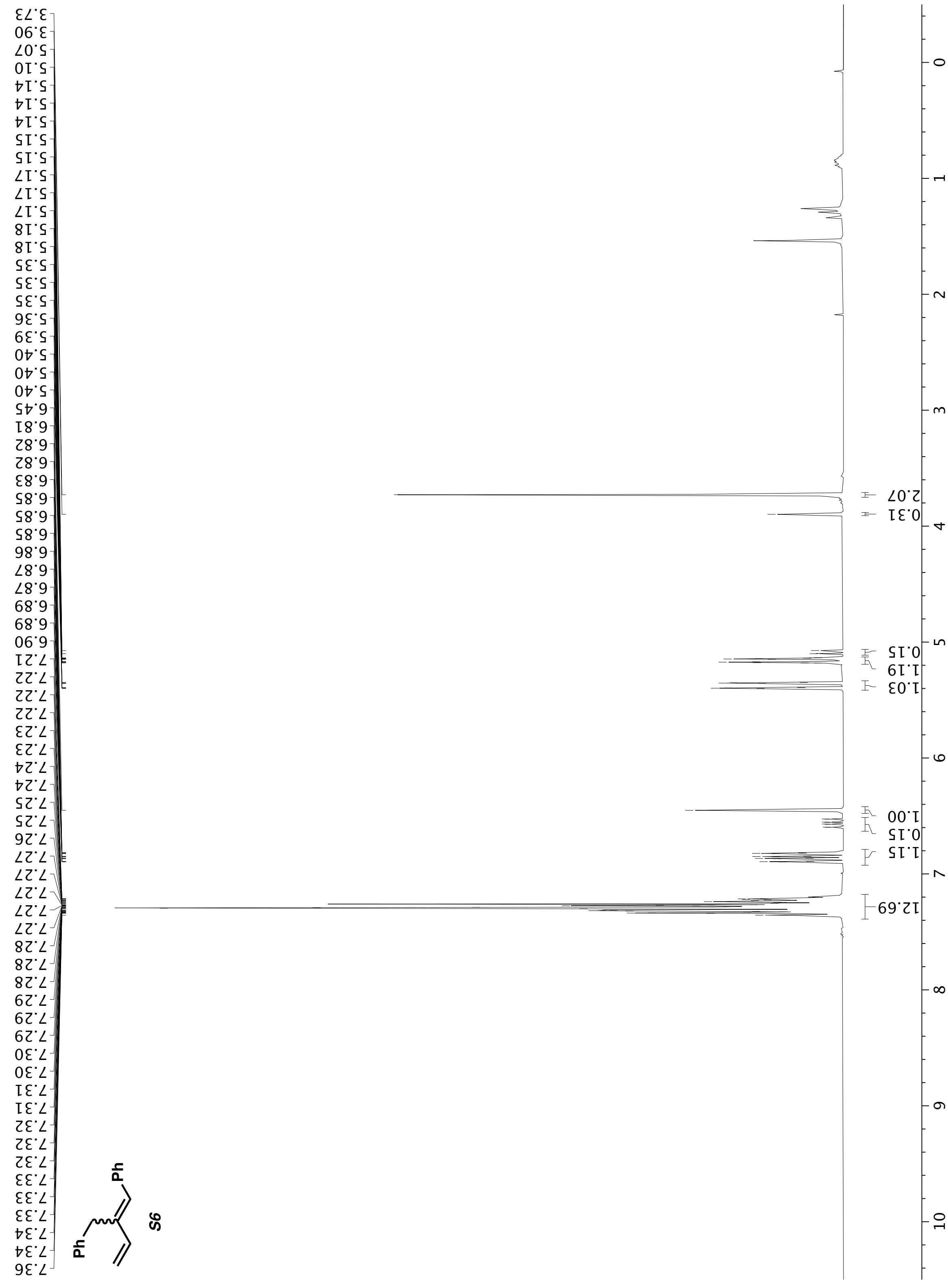

^{1}H NMR (400 MHz, $CDCl_3$) of compound **S6**.

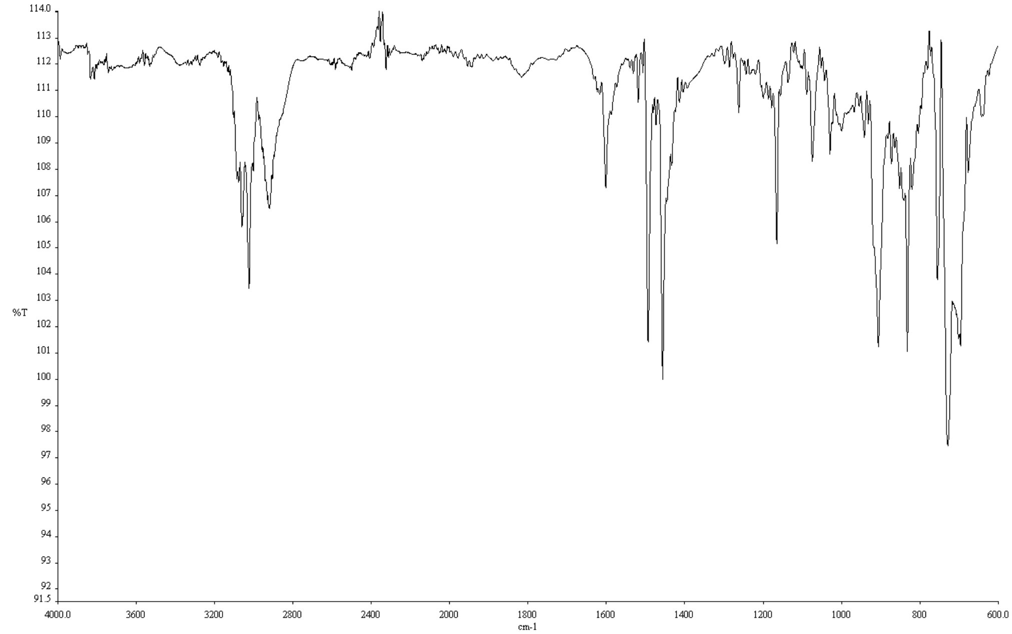

Infrared spectrum (Thin Film, NaCl) of compound **S6**.

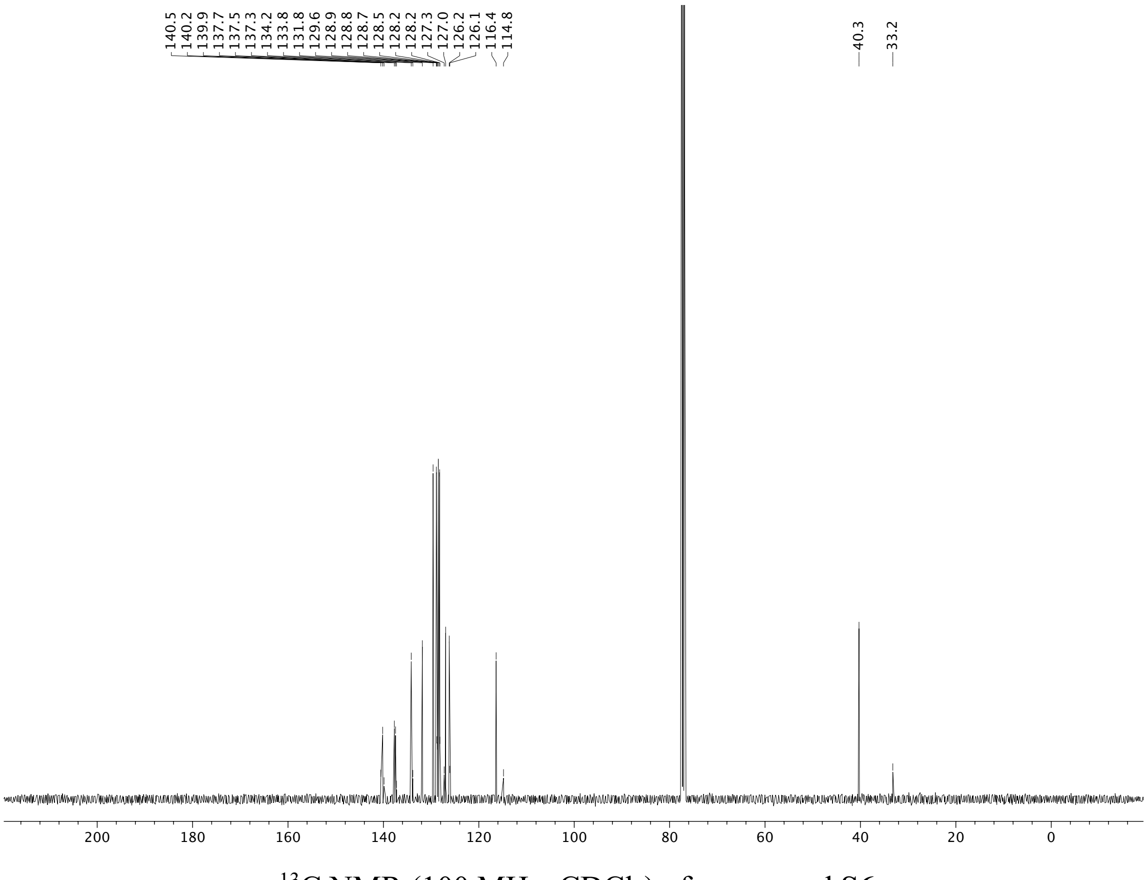

^{13}C NMR (100 MHz, $CDCl_3$) of compound **S6**.

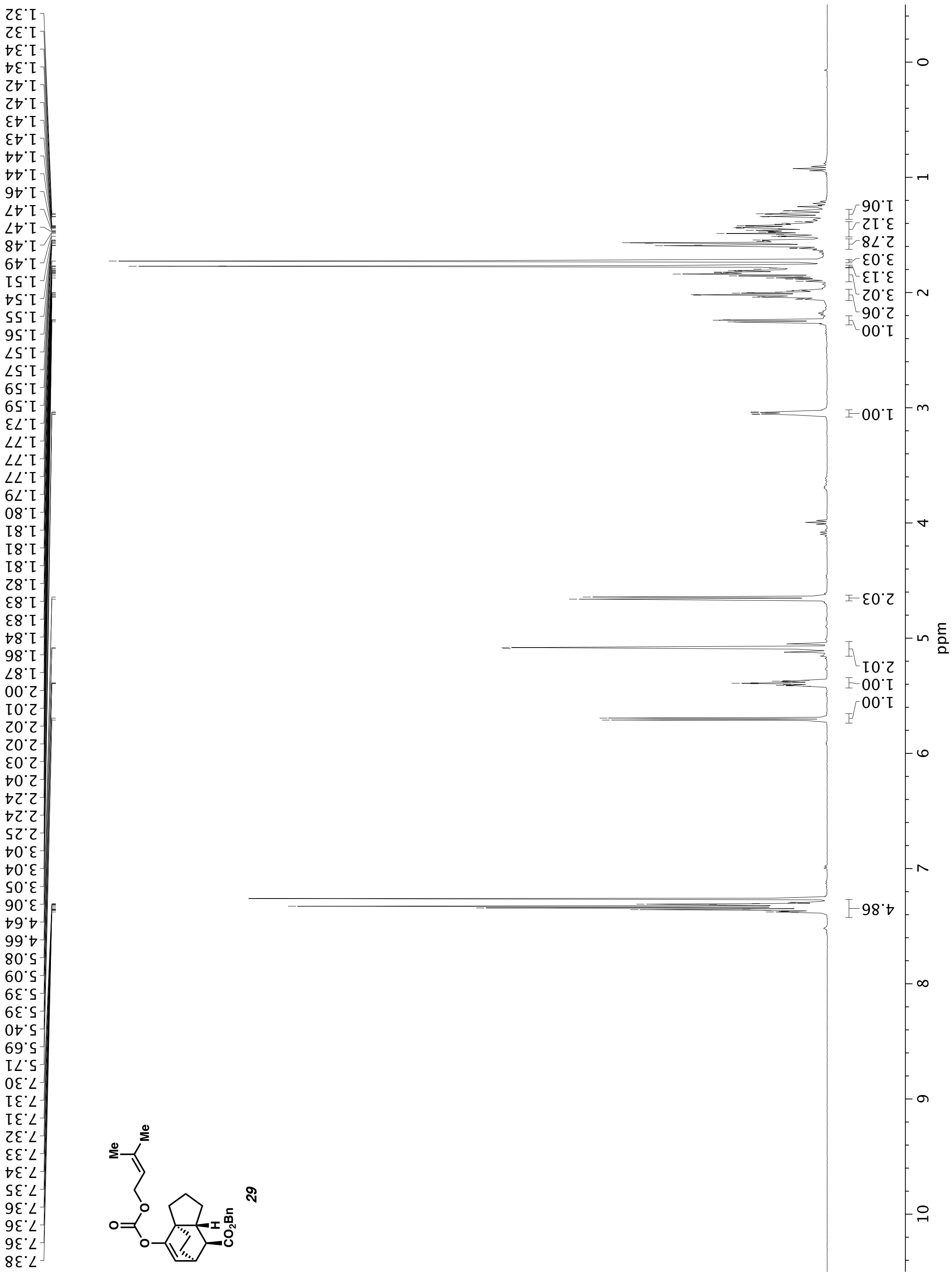

^{1}H NMR (400 MHz, $CDCl_3$) of compound **29.**

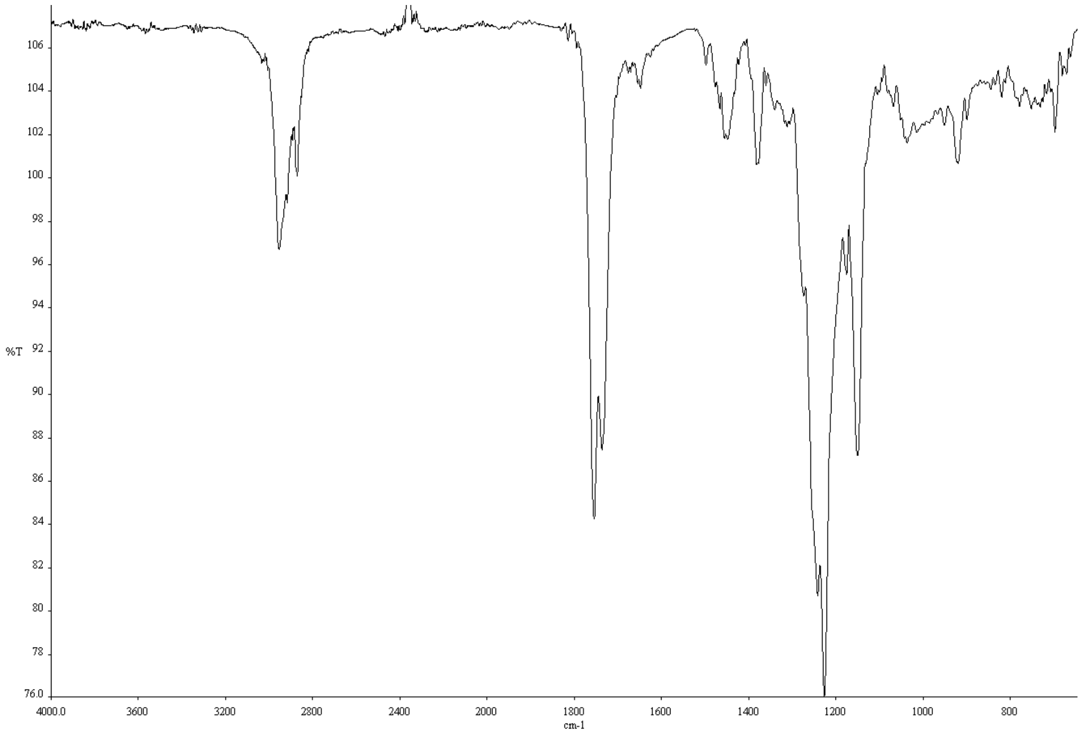

Infrared spectrum (Thin Film, NaCl) of compound **29**.

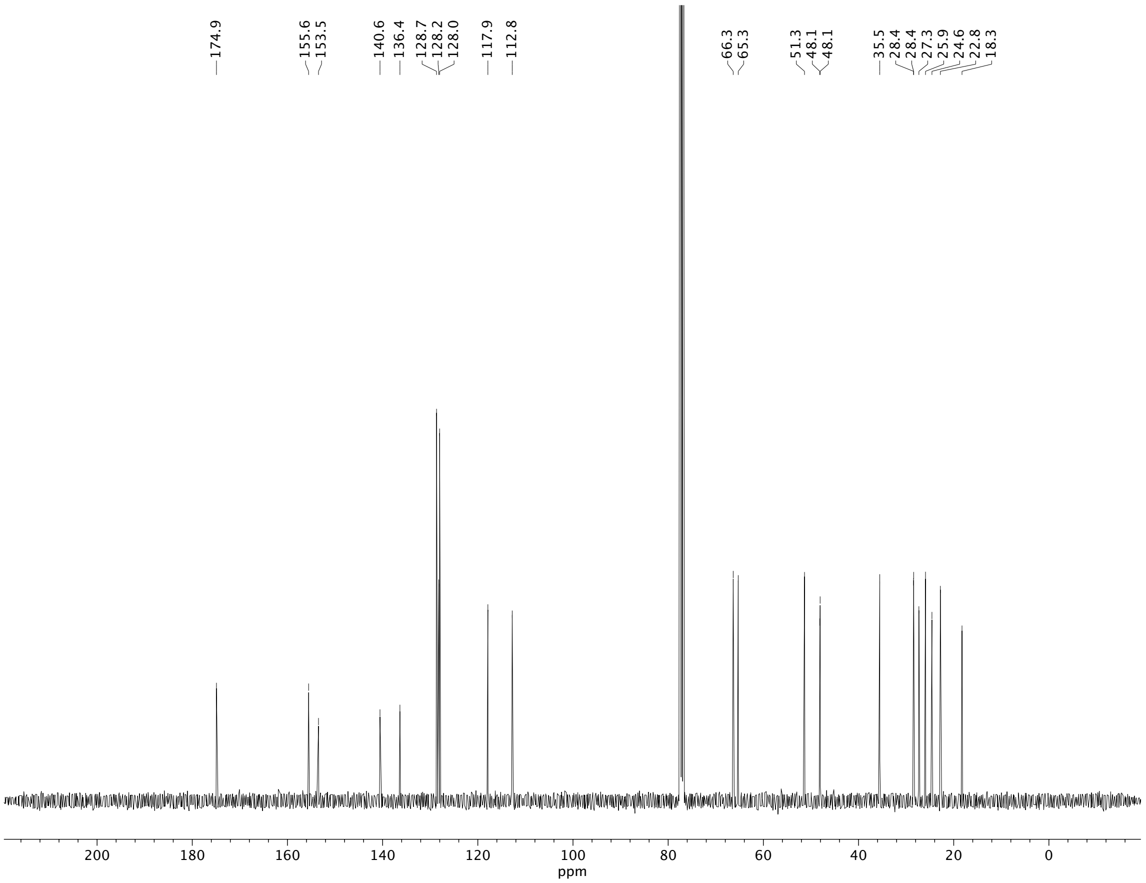

^{13}C NMR (100 MHz, $CDCl_3$) of compound **29**.

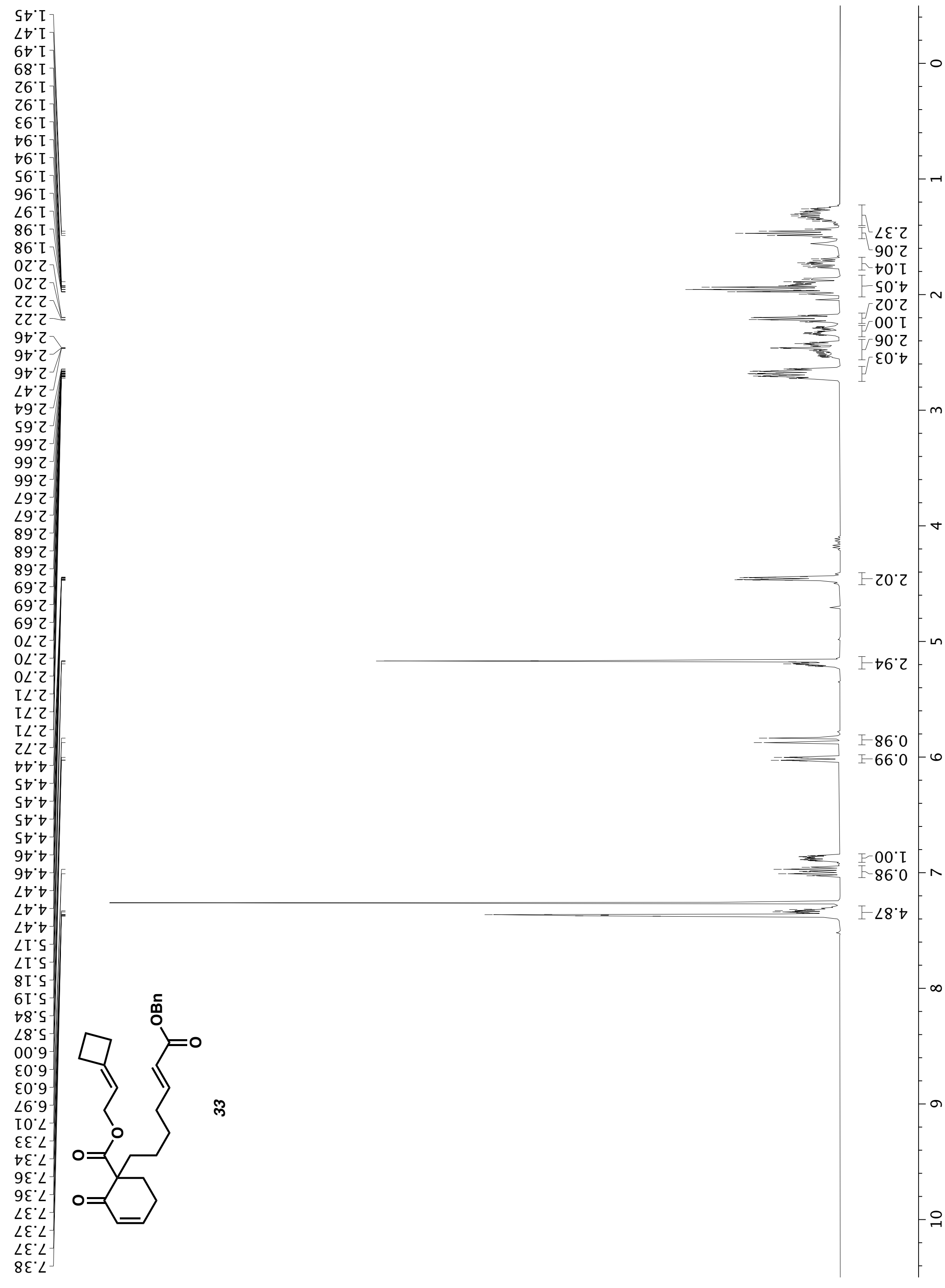

^{1}H NMR (400 MHz, CDCl$_3$) of compound **33**.

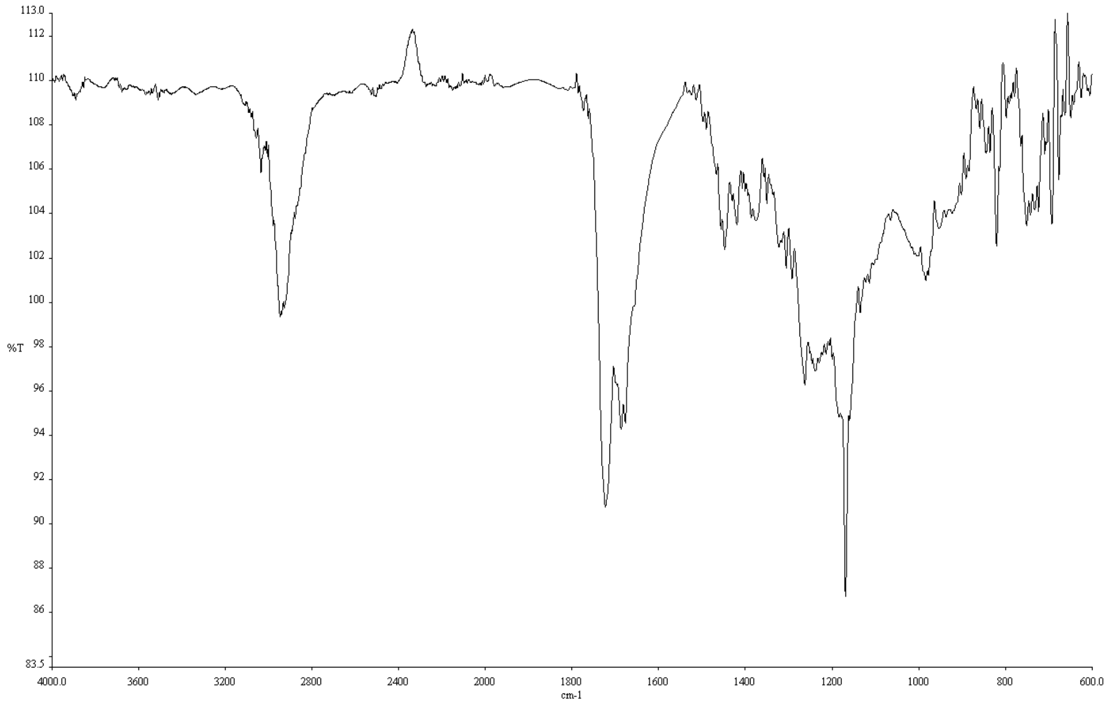

Infrared spectrum (Thin Film, NaCl) of compound **33**.

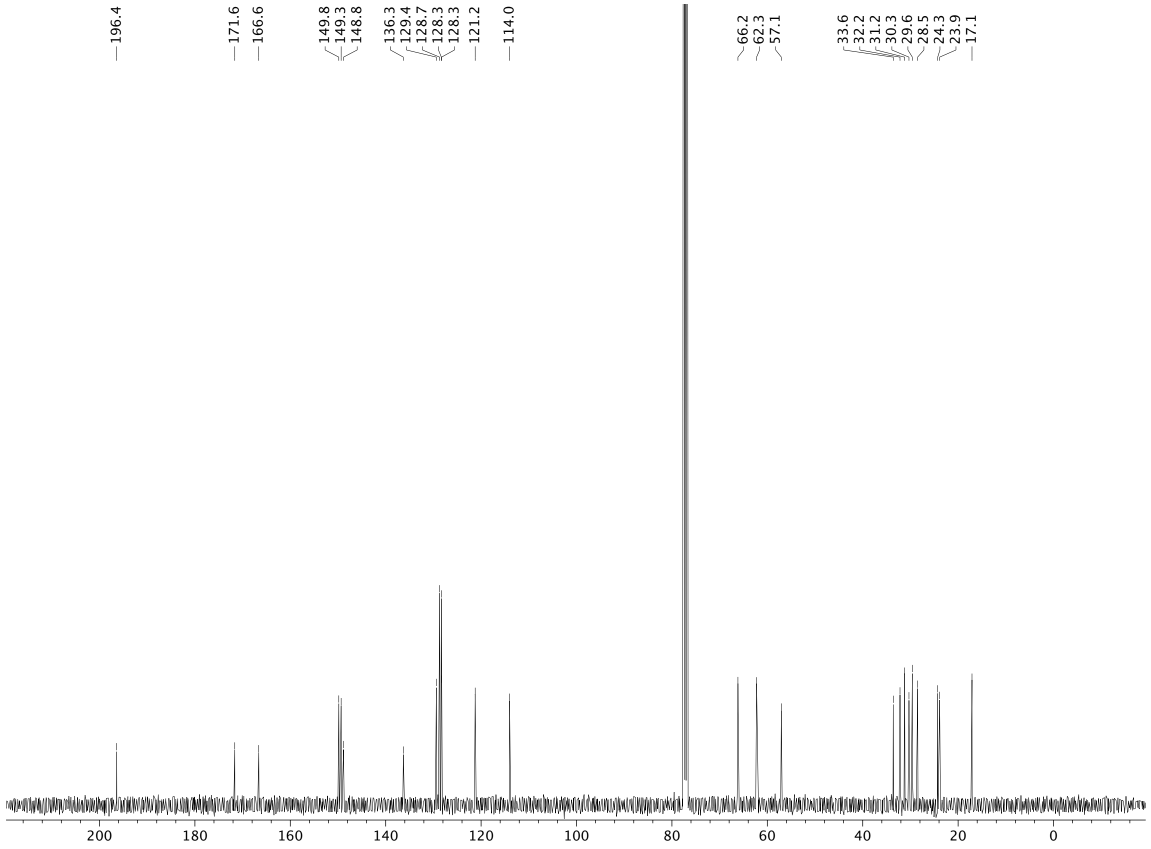

^{13}C NMR (100 MHz, $CDCl_3$) of compound **33**.

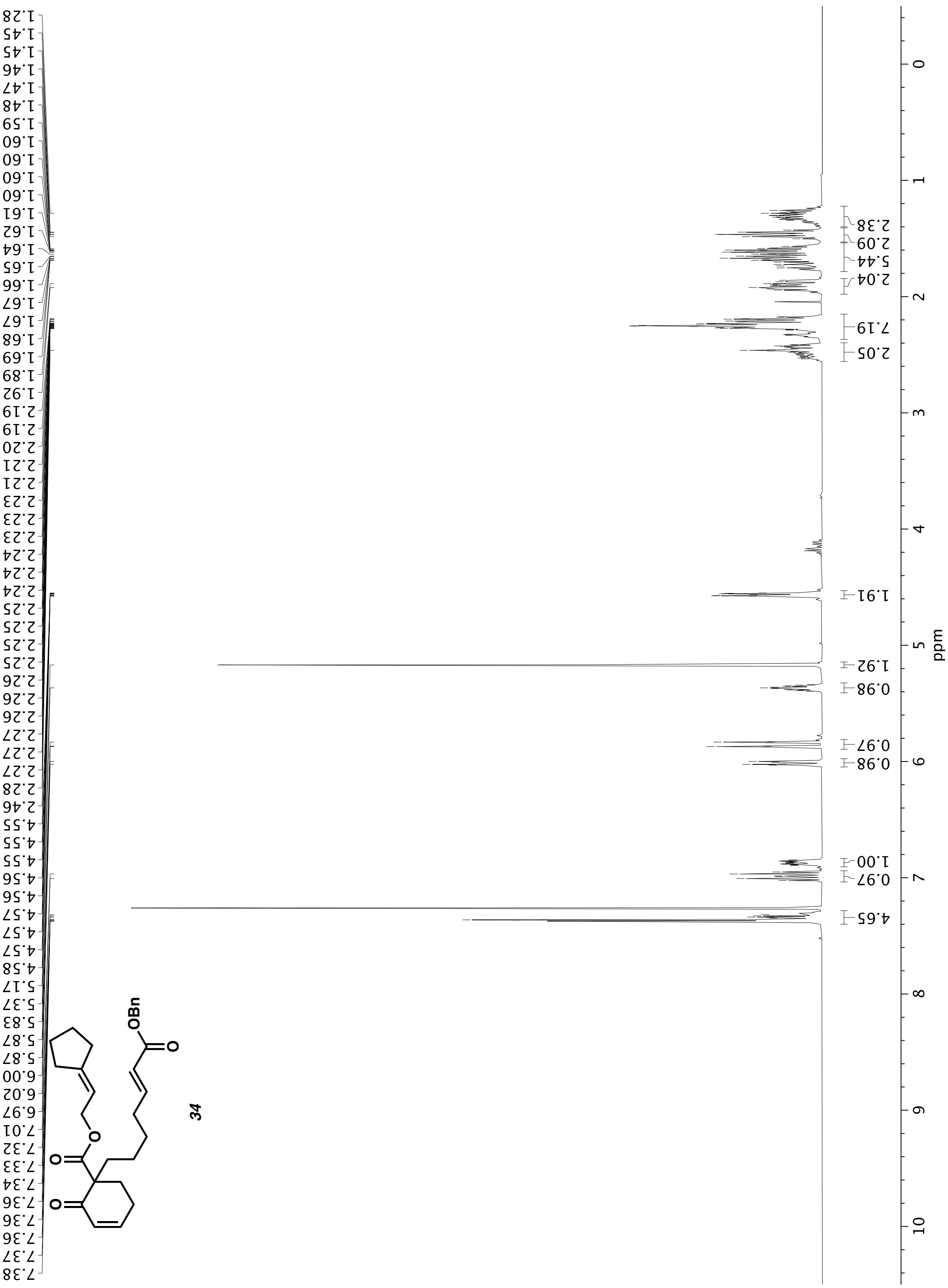

^{1}H NMR (400 MHz, $CDCl_3$) of compound **34**.

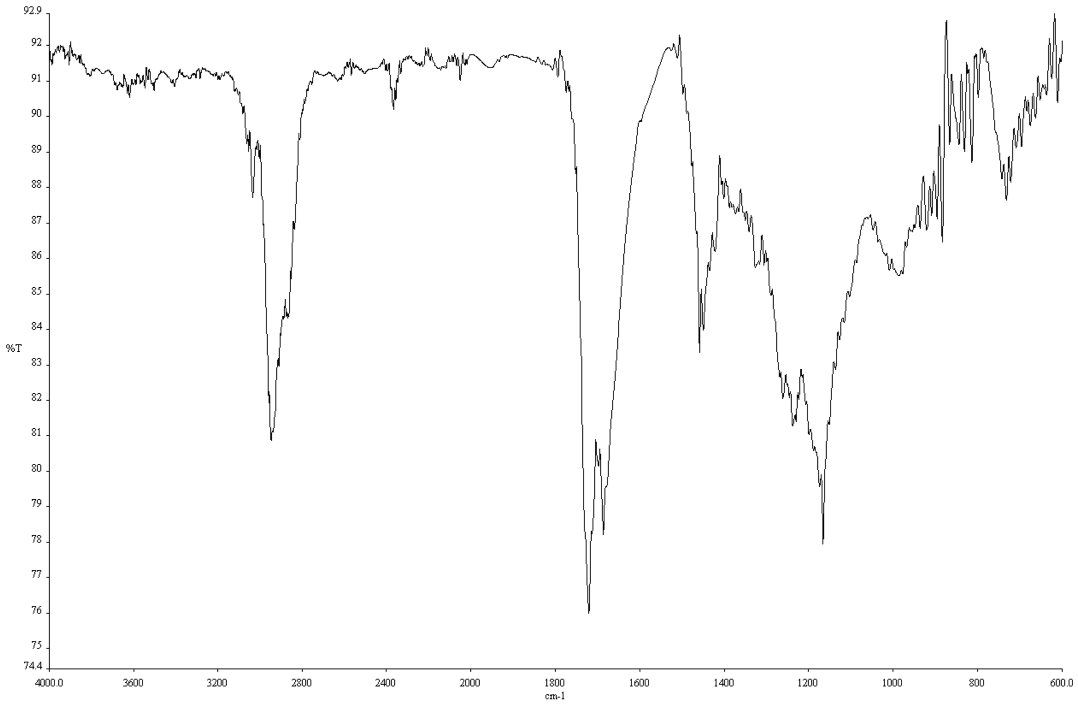

Infrared spectrum (Thin Film, NaCl) of compound **34**.

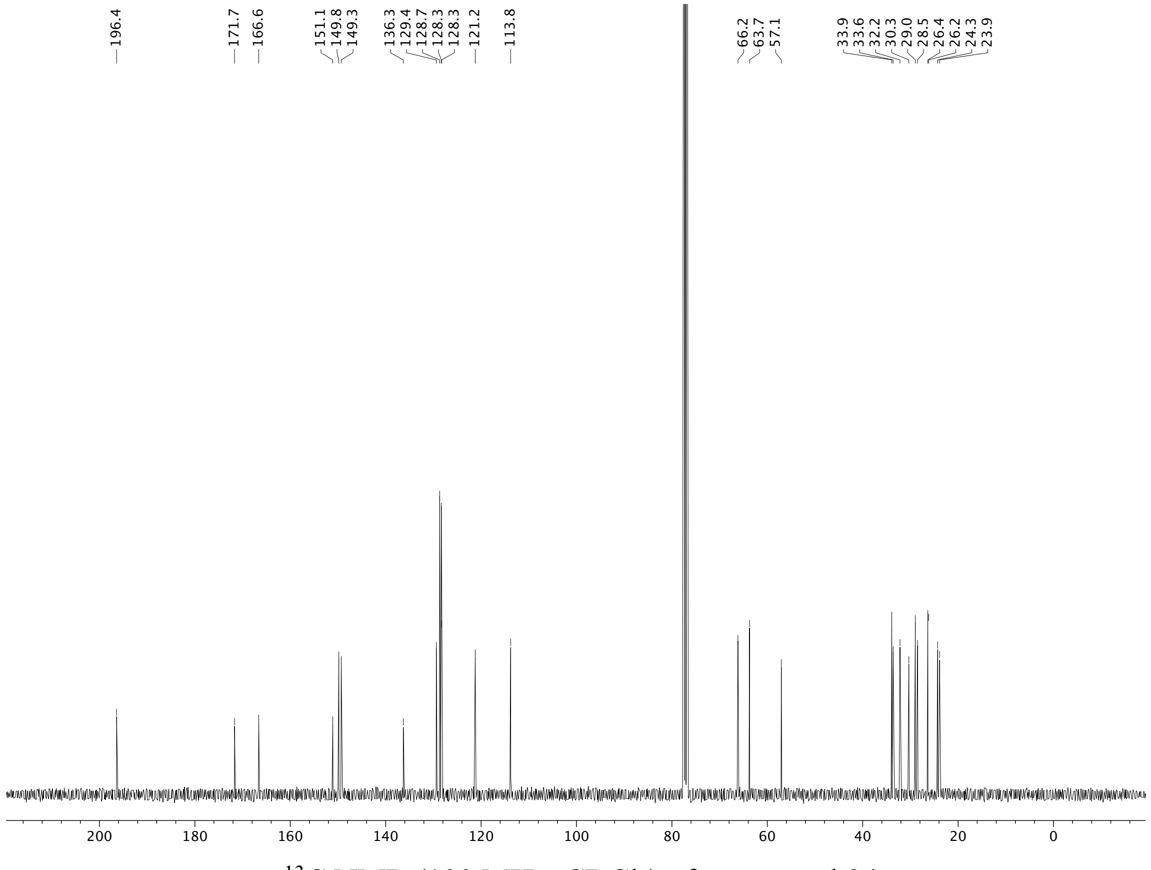

^{13}C NMR (100 MHz, $CDCl_3$) of compound **34**.

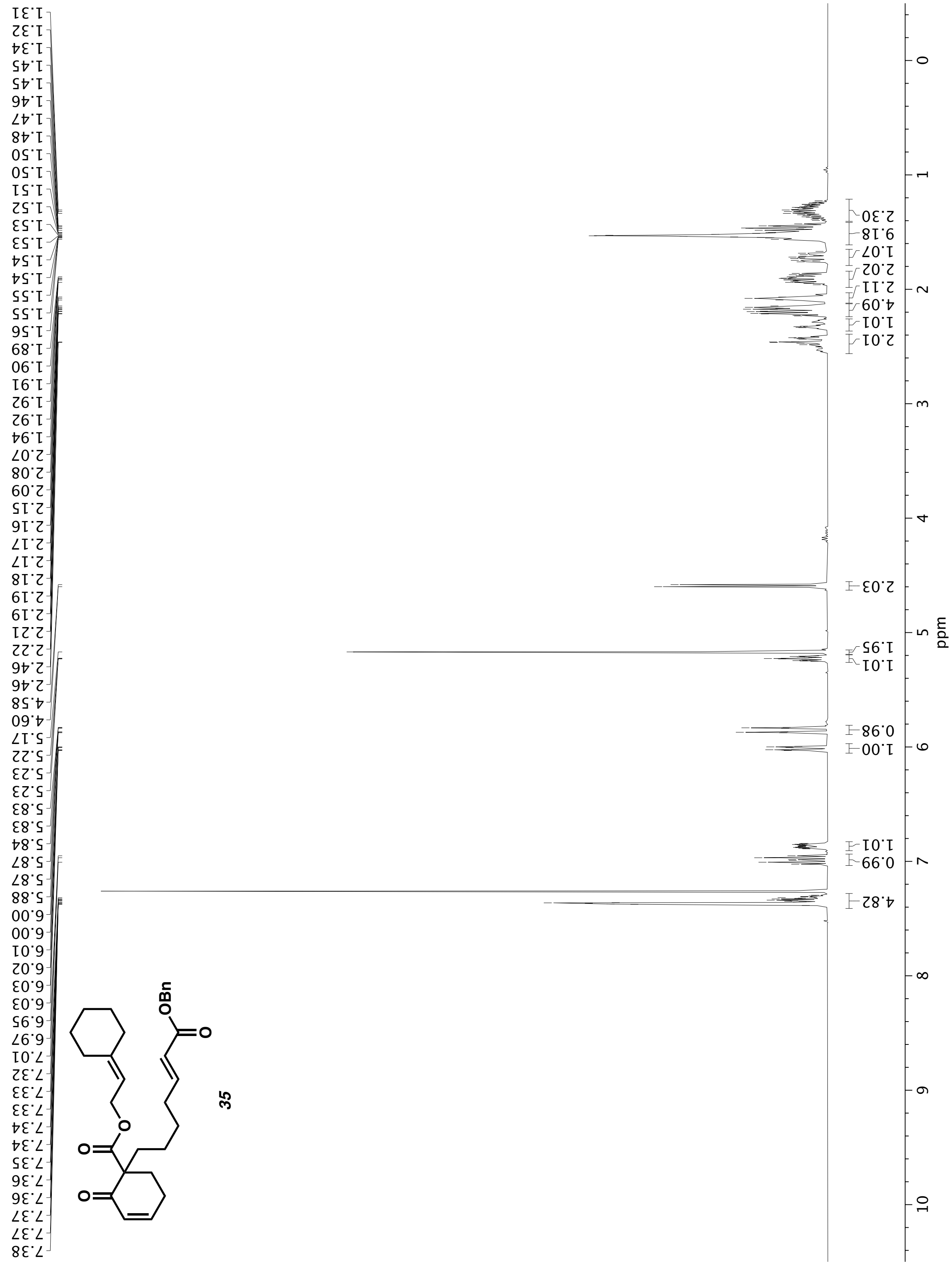

^{1}H NMR (400 MHz, CDCl$_3$) of compound **35**.

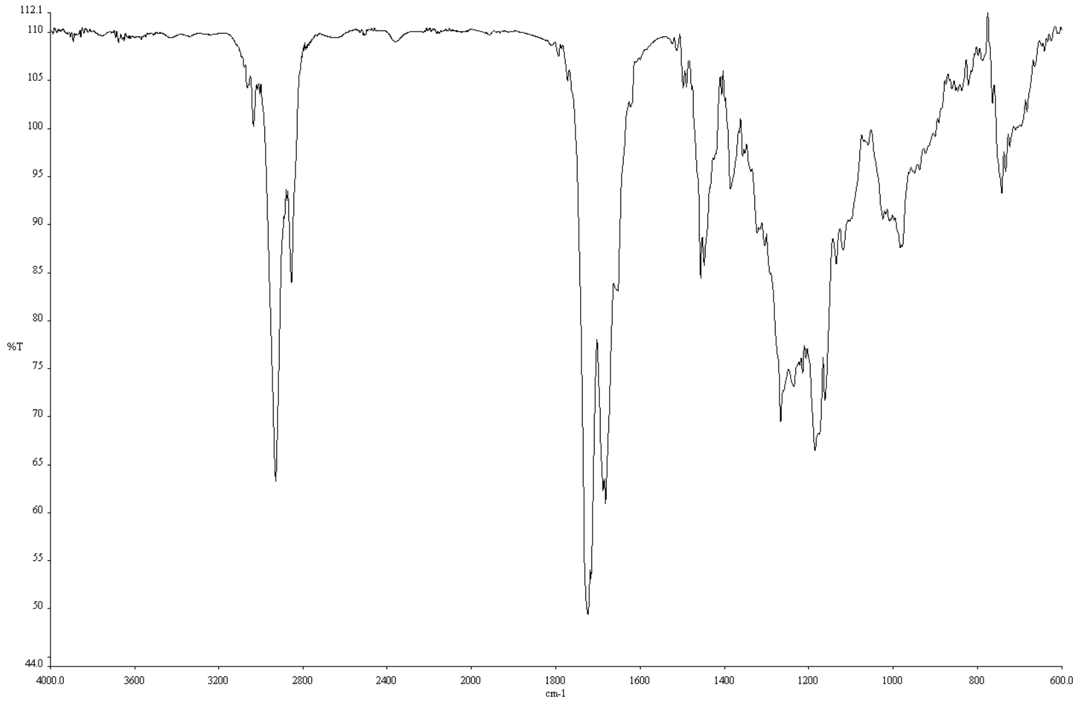

Infrared spectrum (Thin Film, NaCl) of compound **35**.

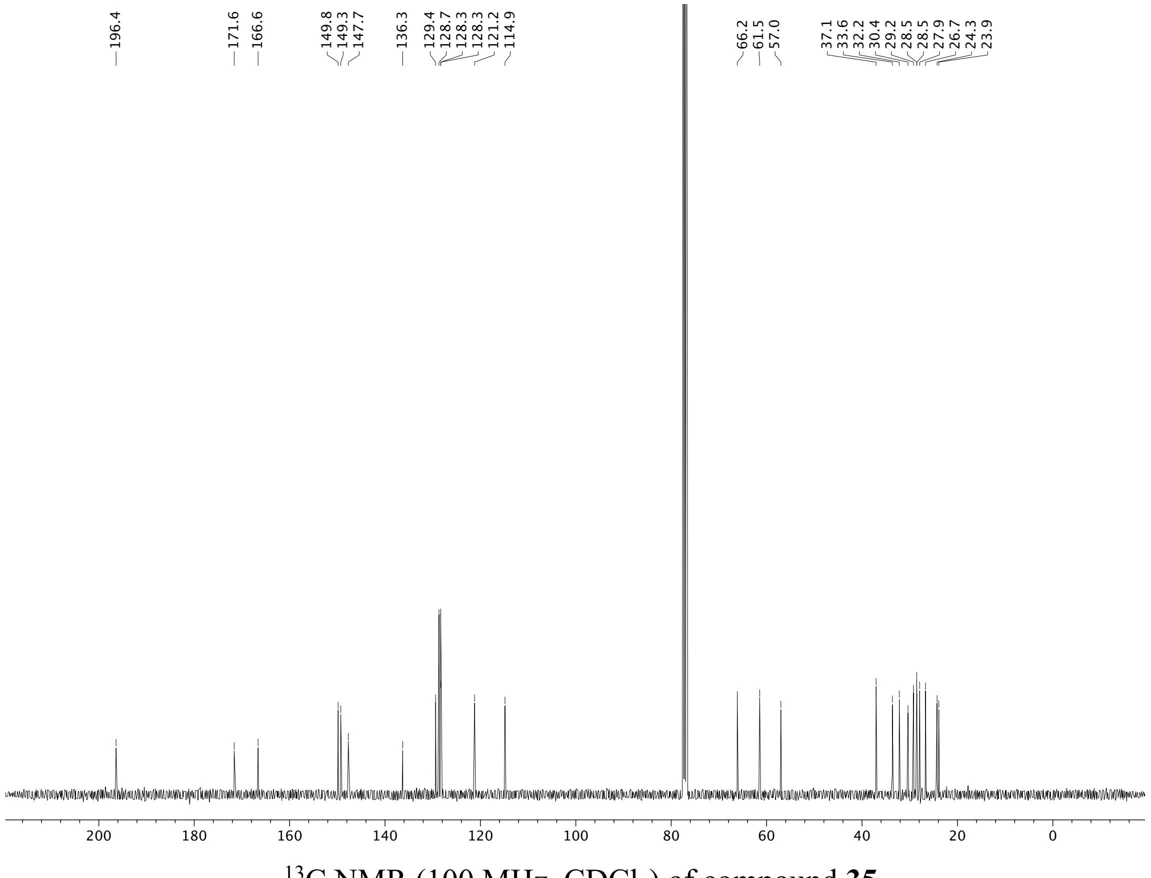

^{13}C NMR (100 MHz, $CDCl_3$) of compound **35**.

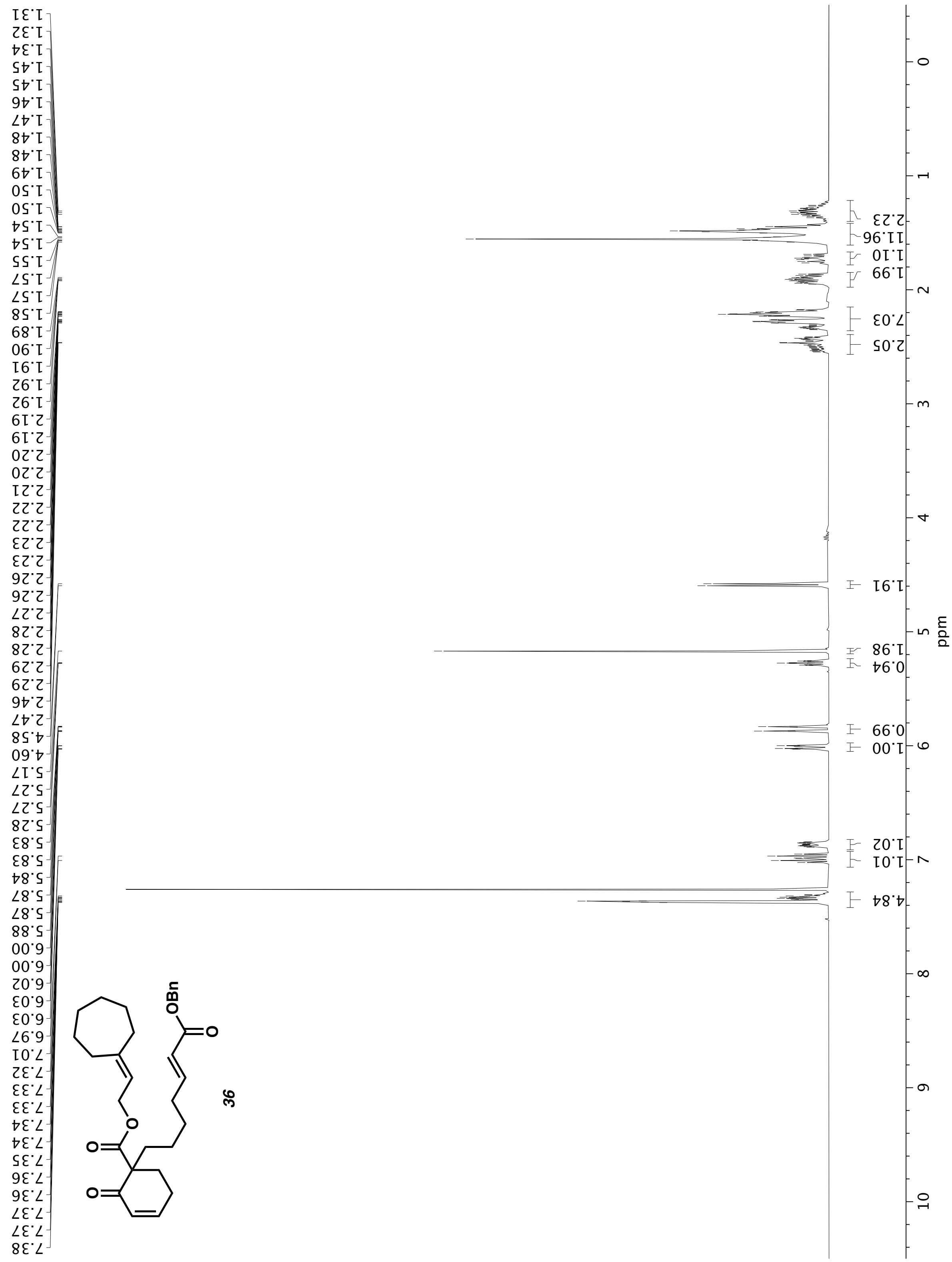

^{1}H NMR (400 MHz, $CDCl_3$) of compound **36**.

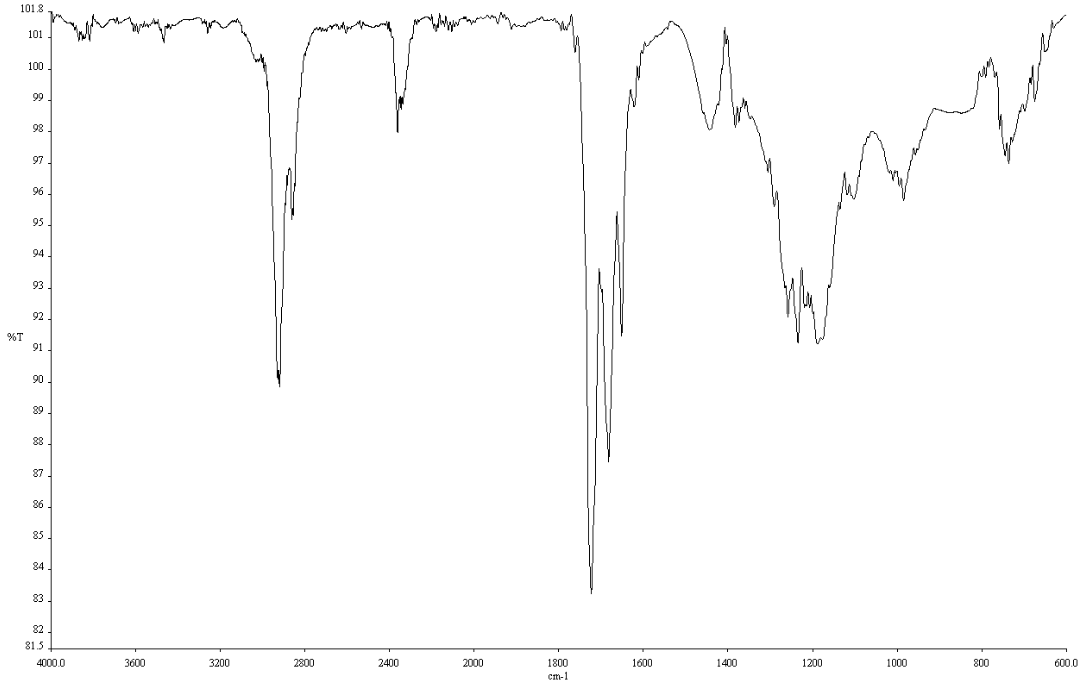

Infrared spectrum (Thin Film, NaCl) of compound **36**.

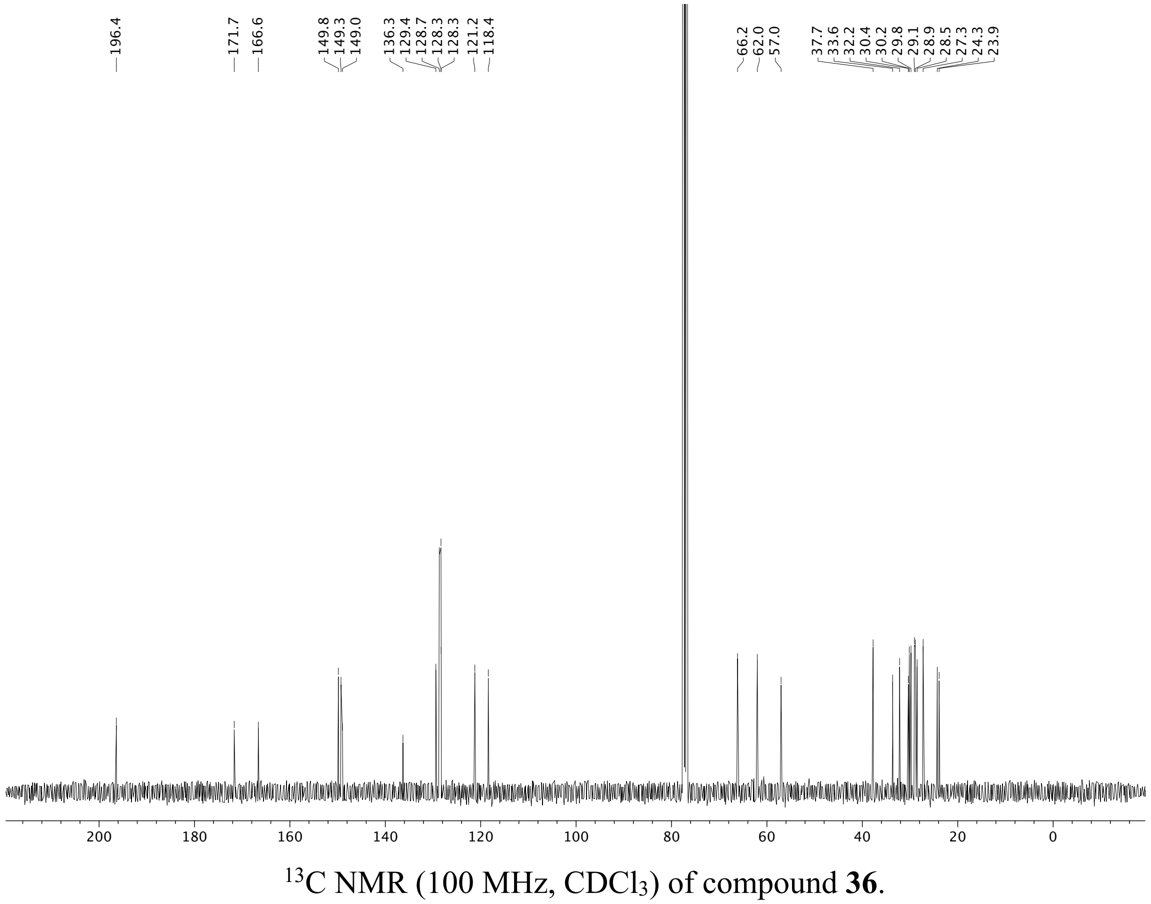

^{13}C NMR (100 MHz, $CDCl_3$) of compound **36**.

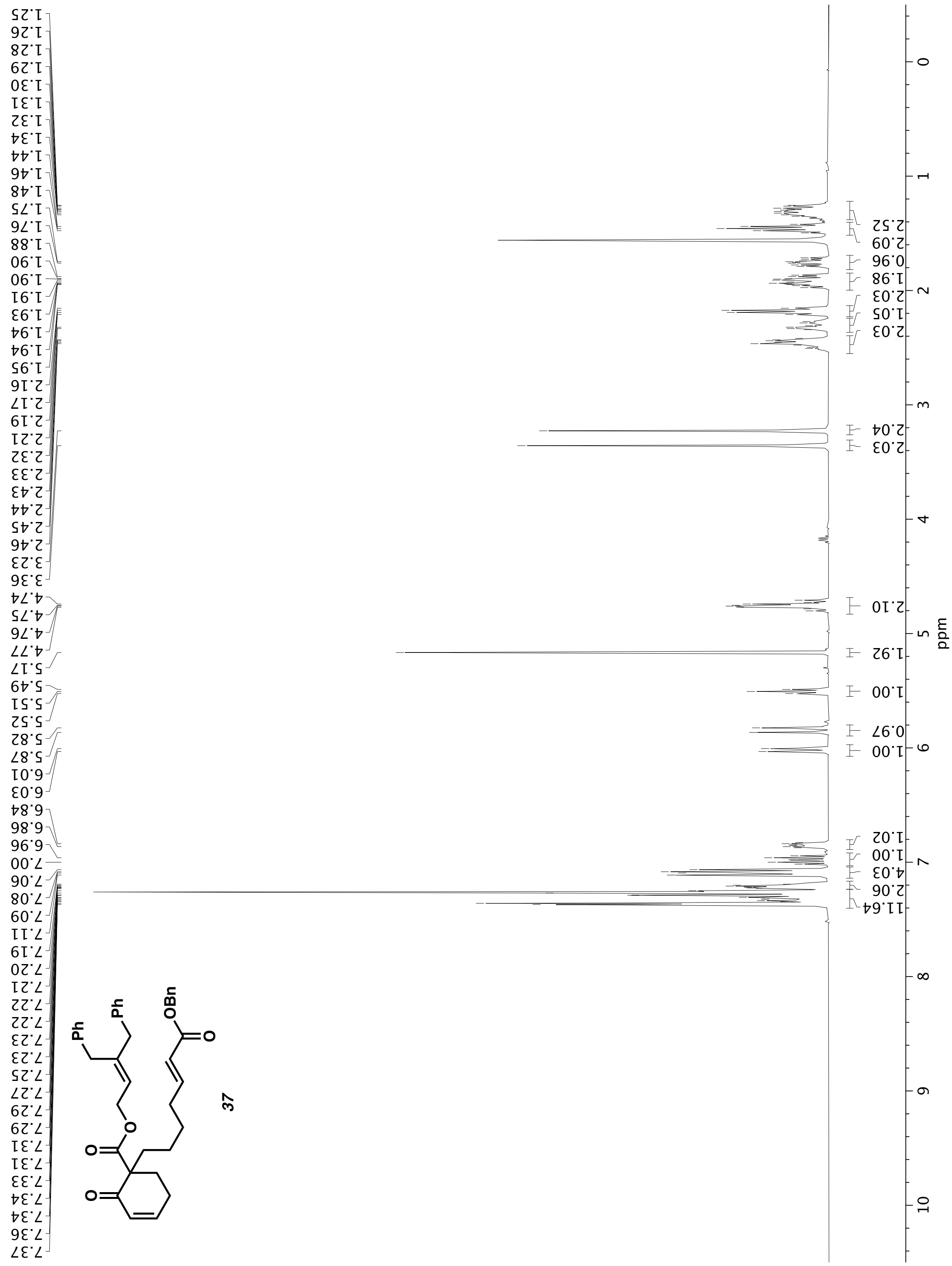

^{1}H NMR (400 MHz, CDCl$_3$) of compound **37**.

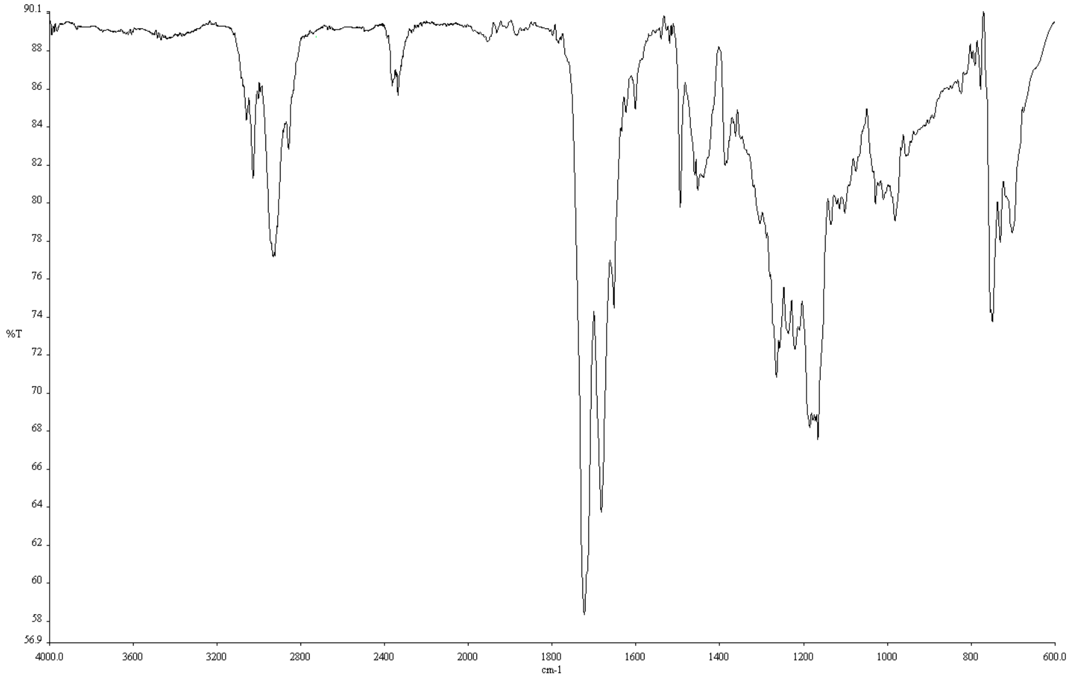

Infrared spectrum (Thin Film, NaCl) of compound **37**.

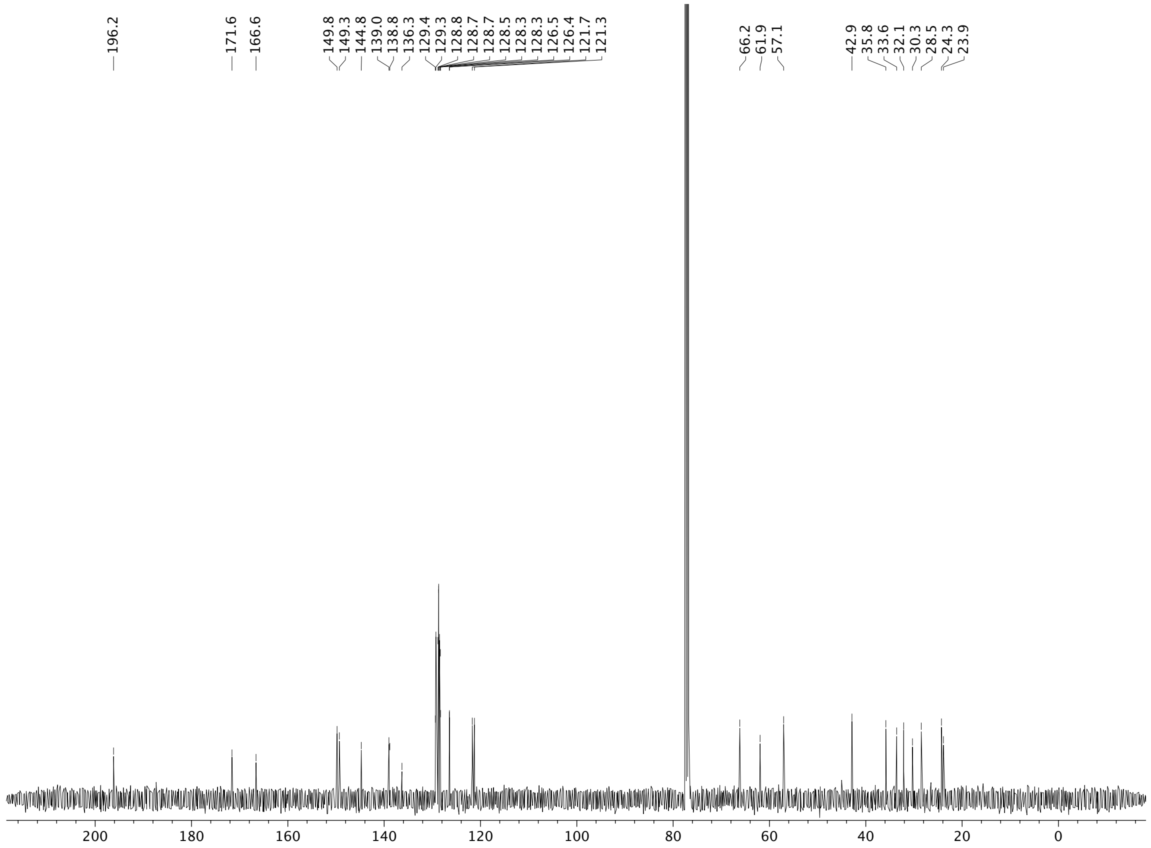

^{13}C NMR (100 MHz, $CDCl_3$) of compound **37**.

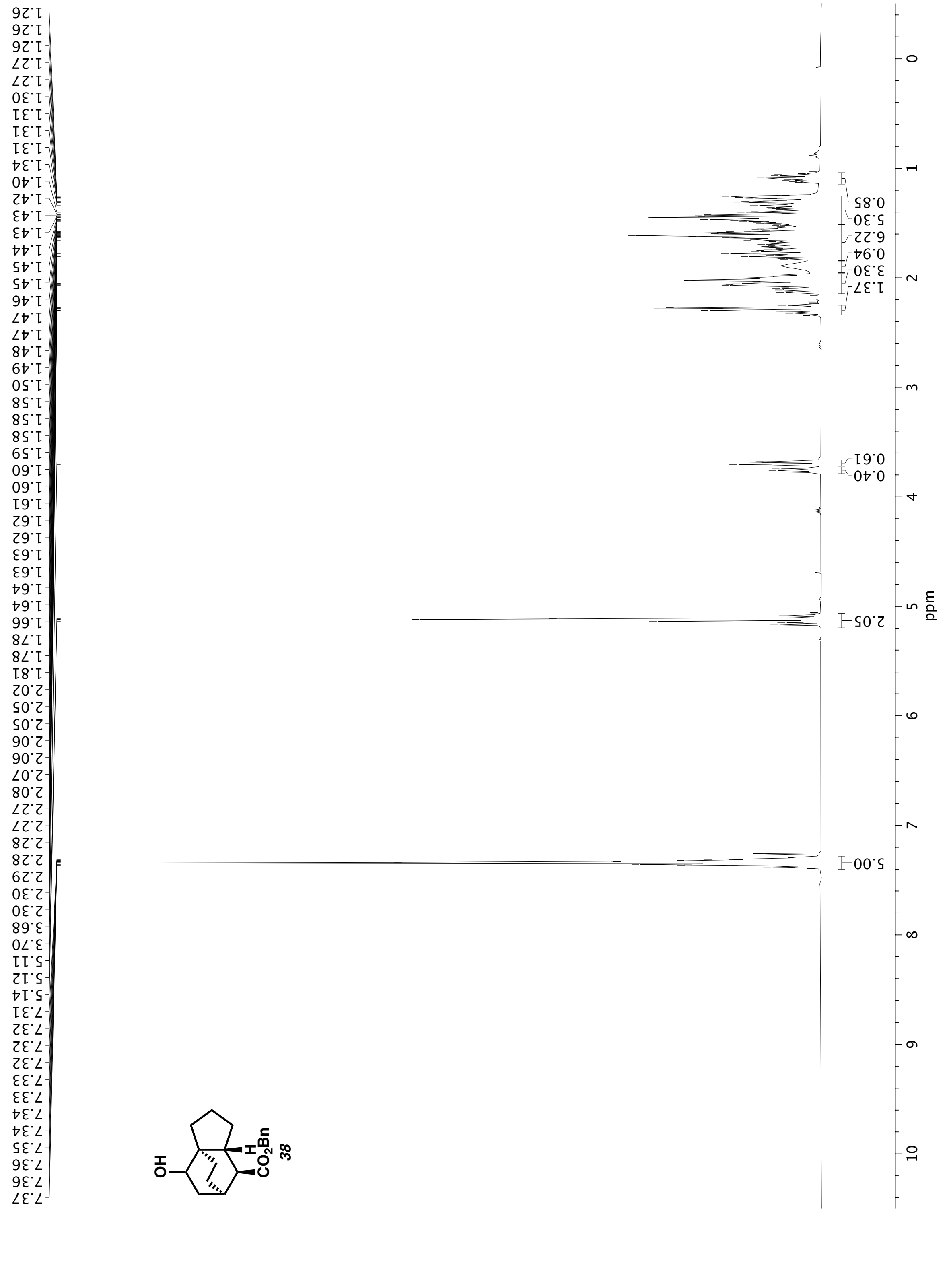

^{1}H NMR (400 MHz, $CDCl_3$) of compound **38**.

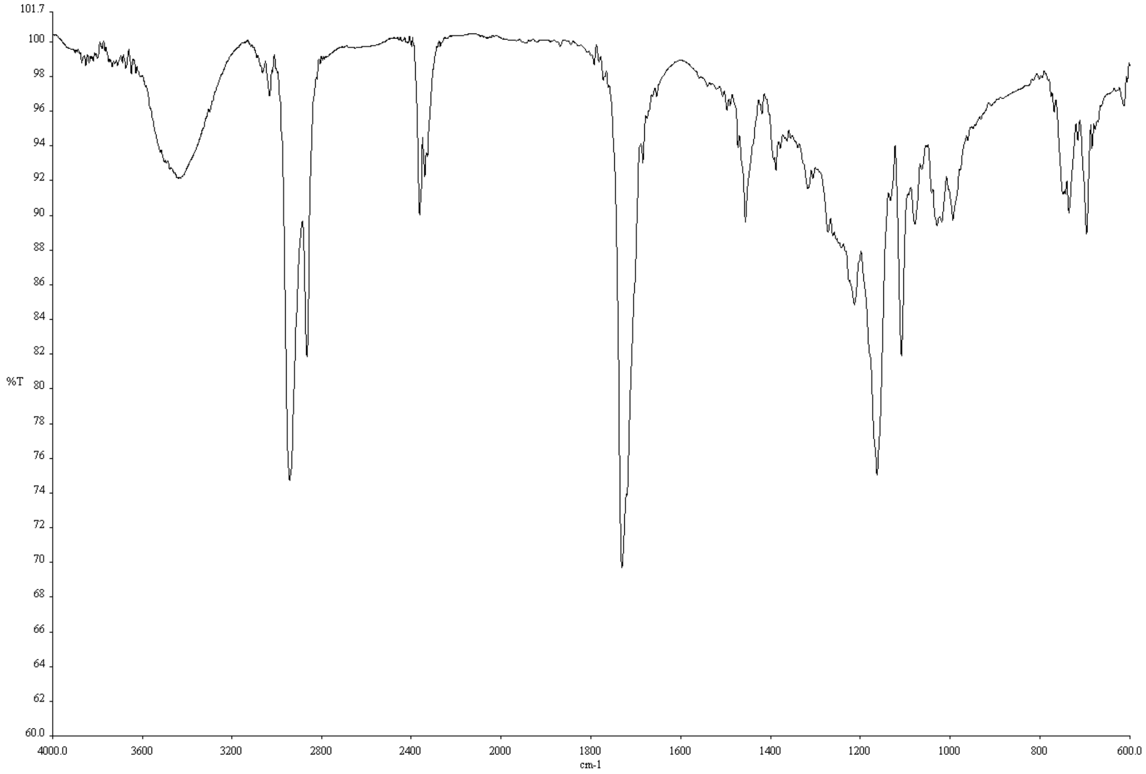

Infrared spectrum (Thin Film, NaCl) of compound **38**.

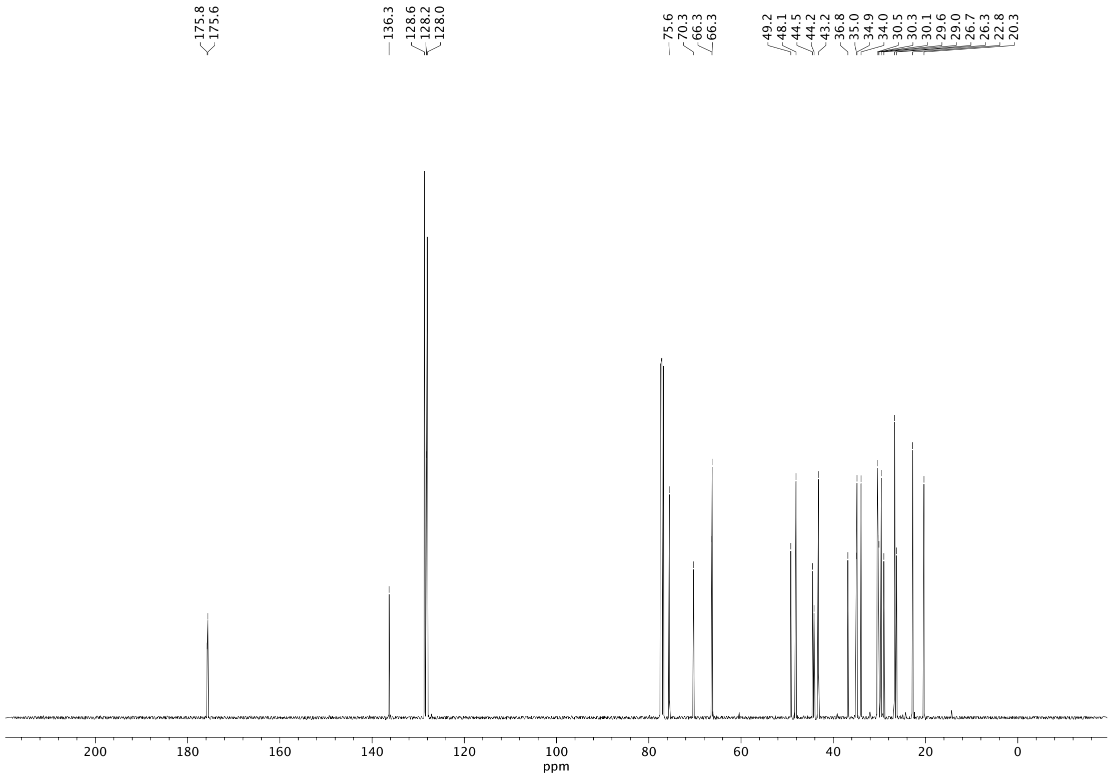

^{13}C NMR (100 MHz, $CDCl_3$) of compound **38**.

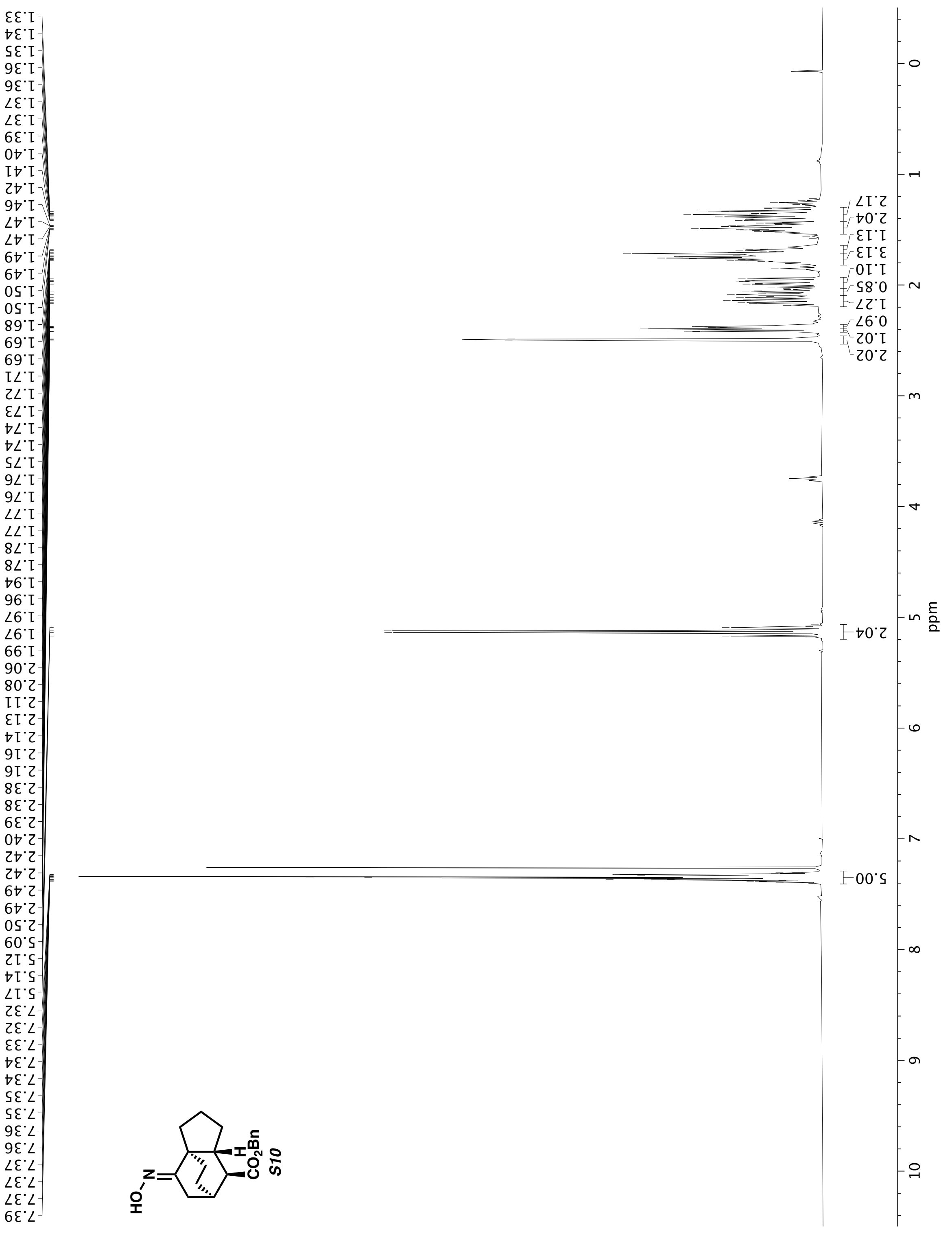

^{1}H NMR (400 MHz, $CDCl_3$) of compound **S10**.

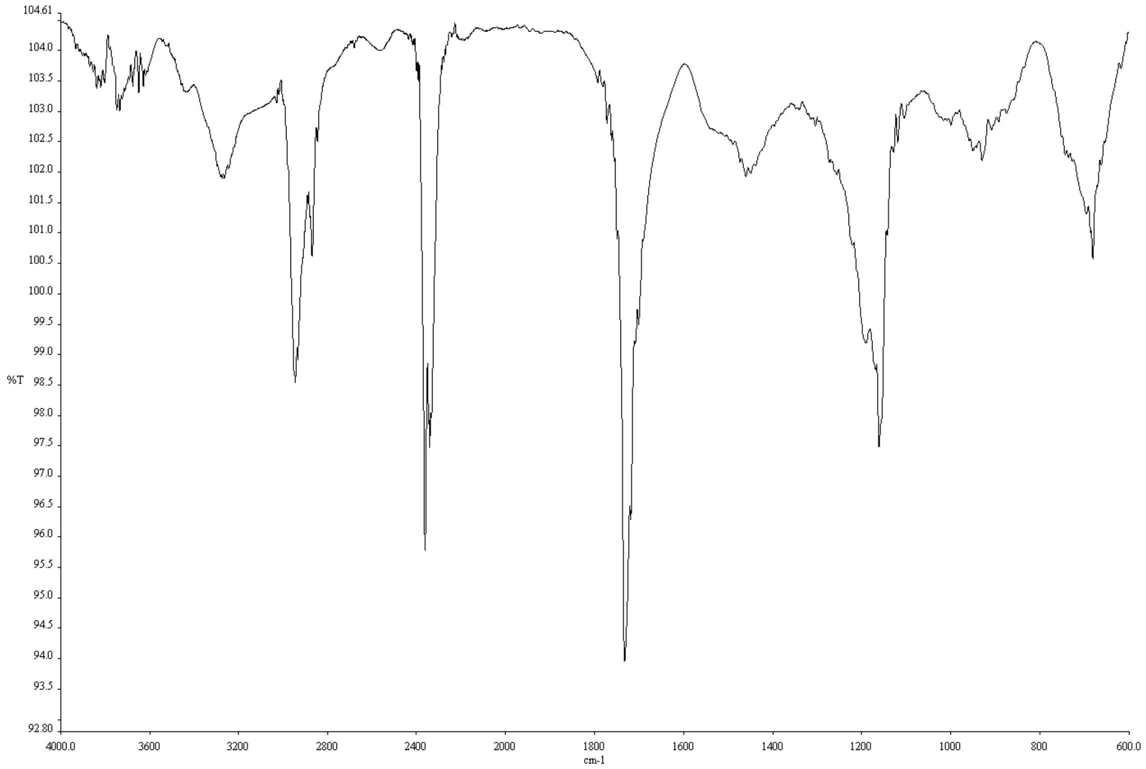

Infrared spectrum (Thin Film, NaCl) of compound **S10**.

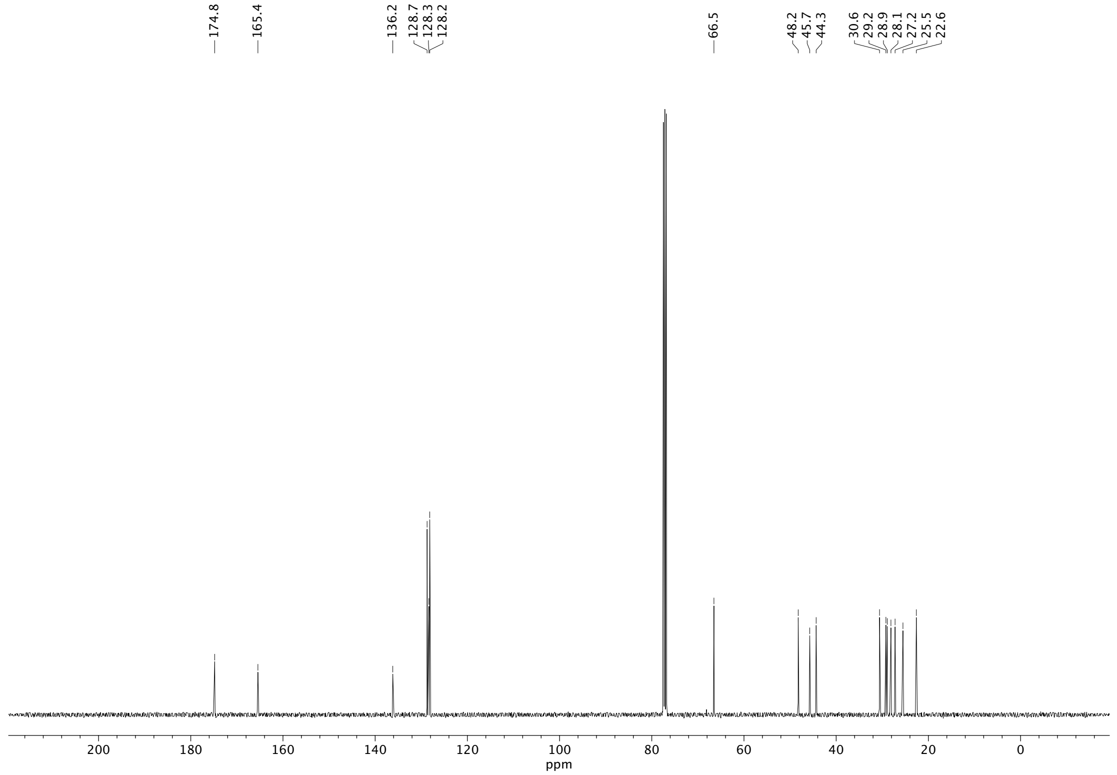

^{13}C NMR (100 MHz, $CDCl_3$) of compound **S10**.

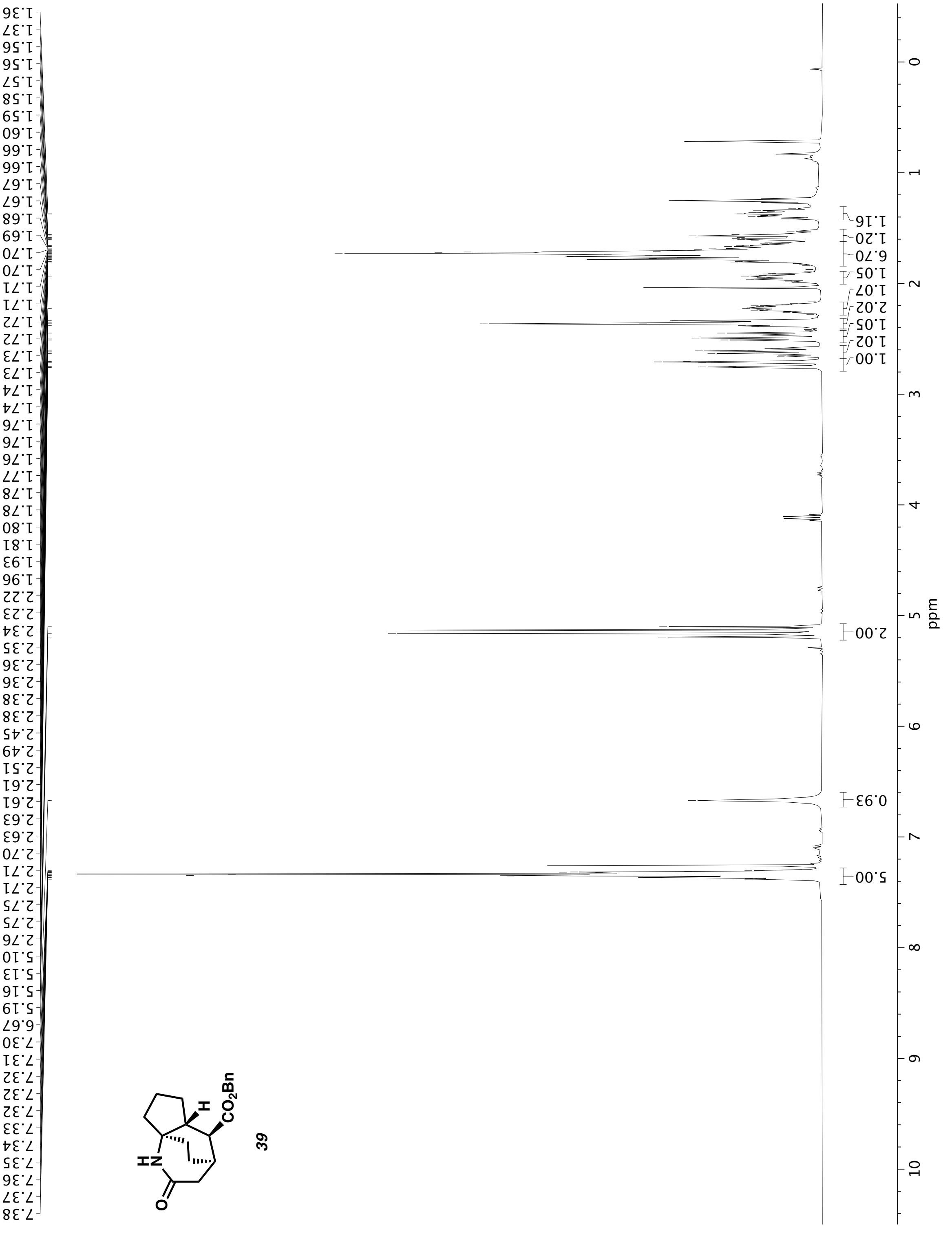

^{1}H NMR (400 MHz, $CDCl_3$) of compound **39**.

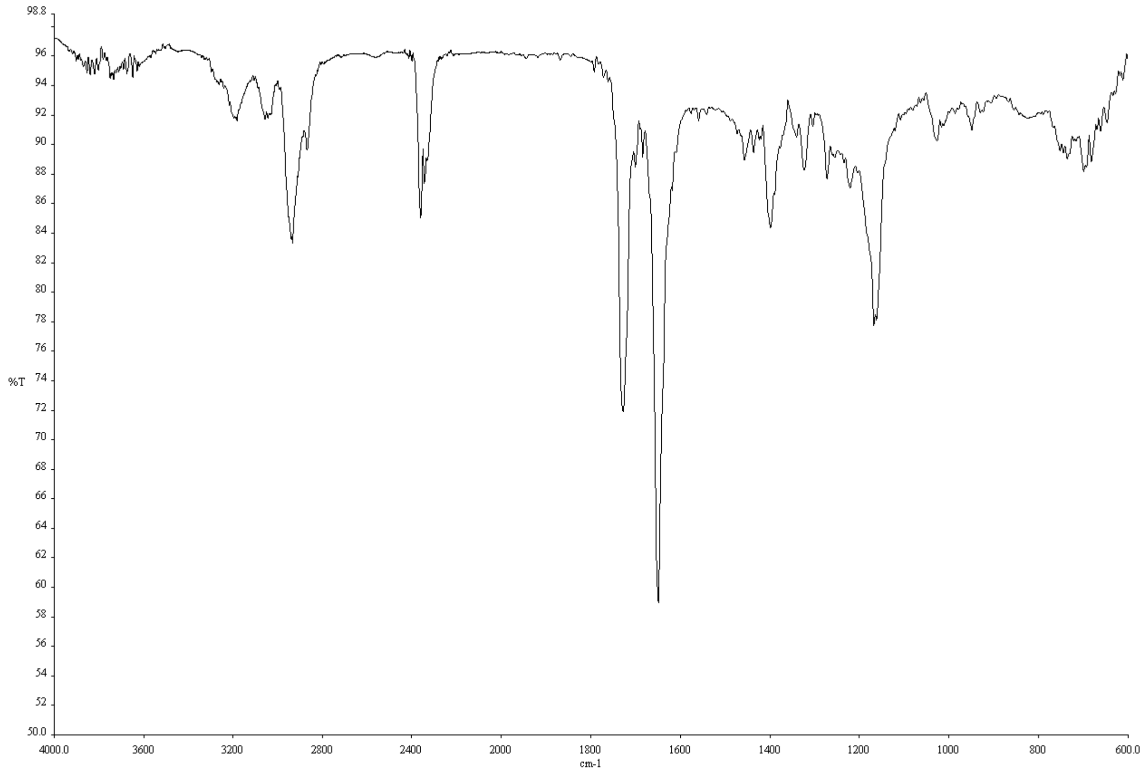

Infrared spectrum (Thin Film, NaCl) of compound **39**.

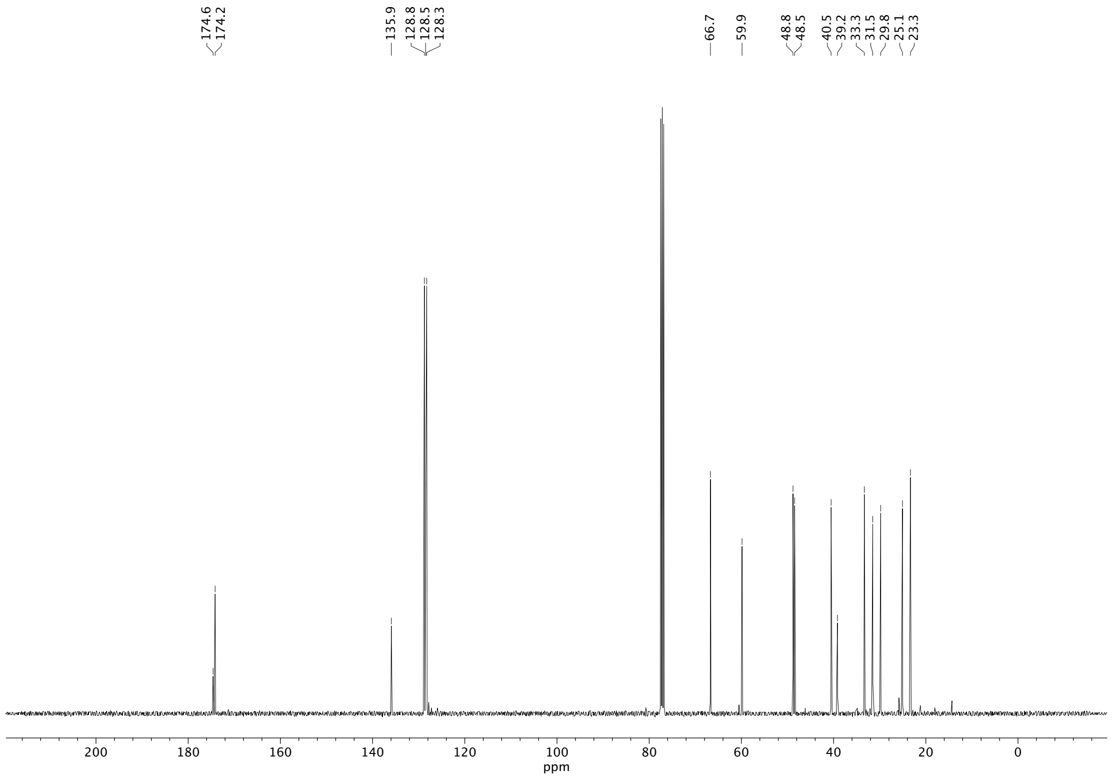

^{13}C NMR (100 MHz, $CDCl_3$) of compound **39**.

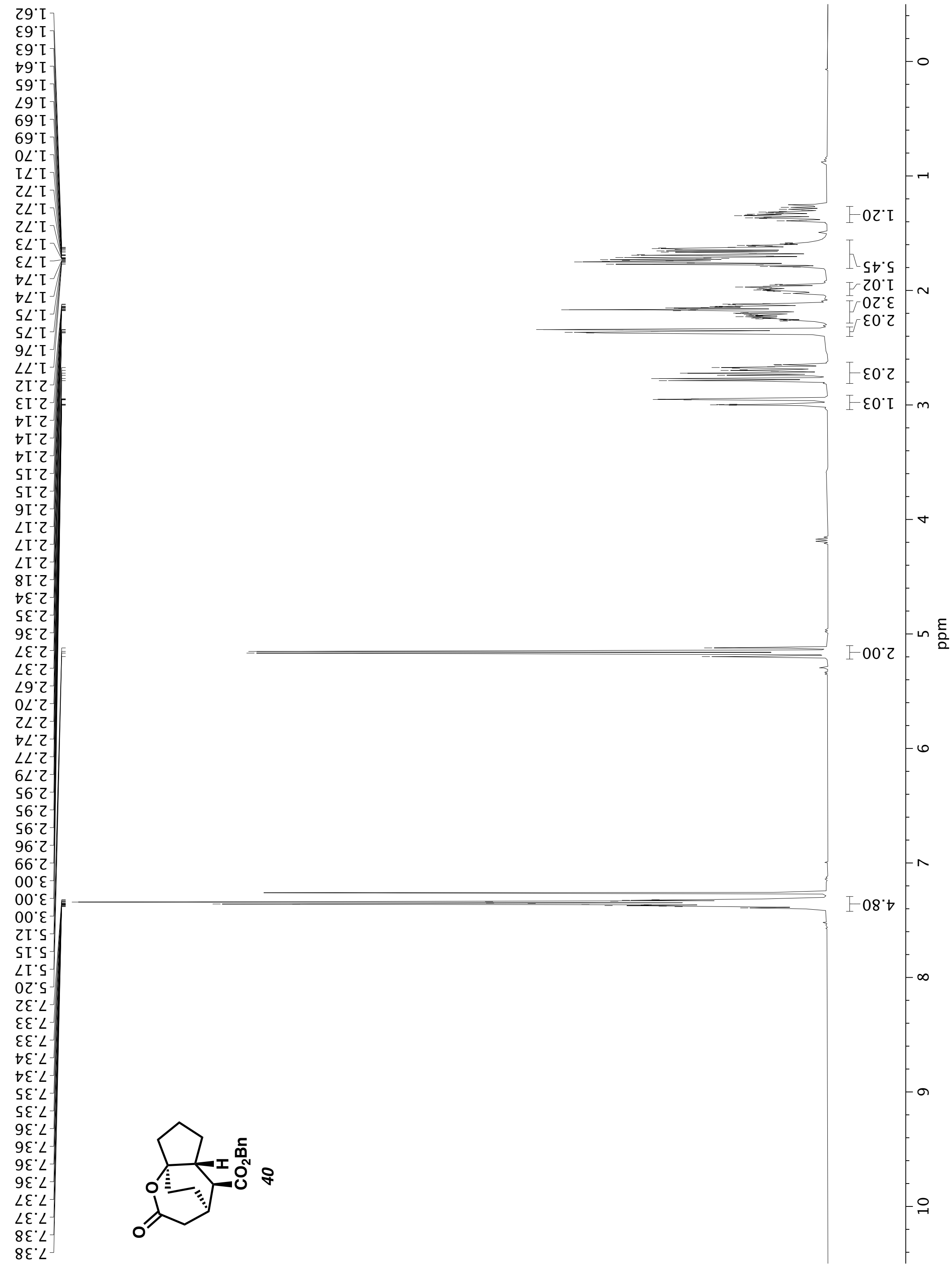

^{1}H NMR (400 MHz, $CDCl_3$) of compound **40**.

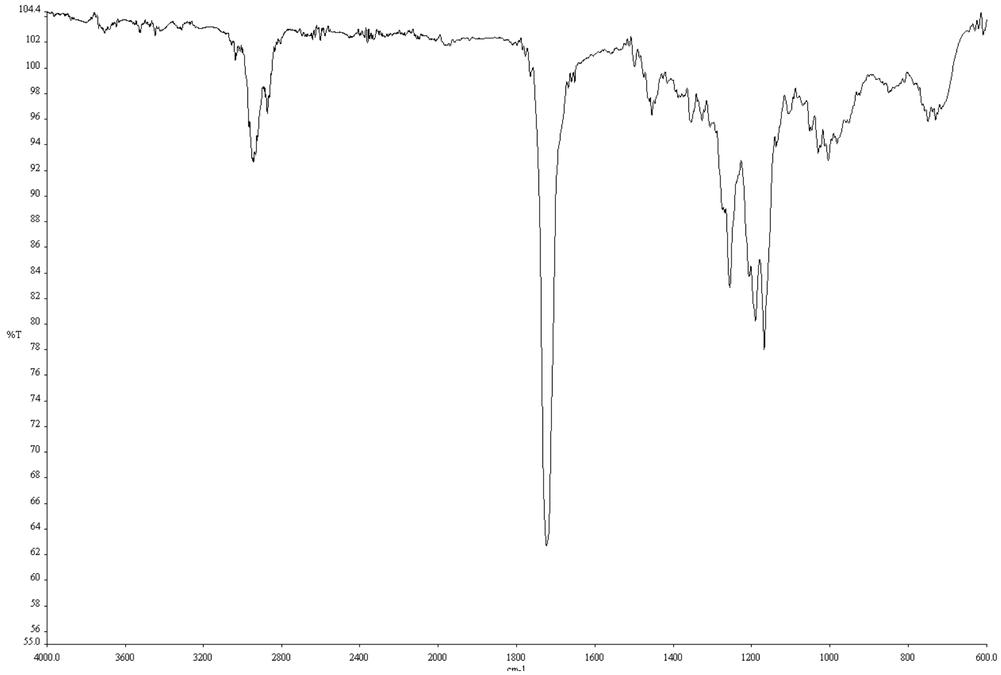

Infrared spectrum (Thin Film, NaCl) of compound **40**.

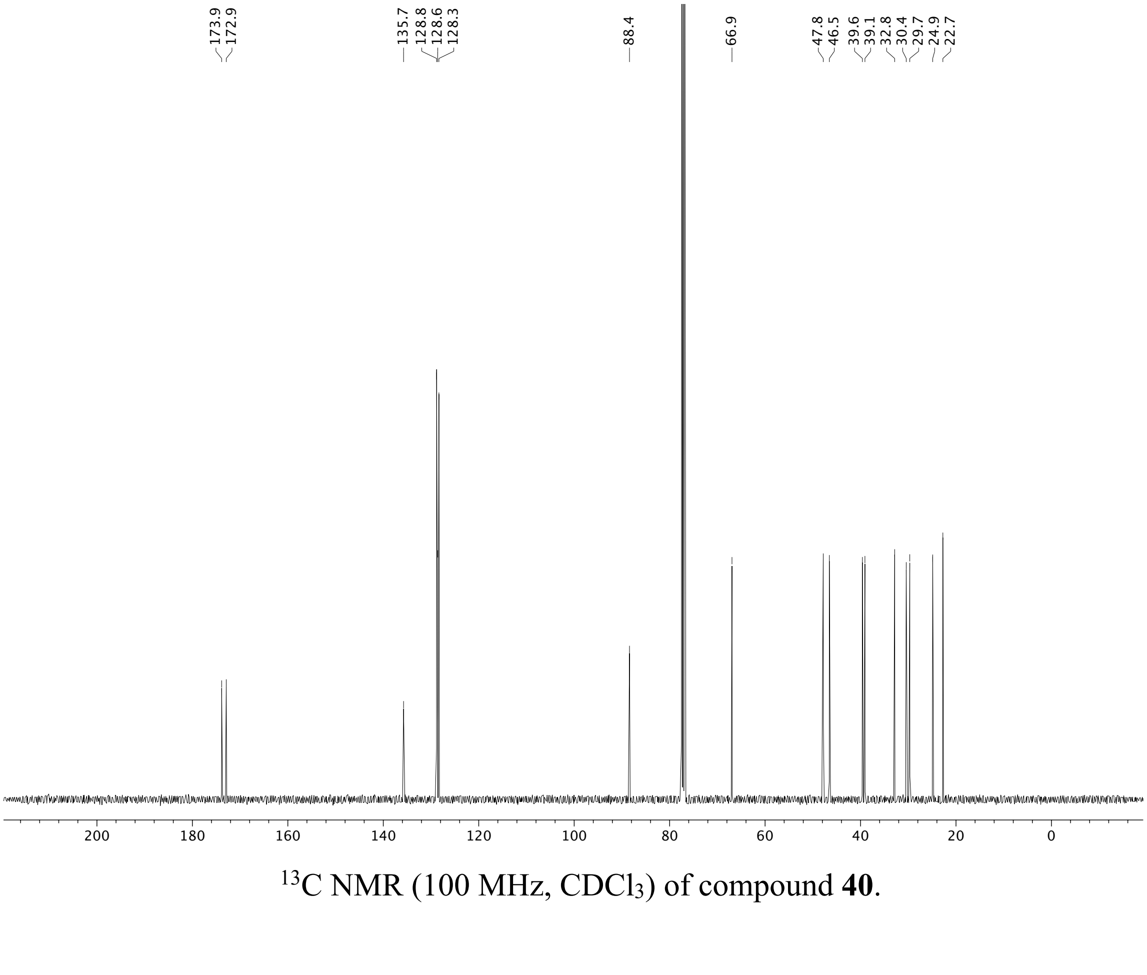

^{13}C NMR (100 MHz, $CDCl_3$) of compound **40**.

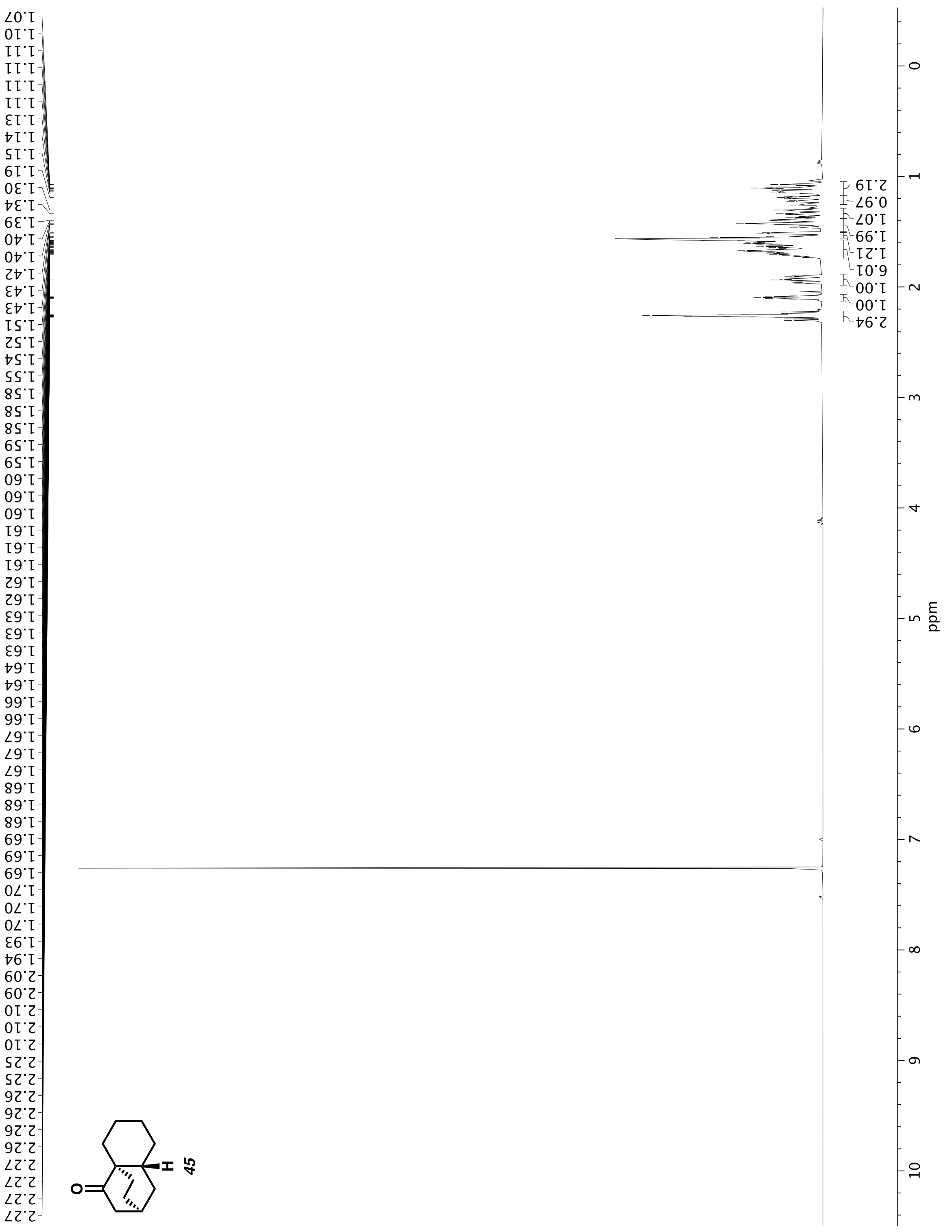

^{1}H NMR (400 MHz, CDCl$_3$) of compound **45**.

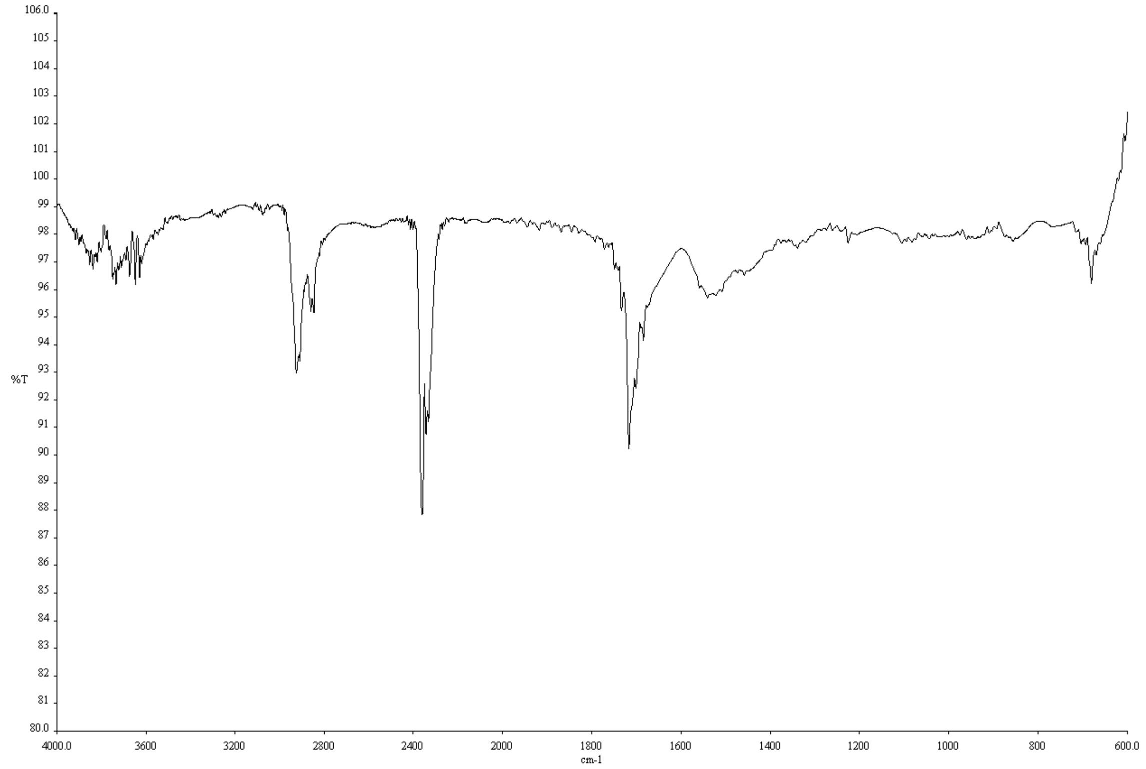

Infrared spectrum (Thin Film, NaCl) of compound **45**.

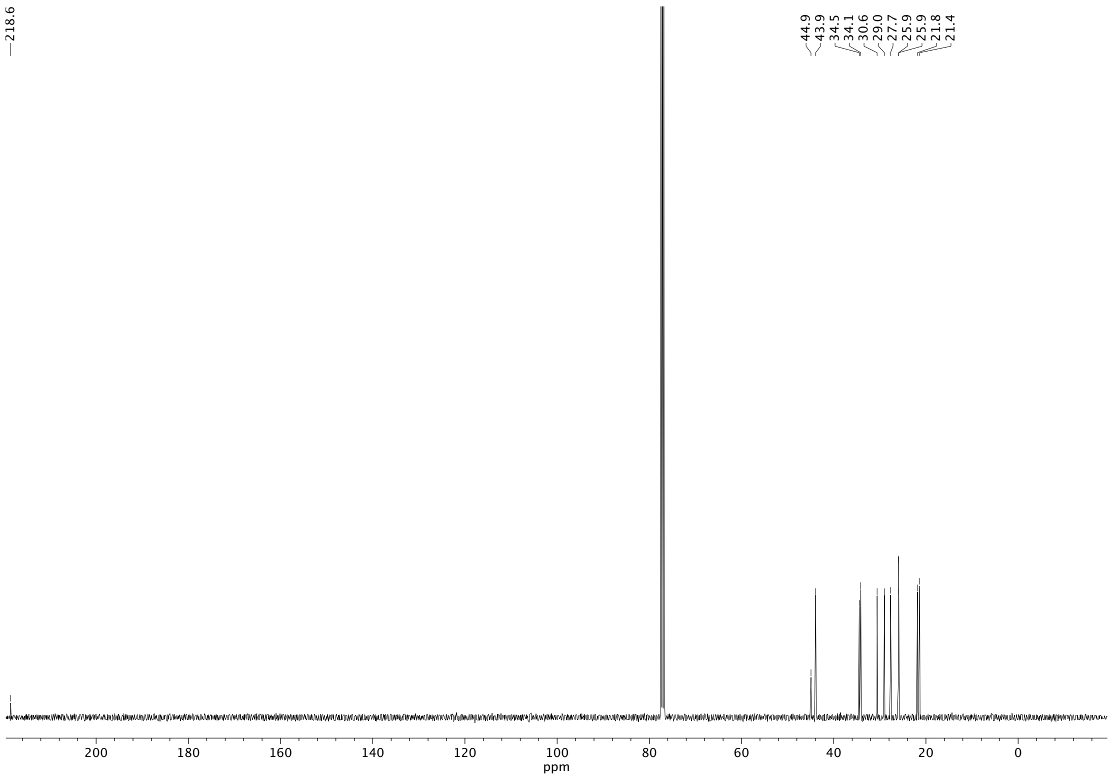

^{13}C NMR (100 MHz, $CDCl_3$) of compound **45**.

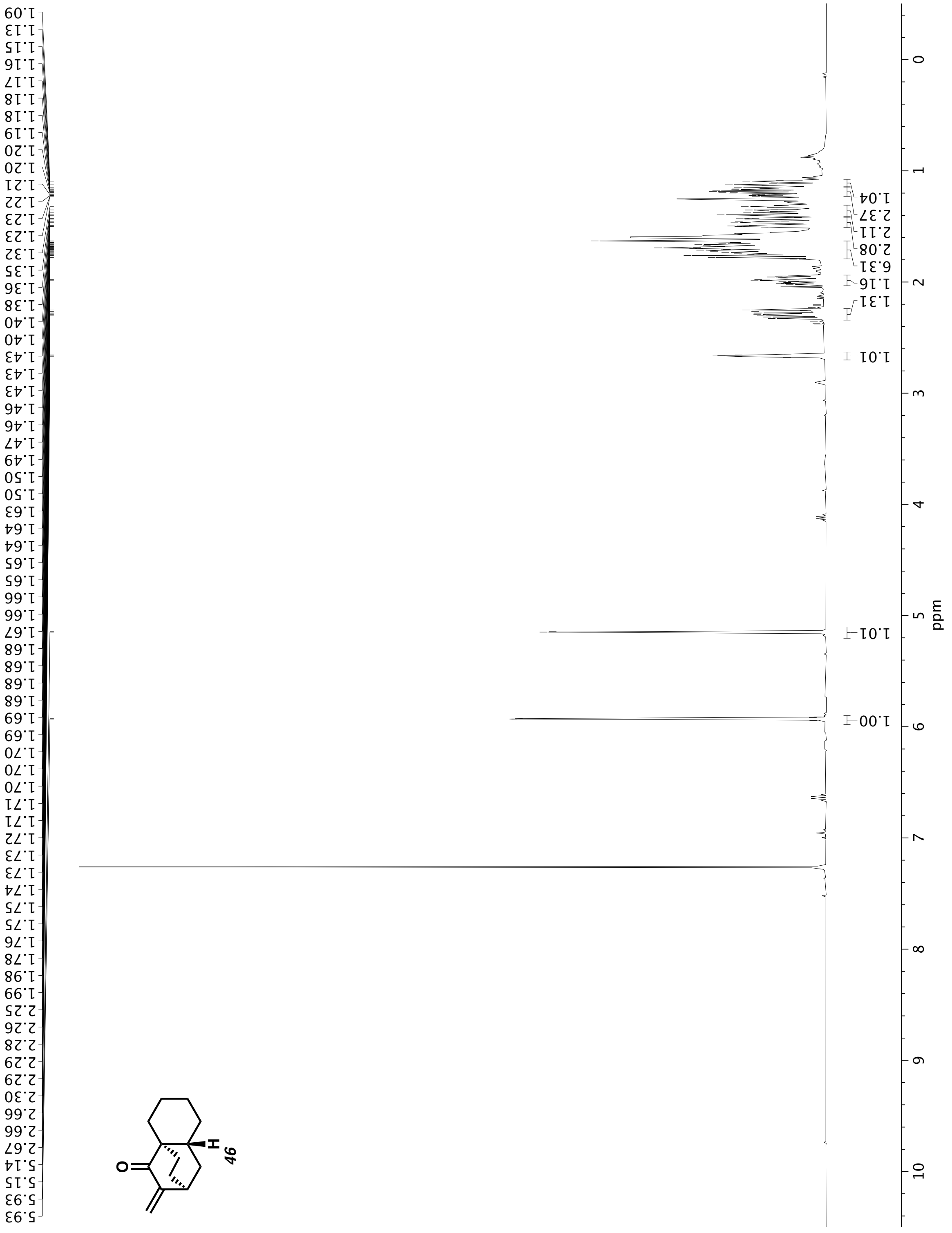

^{1}H NMR (400 MHz, $CDCl_3$) of compound **46**.

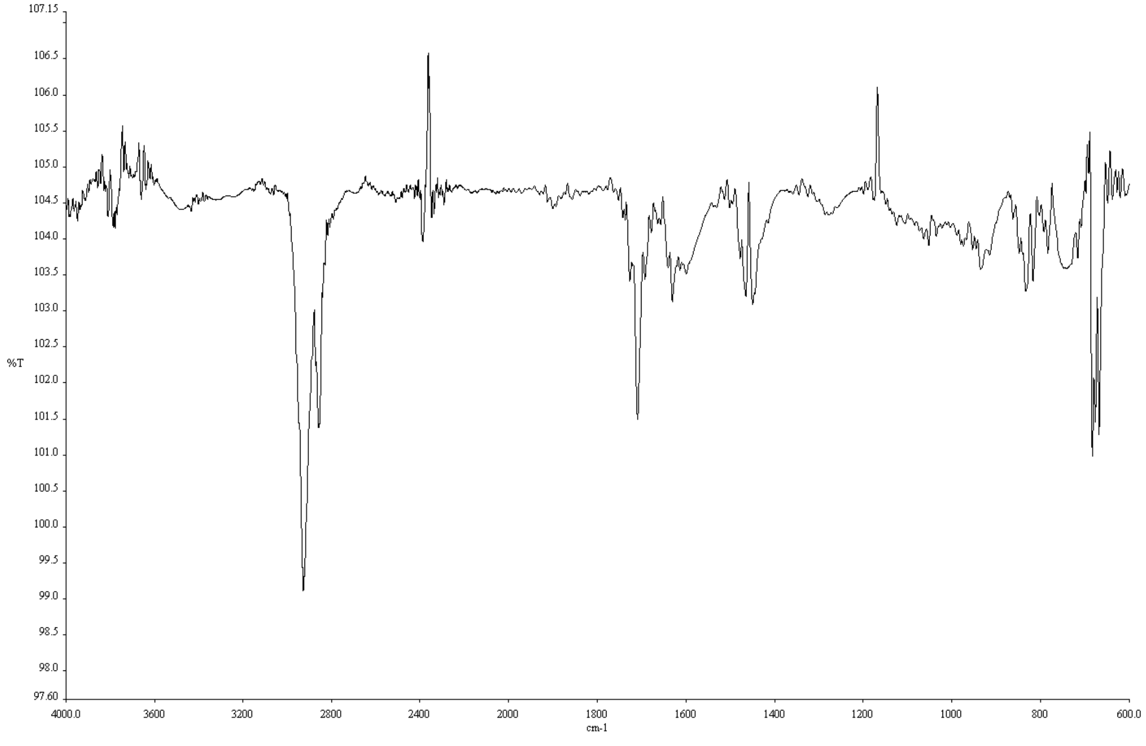

Infrared spectrum (Thin Film, NaCl) of compound **46.**

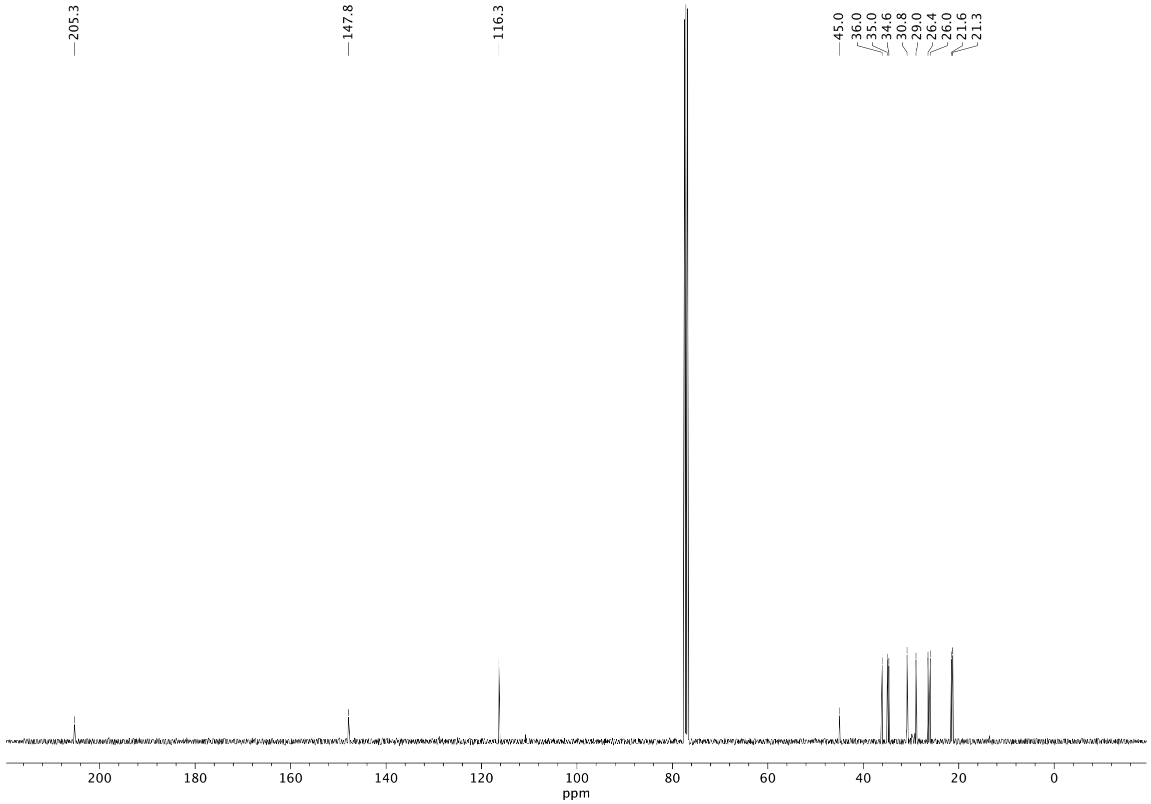

^{13}C NMR (100 MHz, $CDCl_3$) of compound **46**.

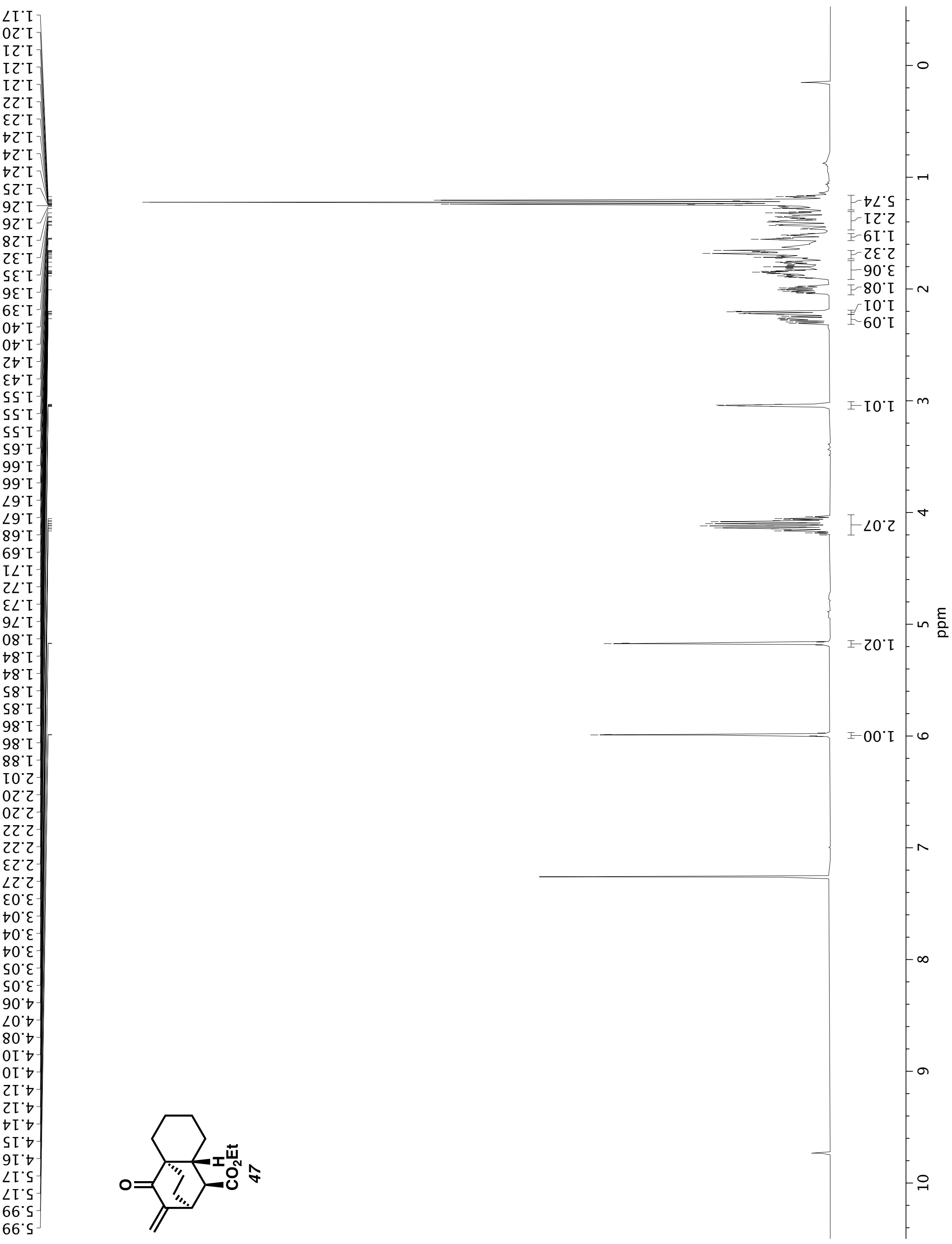

^{1}H NMR (400 MHz, $CDCl_3$) of compound **47**.

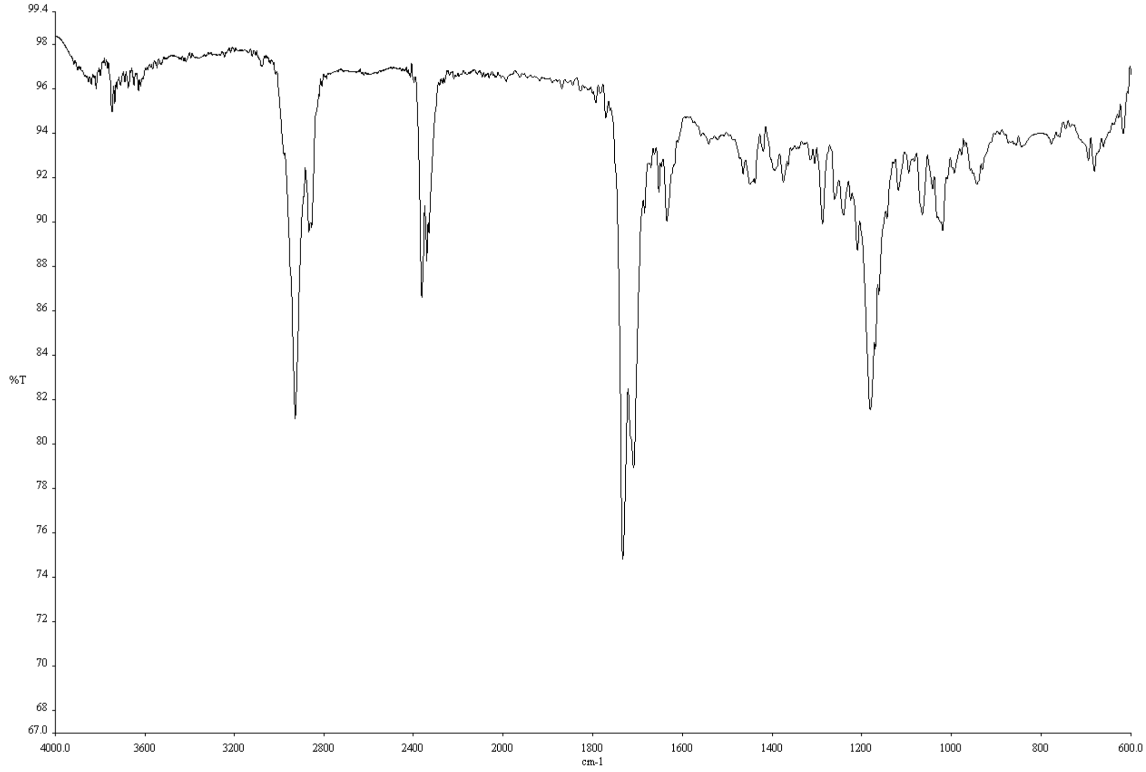

Infrared spectrum (Thin Film, NaCl) of compound **47**.

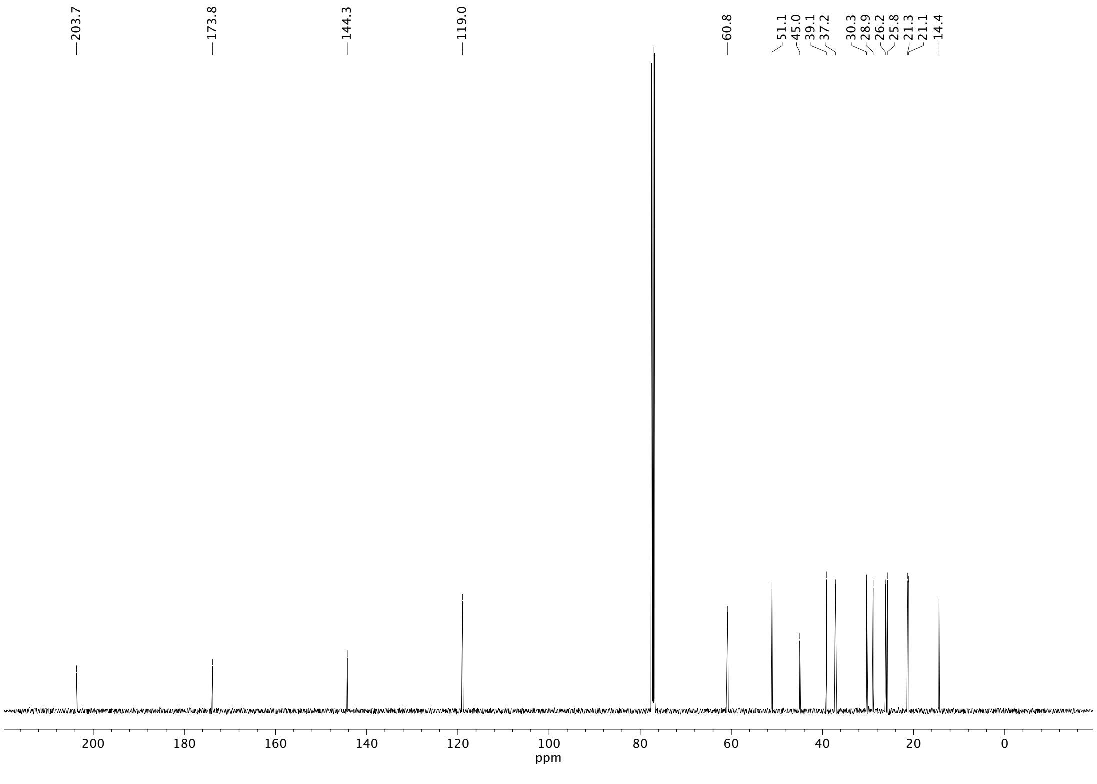

^{13}C NMR (100 MHz, $CDCl_3$) of compound **47**.

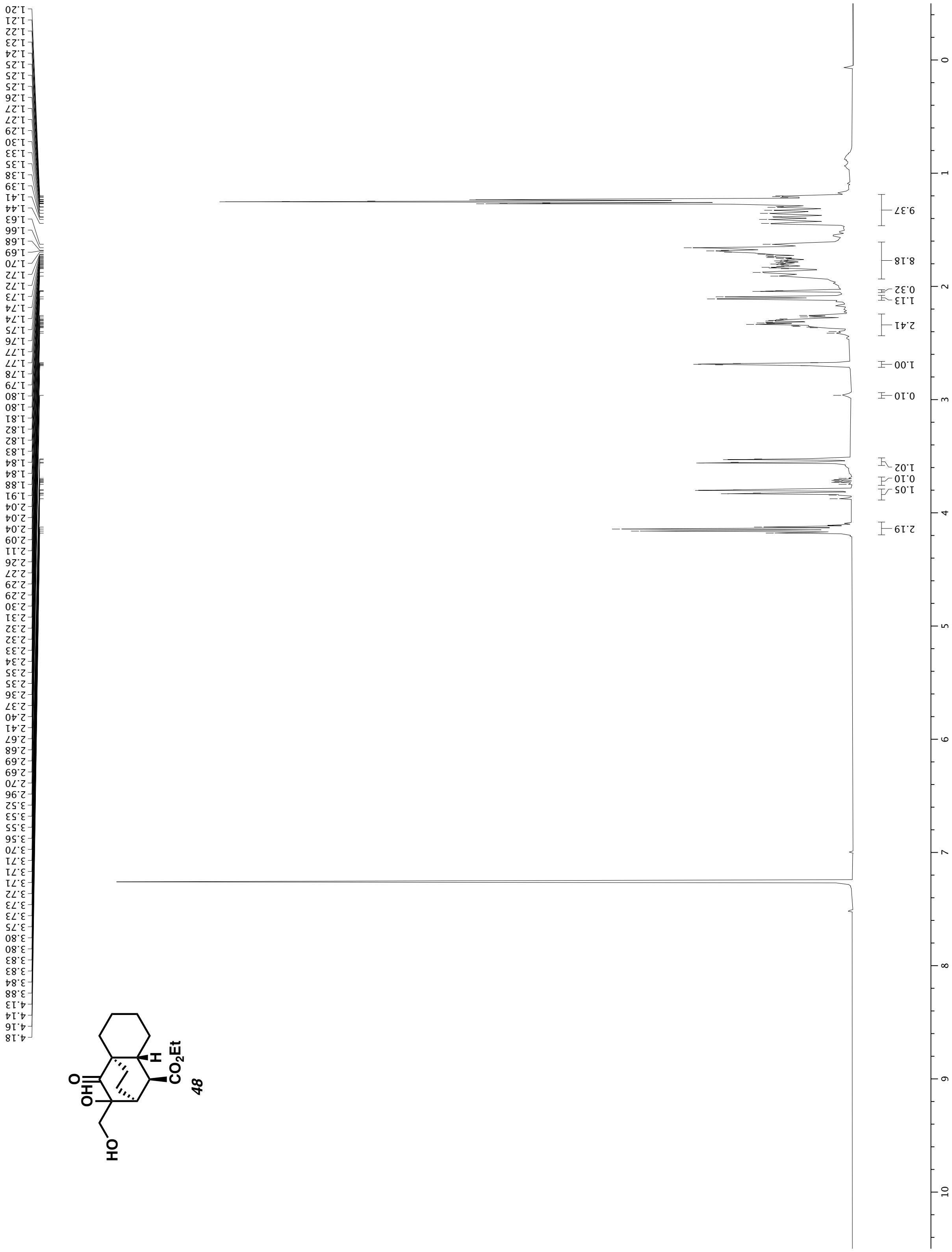

^{1}H NMR (400 MHz, $CDCl_3$) of compound **48**.

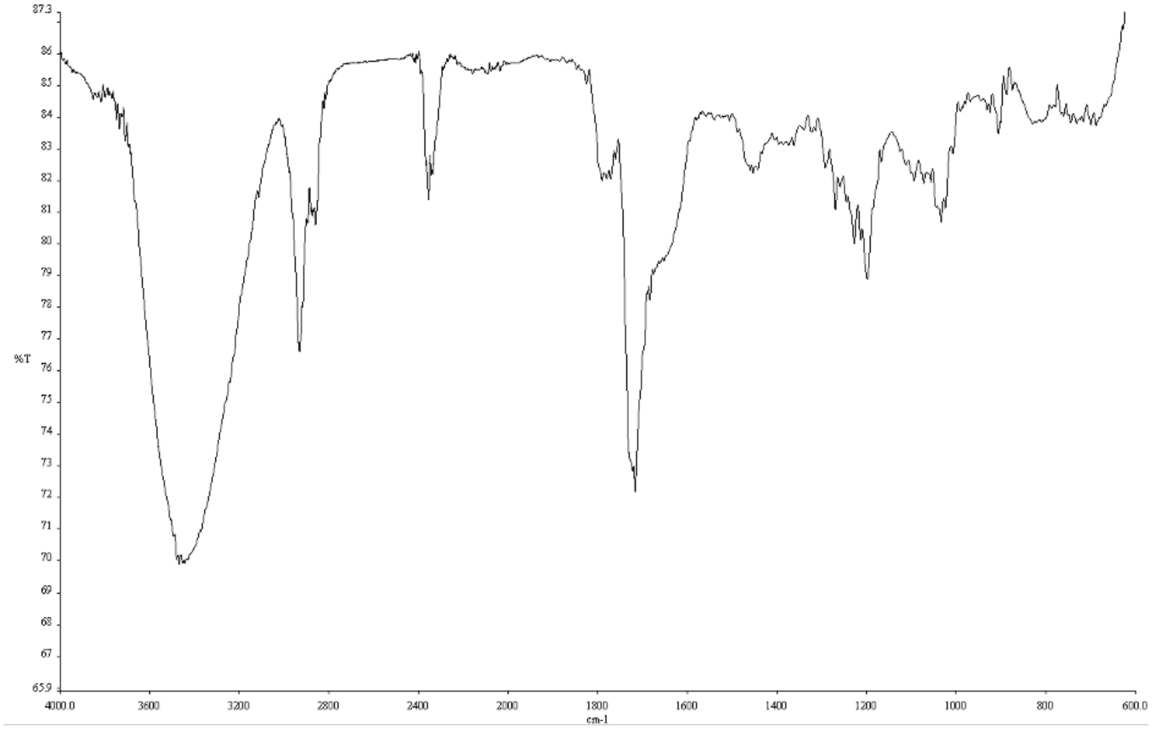

Infrared spectrum (Thin Film, NaCl) of compound **48**.

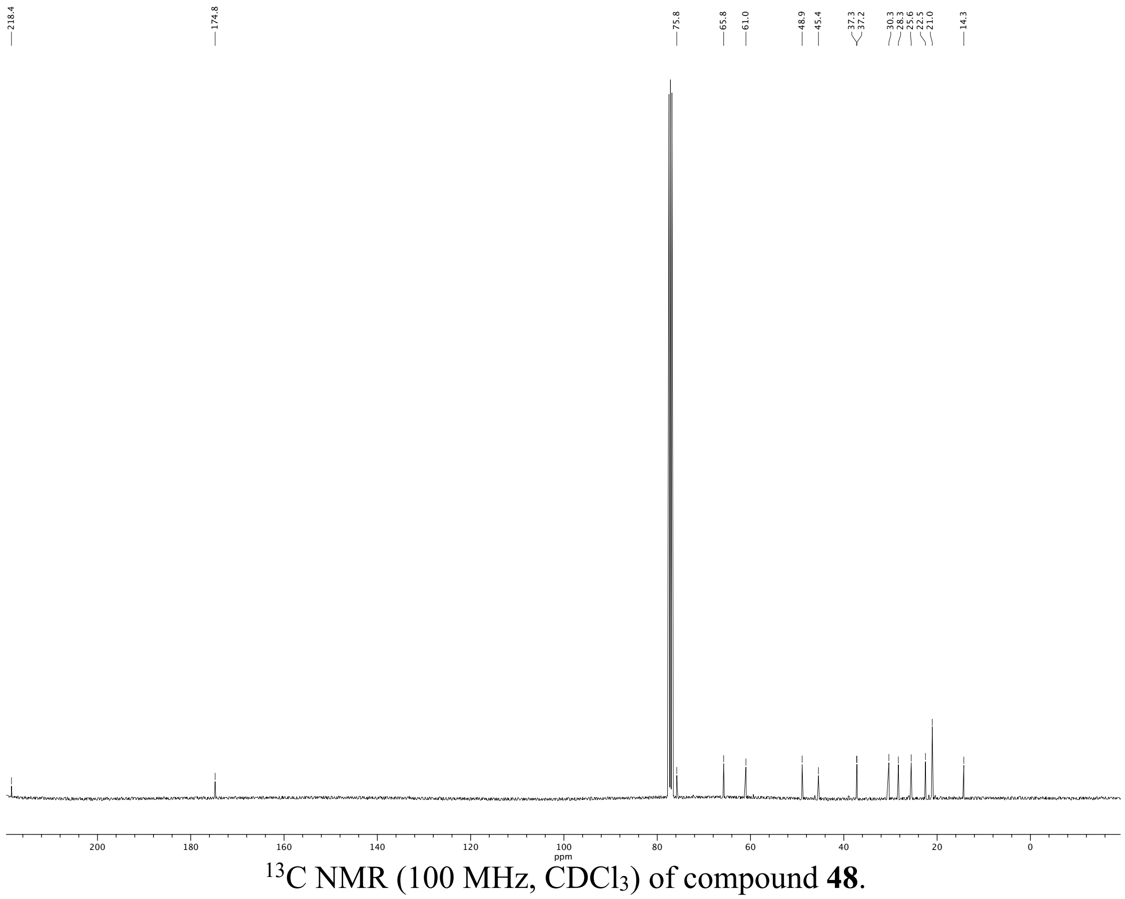

^{13}C NMR (100 MHz, $CDCl_3$) of compound **48**.

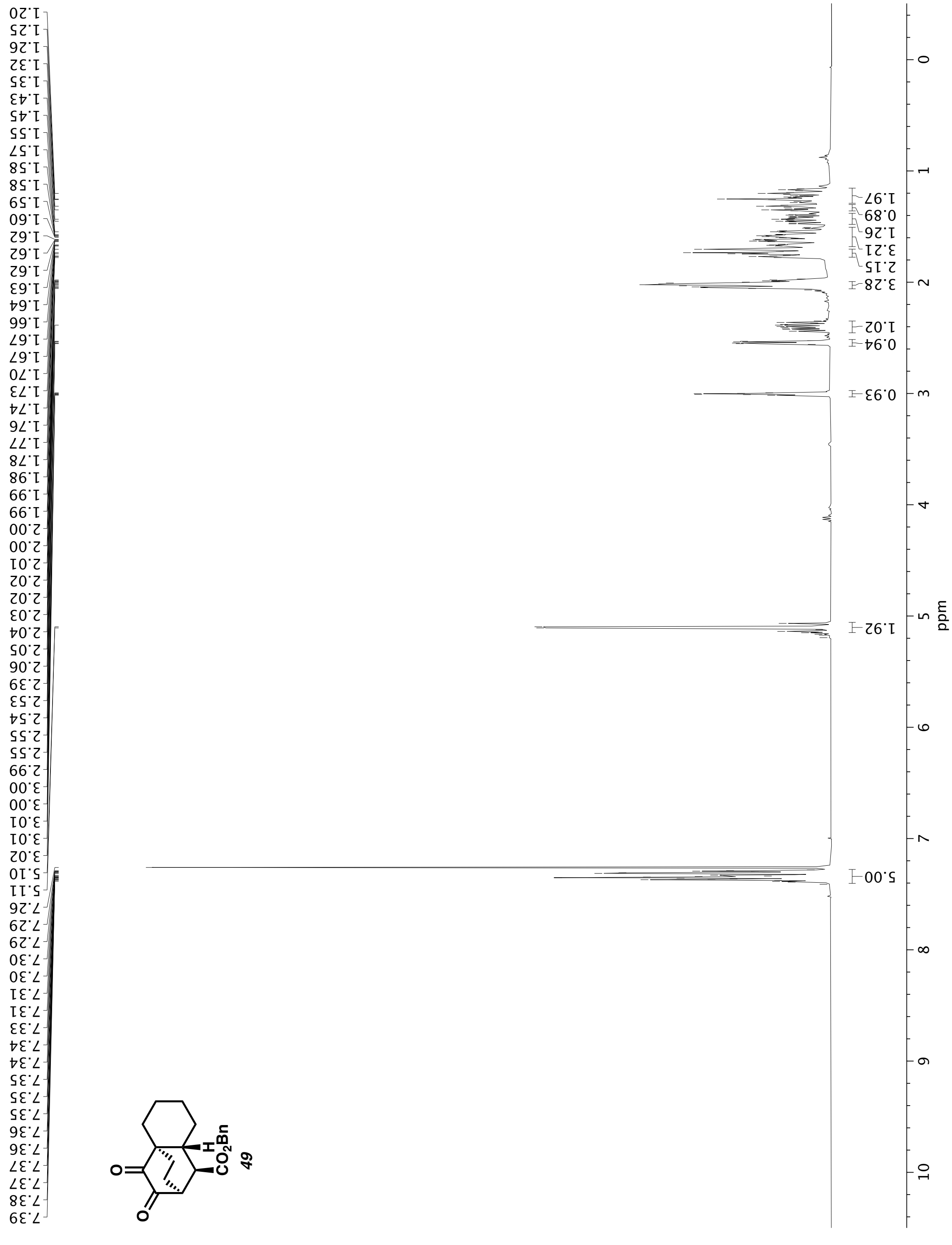

^{1}H NMR (400 MHz, $CDCl_3$) of compound **49**.

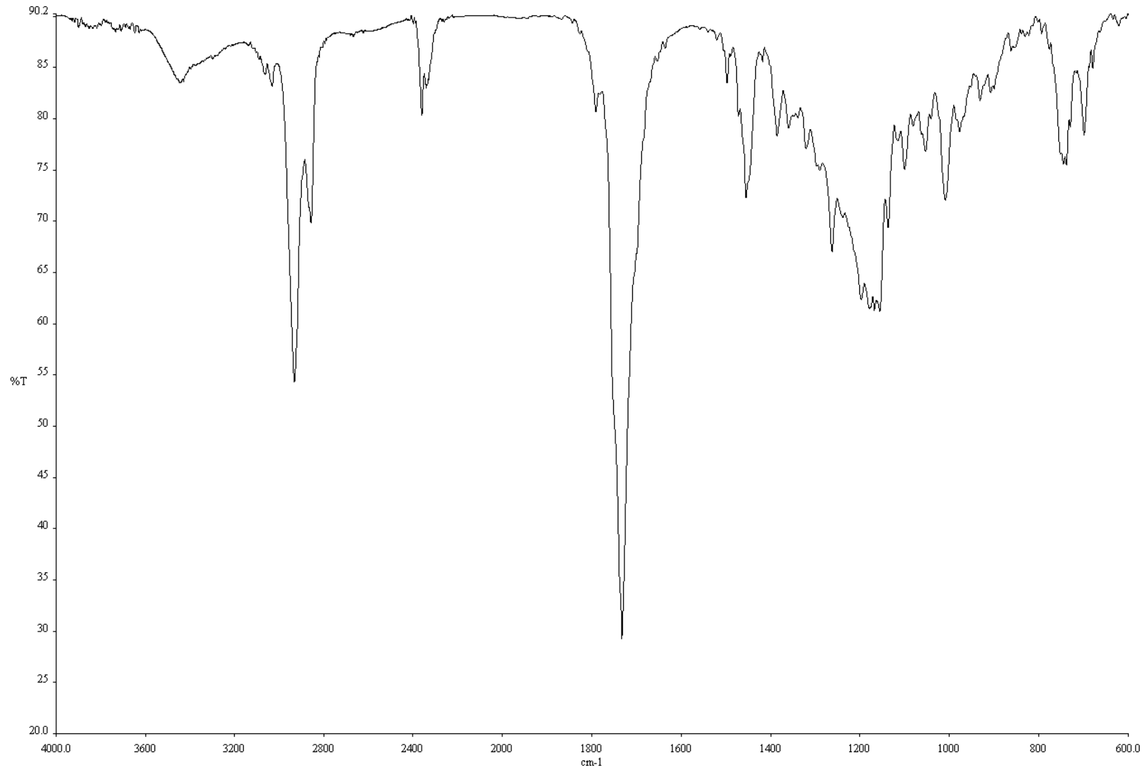

Infrared spectrum (Thin Film, NaCl) of compound **49**.

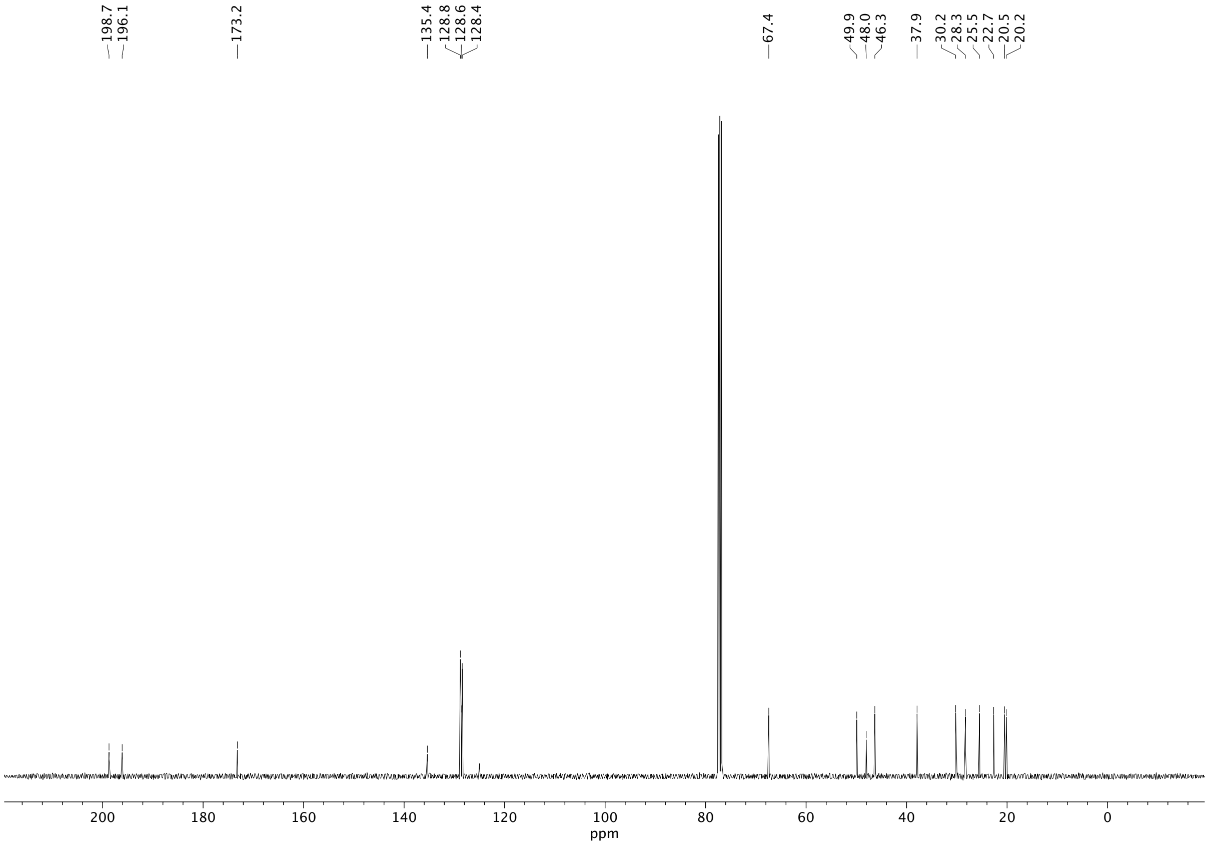

^{13}C NMR (100 MHz, $CDCl_3$) of compound **49**.

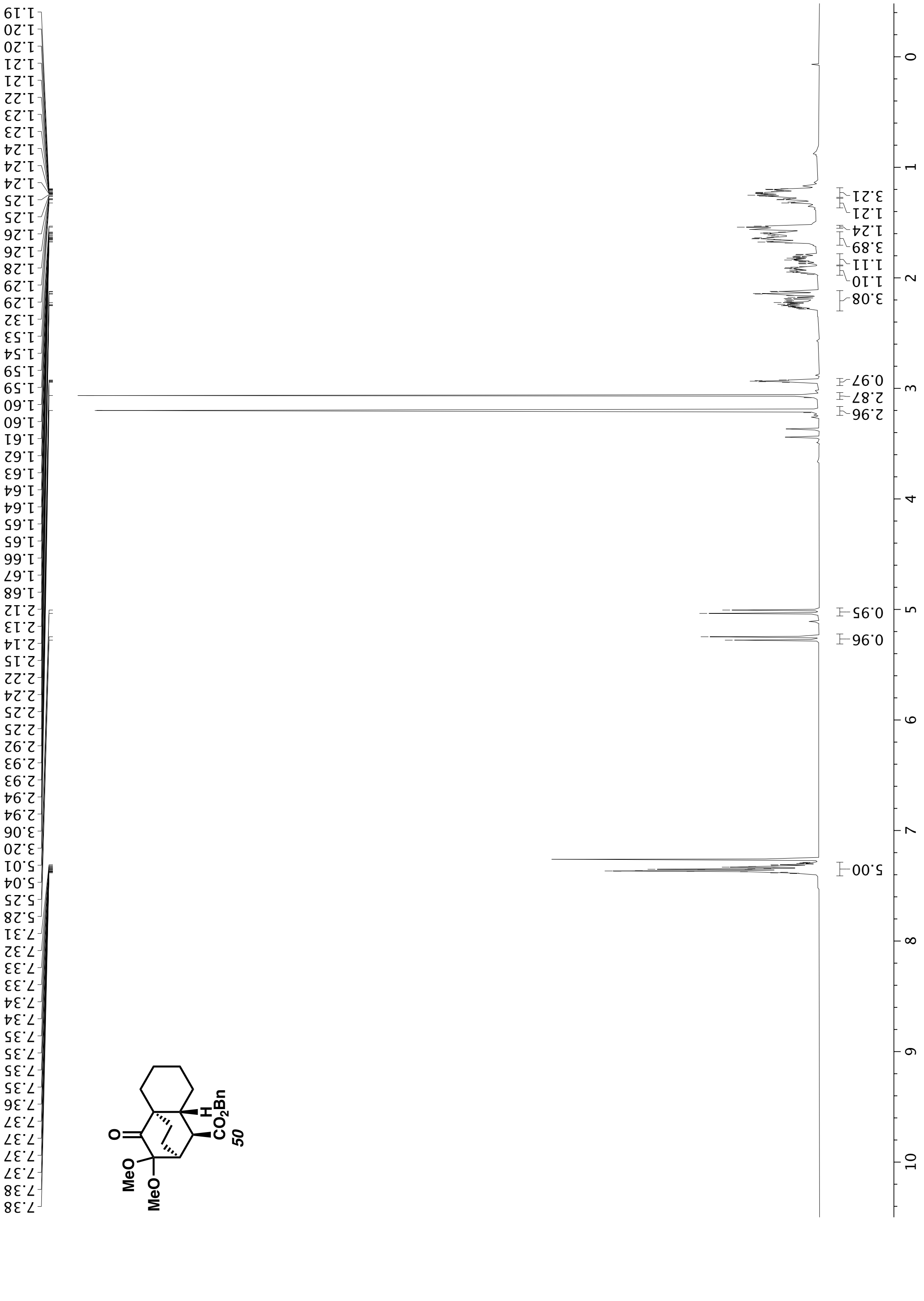

^{1}H NMR (400 MHz, $CDCl_3$) of compound **50**.

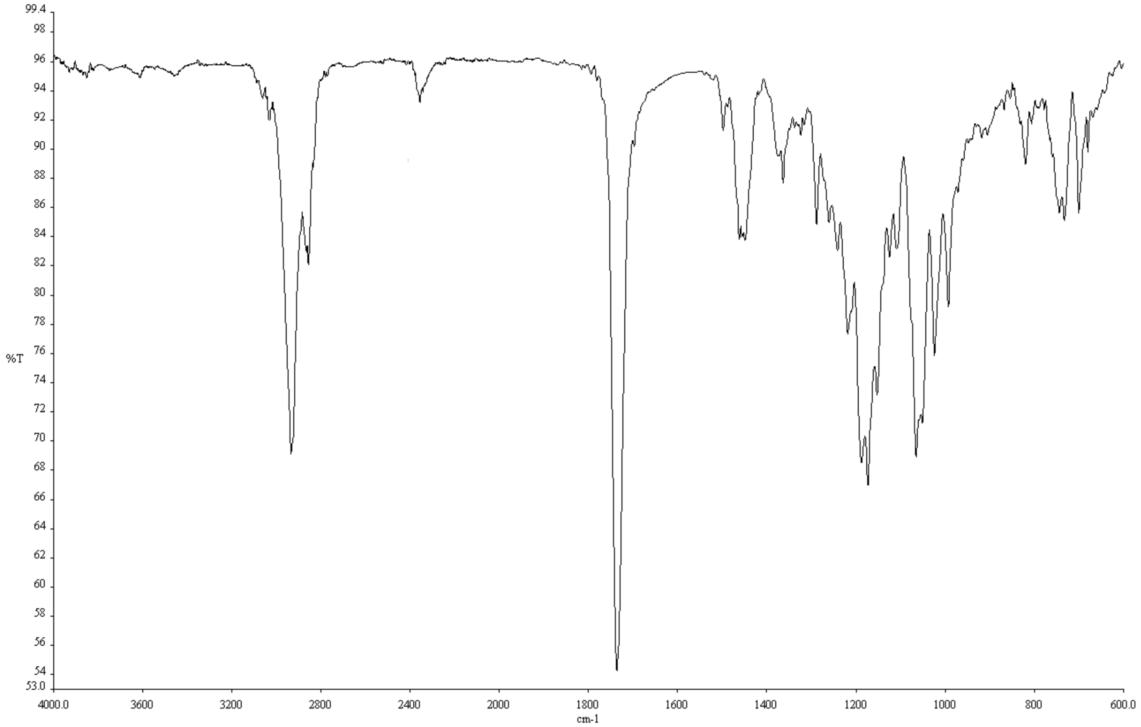

Infrared spectrum (Thin Film, NaCl) of compound **50**.

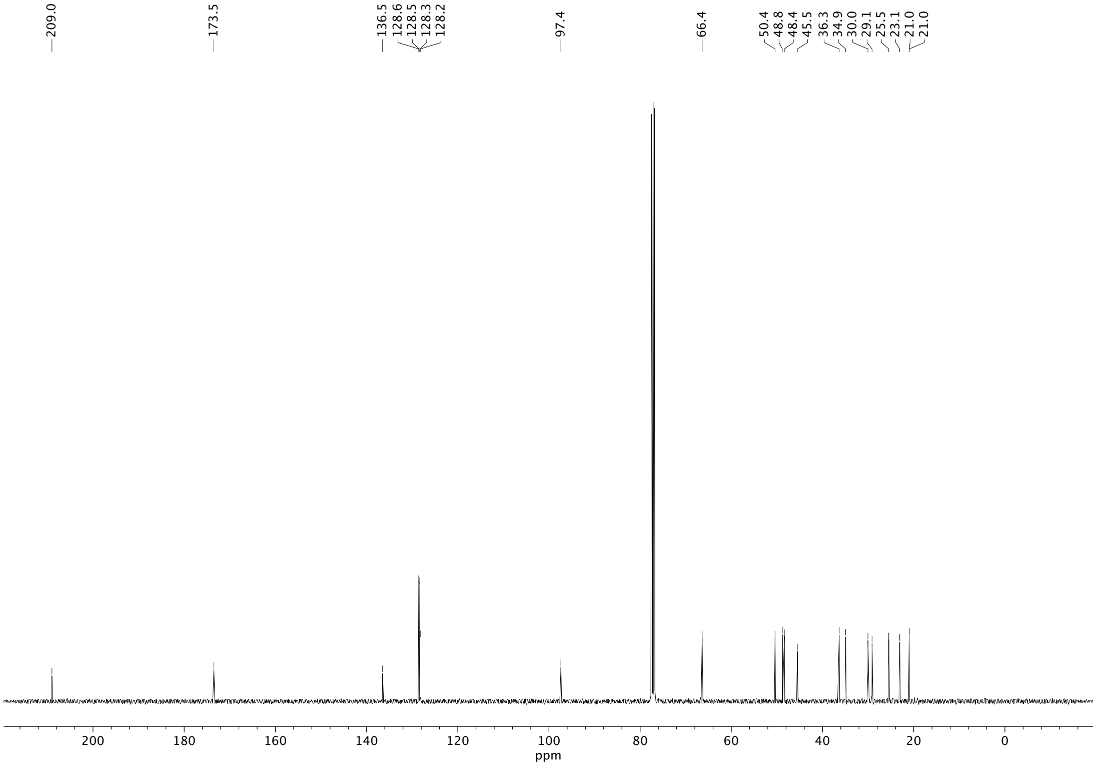

^{13}C NMR (100 MHz, $CDCl_3$) of compound **50**.

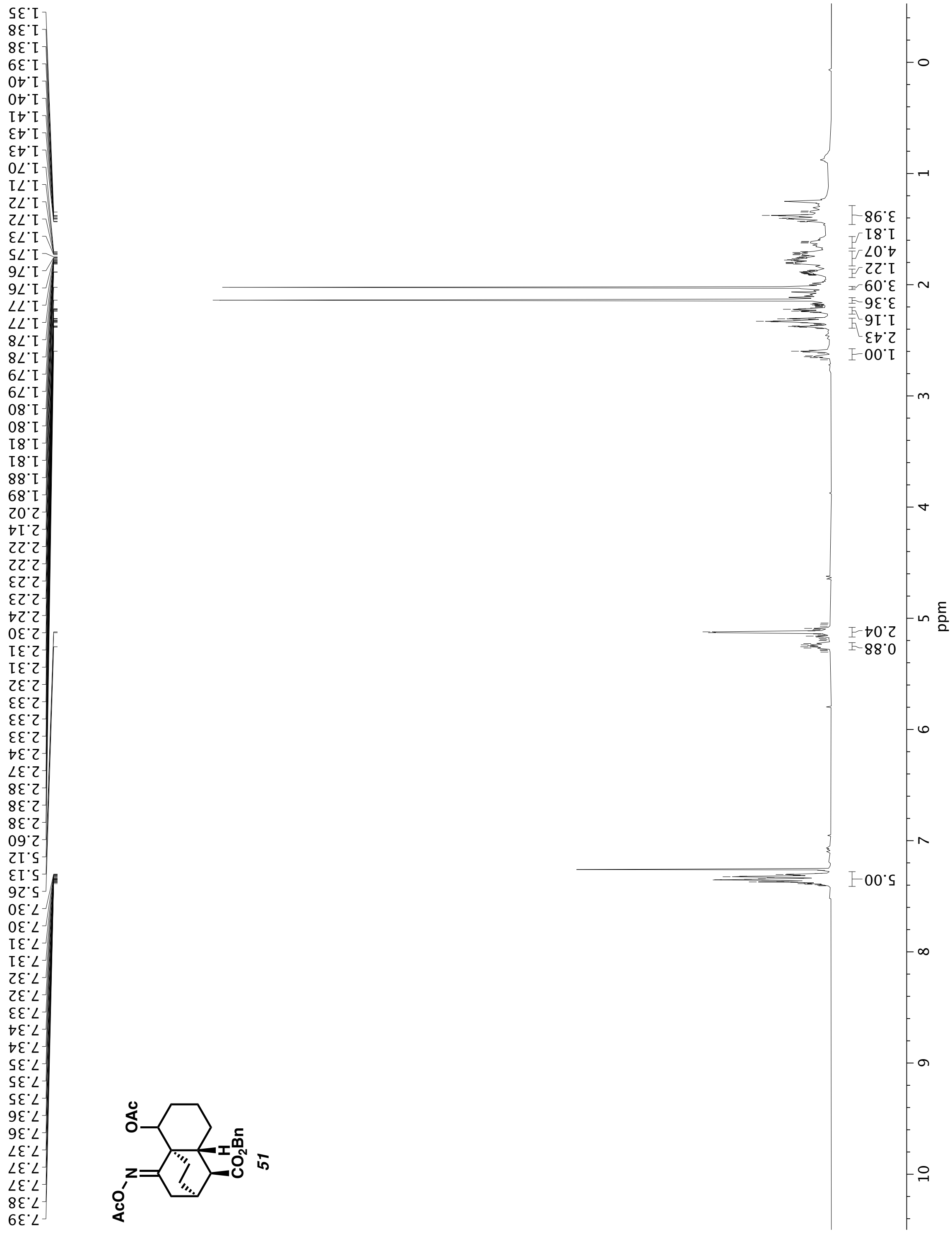

^{1}H NMR (400 MHz, $CDCl_3$) of compound **51**.

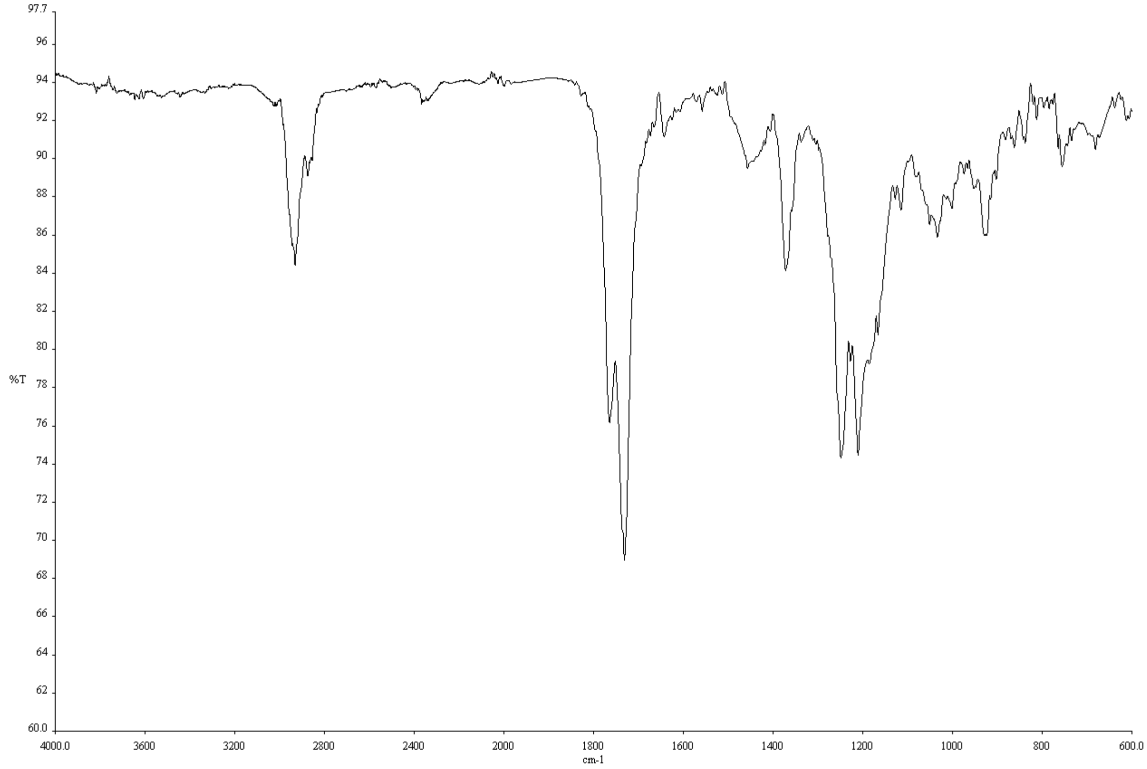

Infrared spectrum (Thin Film, NaCl) of compound **51**.

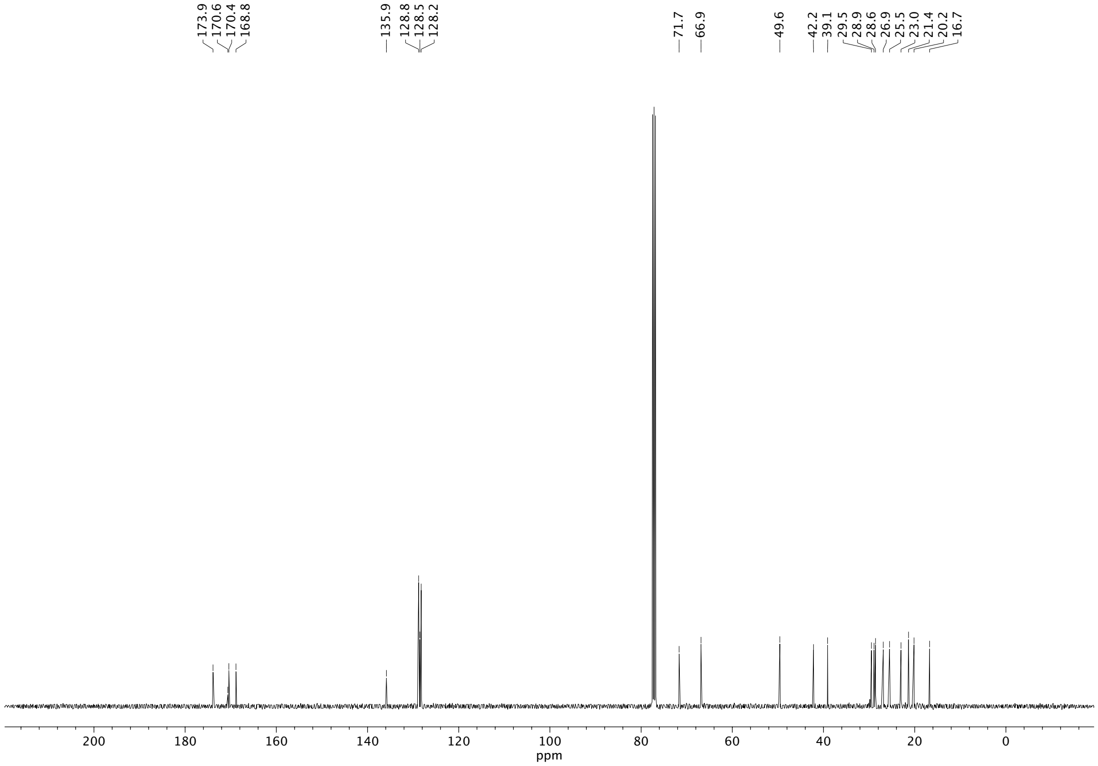

^{13}C NMR (100 MHz, $CDCl_3$) of compound **51**.

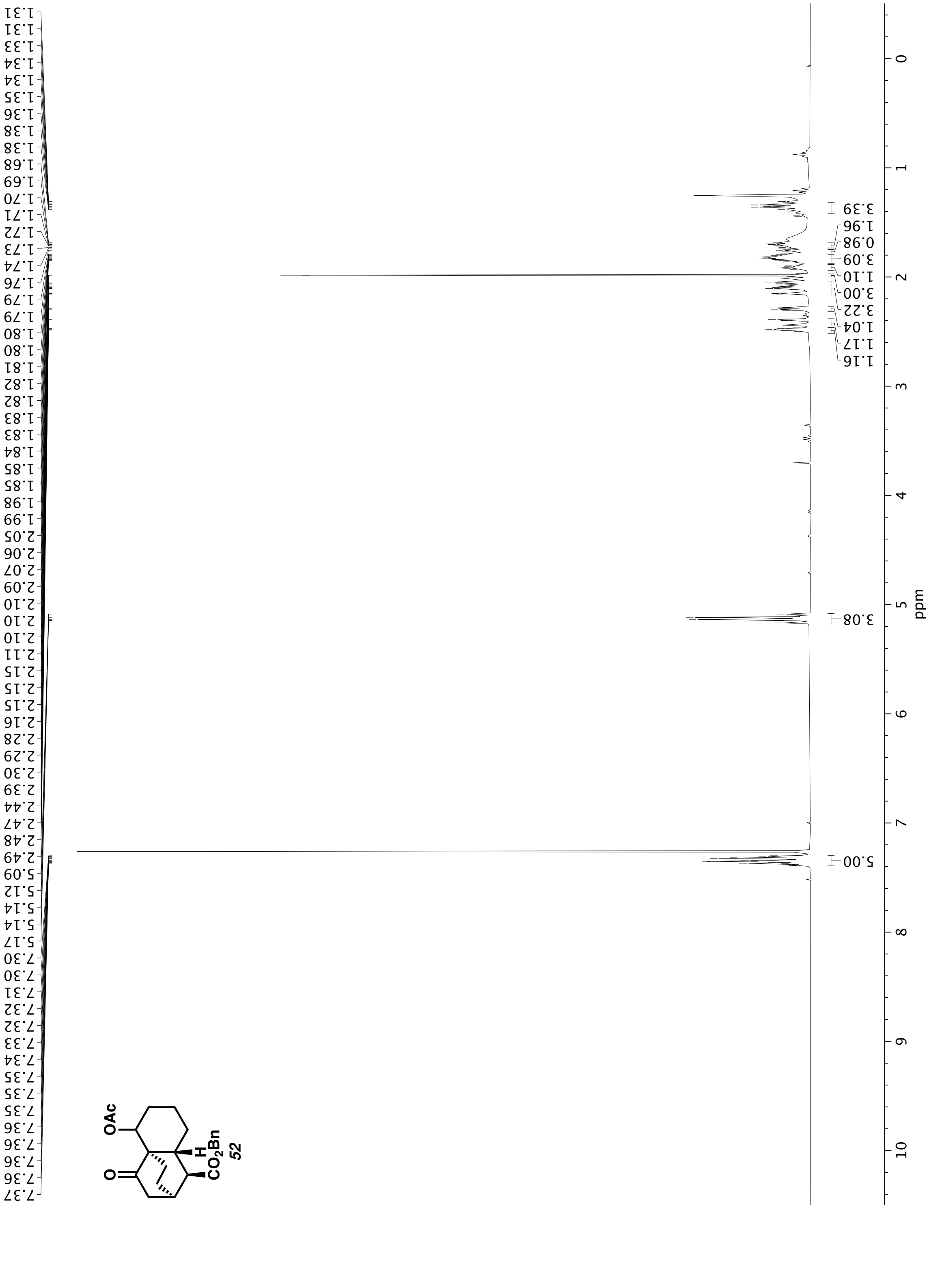

^{1}H NMR (400 MHz, $CDCl_3$) of compound **52**.

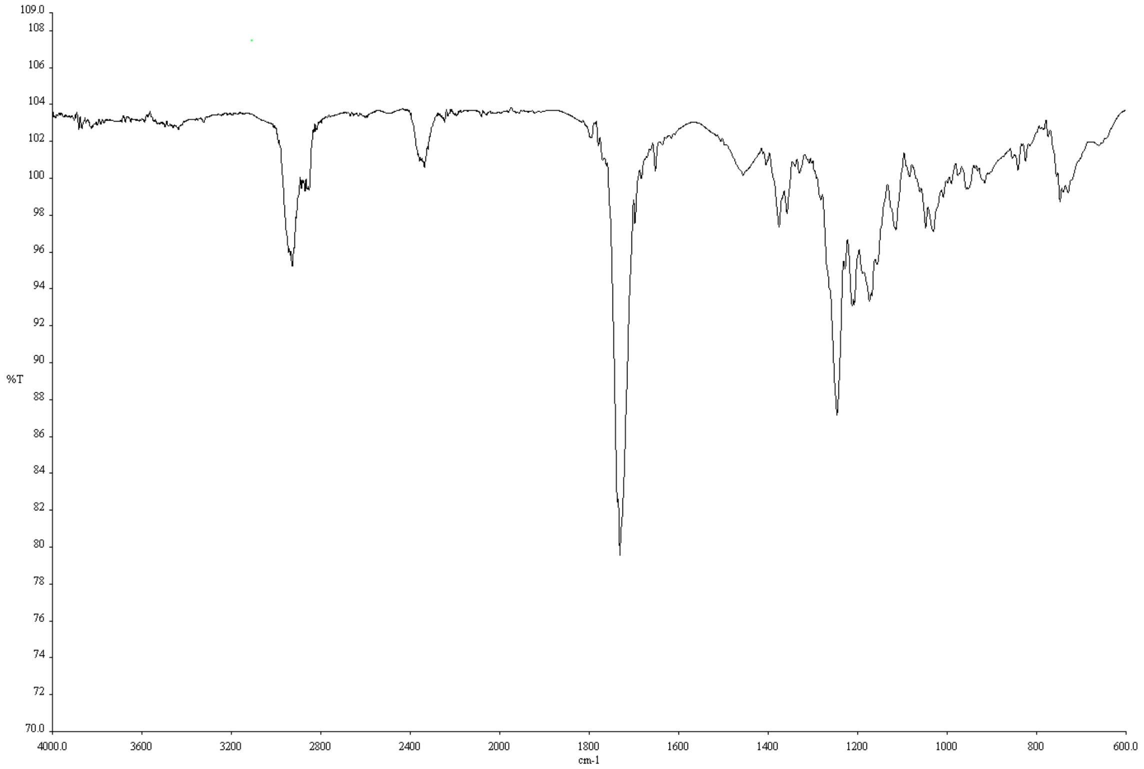

Infrared spectrum (Thin Film, NaCl) of compound **52**.

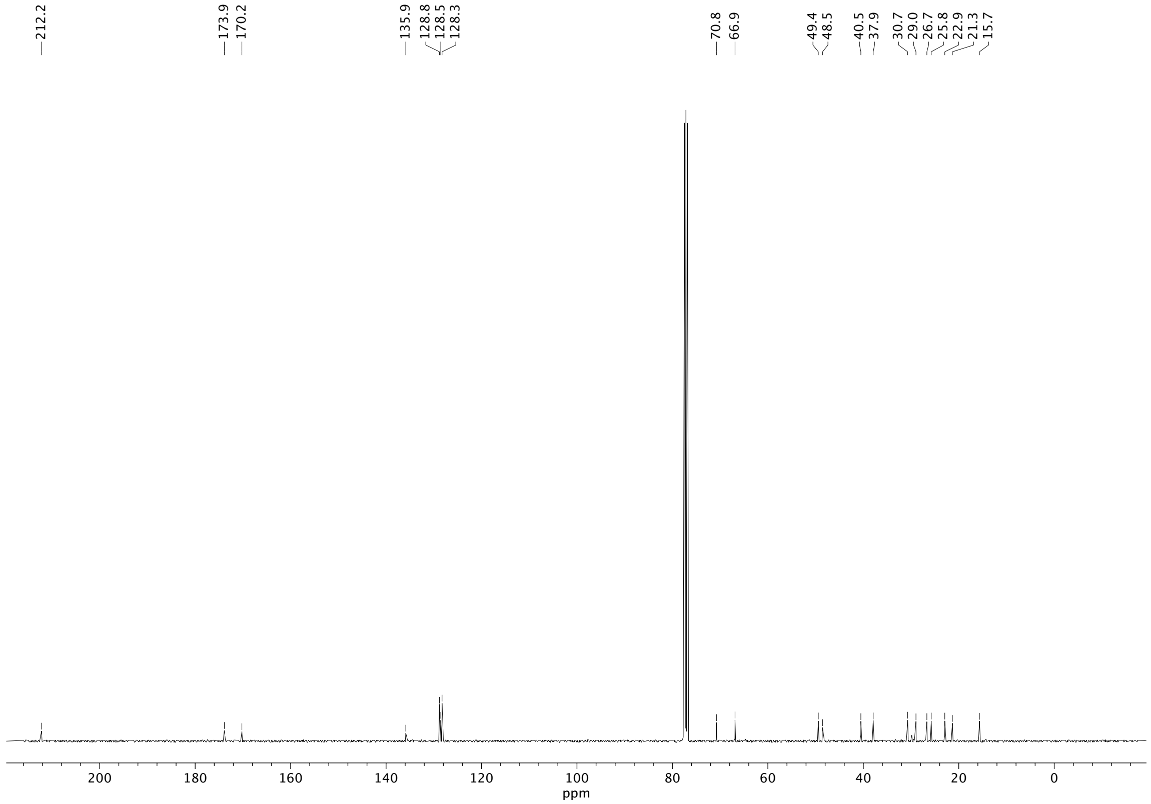

^{13}C NMR (100 MHz, $CDCl_3$) of compound **52**.
